# Access to Diarylmethanols by Wittig Rearrangement of *ortho-*, *meta-*, and *para-*Benzyloxy-*N*-Butylbenzamides

**DOI:** 10.1021/acs.joc.1c03160

**Published:** 2022-03-14

**Authors:** R. Alan Aitken, Andrew D. Harper, Ryan A. Inwood, Alexandra M. Z. Slawin

**Affiliations:** EaStCHEM School of Chemistry, University of St Andrews, North Haugh, St Andrews, Fife KY16 9ST, U.K.

## Abstract

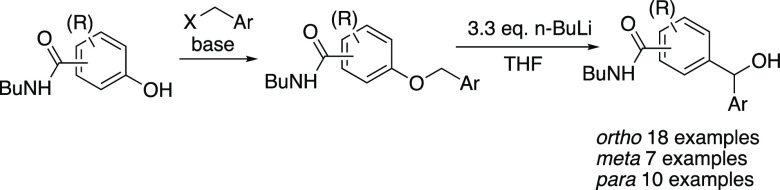

The *N*-butyl amide group, CONHBu, has been found to be an effective promoter of the [1,2]-Wittig rearrangement of aryl benzyl ethers and thus allow the two-step synthesis of isomerically pure substituted diarylmethanols starting from simple hydroxybenzoic acid derivatives. The method is compatible with a wide range of functional groups including methyl, methoxy, and fluoro, although not with nitro and, unexpectedly, is applicable to *meta* as well as *ortho* and *para* isomeric series.

## Introduction

**Scheme 1:**
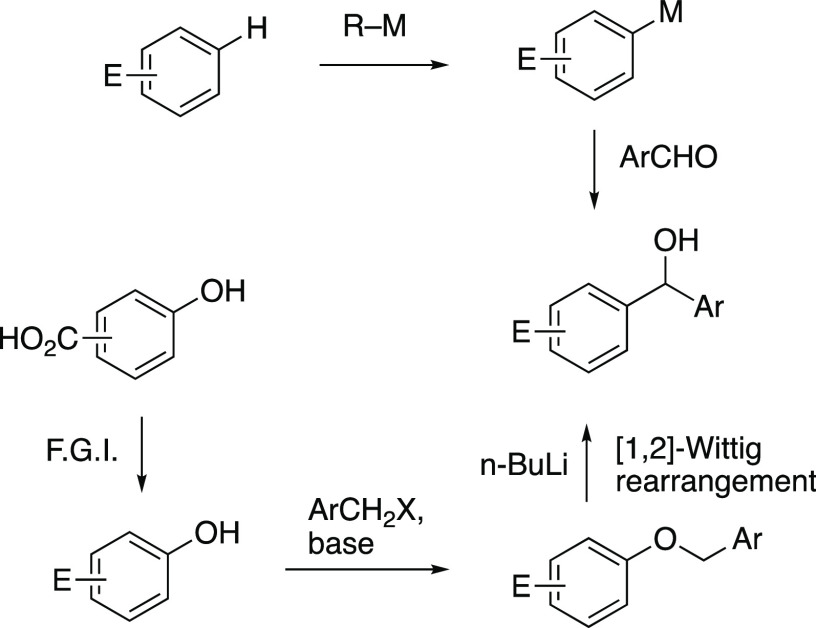
Alternative Approaches to Diarylmethanols

Directed *ortho*-metalation is now well established as a powerful method for regioselective functionalization of aromatic compounds,^1^ and subsequent reaction with an electrophile such as an aromatic aldehyde allows facile construction of *ortho*-substituted diarylmethanols (Scheme 1). In contrast, methods for the analogous *meta-* or *para*-functionalization are nowhere near so well developed despite some recent progress.^2^ In view of the low cost and ready availability of the three isomeric hydroxybenzoic acids, an attractive alternative strategy to access specifically substituted diarylmethanols would be to transform the carboxylic acid into a suitable activating group, *O*-benzylate the phenolic OH, and then conduct a [1,2]-Wittig rearrangement (Scheme 1). Although this is a well-known aromatic rearrangement,^3^ it has not been widely exploited in synthesis, most likely due to the strongly basic conditions required, which limit functional group compatibility, and there have been few recent examples of its use.^4^ Some highlights in recent use of the Wittig rearrangement include rearrangement of benzyl butadienyl ethers,^5^ benzyl pyridyl ethers,^6^ tandem anion translocation—Wittig rearrangement and tandem Wittig rearrangement—aldol reaction,^7^ as well as the study of systems where there is competition between 1,2- and 2,3- or 1,2- and 1,4-Wittig rearrangements.^8^ The Wittig rearrangement has also been used to access a range of chiral binaphthyl ligands that have been applied in asymmetric catalysis.^9^ The rearrangement has been carried out in an enantioselective way by adding a chiral bis(oxazoline) catalyst,^10^ and diastereoselective Wittig rearrangements have been reported directed by adjacent carbohydrate and α-alkoxyamide functions.^11^ Finally, it is also possible to suppress the Wittig rearrangement if desired in order to make use of the unrearranged α-lithiobenzyloxy group in the synthesis of benzofurans and other heterocycles.^12^ In this paper, we describe the discovery and development of the facile Wittig rearrangement of isomeric benzyloxy-*N*-butylbenzamides to furnish the corresponding diarylmethanols in the *ortho*-, *meta*-, and *para*-series.

## Results and Discussion

**Scheme 2:**
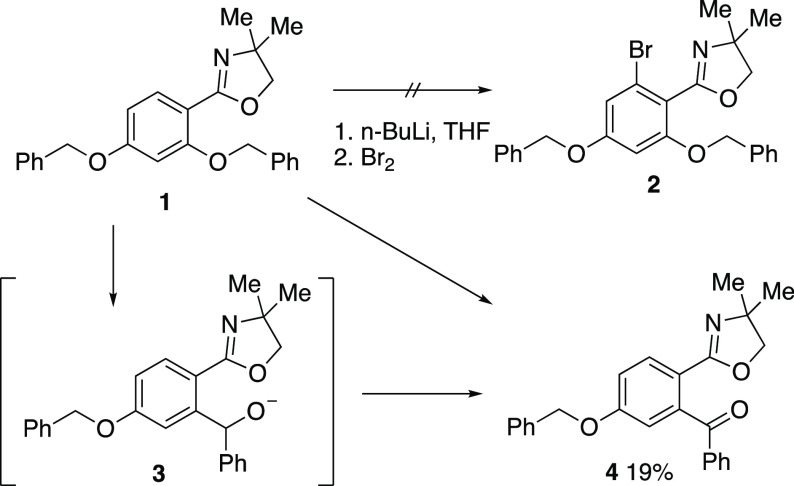
Unexpected Reaction Leading to **4**

Our entry into this area came from a serendipitous discovery during attempted ring bromination of the 2,4-bis(benzyloxy)phenyloxazoline **1**. Treatment with *n-*butyllithium followed by bromine gave not the expected product **2** but instead a product identified by spectroscopic methods and X-ray diffraction (see Supporting Information) as **4**, presumably formed by Wittig rearrangement to afford the intermediate **3**, which was then oxidized by bromine^13^ to the ketone (Scheme 2).

**Scheme 3:**
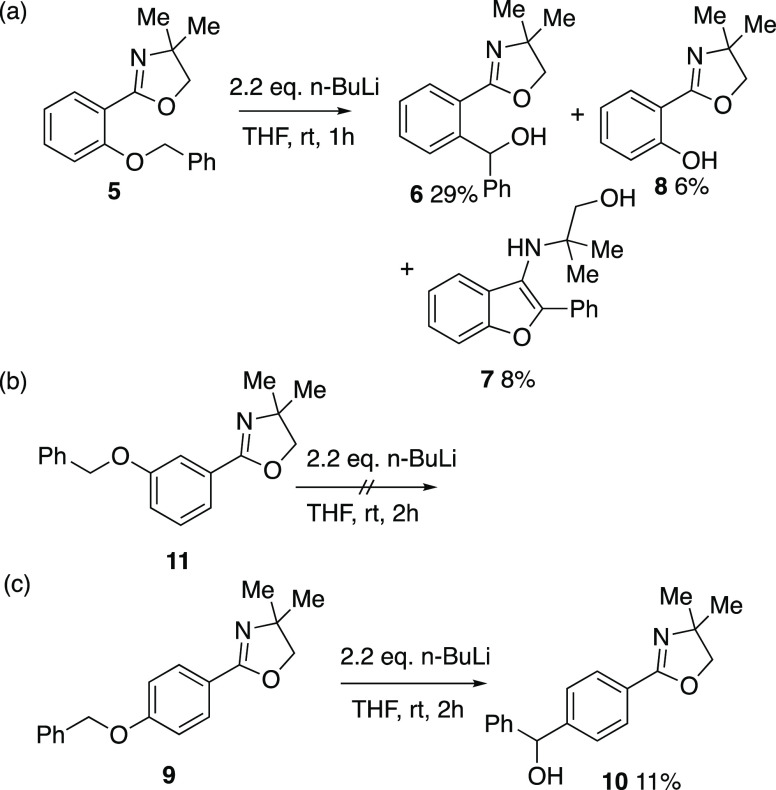
Behavior of Mono(benzyloxy)phenyloxazolines

We then examined the reactivity of the simpler 2-benzyloxyphenyl compound **5** and found that, depending upon the reaction conditions, varying mixtures of the Wittig rearrangement product **6**, the 3-aminobenzofuran **7** resulting from intramolecular nucleophilic ring opening of the oxazoline by the benzyl anion, and the *O*-dealkylation product **8** were formed (Scheme 3). As we have already reported elsewhere,^14^ the process could be optimized toward the formation of **7** using *n*-butyllithium/potassium *tert*-butoxide. However, optimized conditions for the formation of **6**, namely 2.2 equiv *n*-BuLi in THF at rt for 1 h, resulted in an isolated yield after chromatographic purification of just 29%. Under the same conditions, the *para*-isomer **9** gave the rearrangement product **10** but in only 11% isolated yield, and the *meta*-isomer **11** was recovered unchanged. It was clear that the 4,4-dimethyloxazoline group was not an optimal promoter of the Wittig rearrangement.

**Scheme 4:**
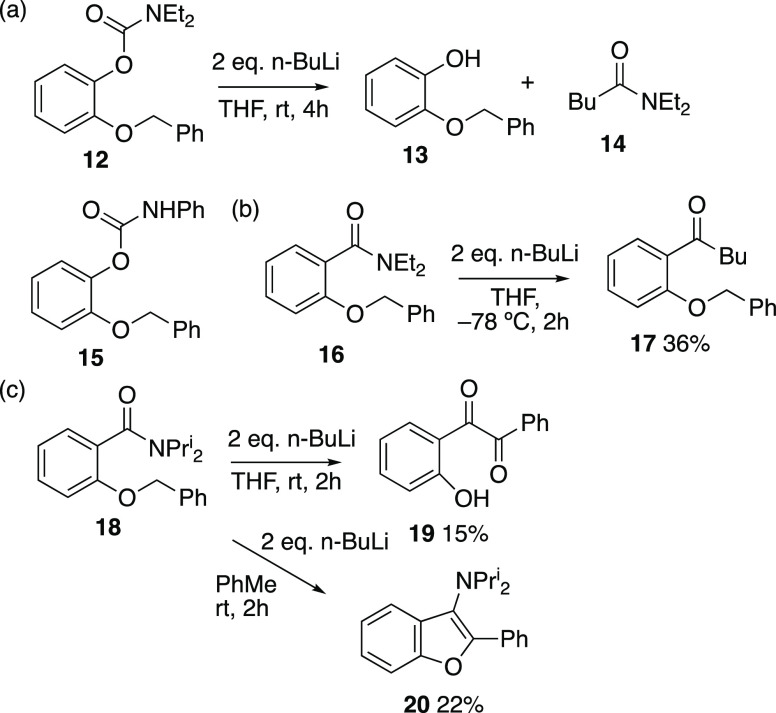
Reaction of Carbamates and Tertiary Benzamides

We next investigated carbamates and tertiary amides, both classic *ortho*-directing groups,^1^ as promoters of Wittig rearrangement, but this was uniformly unsuccessful. The diethylcarbamate **12** suffered a nucleophilic attack at carbonyl to give **13** and **14** in low yield while the phenylcarbamate **15** was recovered unchanged (Scheme 4). The *N,N*-diethyl amide **16** reacted at the carbonyl group to give the ketone **17**. In a previous case where an undesired attack of butyllithium at a diethyl amide was encountered,^15^ this could be suppressed by changing to the *N,N*-diisopropyl amide but the reaction of **18** took a different course, giving 2-hydroxybenzil **19** in THF, but the 3-aminobenzofuran **20** in toluene. There are only a few other synthetic routes to substituted 3-aminobenzofurans. The formation of both these products involves an initial intramolecular nucleophilic attack of benzyloxy anion on the amide carbonyl with hydrolysis and dehydration giving **20**, while loss of diisopropylamine, hydrolysis, and oxidation affords **19**. The closely analogous formation of **19** by base treatment and oxidation of methyl 2-benzyloxybenzoate has already been reported.^16^

**Scheme 5:**
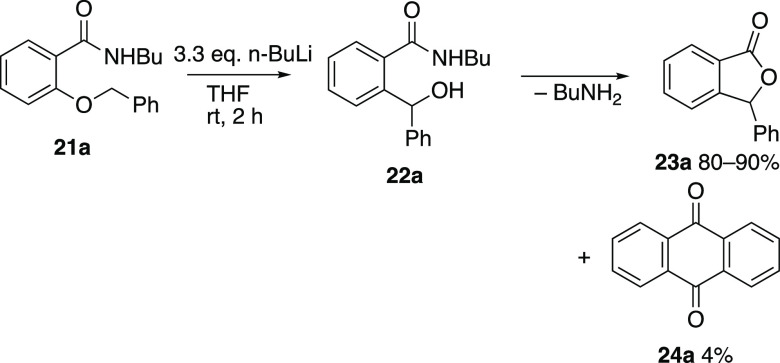
Wittig Rearrangement of 2-Benzyloxy-*N*-butylbenzamide

**Scheme 6:**
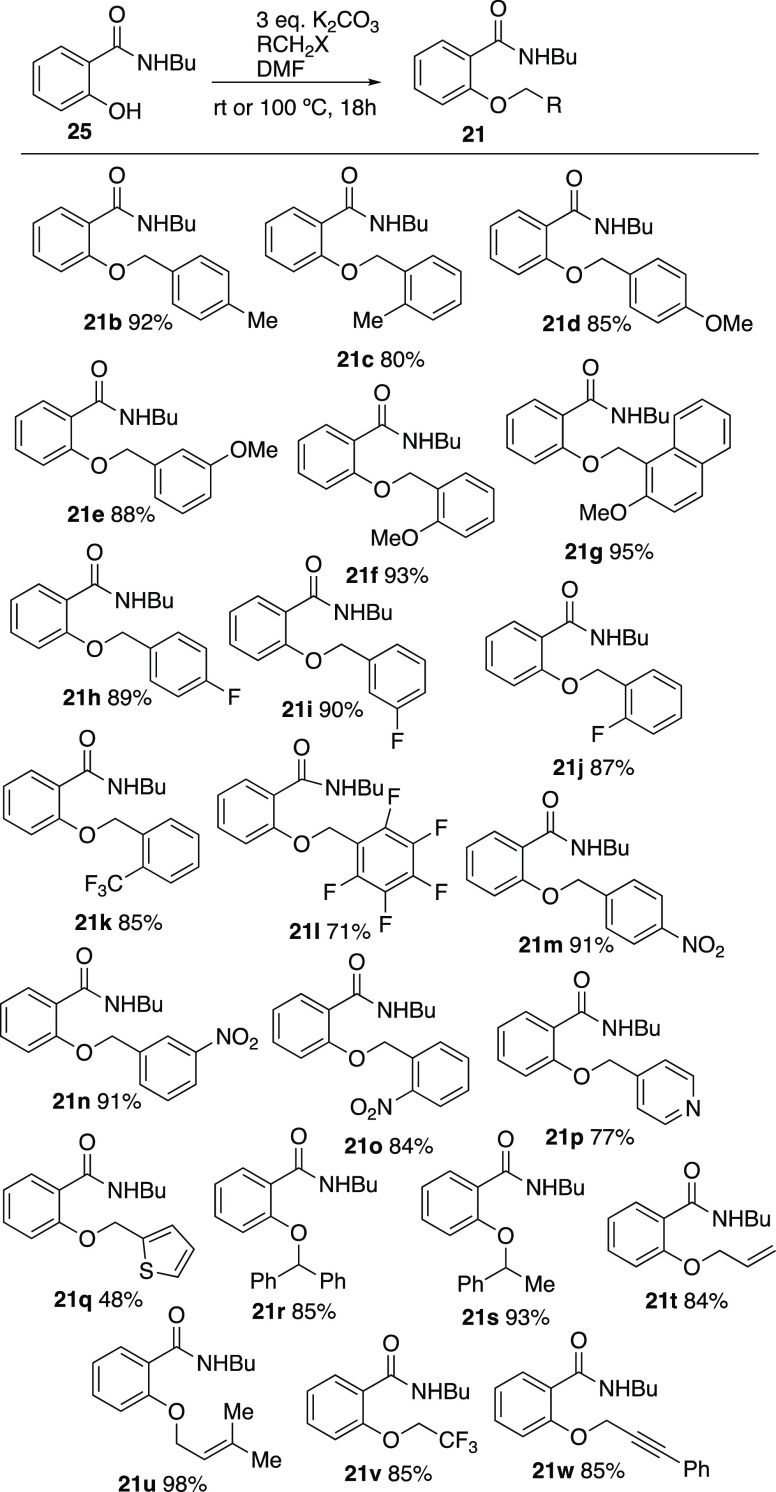
Preparation of *ortho*-substituted *N*-butylbenzamides

Success was finally achieved by moving to the secondary *N*-butylbenzamides and reaction of **21a** with 3.3 equiv *n*-butyllithium in THF at rt for 2 h followed by workup gave an essentially quantitative yield of the diarylmethanol **22a** (Scheme 5). This product was, however, found to be unstable and slowly cyclized over a period of weeks to give mainly 3-phenylphthalide **23a** (80%) accompanied by a low yield of anthraquinone **24a** resulting from an alternative mode of cyclization and subsequent oxidation. Alternatively, treating the crude product **22a** with *p*-toluenesulfonic acid in boiling toluene for 1 h,^17^ followed by aqueous workup and chromatographic purification led directly to **23a** in 90% isolated yield. The need for 3 equiv of n-BuLi is, we believe, due to the first two equivalents reacting to deprotonate the NH and then bring about an amide-directed *ortho*-metalation. Only with the third equivalent of base is the benzyl group deprotonated. Use of less n-BuLi resulted in progressively lower yields and recovery of unreacted starting material.^18^ With these optimized conditions in hand, the scope was now explored, and analogues **21b–w** were prepared in good yield by *O*-alkylation of *N*-butylsalicylamide **25** with benzylic and other halides and potassium carbonate in DMF (Scheme 6).

**Scheme 7:**
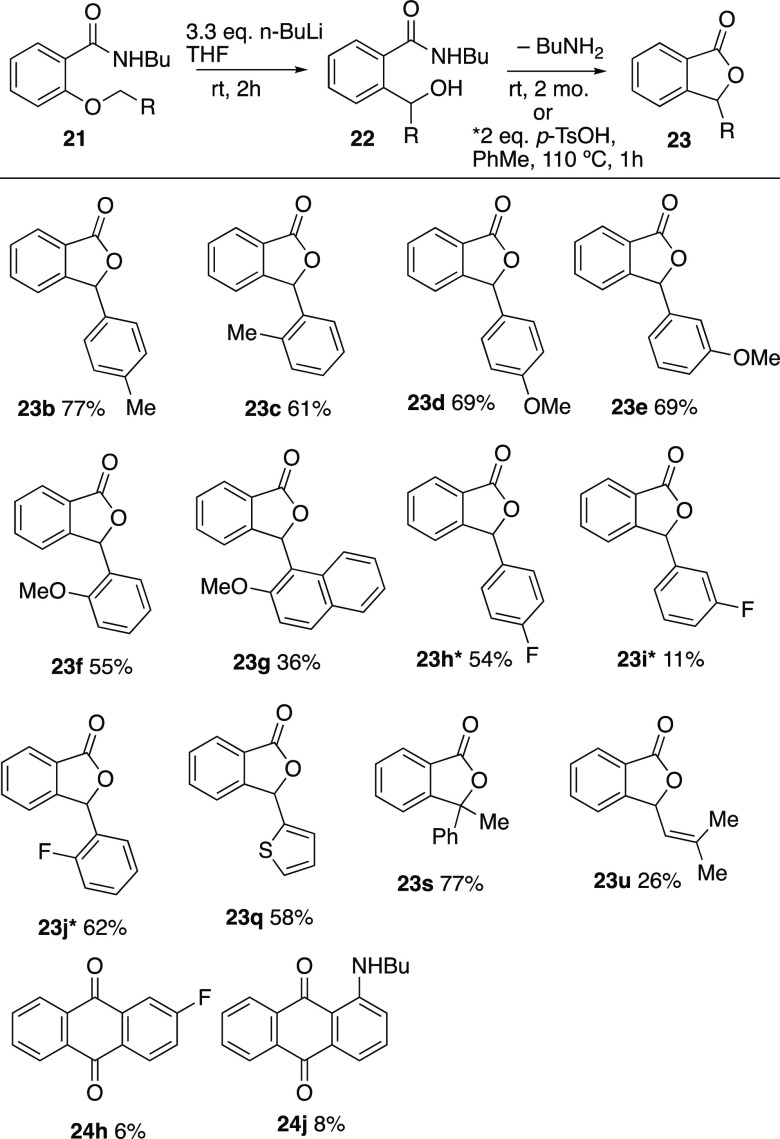
Products from Wittig Rearrangement of *ortho*-Substituted Benzamides

These were now subjected to the Wittig rearrangement conditions used for **21a** and a varied pattern of reactivity emerged (Scheme 7). The systems containing methyl, methoxy, and fluoro substituents all reacted to give the corresponding secondary alcohols **22b–j**, which were fully characterized (see Supporting Information) but cyclized upon storage or *p*-toluenesulfonic acid treatment to give the corresponding phthalides **23**. In the cases of the fluorobenzyl compounds **21h** and **21j**, the corresponding anthraquinone products **24h** and **24j** were also isolated in low yield. The thienyl compound **21q**, the α-methylbenzyl compound **21s**, and the prenyl compound **21u** also underwent rearrangement. However, the remaining compounds bearing more electron-withdrawing substituents did not, and compounds **21k–p**, **r**, **v**, and **w** either underwent decomposition or were recovered unchanged. While all the products shown in Scheme 7 were obtained by spontaneous cyclization, those marked *, in addition to **23a**, were also prepared by the *p*-toluenesulfonic acid method.

**Scheme 8:**
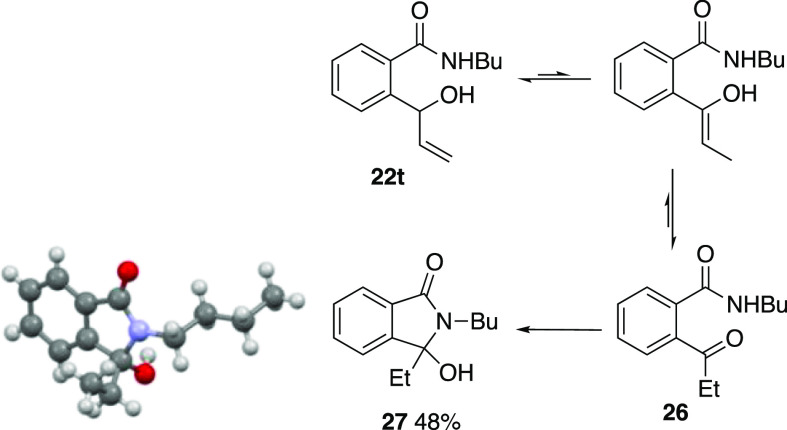
Isomerization of **22t**

The case of the allyl compound **21t** was particularly interesting. It afforded an inseparable mixture of the expected rearrangement product **22t** and an isomer, which proved to be the 3-ethyl-3-hydroxyisoindolinone **27**. Over a period of months, the mixture converted entirely into the latter, whose structure was confirmed by X-ray diffraction (Scheme 8).

We believe this to involve double-bond migration in **22t** to give the enol, which tautomerizes to ketone **26**, which then undergoes ring closure. Such isomerization of allyl carbinols to ethyl ketones occurs under a range of basic conditions.^19^

**Scheme 9:**
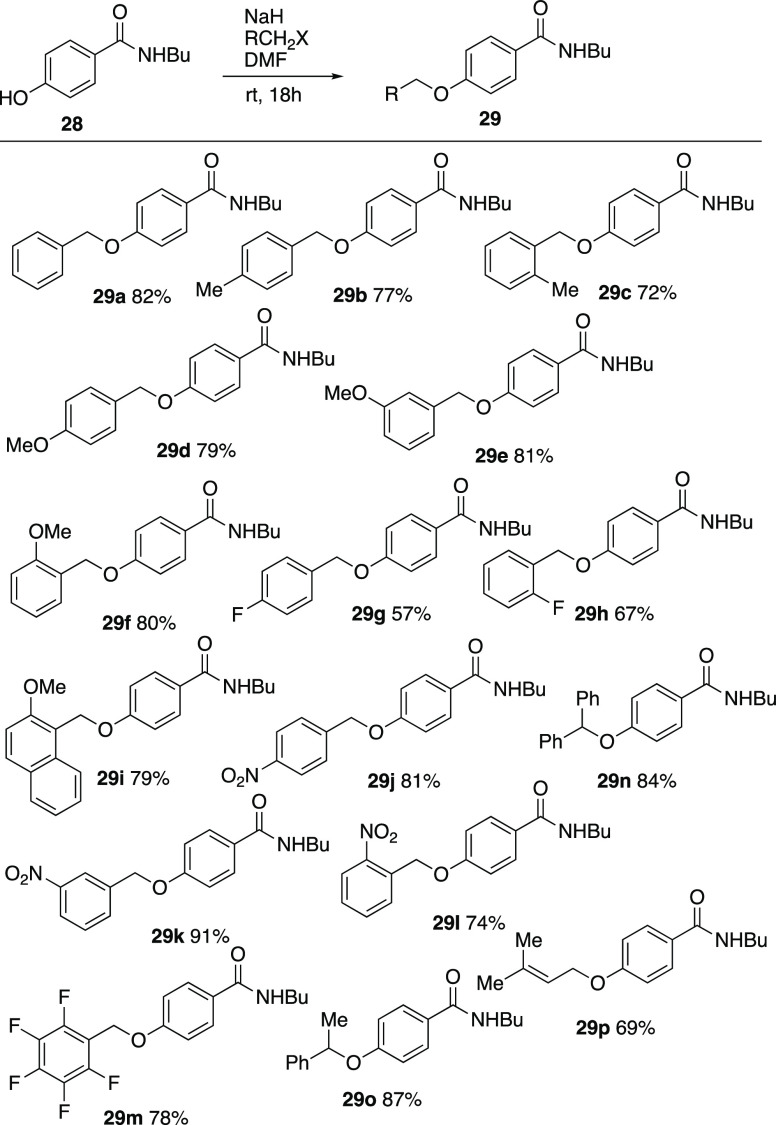
Preparation of *para*-substituted *N*-butylbenzamides

Attention was now turned to the isomeric *para*-system, and a range of substrates **29a–p** were prepared in good yield by *O*-alkylation of *N*-butyl-*p*-hydroxybenzamide **28** (Scheme 9).

**Scheme 10:**
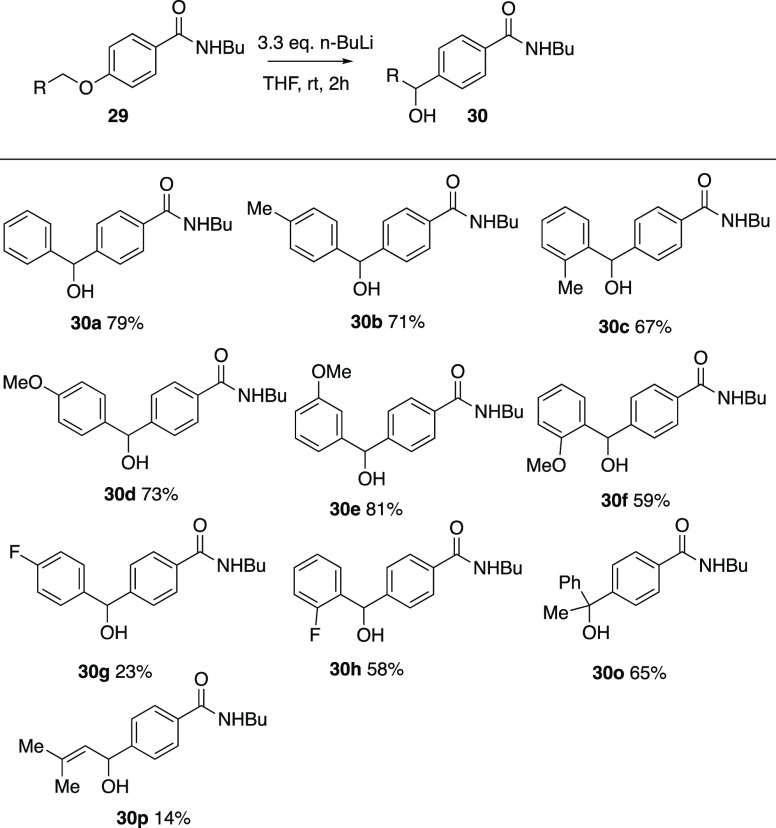
Products from Wittig Rearrangement of *para*-Substituted Benzamides

These were subjected to the normal rearrangement conditions, and a similar pattern emerged as for the *ortho* series. The unsubstituted system as well as those with methyl, methoxy, and fluoro substituents rearranged to give the diarylmethanols **30a–h** mostly in good yield (Scheme 10). The α-methylbenzyl compound **29o** and the prenyl compound **29p** also reacted to give **30o** and **30p** although with a low yield in the latter case. In the case of **30c**, the molecular structure was confirmed by X-ray diffraction and the crystal structure featured each molecule involved in two donor and two acceptor interactions, with head-to-tail hydrogen-bonded dimers linked by C=O...H–O interactions, which were then further linked by N–H...O(H)–C interactions with adjacent molecules (see Supporting Information). Again the three isomeric nitro compounds **29j–l** as well as the pentafluorophenyl compound **29m**, the diphenylmethyl compound **29n** and the methoxynaphthyl system **29i** either decomposed or were recovered unchanged.

**Scheme 11:**
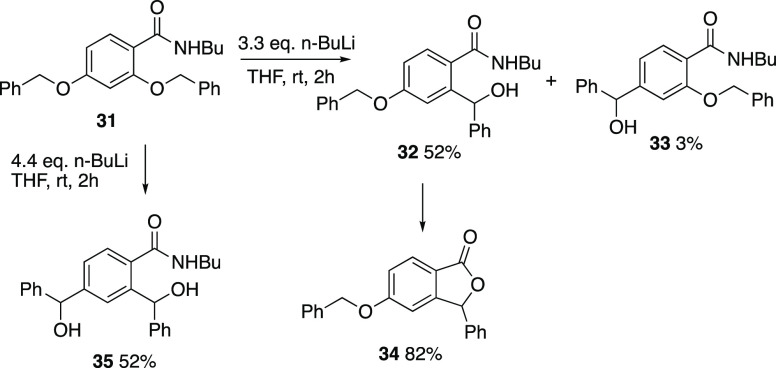
Reactivity of bis(benzyloxy) Compound **31**

Having established the viability of the rearrangement for both *ortho* and *para* isomeric systems, it was of interest to compare the relative ease of the two processes, and for this, the 2,4-bis(benzyloxy)benzamide **31** was prepared. When this was treated with 3.3 equiv n-BuLi, the product was mainly **32** resulting from rearrangement of the *ortho* group with just a trace of the isomer **33** from the reaction of the *para* group (Scheme 11). This appears to be the first example of an amide directing lithiation onto an *ortho*-alkoxy group. Storage of compound **32** over a period of months resulted in spontaneous cyclization to give phthalide **34** in good yield. On the other hand, treatment of **31** with 4.4 equiv of n-BuLi resulted in rearrangement of both groups to give the diol **35** as a 1:1 mixture of diastereomers.

**Scheme 12:**
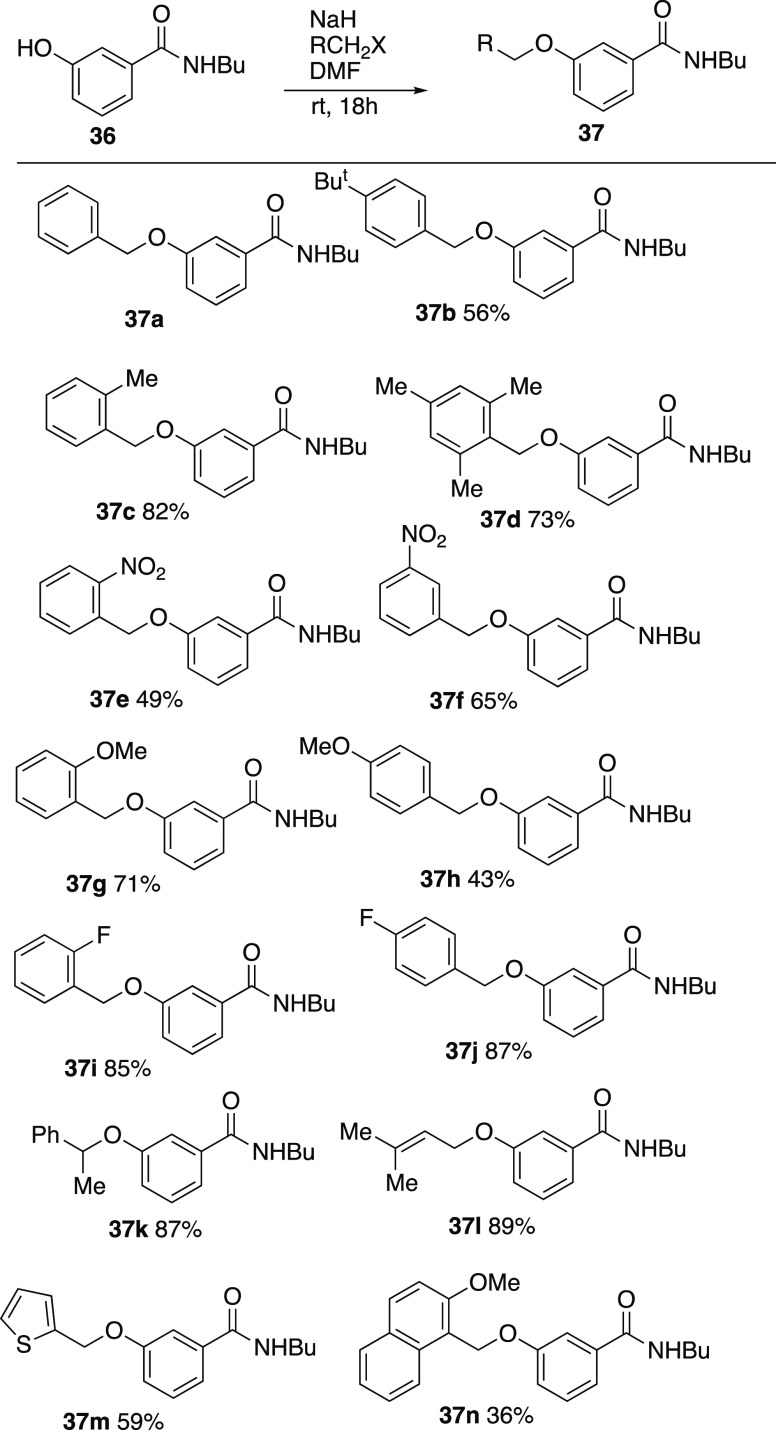
Preparation of *meta*-substituted *N*-butylbenzamides

**Scheme 13:**
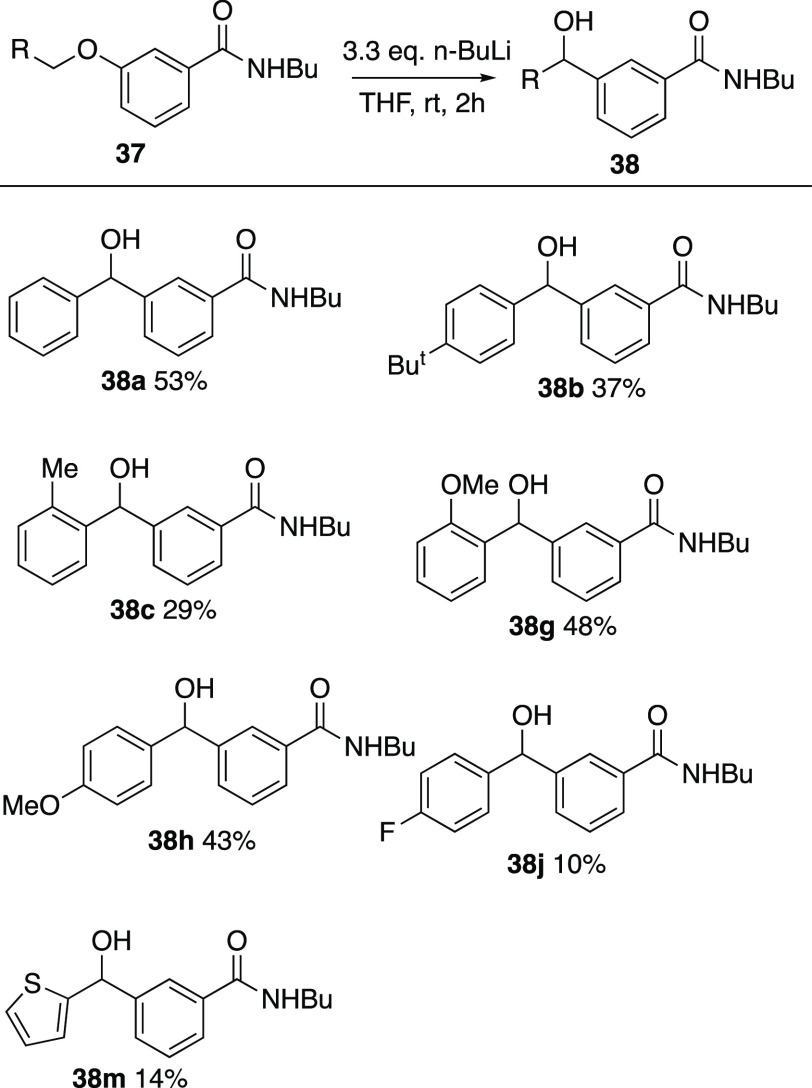
Products from Wittig Rearrangement of *meta*-Substituted Benzamides

Based on the earlier finding that the *meta*-benzyloxy oxazoline **11** did not react, in contrast to *ortho* and *para*-isomers **5** and **9**, and assuming that the activating effect of the amide group in the Wittig rearrangement would be via a spiro anionic intermediate (see below, Scheme 17), we expected that *meta*-benzyloxy *N*-butylbenzamides **37** would not react. However, to our surprise, the unsubstituted compound **37a** did rearrange under the normal conditions to afford **38a**, albeit in a somewhat lower yield than for *ortho* or *para*-isomers. The structure of **38a** was confirmed by X-ray diffraction and again the crystal structure featured each molecule involved in two donor and two acceptor interactions, but in contrast to **30c**, this involved head-to-tail hydrogen-bonded dimers linked by N–H...O(H)–C interactions, which were then further linked by C=O...H–O interactions with adjacent molecules (see Supporting Information). Based on this result, a range of substituted examples **37b–n** were prepared in good-to-moderate yield by *O*-alkylation of **36** (Scheme 12). When these were subjected to the standard rearrangement conditions, a somewhat more restricted pattern of reactivity emerged with successful rearrangement only being observed for alkyl, methoxy, and fluoro substituents as well as the thienyl compound **37m** and, in all cases, the isolated yields were lower than for the isomeric systems (Scheme 13).

**Scheme 14:**
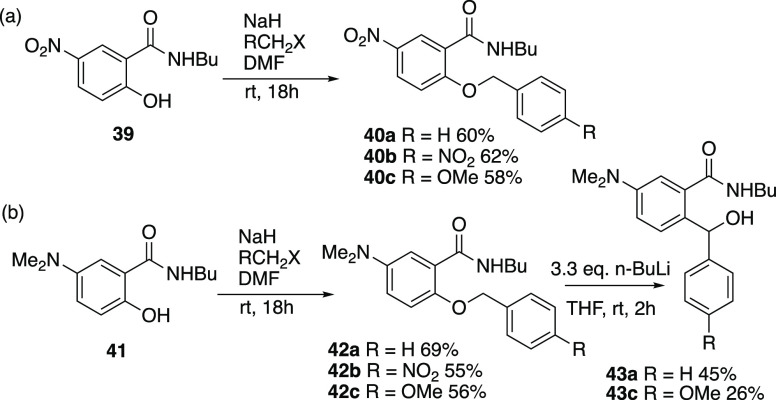
Nitro and Dimethylamino Substituted Systems

**Scheme 15:**
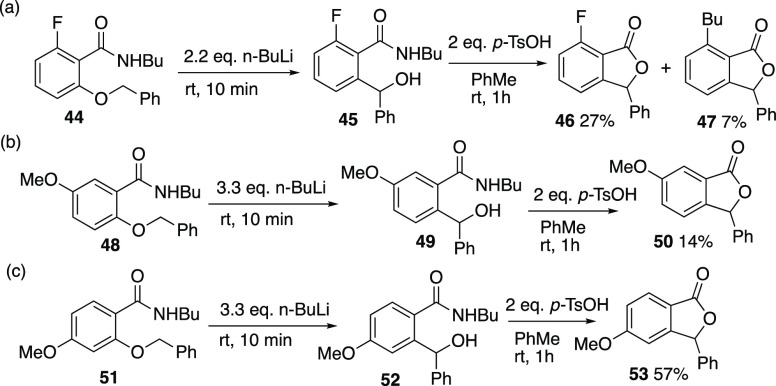
Rearrangement with 4-, 5-, or 6-Substituents

**Scheme 16:**
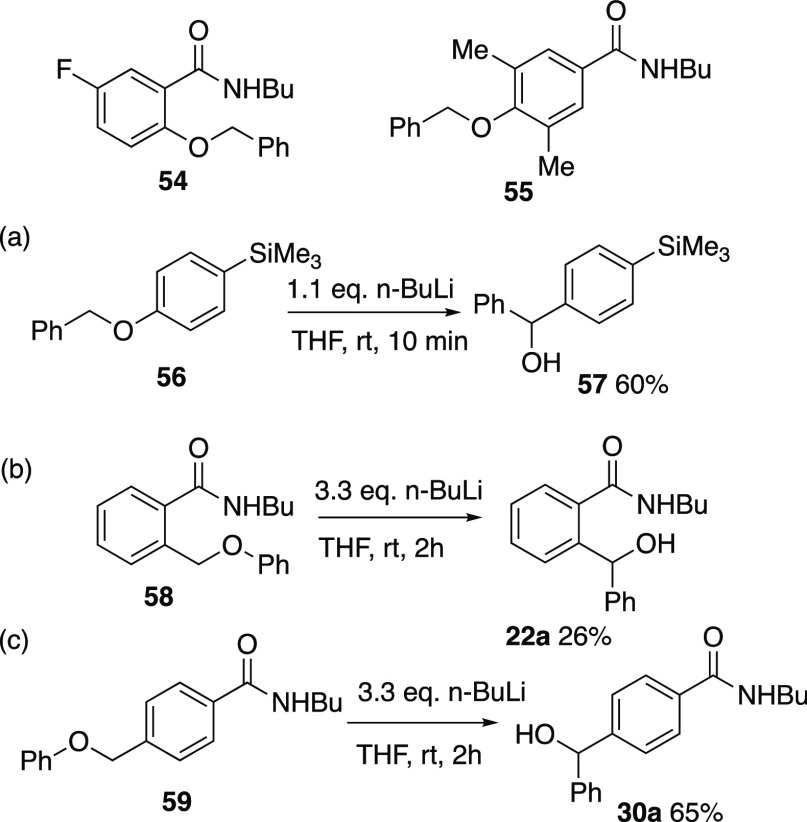
Further Mechanistic Probes Examined

So far substituent effects have only been examined in the benzyl as opposed to the aryl ring. To further examine the scope, we returned to the *ortho* system and investigated the effect of substituents on the other aryl ring. Starting from the 5-nitrosalicylamide **39**, a range of three *O*-benzyl derivatives **40a–c** were prepared, while the corresponding 5-dimethylamino compound **41** led to derivatives **42a–c**. However, when these six compounds were subjected to the usual rearrangement conditions, only in the cases of **42a** and **42c** were the corresponding rearrangement products **43a** and **43c** obtained (Scheme 14). It is clear that the presence of a nitro group on either ring is sufficient to prevent the reaction. Since this is most likely due to incompatibility of the nitro function with the reaction conditions, additional evidence was sought from isomeric compounds with methoxy and fluoro substituents, both of which were compatible with the reaction conditions. Thus the 6-fluoro compound **44** in which *ortho*-metalation is impossible was treated with 2.2 equiv n-BuLi to give the expected rearrangement product **45**, isolated after *p*-toluenesulfonic acid-mediated cyclization as the 7-fluoro-3-phenylphthalide (27%), together with the *n*-butyl compound **47** resulting from nucleophilic aromatic substitution in low yield (Scheme 15). Methoxy substituents in either the 4- or 5-position were also compatible with the rearrangement and **48** reacted to form **49**, isolated as the phthalide **50** while **51** reacted via **52** to give the phthalide product **53** in moderate yield. In contrast to this, the 5-fluoro compound **54** (Scheme 16) underwent decomposition under the normal rearrangement conditions, perhaps due to intervention of an aryne process, while the 3,5-dimethyl-4-benzyloxy compound **55** in which the supposed spiro anion intermediate is sterically disfavored, was recovered largely unchanged.

To further probe the mechanism, the 4-trimethylsilyl compound **56** was prepared and was found to undergo rearrangement readily with n-BuLi to afford the alcohol **57**. Finally, compounds **58** and **59** in which the benzyloxy group of **21a** and **29a** is replaced by the isomeric phenoxymethyl group were prepared, and these were also found to readily undergo the rearrangement under the normal conditions, giving, respectively, **22a** and **30a**.

**Scheme 17:**
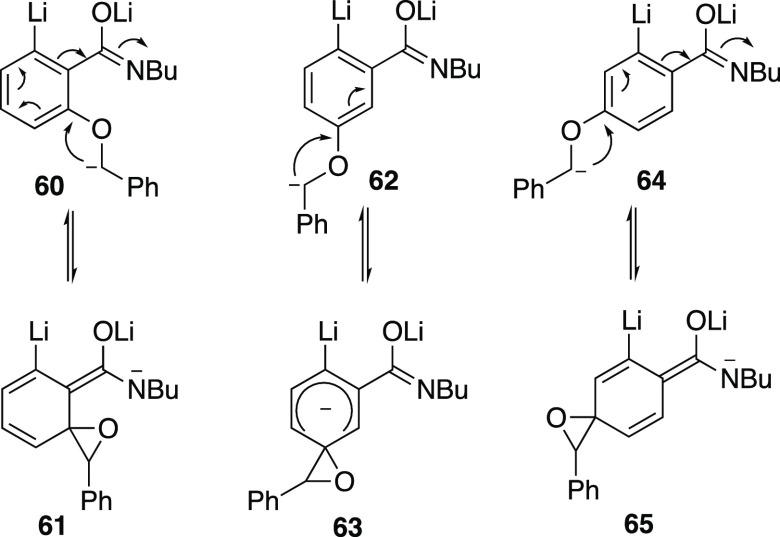
Opportunities for Charge Stabilization in the Three Isomeric Systems

Although there have been a good number of mechanistic studies on the [1,2]-Wittig rearrangement, some early suggestions involving the intermediacy of arynes and carbonyl compounds were later disproven.^20^ The most recent and detailed mechanistic study on aryl benzyl ethers employing both experimental and theoretical methods,^21^ quite clearly points to two major mechanistic possibilities: an anionic mechanism via a spiro-epoxide intermediate that is the normal route for neutral and electron-poor aryl rings, and a radical dissociation/recombination route that is more likely to be important for electron-rich aryl systems. In our system, we envisage initial amide NH deprotonation and *ortho*-metalation in each case before the third equivalent of n-BuLi deprotonates the benzyl group. The resulting intermediates **60**, **62**, and **64** can each cyclize to the spiro epoxides implicated in the Wittig rearrangement (Scheme 17) but, while **61** and **65** are be stabilized by the negative charge being on nitrogen, this is not possible for **63** derived from the *meta* compound thus explaining the lower yields obtained in that case.

The recent mechanistic study^21^ was focused on substituent effects on the aryl ring rather than the benzyl ring, and substituent effects on the benzyl ring do not seem to have been examined in detail until now. Overall our results on the three isomeric benzyloxybenzamide systems show that a single *N*-butylbenzamide group on the aryl ring is sufficient to facilitate the rearrangement of *ortho* and *para* benzyloxy systems, presumably via the anionic spiro-epoxide intermediates. The *meta* system is slightly less prone to rearrangement presumably reflecting the reduced capacity for delocalization of the negative charge to a *meta* disposed electron-withdrawing amide. In all three series, the reaction proceeds with a wide range of both electron-donating and electron-withdrawing benzyl substituents. Only nitro compounds were uniformly unsuccessful due to the incompatibility of that group with butyllithium. The fact that the silyl compound **56** rearranges much more rapidly than benzyl phenyl ether is consistent with the anionic mechanism in that case where the stabilizing effect of silicon upon the α-anion is key. For the isomeric phenoxymethyl compounds **58** and **59** where the spiro-epoxide intermediate cannot be stabilized, the amide group nevertheless promotes the reaction perhaps by favoring the benzylic deprotonation, but the rearrangement must necessarily proceed by the radical route in these cases.

The fact that **48** gives a substantially lower yield than the isomer **51** implies that the rearrangement is discouraged by the presence of a second inductively electron-withdrawing (but mesomerically electron-donating) group in the *para* position to the rearranging group. The occurrence of the rearrangement for **42a** and **42c**, which also have an inductively electron-withdrawing group *para* to the reaction site, can perhaps be taken to indicate that the electron-donating mesomeric effect outweighs the inductive effect in these cases.

**Scheme 18:**
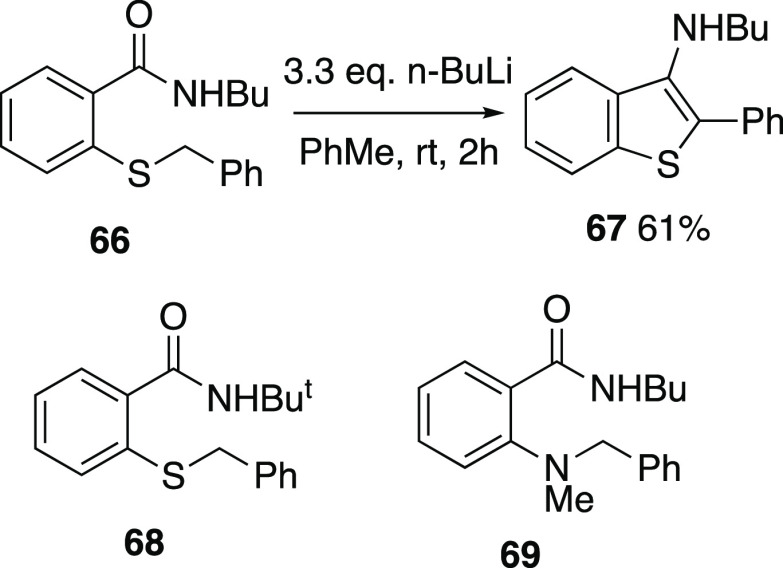
Attempted Extension to thia- and aza-Analogues

Finally, we examined briefly whether the process could be extended from the ethers to the corresponding sulfides or amines since both thia- and aza-[1,2]-Wittig rearrangements are known.^22,23^ The 2-benzylthio-*N*-butylbenzamide **66** was readily prepared, but upon treatment with butyllithium, it underwent dehydrative cyclization to afford the 3-aminobenzothiophene **67** in moderate yield, in a reaction analogous to the formation of **20** from **18** (Scheme 18). In an attempt to suppress this process, the more bulky tert-butylbenzamide **68** was prepared, but it was recovered unreacted from BuLi treatment as was the 2-(benzylmethylamino) analogue **69**.

## Experimental Section

^1^H and ^13^C NMR spectra were recorded in CDCl_3_ unless otherwise stated with internal TMS as reference. IR spectra were recorded using the ATR technique. HRMS measurements were made either using ESI with TOF analyzer or NSI with an ion trap analyzer.

The following procedures are illustrative; full experimental procedures and characterization data are given in the Supporting Information.

### 2-(Benzyloxy)-*N*-butylbenzamide **21a**

A solution of 2-(benzyloxy)benzoyl chloride^13^ (10.85 g, 44.0 mmol) in toluene (60 mL) was added dropwise to a stirred 0 °C solution of *n*-butylamine (12.1 mL, 8.95 g, 0.122 mol) in toluene (60 mL). Once the addition was complete, the reaction mixture was allowed to warm to rt for 1 h before being poured into water and washed with 2 M NaOH and brine. The organic layer was dried and evaporated to afford, after recrystallization (EtOAc/hexane), **21a** (9.34 g, 75%) as colorless crystals, mp 52–54 °C; IR 3380, 1648, 1599, 1558, 1292, 1238, 1164, 1101, 1005, 865, 752, 700 cm^–1^; ^1^H NMR (500 MHz) δ 8.25 (dd, *J* = 7.8, 1.8 Hz,1H, ArH), 7.88 (br s, 1H, NH), 7.47–7.38 (m, 6H, ArH and Ph), 7.12–7.08 (m, 1H, ArH), 7.06 (d, *J* = 8.5 Hz, 1H, ArH), 5.15 (s, 2H, OCH_2_), 3.34 (td, *J* = 7.0, 5.5 Hz, 2H, NCH_2_), 1.35–1.29 (m, 2H, NCH_2_C*H*_2_), 1.19–1.11 (m, 2H, C*H*_2_CH_3_), 0.80 (t, *J* = 7.3 Hz, 3 H, CH_3_); ^13^C NMR (75 MHz) δ 164.9(C), 156.8(C), 135.5(C), 132.5 (CH), 132.4 (CH), 128.9 (2CH), 128.8 (CH), 128.2 (2CH), 122.0(C), 121.6 (CH), 112.4 (CH), 71.4 (OCH_2_), 39.4 (NCH_2_), 31.2 (CH_2_), 20.0 (CH_2_), and 13.7 (CH_3_); HRMS (ESI^+^) *m*/*z* [M + Na^+^] calcd for C_18_H_21_NaNO_2_ 306.1465, found 306.1455.

### *N*-Butyl-2-(hydroxy(phenyl)methyl)benzamide **22a**, Anthraquinone **24a** and 3-Phenylphthalide **23a**

Under a nitrogen atmosphere, *n*-butyllithium (2.6 mL, 6.50 mmol) was added dropwise to a stirred solution of 2-(benzyloxy)-*N*-butylbenzamide **21a** (0.5678 g, 2.00 mmol) in dry THF (20 mL). After stirring at rt for 2 h, the reaction mixture was quenched by addition of sat. aq. NH_4_Cl and extracted with Et_2_O (×3). The combined organic layers were washed with 2 M NaOH and water before being dried and evaporated to give **22a** as a pale yellow oil: IR 3296, 3064, 2931, 1635, 1540, 1450, 1303, 1228, 1104, 1024, 757, 699 cm^–1^; ^1^H NMR (500 MHz) δ 7.38 (t, *J* = 7.3 Hz, 2H, ArH), 7.29–7.17 (m, 7H, ArH and Ph), 6.31 (t, *J* = 5.3 Hz, 1H, NH), 5.79 (s, 1H, C*H*OH), 3.21–3.14 (m, 1H, NCH_2_), 3.12–3.05 (m, 1H, NCH_**2**_), 1.29–1.23 (m, 2H, NCH_2_C*H*_2_), 1.22–1.15 (m, 2H, C*H*_2_CH_3_), 0.85 (t, *J* = 7.0 Hz, 3H, CH_3_); ^13^C NMR (125 MHz) δ 170.8(C), 143.1(C), 142.7(C), 135.8(C), 130.6 (CH), 129.9 (CH), 127.74 (CH), 127.71 (2CH), 127.69 (CH), 126.7 (CH), 126.2 (2CH), 74.9 (CHOH), 39.7 (NCH_2_), 31.1 (CH_2_), 19.9 (CH_2_), 13.6 (CH_3_); HRMS (ESI^+^) *m*/*z* [M + Na^+^] calcd for C_18_H_21_NaNO_2_ 306.1465, found 306.1456.

On standing at rt in EtOAc solution for 2–3 months, an intramolecular cyclization occurred to give, after purification by column chromatography (SiO_2_, Et_2_O/hexane 2:3), at *R*_f_ 0.80, **24a** (17.5 mg, 4%) as yellow needles, mp 275–279 °C (lit.^24^ 275 °C); ^1^H NMR (500 MHz) δ 8.34–8.30 (m, 4 H, ArH), 7.83–7.79 (m, 4 H, ArH). The ^1^H NMR spectral data were in accordance with those previously reported.^25^

This was followed by a second fraction to give, at *R*_f_ 0.55, **23a** (0.3350 g, 80%) as tan-colored crystals, mp 113–116 °C (lit.^26^ 115.5 °C); ^1^H NMR (500 MHz) δ 7.96 (d, *J* = 7.5 Hz, 1H, ArH), 7.65 (td, *J* = 7.5, 1.0 Hz, 1H, ArH), 7.55 (t, *J* = 7.5 Hz, 1H, ArH), 7.39–7.36 (m, 3H, ArH), 7.33 (dd, *J* = 7.8, 0.8 Hz, 1H, ArH), 7.29–7.26 (m, 2H, ArH), 6.41 (s, 1H, CHPh). The ^1^H NMR spectral data were in accordance with those previously reported.^27^

Alternatively, the following literature procedure^28^ may be employed: A mixture of *N*-butyl-2-(hydroxy(phenyl)methyl)benzamide **22a** (prepared as above from 1.14 g **21a**, assuming 4.02 mmol) and *p*-toluenesulfonic acid monohydrate (1.55 g, 8.15 mmol) in toluene (80 mL) was heated at reflux for 1 h. After cooling to rt, the reaction mixture was washed with water (50 mL), 2 M NaOH (50 mL), and brine (50 mL) before being dried and evaporated. The crude residue was purified by column chromatography (SiO_2_, gradient elution, Et_2_O/hexane 1:4 to Et_2_O) to give **23a** (0.76 g, 90%) as tan-colored crystals.

### *N*-Butyl-4-hydroxybenzamide **28**

A mixture of methyl 4-hydroxybenzoate (30.43 g, 0.20 mol) and *n*-butylamine (100 mL, 74.00 g, 1.01 mol) was heated at reflux for 4 d before being concentrated *in vacuo*. The residue was acidified to pH 1 by the addition of 2 M HCl and extracted with Et_2_O (3 × 100 mL). The combined organic layers were washed with water (100 mL) before being dried and evaporated. The crude residue was recrystallized (EtOAc/PhMe) to give **28** (28.01 g, 72%) as colorless crystals, mp 118–120 °C (lit.^29^ 118.5–119.5 °C); ^1^H NMR (500 MHz) δ 7.95 (br s, 1H, OH), 7.62 (d, *J* = 8.8 Hz, 2H, 2,6-H), 6.86 (d, *J* = 8.8 Hz, 2H, 3,5-H), 6.17 (t, *J* = 5.5 Hz, 1H, NH), 3.44 (td, *J* = 7.0, 5.5 Hz, 2H, NCH_2_), 1.62–1.56 (m, 2H, NCH_2_C*H*_2_), 1.44–1.36 (m, 2H, C*H*_2_CH_3_), 0.94 (t, *J* = 7.3 Hz, 3H, CH_3_). The ^1^H NMR spectral data were in accordance with those previously reported.^30^

### 4-(Benzyloxy)-*N*-butylbenzamide **29a**

*N*-Butyl-4-hydroxybenzamide **28** (3.87 g, 20.0 mmol) was added to a stirred suspension of sodium hydride (60% in mineral oil, prewashed with hexane, 0.82 g, 20.5 mmol) in DMF (20 mL), and the mixture was stirred at rt for 15 min before benzyl bromide (2.4 mL, 3.45 g, 20.2 mmol) was added. After stirring for 18 h at rt, the reaction mixture was poured into water and extracted with CH_2_Cl_2_ followed by Et_2_O (×3). The combined organic layers were washed with brine (×5) and 2 M NaOH before being dried and evaporated. Recrystallization of the residue (EtOAc/hexane) gave **29a** (4.68 g, 82%) as colorless crystals, mp 126–128 °C; (lit.^31^ 119.1–119.7 °C); ^1^H NMR (500 MHz) δ 7.72 (d, *J* = 8.8 Hz, 2H, 2,6-H), 7.44–7.38 (m, 4H, Ph), 7.35–7.32 (m, 1H, Ph), 6.99 (d, *J* = 8.8 Hz, 2H, 3,5-H), 6.03 (t, *J* = 5.5 Hz, 1H, NH), 5.11 (s, 2H, OCH_2_), 3.44 (td, *J* = 7.3, 5.5 Hz, 2H, NCH_2_), 1.62–1.56 (m, 2H, NCH_2_C*H*_2_), 1.45–1.37 (m, 2H, C*H*_2_CH_3_), and 0.95 (t, *J* = 7.3 Hz, 3H, CH_3_). The ^1^H NMR spectral data were in accordance with those previously reported.^31^

### *N*-Butyl-4-(hydroxy(phenyl)methyl)benzamide **30a**

Under a nitrogen atmosphere, *n*-butyllithium (2.5 M in hexane, 6.6 mL, 16.5 mmol) was added dropwise to a stirred solution of 4-(benzyloxy)-*N*-butylbenzamide **29a** (1.41 g, 4.98 mmol) in dry THF (50 mL). After stirring at rt for 2 h, the reaction mixture was quenched by addition of sat. aq. NH_4_Cl and extracted with Et_2_O (×3). The combined organic layers were washed with 2 M NaOH and water before being dried and evaporated. Purification of the residue by column chromatography (SiO_2_, gradient elution, Et_2_O/hexane 7:3 to Et_2_O) and subsequent recrystallization (EtOAc/hexane) gave **30a** (1.11 g, 79%) as colorless crystals, mp 113–114 °C; IR 3432, 3337, 2953, 2926, 1616, 1542, 1448, 1303, 1228, 1045, 736, 694 cm^–1^; ^1^H NMR (400 MHz) δ 7.67 (d, *J* = 8.2 Hz, 2H, ArH), 7.41 (d, *J* = 8.2 Hz, 2H, ArH), 7.36–7.30 (m, 4H, Ph), 7.28–7.24 (m, 1H, Ph), 6.14 (t, *J* = 5.6 Hz, 1H, NH), 5.85 (s, 1H, C*H*OH), 3.41 (td, *J* = 7.2, 5.6 Hz, 2H, NCH_2_), 2.76 (d, *J* = 3.2 Hz, 1H, OH), 1.61–1.53 (m, 2H, NCH_2_C*H*_2_), 1.43–1.34 (m, 2H, C*H*_2_CH_3_), 0.94 (t, *J* = 7.4 Hz, 3H, CH_3_); ^13^C NMR (125 MHz) δ 167.3(C), 147.1(C), 143.4(C), 133.8(C), 128.6 (2CH), 127.8 (CH), 126.9 (2CH), 126.6 (2CH), 126.5 (2CH), 75.7 (CHOH), 39.8 (NCH_2_), 31.7 (CH_2_), 20.1 (CH_2_), 13.8 (CH_3_); HRMS (NSI^+^) *m*/*z* [M + H^+^] calcd for C_18_H_22_NO_2_ 284.1645, found 284.1644.

### 3-(Benzyloxy)-N-butylbenzamide **37a**

To a stirred solution of 3-(benzyloxy)benzoyl chloride^32^ (6.82 g, 27.6 mmol) in CH_2_Cl_2_ (100 mL) at 0 °C, Et_3_N (3.85 mL, 27.6 mmol) was added dropwise. The solution was stirred for 5 min, and then *n*-butylamine (2.73 mL, 27.6 mmol) was added dropwise and the mixture stirred at rt for 18 h. The reaction mixture was poured into H_2_O, extracted (×3) with CH_2_Cl_2_, and the combined organic fractions were dried over MgSO_4_ and concentrated to afford, after recrystallization (EtOH) **37a** (5.80 g, 74%) as off-white crystals, mp 83–86 °C; IR 3298, 3229, 2961, 1626, 1603, 1580,1553, 1016, 698 cm^–1^; ^1^H NMR (400 MHz) δ 7.47–7.38 (m, 5H, ArH), 7.38–7.32 (m, 2H, ArH), 7.32–7.28 (m, 1H, ArH), 7.09 (ddd, *J* = 7.8, 2.6, 1.5 Hz, 1H, ArH), 6.07 (br s, 1H, NH), 5.11 (s, 2H, OCH_2_), 3.45 (td, *J* = 7.1, 5.7 Hz, 2H, NHC*H*_2_), 1.65–1.53 (m, 2H, NHCH_2_C*H*_2_), 1.51–1.30 (m, 2H, C*H*_2_CH_3_), 0.96 (t, *J* = 7.3 Hz, 3H, CH_2_C*H*_3_); ^13^C NMR (100 MHz) δ 167.2(C), 158.9(C), 136.5(C), 136.4(C), 129.6 (CH), 128.6 (CH), 128.1 (CH), 127.5 (CH), 118.8 (CH), 118.1 (CH), 113.3 (CH), 70.1 (OCH_2_), 39.8 (NHCH_2_), 31.7 (CH_2_), 20.1 (CH_2_), 13.8 (CH_3_); HRMS (ESI^+^) *m*/*z* [M + H^+^] calcd for C_18_H_22_NO_2_ 284.1651, found 284.1636.

### *N*-Butyl-3-(hydroxy(phenyl)methyl)benzamide **38a**

Under a nitrogen atmosphere, *n*-butyllithium (2.5 M in hexane, 2.64 mL, 6.60 mmol) was added dropwise to a stirred solution of 3-(benzyloxy)-*N*-butylbenzamide **37a** (567 mg, 2.0 mmol) in dry THF (20 mL). After stirring at rt for 2 h, the reaction mixture was quenched by addition of sat. aq. NH_4_Cl and extracted with Et_2_O (×3). The combined organic layers were dried and evaporated. Purification of the residue by column chromatography (gradient elution, Et_2_O/hexane 1:1 to Et_2_O/hexane 7:3) gave **38a** (298 mg, 53%) as colorless crystals, mp 97–100 °C; IR 3298, 3229, 2961, 2932, 2857, 1626, 1553, 1418, 1327, 1016, 746, 698 cm^–1^; ^1^H NMR (400 MHz) δ 7.75 (t, *J* = 1.8 Hz, 1H, ArH), 7.57 (dt, *J* = 7.7, 1.5 Hz, 1H, ArH), 7.42–7.35 (m, 1H, ArH), 7.31–7.24 (m, 5H, ArH), 7.24–7.21 (m, 1H, ArH) 6.43 (t, *J* = 5.7 Hz, 1H, NH), 5.74 (s, 1H, C*H*OH), 3.71 (s, 1H, CHO*H*), 3.31 (td, *J* = 7.2, 5.7 Hz, 2H, NHC*H*_2_), 1.54–1.45 (m, 2H, NHCH_2_C*H*_2_), 1.39–1.27 (m, 2H, C*H*_2_CH_3_), 0.90 (t, *J* = 7.3 Hz, 3H, CH_2_C*H*_3_); ^13^C NMR (100 MHz) δ 167.6(C), 144.5(C), 143.5(C), 134.7(C), 129.5 (CH), 128.5 (CH), 128.4 (2CH), 127.5 (CH), 126.5 (2CH), 125.9 (CH), 124.7 (CH), 75.6 (CHOH), 39.8 (NHCH_2_), 31.5 (CH_2_), 20.1 (CH_2_), 13.7 (CH_3_); HRMS (ESI^+^) *m*/*z* [M + H^+^] calcd for C_18_H_22_NO_2_ 284.1651, found 284.1637.

## Supporting Information Available

Experimental procedures, characterization data, NMR spectra of all newly synthesized compounds, and X-ray crystallographic details (PDF)

## Accession Codes

CCDC 2105490–2105493 contain the supplementary crystallographic data for this paper. These data can be obtained free of charge via www.ccdc.cam.ac.uk/data_request/cif, or by emailing data_request@ccdc.cam.ac.uk, or by contacting The Cambridge Crystallographic Data Centre, 12 Union Road, Cambridge CB2 1EZ, UK; fax: +44 1223 336033.
