# Supplementary material for: Access to Diarylmethanols by Wittig Rearrangement of ortho-, meta-, and para-Benzyloxy-N-Butylbenzamides
Source: J Org Chem. 2022 Mar 14;87(7):4692–701. doi: 10.1021/acs.joc.1c03160 (PMC9007461; doi:10.1021/acs.joc.1c03160)
Supplement: Supplementary file 1 — jo1c03160_si_001.pdf [file jo1c03160_si_001.pdf]

Access to Diarylmethanols by Wittig Rearrangement of *ortho*-, *meta*- and *para*-Benzyloxy-*N*-butylbenzamides

R. Alan Aitken,* Andrew D. Harper, Ryan A. Inwood and Alexandra M. Z. Slawin

Supporting Information

Instrumentation and General Techniques **S1**

Detailed Experimental Procedures and Product Characterization **S2–S48**

References **S48–S49**

NMR Spectra of New Compounds **S50–S187**

X-Ray Structure Determination Data **S188–S191**

Instrumental and General Techniques

All NMR spectra were recorded at ambient temperature on Bruker AV 300, Bruker AV 400, Bruker AVII 400, Bruker AVIII 500 or Bruker AVIII-HD 500 instruments from solutions in deuteriochloroform unless otherwise indicated. Chemical shifts (δ) are reported in ppm from high to low frequency relative to internal tetramethylsilane (δ_H = 0.00), $CDCl_3$ (δ_C = 77.0) or external $CFCl_3$ (δ_F = 0.00). Coupling constants (*J*) are reported in Hz. Standard abbreviations indicating multiplicity were used as follows: br = broad, s = singlet, d = doublet, t = triplet, q = quartet and m = multiplet. All ^{13}C resonances are singlets unless otherwise indicated.
IR spectra were recorded as Nujol mulls or thin films on a Perkin Elmer 1420 instrument or using the ATR technique on a Shimadzu IRAffinity 1S instrument.
Mass spectrometry was carried out by the services at the University of St Andrews or the EPSRC UK National Mass Spectrometry Facility at Swansea University. The ionisation method used is stated in each case.
All melting points were taken using a Gallenkamp 50W melting point apparatus or a Reichert hot-stage microscope and are reported uncorrected.
Thin Layer Chromatography (TLC) was carried out using 0.20 mm layer of silica (Merck, Kieselgel $60F_{254}$) or alumina (Merck, aluminium oxide $60F_{254}$) on aluminium sheets. The components were observed under UV light.
Preparative Thin Layer Chromatography was carried out using 1.0 mm layers of silica (Merck, Kieselgel 60–80 mesh) or alumina (Merck, aluminium oxide 60G), containing 0.5% Woelm fluorescent green indicator, on glass plates. After locating the components with ultraviolet (UV) light, the bands were scraped off and the products removed from the support by soaking in CH_2Cl_2/MeOH (10:1) for several hours.
Column Chromatography was carried out using silica gel of 40–63 μm particle size or aluminium oxide (63–200 μm).
Kugelrohr Distillation was carried out using a Büchi model GKR-50 apparatus.
Organic solutions were dried by standing over anhydrous magnesium sulfate unless otherwise indicated. After filtration, solutions were evaporated under reduced pressure on a Büchi rotary evaporator using a water pump vacuum.
Commercially available solvents were used without further purification unless otherwise stated. Where pure acetone was required, the commercially available analytical reagent grade solvent was used. Dry Et_2O, THF and toluene were prepared by the addition of sodium wire.

Structures of compounds not appearing in the paper

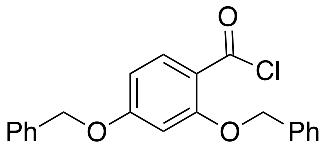

A

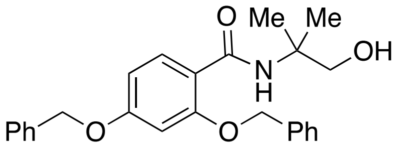

B

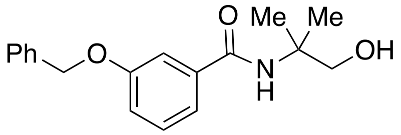

C

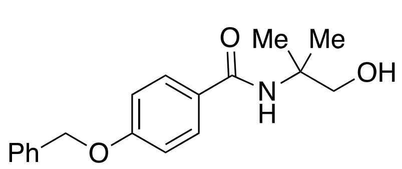

D

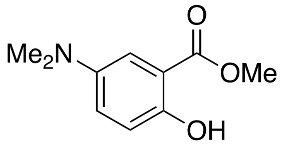

E

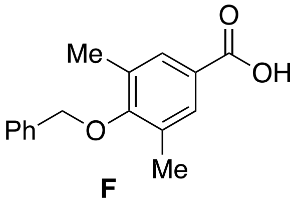

F

[**A** and **B**, data below; **C** data page S5; **D** data page S4; **E** data page S39; **F** data page S45]

2,4-Bis(benzyloxy)benzoyl chloride **A**

A solution of potassium *tert*-butoxide (3.36 g, 29.9 mmol) in EtOH (30 mL) was added dropwise to a solution of 2,4-bis(benzyloxy)benzoic acid[1] (10.01 g, 29.9 mmol) in EtOH (125 mL). The reaction mixture was stirred at rt for 18 h before being concentrated *in vacuo* to afford potassium 2,4-bis(benzyloxy)benzoate as a white solid. This was suspended in dry Et_2O (150 mL), oxalyl chloride (36.0 mL, 0.425 mol) was added, and the reaction mixture was heated at reflux for 24 h. After cooling to rt, the solids were removed by filtration and the filtrate was concentrated *in vacuo* to afford the title compound as a pale yellow solid (13.38 g, >99%) which was used without further purification, mp 74–76 °C; ν_{max} (cm^{-1}) 1777, 1599, 1599, 1564, 1501, 1193, 1128 and 738; δ_H (300 MHz, $CDCl_3$) 8.16 (1H, d, *J* 9.0, 6-H), 7.29–7.49 (10H, m, ArCH), 6.62 (1H, dd, *J* 9.0, 2.4, 5-H), 6.58 (1H, d, *J* 2.4, 3-H), 5.15 (2H, s, CH_2) and 5.10 (2H, s, CH_2); δ_C (75 MHz, $CDCl_3$) 165.3 (CO), 162.2 (C), 161.1 (C), 138.0 (CH), 135.7 (C), 135.5 (C), 128.8 (2CH), 128.6 (2CH), 128.5 (CH), 128.0 (CH), 127.5 (2CH), 126.7 (2CH), 115.0 (C), 106.5 (CH), 100.6 (CH), 70.51 (CH_2) and 70.46 (CH_2) HRMS (ES^+): found, 317.1171. $C_{21}H_{17}O_3$ (M–Cl) requires 317.1178.

2,4-Bis(benzyloxy)-*N*-(1-hydroxy-2-methylpropan-2-yl)benzamide **B**

A solution of **A** (13.12 g, 29.9 mmol) in dry CH_2Cl_2 (40 mL) was added dropwise to a 0 °C solution of 2-amino-2-methylpropan-1-ol (6.63 g, 74.4 mmol) in dry CH_2Cl_2 (100 mL). The reaction mixture was allowed to warm to rt over a period of 18 h before being poured into H_2O. The two layers were separated and the aqueous layer was re-extracted with CH_2Cl_2 (× 2). The combined organic layers were washed with brine, dried over $MgSO_4$ and concentrated *in vacuo* to afford the title compound as a pale yellow solid (11.53 g, 95%), mp 104–106 °C; ν_{max} (cm^{-1}) 3356, 3220, 1624, 1598, 1544, 1496, 1257 and 1098; δ_H (300 MHz, $CDCl_3$) 8.15 (1H, d, *J* 8.4, 6-H), 7.99 (1H, br s, CON*H*), 7.47–7.32 (10H, m, ArCH), 6.72–6.67 (2H, m, 3,5-H), 5.51 (1H, br s, OH), 5.12 (2H, s, OC*H*$_2$Ph), 5.06 (2H, s, OC*H*$_2$Ph), 3.52 (2H, s, *C*H_2OH) and 1.02 (6H, s, CMe_2) δ_C (75 MHz, $CDCl_3$) 165.5 (CO), 162.6 (C), 158.0 (C), 136.1 (C), 134.9 (C), 133.8 (CH), 129.1 (CH), 128.92 (2CH), 128.86 (2CH), 128.7 (2CH), 128.3 (CH), 127.5 (2CH), 114.6 (C), 106.5 (CH), 100.3 (CH), 71.6 (CH_2), 71.3 (CH_2), 70.3 (CH_2), 56.0 (NH*C*Me_2) and 24.5 (Me); HRMS (ES^+): found, 406.2010. $C_{25}H_{28}NO_4$ (M+H) requires 406.2018.

2-(2,4-Bis(benzyloxy)phenyl)-4,4-dimethyl-4,5-dihydro-1,3-oxazole **1**

Triethylamine (19.5 mL, 0.140 mol) was added to a solution of **B** (11.28 g, 27.8 mmol) in CH_2Cl_2 (60 mL). The resultant solution was cooled to 0 °C and methanesulfonyl chloride (4.3 mL, 55.6 mmol) was added dropwise. The reaction mixture was allowed to warm to rt over a period of 20 h before being poured into H_2O. The two layers were separated and the aqueous layer was re-extracted with CH_2Cl_2 (× 2). The combined organic layers were washed with sat. aq. $NaHCO_3$ (× 3) and brine, dried over $MgSO_4$ and concentrated *in vacuo* to afford the title compound as an orange solid (10.46 g, 97%) which was used without further purification, mp 84–86 °C; ν_{max} (cm^{-1}) 1722, 1638, 1609 and 1496; δ_H (300 MHz, $CDCl_3$) 7.70 (1H, d, *J* 8.4, 6-H), 7.53–7.50 (2H, m, CH), 7.43–7.25 (8H, m, CH), 6.62–6.56 (2H, m, 3,5-H), 5.12 (2H, s, OC*H*$_2$Ph), 5.05 (2H, s, OC*H*$_2$Ph), 4.06 (2H, s, CMe_2CH_2O) and 1.39 (6H, s, CMe_2); δ_C (75 MHz, $CDCl_3$) 161.8 (C), 160.9 (C), 158.7 (C), 136.9 (C), 136.3 (C), 132.3 (CH), 128.6 (2CH), 128.2 (2CH), 128.1 (CH), 127.50 (2CH), 127.45 (CH), 126.6 (2CH), 111.3 (C), 106.0 (CH), 101.5 (CH), 78.6 (O*C*H_2CMe_2), 70.4 (O*C*H_2Ph), 70.1 (O*C*H_2Ph), 67.3 (*C*Me_2) and 28.4 (2CH_3); HRMS (ES$^+$): found, 388.1902. $C_{25}H_{26}NO_3$ (M+H) requires 388.1913.

(5-(Benzyloxy)-2-(4,4-dimethyl-4,5-dihydrooxazol-2-yl)phenyl)(phenyl)methanone **4**

A 2.5 M solution of *n*-butyllithium in hexane (5.4 mL, 13.5 mmol) was added dropwise to a –78 °C solution of **1** (5.27 g, 13.6 mmol) in dry THF (90 mL). The resultant solution was stirred at –78 °C for 5 min before being warmed to rt over a period of 2 h. The solution was then cooled to –78 °C and bromine (0.70 mL, 13.7 mmol) was added dropwise. The reaction mixture was then allowed to warm to rt over a period of 18 h before being poured into sat. aq. NH_4Cl and extracted with Et_2O (× 3). The combined organic layers were dried over $MgSO_4$, filtered and concentrated *in vacuo*. The crude residue was purified by column chromatography (gradient elution 2:3 Et_2O:hexane to 1:9 MeOH:EtOAc) to give the title compound as an orange solid (0.97 g, 19%) which was suitable for X-ray structure determination, mp 86–88 °C; ν_{max} (cm^{-1}) 1662, 1645, 1596, 1579, 1496 and 1008; δ_H (400 MHz, $CDCl_3$) 7.85 (1H, d, *J* 8.4, CH), 7.73–7.70 (2H, m, CH), 7.51–7.46 (1H, m, CH), 7.41–7.25 (7H, m, CH), 7.11–7.07 (2H, m, CH), 5.08 (2H, OC*H*$_2$Ph), 3.53 (2H, OC*H*$_2CMe_2$) and 0.99 (6H, s, CMe_2); δ_C (75 MHz, $CDCl_3$) 195.7 (C=O), 160.3 (2C), 141.1 (C), 137.2 (C), 135.6 (C), 132.2 (CH), 130.3 (CH), 128.6 (2CH), 128.2 (2CH), 127.9 (2CH), 127.7 (CH), 127.1 (2CH), 119.1 (C), 115.8 (CH), 114.0 (CH), 78.6 (O*C*H_2CMe_2), 69.7 (O*C*H_2Ph), 67.1 (*C*Me_2) and 27.3 (2CH_3); HRMS (ES$^+$): found, 386.1740. $C_{25}H_{24}NO_3$ (M+H) requires 386.1756.

For X-ray structural data of **4** see page S188

(2-(4,4-Dimethyl-4,5-dihydrooxazol-2-yl)phenyl)(phenyl)methanol **6**

Under a nitrogen atmosphere, *n*-butyllithium (2.5 M in hexane, 6.6 mL, 16.5 mmol) was added dropwise to a stirred solution of 2-(2-(benzyloxy)phenyl)-4,4-dimethyl-4,5-dihydrooxazole **5**[2] (2.12 g, 7.53 mmol) in dry THF (75 mL). After stirring at rt for 2 h, the reaction mixture was quenched by addition of sat. aq. NH_4Cl (150 mL) and extracted with Et_2O (3 × 75 mL). The combined organic layers were dried and evaporated to give, after purification by column chromatography (SiO_2, Et_2O/hexane 1:1), at R_f 0.65, **6** (0.62 g, 29%) as orange crystals; mp 56–58 °C. The ^{1}H and ^{13}C NMR spectral data were in accordance with those previously reported.[3]

4-(Benzyloxy)-*N*-(1-hydroxy-2-methylpropan-2-yl)benzamide **D**

A solution of thionyl chloride (1.7 mL, 2.77 g, 23.3 mmol) and 4-(benzyloxy)benzoic acid[4] (2.50 g, 11.0 mmol) in toluene (25 mL) was heated at reflux for 3 h. After cooling to rt, the reaction mixture was evaporated to give 4-(benzyloxy)benzoyl chloride as an off-white solid which was used immediately without further purification.

A solution of 4-(benzyloxy)benzoyl chloride (assuming 11.0 mmol) in CH_2Cl_2 (30 mL) was added dropwise to a stirred 0 °C solution of 2-amino-2-methylpropan-1-ol (2.19 g, 24.6 mmol) in CH_2Cl_2 (30 mL). Once the addition was complete, the reaction mixture was allowed to warm to rt for 18 h before being poured into water. The two layers were separated and the aqueous layer was re-extracted with CH_2Cl_2 (× 2). The combined organic layers were washed successively with 2 M HCl, 2 M NaOH and water before being dried and evaporated. Recrystallisation of the residue (EtOAc/hexane) gave **D** (2.41 g, 73%) as colourless crystals, mp 108–110 °C (lit.[5] 112–113 °C); ν_{max}/cm^{-1} 3264, 3164, 1734, 1627, 1607, 1574, 1507, 1267, 1185, 1067, 1023, 844, 736, 697 and 616; δ_H (400 MHz) 7.69 (2 H, d, *J* 8.8, ArH), 7.43–7.31 (5 H, m, Ph), 6.98 (2 H, d, *J* 8.8, ArH), 6.13 (1 H, br s, NH), 5.10 (2 H, s, OCH_2Ph), 4.93 (1 H, br s, OH), 3.67 (2 H, s, CH_2OH) and 1.40 (6 H, s, CMe_2); δ_C (100 MHz) 167.9 (C=O), 161.4 (C–O), 136.2 (C), 128.7 (2CH), 128.6 (2CH), 128.2 (CH), 127.4 (2CH), 114.7 (2CH), 70.9 (CH_2), 70.1 (CH_2), 56.4 (CMe_2) and 24.8 ($2CH_3$); HRMS (ESI^+): found, 322.1405. $C_{18}H_{21}NaNO_3$ (M+Na) requires 322.1414.

2-(4-Benzyloxy)phenyl)-4,4-dimethyl-4,5-dihydrooxazole **9**

A mixture of 4-(benzyloxy)-*N*-(1-hydroxy-2-methylpropan-2-yl)benzamide **D** (1.94 g, 6.48 mmol) and thionyl chloride (5.0 mL, 8.16 g, 68.5 mmol) was stirred at rt for 1 h. The reaction mixture was concentrated *in vacuo* and the residue was partitioned between 2 M NaOH (50 mL) and toluene (50 mL). The organic layer was washed with water (50 mL) before being dried and evaporated. Purification of the crude residue by Kugelrohr distillation (235 °C/20 Torr) gave **9** (1.54 g, 84%) as a colourless oil which solidified on standing, mp 63–66 °C; ν_{max}/cm^{-1} 1720, 1650, 1609, 1511, 1253, 1168, 1066, 1021, 966, 840, 742, 701 and 637; δ_H (500 MHz) 7.91 (2 H, d, *J* 9.0, ArH), 7.42 (2 H, d, *J* 7.0, Ph), 7.38 (2 H, t, *J* 7.0, Ph), 7.35–7.31 (1 H, m, Ph), 6.98 (2 H, d, *J* 9.0, ArH), 5.10 (2 H, s, OCH_2Ph), 4.11 (2 H, s, OCH_2CMe_2) and 1.39 (6 H, s, CMe_2); δ_C (125 MHz) 162.4 (C), 161.3 (C), 136.2 (C), 130.1 (2CH), 128.5 (2CH), 128.0 (CH), 127.4 (2CH), 119.9 (C), 114.5 (2CH), 79.2 (CH_2), 69.9 (CH_2), 67.0 (CMe_2) and 28.2 ($2CH_3$); HRMS (ESI^+): found, 282.1485. $C_{18}H_{20}NO_2$ (M+H) requires 282.1489.

(4-(4,4-Dimethyl-4,5-dihydrooxazol-2-yl)phenyl)(phenyl)methanol **10**

Under a nitrogen atmosphere, *n*-butyllithium (2.5 M in hexane, 0.80 mL, 2.00 mmol) was added dropwise to a stirred solution of 2-(4-benzyloxy)phenyl)-4,4-dimethyl-4,5-dihydrooxazole **9** (0.2807 g, 1.00 mmol) in dry THF (10 mL). After stirring at rt for 2 h, the reaction mixture was quenched by addition of sat. aq. NH_4Cl (40 mL) and extracted with Et_2O (3 × 20 mL). The combined organic layers were dried and evaporated and the crude residue was purified by preparative TLC (SiO_2, Et_2O/hexane 4:1) to give, at R_f 0.40, **10** (29.8 mg, 11%) as a pale yellow solid, mp 143–147 °C; ν_{max}/cm^{-1} 3171, 2967, 1641, 1357, 1250, 1179, 1074, 1017, 965, 745 and 704; δ_H (500 MHz) 7.88 (2 H, d, *J* 8.5, ArH), 7.41 (2 H, d, *J* 8.5, ArH), 7.35–7.30 (4 H, m, Ph), 7.27–7.25 (1 H, m, Ph), 5.85 (1 H, s, CHOH), 4.09 (2 H, s, CH_2), 3.53 (1 H, br s, OH) and 1.36 (6 H, s, CMe_2); δ_C (125 MHz) 162.1 (C=N), 147.1 (C), 143.5 (C), 128.5 (2CH), 128.4 (2CH), 127.7 (CH), 126.6 (2CH), 126.3 (2CH), 79.1 (CH_2), 75.7 (CHOH), 67.5 (CMe_2) and 28.3 ($2CH_3$); HRMS (NSI^+): found, 282.1487. $C_{18}H_{20}NO_2$ (M+H) requires 282.1489.

3-(Benzyloxy)-*N*-(1-hydroxy-2-methylpropan-2-yl)benzamide **C**

A solution of 3-(benzyloxy)benzoyl chloride[6] (10.01 g, 40.6 mmol) in CH_2Cl_2 (50 mL) was added dropwise to a stirred 0 °C solution of 2-amino-2-methylpropan-1-ol (7.25 g, 81.3 mmol) in CH_2Cl_2 (80 mL). Once the addition was complete, the reaction mixture was allowed to warm to rt for 18 h before being poured into water. The two layers were separated and the aqueous layer was re-extracted with CH_2Cl_2 (× 2). The combined organic layers were washed successively with 2 M HCl, 2 M NaOH and water before being dried and evaporated to give **C** (11.51 g, 95%) as a pale yellow oil which solidified on standing and was used without further purification, mp 84–88 °C; ν_{max}/cm^{-1} 3336, 1717, 1630, 1578, 1113, 1065, 1032, 913, 892, 854 and 722; δ_H (300 MHz) 7.45–7.24 (8 H, m, ArH and Ph), 7.10 (1 H, ddd, *J* 8.1, 2.7, 1.2, ArH), 6.20 (1 H, br s, NH), 5.10 (2 H, s, OCH_2Ph), 4.05 (1 H, br s, OH), 3.69 (2 H, s, CH_2OH) and 1.40 (6 H, s, CMe_2); δ_C (100 MHz) 168.0 (C=O), 158.7 (C–O), 136.4 (C), 136.2 (C), 129.5 (CH), 128.5 (2CH), 128.0 (CH), 127.4 (2CH), 119.0 (CH), 118.2 (CH), 113.3 (CH), 70.4 (CH_2), 70.0 (CH_2), 56.2 (CMe_2) and 24.3 ($2CH_3$); HRMS (ESI^+): found, 322.1409. $C_{18}H_{21}NaNO_3$ (M+Na) requires 322.1414.

2-(3-Benzyloxy)phenyl)-4,4-dimethyl-4,5-dihydrooxazole **11**

Methanesulfonyl chloride (6.0 mL, 8.88 g, 77.5 mmol) was added dropwise to a stirred 0 °C solution of 3-(benzyloxy)-*N*-(1-hydroxy-2-methylpropan-2-yl)benzamide **C** (11.11 g, 37.1 mmol) and triethylamine (26 mL, 18.88 g, 0.187 mol) in CH_2Cl_2 (100 mL) and the reaction mixture was allowed to warm to rt for 20 h. The mixture was poured into water (100 mL), the two layers were separated and the aqueous layer re-extracted with CH_2Cl_2 (2 × 50 mL). The combined organic layers were washed with 2 M HCl, 2 M NaOH and water before being dried and evaporated. The crude residue was purified by Kugelrohr distillation (235 °C/20 Torr) to give **11** (6.70 g, 64%) as a pale yellow oil which slowly solidified on standing, mp 49–52 °C; ν_{max}/cm^{-1} 1721, 1652, 1584, 1232, 1196, 1107, 1058, 1020, 896, 858, 795, 722 and 616; δ_H (500 MHz) 7.60 (1 H, dd, *J* 2.5, 1.5, ArH), 7.56 (1 H, dt, *J* 7.5, 1.3, ArH), 7.43 (2 H, d, *J* 7.5, Ph), 7.38 (2 H, t, *J* 7.5, Ph), 7.34–7.30 (2 H, m, ArH and Ph), 7.09 (1 H, ddd, *J* 8.5, 2.5, 1.0, ArH), 5.10 (2 H, s, OCH_2Ph), 4.12 (2 H, s, CH_2) and 1.40 (6 H, s, CMe_2); δ_C (125 MHz) 162.1 (C), 158.7 (C), 136.7 (C), 129.4 (CH), 129.2 (C), 128.5 (2CH), 128.0 (CH), 127.5 (2CH), 121.0 (CH), 118.8 (CH), 113.6 (CH), 79.2 (CH_2), 70.1 (OCH_2Ph), 67.6 (CMe_2) and 28.4 ($2CH_3$); HRMS (ESI^+): found, 282.1478. $C_{18}H_{20}NO_2$ (M+H) requires *M*, 282.1489.

2-(Benzyloxy)phenyl *N,N*-diethylcarbamate **12**

Diethylcarbamoyl chloride (6.4 mL, 6.85 g, 50.5 mmol) was added to a stirred solution of 2-(benzyloxy)phenol **13**[7] (8.02 g, 40.1 mmol), triethylamine (7.0 mL, 5.08 g, 50.2 mmol) and DMAP (1.00 g, 8.19 mmol) in THF (400 mL) and the reaction mixture was heated at reflux for 18 h. After cooling to rt and evaporation of the solvent, the residue was dissolved in Et_2O (200 mL) and washed with 2 M HCl (200 mL), 2 M NaOH (200 mL) and brine (100 mL) before being dried and evaporated. The crude residue was purified by filtration through a silica plug (CH_2Cl_2) followed by trituration (EtOAc/hexane) to give **12** (6.38 g, 53%) as pale yellow crystals, mp 54–56 °C; ν_{max}/cm^{-1} 1731, 1605, 1502, 1259, 1194, 1157, 1039, 961, 866, 822, 743 and 698; δ_H (500 MHz) 7.40 (2 H, d, *J* 7.3, Ph), 7.33 (2 H, t, *J* 7.3, Ph), 7.31–7.27 (1 H, m, Ph), 7.15–7.12 (2 H, m, ArH), 7.00–6.98 (1 H, m, ArH), 6.93 (1 H, td, *J* 7.8, 1.5, ArH), 5.04 (2 H, s, OCH_2), 3.38 (2 H, q, *J* 7.0, NCH_2), 3.33 (2 H, q, *J* 7.0, NCH_2) and 1.12 (6 H, t, *J* 7.0, CH_3); δ_C (75 MHz) 154.0 (C), 150.7 (C), 141.0 (C), 136.8 (C), 128.2 (2CH), 127.7 (CH), 127.3 (2CH), 126.0 (CH), 123.2 (CH), 120.9

(CH), 113.6 (CH), 70.4 (OCH_2), 42.0 (NCH_2), 41.8 (NCH_2), 13.8 (CH_3) and 13.2 (CH_3); HRMS (ESI^+): found, 322.1409. $C_{18}H_{21}NaNO_3$ (M+Na) requires 322.1414.

Attempted [1,2]-Wittig Rearrangement of 2-(Benzyloxy)phenyl *N,N*-diethylcarbamate **12**
Under a nitrogen atmosphere, *n*-butyllithium (2.5 M in hexane, 0.40 mL, 1.00 mmol) was added dropwise to a stirred solution of 2-(benzyloxy)phenyl *N,N*-diethylcarbamate **12** (0.15 g, 0.50 mmol) in dry THF (5 mL). After stirring at rt for 4 h, the reaction mixture was poured into sat. aq. NH_4Cl (20 mL) and extracted with Et_2O (3 × 20 mL). The combined organic layers were dried and evaporated to give, after purification of the crude residue by preparative TLC (SiO_2, Et_2O/hexane 3:1), at R_f 0.85, **13** (18.2 mg, 18%) as a colourless oil; spectroscopic data as previously reported.[7] This was followed by a second fraction, at R_f 0.30, to give *N,N*-diethylpentanamide **14** (6.0 mg, 8%) as a brown oil; δ_H (400 MHz) 3.37 (2 H, q, *J* 7.2, NCH_2), 3.31 (2 H, q, *J* 7.2, NCH_2), 2.31–2.27 (2 H, m, C(O)CH_2), 1.67–1.59 (2 H, m, C(O)CH_2CH_2), 1.41–1.31 (2 H, m, C(O)$CH_2CH_2CH_2$), 1.17 (3 H, t, *J* 7.2, NCH_2CH_3), 1.11 (3 H, t, *J* 7.2, NCH_2CH_3) and 0.93 (3 H, t, *J* 7.4, C(O)$CH_2CH_2CH_2CH_3$). The ^1H NMR spectral data was consistent with that previously reported.[8]

2-(Benzyloxy)phenyl *N*-phenylcarbamate **15**
A solution of phenyl isocyanate (1.35 mL, 1.48 g, 12.4 mmol) in CH_2Cl_2 (20 mL) was added dropwise to a stirred solution of 2-(benzyloxy)phenol **13** (2.51 g, 12.5 mmol) and triethylamine (1.8 mL, 1.31 g, 12.9 mmol) in CH_2Cl_2 (15 mL). Once the addition was complete, the reaction mixture was stirred at rt for 3 h before being washed with 2 M HCl (50 mL), 2 M NaOH (50 mL) and brine (20 mL). The organic layer was dried and evaporated and the crude residue was recrystallised (PhMe) to give **15** (1.90 g, 48%) as tan-coloured crystals, mp 139–142 °C; ν_{max}/cm^{-1} 3297, 1721, 1601, 1547, 1319, 1226, 1189, 1110, 1009, 900, 755 and 728; δ_H (500 MHz) 7.42 (2 H, d, *J* 8.0, ArH), 7.37 (2 H, dd, *J* 6.5, 3.0, ArH), 7.32 (2 H, t, *J* 8.0, ArH), 7.24–7.16 (5 H, m, ArH), 7.12–7.08 (1 H, m, ArH), 7.02 (1 H, dd, *J* 8.3, 1.3, ArH), 7.01 (1 H, br s, NH), 6.97 (1 H, td, *J* 7.5, 1.3, ArH) and 5.09 (2 H, s, CH_2); δ_C (75 MHz, 54 °C) 151.5 (C), 151.0 (C), 140.5 (C), 137.7 (C), 136.8 (C), 129.0 (2CH), 128.4 (2CH), 127.8 (CH), 127.3 (2CH), 126.7 (CH), 123.8 (CH), 123.3 (CH), 121.3 (CH), 119.2 (2CH), 114.5 (CH) and 71.1 (CH_2); HRMS (ESI^+): found, 342.1092. $C_{20}H_{17}NaNO_3^+$ (M+Na) requires 342.1101.

2-(Benzyloxy)-*N,N*-diethylbenzamide **16**
A solution of 2-(benzyloxy)benzoyl chloride[2] (5.00 g, 20.3 mmol) in toluene (30 mL) was added dropwise to a stirred 0 °C solution of diethylamine (6.4 mL, 4.52 g, 61.9 mmol) in toluene (10 mL). Once the addition was complete, the reaction mixture was allowed to warm to rt for 1 h before being poured into water and washed with 2 M NaOH and brine. The organic layer was dried and evaporated to afford the crude amide product which gave, after purification by Kugelrohr distillation (176 °C/20 Torr), **16** (3.87 g, 67%) as a pale yellow oil; ν_{max}/cm^{-1} 2974, 2934, 1635, 1447, 1381, 1294, 1244, 1162, 1124, 1088, 1024, 943, 878, 753, 698 and 632; δ_H (500 MHz) 7.40 (2 H, d, *J* 7.3, Ph), 7.35 (2 H, t, *J* 7.3, Ph), 7.32–7.28 (2 H, m, ArH), 7.24 (1 H, dd, *J* 7.5, 1.5, ArH), 6.99 (1 H, t, *J* 7.5, ArH), 6.96 (1 H, d, *J* 8.0, ArH), 5.11 and 5.07 (2 H, AB pattern, J_{AB} 11.0, OCH_2), 3.83–3.77 (1 H, m, NCH), 3.33–3.27 (1 H, m, NCH), 3.23–3.08 (2 H, m, NCH_2), 1.17 (3 H, t, *J* 7.0, CH_3) and 1.00 (3 H, t, *J* 7.0, CH_3); δ_C (125 MHz) 168.6 (C=O), 154.2 (C–O), 136.7 (C), 129.8 (CH), 128.4 (2CH), 127.7 (CH), 127.53 (CH), 127.48 (C), 127.0 (2CH), 121.2 (CH),

112.5 (CH), 70.2 (OCH_2), 42.6 (NCH_2), 38.7 (NCH_2), 14.0 (CH_3) and 12.7 (CH_3); HRMS (ESI^+): found, 284.1639. $C_{18}H_{22}NO_2$ (M+H) requires 284.1645.

Attempted [1,2]-Wittig Rearrangement of 2-(Benzyloxy)-*N,N*-diethylbenzamide **16**

Under a nitrogen atmosphere, *n*-butyllithium (2.5 M in hexane, 0.40 mL, 1.00 mmol) was added dropwise to a stirred –78 °C solution of 2-(benzyloxy)-*N,N*-diethylbenzamide **16** (0.14 g, 0.49 mmol) in dry toluene (5 mL). After stirring at –78 °C for 2 h, the reaction mixture was quenched by addition of sat. aq. NH_4Cl (20 mL) and, after separation of the two layers, the aqueous layer was further extracted with Et_2O (2 × 20 mL). The combined organic layers were dried and evaporated to give, after purification of the crude residue by preparative TLC (SiO_2, Et_2O/hexane 1:1), at R_f 0.90, 1-(2-(benzyloxy)phenyl)pentan-1-one **17** (47.2 mg, 36%) as a yellow oil; δ_H (300 MHz) 7.66 (1 H, dd, *J* 8.0, 1.7, ArH), 7.45–7.33 (6 H, m, ArH and Ph), 7.03–6.98 (2 H, m, ArH), 5.14 (2 H, s, OCH_2), 2.97–2.92 (2 H, m, C(O)CH_2), 1.65–1.55 (2 H, m, C(O)$CH_2C\mathit{H}_2$), 1.30–1.18 (2 H, m, $C\mathit{H}_2CH_3$) and 0.82 (3 H, t, *J* 7.4, CH_3); δ_C (125 MHz) 203.4 (C=O), 157.4 (C–O), 136.2 (C), 133.0 (CH), 130.2 (CH), 129.2 (C), 128.6 (2CH), 128.2 (CH), 127.6 (2CH), 120.9 (CH), 112.6 (CH), 70.6 (OCH_2), 43.7(CH_2), 26.5 (CH_2), 22.4 (CH_2) and 13.8 (CH_3). The ^1H and ^{13}C NMR spectral data was in accordance with that previously reported.[9]

2-(Benzyloxy)-*N,N*-diisopropylbenzamide **18**

A solution of 2-(benzyloxy)benzoyl chloride[2] (10.82 g, 43.9 mmol) in toluene (60 mL) was added dropwise to a stirred 0 °C solution of diisopropylamine (18.5 mL, 13.36 g, 0.132 mol) in toluene (20 mL). Once the addition was complete, the reaction mixture was allowed to warm to rt for 1 h before being poured into water and washed with 2 M NaOH and brine. The organic layer was dried and evaporated to afford the crude amide product which gave, after recrystallisation (hexane), **18** (11.52 g, 84%) as colourless crystals, mp 80–83 °C; ν_{max}/cm^{-1} 1724, 1623, 1341, 1238, 1164, 1120, 1035, 919, 858, 842, 750 and 699; δ_H (500 MHz) 7.43 (2 H, d, *J* 7.5, Ph), 7.34 (2 H, t, *J* 7.5, Ph), 7.30–7.26 (2 H, m, Ph and ArH), 7.18 (1 H, d, *J* 7.0, ArH), 6.98 (1 H, t, *J* 7.5, ArH), 6.94 (1 H, d, *J* 8.5, ArH), 5.11 and 5.05 (2 H, AB pattern, J_{AB} 11.5, OCH_2), 3.76–3.68 (1 H, m, $C\mathit{H}Me_2$), 3.48–3.40 (1 H, m, $C\mathit{H}Me_2$), 1.55 (3 H, d, *J* 6.5, CH_3), 1.49 (3 H, d, *J* 6.5, CH_3), 1.03 (3 H, d, *J* 6.5, CH_3) and 1.02 (3 H, d, *J* 6.5, CH_3); δ_C (125 MHz) 168.3 (C=O), 154.1 (C–O), 136.7 (C), 129.3 (CH), 129.1 (C), 128.3 (2CH), 127.7 (CH), 127.1 (2CH), 126.8 (CH), 121.2 (CH), 112.4 (CH), 70.1 (OCH_2), 50.8 (NCH), 45.6 (NCH), 20.8 ($2CH_3$), 20.4 (CH_3) and 20.1 (CH_3); HRMS (ESI^+): found, 312.1953. $C_{20}H_{26}NO_2$ (M+H) requires 312.1958.

Attempted [1,2]-Wittig Rearrangement of **18** in THF giving **19**

Under a nitrogen atmosphere, *n*-butyllithium (2.5 M in hexane, 0.80 mL, 2.00 mmol) was added dropwise to a stirred solution of 2-(benzyloxy)-*N,N*-diisopropylbenzamide **18** (0.3117 g, 1.00 mmol) in dry THF (10 mL). After stirring at rt for 2 h, the reaction mixture was poured into water (40 mL) and extracted with Et_2O (3 × 30 mL). The combined organic layers were dried and evaporated to give, after purification of the crude residue by repeated preparative TLC (SiO_2, Et_2O/hexane 2:3 then CH_2Cl_2/hexane 4:1), 1-(2-hydroxyphenyl)-2-phenylethane-1,2-dione **19** (35.0 mg, 15%) as a colourless solid, mp 72–74 °C; (lit.[10] 74 °C); δ_H (400 MHz) 11.40 (1 H, s, OH), 7.99–7.96 (2 H, m, ArH), 7.71–7.66 (1 H, m, ArH), 7.59–7.51 (3 H, m, ArH), 7.47 (1 H, dd, *J* 8.0, 1.6, ArH), 7.09 (1 H, dd, *J* 8.6, 0.6, ArH) and 6.91–6.87 (1 H, m, ArH); δ_C (100 MHz) 199.3 (4ry, C=O), 192.0 (4ry, C=O), 163.4 (4ry, C–O), 138.1 (CH), 135.2 (CH), 132.7 (4ry, Ar C), 132.4

(CH), 130.0 (2 × Ph CH), 129.1 (2 × Ph CH), 119.7 (CH), 118.7 (CH) and 116.8 (4ry, Ar C). The ^{1}H and ^{13}C NMR spectral data was in accordance with that previously reported.[11]

Attempted [1,2]-Wittig Rearrangement of **18** in PhMe giving **20**

Under a nitrogen atmosphere, *n*-butyllithium (2.5 M in hexane, 0.80 mL, 2.00 mmol) was added dropwise to a stirred solution of 2-(benzyloxy)-*N,N*-diisopropylbenzamide **18** (0.3115 g, 1.00 mmol) in dry toluene (10 mL). After stirring at rt for 2 h, the reaction mixture was poured into water (40 mL), the two layers were separated and the aqueous layer was further extracted with Et_2O (2 × 30 mL). The combined organic layers were dried and evaporated to give, after purification of the crude residue by repeated preparative TLC (SiO_2, Et_2O/hexane 2:3 then hexane), *N,N*-diisopropyl-2-phenylbenzofuran-3-amine **20** (65.0 mg, 22%) as a brown oil; ν_{max}/cm^{-1} 1601, 1450, 1381, 1250, 1207, 752 and 694; δ_H (400 MHz) 8.47 (2 H, d, *J* 7.6, ArH), 7.64 (1 H, d, *J* 7.6, ArH), 7.47–7.45 (1 H, m, ArH), 7.43–7.38 (2 H, m, ArH), 7.31–7.27 (1 H, m, ArH), 7.25–7.21 (1 H, m, ArH), 7.18–7.14 (1 H, m, ArH), 3.81 (2 H, septet, *J* 6.4, C*H*Me$_2$) and 1.09 (12 H, d, *J* 6.4, CH*Me*$_2$); δ_C (100 MHz) 153.1 (C), 151.2 (C), 131.6 (C), 130.6 (C), 128.0 (2CH), 127.7 (CH), 126.4 (2CH), 124.1 (C), 123.8 (CH), 122.1 (2CH), 111.4 (CH), 49.3 (2NCH), 21.5 (4CH_3); HRMS (CI^+): found, 294.1855. $C_{20}H_{24}NO$ (M+H) requires 294.1852.

2-(Benzyloxy)-*N*-butylbenzamide **21a**

A solution of 2-(benzyloxy)benzoyl chloride[2] (10.85 g, 44.0 mmol) in toluene (60 mL) was added dropwise to a stirred 0 °C solution of *n*-butylamine (12.1 mL, 8.95 g, 0.122 mol) in toluene (60 mL). Once the addition was complete, the reaction mixture was allowed to warm to rt for 1 h before being poured into water and washed with 2 M NaOH and brine. The organic layer was dried and evaporated to afford, after recrystallisation (EtOAc/hexane), **21a** (9.34 g, 75%) as colourless crystals, mp 52–54 °C; ν_{max}/cm^{-1} 3380, 1648, 1599, 1558, 1292, 1238, 1164, 1101, 1005, 865, 752 and 700; δ_H (500 MHz) 8.25 (1 H, dd, *J* 7.8, 1.8, ArH), 7.88 (1 H, br s, NH), 7.47–7.38 (6 H, m, ArH and Ph), 7.12–7.08 (1 H, m, ArH), 7.06 (1 H, d, *J* 8.5, ArH), 5.15 (2 H, s, OCH_2), 3.34 (2 H, td, *J* 7.0, 5.5, NCH_2), 1.35–1.29 (2 H, m, NCH_2CH_2), 1.19–1.11 (2 H, m, CH_2CH_3) and 0.80 (3 H, t, *J* 7.3, CH_3); δ_C (75 MHz) 164.9 (C=O), 156.8 (C–O), 135.5 (C), 132.5 (CH), 132.4 (CH), 128.9 (2CH), 128.8 (CH), 128.2 (2CH), 122.0 (C), 121.6 (CH), 112.4 (CH), 71.4 (OCH_2), 39.4 (NCH_2), 31.2 (CH_2), 20.0 (CH_2) and 13.7 (CH_3); HRMS (ESI^+): found, 306.1455. $C_{18}H_{21}NaNO_2$ (M+Na) requires 306.1465.

N-Butyl-2-(hydroxy(phenyl)methyl)benzamide **22a**, Anthraquinone **24a** and 3-Phenylphthalide **23a**

Under a nitrogen atmosphere, *n*-butyllithium (2.6 mL, 6.50 mmol) was added dropwise to a stirred solution of 2-(benzyloxy)-*N*-butylbenzamide **21a** (0.5678 g, 2.00 mmol) in dry THF (20 mL). After stirring at rt for 2 h, the reaction mixture was quenched by addition of sat. aq. NH_4Cl and extracted with Et_2O (× 3). The combined organic layers were washed with 2 M NaOH and water before being dried and evaporated to give **22a** as a pale yellow oil; ν_{max}/cm^{-1} 3296, 3064, 2931, 1635, 1540, 1450, 1303, 1228, 1104, 1024, 757 and 699; δ_H (500 MHz) 7.38 (2 H, t, *J* 7.3, ArH), 7.29–7.17 (7 H, m, ArH and Ph), 6.31 (1 H, t, *J* 5.3, NH), 5.79 (1 H, s, C*H*OH), 3.21–3.14 (1 H, m, NCH_2), 3.12–3.05 (1 H, m, NCH_2), 1.29–1.23 (2 H, m, NCH_2CH_2), 1.22–1.15 (2 H, m, CH_2CH_3) and 0.85 (3 H, t, *J* 7.0, CH_3); δ_C (125 MHz) 170.8 (C=O), 143.1 (C), 142.7 (C), 135.8 (C), 130.6 (CH), 129.9 (CH), 127.74 (CH), 127.71 (2CH), 127.69 (CH), 126.7 (CH), 126.2 (2CH),

74.9 (CHOH), 39.7 (NCH_2), 31.1 (CH_2), 19.9 (CH_2) and 13.6 (CH_3); HRMS (ESI$^+$): found, 306.1456. $C_{18}H_{21}NaNO_2$ (M+Na) requires *M*, 306.1465.
On standing at rt in EtOAc solution for 2–3 months, an intramolecular cyclisation occurred to give, after purification by column chromatography (SiO_2, Et_2O/hexane 2:3), at R_f 0.80, **24a** (17.5 mg, 4%) as yellow needles, mp 275–279 °C; (lit.[12] 275 °C); δ_H (500 MHz) 8.34–8.30 (4 H, m, ArH) and 7.83–7.79 (4 H, m, ArH). The ^{1}H NMR spectral data was in accordance with that previously reported.[13]
This was followed by a second fraction to give, at R_f 0.55, **23a** (0.3350 g, 80%) as tan-coloured crystals, mp 113–116 °C; (lit.[14] 115.5 °C); δ_H (500 MHz) 7.96 (1 H, d, *J* 7.5, ArH), 7.65 (1 H, td, *J* 7.5, 1.0, ArH), 7.55 (1 H, t, *J* 7.5, ArH), 7.39–7.36 (3 H, m, ArH), 7.33 (1 H, dd, *J* 7.8, 0.8, ArH), 7.29–7.26 (2 H, m, ArH) and 6.41 (1 H, s, CHPh). The ^{1}H NMR spectral data was in accordance with that previously reported.[15]
Alternatively, the following literature procedure[16] may be employed:– A mixture of *N*-butyl-2-(hydroxy(phenyl)methyl)benzamide **22a** (prepared as above from 1.14 g **21a**, assuming 4.02 mmol) and *p*-toluenesulfonic acid monohydrate (1.55 g, 8.15 mmol) in toluene (80 mL) was heated at reflux for 1 h. After cooling to rt, the reaction mixture was washed with water (50 mL), 2 M NaOH (50 mL) and brine (50 mL) before being dried and evaporated. The crude residue was purified by column chromatography (SiO_2, gradient elution, Et_2O/hexane 1:4 to Et_2O) to give **23a** (0.76 g, 90%) as tan-coloured crystals.

N-Butylsalicylamide **25**
Following a literature procedure,[17] a mixture of methyl salicylate (25.00 g, 0.164 mol) and *n*-butylamine (85 mL, 62.90 g, 0.860 mol) in methanol (250 mL) was heated at reflux for 18 h before being concentrated *in vacuo*. The residue was acidified to pH 1 by addition of 2 M HCl and extracted with CH_2Cl_2 (3 × 100 mL). The combined organic layers were washed with water (100 mL) before being dried and evaporated to give **25** (31.21 g, 98%) as a pale yellow oil which was used without further purification; δ_H (500 MHz) 12.40 (1 H, s, OH), 7.41–7.37 (1 H, m, ArH), 7.33 (1 H, dd, *J* 8.0, 1.0, ArH), 6.99 (1 H, d, *J* 8.5, ArH), 6.86–6.82 (1 H, m, ArH), 6.27 (1 H, br s, NH), 3.46 (2 H, td, *J* 7.3, 5.5, NCH_2), 1.65–1.59 (2 H, m, NCH_2CH_2), 1.46–1.39 (2 H, m, CH_2CH_3) and 0.97 (3 H, t, *J* 7.3, CH_3); δ_C (125 MHz) 169.9 (C=O), 161.5 (C–O), 134.1 (CH), 125.2 (CH), 118.6 (CH), 118.5 (CH), 114.3 (C), 39.4 (NCH_2), 31.5 (CH_2), 20.1 (CH_2) and 13.7 (CH_3). The ^{1}H NMR spectral data was in accordance with that previously reported.[18] ^{13}C NMR spectral data is reported for the first time.

N-Butyl-2-((4-methylbenzyl)oxy)benzamide **21b**
A solution of 4-methylbenzyl chloride (1.4 mL, 1.49 g, 10.6 mmol), *N*-butylsalicylamide **25** (1.94 g, 10.0 mmol) and potassium carbonate (4.19 g, 30.3 mmol) in DMF (10 mL) was heated at 100 °C for 18 h before being poured into water and extracted with CH_2Cl_2 followed by Et_2O (× 3). The combined organic layers were washed with brine (× 5) and 2 M NaOH before being dried and evaporated to give, after recrystallisation (EtOAc/hexane), **21b** (2.74 g, 92%) as colourless crystals, mp 73–75 °C; ν_{max}/cm^{-1} 3375, 2947, 1645, 1553, 1474, 1236, 997, 953, 802, 750, 592 and 517; δ_H (500 MHz) 8.25 (1 H, dd, *J* 7.8, 1.8, ArH), 7.91 (1 H, br s, NH), 7.45–7.42 (1 H, m, ArH), 7.34 (2 H, d, *J* 8.0, ArH), 7.23 (2 H, d, *J* 8.0, ArH), 7.11–7.08 (1 H, m, ArH), 7.06 (1 H, d, *J* 8.0, ArH), 5.10 (2 H, s, OCH_2), 3.34 (2 H, td, *J* 7.0, 5.5, NCH_2), 2.39 (3 H, s, CH_3), 1.35–1.29 (2 H, m, NCH_2CH_2), 1.18–1.10 (2 H, m, CH_2CH_3) and 0.80 (3 H, t, *J* 7.5, CH_2CH_3); δ_C (125 MHz)

165.0 (C=O), 156.8 (C–O), 138.7 (C), 132.48 (CH), 132.46 (C), 132.3 (CH), 129.5 (2CH), 128.3 (2CH), 121.8 (C), 121.4 (CH), 112.4 (CH), 71.3 (OCH_2), 39.4 (NCH_2), 31.2 (CH_2), 21.2 (CH_3), 20.0 (CH_2) and 13.7 (CH_3); HRMS (NSI^+): found, 298.1800. $C_{19}H_{24}NO_2$ (M+H) requires 298.1802.

N-Butyl-2-((2-methylbenzyl)oxy)benzamide **21c**
Preparation as for **21b** using 2-methylbenzyl bromide (1.4 mL, 1.93 g, 10.4 mmol), *N*-butylsalicylamide **25** (1.94 g, 10.0 mmol) and potassium carbonate (4.19 g, 30.3 mmol) heated at 100 °C in DMF (10 mL) gave, after recrystallisation (EtOAc/hexane), **21c** (2.39 g, 80%) as colourless crystals, mp 65–68 °C; ν_{max}/cm^{-1} 3379, 2930, 1638, 1599, 1472, 1229, 995, 746, 600 and 530; δ_H (400 MHz) 8.26 (1 H, dd, *J* 8.0, 2.0, ArH), 7.82 (1 H, br s, NH), 7.47–7.43 (1 H, m, ArH), 7.38 (1 H, d, *J* 7.6, ArH), 7.34–7.30 (1 H, m, ArH), 7.27–7.22 (2 H, m, ArH), 7.12–7.08 (2 H, m, ArH), 5.14 (2 H, s, OCH_2), 3.30 (2 H, td, *J* 6.8, 5.2, NCH_2), 2.39 (3 H, s, CH_3), 1.28–1.20 (2 H, m, NCH_2CH_2), 1.11–1.02 (2 H, m, CH_2CH_3) and 0.75 (3 H, t, *J* 7.2, CH_2CH_3); δ_C (125 MHz) 164.9 (C=O), 156.9 (C–O), 137.1 (C), 133.4 (C), 132.5 (CH), 132.3 (CH), 130.7 (CH), 129.6 (CH), 129.1 (CH), 126.3 (CH), 121.8 (C), 121.5 (CH), 112.3 (CH), 69.7 (OCH_2), 39.3 (NCH_2), 31.0 (CH_2), 19.9 (CH_2), 18.8 (CH_3) and 13.7 (CH_3); HRMS (NSI^+): found, 298.1797. $C_{19}H_{24}NO_2$ (M+H) requires 298.1802.

N-Butyl-2-((4-methoxybenzyl)oxy)benzamide **21d**
Preparation as for **21b** using 4-methoxybenzyl bromide (2.02 g, 10.0 mmol), *N*-butylsalicylamide **25** (1.93 g, 9.99 mmol) and potassium carbonate (4.20 g, 30.4 mmol) at rt in DMF (10 mL) gave, after recrystallisation (EtOAc/hexane), **21d** (2.67 g, 85%) as colourless crystals, mp 103–106 °C; ν_{max}/cm^{-1} 3397, 2955, 1639, 1516, 1288, 1225, 1177, 1030, 997, 866, 835, 808, 760, 656, 563 and 519; δ_H (500 MHz) 8.25 (1 H, dd, *J* 8.0, 2.0, ArH), 7.91 (1 H, br s, NH), 7.46–7.42 (1 H, m, ArH), 7.38 (2 H, d, *J* 8.5, ArH), 7.11–7.08 (1 H, m, ArH), 7.06 (1 H, d, *J* 8.0, ArH), 6.95 (2 H, d, *J* 8.5, ArH), 5.08 (2 H, s, OCH_2), 3.84 (3 H, s, OCH_3), 3.33 (2 H, td, *J* 7.0, 5.5, NCH_2), 1.34–1.28 (2 H, m, NCH_2CH_2), 1.17–1.09 (2 H, m, CH_2CH_3) and 0.80 (3 H, t, *J* 7.3, CH_2CH_3); δ_C (125 MHz) 165.0 (C=O), 160.0 (C–O), 156.9 (C–O), 132.5 (CH), 132.3 (CH), 130.0 (2CH), 127.5 (C), 121.8 (C), 121.4 (CH), 114.2 (2CH),112.4 (CH), 71.1 (OCH_2), 55.3 (OCH_3), 39.4 (NCH_2), 31.2 (CH_2), 20.0 (CH_2) and 13.8 (CH_3); HRMS (NSI^+): found, 314.1753. $C_{19}H_{24}NO_3$ (M+H) requires 314.1751.

N-Butyl-2-((3-methoxybenzyl)oxy)benzamide **21e**
Preparation as for **21b** using 3-methoxybenzyl bromide (2.02 g, 10.0 mmol), *N*-butylsalicylamide **25** (1.94 g, 10.0 mmol) and potassium carbonate (4.20 g, 30.4 mmol) at rt in DMF (10 mL) gave **21e** (2.78 g, 88%) as a pale yellow oil which solidified on standing and was used without further purification, mp 30–34 °C; ν_{max}/cm^{-1} 3387, 2955, 2868, 1643, 1595, 1541, 1472, 1267, 1155, 1036, 995, 789, 756, 698, 584 and 530; δ_H (500 MHz) 8.25 (1 H, dd, *J* 7.8, 1.8, ArH), 7.90 (1 H, br s, NH), 7.43 (1 H, ddd, *J* 8.5, 7.5, 2.0, ArH), 7.34 (1 H, t, *J* 7.8, ArH), 7.12–7.08 (1 H, m, ArH), 7.05–7.02 (2 H, m, ArH), 6.99–6.98 (1 H, m, ArH), 6.94–6.92 (1 H, m, ArH), 5.12 (2 H, s, OCH_2), 3.83 (3 H, s, OCH_3), 3.35 (2 H, td, *J* 7.0, 5.5, NCH_2), 1.38–1.32 (2 H, m, NCH_2CH_2), 1.21–1.13 (2 H, m, CH_2CH_3) and 0.81 (3 H, t, *J* 7.5, CH_2CH_3); δ_C (125 MHz) 164.9 (C=O), 159.9 (C–O), 156.7 (C–O), 137.0 (C), 132.5 (CH), 132.4 (CH), 130.0 (CH), 121.9 (C), 121.5 (CH), 120.3 (CH), 114.0 (CH), 113.8 (CH), 112.4 (CH), 71.2 (OCH_2), 55.2 (OCH_3), 39.4 (NCH_2), 31.2 (CH_2), 20.0 (CH_2) and 13.7 (CH_3); HRMS (NSI^+): found, 314.1750. $C_{19}H_{24}NO_3$ (M+H) requires 314.1751.

N-Butyl-2-((2-methoxybenzyl)oxy)benzamide **21f**

Preparation as for **21b** using 2-methoxybenzyl bromide (2.03 g, 10.1 mmol), *N*-butylsalicylamide **25** (1.93 g, 9.99 mmol) and potassium carbonate (4.17 g, 30.2 mmol) at rt in DMF (10 mL) gave **21f** (2.90 g, 93%) as a pale yellow oil which was used without further purification; ν_{max}/cm^{-1} 3389, 2957, 1651, 1599, 1537, 1298, 1250, 1030 and 758; δ_H (500 MHz) 8.22 (1 H, dd, *J* 7.8, 1.8, ArH), 7.98 (1 H, br s, NH), 7.43 (1 H, ddd, *J* 8.3, 7.3, 1.8, ArH), 7.39–7.36 (2 H, m, ArH), 7.10–7.06 (2 H, m, ArH), 7.00 (1 H, td, *J* 7.5, 1.0, ArH), 6.96 (1 H, d, *J* 8.5, ArH), 5.20 (2 H, s, OCH_2), 3.86 (3 H, s, OCH_3), 3.34 (2 H, td, *J* 6.8, 5.5, NCH_2), 1.37–1.31 (2 H, m, NCH_2CH_2), 1.21–1.13 (2 H, m, CH_2CH_3) and 0.80 (3 H, t, *J* 7.3, CH_2CH_3); δ_C (125 MHz) 165.2 (C=O), 157.5 (C–O), 157.0 (C–O), 132.4 (CH), 132.2 (CH), 130.2 (CH), 130.1 (CH), 123.8 (C), 122.0 (C), 121.3 (CH), 120.7 (CH), 112.6 (CH), 110.6 (CH), 66.7 (OCH_2), 55.4 (OCH_3), 39.4 (NCH_2), 31.3 (CH_2), 20.0 (CH_2) and 13.8 (CH_3); HRMS (NSI$^+$): found, 314.1745. $C_{19}H_{24}NO_3$ (M+H) requires 314.1751.

N-Butyl-2-((2-methoxynaphthalen-1-yl)methoxy)benzamide **21g**

Preparation as for **21b** using 1-(bromomethyl)-2-methoxynaphthalene (2.52 g, 10.0 mmol), *N*-butylsalicylamide **25** (1.93 g, 9.99 mmol) and potassium carbonate (4.17 g, 30.2 mmol) at rt in DMF (10 mL) gave **21g** (3.46 g, 95%) as a pale yellow solid which was used without further purification, mp 54–58 °C; ν_{max}/cm^{-1} 3385, 2930, 1653, 1597, 1533, 1466, 1252, 1221, 1094, 986, 802, 748 and 579; δ_H (300 MHz) 8.25 (1 H, dd, *J* 7.8, 2.1, ArH), 7.91 (1 H, d, *J* 9.0, ArH), 7.89 (1 H, d, *J* 8.4, ArH), 7.82 (1 H, d, *J* 7.8, ArH), 7.71 (1 H, br s, NH), 7.51–7.45 (2 H, m, ArH), 7.40–7.34 (1 H, m, ArH), 7.32–7.26 (2 H, m, ArH), 7.12–7.06 (1 H, m, ArH), 5.64 (2 H, s, OCH_2), 3.94 (3 H, s, OCH_3), 3.04–2.98 (2 H, m, NCH_2), 0.70–0.64 (4 H, m, $NCH_2CH_2CH_2$) and 0.50–0.44 (3 H, m, CH_2CH_3); δ_C (75 MHz) 164.8 (C=O), 157.2 (C–O), 155.5 (C–O), 133.2 (C), 132.4 (CH), 132.1 (CH), 131.3 (CH), 128.9 (C), 128.5 (CH), 127.4 (CH), 123.7 (CH), 122.6 (CH), 121.7 (C), 121.1 (CH), 115.4 (C), 112.8 (CH), 112.4 (CH), 61.5 (OCH_2), 56.4 (OCH_3), 39.0 (NCH_2), 30.3 (CH_2), 19.5 (CH_2) and 13.4 (CH_3); HRMS (NSI$^+$): found, 364.1908. $C_{23}H_{26}NO_3$ (M+H) requires 364.1907.

N-Butyl-2-((4-fluorobenzyl)oxy)benzamide **21h**

Preparation as for **21b** using 4-fluorobenzyl chloride (1.2 mL, 1.45 g, 10.0 mmol), *N*-butylsalicylamide **25** (1.93 g, 9.99 mmol) and potassium carbonate (4.15 g, 30.0 mmol) heated at 100 °C in DMF (10 mL) gave, after recrystallisation (EtOAc/hexane), **21h** (2.68 g, 89%) as colourless crystals, mp 109–112 °C; ν_{max}/cm^{-1} 3377, 2953, 1645, 1599, 1557, 1512, 1219, 1159, 1001, 951, 754, 596, 523 and 484; δ_H (400 MHz) 8.24 (1 H, dd, *J* 7.8, 1.8, ArH), 7.78 (1 H, br s, NH), 7.46–7.42 (3 H, m, ArH), 7.15–7.08 (3 H, m, ArH), 7.04 (1 H, dd, *J* 8.4, 0.8, ArH), 5.11 (2 H, s, OCH_2), 3.34 (2 H, td, *J* 6.8, 5.2, NCH_2), 1.36–1.29 (2 H, m, NCH_2CH_2), 1.19–1.10 (2 H, m, CH_2CH_3) and 0.81 (3 H, t, *J* 7.2, CH_3); δ_C (100 MHz) 164.9 (C=O), 162.9 (d, J_{CF} 246, CF), 156.6 (C–O), 132.5 (CH), 132.4 (CH), 131.4 (d, J_{CF} 3.2, C), 130.2 (d, J_{CF} 8.3, 2CH), 122.0 (C), 121.7 (CH), 115.8 (d, J_{CF} 21.5, 2CH), 112.3 (CH), 70.6 (OCH_2), 39.4 (NCH_2), 31.2 (CH_2), 20.0 (CH_2) and 13.7 (CH_3); δ_F (376 MHz) –112.6; HRMS (ESI$^+$): found, 324.1355. $C_{18}H_{20}FNaNO_2$ (M+Na) requires 324.1370.

N-Butyl-2-((3-fluorobenzyl)oxy)benzamide **21i**

Preparation as for **21b** using 3-fluorobenzyl chloride (1.2 mL, 1.43 g, 9.91 mmol), *N*-butylsalicylamide **25** (1.93 g, 9.99 mmol) and potassium carbonate (4.18 g, 30.2 mmol) heated at 100 °C in DMF (10 mL) gave, after recrystallisation (EtOAc/hexane), **21i** (2.70 g, 90%) as

colourless crystals, mp 97–99 °C; ν_{max}/cm^{-1} 3379, 2955, 2870, 1643, 1553, 1450, 1236, 1152, 1001, 897, 783, 752, 698 and 592; δ_H (400 MHz) 8.24 (1 H, dd, *J* 8.0, 2.0, ArH), 7.77 (1 H, br s, NH), 7.46–7.38 (2 H, m, ArH), 7.24–7.22 (1 H, m, ArH), 7.19–7.16 (1 H, m, ArH), 7.13–7.07 (2 H, m, ArH), 7.02 (1 H, dd, *J* 8.2, 0.6, ArH), 5.15 (2 H, s, OCH_2), 3.37 (2 H, td, *J* 7.0, 5.2, NCH_2), 1.42–1.34 (2 H, m, NCH_2CH_2), 1.24–1.15 (2 H, m, CH_2CH_3) and 0.83 (3 H, t, *J* 7.2, CH_3); δ_C (100 MHz) 164.9 (C=O), 163.0 (d, J_{CF} 246, CF), 156.4 (C–O), 138.0 (d, J_{CF} 7.2, C), 132.5 (CH), 132.4 (CH), 130.5 (d, J_{CF} 8.2, CH), 123.6 (d, J_{CF} 2.9, CH), 122.1 (C), 121.8 (CH), 115.7 (d, J_{CF} 20.9, CH), 114.9 (d, J_{CF} 21.8, CH), 112.3 (CH), 70.5 (d, J_{CF} 1.4, OCH_2), 39.4 (NCH_2), 31.3 (CH_2), 20.1 (CH_2) and 13.7 (CH_3); δ_F (376 MHz) –112.0; HRMS (ESI^+): found, 324.1363. $C_{18}H_{20}FNaNO_2$ (M+Na) requires 324.1370.

N-Butyl-2-((2-fluorobenzyl)oxy)benzamide **21j**

Preparation as for **21b** using 2-fluorobenzyl chloride (1.2 mL, 1.46 g, 10.1 mmol), *N*-butylsalicylamide **25** (1.94 g, 10.0 mmol) and potassium carbonate (4.18 g, 30.2 mmol) heated at 100 °C in DMF (10 mL) gave, after recrystallisation (EtOAc/hexane), **21j** (2.64 g, 87%) as colourless crystals, mp 61–63 °C; ν_{max}/cm^{-1} 3406, 2955, 1634, 1528, 1450, 1227, 997, 959, 756, 660 and 521; δ_H (400 MHz) 8.23 (1 H, dd, *J* 7.8, 1.8, ArH), 7.78 (1 H, br s, NH), 7.48–7.37 (3 H, m, ArH), 7.20 (1 H, td, *J* 7.6, 1.2, ArH), 7.17–7.12 (1 H, m, ArH), 7.12–7.06 (2 H, m, ArH), 5.22 (2 H, s, OCH_2), 3.36 (2 H, td, *J* 7.0, 5.6, NCH_2), 1.40–1.32 (2 H, m, NCH_2CH_2), 1.23–1.14 (2 H, m, CH_2CH_3) and 0.81 (3 H, t, *J* 7.4, CH_3); δ_C (100 MHz) 164.9 (C=O), 160.9 (d, J_{CF} 247, CF), 156.4 (C–O), 132.4 (CH), 132.3 (CH), 130.8 (d, J_{CF} 8.2, CH), 130.6 (d, J_{CF} 3.6, CH), 124.5 (d, J_{CF} 3.6, CH), 122.7 (d, J_{CF} 14.4, C), 122.2 (C), 121.7 (CH), 115.7 (d, J_{CF} 21.0, CH), 112.4 (CH), 65.1 (d, J_{CF} 3.8, OCH_2), 39.4 (NCH_2), 31.2 (CH_2), 20.0 (CH_2) and 13.7 (CH_3); δ_F (376 MHz) –117.9; HRMS (ESI^+): found, 324.1358. $C_{18}H_{20}FNNaO_2$ (M+Na) requires 324.1370.

N-Butyl-2-((2-(trifluoromethyl)benzyl)oxy)benzamide **21k**

Preparation as for **21b** using 2-(trifluoromethyl)benzyl chloride (1.5 mL, 2.01 g, 10.3 mmol), *N*-butylsalicylamide **25** (1.93 g, 9.99 mmol) and potassium carbonate (4.20 g, 30.4 mmol) heated at 100 °C in DMF (10 mL) gave, after recrystallisation (EtOAc/hexane), **21k** (3.00 g, 85%) as a colourless solid, mp 51–54 °C; ν_{max}/cm^{-1} 3321, 2968, 1630, 1530, 1315, 1238, 1167, 1103, 1034, 750 and 654; δ_H (500 MHz) 8.22 (1 H, dd, *J* 7.8, 1.8, ArH), 7.77 (1 H, d, *J* 8.0, ArH), 7.66 (1 H, br s, NH), 7.65 (1 H, d, *J* 7.5, ArH), 7.62 (1 H, t, *J* 7.5, ArH), 7.52 (1 H, t, *J* 7.5, ArH), 7.44–7.41 (1 H, m, ArH), 7.11 (1 H, t, *J* 7.5, ArH), 7.00 (1 H, d, *J* 8.0, ArH), 5.36 (2 H, s, OCH_2), 3.36 (2 H, td, *J* 6.8, 5.5, NCH_2), 1.38–1.32 (2 H, m, NCH_2CH_2), 1.19–1.12 (2 H, m, CH_2CH_3) and 0.80 (3 H, t, *J* 7.3, CH_3); δ_C (125 MHz) 165.0 (C=O), 156.2 (C–O), 133.9 (C), 132.5 (CH), 132.4 (2CH), 130.1 (CH), 128.8 (CH), 128.3 (q, J_{CF} 30.8, CCF_3), 126.3 (q, J_{CF} 5.5, CH), 124.1 (q, J_{CF} 272, CF_3), 122.3 (C), 121.8 (CH), 112.3 (CH), 67.4 (q, J_{CF} 2.5, OCH_2), 39.4 (NCH_2), 31.2 (CH_2), 20.0 (CH_2) and 13.7 (CH_3); δ_F (470 MHz) –59.7; HRMS (NSI^+): found, 352.1518. $C_{19}H_{21}F_3NO_2$ (M+H) requires 352.1519.

N-Butyl-2-((perfluorophenyl)methoxy)benzamide **21l**

Preparation as for **21b** using 2,3,4,5,6-pentafluorobenzyl bromide (1.5 mL, 2.59 g, 9.93 mmol), *N*-butylsalicylamide **25** (1.94 g, 10.0 mmol) and potassium carbonate (4.16 g, 30.1 mmol) heated at 100 °C in DMF (10 mL) gave, after recrystallisation (EtOAc/hexane), **21l** (2.62 g, 71%) as pale orange crystals, mp 86–89 °C; ν_{max}/cm^{-1} 3387, 2961, 1638, 1506, 1381, 1290, 1223, 1061, 1009, 939, 752 and 687; δ_H (400 MHz) 8.18 (1 H, dd, *J* 7.8, 1.8, ArH), 7.49–7.45 (1 H, m, ArH), 7.45 (1

H, br s, NH), 7.17–7.13 (1 H, m, ArH), 7.10 (1 H, d, *J* 8.0, ArH), 5.30 (2 H, s, OCH_2), 3.40 (2 H, td, *J* 7.0, 5.6, NCH_2), 1.48–1.41 (2H, m, NCH_2CH_2), 1.31–1.22 (2H, m, CH_2CH_3) and 0.89 (3H, t, *J* 7.2, CH_3); δ_C (125 MHz) 164.6 (C=O), 155.3 (C–O), 145.6 (dm, J_{CF} 249, 2CF), 142.0 (dm, J_{CF} 256, CF), 137.6 (dm, J_{CF} 253, 2CF), 132.6 (CH), 132.5 (CH), 122.9 (C), 122.6 (CH), 112.6 (CH), 109.2 (td, J_{CF} 17.4, 3.6, Ar C), 58.2 (OCH_2), 39.5 (NCH_2), 31.3 (CH_2), 20.0 (CH_2) and 13.6 (CH_3); δ_F (376 MHz) –141.8 to –141.9 (m, 2CF), –151.0 to –151.1 (m, CF) and –160.4 to –160.5 (m, 2CF); HRMS (NSI^+): found, 374.1172. $C_{18}H_{17}F_5NO_2$ (M+H) requires 374.1174.

N-Butyl-2-((4-nitrobenzyl)oxy)benzamide **21m**

Preparation as for **21b** using 4-nitrobenzyl bromide (2.19 g, 10.1 mmol), *N*-butylsalicylamide **25** (1.94 g, 10.0 mmol) and potassium carbonate (4.17 g, 30.2 mmol) at rt in DMF (10 mL) gave, after recrystallisation (EtOAc/hexane), **21m** (2.99 g, 91%) as colourless crystals, mp 122–125 °C; ν_{max}/cm^{-1} 3385, 2953, 2872, 1641, 1516, 1350, 1234, 1007, 856, 831, 756, 702, 588, 532 and 500; δ_H (500 MHz) 8.30 (2 H, d, *J* 8.5, ArH), 8.21 (1 H, dd, *J* 7.8, 1.8, ArH), 7.64 (2 H, d, *J* 8.5, ArH), 7.57 (1 H, br s, NH), 7.45–7.41 (1H, m, ArH), 7.15–7.11 (1 H, m, ArH), 6.99 (1 H, d, *J* 8.5, ArH), 5.29 (2 H, s, OCH_2), 3.40 (2 H, td, *J* 7.0, 5.5, NCH_2), 1.44–1.39 (2 H, m, NCH_2CH_2), 1.26–1.19 (2 H, m, CH_2CH_3) and 0.83 (3 H, t, *J* 7.3, CH_3); δ_C (125 MHz) 164.9 (C=O), 155.9 (C–O), 148.0 (CNO_2), 142.8 (C), 132.5 (CH), 132.4 (CH), 128.4 (2CH), 124.1 (2CH), 122.5 (C), 122.2 (CH), 112.4 (CH), 69.9 (OCH_2), 39.5 (NCH_2), 31.4 (CH_2), 20.1 (CH_2) and 13.7 (CH_3); HRMS (NSI^+): found, 329.1494. $C_{18}H_{21}N_2O_4$ (M+H) requires 329.1496.

N-Butyl-2-((3-nitrobenzyl)oxy)benzamide **21n**

Preparation as for **21b** using 3-nitrobenzyl bromide (2.18 g, 10.1 mmol), *N*-butylsalicylamide **25** (1.94 g, 10.0 mmol) and potassium carbonate (4.20 g, 30.4 mmol) at rt in DMF (10 mL) gave, after recrystallisation (EtOAc/hexane), **21n** (3.01 g, 91%) as colourless crystals, mp 130–133 °C; ν_{max}/cm^{-1} 3381, 2953, 2870, 1641, 1522, 1472, 1346, 1227, 1099, 1009, 924, 810, 760, 735, 694 and 673; δ_H (500 MHz) 8.36–8.35 (1 H, m, ArH), 8.29–8.26 (1 H, m, ArH), 8.21 (1 H, dd, *J* 8.0, 2.0, ArH), 7.81 (1 H, d, *J* 7.5, ArH), 7.64 (1 H, t, *J* 7.8, ArH), 7.58 (1 H, br s, NH), 7.47–7.43 (1 H, m, ArH), 7.15–7.12 (1 H, m, ArH), 7.03 (1 H, d, *J* 8.0, ArH), 5.28 (2 H, s, OCH_2), 3.39 (2 H, td, *J* 7.0, 5.5, NCH_2), 1.41–1.35 (2 H, m, NCH_2CH_2), 1.23–1.15 (2 H, m, CH_2CH_3) and 0.81 (3 H, t, *J* 7.3, CH_3); δ_C (125 MHz) 164.9 (C=O), 156.0 (C–O), 148.5 (CNO_2), 137.7 (C), 133.7 (CH), 132.54 (CH), 132.45 (CH), 130.0 (CH), 123.7 (CH), 122.7 (CH), 122.5 (C), 122.2 (CH), 112.5 (CH), 70.0 (OCH_2), 39.4 (NCH_2), 31.4 (CH_2), 20.1 (CH_2) and 13.7 (CH_3); HRMS (NSI^+): found, 329.1495. $C_{18}H_{21}N_2O_4$ (M+H) requires 329.1496.

N-Butyl-2-((2-nitrobenzyl)oxy)benzamide **21o**

Preparation as for **21b** using 2-nitrobenzyl bromide (2.19 g, 10.1 mmol), *N*-butylsalicylamide **25** (1.94 g, 10.0 mmol) and potassium carbonate (4.17 g, 30.2 mmol) at rt in DMF (10 mL) gave, after recrystallisation (EtOAc/hexane), **21o** (2.76 g, 84%) as a colourless light-sensitive solid, mp 98–100 °C; ν_{max}/cm^{-1} 3281, 2930, 1632, 1514, 1337, 1304, 1234, 1105, 1026, 866, 748, 725 and 671; δ_H (500 MHz) 8.20 (1 H, d, *J* 8.5, ArH), 8.17 (1 H, dd, *J* 7.8, 1.8, ArH), 7.74 (1 H, d, *J* 7.5, ArH), 7.71–7.68 (1 H, m, ArH), 7.58–7.55 (1 H, m, ArH), 7.55 (1 H, br s, NH), 7.42–7.38 (1 H, m, ArH), 7.13–7.10 (1 H, m, ArH), 6.95 (1 H, d, *J* 8.0, ArH), 5.63 (2 H, s, OCH_2), 3.46–3.42 (2 H, m, NCH_2), 1.53–1.47 (2 H, m, NCH_2CH_2), 1.33–1.26 (2 H, m, CH_2CH_3) and 0.88 (3 H, t, *J* 7.5, CH_3); δ_C (125 MHz) 165.1 C=O), 155.7 (C–O), 147.3 (CNO_2), 134.1 (CH), 132.4 (CH), 132.2 (CH), 132.1 (C), 129.2 (CH), 129.1 (CH), 125.3 (CH), 122.8 (C), 122.0 (CH), 112.7 (CH), 67.9 (OCH_2),

39.5 (NCH_2), 31.5 (CH_2), 20.1 (CH_2) and 13.7 (CH_3); HRMS (NSI^+): found, 329.1486. $C_{18}H_{21}N_2O_4$ (M+H) requires 329.1496.

N-Butyl-2-(pyridin-4-ylmethoxy)benzamide **21p**

Preparation as for **21b** using 4-(chloromethyl)pyridine hydrochloride[19] (1.68 g, 10.2 mmol), *N*-butylsalicylamide **25** (1.95 g, 10.1 mmol) and potassium carbonate (4.20 g, 30.4 mmol) heated at 100 °C in DMF (10 mL) gave, after recrystallisation (EtOAc/hexane), **21p** (2.22 g, 77%) as yellow needles, mp 77–80 °C; ν_{max}/cm^{-1} 3383, 2874, 1643, 1595, 1557, 1476, 1377, 1308, 1225, 1101, 1011, 820, 760, 664 and 511; δ_H (400 MHz) 8.68 (2 H, d, *J* 7.5, ArH), 8.22 (1 H, dd, *J* 7.8, 1.8, ArH), 7.63 (1 H, br s, NH), 7.45–7.40 (1 H, m, ArH), 7.37 (2 H, d, *J* 7.5, ArH), 7.15–7.11 (1 H, m, ArH), 6.97 (1 H, dd, *J* 8.4, 0.8, ArH), 5.20 (2 H, s, OCH_2), 3.42 (2 H, td, *J* 7.0, 5.6, NCH_2), 1.49–1.42 (2 H, m, NCH_2CH_2), 1.31–1.21 (2 H, m, CH_2CH_3) and 0.86 (3 H, t, *J* 7.4, CH_3); δ_C (125 MHz) 164.9 (C=O), 156.0 (C–O), 150.4 (2CH), 144.5 (C), 132.5 (CH), 132.4 (CH), 122.4 (C), 122.1 (CH), 121.9 (2CH), 112.4 (CH), 69.4 (OCH_2), 39.5 (NCH_2), 31.4 (CH_2), 20.1 (CH_2) and 13.7 (CH_3); HRMS (NSI^+): found, 285.1594. $C_{17}H_{21}N_2O_2$ (M+H) requires 285.1598.

N-Butyl-2-(thiophen-2-ylmethoxy)benzamide **21q**

Preparation as for **21b** using 2-(bromomethyl)thiophene (1.80 g, 10.2 mmol), *N*-butylsalicylamide **25** (1.93 g, 9.99 mmol) and potassium carbonate (4.15 g, 30.0 mmol) at rt in DMF (10 mL) gave, after purification by column chromatography (SiO_2, gradient elution, Et_2O/hexane 3:2 to Et_2O), **21q** (1.40 g, 48%) as a yellow solid, mp 66–68 °C; ν_{max}/cm^{-1} 3375, 2924, 1639, 1597, 1531, 1300, 1231, 988, 752, 712 and 596; δ_H (400 MHz) 8.25 (1 H, dd, *J* 7.6, 2.0, ArH), 7.86 (1 H, br s, NH), 7.47–7.43 (1 H, m, ArH), 7.40 (1 H, dd, *J* 5.2, 1.2, ArH), 7.18–7.16 (1 H, m, ArH), 7.14–7.10 (1 H, m, ArH), 7.07–7.05 (2 H, m, ArH), 5.32 (2 H, s, OCH_2), 3.37 (2 H, td, *J* 6.8, 5.6, NCH_2), 1.43–1.36 (2 H, m, NCH_2CH_2), 1.26–1.16 (2 H, m, CH_2CH_3) and 0.84 (3 H, t, *J* 7.4, CH_3); δ_C (125 MHz) 164.8 (C=O), 156.2 (C–O), 137.4 (C), 132.5 (2CH), 128.0 (CH), 127.11 (CH), 127.07 (CH), 122.1 (C), 121.8 (CH), 112.4 (CH), 65.7 (OCH_2), 39.5 (NCH_2), 31.2 (CH_2), 20.0 (CH_2) and 13.8 (CH_3); HRMS (NSI^+): found, 312.1026. $C_{16}H_{19}NaNO_2S$ (M+Na) requires 312.1029.

2-(Diphenylmethoxy)-*N*-butylbenzamide **21r**

Preparation as for **21b** using benzhydryl bromide (2.48 g, 10.0 mmol), *N*-butylsalicylamide **25** (1.94 g, 10.0 mmol) and potassium carbonate (4.19 g, 30.3 mmol) at rt in DMF (10 mL) gave, after purification by column chromatography (SiO_2, Et_2O/hexane 7:3), at R_f 0.75, **21r** (3.08 g, 85%) as a colourless oil; ν_{max}/cm^{-1} 3395, 2957, 1651, 1599, 1531, 1477, 1221, 1001, 756 and 704; δ_H (500 MHz) 8.24 (1 H, dd, *J* 8.0, 1.5, ArH), 7.92 (1 H, br s, NH), 7.40–7.31 (10 H, m, Ph), 7.27–7.23 (1 H, m, ArH), 7.03 (1 H, t, *J* 7.8, ArH), 6.84 (1 H, d, *J* 8.5, ArH), 6.34 (1 H, s, $CHPh_2$), 3.31 (2 H, td, *J* 7.0, 5.0, NCH_2), 1.26–1.21 (2 H, m, NCH_2CH_2), 1.17–1.10 (2 H, m, CH_2CH_3) and 0.79 (3 H, t, *J* 7.8, CH_3); δ_C (100 MHz) 165.0 (C=O), 156.1 (C–O), 139.7 (2C), 132.29 (CH), 132.27 (CH), 128.9 (4CH), 128.4 (2CH), 126.9 (4CH), 122.2 (C), 121.6 (CH), 114.3 (CH), 83.5 ($CHPh_2$), 39.5 (NCH_2), 31.1 (CH_2), 20.0 (CH_2) and 13.8 (CH_3); HRMS (NSI^+): found, 382.1769. $C_{24}H_{25}NaNO_2$ (M+Na) requires 382.1778.

N-Butyl-2-(1-phenylethoxy)benzamide **21s**

Preparation as for **21b** using (1-bromoethyl)benzene (1.85 g, 10.0 mmol), *N*-butylsalicylamide **25** (1.94 g, 10.0 mmol) and potassium carbonate (4.17 g, 30.2 mmol) heated at 100 °C in DMF (10 mL) gave **21s** (2.76 g, 93%) as a pale yellow oil which was used without further purification;

ν_{max}/cm^{-1} 3406, 2930, 1655, 1533, 1479, 1294, 1229, 1067, 758 and 702; δ_H (400 MHz) 8.21 (1 H, dd, *J* 7.8, 1.8, ArH), 8.13 (1 H, br s, NH), 7.39–7.28 (5 H, m, Ph), 7.25 (1H, ddd, *J* 8.4, 7.2, 2.0, ArH), 7.00 (1 H, ddd, *J* 8.0, 7.2, 1.2, ArH), 6.82–6.79 (1 H, m, ArH), 5.48 (1 H, q, *J* 6.4, C*H*CH$_3$), 3.49 (2 H, td, *J* 7.0, 5.6, NCH$_2$), 1.74 (3 H, d, *J* 6.4, CHC*H*$_3$), 1.62–1.55 (2 H, m, NCH$_2$C*H*$_2$), 1.47–1.38 (2 H, m, C*H*$_2$CH$_3$) and 0.95 (3 H, t, *J* 7.2, CH$_2$C*H*$_3$); δ_C (125 MHz) 165.3 (C=O), 155.8 (C–O), 141.6 (C), 132.24 (CH), 132.22 (CH), 128.9 (2CH), 128.1 (CH), 125.4 (2CH), 122.3 (C), 121.3 (CH), 114.1 (CH), 77.5 (*C*HCH$_3$), 39.5 (NCH$_2$), 31.5 (CH$_2$), 24.1 (CH*C*H$_3$), 20.3 (CH$_2$) and 13.8 (CH$_2$*C*H$_3$); HRMS (ESI$^+$): found, 320.1616. $C_{19}H_{23}NaNO_2$ (M+Na) requires 320.1621.

2-(Allyloxy)-*N*-butylbenzamide **21t**

A solution of 2-(allyloxy)benzoyl chloride[20] (4.97 g, 25.3 mmol) in toluene (30 mL) was added dropwise to a stirred 0 °C solution of *n*-butylamine (8.0 mL, 5.92 g, 80.9 mmol) in toluene (30 mL). Once the addition was complete, the reaction mixture was allowed to warm to rt for 1 h before being poured into water and washed with 2 M NaOH and brine. The organic layer was dried and evaporated to afford **21t** (4.93 g, 84%) as a pale yellow oil which was used without further purification; ν_{max}/cm^{-1} 3407, 2930, 1653, 1601, 1535, 1379, 1298, 1228, 1163, 1104, 995, 935, 857 and 756; δ_H (500 MHz) 8.22 (1 H, dd, *J* 7.8, 1.8, ArH), 7.95 (1 H, br s, NH), 7.42–7.39 (1 H, m, ArH), 7.08 (1 H, t, *J* 7.5, ArH), 6.95 (1 H, d, *J* 8.5, ArH), 6.14–6.06 (1 H, m, C*H*=CH$_2$), 5.46 (1 H, dm, *J* 17.0, CH=C*H*$_2$), 5.39 (1 H, dm, *J* 10.5, CH=C*H*$_2$), 4.66 (2 H, dd, *J* 5.5, 1.0, OCH$_2$), 3.49–3.45 (2 H, m, NCH$_2$), 1.61–1.55 (2 H, m, NCH$_2$C*H*$_2$), 1.46–1.38 (2 H, m, C*H*$_2$CH$_3$) and 0.95 (3 H, t, *J* 7.5, CH$_3$); δ_C (125 MHz) 165.1 (C=O), 156.4 (C–O), 132.4 (CH), 132.3 (CH), 132.0 (CH), 122.0 (C), 121.5 (CH), 119.3 (=CH$_2$), 112.6 (CH), 69.9 (OCH$_2$), 39.4 (NCH$_2$), 31.5 (CH$_2$), 20.2 (CH$_2$) and 13.7 (CH$_3$); HRMS (ESI$^+$): found, 256.1303. $C_{14}H_{19}NaNO_2$ (M+Na) requires 256.1308.

N-Butyl-2-((3-methylbut-2-en-1-yl)oxy)benzamide **21u**

Preparation as for **21b** using prenyl bromide (1.52 g, 10.2 mmol), *N*-butylsalicylamide **25** (1.94 g, 10.0 mmol) and potassium carbonate (4.15 g, 30.0 mmol) heated at 100 °C in DMF (10 mL) gave **21u** (2.58 g, 98%) as a pale yellow oil which was used without further purification; ν_{max}/cm^{-1} 3393, 2932, 1655, 1599, 1537, 1483, 1298, 1227, 989 and 756; δ_H (400 MHz) 8.23 (1 H, dd, *J* 8.0, 2.0, ArH), 8.10 (1 H, br s, NH), 7.43–7.39 (1 H, m, ArH), 7.08–7.04 (1 H, m, ArH), 6.97 (1 H, d, *J* 8.4, ArH), 5.56–5.51 (1 H, m, C*H*=CMe$_2$), 4.62 (2 H, d, *J* 7.2, OCH$_2$), 3.46 (2 H, td, *J* 6.8, 5.2, NCH$_2$), 1.83 (3 H, s, CMe$_2$), 1.77 (3 H, s, CMe$_2$), 1.60–1.53 (2 H, m, NCH$_2$C*H*$_2$), 1.48–1.37 (2 H, m, C*H*$_2$CH$_3$) and 0.96 (3 H, t, *J* 7.2, CH$_2$C*H*$_3$); δ_C (100 MHz) 165.1 (C=O), 156.9 (C–O), 140.2 (C), 132.4 (CH), 132.2 (CH), 121.8 (C), 121.2 (CH), 118.4 (CH), 112.5 (CH), 65.6 (OCH$_2$), 39.3 (NCH$_2$), 31.4 (CH$_2$), 25.8 (CH$_3$), 20.2 (CH$_2$), 18.2 (CH$_3$) and 13.8 (CH$_3$); HRMS (ESI$^+$): found, 284.1612. $C_{16}H_{23}NaNO_2$ (M+Na) requires 284.1621.

N-Butyl-2-(2,2,2-trifluoroethoxy)benzamide **21v**

A mixture of ethyl 2-(2,2,2-trifluoroethoxy)benzoate (2.52 g, 10.2 mmol) and *n*-butylamine (30 mL, 22.20 g, 0.304 mol) was heated at reflux for 3 d. After cooling to rt, the reaction mixture was concentrated *in vacuo*. The residue was dissolved in CH_2Cl_2 (100 mL) and washed with 2 M HCl (50 mL) and sat. aq. Na_2CO_3 (50 mL) before being dried and evaporated to give **21v** (2.38 g, 85%) as tan-coloured crystals which were used without further purification, mp 63–65 °C; ν_{max}/cm^{-1} 3424, 2932, 1639, 1541, 1275, 1229, 1155, 1109, 1061, 966, 866, 754 and 691; δ_H (400 MHz) 8.13 (1 H, dd, *J* 7.8, 1.8, ArH), 7.49 (1 H, br s, NH), 7.43–7.39 (1 H, m, ArH), 7.12 (1 H, t, *J* 7.6, ArH),

6.90 (1 H, d, *J* 8.4, ArH), 4.50 (2 H, q, J_{HF} 8.0, OCH_2), 3.44 (2 H, td, *J* 7.2, 5.2, NCH_2), 1.61–1.54 (2 H, m, NCH_2CH_2), 1.45–1.36 (2 H, m, CH_2CH_3) and 0.95 (3 H, t, *J* 7.2, CH_3); δ_C (125 MHz) 164.3 (C=O), 154.4 (C–O), 132.3 (CH), 132.1 (CH), 122.9 (q, J_{CF} 276, CF_3), 122.6 (CH), 122.3 (C), 112.1 (CH), 65.8 (q, J_{CF} 35.6, OCH_2), 39.4 (NCH_2), 30.9 (CH_2), 19.7 (CH_2) and 13.3 (CH_3); δ_F (376 MHz) –73.9; HRMS (NSI^+): found, 276.1205. $C_{13}H_{17}F_3NO_2$ (M+H) requires 276.1206.

N-Butyl-2-((3-phenylprop-2-yn-1-yl)oxy)benzamide **21w**

Preparation as for **21b** using (3-bromoprop-1-yn-1-yl)benzene[21] (1.95 g, 10.0 mmol), *N*-butylsalicylamide **25** (1.93 g, 9.99 mmol) and potassium carbonate (4.15 g, 30.0 mmol) in DMF (10 mL) gave, after filtration through a silica plug (Et_2O), **21w** (2.60 g, 85%) as a pale yellow solid, mp 52–56 °C; ν_{max}/cm^{-1} 3366, 2930, 1636, 1530, 1483, 1379, 1227, 1015, 752 and 691; δ_H (500 MHz) 8.23 (1 H, dd, *J* 7.8, 1.8, ArH), 7.90 (1 H, br s, NH), 7.45–7.41 (3 H, m, ArH and Ph), 7.36–7.26 (3 H, m, Ph), 7.10 (1 H, t, *J* 7.8, ArH), 7.07 (1 H, d, *J* 8.5, ArH), 5.01 (2 H, s, OCH_2), 3.47 (2 H, td, *J* 7.0, 5.5, NCH_2), 1.62–1.56 (2 H, m, NCH_2CH_2), 1.45–1.37 (2 H, m, CH_2CH_3) and 0.88 (3 H, t, *J* 7.5, CH_3); δ_C (125 MHz) 164.8 (C=O), 155.6 (C–O), 132.3 (CH), 132.2 (CH), 131.6 (2CH), 128.9 (CH), 128.3 (2CH), 122.3 (C), 121.9 (CH), 121.6 (C), 112.8 (CH), 88.1 (C≡C), 82.4 (C≡C), 57.8 (OCH_2), 39.5 (NCH_2), 31.4 (CH_2), 20.1 (CH_2) and 13.7 (CH_3); HRMS (NSI^+): found, 308.1646. $C_{20}H_{22}NO_2$ (M+H) requires 308.1645.

N-Butyl-2-(hydroxy(*p*-tolyl)methyl)benzamide **22b** and 3-(*p*-Tolyl)phthalide **23b**

Under a nitrogen atmosphere, *n*-butyllithium (2.5 M in hexane, 2.6 mL, 6.50 mmol) was added dropwise to a stirred solution of *N*-butyl-2-((4-methylbenzyl)oxy)benzamide **21b** (0.5951 g, 2.00 mmol) in dry THF (20 mL). After stirring at rt for 2 h, the reaction mixture was quenched by addition of sat. aq. NH_4Cl and extracted with Et_2O (× 3). The combined organic layers were washed with 2 M NaOH and water before being dried and evaporated to give **22b** as an orange oil; ν_{max}/cm^{-1} 3287, 1932, 1628, 1512, 1458, 1250, 1026 and 741; δ_H (400 MHz) 7.38–7.33 (2 H, m, ArH), 7.26–7.22 (2 H, m, ArH), 7.12 (2 H, d, *J* 8.2, ArH), 7.04 (2 H, d, *J* 8.2, ArH), 6.48 (1 H, t, *J* 5.6, NH), 5.75 (2 H, br s, C*H*O*H*), 3.23–3.15 (1 H, m, NCH_2), 3.10–3.02 (1 H, m, NCH_2), 2.29 (3 H, s, CH_3), 1.29–1.13 (4 H, m, $NCH_2CH_2CH_2$) and 0.85 (3 H, t, *J* 7.2, CH_2CH_3); δ_C (100 MHz) 170.8 (C=O), 143.2 (C), 139.7 (C), 136.1 (C), 135.8 (C), 130.4 (CH), 129.7 (CH), 128.4 (2CH), 127.7 (CH), 127.5 (CH), 126.1 (2CH), 74.7 (CHOH), 39.7 (NCH_2), 31.1 (CH_2), 20.9 (CH_3), 19.9 (CH_2) and 13.6 (CH_3); HRMS (ESI^+): found, 320.1612. $C_{19}H_{23}NaNO_2$ (M+Na) requires 320.1621.

On standing at rt in EtOAc solution for 2–3 months, an intramolecular cyclisation occurred to give, after purification by column chromatography (SiO_2, Et_2O/hexane 1:4), at R_f 0.40, **23b** (0.3467 g, 77%) as colourless crystals, mp 125–128 °C; (lit.[22] 129 °C); δ_H (400 MHz) 7.93 (1 H, d, *J* 7.6, ArH), 7.62 (1 H, td, *J* 7.4, 1.2, ArH), 7.55–7.50 (1 H, m, ArH), 7.31 (1 H, dd, *J* 7.6, 0.8, ArH), 7.18–7.12 (4 H, m, ArH), 6.36 (1 H, s, CHAr) and 2.33 (3 H, s, CH_3). The ^{1}H NMR spectral data was in accordance with that previously reported.[23]

N-Butyl-2-(hydroxy(*o*-tolyl)methyl)benzamide **22c** and 3-(*o*-Tolyl)phthalide **23c**

Reaction as described for **22b** using *n*-butyllithium (2.6 mL, 6.50 mmol) and *N*-butyl-2-((2-methylbenzyl)oxy)benzamide **21c** (0.5954 g, 2.00 mmol) in dry THF (20 mL) gave **22c** as an orange oil; ν_{max}/cm^{-1} 3325, 1955, 1628, 1543, 1458, 1312, 1018, 756 and 664; δ_H (400 MHz) 7.68 (1 H, d, *J* 7.2, ArH), 7.45–7.40 (1 H, m, ArH), 7.28–7.23 (3 H, m, ArH), 7.19 (1 H, td, *J* 7.2, 1.2, ArH), 7.08 (1 H, d, *J* 7.6, ArH), 6.95–6.91 (1 H, m, ArH), 6.71 (1 H, t, *J* 5.4, NH), 6.03 (1 H, s, C***H***OH), 5.27 (1 H, br s, OH), 3.41–3.32 (2 H, m, NCH_2), 1.93 (3 H, s, CH_3), 1.56–1.47 (2 H, m,

NCH_2CH_2), 1.40–1.31 (2 H, m, CH_2CH_3) and 0.93 (3 H, t, *J* 7.2, CH_2CH_3); δ_C (100 MHz) 170.5 (C=O), 141.8 (C), 139.7 (C), 136.2 (C), 134.6 (C), 130.5 (CH), 130.0 (CH), 128.1 (CH), 127.5 (CH), 127.3 (CH), 127.0 (CH), 126.3 (CH), 125.7 (CH), 70.5 (CHOH), 39.7 (NCH_2), 31.4 (CH_2), 20.0 (CH_2), 19.2 (CH_3) and 13.6 (CH_3); HRMS (ESI^+): found, 320.1613. $C_{19}H_{23}NaNO_2$ (M+Na) requires 320.1621.

On standing at rt in EtOAc solution for 2–3 months, an intramolecular cyclisation occurred to give, after purification by column chromatography (SiO_2, Et_2O/hexane 1:4), at R_f 0.40, **23c** (0.2727 g, 61%) as colourless crystals, mp 105–108 °C; (lit.[24] 113 °C); δ_H (400 MHz) 7.94 (1 H, d, *J* 7.6, ArH), 7.65 (1 H, td, *J* 7.6, 1.2, ArH), 7.55 (1 H, t, *J* 7.4, ArH), 7.33 (1 H, dd, *J* 7.6, 0.8, ArH), 7.27–7.22 (2 H, m, ArH), 7.12–7.08 (1 H, m, ArH), 6.90 (1 H, d, *J* 7.6, ArH), 6.66 (1 H, s, CHAr) and 2.47 (3 H, s, CH_3). The ^{1}H NMR spectral data was in accordance with that previously reported.[23]

N-Butyl-2-(hydroxy(4-methoxyphenyl)methyl)benzamide **22d** and 3-(4-Methoxyphenyl)phthalide **23d**

Reaction as described for **22b** using *n*-butyllithium (2.6 mL, 6.50 mmol) and *N*-butyl-2-((4-methoxybenzyl)oxy)benzamide **21d** (0.6268 g, 2.00 mmol) in dry THF (20 mL) gave **22d** as an orange oil; ν_{max}/cm^{-1} 3287, 2955, 1628, 1512, 1458, 1304, 1242, 1173 and 1026; δ_H (400 MHz) 7.40–7.35 (2 H, m, ArH), 7.29–7.25 (2 H, m, ArH), 7.17 (2 H, d, *J* 8.8, ArH), 6.79 (2 H, d, *J* 8.8, ArH), 6.37 (1 H, t, *J* 5.6, NH), 5.78 (1 H, s, C*H*OH), 5.67 (1 H, br s, OH), 3.75 (3 H, s, OCH_3), 3.26–3.18 (1 H, m, NCH_2), 3.15–3.07 (1 H, m, NCH_2), 1.33–1.16 (4 H, m, $NCH_2CH_2CH_2$) and 0.86 (3 H, t, *J* 7.2, CH_2CH_3); δ_C (100 MHz) 170.8 (C=O), 158.3 (C–O), 143.3 (C), 135.8 (C), 135.0 (C), 130.5 (CH), 129.6 (CH), 127.7 (CH), 127.6 (CH), 127.4 (2CH), 113.1 (2CH), 74.4 (CHOH), 55.1 (OCH_3), 39.7 (NCH_2), 31.2 (CH_2), 19.9 (CH_2) and 13.6 (CH_3); HRMS (ESI^+): found, 336.1561. $C_{19}H_{23}NaNO_3$ (M+Na) requires 336.1570.

On standing at rt in EtOAc solution for 2–3 months, an intramolecular cyclisation occurred to give, after purification by column chromatography (SiO_2, Et_2O/hexane 2:3), at R_f 0.45, **23d** (0.3779 g, 69%) as orange crystals, mp 114–117 °C; (lit.[25] 116–117 °C); δ_H (400 MHz) 7.94 (1 H, d, *J* 7.6, ArH), 7.65 (1 H, td, *J* 7.6, 1.2, ArH), 7.57–7.52 (1 H, m, ArH), 7.31 (1 H, dd, *J* 7.6, 0.8, ArH), 7.16 (2 H, d, *J* 8.8, ArH), 6.88 (2 H, d, *J* 8.8, ArH), 6.36 (1 H, s, CHAr) and 3.79 (3 H, s, CH_3). The ^{1}H NMR spectral data was in accordance with that previously reported.[15]

N-Butyl-2-(hydroxy(3-methoxyphenyl)methyl)benzamide **22e** and 3-(3-Methoxyphenyl)phthalide **23e**

Reaction as described for **22b** using *n*-butyllithium (2.6 mL, 6.50 mmol) and *N*-butyl-2-((3-methoxybenzyl)oxy)benzamide **21e** (0.6272 g, 2.00 mmol) in dry THF (20 mL) gave **22e** as a yellow oil; ν_{max}/cm^{-1} 3283, 2957, 1630, 1599, 1541, 1487, 1456, 1435, 1260, 1152, 1042 and 737; δ_H (500 MHz) 7.41–7.38 (2 H, m, ArH), 7.31–7.28 (2 H, m, ArH), 7.16 (1 H, t, *J* 8.0, ArH), 6.93 (1 H, s, ArH), 6.79 (1 H, d, *J* 7.5, ArH), 6.75 (1 H, dd, *J* 8.3, 1.8, ArH), 6.11 (1 H, t, *J* 5.8, NH), 5.80 (1 H, s, C*H*OH), 5.74 (1 H, br s, OH), 3.76 (3 H, s, OCH_3), 3.28–3.21 (1 H, m, NCH_2), 3.16–3.10 (1 H, m, NCH_2), 1.33–1.27 (2 H, m, NCH_2CH_2), 1.25–1.18 (2 H, m, CH_2CH_3) and 0.87 (3 H, t, *J* 7.3, CH_2CH_3); δ_C (125 MHz) 170.9 (C=O), 159.3 (C–O), 144.6 (C), 143.2 (C), 135.8 (C), 130.7 (CH), 130.0 (CH), 128.7 (CH), 127.8 (CH), 127.6 (CH), 118.8 (CH), 112.3 (CH), 111.9 (CH), 74.9 (CHOH), 55.1 (OCH_3), 39.8 (NCH_2), 31.2 (CH_2), 19.9 (CH_2) and 13.7 (CH_3); HRMS (ESI^+): found, 336.1562. $C_{19}H_{23}NaNO_3$ (M+Na) requires 336.1570.

On standing at rt in EtOAc solution for 2–3 months, an intramolecular cyclisation occurred

to give, after purification by column chromatography (SiO_2, Et_2O/hexane 2:3), at R_f 0.45, **23e** (0.3299 g, 69%) as colourless crystals, mp 110–112 °C; (lit.[26] 112–114 °C); δ_H (400 MHz) 7.93 (1 H, d, *J* 7.6, ArH), 7.63 (1 H, td, *J* 7.6, 0.8, ArH), 7.53 (1 H, t, *J* 7.6, ArH), 7.34 (1 H, dd, *J* 7.6, 0.8, ArH), 7.28 (1 H, t, *J* 8.0, ArH), 6.90–6.85 (2 H, m, ArH), 6.79 (1 H, t, *J* 2.0, ArH), 6.36 (1 H, s, CHAr) and 3.75 (3 H, s, OCH_3). The ^{1}H NMR spectral data was in accordance with that previously reported.[27]

N-Butyl-2-(hydroxy(2-methoxyphenyl)methyl)benzamide **22f** and 3-(2-Methoxyphenyl)phthalide **23f**

Reaction as described for **22b** using *n*-butyllithium (2.6 mL, 6.50 mmol) and *N*-butyl-2-((2-methoxybenzyl)oxy)benzamide **21f** (0.6263 g, 2.00 mmol) in dry THF (20 mL) gave **22f** as an orange oil; ν_{max}/cm^{-1} 3308, 2957, 1630, 1589, 1541, 1491, 1464, 1287, 1246, 1028 and 756; δ_H (500 MHz) 7.58 (1 H, d, *J* 7.5, ArH), 7.42 (1 H, dd, *J* 7.5, 1.0, ArH), 7.32–7.23 (3 H, m, ArH), 7.13 (1 H, d, *J* 7.5, ArH), 7.01 (1 H, t, *J* 7.5, ArH), 6.81 (1 H, d, *J* 8.0, ArH), 6.38 (1 H, t, *J* 5.8, NH), 6.15 (1 H, s, C*H*OH), 5.21 (1 H, br s, OH), 3.63 (3 H, s, OCH_3), 3.39–3.34 (2 H, m, NCH_2), 1.53–1.47 (2 H, m, NCH_2CH_2), 1.38–1.31 (2 H, m, CH_2CH_3) and 0.93 (3 H, t, *J* 7.3, CH_2CH_3); δ_C (125 MHz) 170.7 (C=O), 156.0 (C–O), 142.2 (C), 135.9 (C), 130.30 (C), 130.28 (CH), 128.6 (CH), 128.3 (CH), 127.45 (CH), 127.36 (CH), 127.33 (CH), 120.4 (CH), 110.2 (CH), 69.6 (CHOH), 55.2 (OCH_3), 39.8 (NCH_2), 31.4 (CH_2), 20.0 (CH_2) and 13.7 (CH_3); HRMS (ESI$^+$): found, 336.1561. $C_{19}H_{23}NaNO_3$ (M+Na) requires 336.1570.

On standing at rt in EtOAc solution for 2–3 months, an intramolecular cyclisation occurred to give, after purification by column chromatography (SiO_2, Et_2O/hexane 2:3), at R_f 0.55, **23f** (0.2623 g, 55%) as a pale orange solid, mp 73–76 °C; ν_{max}/cm^{-1} 1767, 1589, 1491, 1464, 1281, 1248, 1061, 999, 737, 687 and 621; δ_H (500 MHz) 7.91 (1 H, d, *J* 7.5, ArH), 7.59 (1 H, td, *J* 7.5, 1.0, ArH), 7.49 (1 H, t, *J* 7.5, ArH), 7.43 (1 H, dd, *J* 7.8, 0.8, ArH), 7.32–7.29 (1 H, m, ArH), 7.07 (1 H, dd, *J* 7.8, 1.8, ArH), 6.96 (1 H, d, *J* 8.5, ArH), 6.88 (1 H, td, *J* 7.5, 0.8, ArH), 6.84 (1 H, s, CHAr) and 3.89 (3 H, s, CH_3). The ^{1}H NMR spectral data was in accordance with that previously reported.[26]

N-Butyl-2-(hydroxy(2-methoxynaphthalen-1-yl)methyl)benzamide **22g** and 3-(2-Methoxynaphthalen-1-yl)phthalide **23g**

Reaction as described for **22b** using *n*-butyllithium (2.6 mL, 6.50 mmol) and *N*-butyl-2-((2-methoxynaphthalen-1-yl)methoxy)benzamide **21g** (0.7263 g, 2.00 mmol) in dry THF (20 mL) gave **22g** as a yellow oil; ν_{max}/cm^{-1} 3297, 2934, 1625, 1597, 1513, 1465, 1251, 1086, 909, 809 and 735; δ_H (500 MHz) 7.95 (1 H, d, *J* 9.0, ArH), 7.88 (1 H, d, *J* 9.0, ArH), 7.79 (1 H, dd, *J* 8.0, 1.5, ArH), 7.71 (1 H, dd, *J* 7.8, 1.3, ArH), 7.48 (1 H, br s, NH), 7.39–7.26 (4 H, m, ArH), 7.17 (1 H, td, *J* 7.5, 1.0, ArH), 6.98 (1 H, dd, *J* 7.8, 0.8, ArH), 6.92 (1 H, d, *J* 7.0, C***H***OH), 5.68 (1 H, d, *J* 7.0, OH), 3.98 (3 H, s, OCH_3), 3.58–3.52 (1 H, m, NCH_2), 3.52–3.46 (1 H, m, NCH_2), 1.67–1.61 (2 H, m, NCH_2CH_2), 1.49–1.41 (2 H, m, CH_2CH_3) and 0.95 (3 H, t, *J* 7.5, CH_2CH_3); δ_C (125 MHz) 169.8 (C=O), 154.9 (C–O), 140.0 (C), 136.8 (C), 132.1 (C), 130.2 (CH), 130.1 (CH), 129.4 (C), 129.1 (CH), 128.3 (CH), 128.0 (CH), 127.9 (CH), 127.0 (CH), 124.1 (CH), 123.9 (CH), 120.9 (C), 113.1 (CH), 69.5 (CHOH), 56.5 (OCH_3), 40.0 (NCH_2), 31.5 (CH_2), 20.2 (CH_2) and 13.8 (CH_3); HRMS (ESI$^+$): found, 386.1722. $C_{23}H_{25}NaNO_3$ (M+Na) requires 386.1727.

On standing at rt in EtOAc solution for 2–3 months, an intramolecular cyclisation occurred to give, after purification by column chromatography (SiO_2, Et_2O/hexane 2:3), at R_f 0.45, **23g** (0.2068 g, 36%) as tan-coloured crystals, mp 127–130 °C; (lit.[28] 139 °C); ν_{max}/cm^{-1} 2940, 2841,

1748, 1597, 1512, 1464, 1288, 1252, 1094, 1067, 947, 812 and 737; δ_H (500 MHz) 8.02 (1 H, dd, *J* 6.8, 1.3, ArH), 7.88 (1 H, d, *J* 9.0, ArH), 7.79 (1 H, d, *J* 8.5, ArH), 7.62 (1 H, br s, ArH), 7.56–7.50 (2 H, m, ArH), 7.44 (1 H, s, CHAr), 7.38 (1 H, br s, ArH), 7.33 (1 H, t, *J* 7.3, ArH), 7.24 (1 H, d, *J* 9.0, ArH), 7.18–7.16 (1 H, m, ArH) and 3.74 (3 H, br s, CH_3); δ_C (125 MHz) 171.4 (C=O), 156.8 (C–O), 150.9 (C), 133.9 (CH), 132.5 (C), 131.9 (CH), 129.2 (C), 128.9 (2CH), 128.6 (CH), 127.4 (CH), 126.7 (C), 125.3 (CH), 123.7 (CH), 121.8 (CH), 115.2 (C), 113.6 (CH), 76.7 (CHAr) and 56.6 (CH_3); HRMS (ESI^+): found, 313.0826. $C_{19}H_{14}NaO_3$ (M+Na) requires 313.0835.

N-Butyl-2-((4-fluorophenyl)(hydroxy)methyl)benzamide **22h**, 2-Fluoroanthraquinone **24h** and 3-(4-Fluorophenyl)phthalide **23h**

Reaction as described for **22b** using *n*-butyllithium (2.0 mL, 5.00 mmol) and *N*-butyl-2-((4-fluorobenzyl)oxy)benzamide **21h** (0.4525 g, 1.50 mmol) in dry THF (15 mL) gave **22h** as a brown oil; ν_{max}/cm^{-1} 3304, 2959, 1626, 1601, 1500, 1439, 1304, 1221, 1155, 1030, 750 and 561; δ_H (500 MHz) 7.44–7.40 (2 H, m, ArH), 7.34–7.30 (2 H, m, ArH), 7.27–7.23 (2 H, m, ArH), 6.95 (2 H, t, *J* 8.8, ArH), 6.07 (1 H, t, *J* 5.8, NH), 5.80 (1 H, s, C*H*OH), 3.27–3.20 (1 H, m, NCH_2), 3.17–3.10 (1 H, m, NCH_2), 1.33–1.27 (2 H, m, NCH_2CH_2), 1.25–1.18 (2 H, m, CH_2CH_3) and 0.88 (3 H, t, *J* 7.3, CH_3); δ_C (125 MHz) 170.9 (C=O), 161.7 (d, J_{CF} 243, CF), 143.3 (C), 138.7 (d, J_{CF} 2.9, C), 135.7 (C), 130.8 (CH), 130.1 (CH), 127.92 (d, J_{CF} 7.5, 2CH) 127.90 (CH), 127.7 (CH), 114.5 (d, J_{CF} 21.1, 2CH), 74.7 (CHOH), 39.8 (NCH_2), 31.2 (CH_2), 19.9 (CH_2) and 13.6 (CH_3); δ_F (470 MHz) –116.4; HRMS (ESI^+): found, 324.1364. $C_{18}H_{20}FNaNO_2$ (M+Na) requires 324.1370.

On standing at rt in EtOAc solution for 2–3 months, an intramolecular cyclisation occurred to give, after purification by column chromatography (SiO_2, gradient elution, Et_2O/hexane 1:9 to Et_2O), **24h** (21.3 mg, 6%) as orange crystals, mp 197–201 °C; (lit.[29] 204 °C); δ_H (400 MHz) 8.37 (1 H, dd, *J* 8.8, 5.2, ArH), 8.34–8.30 (2 H, m, ArH), 7.95 (1 H, dd, *J* 8.8, 2.8, ArH), 7.86–7.79 (2 H, m, ArH) and 7.47 (1 H, ddd, *J* 8.4, 8.0, 2.8, ArH); δ_F (376 MHz) –101.6. The ^1H and ^{19}F NMR spectral data was in accordance with that previously reported.[30]

This was followed by a second fraction to give **23h** (0.1839 g, 54%) as pale orange crystals, mp 95–98 °C; (lit.[31] 100–101 °C); δ_H (400 MHz) 7.96 (1 H, d, *J* 7.6, ArH), 7.67 (1 H, td, *J* 7.6, 1.2, ArH), 7.57 (1 H, t, *J* 7.4, ArH), 7.32 (1 H, dd, *J* 7.6, 0.8, ArH), 7.28–7.23 (2 H, m, ArH), 7.06 (2 H, t, *J* 8.6, ArH) and 6.40 (1 H, s, CHAr); δ_F (376 MHz) –111.8. The ^1H NMR spectral data was in accordance with that previously reported.[32]

N-Butyl-2-((3-fluorophenyl)(hydroxy)methyl)benzamide **22i** and 3-(3-Fluorophenyl)phthalide **23i**

Reaction as described for **22b** using *n*-butyllithium (2.6 mL, 6.50 mmol) and *N*-butyl-2-((3-fluorobenzyl)oxy)benzamide **21i** (0.6022 g, 2.00 mmol) in dry THF (20 mL) gave **22i** as a brown oil; ν_{max}/cm^{-1} 3289, 2959, 1624, 1591, 1541, 1447, 1306, 1246, 1028, 750 and 691; δ_H (500 MHz) 7.43–7.40 (2 H, m, ArH), 7.33–7.29 (2 H, m, ArH), 7.20 (1 H, td, *J* 8.0, 6.0, ArH), 7.03–6.99 (2 H, m, ArH), 6.89 (1 H, td, *J* 8.3, 2.5, ArH), 6.22 (1 H, t, *J* 4.8, NH), 5.99 (1 H, br s, OH), 5.76 (1 H, s, C*H*OH), 3.26–3.19 (1 H, m, NCH_2), 3.13–3.07 (1 H, m, NCH_2), 1.31–1.25 (2 H, m, NCH_2CH_2), 1.23–1.16 (2 H, m, CH_2CH_3) and 0.86 (3 H, t, *J* 7.0, CH_3); δ_C (125 MHz) 170.8 (C=O), 162.6 (d, J_{CF} 244, CF), 145.8 (d, J_{CF} 6.8, C), 142.8 (C), 135.7 (C), 130.8 (CH), 130.2 (CH), 129.2 (d, J_{CF} 8.0, CH), 128.0 (CH), 127.8 (CH), 121.9 (d, J_{CF} 2.6, CH), 113.5 (d, J_{CF} 20.6, CH), 113.3 (d, J_{CF} 22.0, CH), 74.8 (d, J_{CF} 0.8, CHOH), 39.8 (NCH_2), 31.1 (CH_2), 19.9 (CH_2) and 13.6 (CH_3); δ_F (376 MHz) –113.6; HRMS (ESI^+): found, 324.1364. $C_{18}H_{20}FNaNO_2$ (M+Na) requires 324.1370.

On standing at rt in EtOAc solution for 2–3 months, an intramolecular cyclisation occurred to give, after purification by repeated column chromatography (SiO_2, Et_2O/hexane 1:4 then CH_2Cl_2), **23i** (49.7 mg, 11%) as a tan-coloured solid, mp 87–90 °C; (lit.[33] 85–87 °C); δ_H (400 MHz) 7.97 (1 H, d, *J* 8.0, ArH), 7.67 (1 H, td, *J* 7.6, 1.2, ArH), 7.60–7.55 (1 H, m, ArH), 7.40–7.34 (2 H, m, ArH), 7.13–7.11 (1 H, m, ArH), 7.07 (1 H, tdd, *J* 8.4, 2.4, 0.8, ArH), 6.99–6.95 (1 H, m, ArH) and 6.39 (1 H, s, CHAr); δ_F (376 MHz) –111.5. The ^{1}H NMR spectral data was in accordance with that previously reported.[33]

N-Butyl-2-((2-fluorophenyl)(hydroxy)methyl)benzamide **22j**, 1-(Butylamino)anthraquinone **24j** and 3-(2-Fluorophenyl)phthalide **23j**

Reaction as described for **22b** using *n*-butyllithium (2.6 mL, 6.50 mmol) and *N*-butyl-2-((2-fluorobenzyl)oxy)benzamide **21j** (0.6033 g, 2.00 mmol) in dry THF (20 mL) gave **22j** as a brown oil; ν_{max}/cm^{-1} 3300, 3202, 2934, 2872, 1624, 1551, 1456, 1314, 1227, 1018, 806, 756 and 691; δ_H (500 MHz) 7.71 (1 H, td, *J* 7.5, 1.5, ArH), 7.37 (1 H, dd, *J* 7.5, 1.0, ArH), 7.32 (1 H, td, *J* 7.5, 1.5, ArH), 7.26–7.21 (2 H, m, ArH), 7.17 (1 H, td, *J* 7.5, 1.0, ArH), 7.14 (1 H, d, *J* 8.0, ArH), 6.90 (1 H, ddd, *J* 10.5, 8.3, 1.3, ArH), 6.39 (1 H, t, *J* 5.5, NH), 6.05 (1 H, s, C*H*OH), 5.86 (1 H, br s, OH), 3.28 (2 H, td, *J* 7.3, 5.5, NCH_2), 1.48–1.42 (2 H, m, NCH_2CH_2), 1.34–1.26 (2 H, m, CH_2CH_3) and 0.91 (3 H, t, *J* 7.3, CH_3); δ_C (125 MHz) 170.9 (C=O), 159.4 (d, J_{CF} 245, CF), 142.1 (C), 135.5 (C), 130.7 (CH), 129.3 (d, J_{CF} 13.0, C), 128.8 (CH), 128.6 (d, J_{CF} 8.0, CH), 128.1 (d, J_{CF} 3.8, CH), 127.7 (CH), 127.3 (CH), 123.7 (d, J_{CF} 3.1, CH), 114.7 (d, J_{CF} 21.0, CH), 69.1 (d, J_{CF} 2.5, CHOH), 39.8 (NCH_2), 31.2 (CH_2), 19.9 (CH_2) and 13.6 (CH_3); δ_F (376 MHz) –116.2; HRMS (ESI$^+$): found, 324.1363. $C_{18}H_{20}FNaNO_2$ (M+Na) requires 324.1370.

On standing at rt in EtOAc solution for 2–3 months, an intramolecular cyclisation occurred to give, after purification by column chromatography (SiO_2, Et_2O/hexane 1:4), at R_f 0.70, **24j** (46.9 mg, 8%) as a dark red solid, mp 70–73 °C; (lit.[34] 81–82 °C); δ_H (400 MHz) 9.73 (1 H, br s, NH), 8.26 (1 H, dd, *J* 7.6, 1.2, ArH), 8.23 (1 H, dd, *J* 7.6, 1.2, ArH), 7.75 (1 H, td, *J* 7.6, 1.6, ArH), 7.69 (1 H, td, *J* 7.4, 1.6, ArH), 7.57 (1 H, dd, *J* 7.6, 1.2, ArH), 7.54–7.50 (1 H, m, ArH), 7.04 (1 H, dd, *J* 8.4, 1.2, ArH), 3.33 (2 H, td, *J* 7.0, 5.2, NCH_2), 1.79–1.72 (2 H, m, NCH_2CH_2), 1.57–1.48 (2 H, m, CH_2CH_3) and 1.01 (3 H, t, *J* 7.4, CH_3); δ_C (125 MHz) 184.8 (C=O), 183.8 (C=O), 151.7 (C), 135.2 (CH), 135.0 (C), 134.5 (C), 133.9 (CH), 132.9 (C), 132.8 (CH), 126.64 (CH), 126.56 (CH), 117.9 (CH), 115.5 (CH), 112.7 (C), 42.6 (NCH_2), 31.1 (CH_2), 20.3 (CH_2) and 13.9 (CH_3). The ^{1}H NMR spectral data was in accordance with that previously reported.[35]

This was followed by a second fraction to give, at R_f 0.45, **23j** (0.2817 g, 62%) as orange crystals, mp 94–97 °C; (lit.[34] 98–99 °C); δ_H (400 MHz) 7.95 (1 H, d, *J* 7.6, ArH), 7.66 (1 H, td, *J* 7.4, 1.2, ArH), 7.55 (1 H, t, *J* 7.4, ArH), 7.43 (1 H, dt, *J* 7.6, 0.8, ArH), 7.38–7.32 (1 H, m, ArH), 7.17–7.08 (3 H, m, ArH) and 6.73 (1 H, s, CHAr); δ_F (376 MHz) –118.3. The ^{1}H NMR spectral data was in accordance with that previously reported.[36]

3-(Thiophen-2-yl)phthalide **23q**

Reaction as described for **22b** using *n*-butyllithium (2.6 mL, 6.50 mmol) and *N*-butyl-2-(thiophen-2-ylmethoxy)benzamide **21q** (0.5790 g, 2.00 mmol) in dry THF (20 mL) gave *N*-butyl-2-(hydroxy(thiophen-2-yl)methyl)benzamide as a brown oil. On standing at rt in EtOAc solution for 2–3 months, an intramolecular cyclisation occurred to give, after purification by column chromatography (SiO_2, Et_2O/hexane 2:3), at R_f 0.50, **23q** (0.2488 g, 58%) as tan-coloured crystals, mp 74–78 °C; δ_H (400 MHz) 7.94 (1 H, d, *J* 7.6, ArH), 7.70 (1 H, td, *J* 7.6, 1.2, ArH), 7.59 (1 H, t, *J* 7.4, ArH), 7.47 (1 H, dd, *J* 7.6, 0.8, ArH), 7.35 (1 H, dd, *J* 4.8, 1.2, ArH), 7.14 (1 H,

ddd, *J* 3.6, 1.2, 0.8, ArH), 7.01 (1 H, dd, *J* 4.8, 3.6, ArH) and 6.67 (1 H, s, CHAr). The ^{1}H NMR spectral data was in accordance with that previously reported.[37]

N-Butyl-2-(1-hydroxy-1-phenylethyl)benzamide **22s** and 3-Methyl-3-phenylphthalide **23s**

Reaction as described for **22b** using *n*-butyllithium (2.6 mL, 6.50 mmol) and *N*-butyl-2-(1-phenylethoxy)benzamide **21s** (0.5950 g, 2.00 mmol) in dry THF (20 mL) gave **22s** as an orange oil which was used without further purification; ν_{max}/cm^{-1} 3291, 2957, 1626, 1541, 1445, 1308, 1225, 1123, 1047, 928, 758 and 700; δ_H (500 MHz) 7.74 (1 H, d, *J* 8.0, ArH), 7.50–7.47 (1 H, m, ArH), 7.30 (2 H, d, *J* 4.0, ArH), 7.23–7.17 (4 H, m, ArH), 7.15–7.12 (1 H, m, ArH), 6.84 (1 H, br s, OH), 5.64 (1 H, t, *J* 5.8, NH), 2.91–2.77 (2 H, m, NCH_2), 1.81 (3 H, s, C*Me*OH), 1.14–0.99 (4 H, m, $NCH_2CH_2CH_2$) and 0.82 (3 H, t, *J* 7.0, CH_2CH_3); δ_C (125 MHz) 172.6 (C=O), 149.2 (C), 146.2 (C), 136.7 (C), 130.0 (CH), 128.2 (CH), 127.4 (CH), 127.3 (2CH), 126.7 (CH), 126.1 (CH), 125.8 (2CH), 75.9 (COH), 39.6 (NCH_2), 31.2 (C*Me*OH), 30.8 (CH_2), 19.9 (CH_2) and 13.6 (CH_3); HRMS (ESI$^+$): found, 320.1614. $C_{19}H_{23}NaNO_2$ (M+Na) requires 320.1621.

On standing at rt for 3 d, an intramolecular cyclisation reaction occurred to give, after purification by column chromatography (SiO_2, Et_2O/hexane 1:4), at R_f 0.45, **23s** (0.3474 g, 77%) as a colourless solid, mp 76–78 °C; (lit.[38] 76.8–78 °C); δ_H (400 MHz) 7.89 (1 H, dt, *J* 7.6, 0.8, ArH), 7.64 (1 H, td, *J* 7.6, 0.8, ArH), 7.51–7.43 (4 H, m, ArH), 7.35–7.26 (3 H, m, ArH) and 2.02 (3 H, s, CH_3). The ^{1}H NMR spectral data was in accordance with that previously reported.[39]

N-Butyl-2-(1-hydroxyallyl)benzamide **22t** and 2-Butyl-3-ethyl-3-hydroxyisoindolin-1-one **27**

Reaction as described for **22b** using *n*-butyllithium (6.6 mL, 16.5 mmol) and 2-(allyloxy)-*N*-butylbenzamide **21t** (1.16 g, 4.97 mmol) in dry THF (50 mL) gave, after purification by column chromatography (SiO_2, Et_2O/hexane 1:1), at R_f 0.35, an inseparable 1:1 mixture of isomers **22t** and **27** (0.56 g, 48%) as an orange oil.

Data for **22t** (mixture): δ_H (400 MHz) 7.44–7.33 (3 H, m, ArH), 7.25 (1 H, td, *J* 7.2, 1.6, ArH), 6.83 (1 H, t, *J* 5.2, NH), 6.05–5.97 (1 H, m, C*H*=CH_2), 5.26–5.21 (1 H, m, C*H*OH), 5.16–5.12 (2 H, m, CH=C*H*$_2$), 3.40–3.25 (2 H, m, NCH_2), 1.59–1.50 (2 H, m, NCH_2CH_2), 1.41–1.26 (2 H, m, CH_2CH_3) and 0.93 (3 H, t, *J* 7.2, CH_3); δ_C (100 MHz) 170.6 (C=O), 141.3 (C), 138.7 (CH), 135.5 (C), 130.6 (CH), 128.8 (CH), 127.8 (CH), 127.6 (CH), 114.7 (=CH_2), 73.3 (CHOH), 39.8 (NCH_2), 31.2 (CH_2), 20.0 (CH_2) and 13.6 (CH_3).

On standing at rt for several months, **22t** isomerised to give **27** as colourless crystals suitable for X-ray structure determination, mp 85–89 °C.

Data for **27** (pure): ν_{max}/cm^{-1} 3296, 2959, 1667, 1414, 1325, 1088, 1043, 851, 762, 706, 608 and 584; δ_H (500 MHz) 7.55–7.50 (2 H, m, ArH), 7.47 (1 H, d, *J* 7.5, ArH), 7.38 (1 H, t, *J* 7.5, ArH), 3.98 (1 H, br s, OH), 3.40–3.34 (1 H, m, NCH_2), 3.00–2.94 (1 H, m, NCH_2), 2.20–2.12 (1 H, m, C(OH)CH_2), 2.10–2.03 (1 H, m, C(OH)CH_2), 1.70–1.61 (1 H, m, NCH_2CH_2), 1.59–1.50 (1 H, m, NCH_2CH_2), 1.36–1.28 (2 H, m, $CH_2CH_2CH_3$), 0.91 (3 H, t, *J* 7.5, $CH_2CH_2CH_3$) and 0.47 (3 H, t, *J* 7.5, C(OH)CH_2CH_3); δ_C (125 MHz) 167.6 (C=O), 146.5 (C), 132.0 (CH), 131.4 (C), 129.1 (CH), 122.9 (CH), 121.5 (CH), 92.0 (C(OH)Et), 38.4 (NCH_2), 30.9 (CH_2), 29.0 (CH_2), 20.6 (CH_2), 13.7 (CH_3) and 7.8 (CH_3); HRMS (ESI$^+$): found, 234.1483. $C_{14}H_{20}NO_2$ (M+H) requires *M*, 234.1489.

For X-ray structural data of **27** see page S189

3-(2-Methylprop-1-en-1-yl)phthalide **23u**

Reaction as described for **22b** using *n*-butyllithium (2.6 mL, 6.50 mmol) and *N*-butyl-2-((3-

methylbut-2-en-1-yl)oxy)benzamide **21u** (0.5226 g, 2.00 mmol) in dry THF (20 mL) gave *N*-butyl-2-(1-hydroxy-3-methylbut-2-en-1-yl)benzamide as an orange oil. On standing at rt in EtOAc solution for 2–3 months, an intramolecular cyclisation occurred to give, after purification by column chromatography (SiO_2, Et_2O/hexane 1:4), at R_f 0.45, **23u** (98.3 mg, 26%) as a colourless solid, mp 48–50 °C; ν_{max}/cm^{-1} 2976, 2913, 1749, 1437, 1375, 1281, 1096, 1065, 964, 756 and 691; δ_H (400 MHz) 7.88 (1 H, d, *J* 7.6, ArH), 7.67 (1 H, td, *J* 7.6, 1.2, ArH), 7.54–7.50 (1 H, m, ArH), 7.37 (1 H, dq, *J* 7.6, 0.8, ArH), 6.15 (1 H, d, *J* 9.2, OCH), 5.14–5.09 (1 H, m, C*H*=CMe_2), 1.97 (3 H, d, *J* 1.2, CH_3) and 1.84 (3 H, d, *J* 1.6, CH_3); δ_C (125 MHz) 170.6 (C=O), 150.0 (C), 141.6 (C), 134.0 (CH), 129.0 (CH), 125.9 (C), 125.4 (CH), 122.3 (CH), 120.1 (CH), 78.4 (OCH), 25.8 (CH_3) and 18.6 (CH_3); HRMS (NSI^+): found, 189.0906. $C_{12}H_{13}O_2$ (M+H) requires 189.0910.

N-Butyl-4-hydroxybenzamide **28**

A mixture of methyl 4-hydroxybenzoate (30.43 g, 0.200 mol) and *n*-butylamine (100 mL, 74.00 g, 1.01 mol) was heated at reflux for 4 d before being concentrated *in vacuo*. The residue was acidified to pH 1 by addition of 2 M HCl and extracted with Et_2O (3 × 100 mL). The combined organic layers were washed with water (100 mL) before being dried and evaporated. The crude residue was recrystallised (EtOAc/PhMe) to give **28** (28.01 g, 72%) as colourless crystals, mp 118–120 °C; (lit.[40] 118.5–119.5 °C); δ_H (500 MHz) 7.95 (1 H, br s, OH), 7.62 (2 H, d, *J* 8.8, 2,6-H), 6.86 (2 H, d, *J* 8.8, 3,5-H), 6.17 (1 H, t, *J* 5.5, NH), 3.44 (2 H, td, *J* 7.0, 5.5, NCH_2), 1.62–1.56 (2 H, m, NCH_2CH_2), 1.44–1.36 (2 H, m, CH_2CH_3) and 0.94 (3 H, t, *J* 7.3, CH_3). The ^{1}H NMR spectral data was in accordance with that previously reported.[41]

4-(Benzyloxy)-*N*-butylbenzamide **29a**

N-Butyl-4-hydroxybenzamide **28** (3.87 g, 20.0 mmol) was added to a stirred suspension of sodium hydride (60% in mineral oil, pre-washed with hexane, 0.82 g, 20.5 mmol) in DMF (20 mL) and the mixture was stirred at rt for 15 min before benzyl bromide (2.4 mL, 3.45 g, 20.2 mmol) was added. After stirring for 18 h at rt, the reaction mixture was poured into water and extracted with CH_2Cl_2 followed by Et_2O (× 3). The combined organic layers were washed with brine (× 5) and 2 M NaOH before being dried and evaporated. Recrystallisation of the residue (EtOAc/hexane) gave **29a** (4.68 g, 82%) as colourless crystals, mp 126–128 °C; (lit.[42] 119.1–119.7 °C); δ_H (500 MHz) 7.72 (2 H, d, *J* 8.8, 2,6-H), 7.44–7.38 (4 H, m, Ph), 7.35–7.32 (1 H, m, Ph), 6.99 (2 H, d, *J* 8.8, 3,5-H), 6.03 (1 H, t, *J* 5.5, NH), 5.11 (2 H, s, OCH_2), 3.44 (2 H, td, *J* 7.3, 5.5, NCH_2), 1.62–1.56 (2 H, m, NCH_2CH_2), 1.45–1.37 (2 H, m, CH_2CH_3) and 0.95 (3 H, t, *J* 7.3, CH_3). The ^{1}H NMR spectral data was in accordance with that previously reported.[42]

N-Butyl-4-((4-methylbenzyl)oxy)benzamide **29b**

Preparation as for **29a** using *N*-butyl-4-hydroxybenzamide **28** (0.96 g, 4.97 mmol), sodium hydride (0.20 g, 5.00 mmol) and 4-methylbenzyl chloride (0.66 mL, 0.70 g, 4.98 mmol) in DMF (10 mL) gave, after recrystallisation (EtOAc/hexane), **29b** (1.14 g, 77%) as colourless crystals, mp 136–138 °C; ν_{max}/cm^{-1} 3331, 2959, 2866, 1624, 1607, 1533, 1503, 1296, 1250, 1001, 837, 800 and 768; δ_H (500 MHz) 7.71 (2 H, d, *J* 8.5, ArH), 7.31 (2 H, d, *J* 8.0, ArH), 7.19 (2 H, d, *J* 8.0, ArH), 6.97 (2 H, d, *J* 8.5, ArH), 6.08 (1 H, t, *J* 5.5, NH), 5.05 (2 H, s, OCH_2), 3.43 (2 H, td, *J* 7.0, 5.5, NCH_2), 2.36 (3 H, s, CH_3), 1.61–1.55 (2 H, m, NCH_2CH_2), 1.44–1.36 (2 H, m, CH_2CH_3) and 0.95 (3 H, t, *J* 7.5, CH_2CH_3); δ_C (125 MHz) 166.9 (C=O), 161.2 (C–O), 138.0 (C), 133.3 (C), 129.3 (2CH), 128.5 (2CH), 127.6 (2CH), 127.2 (C), 114.5 (2CH), 70.0 (OCH_2), 39.7 (NCH_2), 31.8 (CH_2), 21.2

(CH_3), 20.1 (CH_2) and 13.8 (CH_3); HRMS (NSI$^+$): found, 298.1799. $C_{19}H_{24}NO_2$ (M+H) requires 298.1802.

N-Butyl-4-((2-methylbenzyl)oxy)benzamide **29c**

Preparation as for **29a** using *N*-butyl-4-hydroxybenzamide **28** (0.96 g, 4.97 mmol), sodium hydride (0.20 g, 5.00 mmol) and 2-methylbenzyl bromide (0.67 mL, 0.93 g, 5.00 mmol) in DMF (10 mL) gave, after recrystallisation (EtOAc/hexane), **29c** (1.07 g, 72%) as colourless crystals, mp 146–149 °C; ν_{max}/cm^{-1} 3302, 2963, 1624, 1605, 1506, 1250, 1229, 1003, 843 and 770; δ_H (500 MHz) 7.74 (2 H, d, *J* 8.8, ArH), 7.38 (1 H, d, *J* 7.5, ArH), 7.28–7.25 (1 H, m, ArH), 7.23–7.20 (2 H, m, ArH), 6.99 (2 H, d, *J* 8.8, ArH), 6.16 (1 H, t, *J* 5.8, NH), 5.06 (2 H, s, OCH_2), 3.43 (2 H, td, *J* 7.3, 5.8, NCH_2), 2.37 (3 H, s, CH_3), 1.61–1.55 (2 H, m, NCH_2CH_2), 1.44–1.36 (2 H, m, CH_2CH_3) and 0.95 (3 H, t, *J* 7.5, CH_2CH_3); δ_C (125 MHz) 166.9 (C=O), 161.2 (C–O), 136.7 (C), 134.1 (C), 130.4 (CH), 128.59 (2CH), 128.57 (CH), 128.4 (CH), 127.3 (C), 126.0 (CH), 114.4 (2CH), 68.6 (OCH_2), 39.7 (NCH_2), 31.8 (CH_2), 20.1 (CH_2), 18.9 (CH_3) and 13.8 (CH_3); HRMS (NSI$^+$): found, 298.1798. $C_{19}H_{24}NO_2$ (M+H) requires 298.1802.

N-Butyl-4-((4-methoxybenzyl)oxy)benzamide **29d**

Preparation as for **29a** using *N*-butyl-4-hydroxybenzamide **28** (0.96 g, 4.97 mmol), sodium hydride (0.20 g, 5.00 mmol) and 4-methoxybenzyl bromide (1.00 g, 4.97 mmol) in DMF (10 mL) gave, after recrystallisation (EtOAc/hexane), **29d** (1.23 g, 79%) as a colourless solid, mp 132–134 °C; ν_{max}/cm^{-1} 3326, 2959, 2934, 1630, 1609, 1541, 1504, 1244, 1003, 835, 824 and 768; δ_H (500 MHz) 7.72 (2 H, d, *J* 9.0, ArH), 7.35 (2 H, d, *J* 8.5, ArH), 6.98 (2 H, d, *J* 9.0, ArH), 6.92 (2 H, d, *J* 8.5, ArH), 6.04 (1 H, t, *J* 5.8, NH), 5.02 (2 H, s, OCH_2), 3.82 (3 H, s, OCH_3), 3.44 (2 H, td, *J* 7.3, 5.8, NCH_2), 1.62–1.56 (2 H, m, NCH_2CH_2), 1.44–1.37 (2 H, m, CH_2CH_3) and 0.95 (3 H, t, *J* 7.5, CH_2CH_3); δ_C (125 MHz) 166.9 (C=O), 161.2 (C–O), 159.5 (C–O), 129.2 (2CH), 128.5 (2CH), 128.3 (C), 127.2 (C), 114.5 (2CH), 114.0 (2CH), 69.8 (OCH_2), 55.3 (OCH_3), 39.7 (NCH_2), 31.8 (CH_2), 20.1 (CH_2) and 13.8 (CH_3); HRMS (NSI$^+$): found, 314.1749. $C_{19}H_{24}NO_3$ (M+H) requires 314.1751.

N-Butyl-4-((3-methoxybenzyl)oxy)benzamide **29e**

Preparation as for **29a** using *N*-butyl-4-hydroxybenzamide **28** (0.96 g, 4.97 mmol), sodium hydride (0.20 g, 5.00 mmol) and 3-methoxybenzyl bromide (1.00 g, 4.97 mmol) in DMF (10 mL) gave, after recrystallisation (EtOAc/hexane), **29e** (1.26 g, 81%) as colourless crystals, mp 89–91 °C; ν_{max}/cm^{-1} 3318, 2934, 1628, 1609, 1537, 1504, 1246, 1057, 1005, 891, 845 and 770; δ_H (500 MHz) 7.72 (2 H, d, *J* 9.0, ArH), 7.30 (1 H, t, *J* 8.0, ArH), 7.01–6.97 (4 H, m, ArH), 6.87 (1 H, dd, *J* 8.5, 2.0, ArH), 6.06 (1 H, t, *J* 5.8, NH), 5.08 (2 H, s, OCH_2), 3.82 (3 H, s, OCH_3), 3.43 (2 H, td, *J* 7.3, 5.8, NCH_2), 1.61–1.55 (2 H, m, NCH_2CH_2), 1.44–1.37 (2 H, m, CH_2CH_3) and 0.95 (3 H, t, *J* 7.3, CH_2CH_3); δ_C (125 MHz) 166.9 (C=O), 161.1 (C–O), 159.8 (C–O), 137.9 (C), 129.7 (CH), 128.6 (2CH), 127.4 (C), 119.6 (CH), 114.6 (2CH), 113.6 (CH), 112.9 (CH), 69.9 (OCH_2), 55.2 (OCH_3), 39.7 (NCH_2), 31.8 (CH_2), 20.1 (CH_2) and 13.8 (CH_3); HRMS (NSI$^+$): found, 314.1749. $C_{19}H_{24}NO_3$ (M+H) requires 314.1751.

N-Butyl-4-((2-methoxybenzyl)oxy)benzamide **29f**

Preparation as for **29a** using *N*-butyl-4-hydroxybenzamide **28** (0.96 g, 4.97 mmol), sodium hydride (0.20 g, 5.00 mmol) and 2-methoxybenzyl bromide (1.00 g, 4.97 mmol) in DMF (10 mL) gave, after recrystallisation (EtOAc/hexane), **29f** (1.25 g, 80%) as colourless crystals, mp 114–118 °C;

ν_{max}/cm^{-1} 3291, 2959, 2926, 1626, 1605, 1495, 1288, 1238, 1028, 997, 847 and 758; δ_H (400 MHz) 7.71 (2 H, d, *J* 9.0, ArH), 7.44–7.41 (1 H, m, ArH), 7.33–7.28 (1 H, m, ArH), 7.00 (2 H, d, *J* 9.0, ArH), 6.97 (1 H, td, *J* 7.4, 1.2, ArH), 6.92 (1 H, dd, *J* 8.4, 0.8, ArH), 6.03 (1 H, t, *J* 5.6, NH), 5.15 (2 H, s, OCH_2), 3.87 (3 H, s, OCH_3), 3.44 (2 H, td, *J* 7.2, 5.6, NCH_2), 1.62–1.55 (2 H, m, NCH_2CH_2), 1.45–1.36 (2 H, m, CH_2CH_3) and 0.95 (3 H, t, *J* 7.4, CH_2CH_3); δ_C (125 MHz) 167.0 (C=O), 161.4 (C–O), 156.8 (C–O), 129.1 (CH), 128.6 (CH), 128.5 (2CH), 127.1 (C), 124.7 (C), 120.6 (CH), 114.6 (2CH), 110.3 (CH), 65.1 (OCH_2), 55.4 (OCH_3), 39.7 (NCH_2), 31.8 (CH_2), 20.2 (CH_2) and 13.8 (CH_3); HRMS (NSI^+): found, 314.1750. $C_{19}H_{24}NO_3$ (M+H) requires 314.1751.

N-Butyl-4-((4-fluorobenzyl)oxy)benzamide **29g**

Preparation as for **29a** using *N*-butyl-4-hydroxybenzamide **28** (0.96 g, 4.97 mmol), sodium hydride (0.20 g, 5.00 mmol) and 4-fluorobenzyl chloride (0.6 mL, 0.72 g, 5.01 mmol) in DMF (10 mL) gave, after recrystallisation (EtOAc/hexane), **29g** (0.86 g, 57%) as colourless crystals, mp 140–141 °C; ν_{max}/cm^{-1} 3312, 2963, 2874, 1603, 1504, 1383, 1227, 1001, 874, 841, 816, 768 and 640; δ_H (300 MHz) 7.73 (2 H, d, *J* 8.9, ArH), 7.42–7.38 (2 H, dd, *J* 8.7, 5.4, ArH), 7.08 (2 H, t, *J* 8.7, ArH), 6.97 (2 H, d, *J* 8.9, ArH), 6.07 (1 H, t, *J* 5.7, NH), 5.06 (2 H, s, OCH_2), 3.44 (2 H, td, *J* 7.2, 5.7, NCH_2), 1.64–1.54 (2 H, m, NCH_2CH_2), 1.47–1.34 (2 H, m, CH_2CH_3) and 0.95 (3 H, t, *J* 7.4, CH_3); δ_C (125 MHz) 166.9 (C=O), 162.5 (d, J_{CF} 245, CF), 160.9 (C–O), 132.1 (d, J_{CF} 3.1, C), 129.3 (d, J_{CF} 8.3, 2CH), 128.6 (2CH), 127.5 (C), 115.6 (d, J_{CF} 21.4, 2CH), 114.5 (2CH), 69.4 (OCH_2), 39.7 (NCH_2), 31.8 (CH_2), 20.1 (CH_2) and 13.8 (CH_3); δ_F (282 MHz) –113.9; HRMS (NSI^+): found, 302.1548. $C_{18}H_{21}FNO_2$ (M+H) requires 302.1551.

N-Butyl-4-((2-fluorobenzyl)oxy)benzamide **29h**

Preparation as for **29a** using *N*-butyl-4-hydroxybenzamide **28** (0.96 g, 4.97 mmol), sodium hydride (0.20 g, 5.00 mmol) and 2-fluorobenzyl chloride (0.6 mL, 0.73 g, 5.05 mmol) in DMF (10 mL) gave, after recrystallisation (EtOAc/hexane), **29h** (1.01 g, 67%) as colourless crystals, mp 116–118 °C; ν_{max}/cm^{-1} 3298, 2928, 1624, 1607, 1506, 1252, 1231, 1053, 837, 752 and 656; δ_H (500 MHz) 7.73 (2 H, d, *J* 9.0, ArH), 7.49 (1 H, td, *J* 7.5, 1.5, ArH), 7.35–7.31 (1 H, m, ArH), 7.17 (1 H, td, *J* 7.5, 1.0, ArH), 7.12–7.08 (1 H, m, ArH), 7.01 (2 H, d, *J* 9.0, ArH), 6.06 (1 H, t, *J* 5.8, NH), 5.17 (2 H, s, OCH_2), 3.45 (2 H, td, *J* 7.3, 5.8, NCH_2), 1.62–1.56 (2 H, m, NCH_2CH_2), 1.45–1.38 (2 H, m, CH_2CH_3) and 0.95 (3 H, t, *J* 7.5, CH_3); δ_C (125 MHz) 166.9 (C=O), 160.9 (C–O), 160.4 (d, J_{CF} 246, CF), 129.9 (d, J_{CF} 8.1, CH), 129.6 (d, J_{CF} 3.8, CH), 128.6 (2CH), 127.6 (C), 124.3 (d, J_{CF} 3.5, CH), 123.5 (d, J_{CF} 14.1, C), 115.4 (d, J_{CF} 21.0, CH), 114.5 (2CH), 63.7 (d, J_{CF} 4.4, OCH_2), 39.7 (NCH_2), 31.8 (CH_2), 20.1 (CH_2) and 13.8 (CH_3); δ_F (282 MHz) –118.6; HRMS (NSI^+): found, 302.1547. $C_{18}H_{21}FNO_2$ (M+H) requires 302.1551.

N-Butyl-4-((2-methoxynaphthalen-1-yl)methoxy)benzamide **29i**

Preparation as for **29a** using *N*-butyl-4-hydroxybenzamide **28** (0.96 g, 4.97 mmol), sodium hydride (0.20 g, 5.00 mmol) and 1-(bromomethyl)-2-methoxynaphthalene (1.25 g, 4.98 mmol) in DMF (10 mL) gave, after recrystallisation (EtOAc/hexane), **29i** (1.43 g, 79%) as colourless crystals, mp 158–161 °C; ν_{max}/cm^{-1} 3275, 2957, 1626, 1605, 1568, 1504, 1233, 1186, 1094, 984, 843, 812 and 748; δ_H (500 MHz) 7.99 (1 H, d, *J* 9.0, ArH), 7.88 (1 H, d, *J* 9.0, ArH), 7.80 (1 H, d, *J* 8.0, ArH), 7.74 (2 H, d, *J* 8.5, ArH), 7.51–7.48 (1 H, m, ArH), 7.38–7.35 (1 H, m, ArH), 7.30 (1 H, d, *J* 9.0, ArH), 7.10 (2 H, d, *J* 8.5, ArH), 6.07 (1 H, t, *J* 5.8, NH), 5.59 (2 H, s, OCH_2), 3.98 (3 H, s, OCH_3), 3.44 (2 H, td, *J* 7.0, 5.8, NCH_2), 1.61–1.55 (2 H, m, NCH_2CH_2), 1.44–1.37 (2 H, m, CH_2CH_3) and

0.95 (3 H, t, *J* 7.3, CH_2CH_3); δ_C (125 MHz) 167.0 (C=O), 161.6 (C–O), 155.5 (C–O), 133.6 (C), 131.1 (CH), 129.1 (C), 128.5 (2CH), 128.4 (CH), 127.2 (CH), 127.0 (C), 123.7 (CH), 123.4 (CH), 116.3 (C), 114.6 (2CH), 113.1 (CH), 60.9 (OCH_2), 56.6 (OCH_3), 39.7 (NCH_2), 31.8 (CH_2), 20.2 (CH_2) and 13.8 (CH_3); HRMS (NSI^+): found, 364.1908. $C_{23}H_{26}NO_3$ (M+H) requires 364.1907.

N-Butyl-4-((4-nitrobenzyl)oxy)benzamide **29j**

Preparation as for **29a** using *N*-butyl-4-hydroxybenzamide **28** (0.96 g, 4.97 mmol), sodium hydride (0.20 g, 5.00 mmol) and 4-nitrobenzyl bromide (1.08 g, 5.00 mmol) in DMF (10 mL) gave, after recrystallisation (EtOAc/hexane), **29j** (1.32 g, 81%) as pale yellow crystals, mp 146–149 °C; ν_{max}/cm^{-1} 3292, 3092, 2951, 1624, 1530, 1508, 1350, 1256, 1186, 806 and 731; δ_H (500 MHz) 8.25 (2 H, d, *J* 8.8, ArH), 7.75 (2 H, d, *J* 8.8, ArH), 7.61 (2 H, d, *J* 8.8, ArH), 6.98 (2 H, d, *J* 8.8, ArH), 6.13 (1 H, t, *J* 5.8, NH), 5.21 (2 H, s, OCH_2), 3.44 (2 H, td, *J* 7.3, 5.8, NCH_2), 1.62–1.56 (2 H, m, NCH_2CH_2), 1.44–1.37 (2 H, m, CH_2CH_3) and 0.95 (3 H, t, *J* 7.5, CH_3); δ_C (125 MHz) 166.7 (C=O), 160.4 (C–O), 147.6 (CNO_2), 143.8 (C), 128.7 (2CH), 128.0 (C), 127.6 (2CH), 123.8 (2CH), 114.5 (2CH), 68.6 (OCH_2), 39.7 (NCH_2), 31.8 (CH_2), 20.1 (CH_2) and 13.8 (CH_3); HRMS (NSI^+): found, 329.1495. $C_{18}H_{21}N_2O_4$ (M+H) requires 329.1496.

N-Butyl-4-((3-nitrobenzyl)oxy)benzamide **29k**

Preparation as for **29a** using *N*-butyl-4-hydroxybenzamide **28** (0.96 g, 4.97 mmol), sodium hydride (0.20 g, 5.00 mmol) and 3-nitrobenzyl bromide (1.08 g, 5.00 mmol) in DMF (10 mL) gave, after recrystallisation (EtOAc/hexane), **29k** (1.48 g, 91%) as a colourless solid, mp 100–103 °C; ν_{max}/cm^{-1} 3292, 1624, 1531, 1506, 1350, 1256, 1186, 806, 731 and 650; δ_H (500 MHz) 8.33 (1 H, t, *J* 2.0, ArH), 8.20 (1 H, ddd, *J* 8.0, 2.5, 1.0, ArH), 7.78–7.76 (1 H, m, ArH), 7.75 (2 H, d, *J* 9.0, ArH), 7.59 (1 H, t, *J* 8.0, ArH), 7.00 (2 H, d, *J* 9.0, ArH), 6.09 (1 H, t, *J* 5.8, NH), 5.20 (2 H, s, OCH_2), 3.45 (2 H, td, *J* 7.3, 5.8, NCH_2), 1.63–1.57 (2 H, m, NCH_2CH_2), 1.45–1.37 (2 H, m, CH_2CH_3) and 0.96 (3 H, t, *J* 7.3, CH_3); δ_C (125 MHz) 166.8 (C=O), 160.4 (C–O), 148.4 (CNO_2), 138.6 (C), 133.1 (CH), 129.7 (CH), 128.7 (2CH), 128.0 (C), 123.1 (CH), 122.1 (CH), 114.5 (2CH), 68.6 (OCH_2), 39.8 (NCH_2), 31.8 (CH_2), 20.1 (CH_2) and 13.8 (CH_3); HRMS (NSI^+): found, 329.1495. $C_{18}H_{21}N_2O_4$ (M+H) requires 329.1496.

N-Butyl-4-((2-nitrobenzyl)oxy)benzamide **29l**

Preparation as for **29a** using *N*-butyl-4-hydroxybenzamide **28** (0.96 g, 4.97 mmol), sodium hydride (0.20 g, 5.00 mmol) and 2-nitrobenzyl bromide (1.08 g, 5.00 mmol) in DMF (10 mL) gave, after recrystallisation (EtOAc/hexane), **29l** (1.21 g, 74%) as light-sensitive tan-coloured crystals, mp 120–122 °C; ν_{max}/cm^{-1} 3345, 1630, 1609, 1522, 1503, 1339, 1244, 1182, 1051, 839 and 727; δ_H (500 MHz) 8.19 (1 H, dd, *J* 8.0, 1.0, ArH), 7.86 (1 H, dd, *J* 7.8, 0.8, ArH), 7.75 (2 H, d, *J* 8.8, ArH), 7.69 (1 H, td, *J* 7.8, 1.0, ArH), 7.53–7.49 (1 H, m, ArH), 7.01 (2 H, d, *J* 8.8, ArH), 6.10 (1 H, t, *J* 5.8, NH), 5.53 (2 H, s, OCH_2), 3.44 (2 H, td, *J* 7.3, 5.8, NCH_2), 1.63–1.57 (2 H, m, NCH_2CH_2), 1.45–1.37 (2 H, m, CH_2CH_3) and 0.96 (3 H, t, *J* 7.3, CH_3); δ_C (125 MHz) 166.8 (C=O), 160.4 (C–O), 146.8 (CNO_2), 134.1 (CH), 133.2 (C), 128.7 (2CH), 128.5 (CH), 128.4 (CH), 128.0 (C), 125.1 (CH), 114.6 (2CH), 66.8 (OCH_2), 39.7 (NCH_2), 31.8 (CH_2), 20.1 (CH_2) and 13.8 (CH_3); HRMS (NSI^+): found, 329.1494. $C_{18}H_{21}N_2O_4$ (M+H) requires 329.1496.

N-Butyl-4-((perfluorophenyl)methoxy)benzamide **29m**

Preparation as for **29a** using *N*-butyl-4-hydroxybenzamide **28** (0.96 g, 4.97 mmol), sodium hydride (0.20 g, 5.00 mmol) and 2,3,4,5,6-pentafluorobenzyl bromide (0.75 mL, 1.30 g, 4.97 mmol) in

DMF (10 mL) gave, after recrystallisation (EtOAc/hexane), **29m** (1.44 g, 78%) as a colourless solid, mp 143–145 °C; ν_{max}/cm^{-1} 3312, 2961, 2930, 1626, 1605, 1506, 1231, 1057, 937, 843 and 770; δ_H (500 MHz) 7.75 (2 H, d, *J* 8.8, ArH), 6.99 (2 H, d, *J* 8.8, ArH), 6.04 (1 H, t, *J* 5.8, NH), 5.16 (2 H, s, OCH_2), 3.45 (2 H, td, *J* 7.3, 5.8, NCH_2), 1.63–1.57 (2 H, m, NCH_2CH_2), 1.45–1.38 (2 H, m, CH_2CH_3) and 0.96 (3 H, t, *J* 7.3, CH_3); δ_C (125 MHz) 166.7 (C=O), 160.2 (C–O), 145.7 (dm, J_{CF} 249, 2 × Ar CF), 141.8 (dm, J_{CF} 254, Ar CF), 137.5 (dm, J_{CF} 251, 2 × Ar CF), 128.7 (2CH), 128.3 (C), 114.4 (2CH), 109.6 (td, J_{CF} 17.3, 3.6, Ar C), 57.4 (OCH_2), 39.8 (NCH_2), 31.7 (CH_2), 20.1 (CH_2) and 13.7 (CH_3); δ_F (282 MHz) –142.1 to –142.2 (m, 2CF), –152.1 to –152.3 (m, CF) and –161.2 to –161.4 (m, 2CF); HRMS (NSI^+): found, 374.1172. $C_{18}H_{17}F_5NO_2$ (M+H) requires 374.1174.

<u>4-(Diphenylmethoxy)-*N*-butylbenzamide **29n**</u>

Preparation as for **29a** using *N*-butyl-4-hydroxybenzamide **28** (0.96 g, 4.97 mmol), sodium hydride (0.20 g, 5.00 mmol) and benzhydryl bromide (1.23 g, 4.98 mmol) in DMF (10 mL) gave, after purification by column chromatography (SiO_2, Et_2O/hexane 3:2), at R_f 0.50, **29n** (1.50 g, 84%) as colourless crystals, mp 123–126 °C; ν_{max}/cm^{-1} 3333, 2959, 1626, 1603, 1543, 1501, 1231, 1175, 1011, 843, 743 and 694; δ_H (400 MHz) 7.63 (2 H, d, *J* 9.0, ArH), 7.42–7.39 (4 H, m, Ph), 7.36–7.31 (4 H, m, Ph), 7.29–7.25 (2 H, m, Ph), 6.96 (2 H, d, *J* 9.0, ArH), 6.26 (1 H, s, $CHPh_2$), 6.02 (1 H, t, *J* 5.6, NH), 3.39 (2 H, td, *J* 7.2, 5.6, NCH_2), 1.58–1.51 (2 H, m, NCH_2CH_2), 1.42–1.33 (2 H, m, CH_2CH_3) and 0.93 (3 H, t, *J* 7.4, CH_3); δ_C (100 MHz) 166.9 (C=O), 160.4 (C–O), 140.6 (2C), 128.7 (4CH), 128.4 (2CH), 127.9 (2CH), 127.4 (C), 126.8 (4CH), 115.8 (2CH), 81.7 ($CHPh_2$), 39.7 (NCH_2), 31.8 (CH_2), 20.1 (CH_2) and 13.8 (CH_3); HRMS (ESI^+): found, 360.1955. $C_{24}H_{26}NO_2$ (M+H) requires 360.1958.

<u>*N*-Butyl-4-(1-phenylethoxy)benzamide **29o**</u>

Preparation as for **29a** using *N*-butyl-4-hydroxybenzamide **28** (0.96 g, 4.97 mmol), sodium hydride (0.20 g, 5.00 mmol) and (1-bromoethyl)benzene (0.68 mL, 0.92 g, 4.98 mmol) in DMF (10 mL) gave, after purification by column chromatography (SiO_2, Et_2O/hexane 3:2), at R_f 0.45, **29o** (1.29 g, 87%) as a colourless oil which crystallised on standing, mp 52–56 °C; ν_{max}/cm^{-1} 3314, 2930, 1630, 1605, 1543, 1499, 1244, 1177, 1069, 843, 762 and 698; δ_H (500 MHz) 7.61 (2 H, d, *J* 9.0, ArH), 7.36–7.31 (4 H, m, Ph), 7.27–7.24 (1 H, m, Ph), 6.86 (2 H, d, *J* 9.0, ArH), 5.93 (1 H, t, *J* 5.8, NH), 5.36 (1 H, q, *J* 6.5, $CHCH_3$), 3.40 (2 H, td, *J* 7.0, 5.8, NCH_2), 1.65 (3 H, d, *J* 6.5, $CHCH_3$), 1.58–1.52 (2 H, m, NCH_2CH_2), 1.42–1.34 (2 H, m, CH_2CH_3) and 0.93 (3 H, t, *J* 7.5, CH_2CH_3); δ_C (125 MHz) 167.0 (C=O), 160.3 (C–O), 142.5 (C), 128.6 (2CH), 128.4 (2CH), 127.6 (CH), 127.0 (C), 125.4 (2CH), 115.5 (2CH), 76.0 ($CHCH_3$), 39.6 (NCH_2), 31.7 (CH_2), 24.4 ($CHCH_3$), 20.1 (CH_2) and 13.7 (CH_2CH_3); HRMS (NSI^+): found, 298.1799. $C_{19}H_{24}NO_2$ (M+H) requires 298.1802.

<u>*N*-Butyl-4-((3-methylbut-2-en-1-yl)oxy)benzamide **29p**</u>

Preparation as for **29a** using *N*-butyl-4-hydroxybenzamide **28** (0.96 g, 4.97 mmol), sodium hydride (0.20 g, 5.00 mmol) and prenyl bromide (0.74 g, 4.97 mmol) in DMF (10 mL) gave, after recrystallisation (EtOAc/hexane), **29p** (0.89 g, 69%) as colourless crystals, mp 72–74 °C; ν_{max}/cm^{-1} 3298, 2928, 1626, 1607, 1533, 1504, 1306, 1250, 1179, 997, 845, 770, 679 and 627; δ_H (500 MHz) 7.71 (2 H, d, *J* 8.8, ArH), 6.92 (2 H, d, *J* 8.8, ArH), 6.04 (1 H, t, *J* 5.5, NH), 5.50–5.46 (1 H, m, CH=CMe_2), 4.55 (2 H, d, *J* 6.5, OCH_2), 3.44 (2 H, td, *J* 7.3, 5.5, NCH_2), 1.80 (3 H, s, CH=CMe_2), 1.75 (3 H, s, CH=CMe_2), 1.62–1.56 (2 H, m, NCH_2CH_2), 1.45–1.37 (2 H, m, CH_2CH_3)

and 0.95 (3 H, t, *J* 7.5, CH_2CH_3); δ_C (125 MHz) 167.0 (C=O), 161.3 (C–O), 138.7 (C), 128.5 (2CH), 127.0 (C), 119.1 (CH), 114.4 (2CH), 64.9 (OCH_2), 39.7 (NCH_2), 31.8 (CH_2), 25.8 (CH_3), 20.2 (CH_2), 18.2 (CH_3) and 13.8 (CH_3); HRMS (NSI^+): found, 262.1798. $C_{16}H_{24}NO_2$ (M+H) requires 262.1802.

N-Butyl-4-(hydroxy(phenyl)methyl)benzamide **30a**

Under a nitrogen atmosphere, *n*-butyllithium (2.5 M in hexane, 6.6 mL, 16.5 mmol) was added dropwise to a stirred solution of 4-(benzyloxy)-*N*-butylbenzamide **29a** (1.41 g, 4.98 mmol) in dry THF (50 mL). After stirring at rt for 2 h, the reaction mixture was quenched by addition of sat. aq. NH_4Cl and extracted with Et_2O (× 3). The combined organic layers were washed with 2 M NaOH and water before being dried and evaporated. Purification of the residue by column chromatography (SiO_2, gradient elution, Et_2O/hexane 7:3 to Et_2O) and subsequent recrystallisation (EtOAc/hexane) gave **30a** (1.11 g, 79%) as colourless crystals, mp 113–114 °C; ν_{max}/cm^{-1} 3432, 3337, 2953, 2926, 1616, 1542, 1448, 1303, 1228, 1045, 736 and 694; δ_H (400 MHz) 7.67 (2 H, d, *J* 8.2, ArH), 7.41 (2 H, d, *J* 8.2, ArH), 7.36–7.30 (4 H, m, Ph), 7.28–7.24 (1 H, m, Ph), 6.14 (1 H, t, 5.6, NH), 5.85 (1 H, s, C*H*OH), 3.41 (2 H, td, *J* 7.2, 5.6, NCH_2), 2.76 (1 H, d, *J* 3.2, OH), 1.61–1.53 (2 H, m, NCH_2CH_2), 1.43–1.34 (2 H, m, CH_2CH_3) and 0.94 (3 H, t, *J* 7.4, CH_3); δ_C (125 MHz) 167.3 (C=O), 147.1 (C), 143.4 (C), 133.8 (C), 128.6 (2CH), 127.8 (CH), 126.9 (2CH), 126.6 (2CH), 126.5 (2CH), 75.7 (CHOH), 39.8 (NCH_2), 31.7 (CH_2), 20.1 (CH_2) and 13.8 (CH_3); HRMS (NSI^+): found, 284.1644. $C_{18}H_{22}NO_2$ (M+H) requires 284.1645.

N-Butyl-4-(hydroxy(*p*-tolyl)methyl)benzamide **30b**

Reaction as described for **29a** using *n*-butyllithium (2.7 mL, 6.75 mmol) and *N*-butyl-4-((4-methylbenzyl)oxy)benzamide **29b** (0.5937 g, 2.00 mmol) in dry THF (20 mL) gave, after purification by column chromatography (SiO_2, gradient elution, Et_2O/hexane 7:3 to Et_2O), **30b** (0.4183 g, 71%) as a colourless solid, mp 75–79 °C; ν_{max}/cm^{-1} 3304, 2957, 2928, 1634, 1541, 1504, 1308, 1016, 837, 752 and 671; δ_H (300 MHz) 7.66 (2 H, d, *J* 8.1, ArH), 7.41 (2 H, d, *J* 8.1, ArH), 7.22 (2 H, d, *J* 8.1, ArH), 7.13 (2 H, d, *J* 8.1, ArH), 6.15 (1 H, t, *J* 5.6, NH), 5.82 (1 H, s, C*H*OH), 3.42 (2 H, td, *J* 7.2, 5.6, NCH_2), 2.65 (1 H, br s, OH), 2.32 (3 H, s, CH_3), 1.62–1.53 (2 H, m, NCH_2CH_2), 1.43–1.29 (2 H, m, CH_2CH_3) and 0.94 (3H, t, *J* 7.4, CH_2CH_3); δ_C (125 MHz) 167.3 (C=O), 147.3 (C), 140.5 (C), 137.6 (C), 133.7 (C), 129.3 (2CH), 126.9 (2CH), 126.6 (2CH), 126.4 (2CH), 75.6 (CHOH), 39.8 (NCH_2), 31.7 (CH_2), 21.1 (CH_3), 20.1 (CH_2) and 13.8 (CH_3); HRMS (NSI^+): found, 298.1800. $C_{19}H_{24}NO_2$ (M+H) requires 298.1802.

N-Butyl-4-(hydroxy(*o*-tolyl)methyl)benzamide **30c**

Reaction as described for **29a** using *n*-butyllithium (2.7 mL, 6.75 mmol) and *N*-butyl-4-((2-methylbenzyl)oxy)benzamide **29c** (0.5959 g, 2.00 mmol) in dry THF (20 mL) gave, after purification by column chromatography (SiO_2, gradient elution, Et_2O/hexane 7:3 to Et_2O), **30c** (0.3999 g, 67%) as colourless crystals, mp 114–118 °C; ν_{max}/cm^{-1} 3308, 3217, 2957, 2872, 1628, 1553, 1317, 1013, 766, 746 and 665; δ_H (300 MHz) 7.70 (2 H, d, *J* 8.1, ArH), 7.39 (2 H, d, *J* 8.1, ArH), 7.25–7.14 (3 H, m, ArH), 6.10 (1 H, t, *J* 5.6, NH), 6.05 (1 H, s, C*H*OH), 3.44 (2 H, td, *J* 7.2, 5.6, NCH_2), 2.32 (1 H, br s, OH), 2.27 (3 H, s, CH_3), 1.65–1.55 (2 H, m, NCH_2CH_2), 1.45–1.35 (2 H, m, CH_2CH_3) and 0.95 (3 H, t, *J* 7.4, CH_2CH_3); δ_C (125 MHz) 167.3 (C=O), 146.3 (C), 141.0 (C), 135.5 (C), 133.9 (C), 130.7 (CH), 127.9 (CH), 127.1 (2CH), 126.9 (2CH), 126.6 (CH), 126.3 (CH), 73.0 (CHOH), 39.8 (NCH_2), 31.7 (CH_2), 20.1 (CH_2), 19.4 (CH_3) and 13.8 (CH_3); HRMS (NSI^+): found, 298.1802. $C_{19}H_{24}NO_2$ (M+H) requires 298.1802.

For X-ray structural data of **30c** see page S190

<u>*N*-Butyl-4-(hydroxy(4-methoxyphenyl)methyl)benzamide **30d**</u>
Reaction as described for **29a** using *n*-butyllithium (2.7 mL, 6.75 mmol) and *N*-butyl-4-((4-methoxybenzyl)oxy)benzamide **29d** (0.6276 g, 2.00 mmol) in dry THF (20 mL) gave, after purification by column chromatography (SiO_2, gradient elution, Et_2O/hexane 7:3 to Et_2O), **30d** (0.4561 g, 73%) as a pale yellow oil which slowly solidified on standing, mp 82–85 °C; ν_{max}/cm^{-1} 3345, 2959, 2872, 1614, 1535, 1512, 1248, 1173, 1032, 804 and 743; δ_H (500 MHz) 7.57 (2 H, d, *J* 8.3, ArH), 7.27 (2 H, d, *J* 8.3, ArH), 7.17 (2 H, d, *J* 8.8, ArH), 6.78 (2 H, d, *J* 8.8, ArH), 6.61 (1 H, t, *J* 5.5, NH), 5.68 (1 H, s, C*H*OH), 3.97 (1 H, br s, OH), 3.72 (3 H, s, OCH_3), 3.32 (2 H, td, *J* 7.3, 5.5, NCH_2), 1.54–1.48 (2 H, m, NCH_2CH_2), 1.36–1.29 (2 H, m, CH_2CH_3) and 0.90 (3 H, t, *J* 7.5, CH_2CH_3); δ_C (125 MHz) 167.6 (C=O), 158.8 (C–O), 147.6 (C), 135.8 (C), 133.2 (C), 127.8 (2CH), 126.8 (2CH), 126.2 (2CH), 113.6 (2CH), 74.9 (CHOH), 55.1 (OCH_3), 39.7 (NCH_2), 31.5 (CH_2), 20.0 (CH_2) and 13.7 (CH_3); HRMS (NSI^+): found, 314.1750. $C_{19}H_{24}NO_3$ (M+H) requires 314.1751.

<u>*N*-Butyl-4-(hydroxy(3-methoxyphenyl)methyl)benzamide **30e**</u>
Reaction as described for **29a** using *n*-butyllithium (2.7 mL, 6.75 mmol) and *N*-butyl-4-((3-methoxybenzyl)oxy)benzamide **29e** (0.6279 g, 2.00 mmol) in dry THF (20 mL) gave, after purification by column chromatography (SiO_2, gradient elution, Et_2O/hexane 7:3 to Et_2O), **30e** (0.5100 g, 81%) as a colourless solid, mp 70–73 °C; ν_{max}/cm^{-1} 3327, 2959, 2936, 1630, 1537, 1485, 1290, 1254, 1140, 1049, 772 and 745; δ_H (300 MHz) 7.67 (2 H, d, *J* 8.5, ArH), 7.42 (2 H, d, *J* 8.5, ArH), 7.24 (1 H, t, *J* 8.0, ArH), 6.95–6.90 (2 H, m, ArH), 6.80 (1 H, ddd, *J* 8.3, 7.5, 0.8, ArH), 6.13 (1 H, t, *J* 5.5, NH), 5.82 (1 H, s, C*H*OH), 3.77 (3 H, s, OCH_3), 3.42 (2 H, td, *J* 7.3, 5.5, NCH_2), 2.70 (1 H, br s, OH), 1.63–1.53 (2 H, m, NCH_2CH_2), 1.45–1.33 (2 H, m, CH_2CH_3) and 0.94 (3 H, t, *J* 7.5, CH_2CH_3); δ_C (75 MHz) 167.3 (C=O), 159.8 (C–O), 147.0 (C), 145.0 (C), 133.8 (C), 129.6 (CH), 127.0 (2CH), 126.5 (2CH), 118.9 (CH), 113.1 (CH), 112.1 (CH), 75.6 (CHOH), 55.2 (OCH_3), 39.8 (NCH_2), 31.7 (CH_2), 20.1 (CH_2) and 13.8 (CH_3); HRMS (NSI^+): found, 314.1750. $C_{19}H_{24}NO_3$ (M+H) requires 314.1751.

<u>*N*-Butyl-4-(hydroxy(2-methoxyphenyl)methyl)benzamide **30f**</u>
Reaction as described for **29a** using *n*-butyllithium (2.7 mL, 6.75 mmol) and *N*-butyl-4-((2-methoxybenzyl)oxy)benzamide **29f** (0.6265 g, 2.00 mmol) in dry THF (20 mL) gave, after purification by column chromatography (SiO_2, gradient elution, Et_2O/hexane 7:3 to Et_2O) and subsequent recrystallisation (PhMe), **30f** (0.3717 g, 59%) as colourless crystals, mp 128–130 °C; ν_{max}/cm^{-1} 3393, 2957, 2930, 1612, 1537, 1458, 1233, 1028 and 750; δ_H (300 MHz) 7.69 (2 H, d, *J* 8.2, ArH), 7.43 (2 H, d, *J* 8.2, ArH), 7.30–7.15 (2 H, m, ArH), 6.96–6.85 (2 H, m, ArH), 6.14 (1 H, t, *J* 5.6, NH), 6.06 (1 H, d, *J* 5.4, C***H***OH), 3.80 (3 H, s, OCH_3), 3.43 (2 H, td, *J* 7.2, 5.6, NCH_2), 3.19 (1 H, d, *J* 5.4, OH), 1.65–1.52 (2 H, m, NCH_2CH_2), 1.45–1.35 (2 H, m, CH_2CH_3) and 0.94 (3 H, t, *J* 7.2, CH_2CH_3); δ_C (75 MHz) 167.4 (C=O), 156.6 (C–O), 146.8 (C), 133.5 (C), 131.3 (C), 129.0 (CH), 127.8 (CH), 126.7 (2CH), 126.5 (2CH), 120.8 (CH), 110.7 (CH), 71.9 (CHOH), 55.4 (OCH_3), 39.7 (NCH_2), 31.7 (CH_2), 20.1 (CH_2) and 13.8 (CH_3); HRMS (NSI^+): found, 313.1749. $C_{19}H_{24}NO_3$ (M+H) requires 314.1751.

<u>*N*-Butyl-4-((4-fluorophenyl)(hydroxy)methyl)benzamide **30g**</u>
Reaction as described for **29a** using *n*-butyllithium (2.7 mL, 6.75 mmol) and *N*-butyl-4-((4-

fluorobenzyl)oxy)benzamide **29g** (0.6026 g, 2.00 mmol) in dry THF (20 mL) gave, after purification by column chromatography (SiO_2, Et_2O/hexane 4:1), at R_f 0.45, **30g** (0.1380 g, 23%) as a tan-coloured solid, mp 78–81 °C; ν_{max}/cm^{-1} 3323, 3217, 2961, 2874, 1626, 1557, 1506, 1221, 1159, 1015, 839 and 664; δ_H (300 MHz) 7.66 (2 H, d, *J* 8.0, ArH), 7.38 (2 H, d, *J* 8.0, ArH), 7.30 (2 H, dd, *J* 8.8, 5.6, ArH), 7.00 (2 H, t, *J* 8.8, ArH), 6.16 (1 H, t, *J* 5.6, NH), 5.83 (1 H, s, C*H*OH), 3.42 (2 H, td, *J* 7.2, 5.6, NCH_2), 2.93 (1 H, br s, OH), 1.63–1.53 (2 H, m, NCH_2CH_2), 1.45–1.32 (2 H, m, CH_2CH_3) and 0.94 (3 H, t, *J* 7.4, CH_3); δ_C (125 MHz) 167.3 (C=O), 162.2 (d, J_{CF} 244, CF), 147.0 (C), 139.2 (d, J_{CF} 3.0, C), 133.9 (C), 128.3 (d, J_{CF} 8.0, 2CH), 127.0 (2CH), 126.5 (2CH), 115.4 (d, J_{CF} 21.3, 2CH), 75.0 (CHOH), 39.8 (NCH_2), 31.7 (CH_2), 20.1 (CH_2) and 13.8 (CH_3); δ_F (376 MHz) –114.8; HRMS (NSI^+): found, 302.1550. $C_{18}H_{21}FNO_2$ (M+H) requires 302.1551.

N-Butyl-4-((2-fluorophenyl)(hydroxy)methyl)benzamide **30h**

Reaction as described for **29a** using *n*-butyllithium (2.7 mL, 6.75 mmol) and *N*-butyl-4-((2-fluorobenzyl)oxy)benzamide **29h** (0.6033 g, 2.00 mmol) in dry THF (20 mL) gave, after purification by column chromatography (SiO_2, Et_2O/hexane 7:3), at R_f 0.35, **30h** (0.3475 g, 58%) as an orange oil which slowly solidified on standing, mp 73–76 °C; ν_{max}/cm^{-1} 3318, 2959, 2872, 1630, 1541, 1456, 1306, 1225, 1032 and 754; δ_H (400 MHz) 7.54 (2 H, d, *J* 8.4, ArH), 7.45 (1 H, td, *J* 7.6, 2.0, ArH), 7.31 (2 H, d, *J* 8.4, ArH), 7.21–7.16 (1 H, m, ArH), 7.07 (1 H, td, *J* 7.6, 1.2, ArH), 6.94 (1 H, ddd, *J* 10.4, 8.4, 1.2, ArH), 6.48 (1 H, t, *J* 5.6, NH), 6.06 (1 H, s, C*H*OH), 4.14 (1 H, br s, OH), 3.32 (2 H, td, *J* 7.2, 5.6, NCH_2), 1.54–1.47 (2 H, m, NCH_2CH_2), 1.37–1.27 (2 H, m, CH_2CH_3) and 0.89 (3 H, t, *J* 7.4, CH_3); δ_C (125 MHz) 167.6 (C=O), 159.6 (d, J_{CF} 245, CF), 146.5 (C), 133.4 (C), 130.8 (d, J_{CF} 13.1, C), 129.0 (d, J_{CF} 8.1, CH), 127.6 (d, J_{CF} 3.9, CH), 126.8 (2CH), 126.3 (2CH), 124.2 (d, J_{CF} 3.2, CH), 115.1 (d, J_{CF} 21.4, CH), 69.0 (d, J_{CF} 2.9, CHOH), 39.7 (NCH_2), 31.5 (CH_2), 20.0 (CH_2) and 13.7 (CH_3); δ_F (376 MHz) –118.4; HRMS (NSI^+): found, 302.1549. $C_{18}H_{21}FNO_2$ (M+H) requires 302.1551.

N-Butyl-4-(1-hydroxy-1-phenylethyl)benzamide **30o**

Reaction as described for **29a** using *n*-butyllithium (2.7 mL, 6.75 mmol) and *N*-butyl-4-(1-phenylethoxy)benzamide **29o** (0.5943 g, 2.00 mmol) in dry THF (20 mL) gave, after purification by column chromatography (SiO_2, gradient elution, Et_2O/hexane 7:3 to Et_2O), **30o** (0.3867 g, 65%) as colourless crystals, mp 77–80 °C; ν_{max}/cm^{-1} 3387, 2930, 1620, 1535, 1493, 1366, 1287, 1096, 1067, 926, 762 and 694; δ_H (300 MHz) 7.67 (2 H, d, *J* 8.4, ArH), 7.46 (2 H, d, *J* 8.4, ArH), 7.40–7.20 (5 H, m, Ph), 6.13 (1 H, t, *J* 5.6, NH), 3.43 (2 H, td, *J* 7.2, 5.6, NCH_2), 2.38 (1 H, br s, OH), 1.95 (3 H, s, C*Me*OH), 1.62–1.52 (2 H, m, NCH_2CH_2), 1.45–1.32 (2 H, m, CH_2CH_3) and 0.94 (3 H, t, *J* 7.4, CH_2CH_3); δ_C (75 MHz) 167.3 (C=O), 151.4 (C), 147.4 (C), 133.3 (C), 128.3 (2CH), 127.2 (CH), 126.7 (2CH), 126.0 (2CH), 125.8 (2CH), 76.0 (COH), 39.7 (NCH_2), 31.7 (CH_2), 30.6 (CH_3), 20.1 (CH_2) and 13.8 (CH_3); HRMS (NSI^+): found, 298.1798. $C_{19}H_{24}NO_2$ (M+H) requires 298.1802.

N-Butyl-4-(1-hydroxy-3-methylbut-2-en-1-yl)benzamide **30p**

Reaction as described for **29a** using *n*-butyllithium (2.7 mL, 6.75 mmol) and *N*-butyl-4-((3-methylbut-2-en-1-yl)oxy)benzamide **29p** (0.5236 g, 2.00 mmol) in dry THF (20 mL) gave, after purification by column chromatography (SiO_2, gradient elution, Et_2O/hexane 7:3 to Et_2O), **30p** (71.8 mg, 14%) as a colourless oil; ν_{max}/cm^{-1} 3312, 2959, 2930, 1632, 1545, 1501, 1306, 1011, 847 and 764; δ_H (500 MHz) 7.67 (2 H, d, *J* 8.0, ArH), 7.35 (2 H, d, *J* 8.0, ArH), 6.46 (1 H, t, *J* 5.5, NH), 5.45 (1 H, d, *J* 9.0, C*H*OH), 5.34–5.31 (1 H, m, C*H*=CMe_2), 3.40 (2 H, td, *J* 7.3, 5.5, NCH_2),

2.83 (1 H, br s, OH), 1.78 (3 H, d, *J* 1.0, C*Me*$_2$), 1.73 (3 H, d, *J* 1.5, C*Me*$_2$), 1.60–1.54 (2 H, m, NCH$_2$C*H*$_2$), 1.42–1.34 (2 H, m, C*H*$_2$CH$_3$) and 0.93 (3 H, t, *J* 7.3, CH$_2$C*H*$_3$); δ_C (125 MHz) 167.5 (C=O), 147.8 (C), 135.4 (C), 133.3 (C), 127.3 (CH=), 126.9 (2CH), 125.8 (2CH), 70.1 (CHOH), 39.7 (NCH$_2$), 31.6 (CH$_2$), 25.7 (CH$_3$), 20.1 (CH$_2$), 18.3 (CH$_3$) and 13.7 (CH$_3$); HRMS (ESI$^+$): found, 262.1802. $C_{16}H_{24}NO_2$ (M+H) requires 262.1802.

2,4-Bis(benzyloxy)-*N*-butylbenzamide **31**

A solution of oxalyl chloride (2.6 mL, 3.90 g, 30.7 mmol) in dry toluene (50 mL) was added to a stirred mixture of 2,4-bis(benzyloxy)benzoic acid[1] (5.00 g, 15.0 mmol) and pyridine (1.4 mL, 1.37 g, 17.3 mmol) in dry toluene (50 mL). Once the addition was complete, the reaction mixture was stirred at rt for 30 min before being filtered and concentrated *in vacuo* to give 2,4-bis(benzyloxy)benzoyl chloride as an orange oil which was used without further purification.

A solution of 2,4-bis(benzyloxy)benzoyl chloride prepared as above (assuming 15.0 mmol) in toluene (20 mL) was added dropwise to a stirred 0 °C solution of *n*-butylamine (4.5 mL, 3.33 g, 45.5 mmol) in toluene (20 mL). Once the addition was complete, the reaction mixture was allowed to warm to rt for 1 h before being poured into water and washed with 2 M NaOH and brine. The organic layer was dried and evaporated to afford the crude amide product. Recrystallisation of this (EtOAc/hexane) gave **31** (5.17 g, 89%) as a colourless solid, mp 99–101 °C; ν_{max}/cm^{-1} 3336, 1628, 1538, 1306, 1179, 1117, 1029, 836, 750, 722 and 699; δ_H (400 MHz) 8.22 (1 H, d, *J* 8.6, ArH), 7.75 (1 H, t, *J* 5.2, NH), 7.44–7.32 (10 H, m, Ph), 6.70 (1 H, dd, *J* 8.6, 2.2, ArH), 6.66 (1 H, d, *J* 2.2, ArH), 5.10 (2 H, s, OCH$_2$), 5.09 (2 H, s, OCH$_2$), 3.32 (2 H, td, *J* 6.8, 5.2, NCH$_2$), 1.34–1.27 (2 H, m, NCH$_2$C*H*$_2$), 1.18–1.09 (2 H, m, C*H*$_2$CH$_3$) and 0.79 (3 H, t, *J* 7.2, CH$_3$); δ_C (100 MHz) 164.8 (C=O), 162.2 (C–O), 158.0 (C–O), 136.3 (C), 135.3 (C), 133.9 (CH), 128.9 (2CH), 128.8 (CH), 128.7 (2CH), 128.24 (2CH), 128.22 (CH), 127.6 (2CH), 115.1 (C), 106.4 (CH), 100.4 (CH), 71.3 (OCH$_2$), 70.2 (OCH$_2$), 39.3 (NCH$_2$), 31.2 (CH$_2$), 20.0 (CH$_2$) and 13.8 (CH$_3$); HRMS (ESI$^+$): found, 412.1870. $C_{25}H_{27}NaNO_3$ (M+Na) requires 412.1883.

4-(Benzyloxy)-*N*-butyl-2-(hydroxy(phenyl)methyl)benzamide **32**, 2-(Benzyloxy)-*N*-butyl-4-(hydroxy(phenyl)methyl)benzamide **33** and 5-(Benzyloxy)-3-phenylphthalide **34**

Reaction as described for **29a** using *n*-butyllithium (6.6 mL, 16.5 mmol) and 2,4-bis(benzyloxy)-*N*-butylbenzamide **31** (1.95 g, 5.01 mmol) in dry THF (50 mL) gave, after purification by column chromatography (SiO$_2$, Et$_2$O/hexane 7:3), at R$_f$ 0.50, **32** (1.01 g, 52%) as a pale yellow solid; δ_H (400 MHz) 7.35–7.17 (11 H, m, ArH and Ph), 6.88 (1 H, d, *J* 2.4, ArH), 6.81 (1 H, dd, *J* 8.4, 2.4, ArH), 6.15 (1 H, t, *J* 5.8, NH), 6.00 (1 H, d, *J* 7.6, CHOH), 5.78 (1 H, d, *J* 7.6, CHOH), 5.00 and 4.98 (2 H, AB pattern, J_{AB} 12.0, OCH$_2$), 3.25–3.05 (2 H, m, NCH$_2$), 1.31–1.24 (2 H, m, NCH$_2$C*H*$_2$), 1.23–1.14 (2 H, m, C*H*$_2$CH$_3$) and 0.85 (3 H, t, *J* 7.0, CH$_3$); δ_C (100 MHz) 170.5 (C=O), 160.2 (C–O), 145.5 (C), 142.5 (C), 136.1 (C), 129.5 (CH), 128.5 (2CH), 128.2 (C), 128.0 (CH), 127.7 (2CH), 127.4 (2CH), 126.7 (CH), 126.2 (2CH), 116.8 (CH), 112.9 (CH), 74.9 (CHOH), 69.9 (OCH$_2$), 39.7 (NCH$_2$), 31.2 (CH$_2$), 19.9 (CH$_2$) and 13.7 (CH$_3$); HRMS (NSI$^+$): found, 390.2060. $C_{25}H_{28}NO_3$ (M+H) requires 390.2064.

This was followed by a second fraction at R$_f$ 0.40 to give **33** (0.06 g, 3%) as a pale yellow solid, mp 99–102 °C; ν_{max}/cm^{-1} 3329, 2930, 1607, 1541, 1454, 1420, 1225, 1047, 1007, 743 and 696; δ_H (400 MHz) 8.07 (1 H, d, *J* 8.2, ArH), 7.84 (1 H, t, *J* 5.2, NH), 7.40–7.39 (4 H, m, Ph), 7.35–7.25 (6 H, m, Ph), 7.16 (1 H, d, *J* 0.8, ArH), 6.99 (1 H, dd, *J* 8.2, 0.8, ArH), 5.80 (1 H, s, C*H*OH), 5.09 (2 H, s, OCH$_2$Ph), 3.27 (2 H, td, *J* 6.8, 5.2, NCH$_2$), 1.31–1.23 (2 H, m, NCH$_2$C*H*$_2$), 1.15–1.06 (2 H, m, C*H*$_2$CH$_3$) and 0.77 (3 H, t, *J* 7.2, CH$_3$); δ_C (100 MHz) 165.0 (C=O), 156.8 (C–O), 149.0 (C),

143.3 (C), 135.4 (C), 132.3 (CH), 128.82 (2CH), 128.76 (CH), 128.5 (2CH), 128.3 (2CH), 127.7 (CH), 126.6 (2CH), 120.5 (C), 119.6 (CH), 110.1 (CH), 75.6 (CHOH), 71.3 (OCH_2), 39.4 (NCH_2), 31.1 (CH_2), 20.0 (CH_2) and 13.7 (CH_3); HRMS (NSI^+): found, 390.2064. $C_{25}H_{28}NO_3$ (M+H) requires 390.2064.
On standing at rt for 4 months, a lactonisation reaction of **32** occurred to give, after purification by column chromatography (SiO_2, Et_2O/hexane 1:1), at R_f 0.60, **34** (0.67 g, 82%) as a colourless solid, mp 150–152 °C; ν_{max}/cm^{-1} 2922, 1742, 1603, 1485, 1312, 1252, 1067, 970, 847, 768 and 691; δ_H (500 MHz) 7.85 (1 H, d, *J* 8.5, ArH), 7.37–7.31 (8 H, m, Ph), 7.28–7.24 (2 H, m, Ph), 7.11 (1 H, dd, *J* 8.5, 2.0, ArH), 6.81 (1 H, d, *J* 2.0, ArH), 6.29 (1 H, s, CHPh) and 5.05 and 5.04 (2 H, AB pattern, J_{AB} 11.6, CH_2); δ_C (125 MHz) 170.1 (C=O), 163.9 (C–O), 152.4 (C), 136.4 (C), 135.4 (C), 129.2 (CH), 128.9 (2CH), 128.6 (2CH), 128.3 (CH), 127.5 (2CH), 127.1 (CH), 126.9 (2CH), 117.9 (C), 117.3 (CH), 107.6 (CH), 82.0 (CHPh) and 70.5 (CH_2); HRMS (NSI^+): found, 317.1168. $C_{21}H_{17}O_3$ (M+H) requires 317.1172.

N-Butyl-2,4-bis(hydroxy(phenyl)methyl)benzamide **35**
Reaction as described for **29a** using *n*-butyllithium (8.8 mL, 22 mmol) and 2,4-bis(benzyloxy)-*N*-butylbenzamide **31** (1.95 g, 5.01 mmol) in dry THF (50 mL) gave, after purification by column chromatography (SiO_2, Et_2O/hexane 7:3), at R_f 0.35, **35** (1.02 g, 52%) as a colourless solid, mp 116–118 °C; ν_{max}/cm^{-1} 3341, 3061, 2957, 1639, 1533, 1454, 1011 and 696; δ_H (400 MHz) 7.40 (1 H, dd, *J* 3.2, 1.6, ArH), 7.34–7.16 (12 H, m, ArH and Ph), 5.89–5.84 (1 H, m, NH), 5.79–5.77 (2 H, m, C*H*OH), 3.20–3.02 (2 H, m, NCH_2), 1.26–1.11 (4 H, m, $CH_2CH_2CH_3$) and 0.85 (3 H, t, *J* 7.2, CH_3); δ_C (125 MHz) 170.79 and 170.78 (diastereomeric C=O), 146.62 and 146.60 (diastereomeric C), 143.7 (C), 143.1 (C), 142.88 and 142.84 (diastereomeric C), 134.85 and 134.81 (C), 128.7 (2CH), 128.5 and 128.4 (diastereomeric CH), 128.00 and 127.99 (diastereomeric CH), 127.8 (2CH), 126.8 (CH), 126.6 (2CH), 126.3 and 126.2 (diastereomeric 2CH), 125.7 (CH), 125.6 (CH), 75.7 and 75.6 (diastereomeric CHOH), 75.51 and 75.48 (diastereomeric CHOH), 39.8 (NCH_2), 31.1 (CH_2), 19.9 (CH_2) and 13.7 (CH_3); HRMS (NSI^+): found, 390.2063. $C_{25}H_{28}NO_3$ (M+H) requires 390.2064.

3-(Benzyloxy)-*N*-butylbenzamide **37a**
To a stirred solution of 3-(benzyloxy)benzoyl chloride[43] (6.82 g, 27.6 mmol) in CH_2Cl_2 (100 mL) at 0 °C, Et_3N (3.85 mL, 27.6 mmol) was added dropwise. The solution was stirred for 5 min and then *n*-butylamine (2.73 mL, 27.6 mmol) was added dropwise and the mixture stirred at rt for 18 h. The reaction mixture was poured into H_2O, extracted (x 3) with CH_2Cl_2, and the combined organic fractions were dried over $MgSO_4$ and concentrated to afford, after recrystallisation (EtOH) **37a** (5.80 g, 74%) as off-white crystals, mp 83−86 °C; ν_{max}/cm^{-1} 3298, 3229, 2961, 1626, 1603, 1580,1553, 1016 and 698; δ_H (400 MHz) 7.47−7.38 (5 H, m, ArH), 7.38−7.32 (2 H, m, ArH), 7.32−7.28 (1 H, m, ArH), 7.09 (1 H, ddd, *J* 7.8, 2.6, 1.5, ArH), 6.07 (1 H, br s, NH), 5.11 (2 H, s, OCH_2), 3.45 (2 H, td, *J* 7.1, 5.7, $NHCH_2$), 1.65–1.53 (2 H, m, $NHCH_2CH_2$), 1.51–1.30 (2 H, m, CH_2CH_3) and 0.96 (3 H, t, *J* 7.3, CH_2CH_3); δ_C (100 MHz) 167.2 (C=O), 158.9 (C–O), 136.5 (C), 136.4 (C), 129.6 (CH), 128.6 (CH), 128.1 (CH), 127.5 (CH), 118.8 (CH), 118.1 (CH), 113.3 (CH), 70.1 (OCH_2), 39.8 ($NHCH_2$), 31.7 (CH_2), 20.1 (CH_2) and 13.8 (CH_3); HRMS (ESI^+): found, 284.1636, $C_{18}H_{22}NO_2$ (M+H) requires 284.1651.

N-Butyl-3-hydroxybenzamide **36**
A stirred solution of methyl 3-hydroxybenzoate[44] (30.03 g, 197.4 mmol) and *n*-butylamine (98 mL) was heated under reflux for 2 days, the reaction mixture was concentrated, and the residue

acidified to pH 2 by addition of 2 M HCl, EtOAc (200 mL) was added, the layers separated, and the aqueous layer extracted with EtOAc (3 x 200 mL). The organic layers were dried over $MgSO_4$, filtered, and concentrated. The crude residue was purified *via* recrystallisation (PhMe/EtOAc 9:1) to give **36** (33.20 g, 87%) as colourless crystals, mp 110−113 °C; (lit.[45] 112−113 °C); δ_H (400 MHz, CD_3SOCD_3) 9.61 (1 H, s, OH), 8.32 (1 H, t, *J* 5.6, ArH), 7.24−7.22 (2 H, m, ArH), 6.89 (1 H, dt, *J* 3.1, 2.4, 2.1, 1.8, ArH), 3.38 (1 H, s, NH), 3.22 (2 H, td, *J* 7.1, 5.7, NHC*H*$_2$), 1.53−1.43 (2 H, m, $NHCH_2$C*H*$_2$), 1.37−1.25 (C*H*$_2$$CH_3$) and 0.89 (3 H, t, *J* 7.3, CH_2C*H*$_3$); δ_C (100 MHz, CD_3SOCD_3) 166.2 (C=O), 157.3 (C–O), 136.3 (C), 129.2 (CH), 117.9 (CH), 117.6 (CH), 114.2 (CH), 38.8 ($NHCH_2$), 31.3 (CH_2), 19.7 (CH_2) and 13.8 (CH_3). The ^1H and ^{13}C spectral data was in accordance with that previously reported.[45]

N-Butyl-3-((4-(*tert*-butyl)benzyl)oxy)benzamide **37b**

To a stirred suspension of sodium hydride (60% dispersion in mineral oil, pre-washed (x 3) with hexane, 0.20 g, 5.00 mmol) in DMF (10 mL) was added *N*-butyl-3-hydroxybenzamide **36** (0.96 g, 5.00 mmol) and the mixture was stirred at rt for 15 min before adding 1-(bromomethyl)-4-(*tert*-butyl)benzene (1.13 g, 5.00 mmol). After stirring for 18 h at rt, the reaction mixture was poured into water and extracted with CH_2Cl_2 followed by Et_2O (× 3). The combined organic layers were washed with water (× 5) before being dried over $MgSO_4$ and concentrated to give, after recrystallisation (PhMe/EtOAc), **37b** (0.95 g, 56%) as colourless crystals, mp 122−125 °C; ν_{max}/cm^{-1} 3281, 2961, 2932, 2866, 1630, 1584, 1549, 1242, 1009, 706, 554; δ_H (300 MHz) 7.45−7.37 (5 H, m, ArH), 7.37−7.32 (1 H , m, ArH), 7.31−7.28 (1 H, m, ArH), 7.09 (1 H, ddd, *J* 7.7, 2.6, 1.7, ArH), 6.11 (1 H, br s, NH), 5.06 (2 H, s, OCH_2), 3.45 (2 H, td, *J* 7.1, 5.7, NHC*H*$_2$), 1.64−1.52 (2 H, m, $NHCH_2$C*H*$_2$), 1.47−1.36 (2 H, m, C*H*$_2$$CH_3$), 1.33 (9 H, s, $C(CH_3)_3$) and 0.96 (3 H, t, *J* 7.3, CH_2C*H*$_3$); δ_C (100 MHz) 167.3 (C=O), 159.0 (C–O), 151.2 (C), 136.3 (C), 133.5 (C), 129.6 (CH), 127.5 (2CH), 125.6 (2CH), 118.8 (CH), 118.1 (CH), 113.2 (CH), 70.0 (OCH_2), 39.8 ($NHCH_2$), 34.6 (*C*Me_3), 31.7 (CH_2), 31.3 ($3CH_3$), 20.1 (CH_2) and 13.8 (CH_3); HRMS (ESI^+): found, 362.2081. $C_{22}H_{29}NaNO_2$ (M+Na) requires 362.2096.

N-Butyl-3-((2-methylbenzyl)oxy)benzamide **37c**

Preparation as for **37b** using *N*-butyl-3-hydroxybenzamide **36** (0.96 g, 5.00 mmol), 2-methylbenzyl bromide (0.93 g, 5.00 mmol), and NaH (0.20 g, 5.00 mmol) gave **37c** (1.22 g, 82%) as a yellow oil which was used without further purification; ν_{max}/cm^{-1} 3238, 2955, 1630, 1585, 1545, 1489, 1315, 1246, 1004, 745; δ_H (400 MHz) 7.48 (1 H, dd, *J* 2.6, 1.5, ArH), 7.38−7.32 (1 H, m, ArH), 7.32−7.29 (1 H, m, ArH), 7.28−7.22 (1 H, m, ArH), 7.23−7.15 (3 H, m, ArH), 7.06 (1 H, ddd, *J* 7.8, 2.6, ArH), 6.60 (1 H, t, *J* 5.7, NH), 5.00 (2 H, s, OCH_2), 3.40 (2 H, td, *J* 7.2, 5.7, NHC*H*$_2$), 2.33 (3 H, s, $ArCH_3$), 1.67−1.48 (2 H, $NHCH_2$C*H*$_2$), 1.43−1.26 (2 H, m, C*H*$_2$$CH_3$) and 0.92 (3 H, t, *J* 7.3, CH_2C*H*$_3$); δ_C (100 MHz) 167.3 (C=O), 158.9 (C–O), 136.6 (C), 136.2 (C), 134.3 (C), 130.3 (CH), 129.4 (CH), 128.5 (CH), 128.2 (CH), 125.9 (CH), 118.9 (CH), 118.0 (CH), 113.0 (CH), 68.5 (OCH_2), 39.7 ($NHCH_2$), 31.5 (CH_2), 20.0 (CH_2) 18.7 (CH_3) and 13.7 (CH_3); HRMS (ESI^+): found, 320.1612. $C_{19}H_{23}NaNO_2$ (M+Na) requires 320.1626.

N-Butyl-3-((2,4,6-trimethylbenzyl)oxy)benzamide **37d**

Preparation as for **37b** using *N*-butyl-3-hydroxybenzamide **36** (0.96 g, 5.00 mmol), freshly distilled 2,4,6-trimethylbenzyl chloride (0.93 g, 5.00 mmol), and NaH (0.20 g, 5.00 mmol) in DMF (10 mL) gave, after recrystallisation (PhMe/EtOAc), **37d** (1.18 g, 73%) as colourless crystals, mp 122−125 °C; ν_{max}/cm^{-1} 3287, 2951, 2938, 2870, 1630, 1584, 1553, 1439, 1304, 1234, 1009, 989, 692; δ_H (400 MHz) 7.48 (1 H, dd, *J* 2.7, 1.5, ArH), 7.37−7.26 (2 H, m, ArH), 7.11 (1 H, ddd, *J* 7.7,

2.6, 1.5, ArH), 6.91 (2 H, s, ArH), 6.16 (1 H, br s, NH), 5.05 (2 H, s, OCH_2), 3.46 (2 H, td, *J* 7.1, 5.7, NHC*H*$_2$), 2.35 (6 H, s, 2CH_3), 2.29 (3 H, s, CH_3), 1.66−1.54 (2 H, m, $NHCH_2$C*H*$_2$), 1.49−1.34 (2 H, m, C*H*$_2$$CH_3$) and 0.96 (3 H, t, *J* 7.3, CH_2C*H*$_3$); δ_C (100 MHz) 167.3 (C=O), 159.5 (C–O), 138.4 (C), 138.0 (2C), 136.3 (C), 129.52 (CH), 129.50 (C), 129.1 (2CH), 118.6 (CH), 118.3 (CH), 112.7 (CH), 64.6 (OCH_2), 39.8 ($NHCH_2$), 31.7 (CH_2), 21.0 (CH_3), 20.1 (CH_2), 19.5 (2CH_3) and 13.8 (CH_3); HRMS (ESI$^+$): found, 348.1925. $C_{21}H_{28}NaNO_2$ (M+Na) requires 348.1939.

N-Butyl-3-((2-nitrobenzyl)oxy)benzamide **37e**

Preparation as for **37b** using using *N*-butyl-3-hydroxybenzamide **36** (0.41 g, 2.13 mmol), 1-(bromomethyl)-2-nitrobenzene (0.46 g, 2.13 mmol), and NaH (85 mg, 2.13 mmol) gave, after recrystallisation (PhMe/EtOAc), **37e** (0.34 g, 49%) as a red solid, mp 84−87 °C; ν_{max}/cm^{-1} 3404, 2951, 2916, 2870, 2859, 1638, 1587, 1526, 1341, 1300, 1236, 721, 683; δ_H (300 MHz) 8.18 (1 H, dd, *J* 8.2, 1.4, ArH), 7.87 (1 H, dt, *J* 7.8, 1.1, ArH), 7.69 (1 H, td, *J* 7.6, 1.3, ArH), 7.54−7.46 (1 H, m, ArH), 7.46−7.42 (1 H, m, ArH), 7.39−7.28 (2 H, m, ArH), 7.10 (1 H, ddd, *J* 7.5, 2.6, 1.8, ArH), 6.11 (1 H, s, NH), 5.53 (2 H, s, OCH_2), 3.46 (2 H, td, *J* 7.1, 5.7, NHC*H*$_2$), 1.63−1.57 (2 H, m, NCH_2C*H*$_2$), 1.46−1.36 (2 H, m, C*H*$_2$$CH_3$) and 0.96 (3 H, t, *J* 7.2, CH_2C*H*$_3$); δ_C (100 MHz) 167.1 (C=O), 158.3 (C–O), 147.0 (CNO_2), 136.6 (C), 134.0 (CH), 133.4 (C), 129.8 (CH), 128.6 (CH), 128.5 (CH), 125.0 (CH), 119.4 (CH), 117.8 (CH), 113.7 (CH), 67.0 (OCH_2), 39.8 ($NHCH_2$), 31.7 (CH_2), 20.1 (CH_2) and 13.8 (CH_3); HRMS (ESI$^+$): found, 351.1307. $C_{18}H_{20}NaN_2O_4$ (M+Na) requires 351.1321.

N-Butyl-3-((3-nitrobenzyl)oxy)benzamide **37f**

Preparation as for **37b** using *N*-butyl-3-hydroxybenzamide **36** (0.96 g, 5.00 mmol), 1-(bromomethyl)-3-nitrobenzene (1.08 g, 5.00 mmol), and NaH (0.20 g, 5.00 mmol) gave, after recrystallisation (PhMe/EtOAc), **37f** (1.07 g, 65%) as a colourless solid, mp 87−90 °C; ν_{max}/cm^{-1} 3285, 3076, 2959, 2932, 2872, 1636, 1524, 1352, 1136, 1057, 691; δ_H (400 MHz) 8.32 (1 H, ddd, *J* 2.5, 1.8, 0.7, ArH), 8.19 (1 H, ddd, *J* 8.2, 2.4, 1.1, ArH), 7.76 (1 H, ddt, *J* 7.7, 1.8, 0.8, ArH), 7.57 (1 H, t, *J* 7.9, ArH), 7.48−7.44 (1 H, m, ArH), 7.39−7.29 (2 H, m, ArH), 7.11 (1 H, ddd, *J* 7.3, 2.6, 1.9, ArH), 6.24 (1 H, br s, NH), 5.19 (2 H, s, OCH_2), 3.46 (2 H, td, *J* 7.1, 5.7, NHC*H*$_2$), 1.66−1.54 (2 H, m, $NHCH_2$C*H*$_2$), 1.48−1.33 (2 H, m, C*H*$_2$$CH_3$) and 0.96 (3 H, t, *J* 7.3, CH_3); δ_C (100 MHz) 167.0 (C=O), 158.3 (C–O), 148.4 (CNO_2), 138.8 (C), 136.5 (C), 133.1 (CH), 129.7 (CH), 129.6 (CH), 123.0 (CH), 122.1 (CH), 119.3 (CH), 118.2 (CH), 113.2 (CH), 68.6 (OCH_2), 39.8 ($NHCH_2$), 31.7 (CH_2), 20.1 (CH_2) and 13.8 (CH_3); HRMS (ESI$^+$): found, 329.1491. $C_{18}H_{21}N_2O_4$ (M+H) requires 329.1501.

N-Butyl-3-((2-methoxybenzyl)oxy)benzamide **37g**

Preparation as for **37b** using *N*-butyl-3-hydroxybenzamide **36** (0.96 g, 5.00 mmol), 1-(bromomethyl)-2-methoxybenzene (1.00 g, 5.00 mmol), and NaH (0.20 g, 5.00 mmol) gave, after purification *via* flash column chromatography (Et_2O/hexane 7:3) at R_f 0.42 **37g** (1.11 g, 71%) as a pale-yellow solid which was used without further purification, mp 88−91 °C; ν_{max}/cm^{-1} 3318, 2955, 2932, 1638, 1584, 1533, 1290, 1242, 752; δ_H (400 MHz) 7.48−7.40 (2 H, m, ArH), 7.31−7.23 (3 H, m, ArH), 7.11−7.04 (1 H, m, ArH), 6.96 (1 H, td , *J* 7.5, 1.1, ArH), 6.89 (1 H ,dd *J* 8.2, 1.0, ArH), 6.36 (1 H, t, *J* 5.7, NH), 5.12 (2 H, s, OCH_2), 3.83 (3 H, s, OCH_3), 3.41 (2 H, td, *J* 7.2, 5.7, NHC*H*$_2$), 1.65−1.50 (2 H, m, $NHCH_2$C*H*$_2$), 1.45−1.29 (2 H, m, C*H*$_2$$CH_3$) and 0.93 (3 H, t, *J* 7.3, CH_2C*H*$_3$) ; δ_C (100 MHz) 167.4 (C=O), 159.1 (C–O), 156.9 (C–O), 136.3 (C), 129.5 (CH), 129.1 (CH), 128.7 (CH), 124.9 (C), 120.6 (CH), 118.7 (CH), 118.1 (CH), 113.3 (CH), 110.3 (CH), 65.2 (OCH_2), 55.4 (OCH_3), 39.8 ($NHCH_2$), 31.7 (CH_2), 20.1 (CH_2) and 13.8 (CH_3); HRMS (ESI$^+$):

found, 314.1744. $C_{19}H_{24}NO_3$ (M+H) requires 314.1756.

N-Butyl-3-((4-methoxybenzyl)oxy)benzamide **37h**

Preparation as for **37b** using *N*-butyl-3-hydroxybenzamide **36** (0.96 g, 5.00 mmol), 1-(bromomethyl)-4-methoxybenzene (1.00 g, 5.00 mmol), and NaH (0.20 g, 5.00 mmol) gave, after recrystallisation (PhMe/EtOAc), **37h** (0.68 g, 43%) as colourless crystals, mp 97−100 °C; ν_{max}/cm^{-1} 3264, 2959, 2932, 1628, 1584, 1514, 1238, 1175, 1011; δ_H (400 MHz) 7.42 (1 H, dd, *J* 2.6, 1.5, ArH), 7.38−7.35 (2 H, m, ArH), 7.35−7.30 (1 H, m, ArH), 7.28 (1 H, t, *J* 1.4, ArH), 7.08 (1 H, ddd, *J* 8.0, 2.6, 1.3, ArH), 6.92 (2 H, d, *J* 8.6, ArH), 6.06 (1 H, br s, NH), 5.03 (2 H, s, OCH_2), 3.82 (3 H, s, OCH_3), 3.45 (2 H, td, *J* 7.2, 5.7, NHCH_2), 1.63−1.54 (2 H, m, NHCH$_2$CH_2), 1.47−1.37 (CH_2CH$_3$) and 0.96 (3 H, t, *J* 7.3, CH$_2$CH_3); δ_C (100 MHz) 167.2 (C=O), 159.5 (C–O), 158.9 (C–O), 136.3 (C),129.5 (CH), 129.3 (2CH), 128.5 (C), 118.8 (CH), 118.2 (CH), 114.0 (2CH), 113.2 (CH), 69.9 (OCH_2), 55.3 (OCH_3), 39.8 ($NHCH_2$), 31.7 (CH_2), 20.1 (CH_2) and 13.8 (CH_3); HRMS (ESI$^+$): found, 314.1746. $C_{19}H_{24}NO_3$ (M+H) requires 314.1756.

N-Butyl-3-((2-fluorobenzyl)oxy)benzamide **37i**

To a stirred solution of NaI (0.82 g, 5.5 mmol) in acetone (10 mL) was added 2-fluorobenzyl chloride (0.65 mL, 0.80 g 5.5 mmol) and the solution stirred at rt until no further precipitation was observed. The solution was filtered and the filtrate concentrated to give 2-fluorobenzyl iodide. Separately, to a suspension of sodium hydride (60% dispersion in mineral oil, pre-washed (x 3) with hexane, 0.20 g, 5.00 mmol) in DMF (10 mL) was added *N*-butyl-3-hydroxybenzamide **36** (0.96 g, 5.00 mmol) and the mixture was stirred at rt for 15 min before adding 2-fluorobenzyl iodide prepared as above. After stirring for 18 h at rt, the reaction mixture was poured into water and extracted with CH_2Cl_2 followed by Et_2O (× 3). The combined organic layers were washed with water (× 5) before being dried over $MgSO_4$ and concentrated to give **37i** (1.28 g, 85%) as a colourless solid which was used without further purification, mp 70−73 °C; ν_{max}/cm^{-1} 3296, 2953, 2930, 2868, 1635, 1585, 1531, 1233, 1011, 756, 704, 689; δ_H (400 MHz) 7.50 (1 H, td, *J* 7.5, 1.8, ArH), 7.44 (1 H, dd, *J* 2.8, 1.5, ArH), 7.38−7.29 (3 H, m, ArH), 7.17 (1 H, td, *J* 7.5, 1.2, ArH), 7.13−7.07 (2 H, m, ArH), 6.09 (1 H, s, NH), 5.17 (2 H, s, OCH_2), 3.45 (2 H, td, *J* 7.2, 5.7, NHCH_2), 1.63−1.58 (2 H, m, NHCH$_2$CH_2), 1.49−1.37 (2 H, m, CH_2CH$_3$) and 0.96 (3 H, t, *J* 7.3, CH$_2$CH_3); δ_C (100 MHz) 167.2 (4ry, C=O), 160.5 (d, J_{CF} 247.3, CF), 158.7 (C–O), 136.4 (C), 129.9 (d, J_{CF} 8.2, CH), 129.8 (d, J_{CF} 3.8, CH), 129.6 (CH), 124.3 (d, J_{CF} 3.5, CH), 123.7 (d, J_{CF} 14.2, C), 119.1 (CH), 118.0 (CH), 115.4 (d, J_{CF} 21.1, CH), 113.3 (CH), 63.9 (d, J_{CF} 4.3, OCH_2), 39.8 ($NHCH_2$), 31.7 (CH_2), 20.1 (CH_2) and 13.8 (CH_3); δ_F (376 MHz) −118.5; HRMS (ESI$^+$): found, 324.1364**.** $C_{18}H_{20}FNaNO_2$ (M+Na) requires 324.1376.

N-Butyl-3-((4-fluorobenzyl)oxy)benzamide **37j**

Preparation as for **37i** using NaI (0.82 g, 5.5 mmol) and 4-fluorobenzyl chloride (0.65 mL, 0.80 g 5.5 mmol) in acetone (10 mL) and *N*-butyl-3-hydroxybenzamide **36** (0.96 g, 5.00 mmol) and NaH (0.20 g, 5.00 mmol) in DMF (10 mL) at rt for 18 h gave **37j** (1.31 g, 87%) as a colourless solid which was used without further purification, mp 66−69 °C; ν_{max}/cm^{-1} 3285, 2955, 2930, 2918, 2868, 1634, 1541, 1512, 1308, 1013, 715; δ_H (400 MHz) 7.46−7.37 (3 H, m, ArH), 7.35−7.25 (2 H, m, ArH), 7.12−7.02 (3 H, m, ArH), 6.22 (1 H, br s, NH), 5.04 (2 H, s, OCH_2), 3.44 (2 H, td, *J* 7.1, 5.7, NHCH_2), 1.66−1.52 (2 H, m, NHCH$_2$CH_2), 1.48−1.33 (2 H, m, CH_2CH$_3$) and 0.95 (3 H, t, *J* 7.3, CH$_2$C$\boldsymbol{H_3}$); δ_C (100 MHz) 167.2 (C=O), 162.5 (d, J_{CF} 246.5, CF), 158.7 (C–O), 136.4 (C), 132.3 (C), 129.6 (CH), 129.4 (d, J_{CF} 8.2, 2CH), 118.9 (CH), 118.2 (CH), 115.5 (d, *J* 21.5, 2CH), 113.2 (CH), 69.4 (OCH_2), 39.8 ($NHCH_2$), 31.7 (CH_2), 20.1 (CH_2) and 13.8 (CH_3); δ_F (376 MHz)

−114.0; HRMS (ESI$^+$): found, 324.1365. $C_{18}H_{20}FNaNO_2$ (M+Na) requires 324.1376.

N-Butyl-3-(1-phenylethoxy)benzamide **37k**
Preparation as for **37b** using *N*-butyl-3-hydroxybenzamide **36** (0.96 g, 5.00 mmol), 1-(bromoethyl)benzene (0.71 mL, 0.96 g, 5.00 mmol), and NaH (0.20 g, 5.00 mmol) gave **37k** (1.29 g, 87%) as a slightly yellow oil which was used without further purification; ν_{max}/cm^{-1} 3316, 2957, 2930, 2872, 1634, 1578, 1541, 1302, 1287, 1234, 1069, 752, 698; δ_H (400 MHz) 7.39−7.35 (2 H, m, ArH), 7.35−7.29 (3 H, m, ArH), 7.27−7.24 (1 H, m, ArH), 7.23−7.19 (2 H, m, ArH), 6.99−6.91 (1 H, m, ArH), 6.02 (1 H, br s, NH), 5.36 (1 H, q, *J* 6.4, OC*H*CH$_3$), 3.40 (2 H, tdd, *J* 7.1, 5.8, 0.8, NHC*H*$_2$), 1.64 (3 H, d, *J* 6.4, OCHC*H*$_3$), 1.60−1.50 (NHCH$_2$C*H*$_2$), 1.44−1.34 (C*H*$_2$CH$_3$) and 0.94 (3 H, t, *J* 7.3, CH$_2$C*H*$_3$); δ_C (100 MHz) 167.3 (C=O), 158.0 (C–O), 142.6 (C), 136.2 (C), 129.4 (CH), 128.6 (2CH), 127.6 (CH), 125.6 (2CH), 118.8 (CH), 118.7 (CH), 114.6 (CH), 76.1 (OCH), 39.7 (NHCH$_2$), 31.6 (CH$_2$), 24.3 (CH$_3$), 20.1 (CH$_2$) and 13.8 (CH$_3$); HRMS (ESI$^+$): found, 320.1613. $C_{19}H_{23}NaNO_2$ (M+Na) requires 320.1626.

N-Butyl-3-((3-methylbut-2-en-1-yl)oxy)benzamide **37l**
Preparation as for **37b** using *N*-butyl-3-hydroxybenzamide **36** (0.96 g, 5.00 mmol), 1-bromo-3-methylbut-2-ene (0.75 g, 5.00 mmol), and NaH (0.20 g, 5.00 mmol) in DMF (10 mL) gave **37l** (1.09 g, 89%) as a colourless oil which was used without further purification; ν_{max}/cm^{-1} 3296, 2957, 2930, 2870, 1634, 1582, 1537, 1317, 1306, 1240, 1007, 988, 689; δ_H (400 MHz) 7.40−7.36 (1 H, m, ArH), 7.32−7.26 (2 H, m, ArH), 7.04 (1 H, dt, *J* 7.0, 2.5, ArH), 6.30 (1 H, br s, NH), 5.48 (1 H, tdq, *J* 6.8, 2.9, 1.4, =C*H*), 4.53 (2 H, d, *J* 6.8, OCH$_2$), 3.43 (2 H, td, *J* 7.1, 5.7, NHC*H*$_2$), 1.79 (3 H, s, C*Me*$_2$), 1.74 (3 H, s, C*Me*$_2$), 1.65−1.52 (2 H, m, NHCH$_2$C*H*$_2$), 1.47−1.34 (2 H, m, C*H*$_2$CH$_3$) and 0.94 (3 H, t, *J* 7.3, CH$_2$C*H*$_3$); δ_C (100 MHz) 167.4 (C=O), 159.0 (C–O), 138.5 (C), 136.2 (C), 129.4 (CH), 119.2 (CH), 118.5 (CH), 118.1 (CH), 112.9 (CH), 64.9 (OCH$_2$), 39.8 (NHCH$_2$), 31.6 (CH$_2$), 25.8 (CH$_3$), 20.1 (CH$_2$), 18.1 (CH$_3$) and 13.7 (CH$_3$); HRMS (ESI$^+$): found, 284.1614. $C_{16}H_{23}NaNO_2$ (M+Na) requires 284.1626.

N-Butyl-3-(thiophen-2-ylmethoxy)benzamide **37m**
Preparation as for **37b** using *N*-butyl-3-hydroxybenzamide **36** (0.96 g, 5.00 mmol), 2-(bromomethyl)thiophene (0.89 g, 5.00 mmol), and NaH (0.20 g, 5.00 mmol) in DMF (10 mL) gave, after recrystallisation (PhMe/EtOAc), **37m** (0.86 g, 59%) as colourless crystals, mp 88−90 °C; ν_{max}/cm^{-1} 3275, 2953, 2934, 2872, 1624, 1584, 1541, 1236, 1009, 712, 691; δ_H (400 MHz) 7.44 (1 H, dd, *J* 2.7, 1.6, ArH), 7.33 (1 H, dt, *J* 7.7, 1.3, ArH), 7.21−7.13 (2 H, m, ArH), 7.06−7.03 (2 H, m, ArH), 6.87 (1 H, dd, *J* 5.1, 3.5, ArH), 6.75 (1 H, t, *J* 5.7, NH), 5.15 (2 H, s, OCH$_2$), 3.39 (2 H, td, *J* 7.2, 5.7, NHC*H*$_2$), 1.62−1.49 (2 H, m, NHCH$_2$C*H*$_2$), 1.43−1.28 (2 H, m, C*H*$_2$CH$_3$) and 0.91 (3 H, t, *J* 7.3, CH$_2$C*H*$_3$); δ_C (100 MHz) 167.1 (C=O), 158.2 (C–O), 138.6 (C), 136.1 (C), 129.3 (CH), 126.9 (CH), 126.6 (CH), 126.2 (CH), 119.3 (CH), 118.1 (CH), 113.1 (CH), 64.8 (OCH$_2$), 39.7 (NHCH$_2$), 31.5 (CH$_2$), 20.0 (CH$_2$) and 13.6 (CH$_3$); HRMS (ESI$^+$): found, 312.1020. $C_{16}H_{20}NaNO_2S$ (M+Na) requires 312.1034.

N-Butyl-3-((2-methoxynaphthalen-1-yl)methoxy)benzamide **37n**
Preparation as for **37b** using *N*-butyl-3-hydroxybenzamide **36** (0.96 g, 5.00 mmol), 1-(bromomethyl)-2-methoxynaphthalene (1.26 g, 5.00 mmol), and NaH (0.20 g, 5.00 mmol) gave, after recrystallisation (PhMe/EtOAc), **37n** (0.65 g, 36%) as a colourless solid, mp 123−126 °C; ν_{max}/cm^{-1} 3250, 3069, 2955, 2932, 2872, 1634, 1555, 1470, 1315, 1269, 1236, 1003, 984, 812, 717; δ_H (400 MHz) 7.97 (1 H, dt, *J* 8.7, 1.0, ArH), 7.81 (1 H, d, *J* 9.1, ArH), 7.78−7.72 (1 H, m,

ArH), 7.56 (1 H, dd, *J* 2.6, 1.5 ,ArH), 7.45 (1 H, ddd, *J* 8.5, 6.8, 1.4, ArH), 7.35−7.28 (2 H, m, ArH), 7.27−7.20 (2 H, m, ArH), 7.11 (1 H, ddd, *J* 7.9, 2.6, 1.3, ArH), 6.38 (1 H, t, *J* 5.2, NH), 5.55 (2 H, s, OCH_2), 3.91 (3 H, s, OCH_3), 3.37 (2 H, td, *J* 7.2, 5.7, NHC*H*$_2$), 1.57−1.46 (2 H, m, $NHCH_2CH_2$), 1.42−1.26 (CH_2CH_3) and 0.90 (3 H, t, *J* 7.3, CH_2CH_3); δ_C (100 MHz) 167.3 (C=O), 159.0 (C–O), 155.4 (C–O), 136.1 (C), 133.5 (C), 130.9 (CH), 129.3 (CH), 129.0 (C), 128.2 (CH), 127.0 (CH), 123.6 (CH), 123.4 (CH), 118.8 (CH), 118.1 (CH), 116.4 (C), 113.1 (CH), 113.0 (CH), 60.7 (OCH_2), 56.6 (OCH_3), 39.7 ($NHCH_2$), 31.6 (CH_2), 20.0 (CH_2) and 13.7 (CH_3); HRMS (ESI^+): found, 364.1901. $C_{23}H_{26}NO_3$ (M+H) requires 364.1913

N-Butyl-3-(hydroxy(phenyl)methyl)benzamide **38a**

Under a nitrogen atmosphere, *n*-butyllithium (2.5 M in hexane, 2.64 mL, 6.60 mmol) was added dropwise to a stirred solution of 3-(benzyloxy)-*N*-butylbenzamide **37a** (567 mg, 2.0 mmol) in dry THF (20 mL). After stirring at rt for 2 h, the reaction mixture was quenched by addition of sat. aq. NH_4Cl and extracted with Et_2O (× 3). The combined organic layers were dried and evaporated. Purification of the residue by column chromatography (gradient elution, Et_2O/hexane 1:1 to Et_2O/hexane 7:3) gave **38a** (298 mg, 53%) as colourless crystals, mp 97−100 °C; ν_{max}/cm^{-1} 3298, 3229, 2961, 2932, 2857, 1626, 1553, 1418, 1327, 1016, 746, 698; δ_H (400 MHz) 7.75 (1 H, t, *J* 1.8, ArH), 7.57 (1 H, dt, *J* 7.7, 1.5, ArH), 7.42−7.35 (1 H, m, ArH), 7.31−7.24 (5 H, m, ArH), 7.24−7.21 (1 H, m, ArH) 6.43 (1 H, t, *J* 5.7, NH), 5.74 (1 H, s, C*H*OH), 3.71 (1 H, s, CHO*H*), 3.31 (2 H, td, *J* 7.2, 5.7, NHC*H*$_2$), 1.54−1.45 (2 H, m, $NHCH_2CH_2$), 1.39−1.27 (2 H, m, CH_2CH_3) and 0.90 (3 H, t, *J* 7.3, CH_2CH_3); δ_C (100 MHz) 167.6 (C=O), 144.5 (C), 143.5 (C), 134.7 (C), 129.5 (CH), 128.5 (CH), 128.4 (2CH), 127.5 (CH), 126.5 (2CH), 125.9 (CH), 124.7 (CH), 75.6 (CHOH), 39.8 ($NHCH_2$), 31.5 (CH_2), 20.1 (CH_2) and 13.7 (CH_3); HRMS (ESI^+): found, 284.1637. $C_{18}H_{22}NO_2$ (M+H) requires 284.1651.

For X-ray structural data of **38a** see page S191

N-Butyl-3-((4-(*tert*-butyl)phenyl)(hydroxy)methyl)benzamide **38b**

Reaction as for **37a** using *N*-butyl-3-((4-(*tert*-butyl)benzyl)oxy)benzamide **37b** (0.679 g, 2.0 mmol), and *n*-butyllithium (2.64 mL, 6.60 mmol) in THF (20 mL) gave, after purification *via* flash column chromatography (hexane/Et_2O 1:1) at R_f 0.10, **38b** (250.5 mg, 37%) as a colourless solid, mp 120−123 °C; ν_{max}/cm^{-1} 3269, 2959, 2930, 2870, 1630, 1545, 1485, 1308, 1242, 831, 731, 687, 569; δ_H (400 MHz) 7.83−7.78 (1 H, m, ArH), 7.64 (1 H, dt, *J* 7.7, 1.6, ArH), 7.50 (1 H, dddd, *J* 7.7, 1.9, 1.3, 0.6, ArH), 7.41−7.32 (2 H, m, ArH), 7.30−7.27 (1 H, m, ArH), 7.27−7.25 (1 H, m, ArH), 6.15 (1 H, s, NH), 5.85 (1 H, br s, C*H*OH), 3.43 (2 H, td, *J* 7.2, 5.7, NHC*H*$_2$), 2.50 (1 H, d, *J* 3.0, OH), 1.62−1.51 (2 H, m, $NHCH_2CH_2$), 1.46−1.34 (2 H, m, CH_2CH_3), 1.30 (9 H, s, CMe_3) and 0.95 (3 H, t, *J* 7.3, CH_2CH_3); δ_C (100 MHz) 167.5 (C=O), 150.8 (C–O), 144.4 (C), 140.5 (C), 134.9 (C), 129.4 (CH), 128.6 (CH), 126.4 (2CH), 125.9 (CH), 125.6 (2CH), 124.6 (CH), 75.7 (CHOH), 39.8 ($NHCH_2$), 31.7 (CH_2), 31.3 (C*Me*$_3$), 22.6 (*C*Me$_3$), 20.1 (CH_2) and 13.8 (CH_3); HRMS (ESI^+): found, 340.2264. $C_{22}H_{30}NO_2$ (M+H) requires 340.2277.

N-Butyl-3-(hydroxy(*o*-tolyl)methyl)benzamide **38c**

Reaction as for **37a** using *N*-butyl-3-((2-methylbenzyl)oxy)benzamide **37c** (595 mg, 2.00 mmol), and *n*-butyllithium (2.64 mL, 6.60 mmol) in THF (20 mL) gave, after purification *via* flash column chromatography (hexane/Et_2O 7:3) at R_f 0.23, **38c** (170 mg, 29%) as a colourless oil; ν_{max}/cm^{-1} 3325, 2957, 2930, 2872, 1636, 1540, 1302, 1028, 908, 727; δ_H (400 MHz) 7.78−7.75 (1 H, m, ArH), 7.63 (1 H, dt, *J* 6.8, 1.9, ArH), 7.45−7.37 (1 H, m, ArH), 7.37−7.30 (2 H, m, ArH), 7.22−7.17

(2 H, m, ArH), 7.16−7.10 (1 H, m, ArH), 6.22 (1 H, br s, NH), 6.00 (1 H, s, C*H*OH), 3.39 (2 H, td, *J* 7.2, 5.7, NHCH_2), 2.81 (1 H, br s, OH), 2.24 (3 H, s, CH_3), 1.61−1.50 (2 H, m, $NHCH_2CH_2$), 1.44−1.31 (2 H, m, CH_2CH_3) and 0.94 (3 H, t, *J* 7.3, CH_2CH_3); δ_C (100 MHz) 167.5 (C=O), 143.5 (C), 141.0 (C), 135.4 (C), 134.9 (C), 130.6 (CH), 129.9 (CH), 128.6 (CH), 127.7 (CH), 126.5 (CH), 126.2 (CH), 125.9 (CH), 125.4 (CH), 72.9 (C*H*OH), 39.8 ($NHCH_2$), 31.6 (CH_2), 20.1 (CH_2), 19.4 (CH_3) and 13.8 (CH_3); HRMS (ESI^+): found, 298.1794. $C_{19}H_{24}NO_2$ (M+H) requires 298.1807.

N-Butyl-3-(hydroxy(2-methoxyphenyl)methyl)benzamide **38g**
Reaction as for **37a** using *N*-butyl-3-((2-methoxybenzyl)oxy)benzamide **37g** (0.627 g, 2.00 mmol), and *n*-butyllithium (2.64 mL, 6.60 mmol) in THF (20 mL) gave, after purification *via* flash column chromatography (gradient elution, Et_2O/hexane 1:1 to Et_2O/hexane 7:3), **38g** (300 mg, 48%) as a yellow oil; ν_{max}/cm^{-1} 3337, 2949, 2932, 1636, 1535, 1489, 1240, 1028, 908, 752, 729, 700; δ_H (400 MHz) 7.83−7.78 (1 H, m, ArH), 7.64 (1 H, dt, *J* 7.6, 1.6, ArH), 7.49−7.40 (1 H, m, ArH), 7.32 (1 H, t, *J* 7.7, ArH), 7.25−7.17 (2 H, m, ArH), 6.92 (1 H, td, *J* 7.5, 1.1, ArH), 6.87 (1 H, dd, *J* 8.2, 1.0, ArH), 6.31 (1 H, s, NH), 6.07 (1 H, s, C*H*OH), 3.78 (3 H, s, OCH_3), 3.39 (2 H, td, *J* 7.2, 5.7, NHCH_2), 1.61−1.49 (2 H, m, $NHCH_2CH_2$), 1.42−1.32 (2 H, m, CH_2CH_3) and 0.93 (3 H, t, *J* 7.3, CH_2CH_3); δ_C (100 MHz) 167.6 (C=O), 156.5 (C–O), 143.8 (C), 134.7 (C), 131.5 (C), 129.4 (CH), 128.8 (CH), 128.3 (CH), 127.7 (CH), 125.8 (CH), 124.7 (CH), 120.8 (CH), 110.6 (CH), 71.5 (CHOH), 55.3 (OCH_3), 39.7 ($NHCH_2$), 31.6 (CH_2), 20.1 (CH_2) and 13.7 (CH_3); HRMS (ESI^+): found, 314.1747. $C_{19}H_{24}NO_3$ (M+H) requires 314.1756.

N-Butyl-3-(hydroxy(4-methoxyphenyl)methyl)benzamide **38h**
Reaction as for **37a** using *N*-butyl-3-((4-methoxybenzyl)oxy)benzamide **37h** (627 mg, 2.00 mmol), and *n*-butyllithium (2.64 mL, 6.60 mmol) in THF (20 mL) gave, after purification *via* flash column chromatography (hexane/Et_2O 7:3) at R_f 0.13, **38h** (270 mg, 43%) as an colourless solid, mp 83−86 °C; ν_{max}/cm^{-1} 3289, 3217, 2959, 2932, 2870, 1624, 1580, 1555, 1514, 1246, 1175, 1026, 847, 806, 696; δ_H (400 MHz) 7.80−7.73 (1 H, m, ArH), 7.62 (1 H, dt, *J* 7.6, 1.6, ArH), 7.47−7.41 (1 H, m, ArH), 7.39−7.28 (1 H, m, ArH), 7.23 (2 H, d, *J* 8.8, ArH), 6.83 (2 H, d, *J* 8.8, ArH), 6.25 (1 H, br s, NH), 5.78 (1 H, d, *J* 3.0, C*H*OH), 3.77 (3 H, s, OCH_3), 3.39 (2 H, td, *J* 7.2, 5.7, NHCH_2), 2.90 (1 H, d, *J* 3.0, OH), 1.61−1.47 (2 H, m, NCH_2CH_2), 1.42−1.30 (2 H, m, CH_2CH_3) and 0.93 (3 H, t, *J* 7.3, CH_2CH_3); δ_C (100 MHz) 167.5 (C=O), 159.1 (C–O), 144.6 (C), 135.8 (C), 134.8 (C), 129.3 (CH), 128.6 (CH), 128.0 (2CH), 125.8 (CH), 124.6 (CH), 113.9 (2CH), 75.3 (CHOH), 55.2 (OCH_3), 39.8 ($NHCH_2$), 31.6 (CH_2), 20.1 (CH_2) and 13.8 (CH_3); HRMS (ESI^+): found, 314.1744. $C_{19}H_{24}NO_3$ (M+H) requires 314.1756.

N-Butyl-3-((4-fluorophenyl)(hydroxy)methyl)benzamide **38j**
Reaction as for **37a** using *N*-butyl-3-((4-fluorophenyl)(hydroxy)methyl)benzamide **37j** (603 mg, 2.00 mmol), and *n*-butyllithium (2.64 mL, 6.60 mmol) in THF (20 mL) gave, after purification *via* flash column chromatography (gradient elution Et_2O/hexane 1:1 to Et_2O/hexane 7:3), **38j** (60 mg, 10%) as an orange solid, mp 75−88 °C; ν_{max}/cm^{-1} 3304, 3233, 2963, 2930, 2859, 1626, 1603, 1580, 1553, 1508, 1229, 1024, 1015, 853, 696, 563; δ_H (400 MHz) 7.76 (1 H, d, *J* 1.9, ArH), 7.61 (1 H, dt, *J* 7.5, 1.6, ArH), 7.43 (1 H, dt, *J* 7.7, 1.7, ArH), 7.38−7.33 (1 H, m, ArH), 7.32−7.27 (2 H, m, ArH), 7.02−6.95 (2 H, m, ArH), 6.24 (1 H, br s, NH), 5.80 (1 H, s, C*H*OH), 3.39 (2 H, td, *J* 7.2, 5.7, NHCH_2), 1.61−1.50 ($NHCH_2CH_2$), 1.44−1.31 (CH_2CH_3) and 0.93 (3 H, t, *J* 7.3, CH_2CH_3); δ_C (100 MHz) 167.5 (C=O), 162.1 (d, J_{CF} 246.1, CF) 144.4 (C), 139.3 (d, J_{CF} 3.1, C), 134.9 (C), 129.4 (CH), 128.7 (CH), 128.3 (d, J_{CF} 8.1, 2CH) 125.9 (CH), 124.8 (CH), 115.3 (d, J_{CF} 21.4, 2CH) 75.0 (CHOH), 39.8 ($NHCH_2$), 31.6 (CH_2), 20.1 (CH_2) and 13.8 (CH_3); δ_F (376 MHz) −114.5; HRMS

(ESI$^+$): found, 302.1548. $C_{18}H_{21}FNO_2$ (M+H) requires 302.1556.

N-Butyl-3-(hydroxy(thiophen-2-yl)methyl)benzamide **38m**

Reaction as for **37a** using *N*-butyl-3-(thiophen-2-ylmethoxy)benzamide **37m** (579 mg, 2.00 mmol), and *n*-butyllithium (2.64 mL, 6.60 mmol) in THF (20 mL) gave, after purification *via* flash column chromatography (gradient elution Et_2O/hexane 1:1 to Et_2O/hexane 7:3), **38m** (80 mg, 14%) as a brown oil; ν_{max}/cm^{-1} 3316, 2957, 2932, 2872, 1636, 1537, 1302, 1036, 1022, 908, 729, 696; δ_H (400 MHz) 7.85−7.79 (1 H, m, ArH), 7.68 (1 H, dt, *J* 7.8, 1.5, ArH), 7.57−7.52 (1 H, m, ArH), 7.40 (1 H, t, *J* 7.7, ArH), 7.29−7.24 (1 H, m, ArH), 6.93 (1 H, dd, *J* 5.0, 3.5, ArH), 6.90−6.82 (1 H, m, ArH), 6.21 (1 H, br s, NH), 6.07 (1 H, s, C*H*OH), 3.42 (2 H, td, *J* 7.2, 5.7, NHC*H*$_2$), 1.63−1.52 (2 H, m, NHCH$_2$C*H*$_2$), 1.46−1.35 (2 H, m, C*H*$_2$CH$_3$) and 0.95 (3 H, t, *J* 7.3, CH$_2$C*H*$_3$); δ_C (100 MHz) 167.5 (C=O), 147.7 (C), 143.8 (C), 134.8 (C), 129.3 (CH), 128.7 (CH), 126.6 (CH), 126.4 (CH), 125.5 (CH), 125.0 (CH), 124.5 (CH), 71.8 (CHOH), 39.9 ($NHCH_2$), 31.6 (CH_2), 20.1 (CH_2) and 13.8 (CH_3); HRMS (ESI$^+$): found, 290.1207. $C_{16}H_{21}NO_2S$ (M+H) requires 290.1215.

N-Butyl-2-hydroxy-5-nitrobenzamide **39**

A mixture of methyl 2-hydroxy-5-nitrobenzoate[46] (4.50 g, 19.8 mmol) and *n*-butylamine (50 mL, 37.00 g, 0.506 mol) was heated at reflux for 3 d before being concentrated *in vacuo*. The residue was dissolved in CH_2Cl_2 (40 mL) and washed successively with 2 M HCl (40 mL), water (40 mL) and sat. aq. Na_2CO_3 (40 mL) before being dried and evaporated. The residue was recrystallised (PhMe) to give **39** (4.16 g, 78%) as pale orange needles, mp 111–113 °C; ν_{max}/cm^{-1} 3408, 2959, 2870, 1647, 1597, 1479, 1335, 1290, 1150, 912, 847, 748 and 638; δ_H (500 MHz) 8.45 (1 H, d, *J* 2.5, ArH), 8.28 (1 H, dd, *J* 9.0, 2.5, ArH), 7.07 (1 H, d, *J* 9.0, ArH), 6.73 (1 H, br s, NH), 3.51 (2 H, td, *J* 7.3, 5.5, NCH$_2$), 1.70–1.64 (2 H, m, NCH$_2$C*H*$_2$), 1.48–1.41 (2 H, m, C*H*$_2$CH$_3$) and 0.99 (3 H, t, *J* 7.5, CH$_3$); δ_C (125 MHz) 168.4 (C), 167.3 (C), 139.1 (CNO_2), 129.2 (CH), 122.3 (CH), 119.4 (CH), 113.7 (C), 39.8 (NCH_2), 31.3 (CH_2), 20.1 (CH_2) and 13.7 (CH_3); HRMS (NSI$^+$): found, 261.0848. $C_{11}H_{14}NaN_2O_4$ (M+Na) requires 261.0846.

The requisite phenol (1.0 eq.) was added to a stirred suspension of sodium hydride (60% in mineral oil, pre-washed with hexane, 1.0 eq.) in DMF and the mixture was stirred at rt for 15 min before the alkyl halide (1.0 eq.) was added. After stirring for 18 h at rt, the reaction mixture was poured into water and extracted with CH_2Cl_2 followed by Et_2O (× 3). The combined organic layers were washed with brine (× 5) and 2 M NaOH before being dried and evaporated.

2-(Benzyloxy)-*N*-butyl-5-nitrobenzamide **40a**

N-Butyl-2-hydroxy-5-nitrobenzamide **39** (1.19 g, 4.99 mmol) was added to a stirred suspension of sodium hydride (60% in mineral oil, pre-washed with hexane, 0.20 g, 5.00 mmol) in DMF (10 mL) and the mixture was stirred at rt for 15 min before benzyl bromide (0.60 mL, 0.86 g, 5.04 mmol) was added. After stirring for 18 h at rt, the reaction mixture was poured into water and extracted with CH_2Cl_2 followed by Et_2O (× 3). The combined organic layers were washed with brine (× 5) and 2 M NaOH before being dried and evaporated. Recrystallisation of the residue (EtOAc/hexane) gave **40a** (0.98 g, 60%) as colourless crystals, mp 148–150 °C; (lit.[47] 145–146 °C); ν_{max}/cm^{-1} 3408, 2961, 1655, 1514, 1339, 1275, 1234, 982, 935, 820, 746, 710 and 687; δ_H (500 MHz) 9.12 (1 H, d, *J* 3.0, ArH), 8.32 (1 H, dd, *J* 9.0, 3.0, ArH), 7.65 (1 H, br s, NH), 7.48–7.44 (5 H, m, Ph), 7.18 (1 H, d, *J* 9.0, ArH), 5.27 (2 H, s, OCH$_2$), 3.35 (2 H, td, *J* 7.0, 5.3, NCH$_2$),

1.35–1.29 (2 H, m, NCH_2CH_2), 1.18–1.10 (2 H, m, CH_2CH_3) and 0.80 (3 H, t, *J* 7.3, CH_3); δ_C (125 MHz) 162.6 (C), 160.8 (C), 142.0 (CNO_2), 134.0 (C), 129.4 (CH), 129.1 (2CH), 128.5 (CH), 128.3 (2CH), 127.8 (CH), 122.9 (C), 112.7 (CH), 72.4 (OCH_2), 39.7 (NCH_2), 31.0 (CH_2), 19.9 (CH_2) and 13.7 (CH_3); HRMS ($ASAP^+$): found, 657.2913. $C_{36}H_{41}N_4O_8$ (2M+H) requires 657.2919.

N-Butyl-5-nitro-2-((4-nitrobenzyl)oxy)benzamide **40b**

Preparation as for **40a** using *N*-butyl-2-hydroxy-5-nitrobenzamide **39** (1.19 g, 4.99 mmol), sodium hydride (0.20 g, 5.00 mmol) and 4-nitrobenzyl bromide (1.08 g, 5.04 mmol) in DMF (10 mL) gave, after recrystallisation (EtOAc/hexane), **40b** (1.16 g, 62%) as colourless crystals, mp 128–130 °C; ν_{max}/cm^{-1} 3306, 2928, 1638, 1518, 1339, 1263, 1088, 1036, 843, 737 and 637; δ_H (500 MHz) 9.01 (1 H, d, *J* 3.0, ArH), 8.33 (2 H, d, *J* 8.8, ArH), 8.29 (1 H, dd, *J* 9.0, 3.0, ArH), 7.68 (2 H, d, *J* 8.8, ArH), 7.35 (1 H, br s, NH), 7.12 (1 H, d, *J* 9.0, ArH), 5.40 (2 H, s, OCH_2), 3.40 (2 H, td, *J* 7.0, 5.5, NCH_2), 1.44–1.38 (2 H, m, NCH_2CH_2), 1.25–1.17 (2 H, m, CH_2CH_3) and 0.84 (3 H, t, *J* 7.5, CH_3); δ_C (125 MHz) 162.6 (C=O), 159.9 (C–O), 148.3 (CNO_2), 142.3 (C), 141.1 (C), 128.6 (2CH), 128.3 (CH), 127.7 (CH), 124.3 (2CH), 123.6 (C), 112.7 (CH), 70.7 (OCH_2), 39.8 (NCH_2), 31.3 (CH_2), 20.0 (CH_2) and 13.7 (CH_3); HRMS ($ASAP^+$): found, 374.1346. $C_{18}H_{20}N_3O_6$ (M+H) requires 374.1347.

N-Butyl-2-((4-methoxybenzyl)oxy)-5-nitrobenzamide **40c**

Preparation as for **40a** using *N*-butyl-2-hydroxy-5-nitrobenzamide **39** (1.19 g, 4.99 mmol), sodium hydride (0.20 g, 5.00 mmol) and 4-methoxybenzyl bromide (1.01 g, 5.02 mmol) in DMF (10 mL) gave, after recrystallisation (EtOAc/hexane), **40c** (1.03 g, 58%) as a pale yellow solid, mp 133–135 °C; ν_{max}/cm^{-1} 3399, 2957, 1649, 1611, 1512, 1344, 1234, 1030, 968, 810, 748 and 685; δ_H (500 MHz) 9.12 (1 H, d, *J* 3.0, ArH), 8.32 (1 H, dd, *J* 9.0, 3.0, ArH), 7.67 (1 H, br s,NH), 7.39 (2 H, d, *J* 8.5, ArH), 7.17 (1 H, d, *J* 9.0, ArH), 6.98 (2 H, d, *J* 8.5, ArH), 5.19 (2 H, s, OCH_2), 3.85 (3 H, s, OCH_3), 3.34 (2 H, td, *J* 7.0, 5.5, NCH_2), 1.34–1.28 (2 H, m, NCH_2CH_2), 1.15–1.07 (2 H, m, CH_2CH_3) and 0.80 (3 H, t, *J* 7.3, CH_3); δ_C (125 MHz) 162.6 (C=O), 160.9 (C–O), 160.4 (C–O), 141.9 (CNO_2), 130.2 (2CH), 128.5 (CH), 127.8 (CH), 126.0 (C), 122.8 (C), 114.4 (2CH), 112.7 (CH), 72.2 (OCH_2), 55.3 (OCH_3), 39.7 (NCH_2), 31.0 (CH_2), 20.0 (CH_2) and 13.7 (CH_3); HRMS ($ASAP^+$): found, 359.1615. $C_{19}H_{23}N_2O_5$ (M+H) requires 359.1601.

Methyl 5-(dimethylamino)-2-hydroxybenzoate **E** and *N*-Butyl-5-(dimethylamino)-2-hydroxybenzamide **41**

Paraformaldehyde (5.18 g, 0.172 mol) and sodium cyanoborohydride (3.46 g, 55.1 mmol) were added to a stirred solution of methyl 5-amino-2-hydroxybenzoate[48] (2.87 g, 17.2 mmol) in acetic acid (170 mL) and the reaction mixture was stirred at rt for 20 h before being concentrated *in vacuo*. The residue was neutralised by addition of sat. aq. Na_2CO_3 before being extracted with CH_2Cl_2 (3 × 30 mL). The combined organic layers were dried and evaporated to give methyl 5-(dimethylamino)-2-hydroxybenzoate **E** as a yellow oil which was used without further purification; ν_{max}/cm^{-1} 3227, 2953, 1682, 1614, 1501, 1441, 1356, 1296, 1211, 1084, 876 and 824; δ_H (500 MHz) 10.22 (1 H, s, OH), 7.18 (1 H, d, *J* 3.0, ArH), 7.04 (1 H, dd, *J* 9.0, 3.0, ArH), 6.91 (1 H, d, *J* 9.0, ArH), 3.94 (3 H, s, OCH_3) and 2.87 (6 H, s, NCH_3); δ_C (125 MHz) 170.6 (C=O), 154.1 (C–O), 144.1 (CN), 123.2 (CH), 118.0 (CH), 113.0 (CH), 112.0 (C), 52.2 (OCH_3) and 41.8 ($2NCH_3$); HRMS (NSI^+): found, 196.0963. $C_{10}H_{14}NO_3$ (M+H) requires 196.0968.

A mixture of methyl 5-(dimethylamino)-2-hydroxybenzoate **E** (assuming 14.6 mmol) and *n*-butylamine (50 mL, 37.00 g, 0.506 mol) was heated at reflux for 3 d. After cooling to rt, the

reaction mixture was concentrated *in vacuo* and the crude residue was purified by filtration through a silica plug (CH_2Cl_2) to give **41** (2.83 g, 70%) as a dark brown oil; ν_{max}/cm^{-1} 3331, 2957, 1638, 1584, 1541, 1499, 1352, 1302, 1219 and 818; δ_H (500 MHz) 11.67 (1 H, br s, OH), 6.97 (1 H, dd, *J* 9.0, 3.0, ArH), 6.91 (1 H, d, *J* 9.0, ArH), 6.65 (1 H, d, *J* 3.0, ArH), 6.36 (1 H, br s, NH), 3.45 (2 H, td, *J* 7.3, 5.5, NCH_2), 2.87 (6 H, s, NCH_3), 1.65–1.59 (2 H, m, NCH_2CH_2), 1.45–1.37 (2 H, m, CH_2CH_3) and 0.96 (3 H, t, *J* 7.3, CH_3); δ_C (125 MHz) 170.1 (C=O), 153.7 (C–O), 144.0 (CN), 121.6 (CH), 119.0 (CH), 114.3 (C), 109.3 (CH), 42.0 ($2NCH_3$), 39.5 (NCH_2), 31.6 (CH_2), 20.1 (CH_2) and 13.8 (CH_3); HRMS (NSI^+): found, 237.1598. $C_{13}H_{21}N_2O_2$ (M+H) requires 237.1598.

2-(Benzyloxy)-*N*-butyl-5-(dimethylamino)benzamide **42a**

Preparation as for **40a** using *N*-butyl-5-(dimethylamino)-2-hydroxybenzamide **41** (0.83 g, 3.51 mmol), sodium hydride (0.14 g, 3.50 mmol) and benzyl bromide (0.42 mL, 0.60 g, 3.53 mmol) in DMF (15 mL) gave **42a** (0.79 g, 69%) as a brown solid which was used without further purification, mp 45–48 °C; ν_{max}/cm^{-1} 3391, 2955, 2868, 1651, 1634, 1535, 1499, 1219, 1005, 806, 725 and 692; δ_H (500 MHz) 8.04 (1 H, br s, NH), 7.68 (1 H, d, *J* 3.5, ArH), 7.44–7.36 (5 H, m, Ph), 6.97 (1 H, d, *J* 9.0, ArH), 6.83 (1 H, dd, *J* 9.0, 3.5, ArH), 5.07 (2 H, s, OCH_2), 3.34 (2 H, td, *J* 7.0, 5.5, NCH_2), 2.92 (6 H, s, NCH_3), 1.37–1.31 (2 H, m, NCH_2CH_2), 1.21–1.14 (2 H, m, CH_2CH_3) and 0.81 (3 H, t, *J* 7.3, CH_3); δ_C (125 MHz) 165.4 (C=O), 148.8 (C), 145.9 (C), 136.1 (C), 128.7 (2CH), 128.5 (CH), 128.1 (2CH), 122.3 (C), 116.9 (CH), 116.4 (CH), 114.3 (CH), 72.1 (OCH_2), 41.3 ($2NCH_3$), 39.3 (NCH_2), 31.2 (CH_2), 20.0 (CH_2) and 13.7 (CH_3); HRMS (NSI^+): found, 327.2065. $C_{20}H_{27}N_2O_2$ (M+H) requires 327.2067.

N-Butyl-5-(dimethylamino)-2-((4-nitrobenzyl)oxy)benzamide **42b**

Preparation as for **40a** using *N*-butyl-5-(dimethylamino)-2-hydroxybenzamide **41** (0.83 g, 3.51 mmol), sodium hydride (0.14 g, 3.50 mmol) and 4-nitrobenzyl bromide (0.76 g, 3.52 mmol) in DMF (15 mL) gave, after recrystallisation (EtOAc/hexane), **42b** (0.72 g, 55%) as orange crystals, mp 155–157 °C; ν_{max}/cm^{-1} 3337, 2961, 2860, 1634, 1605, 1508, 1342, 1231, 1038, 835, 797, 735 and 656; δ_H (500 MHz) 8.28 (2 H, d, *J* 8.5, ArH), 7.74 (1 H, br s, NH), 7.62–7.60 (3 H, m, ArH), 6.90 (1 H, d, *J* 9.0, ArH), 6.79 (1 H, dd, *J* 9.0, 3.3, ArH), 5.20 (2 H, s, OCH_2), 3.39 (2 H, td, *J* 7.0, 5.5, NCH_2), 2.93 (6 H, s, NCH_3), 1.45–1.39 (2 H, m, NCH_2CH_2), 1.28–1.20 (2 H, m, CH_2CH_3) and 0.84 (3 H, t, *J* 7.3, CH_2CH_3); δ_C (125 MHz) 165.4 (C=O), 147.8 (C), 147.7 (C), 146.3 (C), 143.6 (C), 128.2 (2CH), 124.0 (2CH), 123.0 (C), 116.5 (CH), 116.2 (CH), 114.7 (CH), 70.9 (OCH_2), 41.1 ($2NCH_3$), 39.4 (NCH_2), 31.5 (CH_2), 20.1 (CH_2) and 13.7 (CH_3); HRMS ($ASAP^+$): found, 372.1921. $C_{20}H_{26}N_3O_4$ (M+H) requires 372.1918.

N-Butyl-5-(dimethylamino)-2-((4-methoxybenzyl)oxy)benzamide **42c**

Preparation as for **40a** using *N*-butyl-5-(dimethylamino)-2-hydroxybenzamide **41** (0.83 g, 3.51 mmol), sodium hydride (0.14 g, 3.50 mmol) and 4-methoxybenzyl bromide (0.70 g, 3.48 mmol) in DMF (15 mL) gave **42c** (0.70 g, 56%) as a brown solid which was used without further purification, mp 66–70 °C; ν_{max}/cm^{-1} 3375, 2949, 1651, 1611, 1499, 1462, 1179, 1034, 1003, 808 and 727; δ_H (500 MHz) 8.07 (1 H, br s, NH), 7.68 (1 H, d, *J* 3.5, ArH), 7.35 (2 H, d, *J* 8.5, ArH), 6.98 (1 H, d, *J* 9.0, ArH), 6.93 (2 H, d, *J* 8.5, ArH), 6.83 (1 H, dd, *J* 9.0, 3.5, ArH), 5.00 (2 H, s, OCH_2), 3.83 (3 H, s, OCH_3), 3.33 (2 H, td, *J* 7.0, 5.5, NCH_2), 2.93 (6 H, s, NCH_3), 1.36–1.30 (2 H, m, NCH_2CH_2), 1.20–1.12 (2 H, m, CH_2CH_3) and 0.81 (3 H, t, *J* 7.5, CH_3); δ_C (125 MHz) 165.4 (C=O), 159.8 (C–O), 148.9 (C), 145.9 (C), 129.8 (2CH), 128.1 (C), 122.2 (C), 117.0 (CH), 116.4 (CH), 114.3 (CH), 114.1 (2CH), 71.9 (OCH_2), 55.3 (OCH_3), 41.3 ($2NCH_3$), 39.4 (NCH_2), 31.2

(CH_2), 20.0 (CH_2) and 13.8 (CH_3); HRMS (NSI^+): found, 357.2171. $C_{21}H_{29}N_2O_3$ (M+H) requires 357.2173.

N-Butyl-5-(dimethylamino)-2-(hydroxy(phenyl)methyl)benzamide **43a**

Reaction as for **37a** using *n*-butyllithium (0.66 mL, 1.65 mmol) and *N*-butyl-5-(dimethylamino)-2-((4-methoxybenzyl)oxy)benzamide **42a** (0.1632 g, 0.50 mmol) in dry THF (5 mL) gave, after purification by preparative TLC (Al_2O_3, Et_2O), at R_f 0.65, **43a** (74.2 mg, 45%) as a brown oil; ν_{max}/cm^{-1} 3287, 2930, 1601, 1541, 1501, 1449, 1225, 1016, 812, 731 and 700; δ_H (400 MHz) 7.32–7.24 (4 H, m, Ph), 7.21–7.16 (1 H, m, Ph), 7.12 (1 H, d, *J* 8.6, ArH), 6.72 (1 H, d, *J* 2.8, ArH), 6.69 (1 H, dd, *J* 8.6, 2.8, ArH), 6.61 (1 H, br s, OH), 6.06 (1 H, t, *J* 5.2, NH), 5.76 (1 H, s, C*H*OH), 3.26–3.08 (2 H, m, NCH_2), 2.95 (6 H, s, NCH_3), 1.32–1.16 (4 H, m, $NCH_2CH_2CH_2$) and 0.87 (3 H, t, *J* 7.2, CH_2CH_3); δ_C (125 MHz) 171.5 (C=O), 149.7 (CN), 143.7 (C), 136.8 (C), 131.2 (CH), 130.6 (C), 127.7 (2CH), 126.4 (CH), 126.3 (2CH), 113.5 (CH), 111.3 (CH), 74.6 (CHOH), 40.4 ($2NCH_3$), 39.7 (NCH_2), 31.2 (CH_2), 20.0 (CH_2) and 13.7 (CH_3); HRMS (ESI^+): found, 349.1877. $C_{20}H_{26}NaN_2O_2$ (M+Na) requires 349.1885.

N-Butyl-5-(dimethylamino)-2-(hydroxy(4-methoxyphenyl)methyl)benzamide **43c**

Reaction as for **37a** using *n*-butyllithium (0.66 mL, 1.65 mmol) and *N*-butyl-5-(dimethylamino)-2-((4-methoxybenzyl)oxy)benzamide **42c** (0.1775 g, 0.50 mmol) in dry THF (5 mL) gave, after purification by preparative TLC (Al_2O_3, Et_2O), at R_f 0.60, **43c** (46.4 mg, 26%) as a brown oil; ν_{max}/cm^{-1} 3304, 2932, 1601, 1508, 1360, 1302, 1246, 1171, 1032, 812 and 731; δ_H (400 MHz) 7.23 (2 H, d, *J* 8.6, ArH), 7.13 (1 H, d, *J* 8.4, ArH), 6.81 (2 H, d, *J* 8.6, ArH), 6.72 (1 H, d, *J* 2.8, ArH), 6.69 (1 H, dd, *J* 8.4, 2.8, ArH), 6.04 (1 H, t, *J* 5.6, NH), 5.74 (1 H, s, C*H*OH), 3.78 (3 H, s, OCH_3), 3.30–3.11 (2 H, m, NCH_2), 2.95 (6 H, s, NCH_3), 1.35–1.18 (4 H, m, $NCH_2CH_2CH_2$) and 0.88 (3 H, t, *J* 7.2, CH_2CH_3); δ_C (125 MHz) 171.5 (C=O), 158.2 (C–O), 149.7 (CN), 136.8 (C), 135.9 (C), 131.0 (CH), 130.9 (C), 127.4 (2CH), 113.6 (CH), 113.1 (2CH), 111.4 (CH), 74.2 (CHOH), 55.2 (OCH_3), 40.4 ($2NCH_3$), 39.7 (NCH_2), 31.3 (CH_2), 20.0 (CH_2) and 13.7 (CH_3); HRMS (ESI^+): found, 379.1981. $C_{21}H_{28}NaN_2O_3$ (M+Na) requires 379.1992.

2-(Benzyloxy)-6-fluorobenzoic acid

To a stirred solution of 2-fluoro-6-hydroxybenzoic acid (2.0 g, 12.8 mmol) in DMF (20 mL) was added benzyl chloride (5.2 mL, 5.72 g, 44.8 mmol) and K_2CO_3 (8.85 g, 64.1 mmol) and the mixture was heated under reflux for 1 h. The mixture was cooled to rt, diluted with H_2O and transferred to a separating funnel. The mixture was extracted with dichloromethane and the extract evaporated. The residue was taken up in a mixture of NaOH (40% aq., 6 mL) and MeOH (12 mL) and heated under reflux for 6 h then cooled to rt and diluted with H_2O. Acidification with conc. HCl led to formation of a precipitate which was filtered off. The precipitate was partitioned between H_2O and EtOAc. The layers were separated, and the aqueous layer extracted with EtOAc (× 3). The combined organic layers were dried over $MgSO_4$ and concentrated to give the crude product. This was redissolved in Et_2O (30 mL) and washed with 2 M NaOH solution (2 × 20 mL). The aqueous layer was reacidified with 2 M HCl solution and extracted with EtOAc (3 × 40 mL). This process was repeated twice. The organic layers were dried over $MgSO_4$ and concentrated to give the title compound (1.97 g, 63%) as an orange solid which was used without further purification, mp 61−63 °C; δ_H (300 MHz) 7.45−7.34 (6 H, m, ArH), 6.89−6.78 (2 H, m, ArH), 5.23 (2 H, s, OCH_2); δ_C (125 MHz) 167.2 (4ry, C=O), 161.6 (4ry, d, J_{CF} 255.6, C-F), 157.7 (4ry, d, J_{CF} 5.6, ArC-O), 135.7 (4ry, ArC), 133.1 (d, J_{CF} 10.9, CH), 128.9 (2 x CH), 128.5 (CH), 127.3 (2 x CH), 110.9 (4ry, d, J_{CF} 15.7, ArC), 109.4 (d, J_{CF} 22.1, CH), 108.9 (d, J_{CF} 3.1, CH) and 71.5 (OCH_2); δ_F (376 MHz)

−110.6. The ^{1}H spectral data was in accordance with that previously reported.[49] The ^{13}C and ^{19}F spectral data are reported for the first time.

<u>2-(benzyloxy)-*N*-butyl-6-fluorobenzamide **44**</u>

To a stirred solution of 2-(benzyloxy)-6-fluorobenzoic acid (1.50 g, 6.1 mmol) and DMF (1 drop) in CH_2Cl_2 (15 mL) at 0 °C was added oxalyl chloride (0.57 mL, 0.84 g, 6.7 mmol) dropwise and the solution stirred at rt for 3 h. The mixture was concentrated to give the crude acid chloride (1.87 g, quant.); δ_H (400 MHz) 7.45−7.31 (6 H, m, ArH), 6.81−6.72 (2 H, m, ArH) and 5.18 (2 H, s, OCH_2). This was redissolved in CH_2Cl_2 (15 mL) and cooled to 0 °C and to it was added Et_3N (0.94 mL, 0.68 g, 6.7 mmol) and *n*-butylamine (0.66 mL, 0.48 g, 6.7 mmol) dropwise and the solution stirred at rt overnight. The mixture was poured into H_2O (15 mL), extracted with CH_2Cl_2 (3 × 20 mL), and the organic layers dried over $MgSO_4$, and concentrated. The crude residue was recrystallised (EtOH) to give **44** (0.86 g, 47%) as colourless crystals, mp 112−115 °C; ν_{max}/cm^{-1} 3291, 2959, 2932, 2870, 1636, 1614, 1456, 1240, 1053, 694, 652; δ_H (400 MHz) 7.42−7.29 (5 H, m, ArH), 7.28−7.22 (1 H, m, ArH), 6.78−6.66 (2 H, m, ArH), 6.05 (1 H, br s, NH), 5.09 (2 H, s, OCH_2), 3.39 (2 H, td, *J* 7.1, 5.8, NHC*H*$_2$); 1.50−1.41 (2 H, m, NHCH$_2$C*H*$_2$), 1.34−1.24 (2 H, m, C*H*$_2$CH$_3$) and 0.85 (3 H, t, *J* 7.3, CH$_2$C*H*$_3$); δ_C (101 MHz) 162.8 (C=O), 160.4 (d, J_{CF} 250.0, CF), 156.5 (d, J_{CF} 7.0, ArC–O), 136.0 (C), 130.9 (d, J_{CF} 10.4, CH), 128.5 (2CH), 128.1 (CH), 127.2 (2CH), 115.4 (d, J_{CF} 18.8, C), 108.8 (d, J_{CF} 22.3, CH) 108.3 (d, J_{CF} 3.0, CH), 70.9 (OCH_2), 39.5 ($NHCH_2$), 31.4 (CH_2), 19.9 (CH_2) and 13.7 (CH_3); δ_F (376 MHz) −113.9; HRMS (ESI$^+$): found, 302.1544. $C_{18}H_{21}NO_2F$ (M+H) requires 302.1556.

<u>*N*-butyl-2-fluoro-6-(hydroxy(phenyl)methyl)benzamide **45**, 7-butyl-3-phenylphthalide **47** and, 7-fluoro-3-phenylphthalide **46**</u>

Reaction as for **37a** using 2-(benzyloxy)-*N*-butyl-6-fluorobenzamide **44** (150.7 mg, 0.5 mmol) and *n*-butyllithium (0.48 mL, 1.1 mmol) in THF (5 mL) for 10 min gave *N*-butyl-2-fluoro-6-(hydroxy(phenyl)methyl)benzamide **45** (140 mg). The crude residue was dissolved in toluene (5 mL), and *p*-TsOH•H_2O (209.2 mg, 1.1 mmol) was added. The mixture was stirred at rt for 1 h before being diluted by addition H_2O (10 mL). The layers were separated, and the aqueous layer was extracted with CH_2Cl_2 (3 × 10 mL). The organic layers were dried over $MgSO_4$ and concentrated. The crude residue was purified *via* preparative TLC (hexane/Et_2O 1:1) to give, at R_f 0.62, 7-butyl-3-phenylphthalide **47** (10.1 mg, 7%) as a pale-yellow solid, mp 56−59 °C; ν_{max}/cm^{-1} 2957, 2930, 2860, 1755, 1597, 1454, 762, 698 ; δ_H (400 MHz) 7.50 (1 H, t, *J* 7.6, ArH), 7.40−7.34 4 H, m, ArH), 7.29−7.26 (3 H, m, ArH), 7.15−7.09 (1 H, m, ArH), 6.32 (1 H, s, CHO), 3.24−3.06 (2 H, m, $ArCH_2$), 1.75−1.63 (2 H, m, ArCH$_2$C*H*$_2$), 1.48−1.38 (2 H, m, C*H*$_2$CH$_3$) and 0.96 (3 H, t, *J* 7.3, CH$_2$C*H*$_3$); δ_C (101 MHz) 170.4 (4ry, C=O), 150.4 (C), 144.9 (C), 136.9 (C), 134.0 (CH), 129.9 (CH), 129.1 (CH), 128.9 (2CH), 126.9 (2CH), 122.5 (C), 120.1 (CH), 81.7 (CHO), 33.3 (CH_2), 30.8 (CH_2), 22.7 (CH_2) and 14.1 (CH_3); HRMS (ESI$^+$): found, 289.1196. $C_{18}H_{18}NaO_2$ (M+Na) requires 289.1204.

This was followed by a second fraction at R_f 0.33 which was 7-fluoro-3-phenylphthalide **46** (31 mg, 27%) as a colourless oil; δ_H (400 MHz) 7.67−7.54 (1 H, m, ArH), 7.41−7.38 (3 H, m, ArH), 7.30−7.26 (2 H, m, ArH), 7.18 (1 H, t, *J* 8.5, ArH), 7.11 (1 H, d, *J* 7.6, ArH) and 6.39 (1 H, s, benzyl CH); δ_C (101 MHz) 166.5 (d, J_{CF} 2.8, C=O), 159.4 (d, J_{CF} 265.0, CF), 152.2 (d, J_{CF} 1.2, C), 136.8 (d, J_{CF} 7.7, CH), 135.8 (C), 129.5 (CH), 129.0 (2CH), 126.8 (2CH), 118.8 (d, J_{CF} 4.4, CH), 116.2 (d, J_{CF} 18.8, CH), 113.4 (d, J_{CF} 14.3, C) and 82.3 (CHO); δ_F (376 MHz) −114.0. The ^{1}H and ^{13}C spectral data was in accordance with that previously reported.[50] The ^{19}F spectral data is reported for the first time.

2-(benzyloxy)-*N*-butyl-5-methoxybenzamide **48**

To a stirred solution of 2-(benzyloxy)-5-methoxybenzoic acid[51] (1.50 g, 5.8 mmol) and DMF (1 drop) in CH_2Cl_2 (15 mL) at 0 °C was added oxalyl chloride (0.55 mL, 0.81 g, 6.4 mmol) dropwise and the solution stirred at rt for 3 h. The mixture was concentrated to give the crude acid chloride (1.75 g, quant.); 7.58 (1 H, d, *J* 3.1, ArH), 7.49−7.44 (2 H, m, ArH), 7.40−7.35 (2 H, m, ArH), 7.35−7.27 (1 H, m, ArH), 7.13−7.08 (1 H, m, ArH), 6.98 (1 H, d, *J* 9.1, ArH), 5.15 (2 H, s, OCH_2), 3.82 (3 H, s, OCH_3). This was redissolved in CH_2Cl_2 (15 mL) and cooled to 0 °C and to it was added Et_3N (0.89 mL, 0.65 g, 6.4 mmol) and *n*-butylamine (0.63 mL, 0.47 g, 6.4 mmol) dropwise and the solution stirred at rt overnight. The mixture was poured into H_2O (30 mL), extracted with CH_2Cl_2 (3 × 30 mL), and the organic layers dried over $MgSO_4$, and concentrated. The crude residue was recrystallised (Et_2O) to give **48** (1.06 g, 58%) as slightly orange crystals, mp 49−52; ν_{max}/cm^{-1} 3372, 2959, 2932, 1643, 1537, 1487, 1474, 1285, 1269, 1206, 1038, 991, 806, 770, 741, 702, 559; δ_H (400 MHz) 8.02 (1 H, t, *J* 5.3, NH), 7.80 (1 H, dd, *J* 2.5, 1.1, ArH), 7.44−7.37 (5 H, m, ArH), 7.00−6.94 (2 H, m, ArH), 5.07 (2 H, s, OCH_2, 3.79 (3 H, s, OCH_3), 3.33. (2 H, td, *J* 6.9, 5.3, $NHCH_2$) 1.39−1.28 (2 H, m, $NHCH_2CH_2$), 1.21−1.09 (CH_2CH_3) and 0.80 (3 H, t, *J* 7.3, CH_3); δ_C (101 MHz) 164.6 (C=O), 153.8 (C–O), 150.8 (C–O), 135.5 (C), 128.6 (2CH), 128.5 (CH), 128.0 (2CH), 122.4 (C), 118.8 (CH), 115.4 (CH), 114.1 (CH), 71.8 (OCH_2), 55.5 (OCH_3), 39.3 ($NHCH_2$), 31.0 (CH_2), 19.8 (CH_2), 13.6 (CH_3); HRMS (ESI$^+$): found, 314.1746. $C_{19}H_{24}NO_3$ (M+H) requires 314.1756.

N-butyl-2-(hydroxy(phenyl)methyl)-5-methoxybenzamide **49** and 6-methoxy-3-phenylphthalide **50**

Reaction as for **37a** using 2-(benzyloxy)-*N*-butyl-5-methoxybenzamide **48** (156.7 mg, 0.5 mmol) and *n*-butyllithium (0.72 mL, 1.65 mmol) in THF (5 mL) for 10 min gave *N*-butyl-2-(hydroxy(phenyl)methyl)-5-methoxybenzamide **49** (90 mg). The crude residue was dissolved in PhMe (5 mL), and *p*-TsOH•H_2O (209.2 mg, 1.1 mmol) was added. The mixture was stirred at rt for 1 h before being diluted by addition H_2O (10 mL). The layers were separated, and the aqueous layer extracted with CH_2Cl_2 (3 × 10 mL). The organic layers were dried over $MgSO_4$ and concentrated. The crude residue was purified *via* repeated preparative TLC (hexane/Et_2O 7:3) to give, at R_f 0.14, 6-methoxy-3-phenylphthalide **50** (17.4 mg, 14%) as a yellow oil; δ_H (400 MHz) 7.40−7.36 (4 H, m, ArH), 7.29−7.26 (2 H, m, ArH), 7.21 (2 H, d, *J* 1.5, ArH), 6.36 (1 H, s, CHO) and 3.89 (3 H, s, OCH_3); δ_C (101 MHz) 169.9 (C=O), 160.8 (C–O), 142.1 (C), 136.6 (C), 129.2 (CH), 128.9 (2CH), 126.9 (2CH), 123.7 (CH), 123.3 (CH), 107.2 (CH), 82.6 (CHO) and 55.8 (OCH_3). The ^1H and ^{13}C spectral data was in accordance with that previously reported.[50]

2-(benzyloxy)-*N*-butyl-4-methoxybenzamide **51**

To a stirred solution of 2-(benzyloxy)-4-methoxybenzoic acid[52] (1.50 g, 5.8 mmol) and DMF (1 drop) in CH_2Cl_2 (15 mL) at 0 °C was added oxalyl chloride (0.55 mL, 0.81 g, 6.4 mmol) dropwise and the solution stirred at rt for 3 h. The mixture was concentrated to give the crude acid chloride (1.85 g, quant.); δ_H (400 MHz) 8.17 (1 H, d, *J* 9.0, ArH), 7.52−7.46 (2 H, m, ArH), 7.41−7. 635 (2 H, m, ArH), 7.35−7.28 (1 H, m, ArH), 6.56 (1 H, dd, *J* 9.0, 2.3, ArH), 6.50 (1 H, d, *J* 2.3, ArH), 5.18 (2 H, s, OCH_2) and 3.86 (3 H, s, OCH_3). The crude residue was redissolved in CH_2Cl_2 (15 mL) and cooled to 0 °C. To the solution was added Et_3N (0.89 mL, 0.65 g, 6.4 mmol) and *n*-butylamine (0.63 mL, 0.47 g, 6.4 mmol) dropwise and the solution stirred at rt overnight. The mixture was poured into H_2O (15 mL), extracted with CH_2Cl_2 (3 × 30 mL), and the organic layers dried over $MgSO_4$, and concentrated. The crude residue was triturated (Et_2O) to give **51** (320 mg, 18%) as a colourless solid. The mother liquor was purified *via* flash column chromatography

(gradient elution hexane/Et_2O 9:1 to hexane/Et_2O 7:3) to give **51** (360 mg, 20%) as a colourless solid for a combined yield of 38%, mp 78−81 °C; ν_{max}/cm^{-1} 3401, 2957, 2930, 2870, 1636, 1605, 1533, 1501, 1261, 1202, 1098, 1001, 839, 696, 554; δ_H (500 MHz) 8.23 (1 H, d, *J* 8.8, ArH), 7.80 (1 H, br s, NH), 7.47−7.39 (5 H, m, ArH), 6.63 (1 H, dd, *J* 8.8, 2.3, ArH), 6.58 (1 H, d, *J* 2.3, ArH), 5.11 (2 H, s, OCH_2), 3.85 (3 H, s, OCH_3), 3.33 (2 H, td, *J* 7.0, 5.2, $NHCH_2$), 1.34−1.26 (2 H, m, $NHCH_2CH_2$) 1.18−1.09 (2 H, m, CH_2CH_3) and 0.79 (3 H, t, *J* 7.3, CH_2CH_3); δ_C (125 MHz) 164.9 (C=O), 163.1 (C–O), 158.0 (C–O), 135.3 (C), 133.9 (CH), 128.9 (2CH), 128.8 (CH), 128.3 (2CH), 114.7 (C), 105.5 (CH), 99.6 (CH), 71.4 (OCH_2), 55.5 (OCH_3), 39.3 ($NHCH_2$), 31.2 (CH_2), 20.0 (CH_2) and 13.8 (CH_3); HRMS (ESI$^+$): found, 314.1744. $C_{19}H_{24}NO_3$ (M+H) requires 314.1756.

N-butyl-2-(hydroxy(phenyl)methyl)-4-methoxybenzamide **52** and 5-methoxy-3-phenylphthalide **53**

Reaction as for **37a** using 2-(benzyloxy)-*N*-butyl-4-methoxybenzamide **51** (156.7 mg, 0.5 mmol) and *n*-butyllithium (0.72 mL, 1.65 mmol) in THF (5 mL) for 10 min gave *N*-butyl-2-(hydroxy(phenyl)methyl)-4-methoxybenzamide **52** (140 mg). The crude product was dissolved in PhMe (5 mL), and *p*-TsOH•H_2O (209.2 mg, 1.1 mmol) was added. The mixture was stirred at rt for 1 h before being diluted by addition H_2O (10 mL). The layers were separated, and the aqueous layer extracted with CH_2Cl_2 (3 × 10 mL). The organic layers were dried over $MgSO_4$ and concentrated. The crude residue was purified *via* preparative TLC (hexane/Et_2O 1:1) to give, at R_f 0.19, 5-methoxy-3-phenylphthalide **53** (68.4 mg, 57%) as a slightly yellow oil; δ_H (300 MHz) 7.85 (1 H, d, *J* 8.5, ArH), 7.42−7.36 (3 H, m, ArH), 7.30−7.26 (2 H, m, ArH), 7.05 (1 H, dd, *J* 8.5, 2.2, ArH), 6.72 (1 H, d, *J* 2.1, ArH), 6.31 (1 H, s, benzyl CH) and 3.83 (3 H, s, OCH_3); δ_C (101 MHz) 170.2 (C=O), 164.8 (C–O), 152.4 (C), 136.4 (C), 129.1 (CH), 128.9 (2CH), 127.0 (CH), 126.9 (2CH), 117.7 (C), 116.8 (CH), 106.5 (CH), 82.0 (CHO) and 55.8 (OCH_3). The ^1H and ^{13}C spectral data was in accordance with that previously reported.[48]

2-(benzyloxy)-*N*-butyl-5-fluorobenzamide **54**

To a stirred solution of 2-(benzyloxy)-5-fluorobenzoic acid[53] **75** (1.50 g, 6.1 mmol) and DMF (1 drop) in CH_2Cl_2 (15 mL) at 0 °C was added oxalyl chloride (0.57 mL, 0.84 g, 6.7 mmol) and the solution was stirred at rt for 3 h. The mixture was concentrated to give the crude acid chloride (1.71 g, quant); δ_H (400 MHz) 7.80 (1 H, dd, *J* 8.3, 3.2, ArH), 7.48−7.42 (2 H, m, ArH), 7.42−7.37 (2 H, m, ArH), 7.37−7.31 (1 H, m, ArH), 7.31−7.24 (1 H, m, ArH), 7.00 (1 H, dd, *J* 9.2, 4.1, ArH) and 5.19 (2 H, s, OCH_2). The crude residue was redissolved in CH_2Cl_2 (15 mL) and cooled to 0 °C and to it was added Et_3N (0.94 mL, 0.68 g, 6.7 mmol) and *n*-butylamine (0.66 mL, 0.48 g, 6.7 mmol) dropwise and the solution stirred at rt overnight. The solution was poured into H_2O (15 mL), extracted with CH_2Cl_2 (3 × 20 mL), and the organic layers dried over $MgSO_4$, and concentrated. The crude residue was recrystalised (EtOH) to give **54** (0.78 g, 43%) as colourless crystals, mp 75−78 °C; ν_{max}/cm^{-1} 3372, 2955, 2872, 1649, 1553, 1485, 1474, 1263, 1190, 1001, 814, 777, 741, 700, 548; δ_H (400 MHz) 7.95 (1 H, dd, *J* 9.6, 3.3, ArH), 7.92 (1 H, br s, NH), 7.46−7.40 (5 H, m, ArH), 7.11 (1 H, ddd, *J* 9.0, 2.7, 3.3, ArH), 7.00 (1 H, dd, *J* 9.0, 4.2, ArH), 5.12 (2 H, s, OCH_2), 3.33. (2 H, td, *J* 7.0, 5.3, $NHCH_2$), 1.36−1.27 (2 H, m, $NHCH_2CH_2$), 1.20−1.09 (2 H, m, CH_2CH_3) and 0.80 (3 H, t, *J* 7.3, CH_2CH_3); δ_C (125 MHz) 163.7 (4ry, d, J_{CF} 1.3, C=O), 157.2 (d, J_{CF} 240.2, CF), 152.8 (d, J_{CF} 1.9, ArC-O), 135.1 (C), 128.9 (3CH), 128.2 (2CH), 123.4 (d J_{CF} 6.7, C), 118.7 (d, J_{CF} 20.0, CH), 118.5 (d, J_{CF} 21.6, CH), 113.8 (d, J_{CF} 7.6, CH), 72.0 (OCH_2), 39.4 ($NHCH_2$), 31.0 (CH_2), 19.9 (CH_2), 13.7 (CH_3); δ_F (376 MHz) −121.7; HRMS (ESI$^+$): found, 324.1364. $C_{18}H_{20}FNO_2Na$ (M+Na) requires 324.1376.

4-(benzyloxy)-3,5-dimethylbenzoic acid **F**

To a stirred solution of 4-hydroxy-3,5-dimethylbenzoic acid (1.0 g, 6.0 mmol) in DMF (10 mL) was added benzyl chloride (2.4 mL, 21.1 mmol) and K_2CO_3 (4.16 g, 30.1 mmol) and the mixture was heated under reflux for 1 h. The mixture was cooled to rt, diluted with H_2O and transferred to a separating funnel. The mixture was extracted with dichloromethane and the extract evaporated. The residue was taken up in a mixture of NaOH (40% aq., 3 mL) and MeOH (6 mL) and heated under reflux for 6 h then cooled to rt and diluted with H_2O. Acidification with conc. HCl led to formation of a precipitate which was filtered off. The precipitate was partitioned between H_2O and EtOAc. The layers were separated, and the aqueous layer extracted with EtOAc (× 3). The combined organic layers were dried over $MgSO_4$ and concentrated to give the crude product. Recrystallisation of this (EtOH/H_2O) gave product **F** (1.08 g, 70%) as colourless crystals, mp 151−154 °C; ν_{max}/cm^{-1} 2916, 2610, 1676, 1597, 1422, 1304, 1202, 1192, 903, 735, 702, 692; δ_H (300 MHz) 7.82 (2 H, s, ArH), 7.50−7.34 (5 H, m, ArH), 4.87 (2 H, s, OCH_2) and 2.34 (6 H, s, $2CH_3$); δ_C (125 MHz) 171.7 (C=O), 160.5 (C–O), 137.0 (C), 131.5 (2C), 131.2 (2CH), 128.6 (2CH), 128.2 (CH), 127.9 (2CH), 124.6 C), 74.1 (OCH_2) and 16.5 ($2CH_3$); HRMS (ESI$^-$): found, 255.1023. $C_{16}H_{15}O_3$ (M−H) requires 255.1021.

4-(benzyloxy)-*N*-butyl-3,5-dimethylbenzamide **55**

To a stirred solution of 4-(benzyloxy)-3,5-dimethylbenzoic acid **F** (1.0 g, 3.9 mmol) and DMF (1 drop) in CH_2Cl_2 (10 mL) at 0 °C was added oxalyl chloride (0.37 mL, 0.55 g, 4.3 mmol) dropwise and the solution stirred at rt for 3 h. The mixture was concentrated to give the crude acid chloride (1.19 g, quant.). δ_H (400 MHz) 7.84−7.82 (2 H, m, ArH), 7.47−7.36 (6 H, m, ArH), 4.88 (2 H, s, OCH_2), 2.34 (6 H, s, 2 x CH_3). The crude residue was redissolved in CH_2Cl_2 (10 mL) and cooled to 0 °C and to it was added Et_3N (0.60 mL, 0.44 g, 4.3 mmol) and *n*-butylamine (0.43 mL, 0.32 g, 4.3 mmol) dropwise and the solution stirred at rt overnight. The mixture was poured into H_2O (10 mL), extracted with CH_2Cl_2 (3 × 20 mL), and the organic layers dried over $MgSO_4$ and concentrated. The crude residue was recrystallised (EtOAc/hexane) to give **55** (0.86 g, 71%) as a colourless solid, mp 91−94 °C; ν_{max}/cm^{-1} 3287, 2959, 2930, 2872, 1630, 1533, 1468, 1454, 1319, 1202, 752, 743, 712, 692; δ_H (300 MHz) 7.50−7.34 (7 H, m, ArH), 6.10 (1 H, br s, NH), 4.82 (2 H, s, OCH_2), 3.44 (2 H, td, *J* 7.1, 5.6, $NHCH_2$), 2.31 (6 H, s, $2CH_3$), 1.65−1.54 (2 H, m, $NHCH_2CH_2$), 1.48−1.35 (2 H, m, CH_2CH_3) and 0.96 (3 H, t, *J* 7.3, CH_2CH_3); δ_C (125 MHz) 167.4 (C=O), 158.2 (C–O), 137.1 (C), 131.4 (2C), 130.3 (C), 128.5 (2CH), 128.1 (CH), 127.8 (2CH), 127.6 (2CH), 74.0 (OCH_2), 39.7 ($NHCH_2$), 31.7 (CH_2), 20.1 (CH_2), 16.5 ($2CH_3$) and 13.8 (CH_3); HRMS (ESI$^+$): found, 312.1952. $C_{20}H_{26}NO_2$ (M+H) requires 312.1964.

Phenyl(4-(trimethylsilyl)phenyl)methanol **57**

Reaction as for **37a** using (4-(benzyloxy)phenyl)trimethylsilane[54] **56** (128.2 mg, 0.5 mmol) and *n*-butyllithium (0.24 mL, 0.55 mmol) in THF (5 mL) at rt for 10 min gave, after purification *via* flash column chromatography (hexane/EtOAc 9:1) at R_f 0.29, **57** (76.7 mg, 60%) as a colourless oil; δ_H (300 MHz) 7.52−7.44 (2 H, m, ArH), 7.40−7.19 (7 H, m, ArH), 5.76 (1 H, s, C*H*OH), 2.35 (1 H, br s, CHO*H*) and 0.24 (9 H, s, $Si(CH_3)_3$); δ_H (101 MHz) 144.2 (C), 143.6 (C), 139.6 (C), 133.5 (2CH), 128.4 (2CH), 127.5 (CH), 126.4 (2CH), 125.8 (2CH), 76.1 (CHOH) and −1.0 ($Si(CH_3)_3$). The ^{1}H and ^{13}C spectral data was in accordance with that previously recorded.[55]

N-butyl-2-(phenoxymethyl)benzamide **58**

A stirred solution of ethyl 2-(phenoxymethyl)benzoate[56] (5.56 g, 21.7 mmol) in *n*-butylamine (20 mL) was heated under reflux for 3 d. *N*-butylamine (10 mL) was added and the mixture heated

under reflux for 4 d. The mixture was concentrated *in vacuo* and the residue acidified to pH 1 by addition of 2 M HCl. EtOAc (50 mL) was added, and the layers separated. The aqueous layer was extracted with EtOAc (2 × 100 mL) and the combined organic layers dried over $MgSO_4$ and concentrated. The crude residue purified *via* recrystallisation (PhMe/EtOAc) to give **58** (1.66 g, 27%) as a colourless solid. The mother liquor was purified *via* flash column chromatography (gradient elution hexane/EtOAc 9:1 to hexane/EtOAc 2:1), to give **58** (0.72 g, 12%) as a colourless solid for a combined yield of 39%, mp 70−73 °C; ν_{max}/cm^{-1} 3246, 2955, 2928, 2870, 1632, 1597, 1555, 1231, 1032, 750, 694; δ_H (300 MHz) 7.62 (1 H, dd, *J* 7.1, 1.9, ArH), 7.60−7.49 (1H, m, ArH), 7.42 (2 H, dtd, *J* 16.6, 7.3, 1.6, ArH), 7.35−7.27 (2 H, m, ArH), 7.04−6.97 (3 H, m, ArH), 6.44 (1 H, br s, NH), 5.17 (2 H, s, OCH_2), 3.37 (2 H, td, *J* 7.0, 5.6, NHC***H***$_2$), 1.47−1.34 (2 H, m, $NHCH_2C$***H***$_2$), 1.34−1.21 (C***H***$_2CH_3$) and 0.83 (3 H, t, *J* 7.2, CH_2C***H***$_3$); δ_C (125 MHz) 168.7 (C=O), 158.1 (C–O), 136.6 (C), 134.0 (C), 130.4 (CH), 130.1 (CH), 129.6 (2CH), 128.6 (CH), 128.2 (CH), 121.4 (CH), 114.7 (2CH), 68.3 (OCH_2), 39.8 ($NHCH_2$), 31.5 (CH_2), 20.1 (CH_2) and 13.6 (CH_3); HRMS (ESI^+): found, 306.1453. $C_{18}H_{21}NaNO_2$ (M+Na) requires 306.1470.

N-butyl-4-(phenoxymethyl)benzamide **59**

A stirred solution of methyl 4-(phenoxymethyl)benzoate[57] (1.0 g, 4.13 mmol) in *n*-butylamine (10 mL) was heated under reflux for 6 d then cooled to rt. The mixture was concentrated, and the residue acidified to pH 2 by addition of 2 M HCl. EtOAc (20 mL) was added, and the layers separated. The aqueous layer was extracted with EtOAc (3 × 20 mL), and the combined organic layers dried over $MgSO_4$ and concentrated to give, after recrystallisation (hexane/EtOAc) **59** (0.61 g, 52%) as colourless crystals, mp 105−107 °C; ν_{max}/cm^{-1} 3343, 2967, 2949, 2934, 2866, 1638, 1528, 1240, 1013, 835, 748, 689; δ_H (300 MHz) 7.82−7.74 (2 H, m, ArH), 7.53−7.46 (2 H, m, ArH), 7.33−7.23 (2 H, m, ArH), 7.02−6.93 (3 H, m, ArH), 6.11 (1 H, br s, NH), 5.12 (2 H, s, OCH_2); 3.46 (2 H, td, *J* 7.1, 5.1, NHC***H***$_2$), 1.68−1.55 (2 H, m, $NHCH_2C$***H***$_2$), 1.50−1.35 (2 H, m, C***H***$_2CH_3$) and 0.96 (3 H, t, *J* 7.3, CH_3); δ_C (125 MHz) 167.1 (C=O), 158.4 (C–O), 140.5 (C), 134.3 (C), 129.5 (2CH), 127.2 (2CH), 127.1 (2CH), 121.1 (CH), 114.8 (2CH), 69.2 (OCH_2), 39.8 ($NHCH_2$), 31.7 (CH_2), 20.1 (CH_2) and 13.8 (CH_3); HRMS (ESI^+): found, 284.1639. $C_{18}H_{22}NO_2$ (M+H) requires 284.1651.

Diphenylmethanol

Reaction as for **37a** using phenyl benzyl ether[58] (92.1 mg, 0.5 mmol) and *n*-butyllithium (0.24 mL, 0.55 mmol) in THF (5 mL) for 20 min gave, after purification *via* preparative TLC (hexane/EtOAc 9:1) at R_f 0.28, diphenylmethanol (29.6 mg, 32%), mp 61−63 °C; (lit.[59] 60−61 °C); δ_H (300 MHz) 7.40−7.29 (8 H, m, ArH), 7.29−7.23 (2 H, m, ArH), 5.83 (1 H, s, C***H***OH) and 2.19 (1 H, br s, CHO***H***); δ_C (125 MHz) 143.9 (4ry, ArC), 128.6 (4 x CH), 127.7 (2 x CH), 126.7 (4 x CH) and 76.4 (CHOH). The ^1H and ^{13}C spectral data was in accordance with that previously reported.[59]

2-(Benzylthio)-*N*-butylbenzamide **66**

Triethylamine (2.9 mL, 2.11 g, 20.8 mmol) and *n*-butylamine (2.3 mL, 1.70 g, 23.3 mmol) were added to a solution of 2-(benzylthio)benzoyl chloride[2] (5.38 g, 20.5 mmol) in dry toluene (70 mL) and the reaction mixture was heated at reflux for 1 h. After cooling to rt, triethylammonium chloride was removed by filtration and the filtrate was evaporated. The crude residue was recrystallised (EtOH) to give **66** (4.71 g, 77%) as pale yellow crystals, mp 88–90 °C; (lit.[60] 91–92 °C); ν_{max}/cm^{-1} 3312, 1716, 1634, 1544, 1311, 1261, 1168, 1068, 1041, 788, 741 and 720; δ_H (300 MHz) 7.59–7.58 (1 H, m, ArH), 7.38–7.35 (1 H, m, ArH), 7.32–7.15 (7 H, m, ArH and Ph), 6.36 (1 H, br s, NH), 4.07 (2 H, s, SCH_2), 3.36 (2 H, td, *J* 7.1, 5.7, NCH_2), 1.59–1.48 (2 H, m,

NCH_2CH_2), 1.45–1.32 (2 H, m, CH_2CH_3) and 0.94 (3 H, t, *J* 7.2, CH_3); δ_C (75 MHz) 167.8 (C=O), 138.1 (C), 137.0 (C), 133.1 (C), 132.1 (CH), 130.2 (CH), 128.8 (CH), 128.7 (2CH), 128.4 (2CH), 127.2 (CH), 127.0 (CH), 40.1 (SCH_2), 39.7 (NCH_2), 31.4 (CH_2), 20.1 (CH_2) and 13.7 (CH_3); HRMS (ESI^+): found, 322.1226. $C_{18}H_{21}NaNOS$ (M+Na) requires 322.1236.

Attempted thia-[1,2]-Wittig Rearrangement of 2-(Benzylthio)-*N*-butylbenzamide **66** giving **67**

Under a nitrogen atmosphere, *n*-butyllithium (2.5 M in hexane, 6.6 mL, 16.5 mmol) was added dropwise to a stirred solution of 2-(benzylthio)-*N*-butylbenzamide **66** (1.50 g, 5.01 mmol) in dry PhMe (50 mL). After stirring at rt for 2 h, the reaction mixture was quenched by addition of sat. aq. NH_4Cl (50 mL), the two layers were separated and the aq. layer was extracted with Et_2O (2 × 30 mL). The combined organic extracts were dried and evaporated and the crude residue was purified by column chromatography (SiO_2, Et_2O/hexane 1:4) to give, at R_f 0.80, *N*-butyl-2-phenylbenzo[*b*]thiophen-3-amine **67** (0.86 g, 61%) as an orange oil; ν_{max}/cm^{-1} 3401, 2957, 1649, 1512, 1341, 1236, 1175, 1069, 1030, 970, 748 and 696; δ_H (500 MHz) 7.77 (1 H, d, *J* 8.0, ArH), 7.72 (1 H, d, *J* 8.0, ArH), 7.61–7.59 (2 H, m, ArH), 7.45 (2 H, t, *J* 7.8, ArH), 7.39–7.36 (1 H, m, ArH), 7.35–7.31 (2 H, m, ArH), 3.64 (1 H, br s, NH), 3.15 (2 H, t, *J* 7.3, NCH_2), 1.53–1.47 (2 H, m, NCH_2CH_2), 1.35–1.27 (2 H, m, CH_2CH_3) and 0.86 (3 H, t, *J* 7.5, CH_3); δ_C (125 MHz) 137.7 (C), 137.6 (C), 135.8 (C), 134.4 (C), 129.0 (2CH), 128.8 (2CH), 127.4 (CH), 124.4 (CH), 123.8 (CH), 122.7 (CH), 122.1 (C), 121.2 (CH), 48.6 (NCH_2), 32.9 (CH_2), 20.1 (CH_2) and 13.9 (CH_3); HRMS (ESI^+): found, 282.1309. $C_{18}H_{20}NS$ (M+H) requires 282.1311.

2-(Benzylthio)-*N*-(*tert*-butyl)benzamide **68**

Triethylamine (2.9 mL, 2.11 g, 20.8 mmol) and *tert*-butylamine (2.5 mL, 1.74 g, 23.8 mmol) were added to a solution of 2-(benzylthio)benzoyl chloride[2] (5.40 g, 20.5 mmol) in dry toluene (70 mL) and the reaction mixture was heated at reflux for 1 h. After cooling to rt, triethylammonium chloride was removed by filtration and the filtrate was evaporated. The crude residue was recrystallised (EtOH) to give **68** (4.24 g, 69%) as pale yellow crystals, mp 115–118 °C; ν_{max}/cm^{-1} 3281, 2976, 1636, 1544, 1452, 1317, 1218, 1067, 876, 780, 741 and 685; δ_H (400 MHz) 7.58–7.56 (1 H, m, ArH), 7.35–7.32 (1 H, m, ArH), 7.29–7.19 (7 H, m, ArH and Ph), 6.28 (1 H, br s, NH), 4.09 (2 H, s, CH_2) and 1.44 (9 H, s, CH_3); δ_C (100 MHz) 167.2 (C=O), 139.0 (C), 137.1 (C), 132.9 (C), 132.0 (CH), 130.0 (CH), 128.8 (2CH), 128.7 (CH), 128.5 (2CH), 127.2 (CH), 127.0 (CH), 51.8 (CMe_3), 40.0 (CH_2) and 28.7 ($3CH_3$); HRMS (ESI^+): found, 322.1226. $C_{18}H_{21}NaNOS$ (M+Na) requires 322.1236.

2-(Benzyl(methyl)amino)-*N*-butylbenzamide **69**

Under a nitrogen atmosphere, *n*-butyllithium (2.5 M in hexane, 33.0 mL, 82.5 mmol) was added dropwise to a stirred –78 °C solution of *N*-benzylmethylamine (10.6 mL, 9.95 g, 82.1 mmol) in dry THF (50 mL). Once the addition was complete, the reaction mixture was allowed to warm to 0 °C for 30 min before a solution of *N*-butyl-2-methoxybenzamide[61] (7.65 g, 36.9 mmol) in dry THF (75 mL) was added dropwise and the reaction mixture was allowed to warm to rt for 60 h. The reaction mixture was quenched by addition of water (20 mL) and concentrated *in vacuo*. The residue dissolved in Et_2O (250 mL) and extracted with 2 M HCl (150 mL). The aqueous layer was adjusted to pH 14 by addition of 2 M NaOH before being extracted with Et_2O (200 mL). The organic layer was washed with water (100 mL) before being dried and evaporated. The crude residue was purified by column chromatography (SiO_2, Et_2O/hexane 3:2) to give, at R_f 0.50, **69** (8.70 g, 80%) as a brown oil; ν_{max}/cm^{-1} 3296, 2957, 1717, 1653, 1538, 1367, 1288, 1156, 1089,

931, 739 and 699; δ_H (300 MHz) 9.78 (1 H, br s, NH), 8.21 (1 H, ddd, *J* 7.8, 1.8, 0.3, ArH), 7.41 (1 H, ddd, *J* 7.8, 7.5, 1.8, ArH), 7.34–7.29 (3 H, m, ArH), 7.25–7.18 (4 H, m, ArH), 4.11 (2 H, s, NCH_2Ph), 3.44 (2 H, td, *J* 7.2, 5.7, NCH_2CH_2), 2.60 (3 H, s, NCH_3), 1.58–1.48 (2 H, m, NCH_2CH_2), 1.41–1.28 (2 H, m, CH_2CH_3) and 0.91 (3 H, t, *J* 7.2, CH_2CH_3); δ_C (75 MHz) 166.2 (C=O), 151.6 (CN), 136.9 (C), 131.6 (CH), 131.5 (CH), 129.2 (2CH), 128.5 (2CH), 128.2 (C), 127.7 (CH), 124.7 (CH), 121.4 (CH), 61.3 (NCH_2Ph), 42.9 (NCH_3), 39.2 (NCH_2), 31.6 (CH_2), 20.3 (CH_2) and 13.8 (CH_3); HRMS (ESI$^+$): found, 319.1770. $C_{19}H_{24}NaN_2O$ (M+Na) requires 319.1781.

References

1 Gardner, R. A.; Kinkade, R.; Wang, C.; Phanstiel, O. *J. Org. Chem.* **2004**, *69*, 3530–3537.
2 Aitken, R. A.; Harper, A. D.; Slawin, A. M. Z. *Synlett* **2017**, *28*, 1738–1742.
3 Dordor, I. M. J.; Mellor, M.; Kennewell, P. D. *J. Chem. Soc., Perkin Trans. 1* **1984**, 1247–1252.
4 Fontes, E.; Lee, W. K.; Heiney, P. A.; Nounesis, G.; Garland, C. W.; Riera, A.; McCauley, J. P.; Smith, A. B. *J. Chem. Phys.* **1990**, *92*, 3917–3929.
5 Marona, H.; Nowak, A. *Acta Pol. Pharm.* **1988**, *45*, 105–107.
6 Tamagnan, G.; Gao, Y.; Bakthavachalam, V.; White, W. L.; Neumeyer, J. L. *Tetrahedron Lett.* **1995**, *36*, 5861–5864.
7 Colquhoun, H. M.; Goodings, E. P.; Maud, J. M.; Stoddart, J. F.; Wolstenholme, J. B.; Williams, D. J. *J. Chem. Soc., Perkin Trans. 2* **1985**, 607–624.
8 Nikishin, G. I.; Svitanko, I. V.; Troyansky, E. I. *J. Chem. Soc., Perkin Trans. 2* **1983**, 595–601.
9 Wagner, P. J.; Meador, M. A.; Park, B. S. *J. Am. Chem. Soc.* **1990**, *112*, 5199–5211.
10 Asahina, Y.; Asano, J. *Ber. Dtsch. Chem. Ges.* **1929**, *62*, 171–177.
11 Furusawa, T.; Kawano, M.; Fujita, M. *Angew. Chem. Int. Ed.* **2007**, *46*, 5717–5719.
12 Kekulé, A.; Franchimont, A. *Ber. Dtsch. Chem. Ges.* **1872**, *5*, 908–910.
13 Barton, D. H. R.; Hui, R. A. H. F.; Ley, S. V. *J. Chem. Soc., Perkin Trans. 1* **1982**, 2179–2185.
14 Mermod, E.; Simonis, H. *Ber. Dtsch. Chem. Ges.* **1908**, *41*, 982–985.
15 Weeks, D. P.; Cella, J. *J. Org. Chem.* **1969**, *34*, 3713.
16 Kawasaki, T.; Kimachi, T. *Tetrahedron* **1999**, *55*, 6847–6862.
17 Kondo, H.; Kuwana, Y.; Takada, H.; Chigrinov, V.; Kwok, H.-S. *Eur. Pat.* 1767523, **2007**.
18 Piccolo, O.; Filippini, L.; Tinucci, L.; Valoti, E.; Citterio, A. *Tetrahedron* **1986**, *42*, 885–891.
19 Mosher, H. S.; Tessieri, J. E. *J. Am. Chem. Soc.* **1951**, *73*, 4925–4927.
20 Tsuchida, H.; Tamura, M.; Hasegawa, E. *J. Org. Chem.* **2009**, *74*, 2467–2475.
21 Padwa, A.; Meske, M.; Ni, Z. *Tetrahedron* **1995**, *51*, 89–106.
22 Gresly, L. *Liebigs Ann. Chem.* **1886**, *234*, 234–241.
23 Al-Hamdany, R.; Al-Rawi, J. M.; Ibrahim, S. *J. Prakt. Chem.* **1987**, *329*, 126–130.
24 Okada, K.; Tanaka, M. *J. Chem. Soc., Perkin Trans. 1* **2002**, 2704–2711.
25 Nourrisson, C. *Ber. Dtsch. Chem. Ges.* **1886**, *19*, 2103–2107.
26 Runyon, S. P.; Mosier, P. D.; Roth, B. L.; Glennon, R. A.; Westkaemper, R. B. *J. Med. Chem.* **2008**, *51*, 6808–6828.
27 Rayabarapu, D. K.; Chang, H.-T.; Cheng, C.-H. *Chem. Eur. J.* **2004**, *10*, 2991–2996.

28 Fieser, L. F.; Fieser, M. *J. Am. Chem. Soc.* **1933**, *55*, 3010–3018.
29 Bergmann, E. D.; Blum, J.; Butanaro, S. *J. Org. Chem.* **1961**, *26*, 3211–3214.
30 Sather, A. C.; Lee, H. G.; De La Rosa, V. Y.; Yang, Y.; Müller, P.; Buchwald, S. L. *J. Am. Chem. Soc.* **2015**, *137*, 13433–13438.
31 Suzuki, K.; Weisburger, E. K.; Weisburger, J. H. *J. Org. Chem.* **1961**, *26*, 2239–2242.
32 Ye, Z.; Lv, G.; Wang, W.; Zhang, M.; Cheng, J. *Angew. Chem. Int. Ed.* **2010**, *49*, 3671–3674.
33 Karthikeyan, J.; Parthasarathy, K.; Cheng, C.-H. *Chem. Commun.* **2011**, *47*, 10461–10463.
34 Bradley, W.; Maisey, R. F. *J. Chem. Soc.* **1954**, 247–252.
35 Denisov, V. Y.; Grishchenkova, T. N. *J. Org. Chem. USSR (Engl. Transl.)* **1983**, *19*, 2083–2087.
36 Kuriyama, M.; Ishiyama, N.; Shimazawa, R.; Shirai, R.; Onomura, O. *J. Org. Chem.* **2009**, *74*, 9210–9213.
37 Huang, L.-L.; Xu, M.-H.; Lin, G.-Q. *J. Am. Chem. Soc.* **2006**, *128*, 5624–5625.
38 Newman, M. S. *J. Org. Chem.* **1962**, *27*, 323–324.
39 Mahendar, L.; Satyanarayana, G. *J. Org. Chem.* **2015**, *80*, 7089–7098.
40 Abramovitz, A. S.; Massey, V. *J. Biol. Chem.* **1976**, *251*, 5321–5326.
41 Mamidi, N.; Manna, D. *J. Org. Chem.* **2013**, *78*, 2386–2396.
42 Kajitani, T.; Kohmoto, S.; Yamamoto, M.; Kishikawa, K. *J. Mater. Chem.* **2004**, *14*, 3449–3456.
43 Myllymäki, M.; Castillo-Melendez, J.; Koskinen, A.; Minkkilä, A.; Saario, S.; Nevalainen, T.; Järvinen, T.; Poso, A.; Salo-Ahen, O. *PCT Int. Appl.* WO 129129 A1, **2008**.
44 Dias, L. C.; Polo, E. C. *J. Org. Chem.* **2017**, *82*, 4072–4112.
45 Bathini, T.; Rawat, V. S.; Bojja, S. *Tetrahedron Lett.* **2015**, *56*, 5656–5660.
46 Davies, I. W.; Marcoux, J.-F.; Taylor, J. D. O.; Dormer, P. G.; Deeth, R. J.; Marcotte, F.-A.; Hughes, D. L.; Reider, P. J. *Org. Lett.* **2002**, *4*, 439–441.
47 Nisato, D.; Frigerio, M.; Boccardi, G.; Palmisano, G. *Synthesis* **1982**, 1081–1083.
48 Schmidt, B.; Hölter, F.; Berger, R.; Jessel, S. *Adv. Synth. Catal.* **2010**, *352*, 2463–2473.
49 Barnes, D.; Coppola, G. M.; Stams, T.; Topiol, S. W. *PCT Int. Appl.*WO 067612 A1, **2007**.
50 Miura, H.; Terajima, S.; Shishido, T. *ACS Catal.* **2018**, *8*, 6246–6254.
51 Yokokawa, F.; Izumi, K.; Omata, J.; Shioiri, T. *Tetrahedron* **2000**, *56*, 3027–3034.
52 Ghosh, A.; Brueckner, A. C.; Cheong, P. H. Y.; Carter, R. G. *J. Org. Chem.* **2019**, *84*, 9196–9214.
53 France, S.; Shah, M. H.; Weatherwax, A.; Wack, H.; Roth, J. P.; Lectka, T. *J. Am. Chem. Soc.* **2005**, *127*, 1206–1215.
54 Aikawa, K.; Hioki, Y.; Mikami, K. *Chem. Asian J.* **2010**, *5*, 2346–2350.
55 Lee, J.; Oh, Y.; Choi, Y. K.; Choi, E.; Kim, K.; Park, J.; Kim, M. J. *ACS Catal.* **2015**, *5*, 683–689.
56 Naporra, F.; Gobleder, S.; Wittmann, H. J.; Spindler, J.; Bodensteiner, M.; Bernhardt, G.; Hübner, H.; Gmeiner, P.; Elz, S.; Strasser, A. *Pharmacol. Res.* **2016**, *113*, 610–625.
57 Kraus, G. A.; Riley, S.; Cordes, T. *Green Chem.* **2011**, *13*, 2734–2736.
58 Yakukhnov, S. A.; Ananikov, V. P. *Adv. Synth. Catal.* **2019**, *361*, 4781–4789.
59 Otevrel, J.; Svestka, D.; Bobal, P. *RSC Adv.* **2020**, *10*, 25029–25045.
60 Gialdi, F.; Ponci, R.; Baruffini, A. *Farm. Ed. Sci.* **1960**, *15*, 856–882.
61 Nordeman, P.; Odell, L. R.; Larhed, M. *J. Org. Chem.* **2012**, *77*, 11393–11398.

NMR Spectra of New Compounds

2,4-bis(benzyloxy)benzoyl chloride **A**

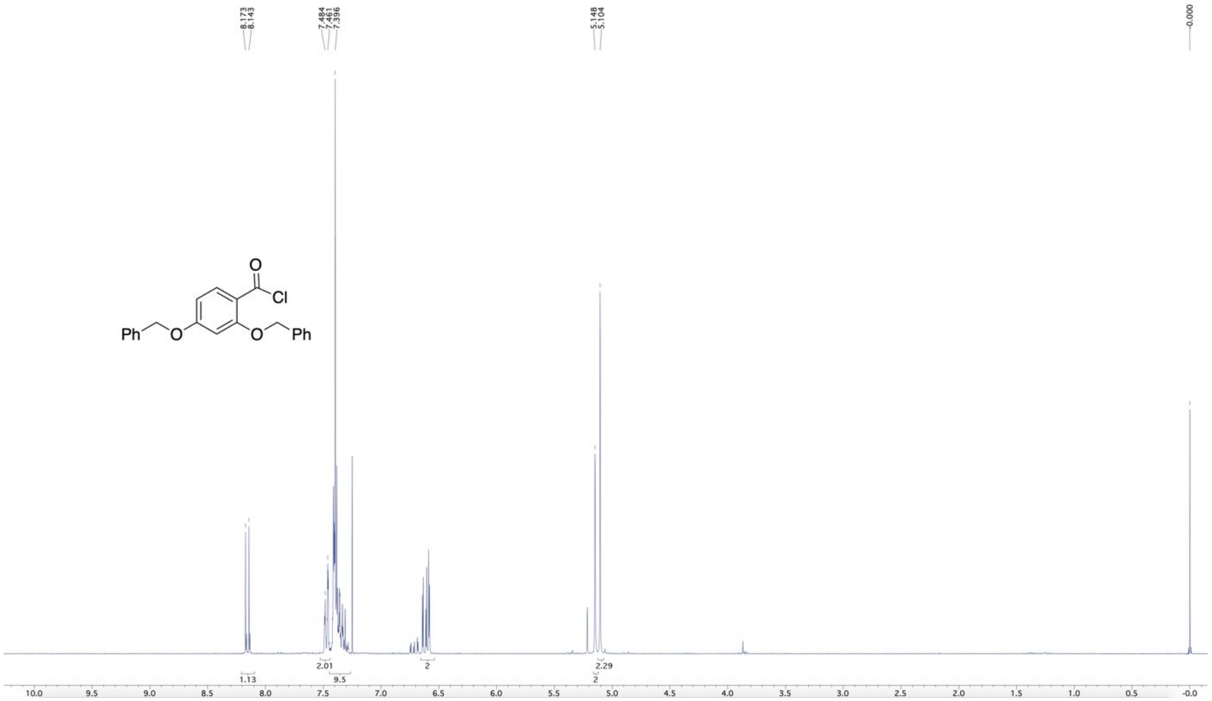

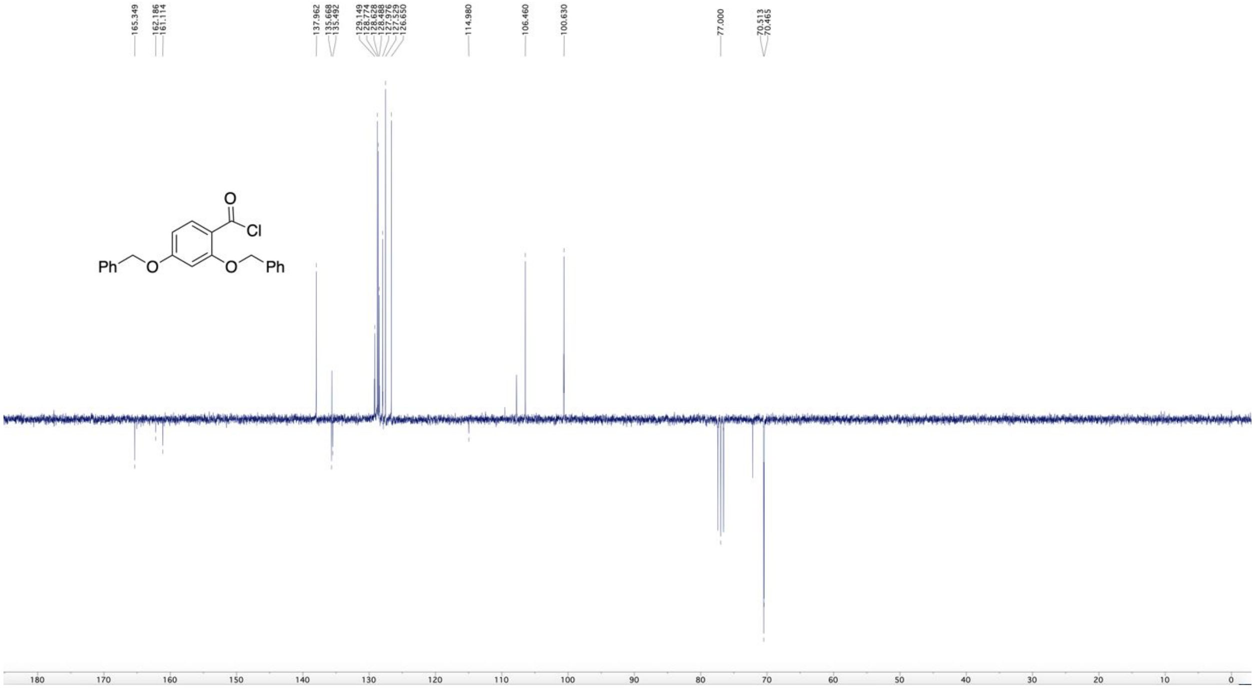

2,4-bis(benzyloxy)-*N*-(1-hydroxy-2-methylpropan-2-yl)benzamide **B**

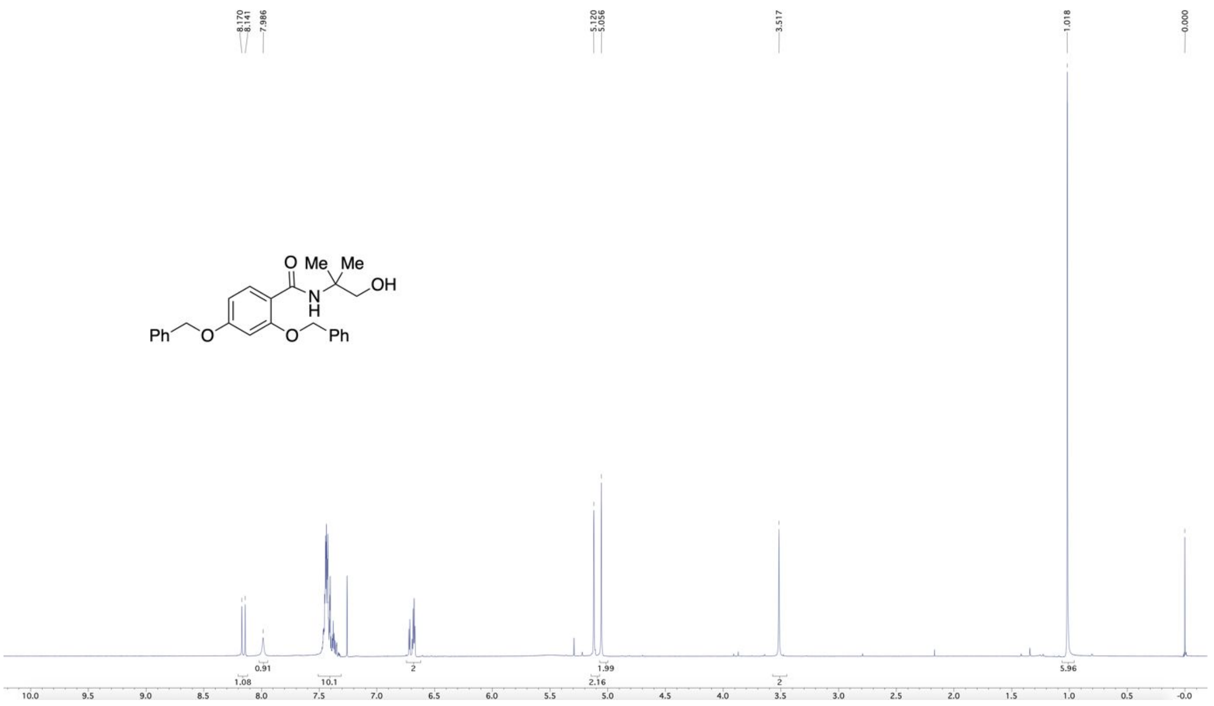

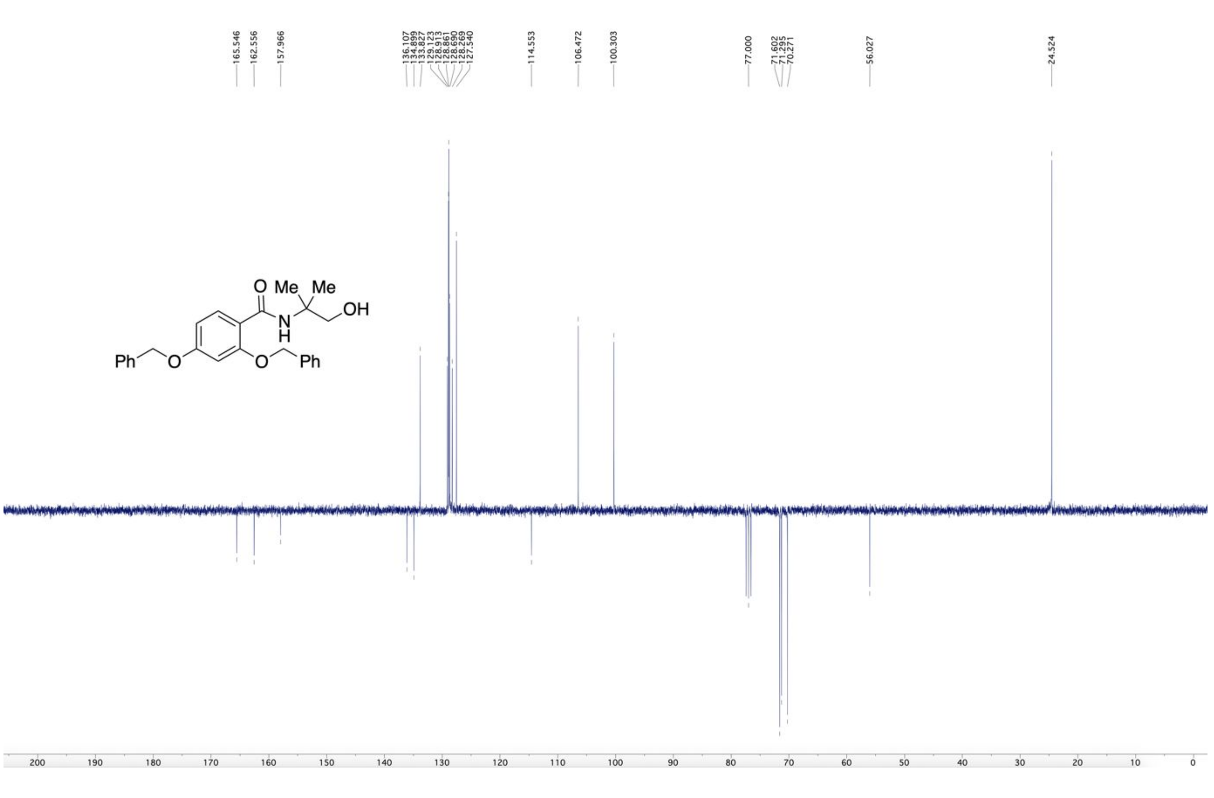

2-(2,4-bis(benzyloxy)phenyl)-4,4-dimethyl-4,5-dihydro-1,3-oxazole **1**

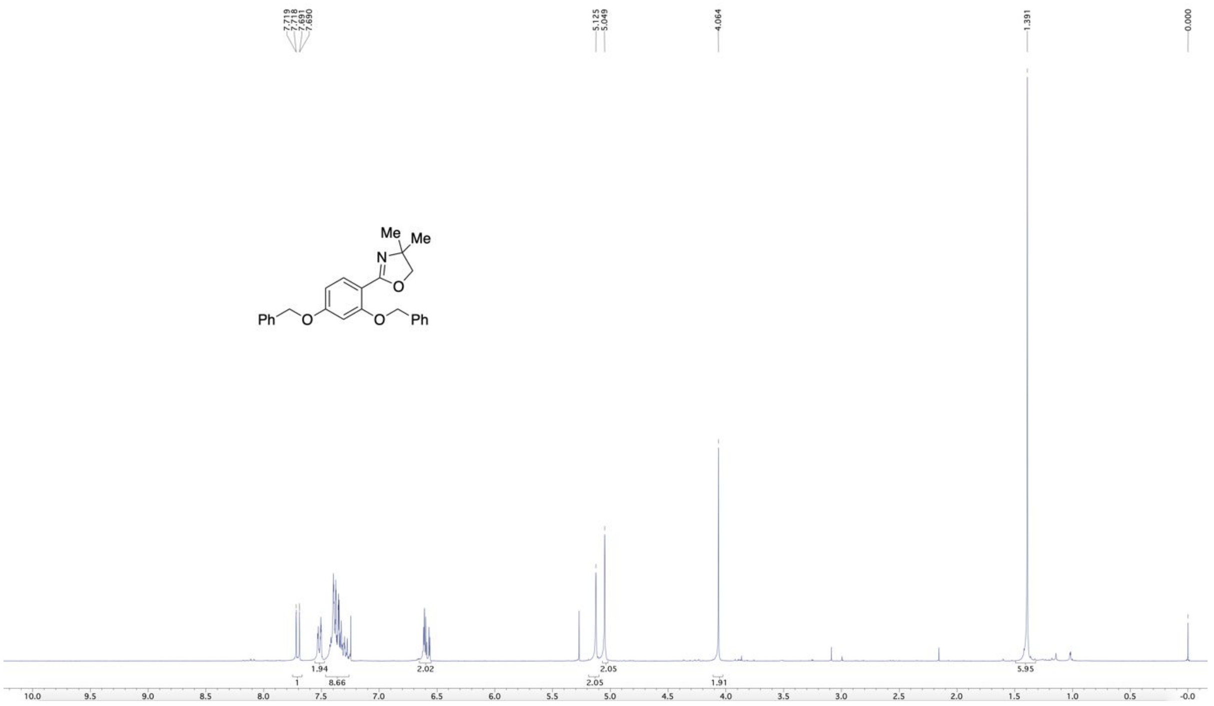

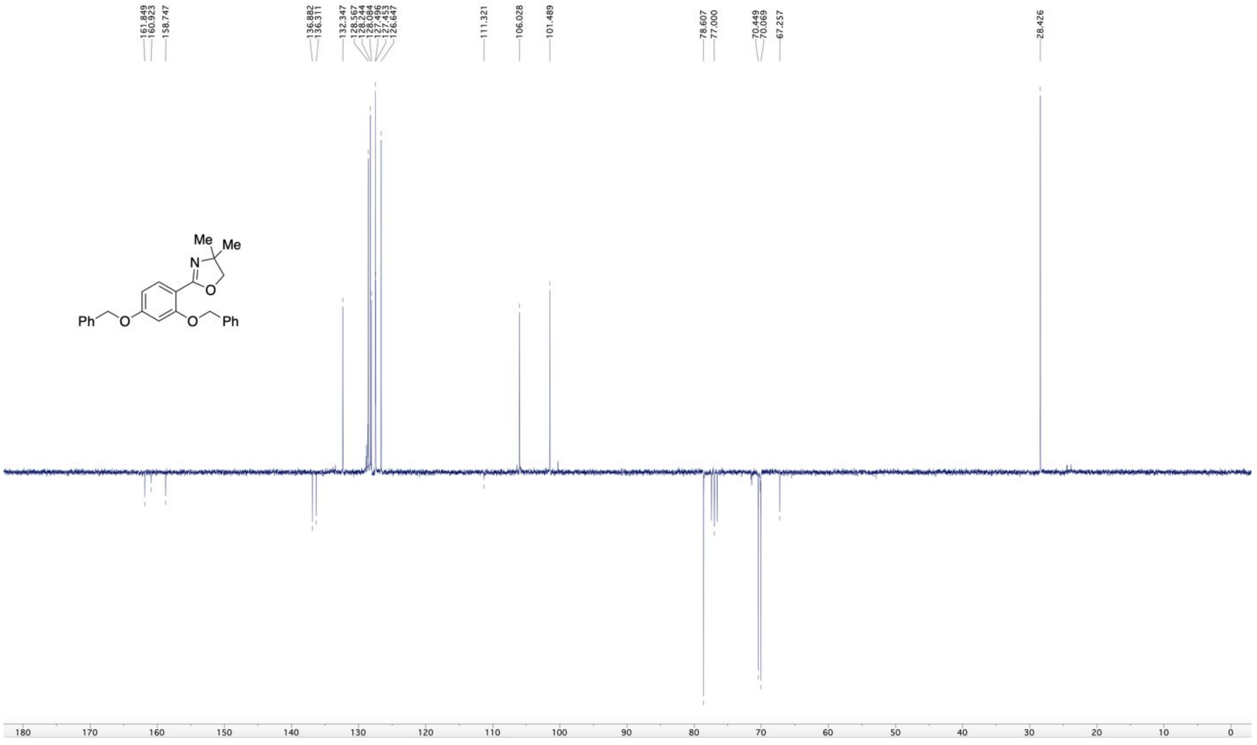

(5-(benzyloxy)-2-(4,4-dimethyl-4,5-dihydrooxazol-2-yl)phenyl)(phenyl)methanone **4**

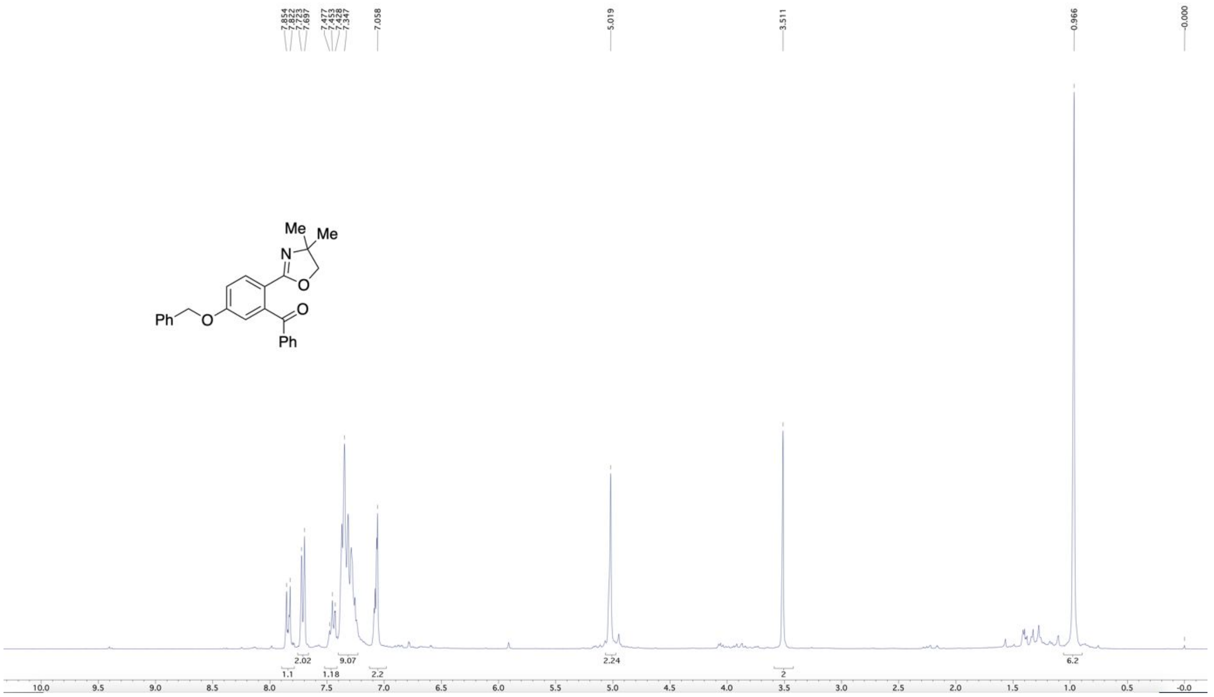

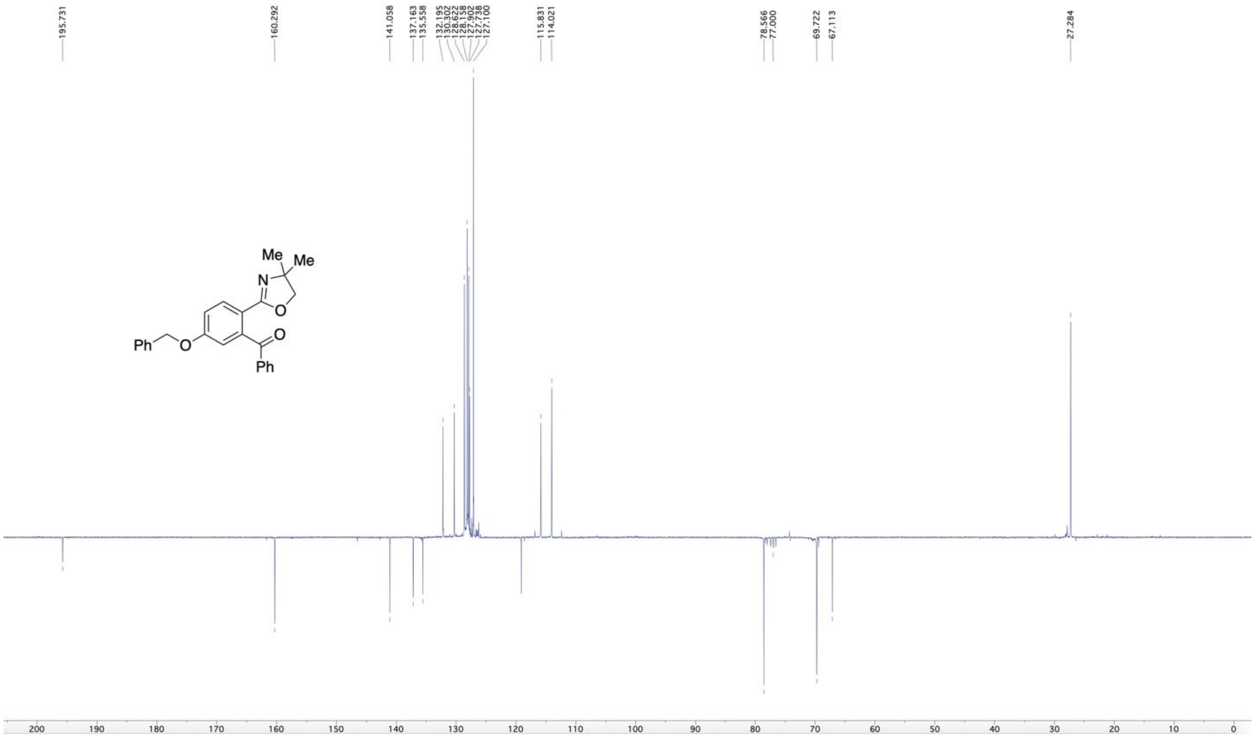

4-(Benzyloxy)-*N*-(1-hydroxy-2-methylpropan-2-yl)benzamide **D**

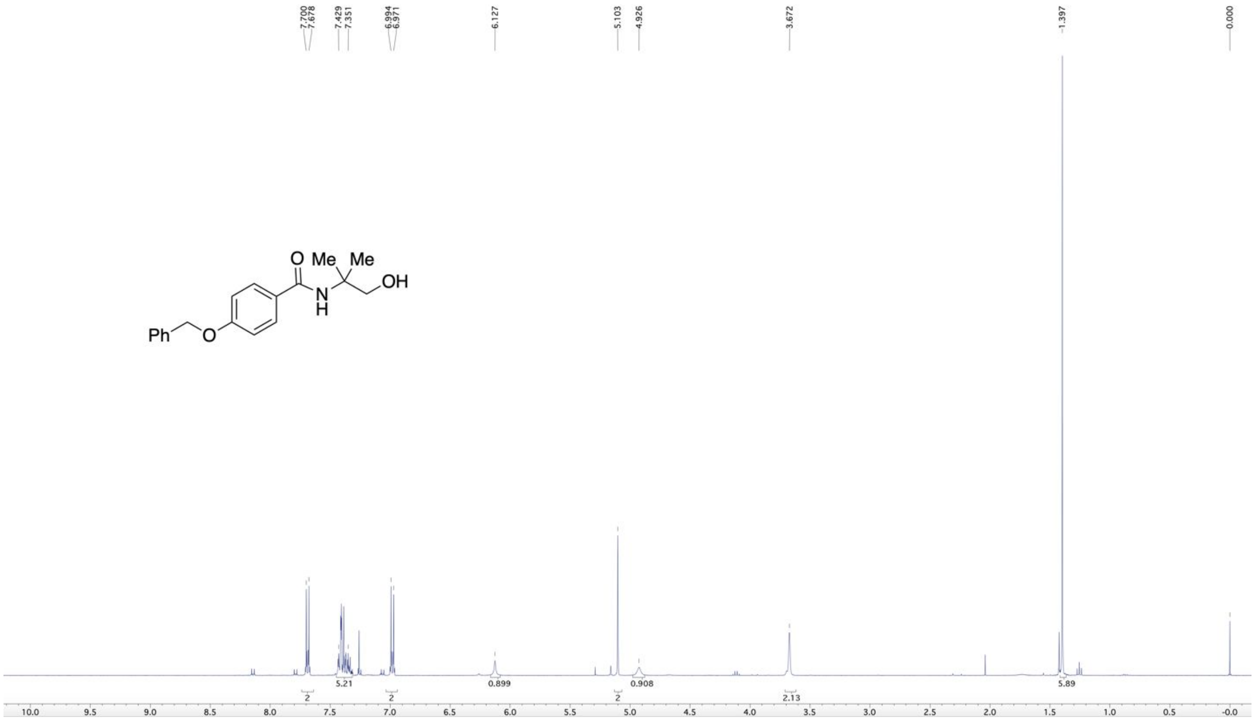

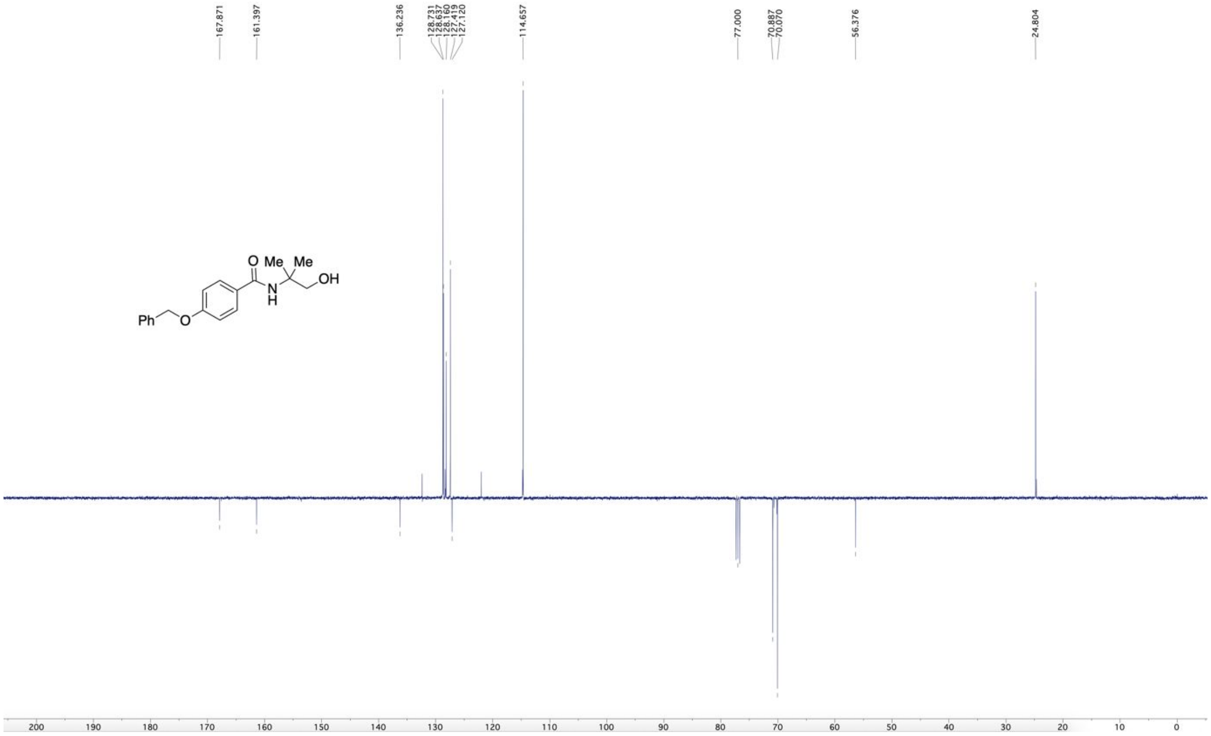

2-(4-Benzyloxy)phenyl)-4,4-dimethyl-4,5-dihydrooxazole **9**

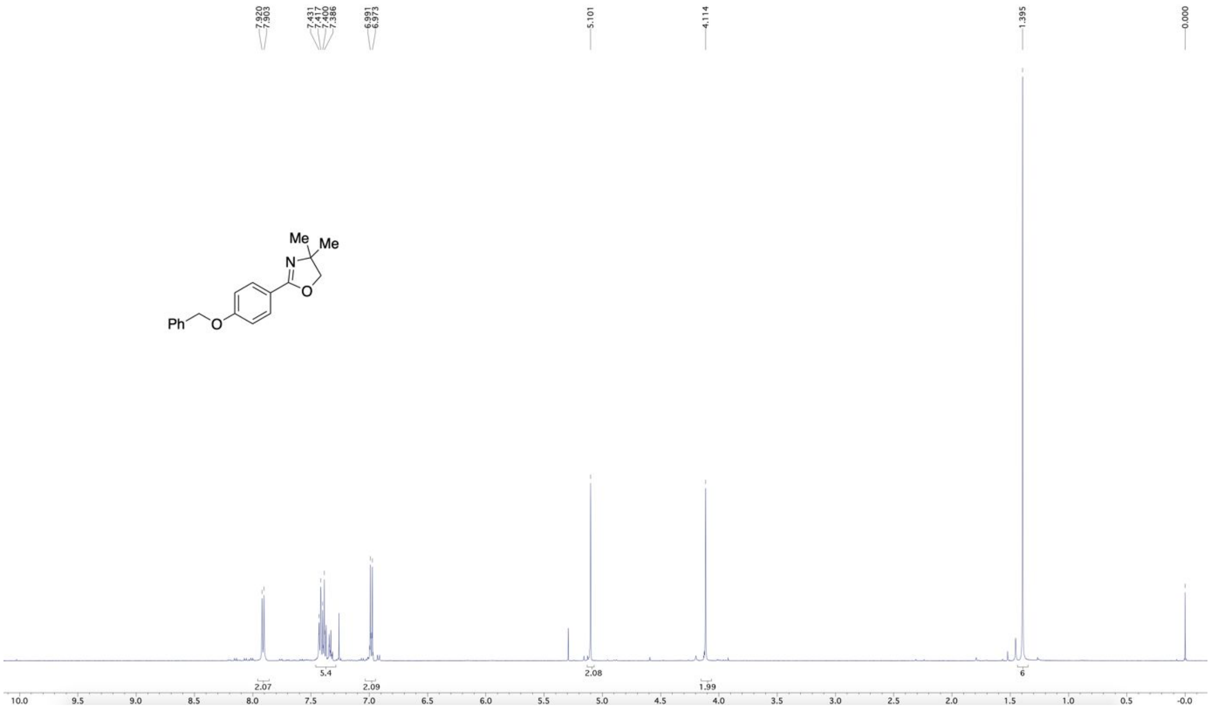

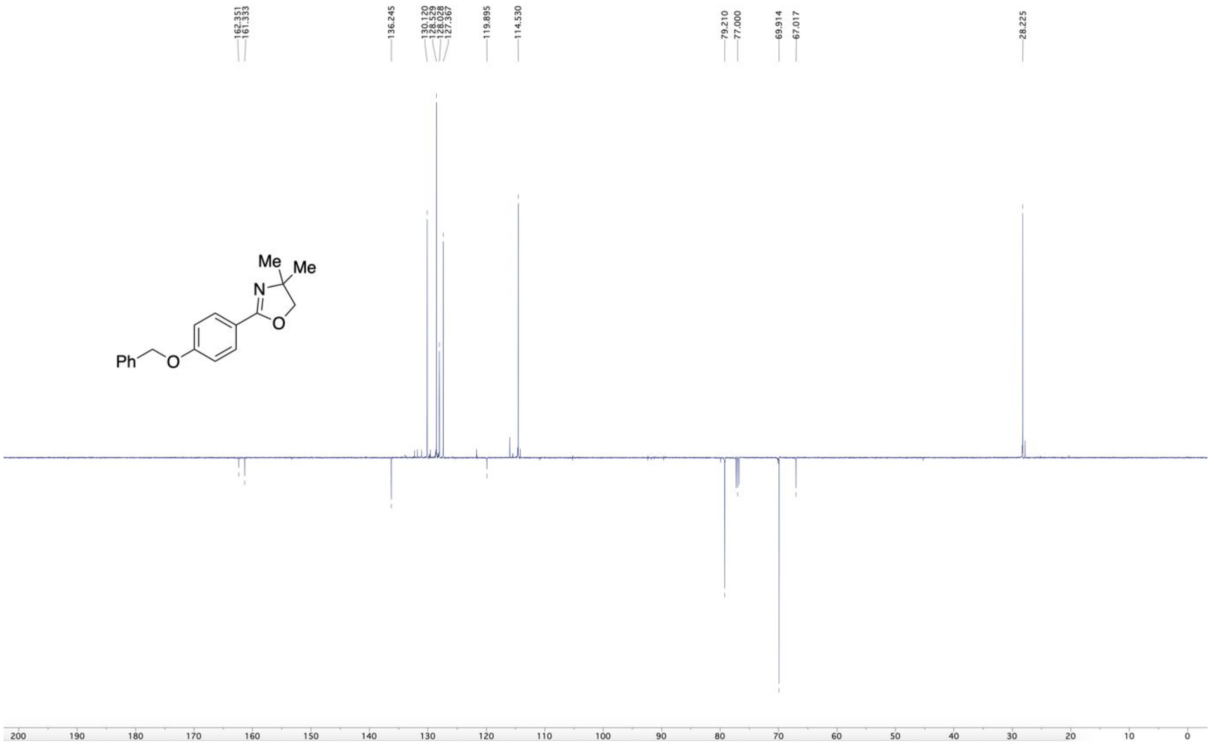

(4-(4,4-Dimethyl-4,5-dihydrooxazol-2-yl)phenyl)(phenyl)methanol **10**

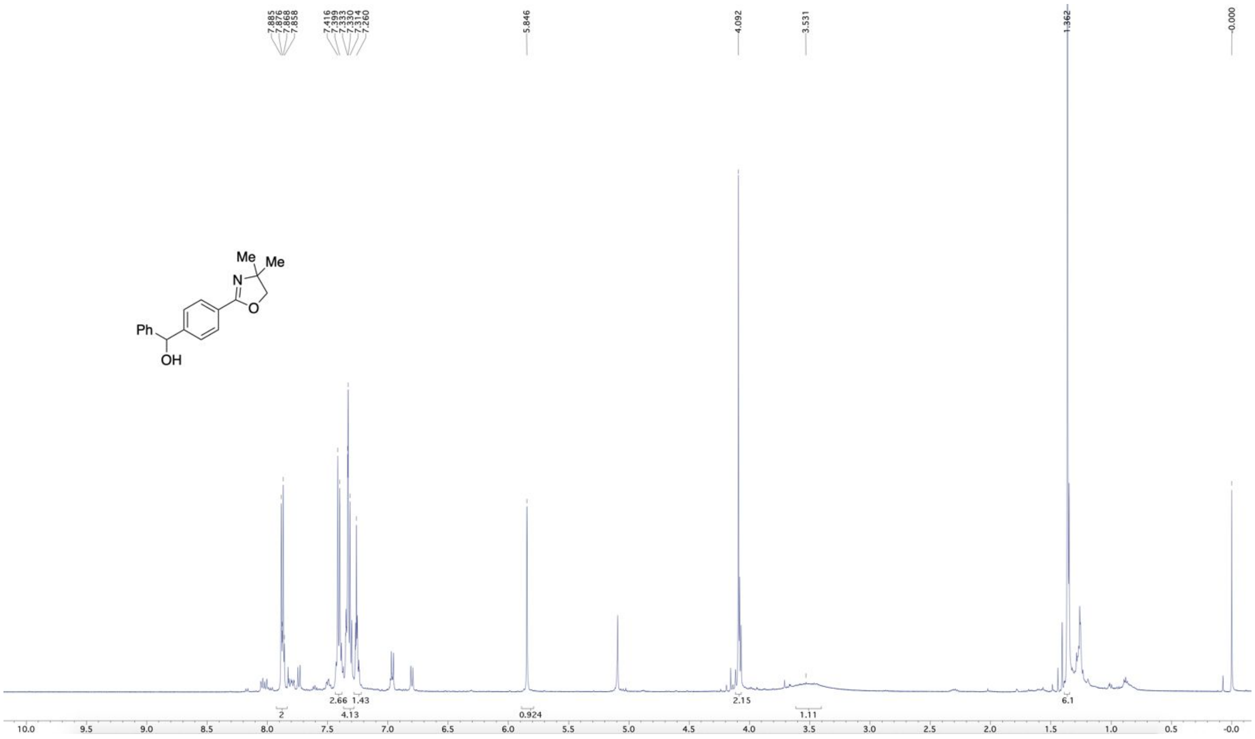

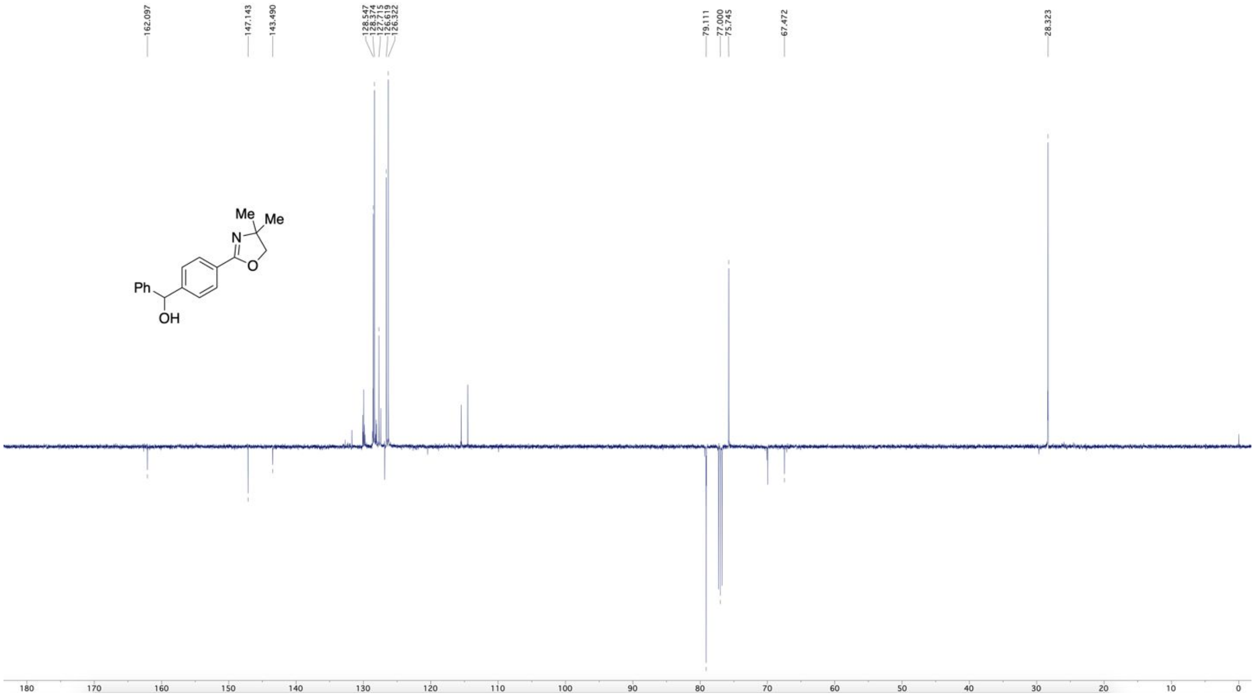

3-(Benzyloxy)-*N*-(1-hydroxy-2-methylpropan-2-yl)benzamide **C**

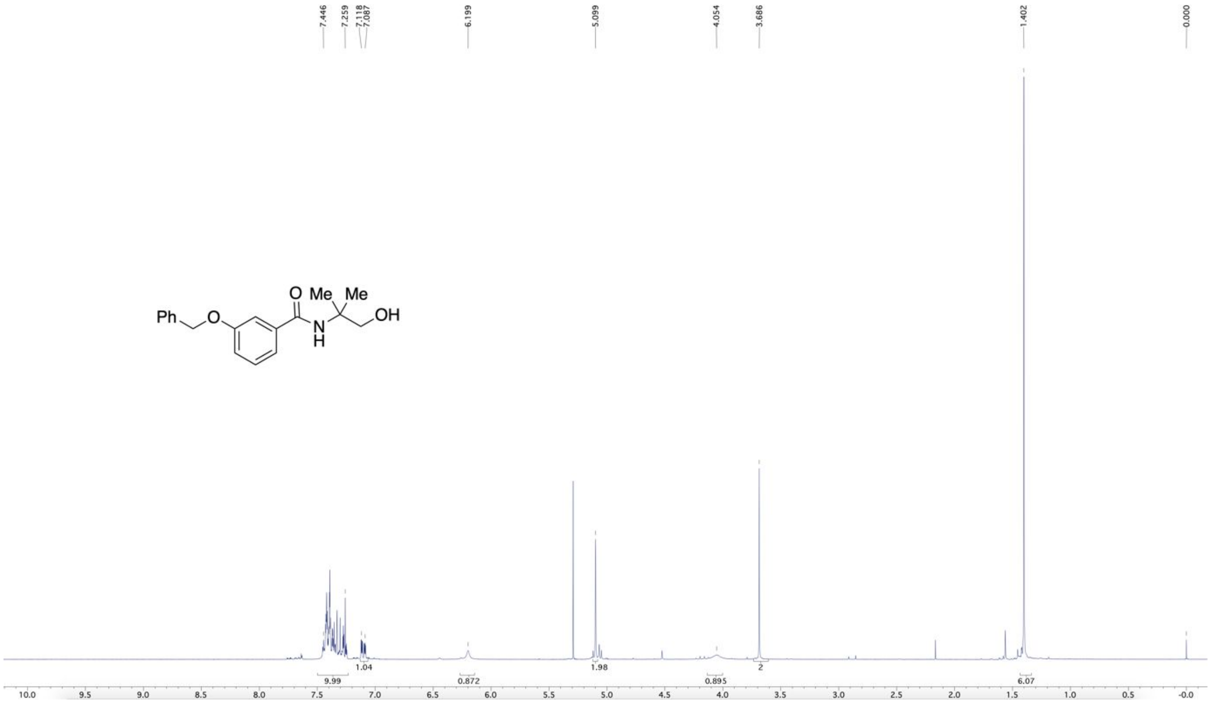

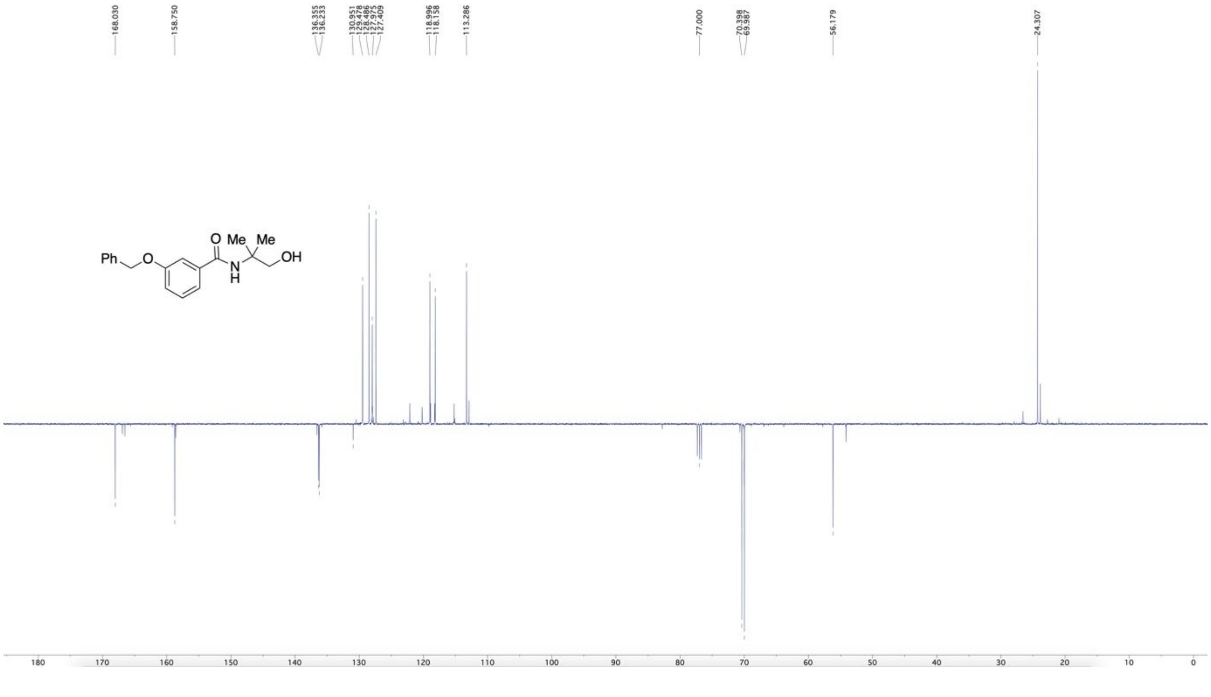

2-(3-Benzyloxy)phenyl)-4,4-dimethyl-4,5-dihydrooxazole **11**

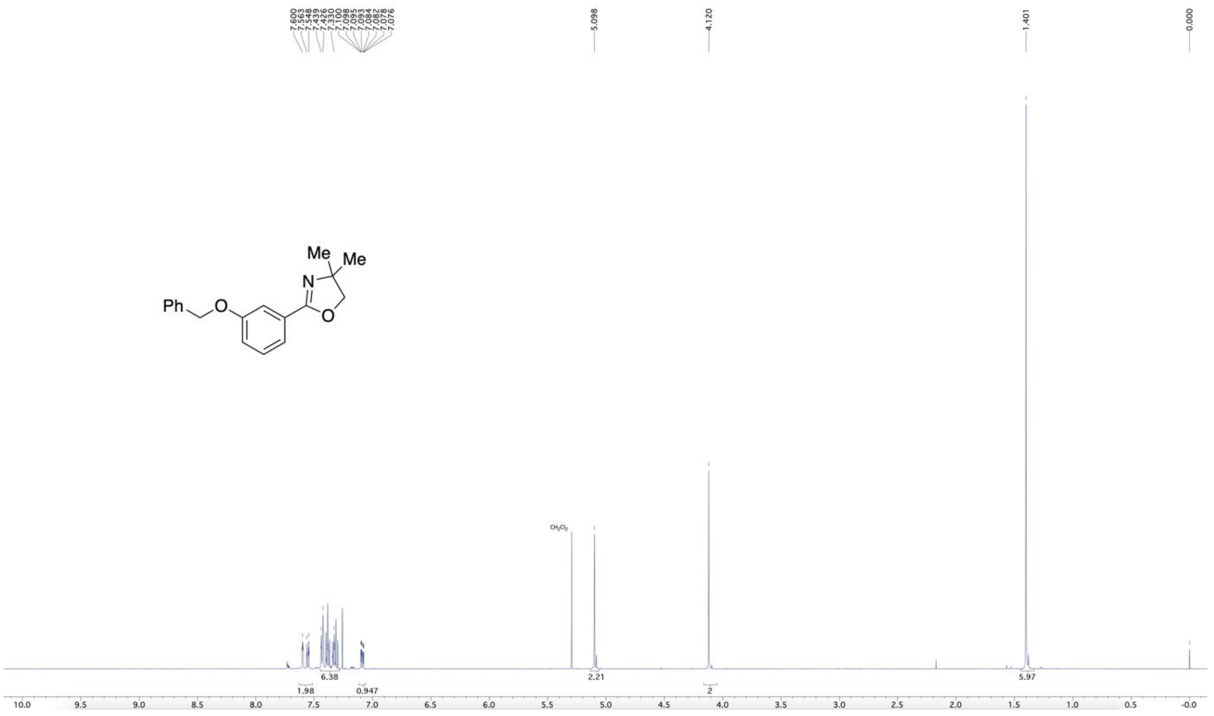

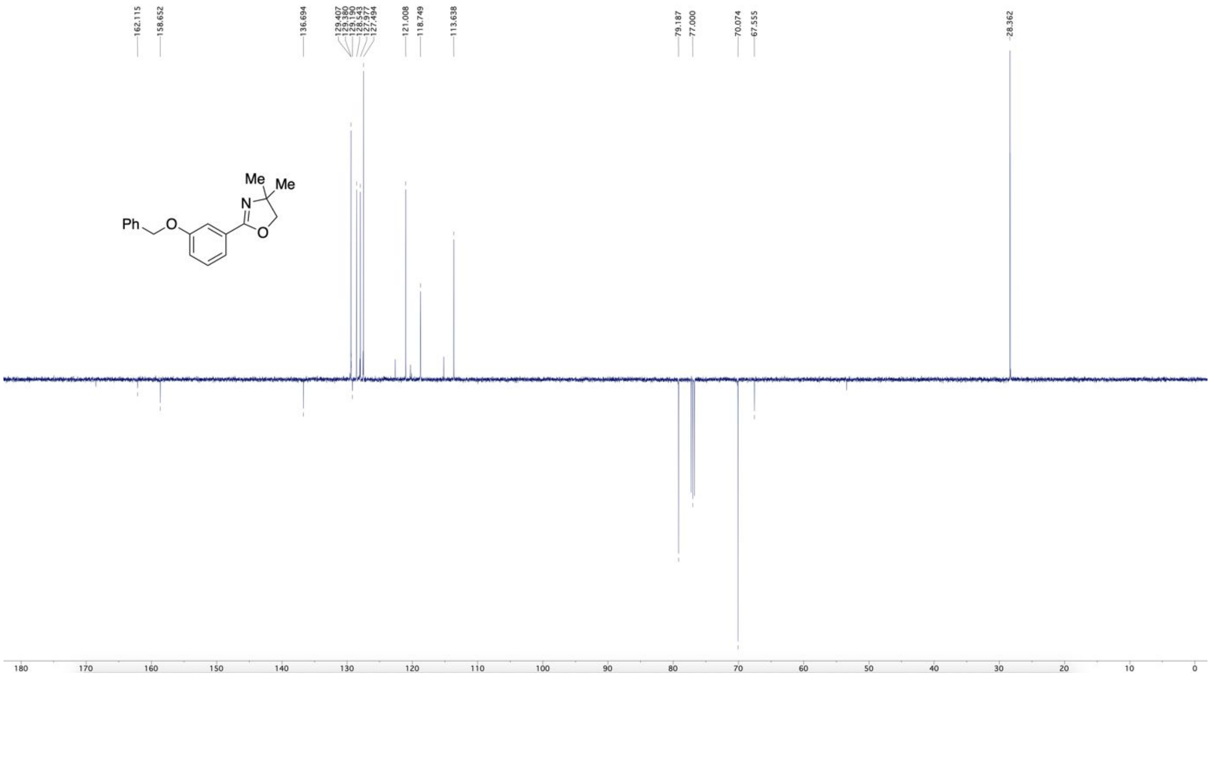

2-(Benzyloxy)phenyl *N,N*-diethylcarbamate **12**

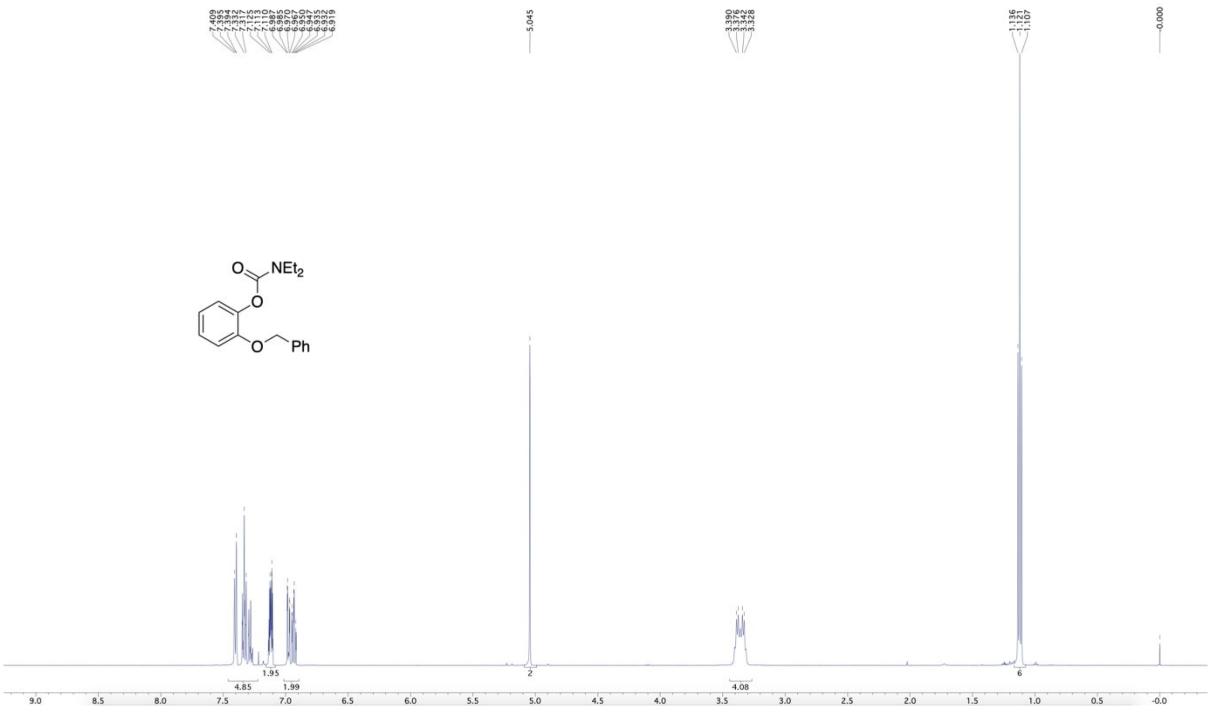

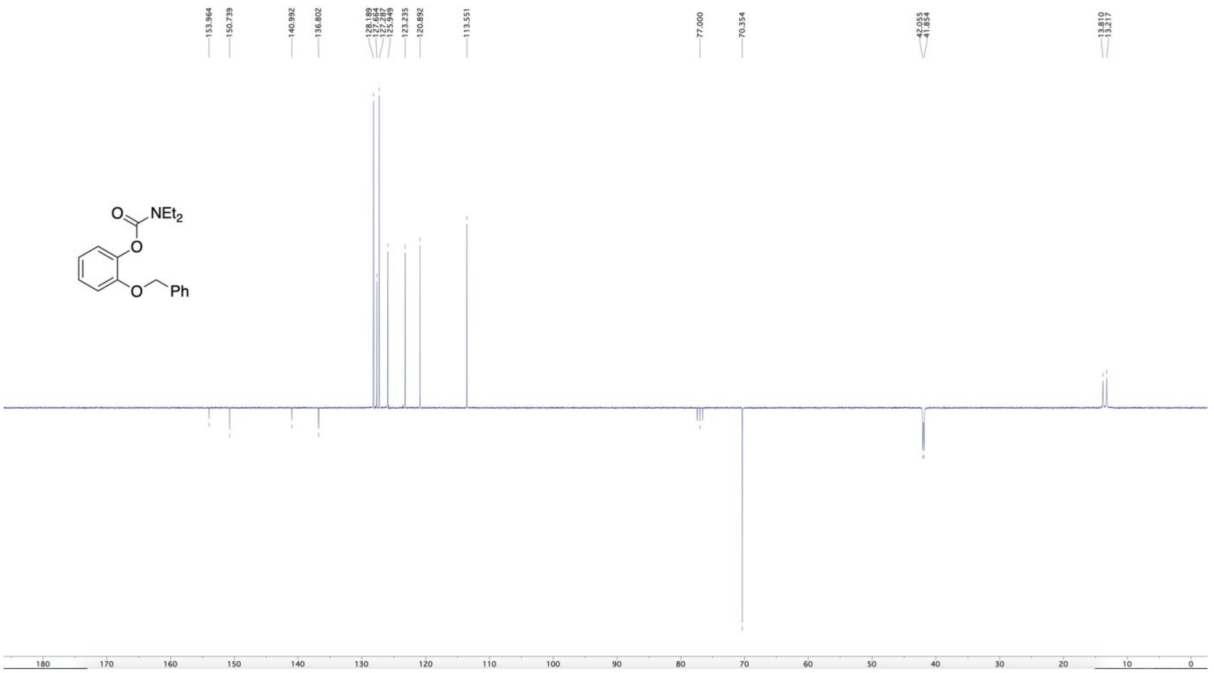

2-(Benzyloxy)phenyl *N*-phenylcarbamate **15**

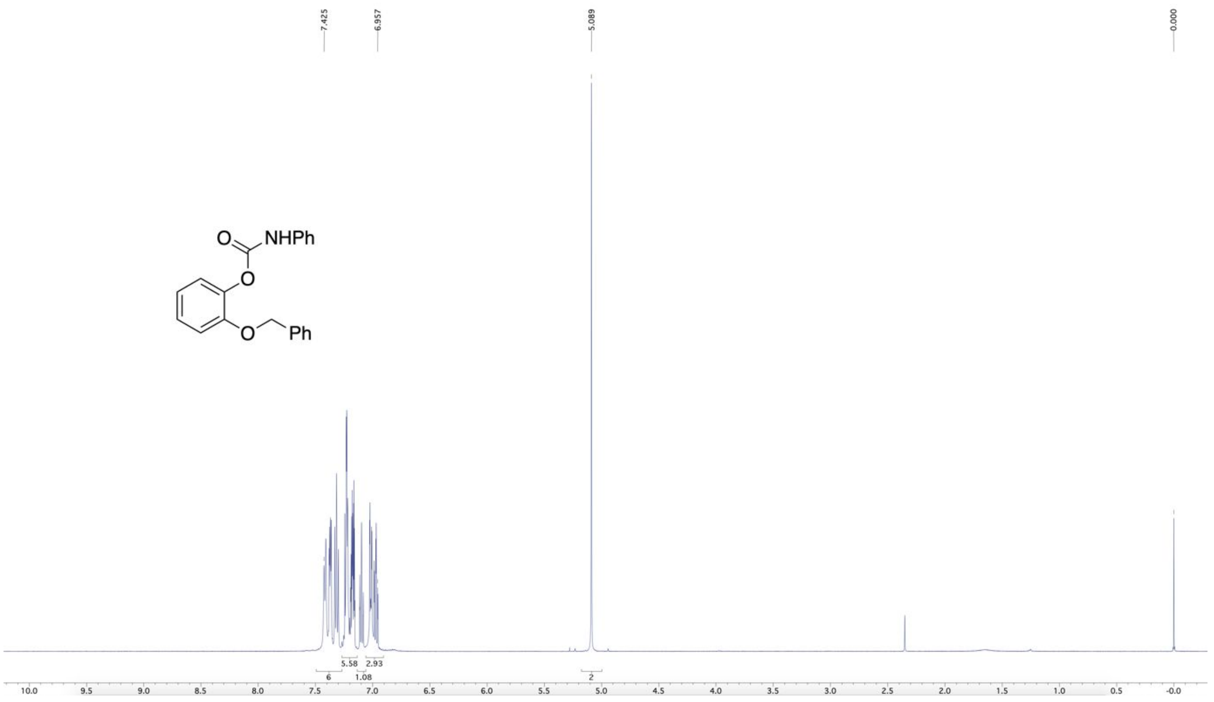

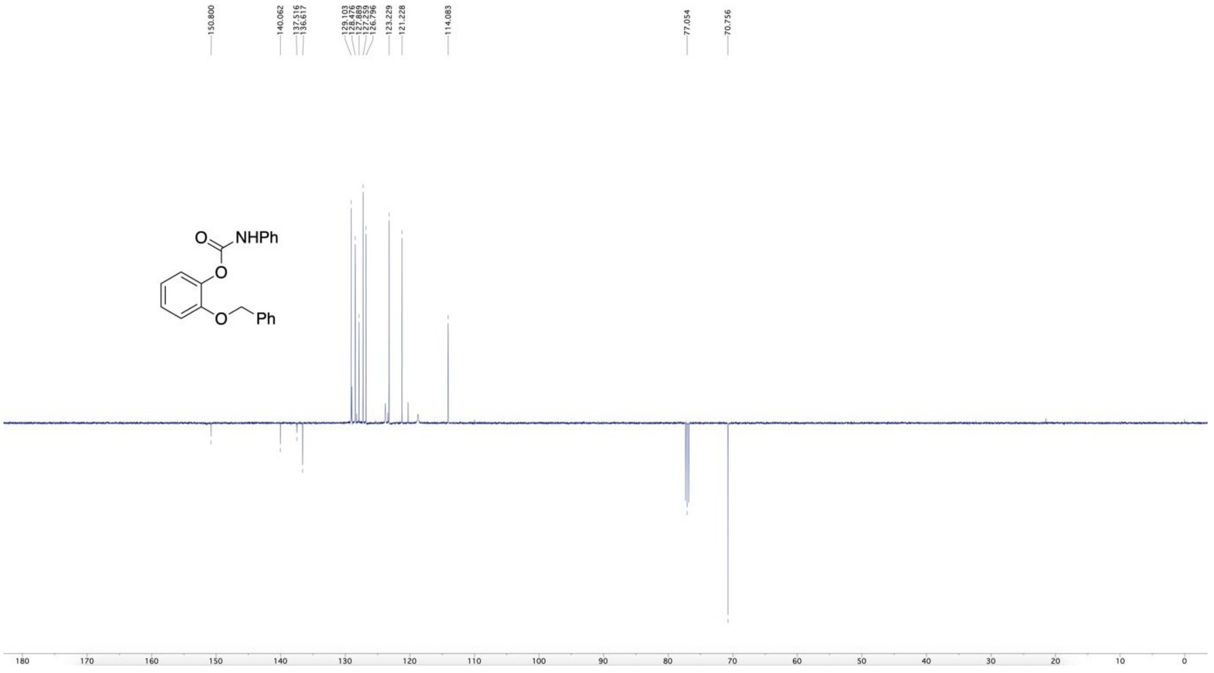

2-(Benzyloxy)-*N,N*-diethylbenzamide **16**

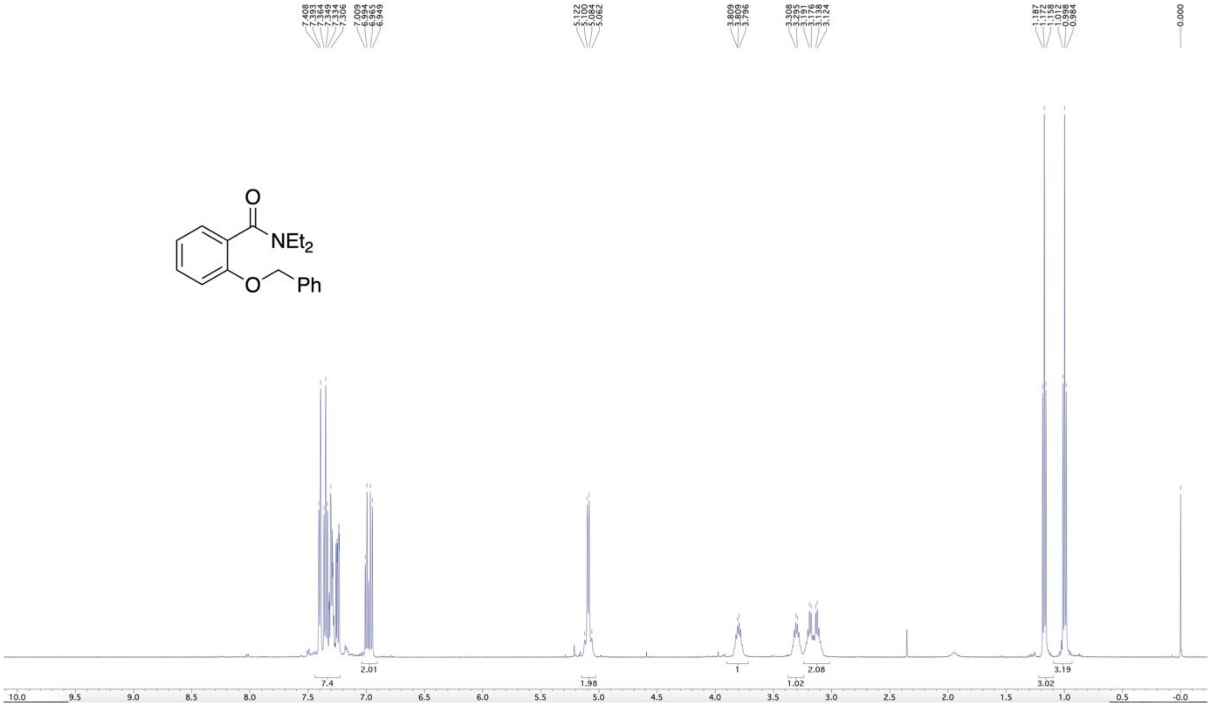

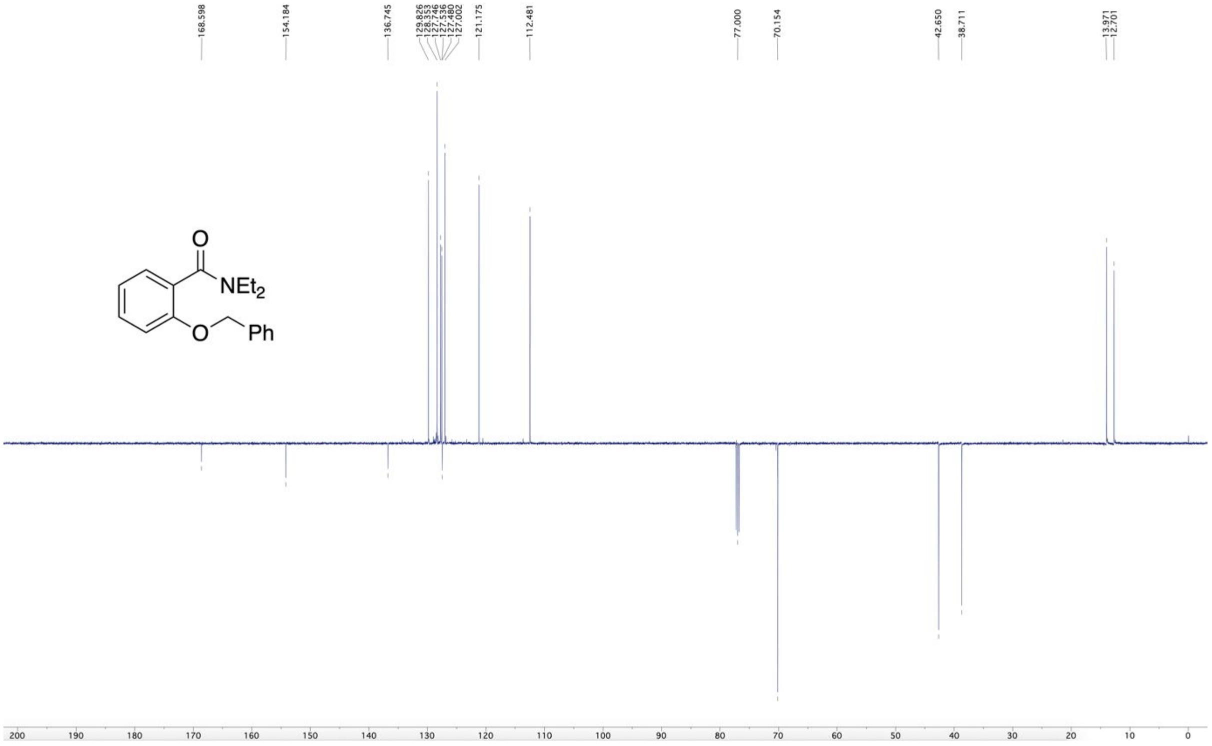

2-(Benzyloxy)-*N,N*-diisopropylbenzamide **18**

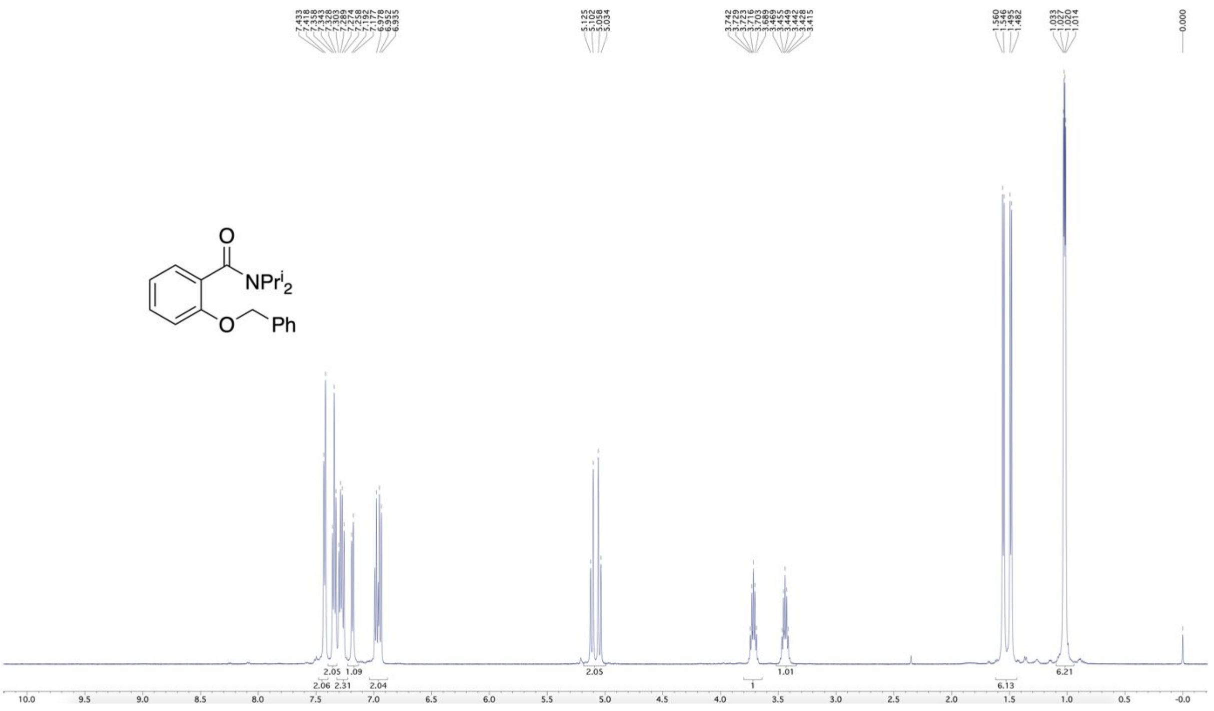

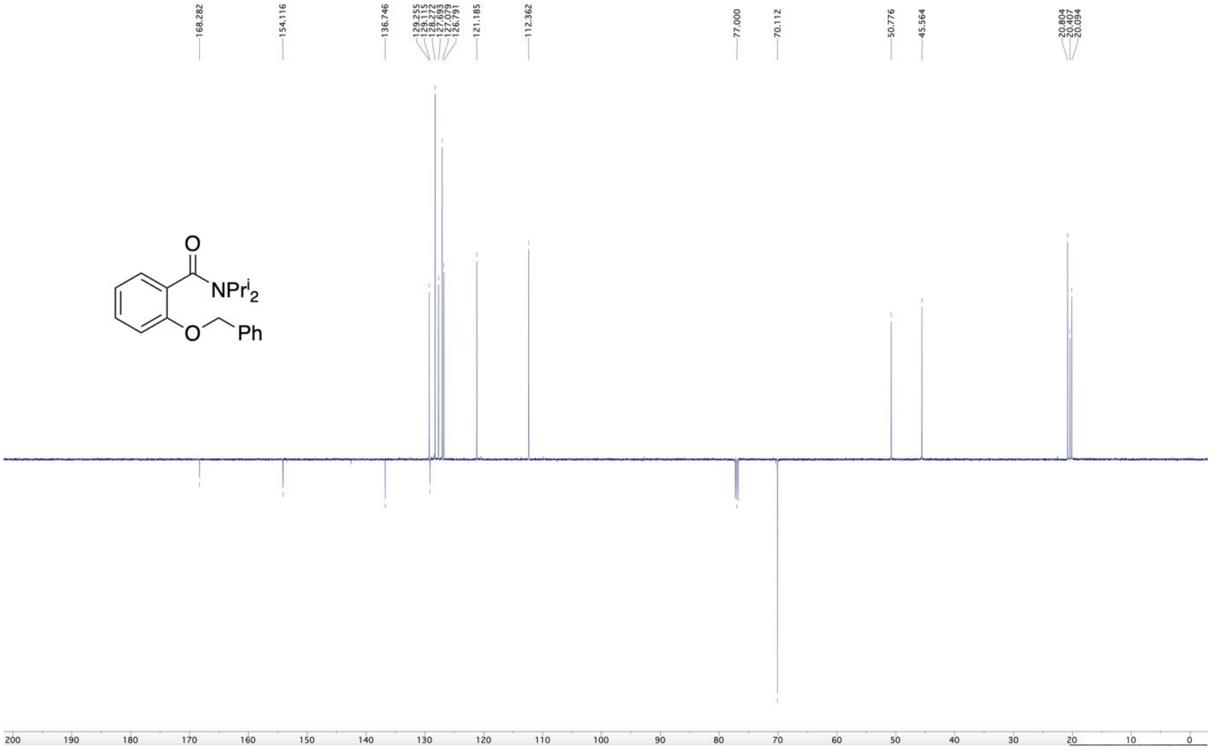

N,N-diisopropyl-2-phenylbenzofuran-3-amine **20**

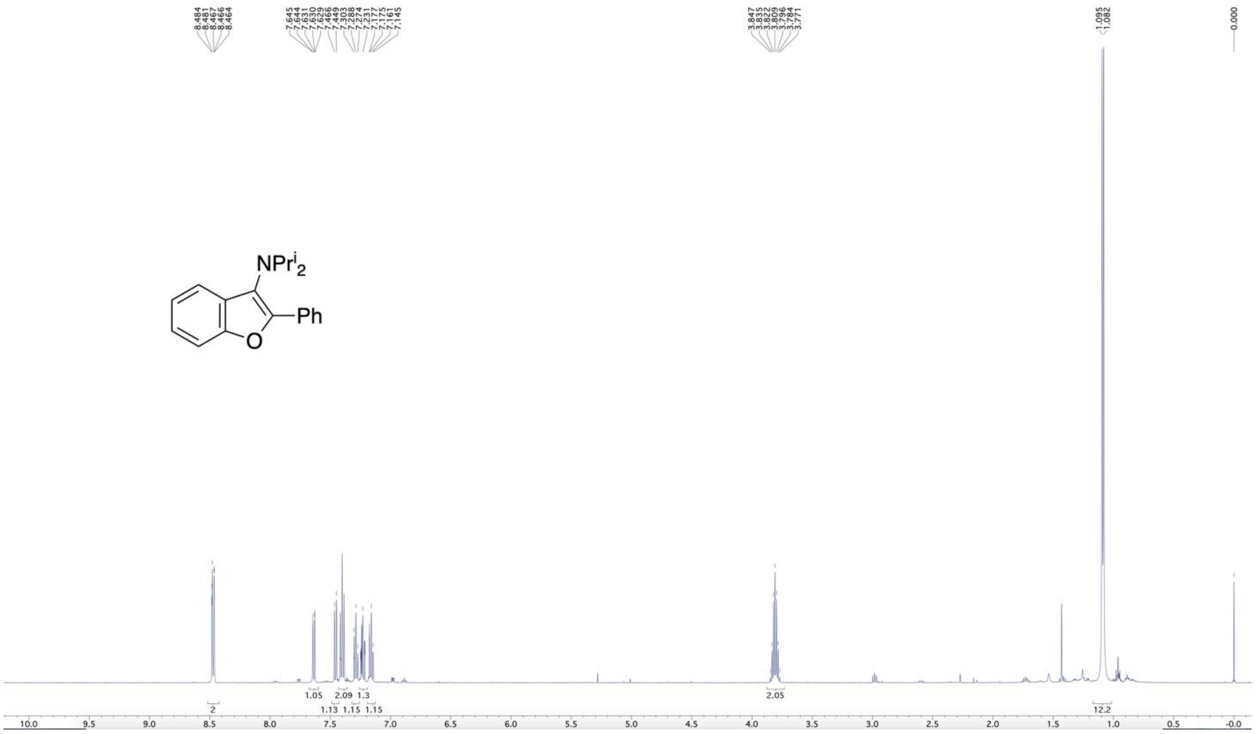

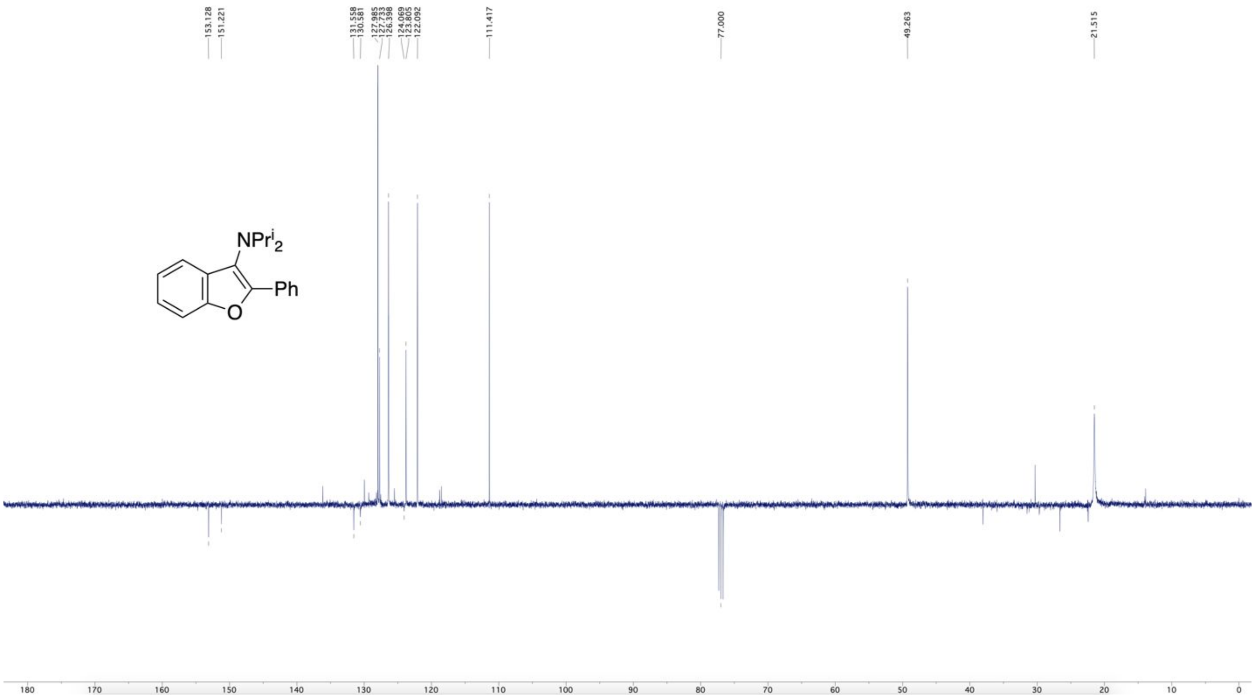

2-(Benzyloxy)-*N*-butylbenzamide **21a**

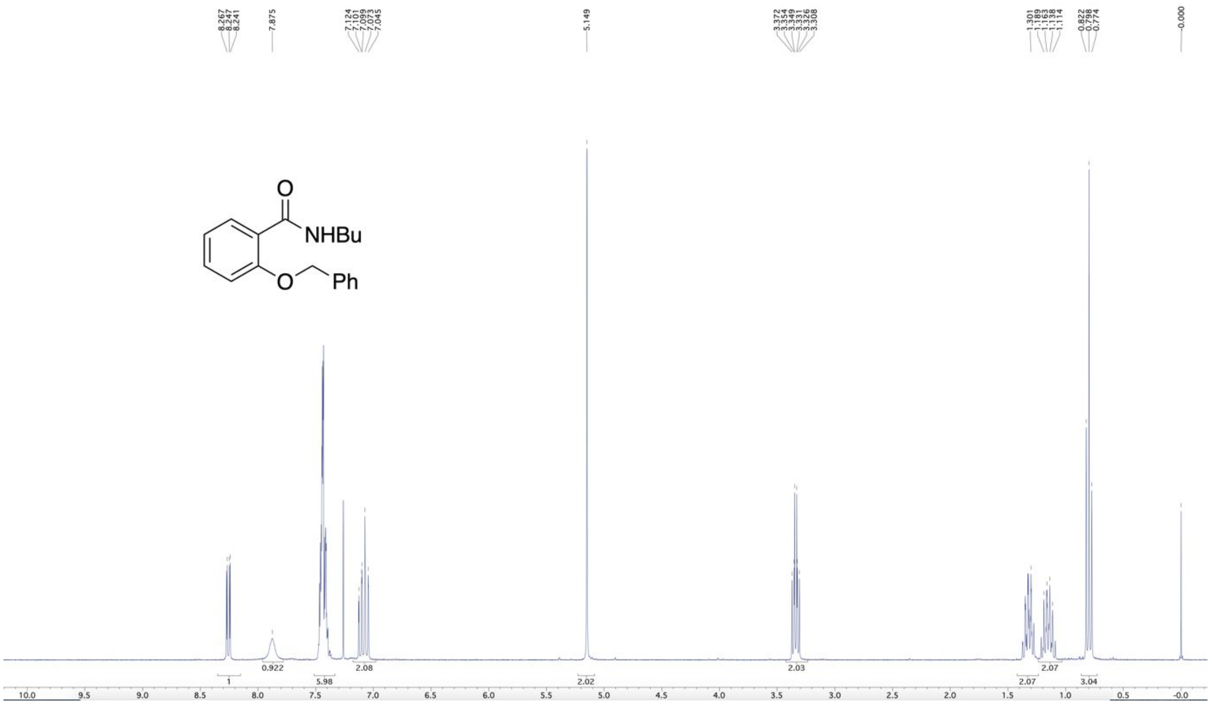

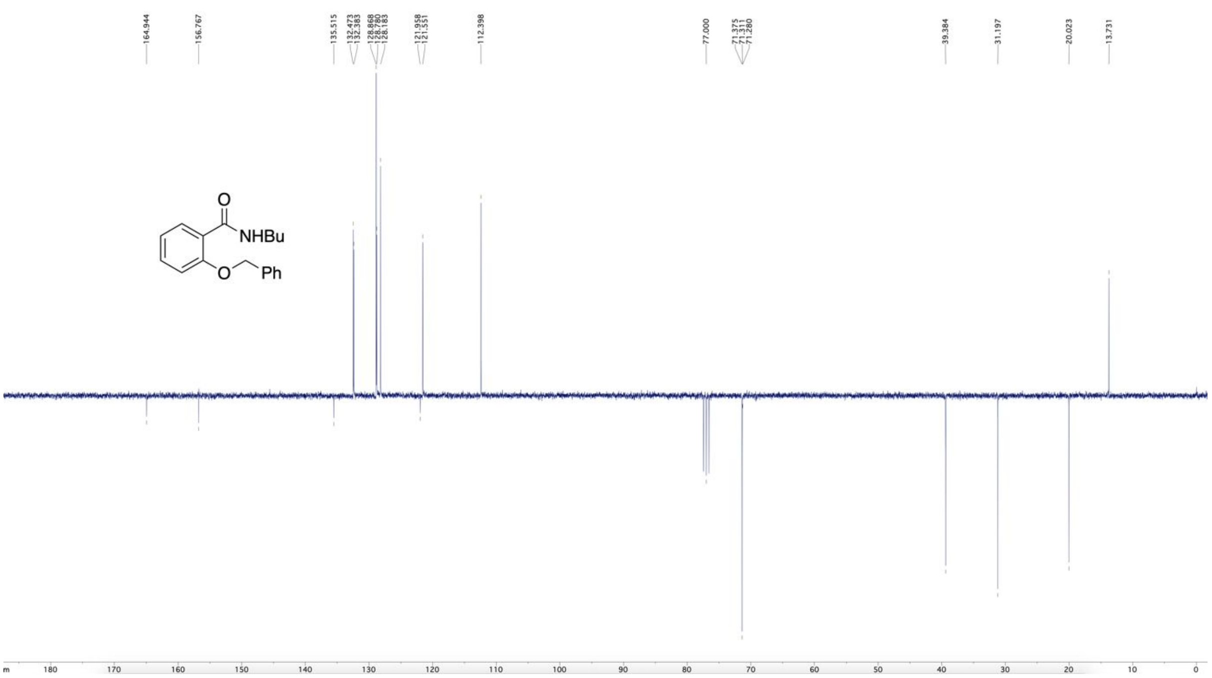

N-Butyl-2-((4-methylbenzyl)oxy)benzamide **21b**

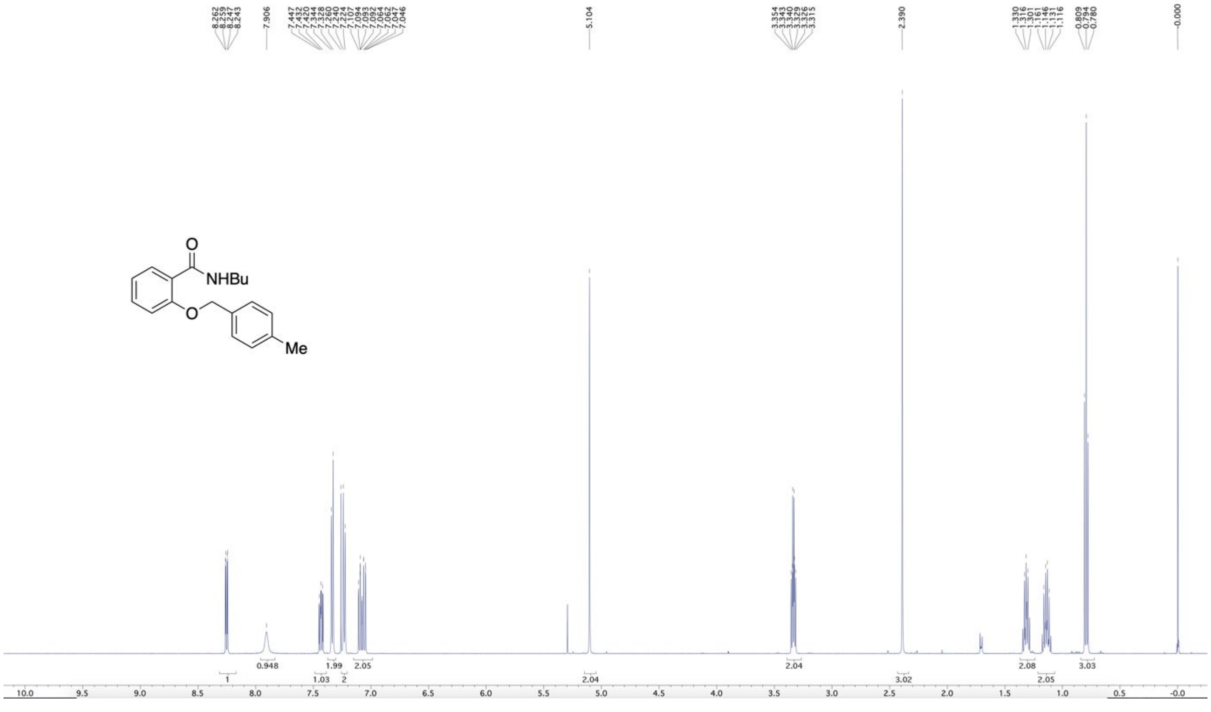

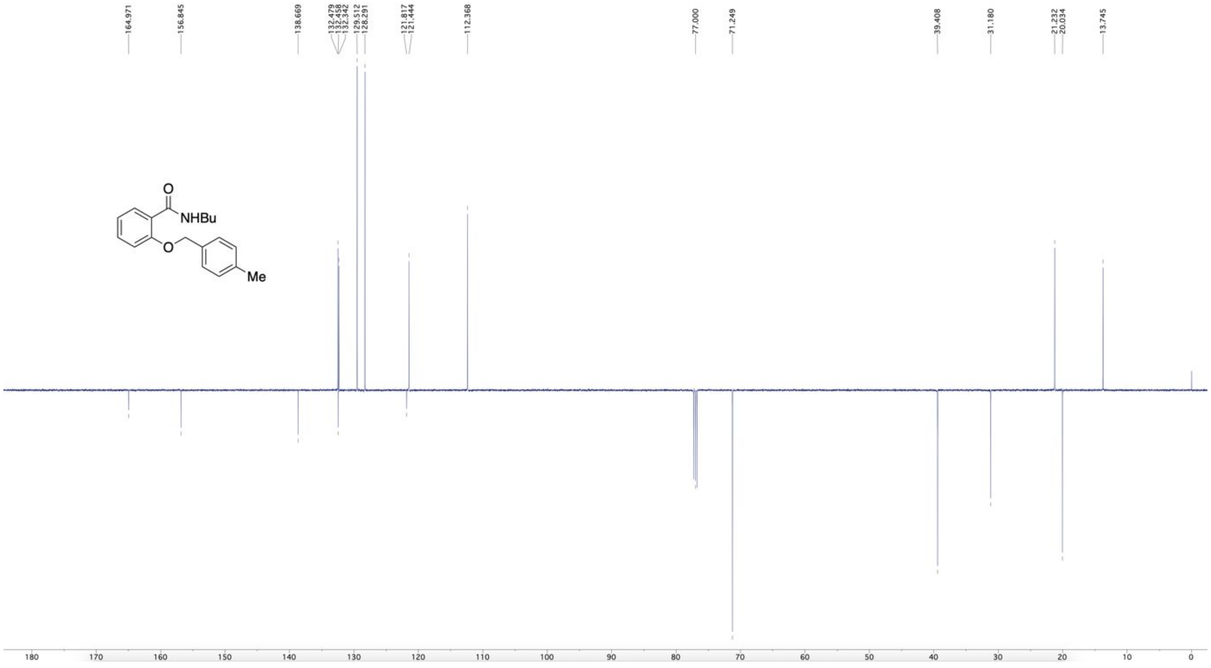

N-Butyl-2-((2-methylbenzyl)oxy)benzamide **21c**

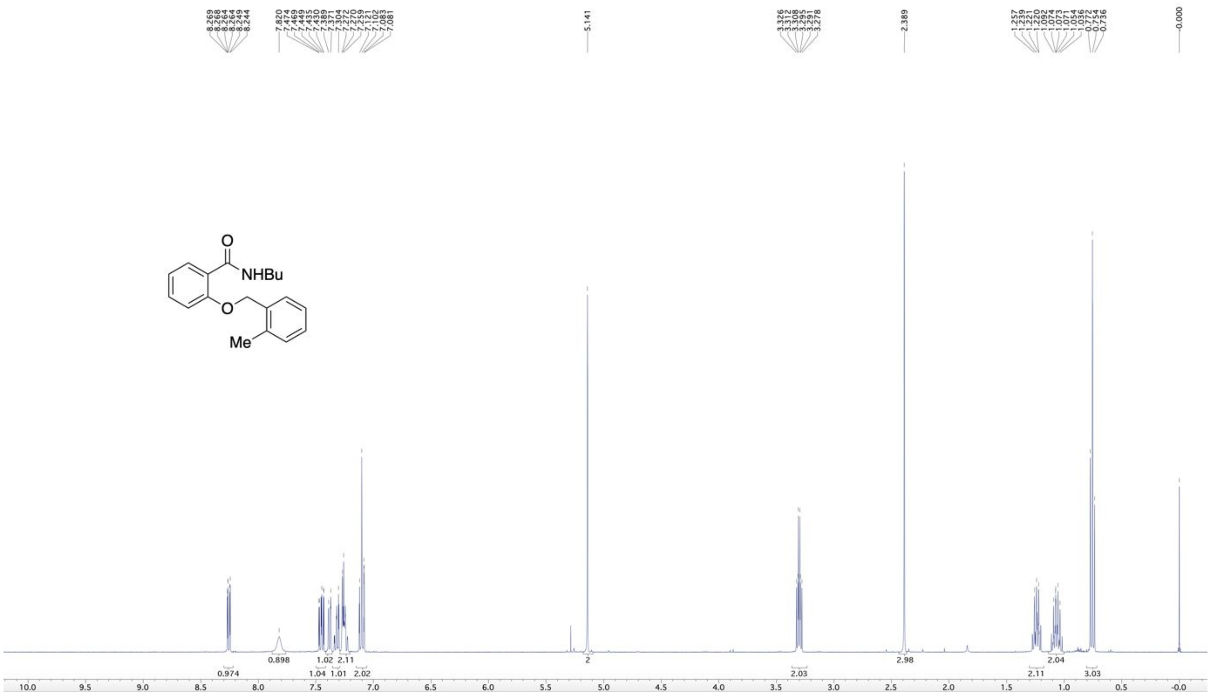

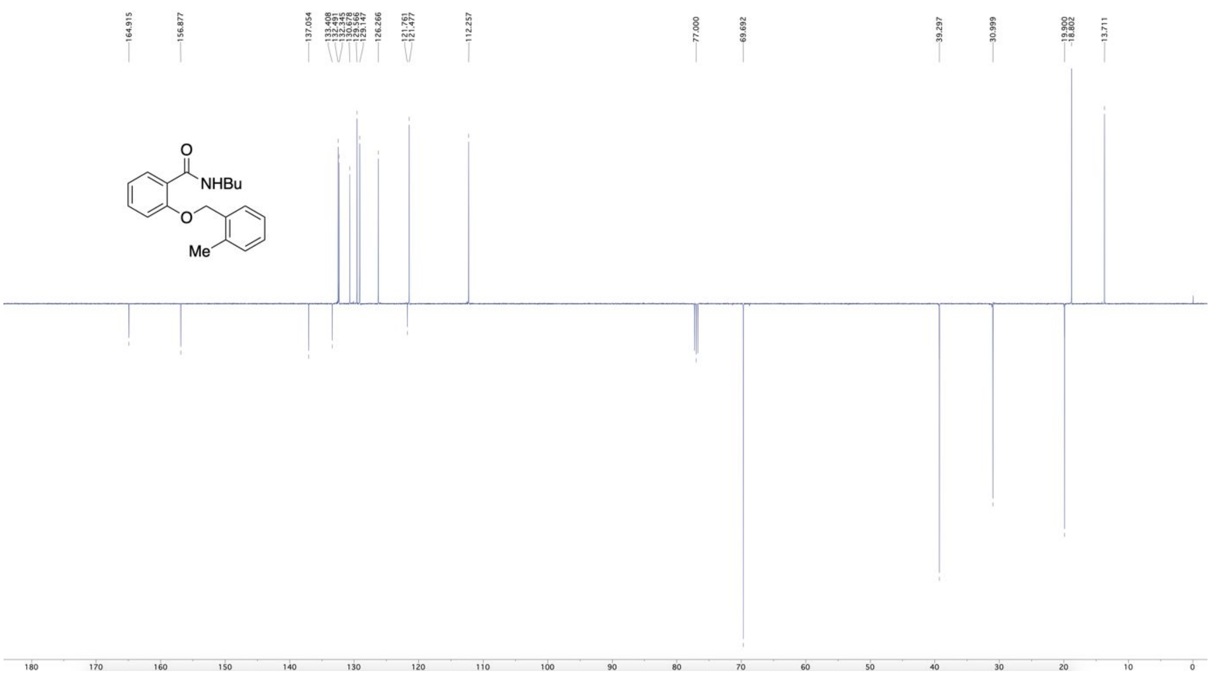

N-Butyl-2-((4-methoxybenzyl)oxy)benzamide **21d**

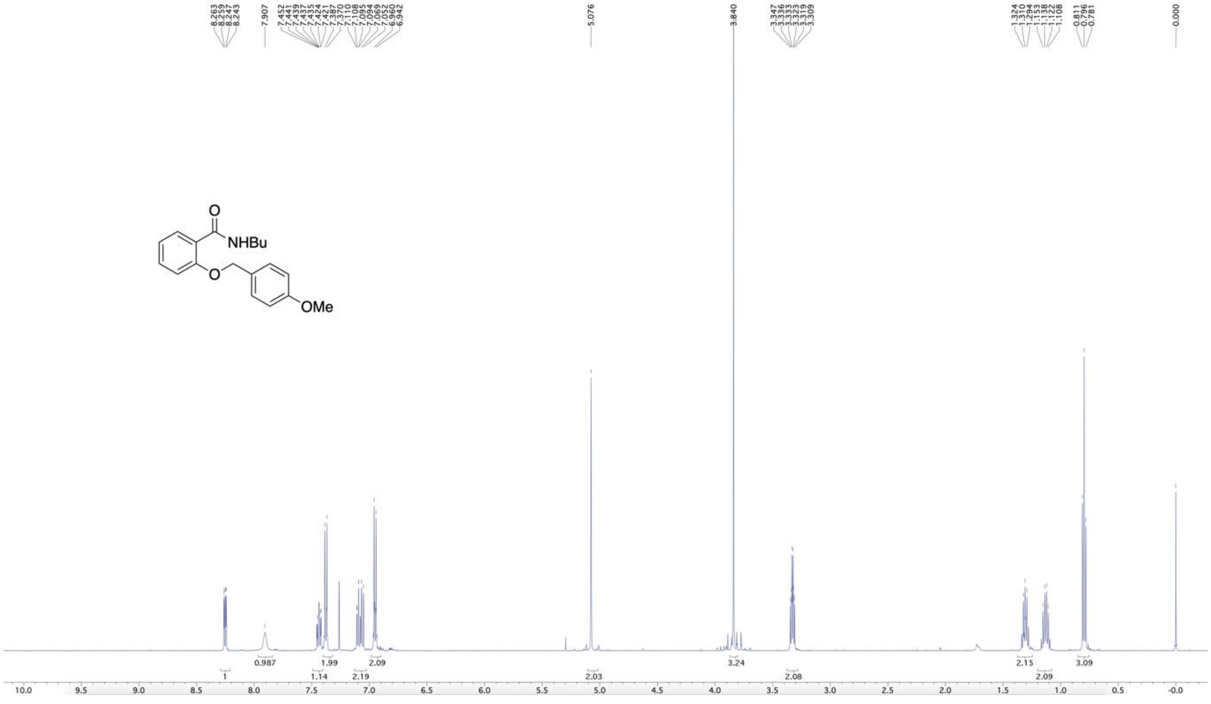

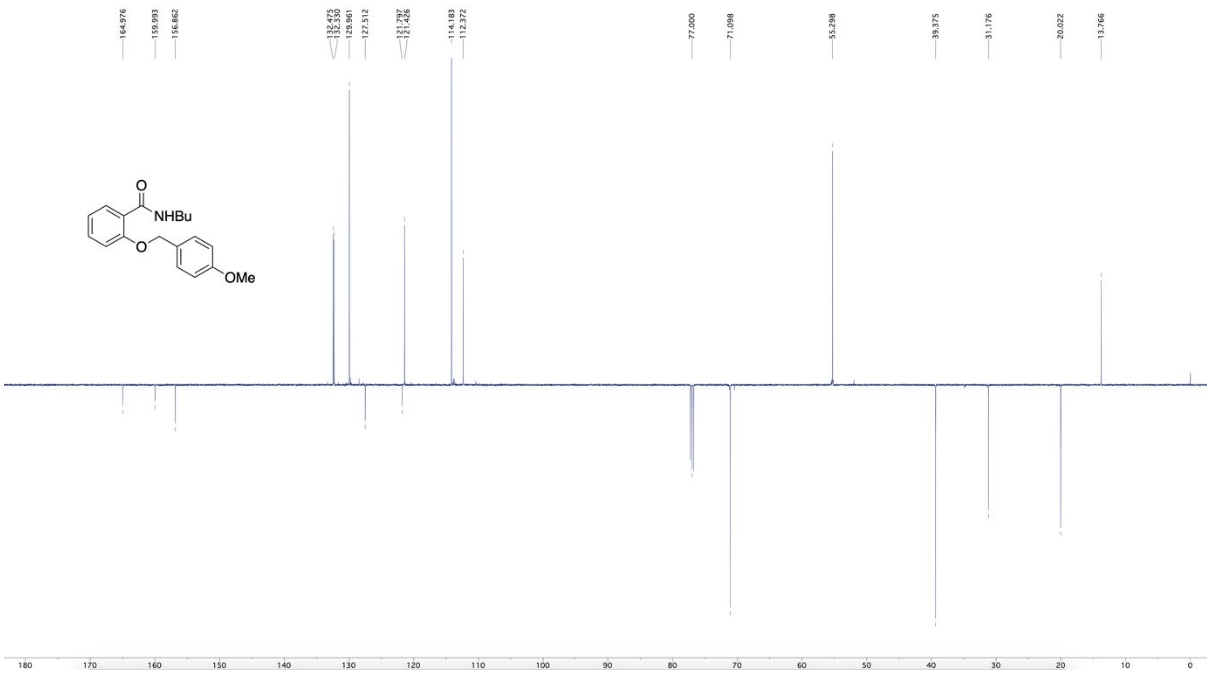

N-Butyl-2-((3-methoxybenzyl)oxy)benzamide **21e**

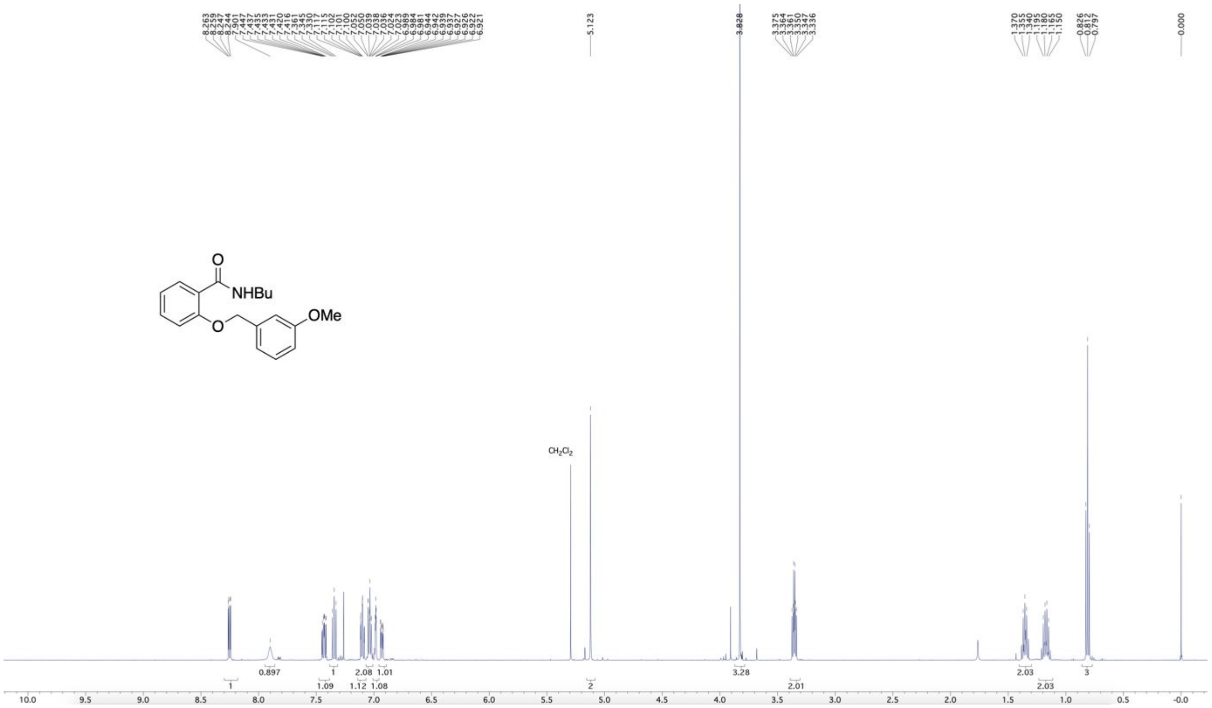

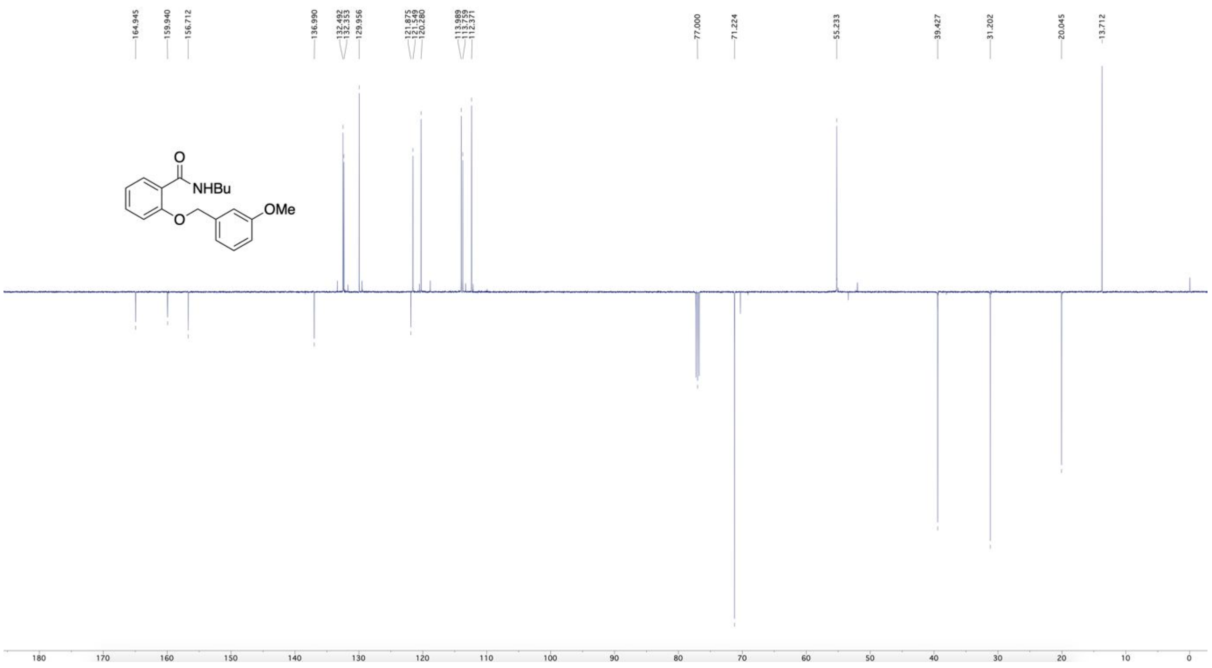

N-Butyl-2-((2-methoxybenzyl)oxy)benzamide **21f**

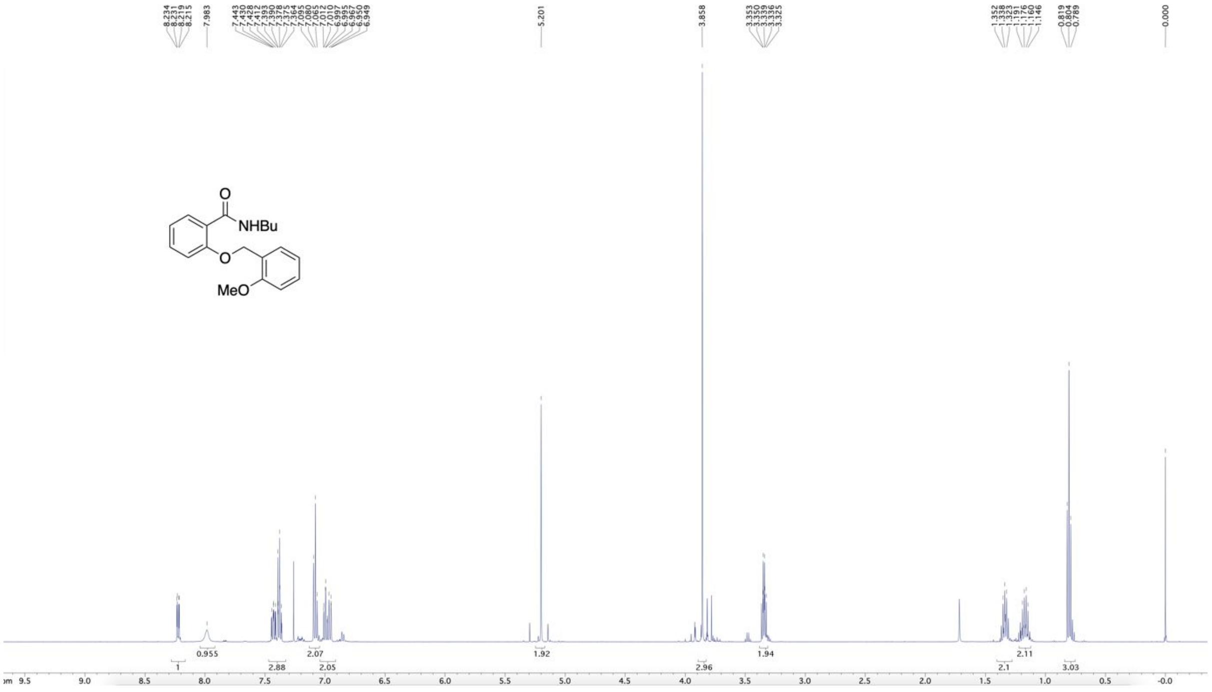

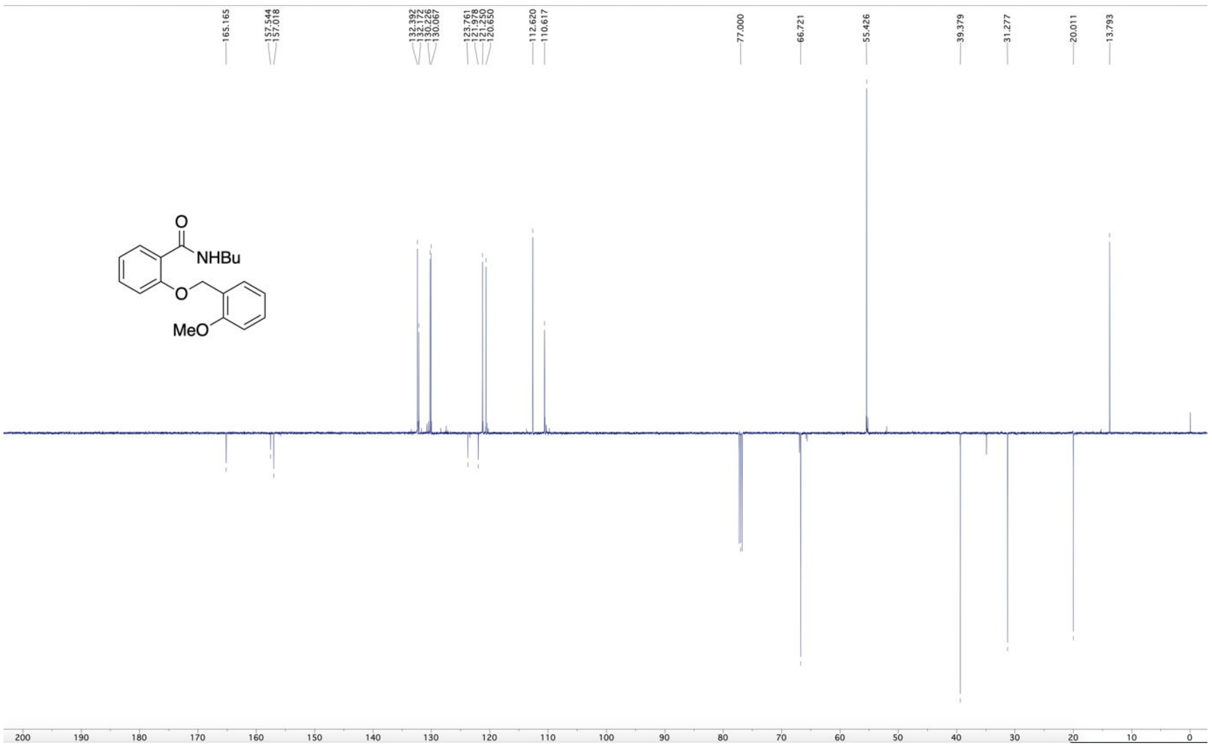

N-Butyl-2-((2-methoxynaphthalen-1-yl)methoxy)benzamide **21g**

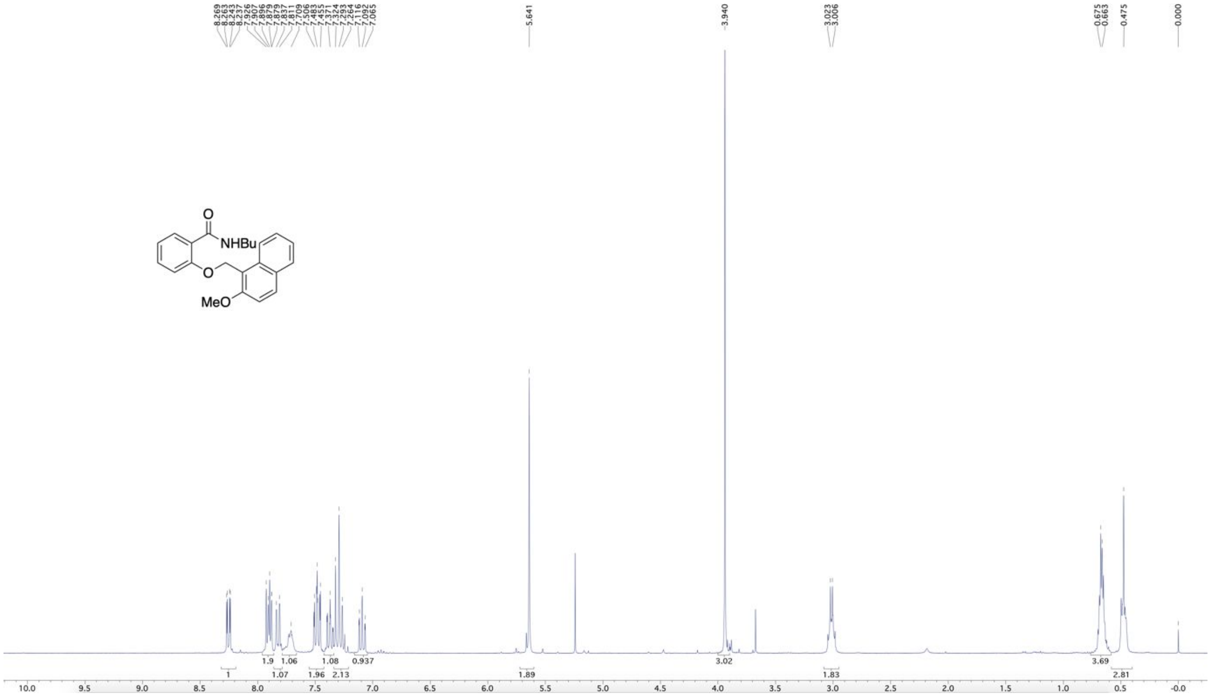

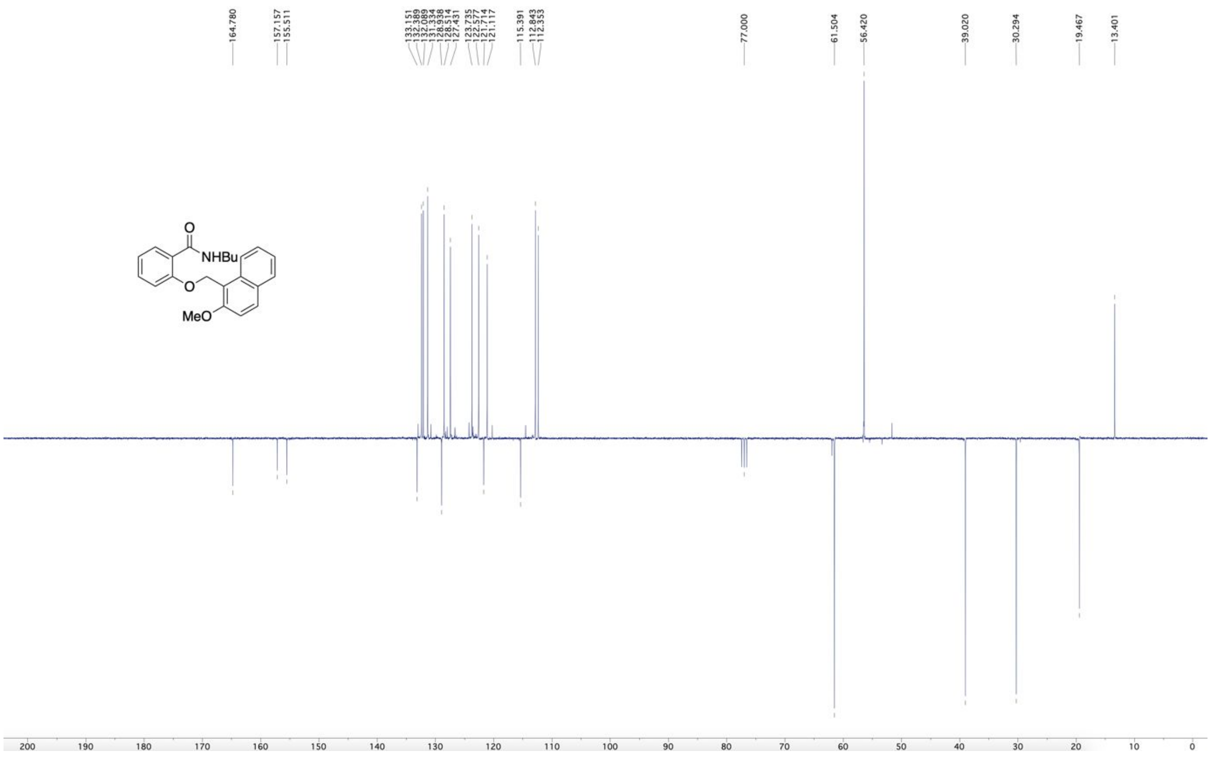

N-Butyl-2-((4-fluorobenzyl)oxy)benzamide **21h**

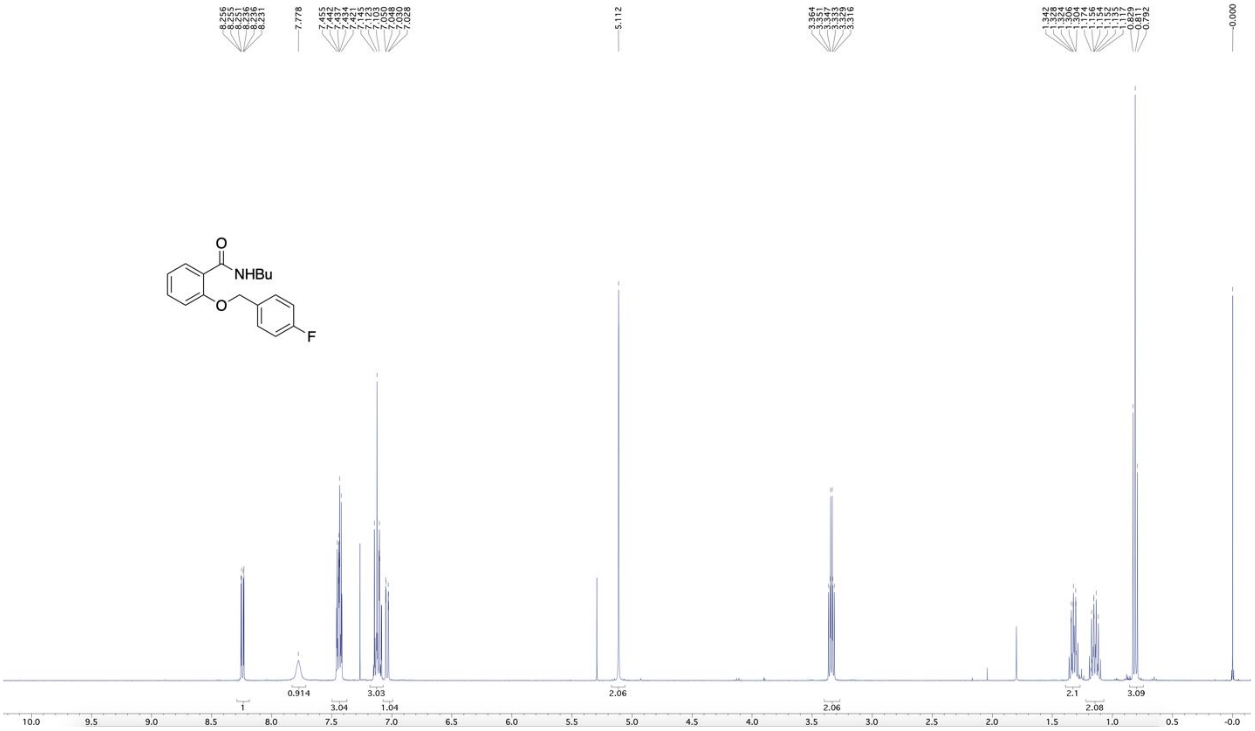

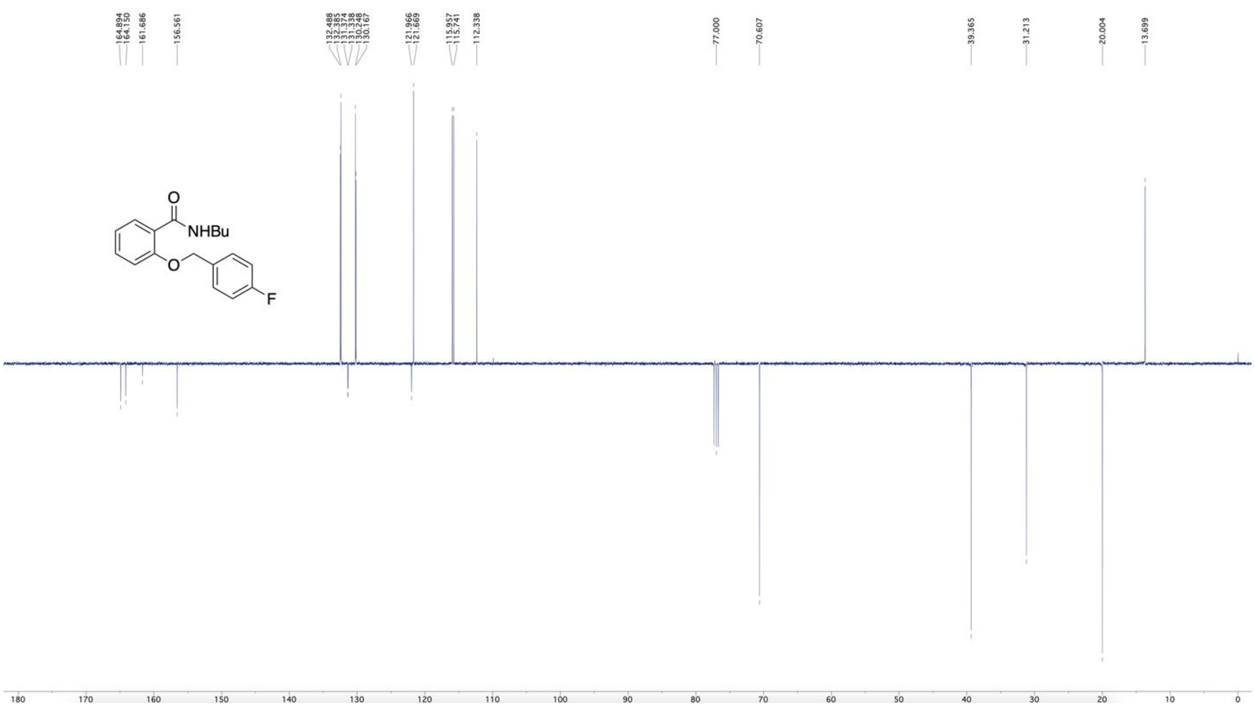

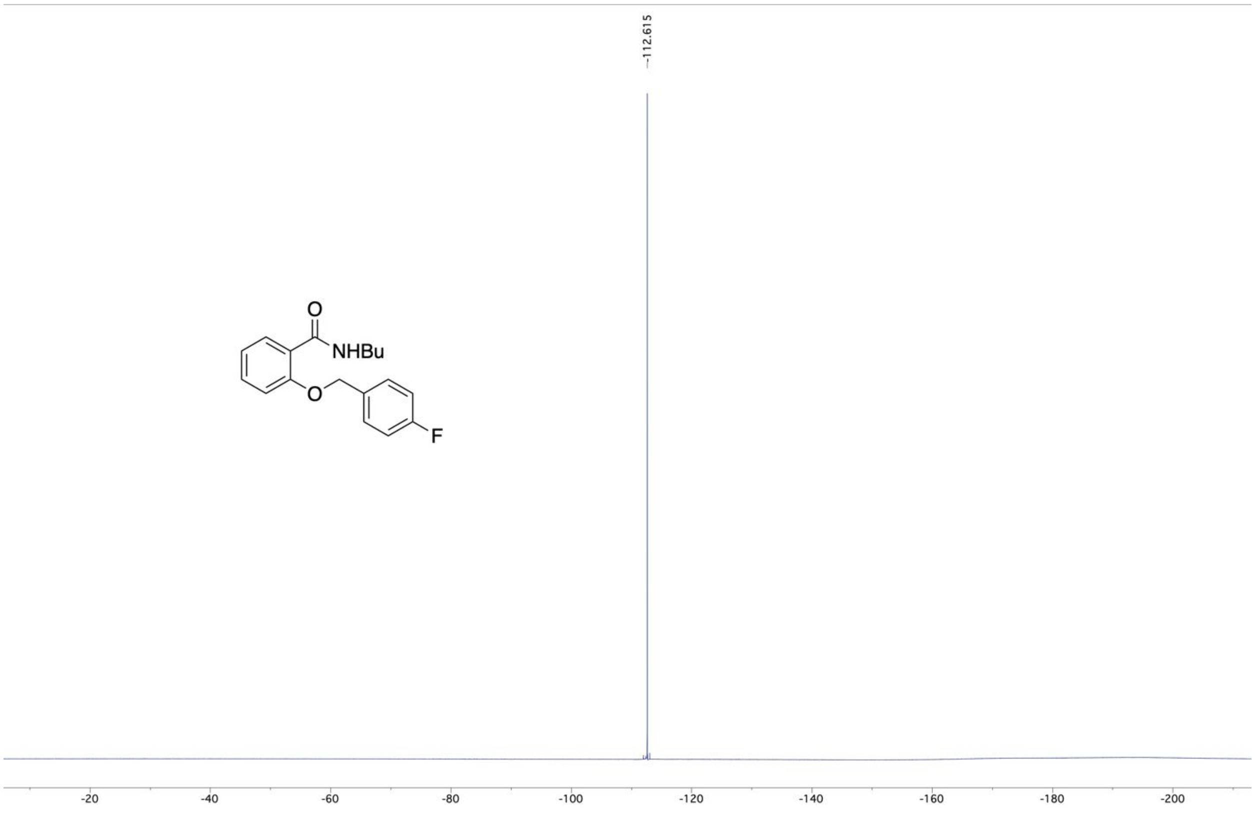

N-Butyl-2-((3-fluorobenzyl)oxy)benzamide **21i**

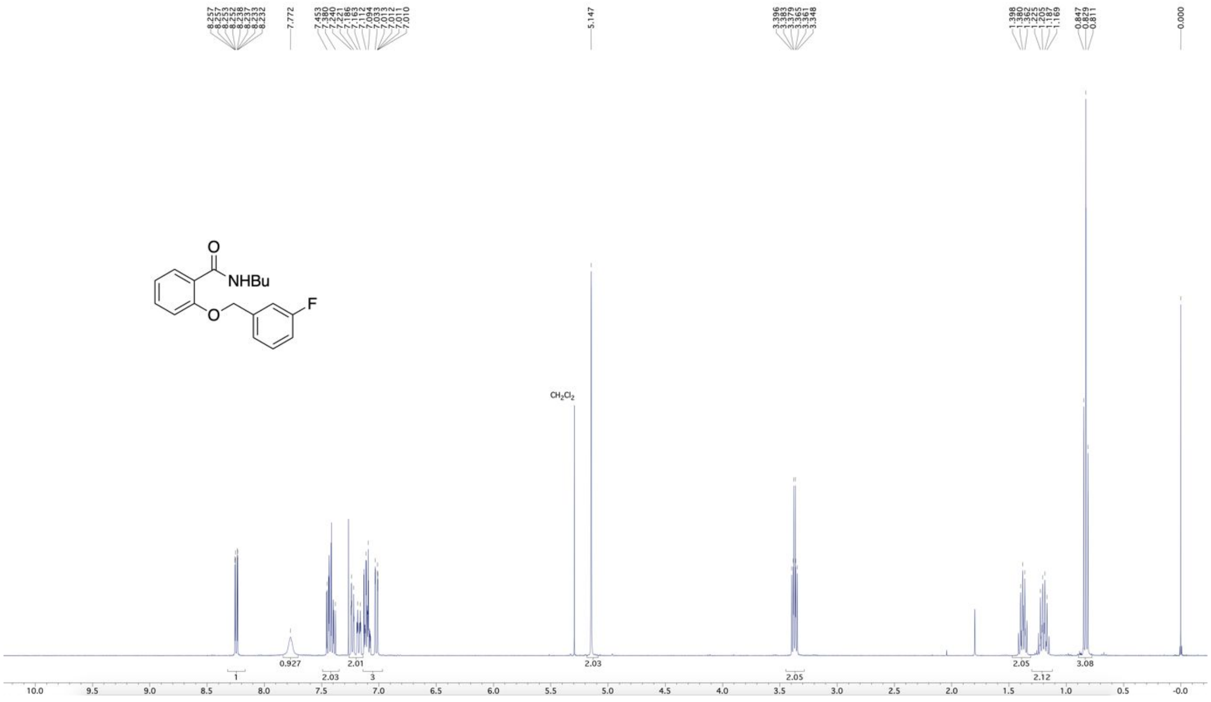

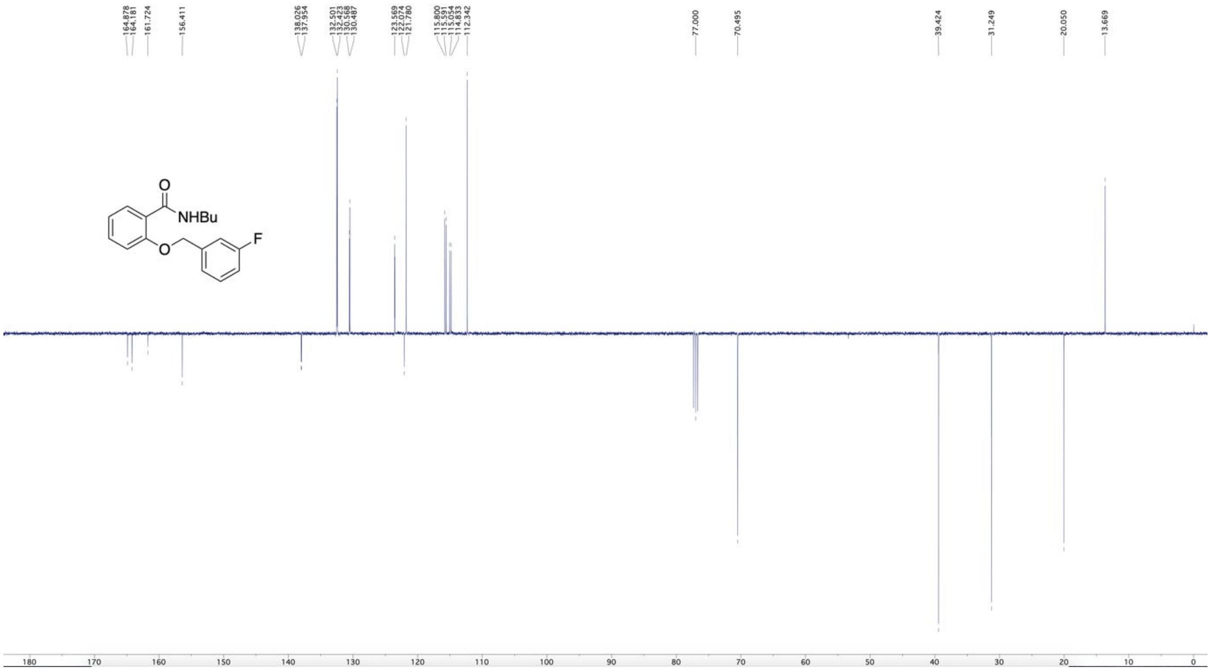

164.878
164.181
161.724
156.411
138.026
137.954
132.501
132.423
130.568
130.487
123.569
122.074
121.780
115.800
115.591
115.054
114.833
112.342
77.000
70.495
39.424
31.249
20.050
13.669
O
NHBu
O
F
180
170
160
150
140
130
120
110
100
90
80
70
60
50
40
30
20
10
0

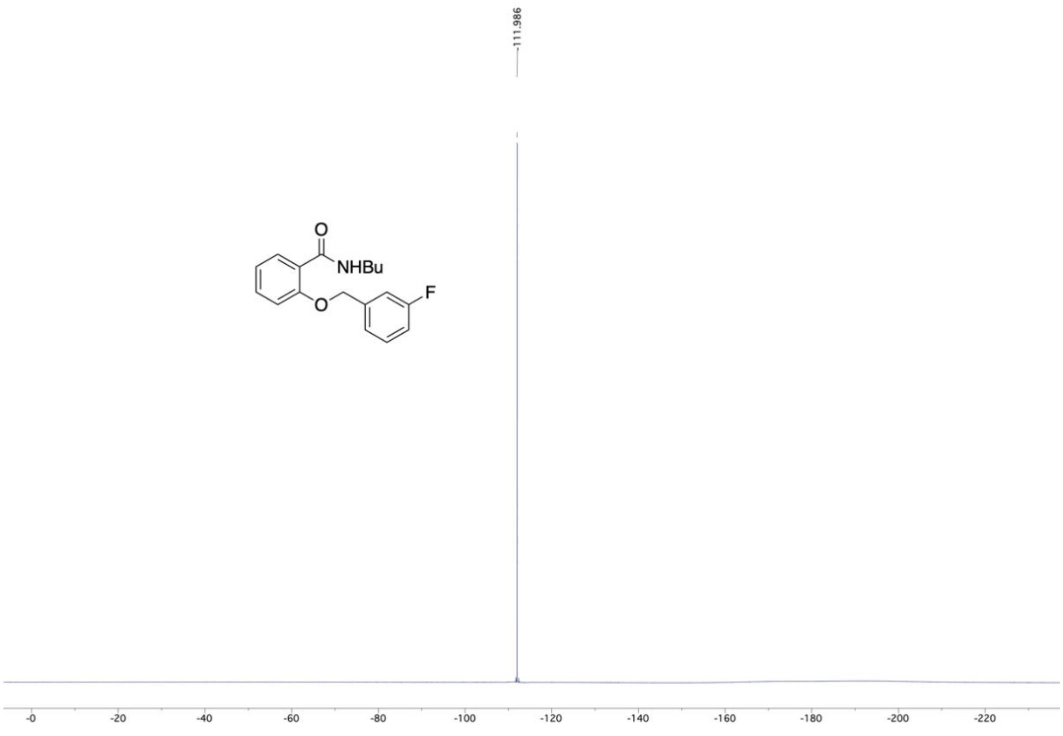

-111.986
O
NHBu
O
F
-0
-20
-40
-60
-80
-100
-120
-140
-160
-180
-200
-220

N-Butyl-2-((2-fluorobenzyl)oxy)benzamide **21j**

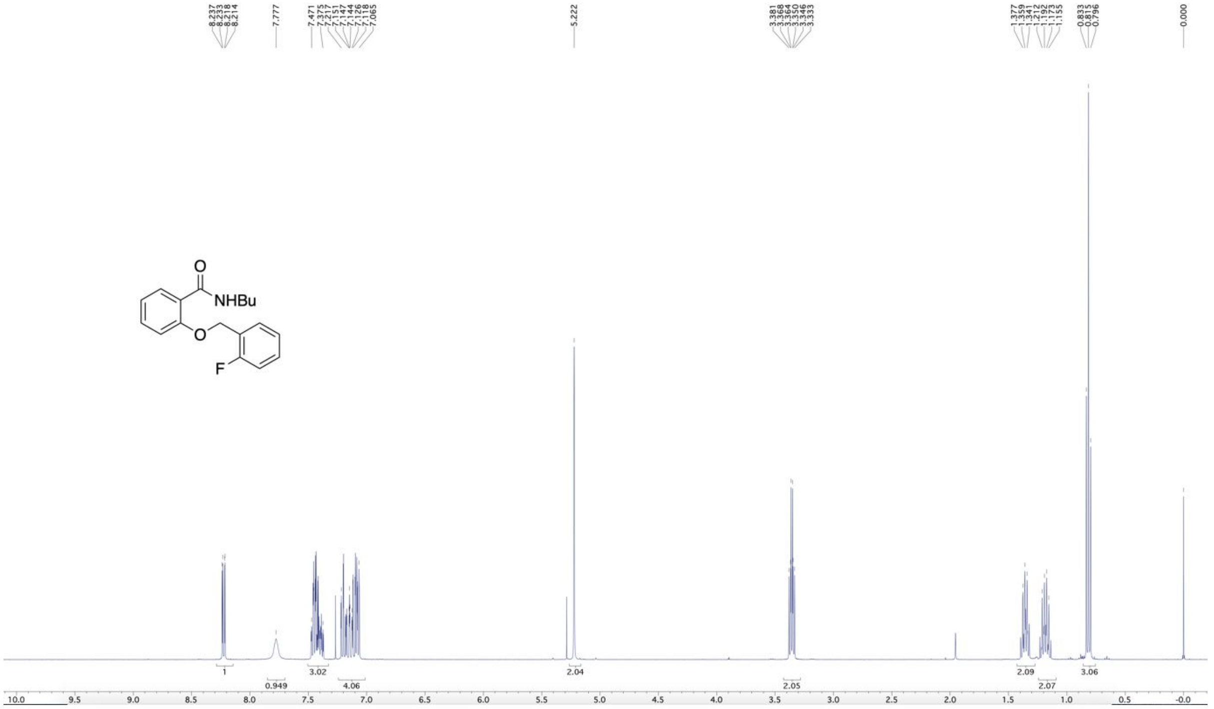

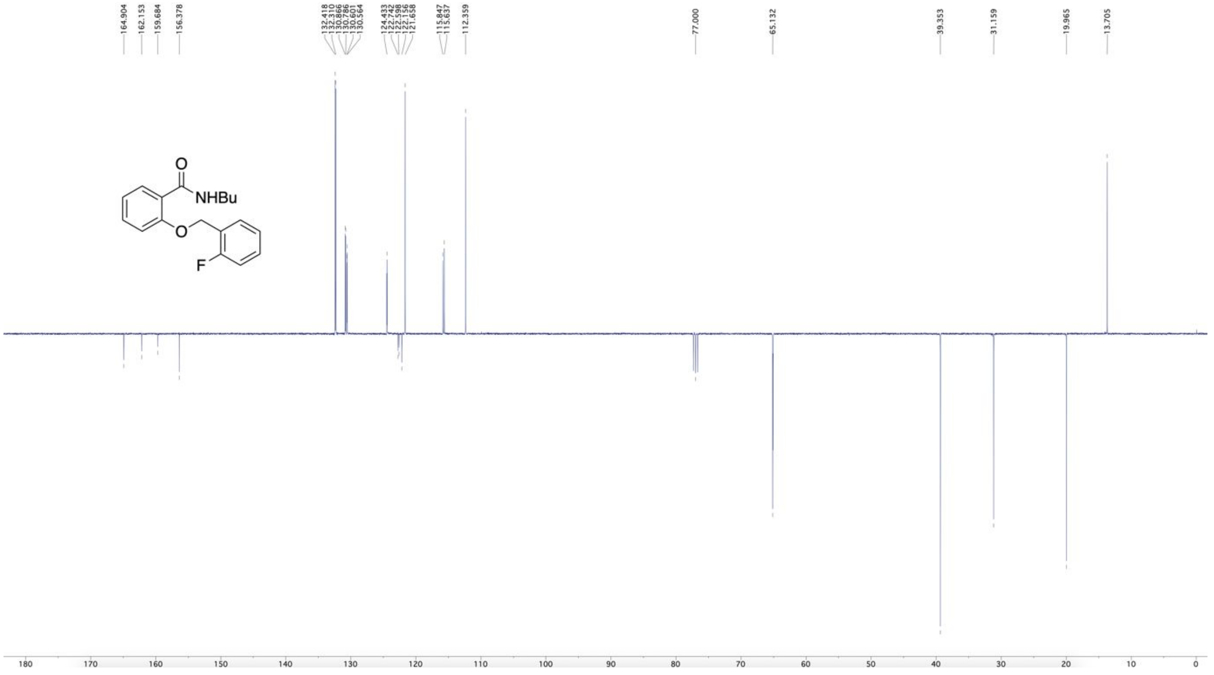

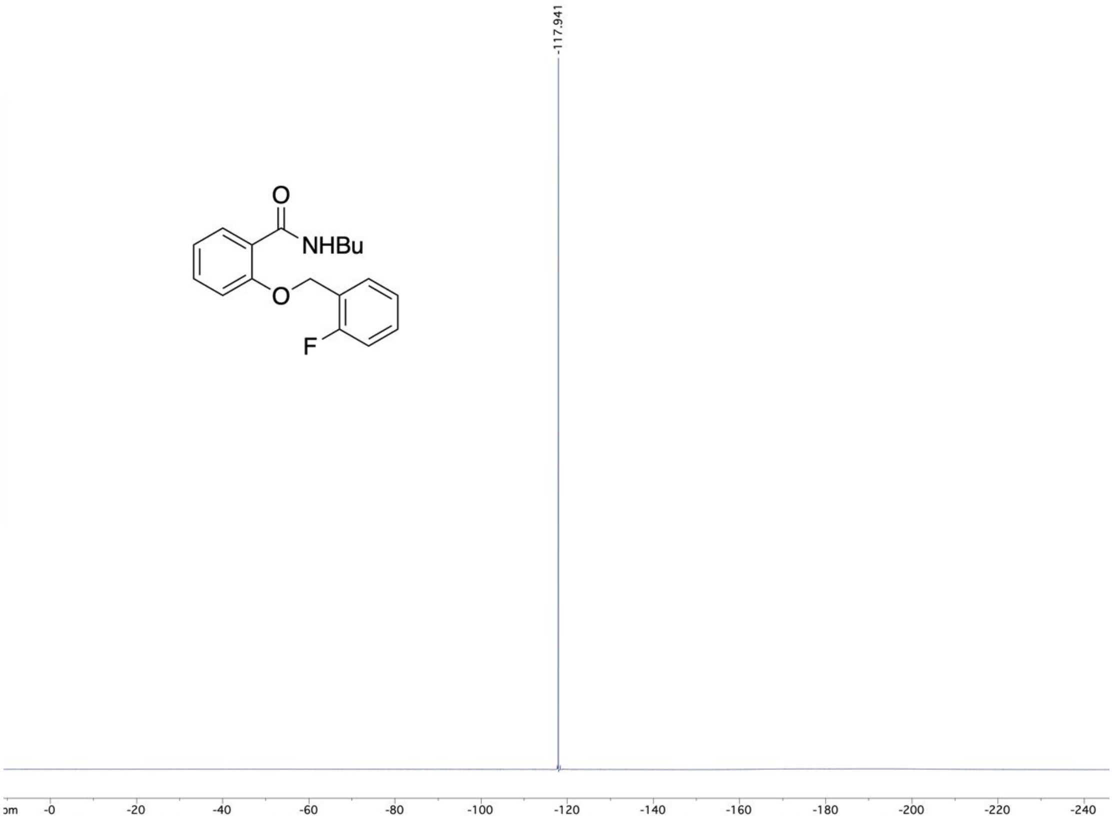

N-Butyl-2-((2-(trifluoromethyl)benzyl)oxy)benzamide **21k**

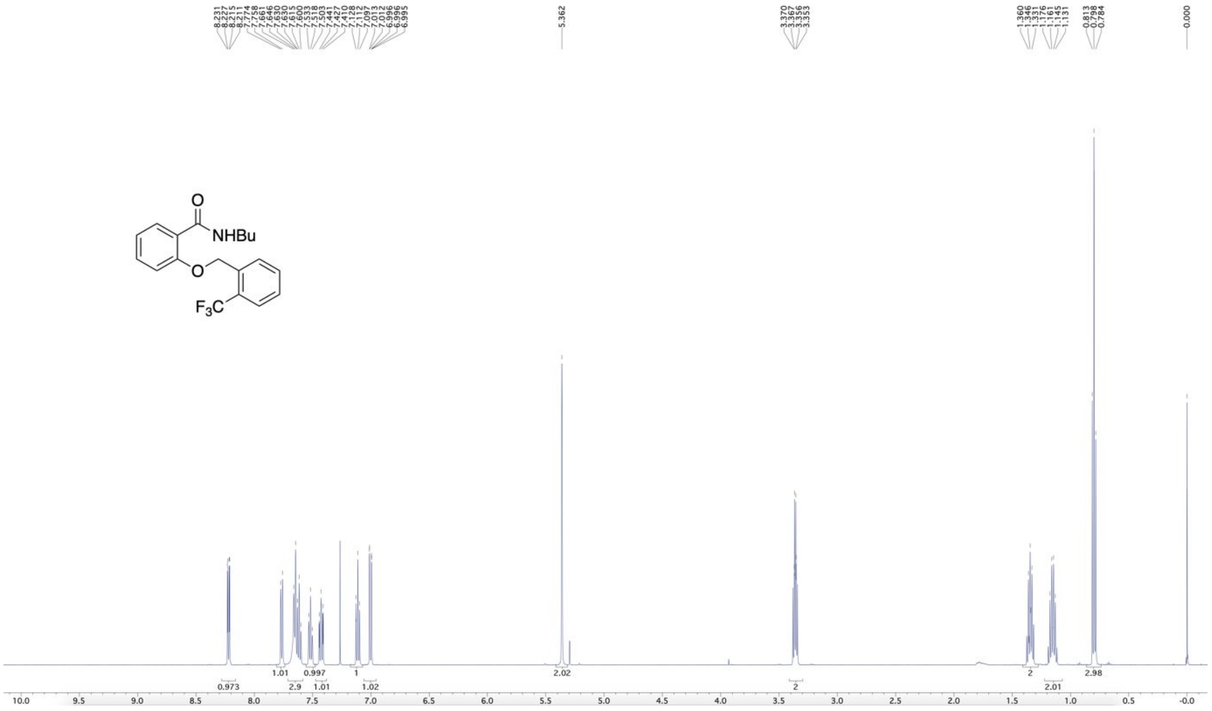

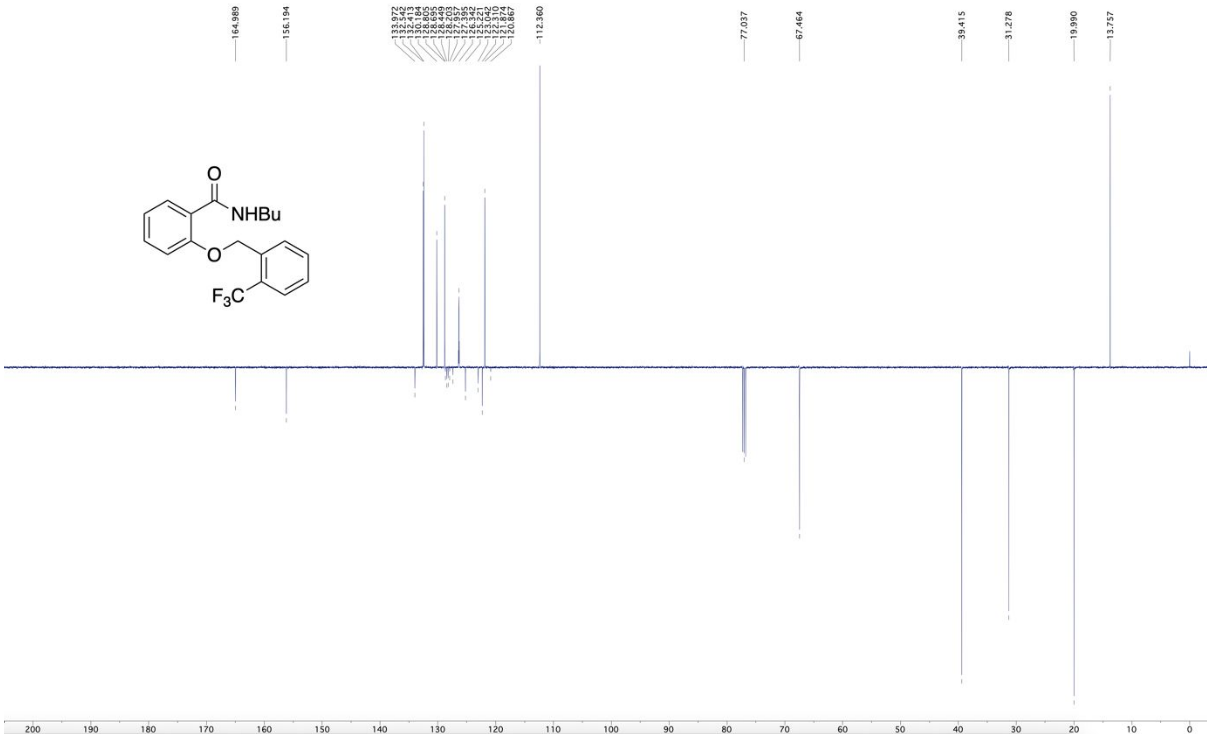

NHBu
O
F3C
164.989
156.194
112.360
77.037
67.464
39.415
31.278
19.990
13.757

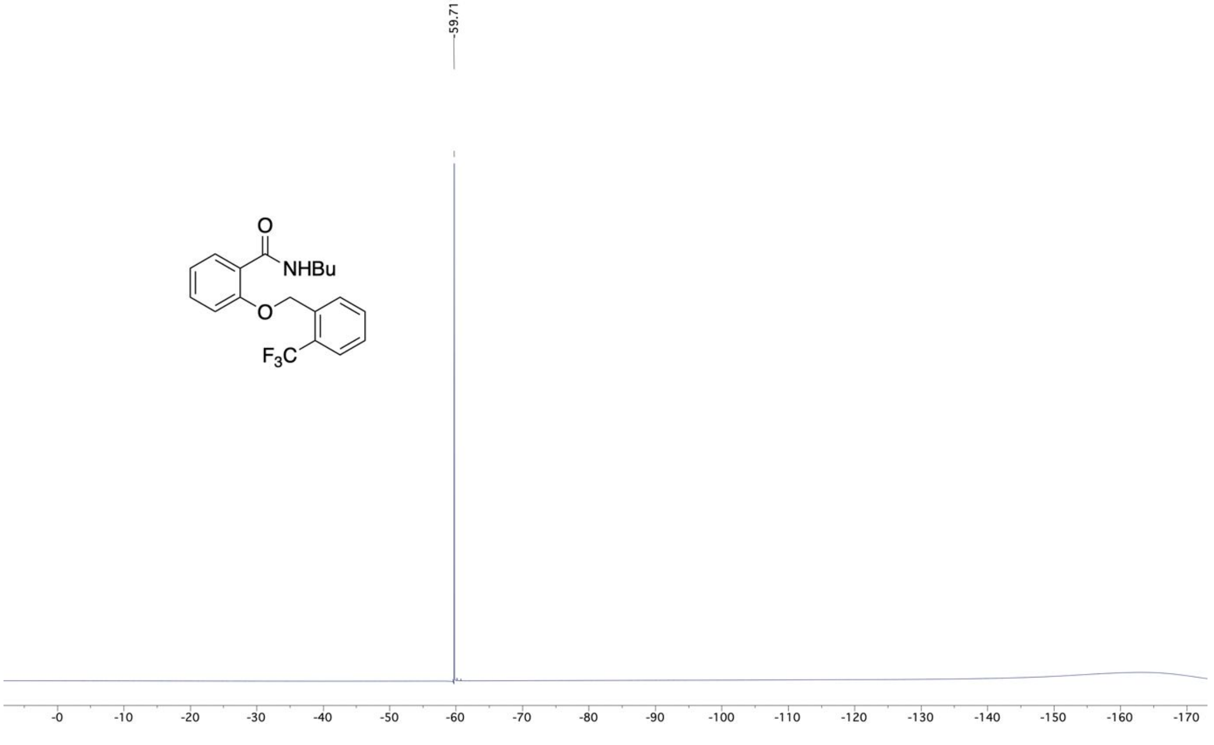

NHBu
O
F3C
-59.711

N-Butyl-2-((perfluorophenyl)methoxy)benzamide **21l**

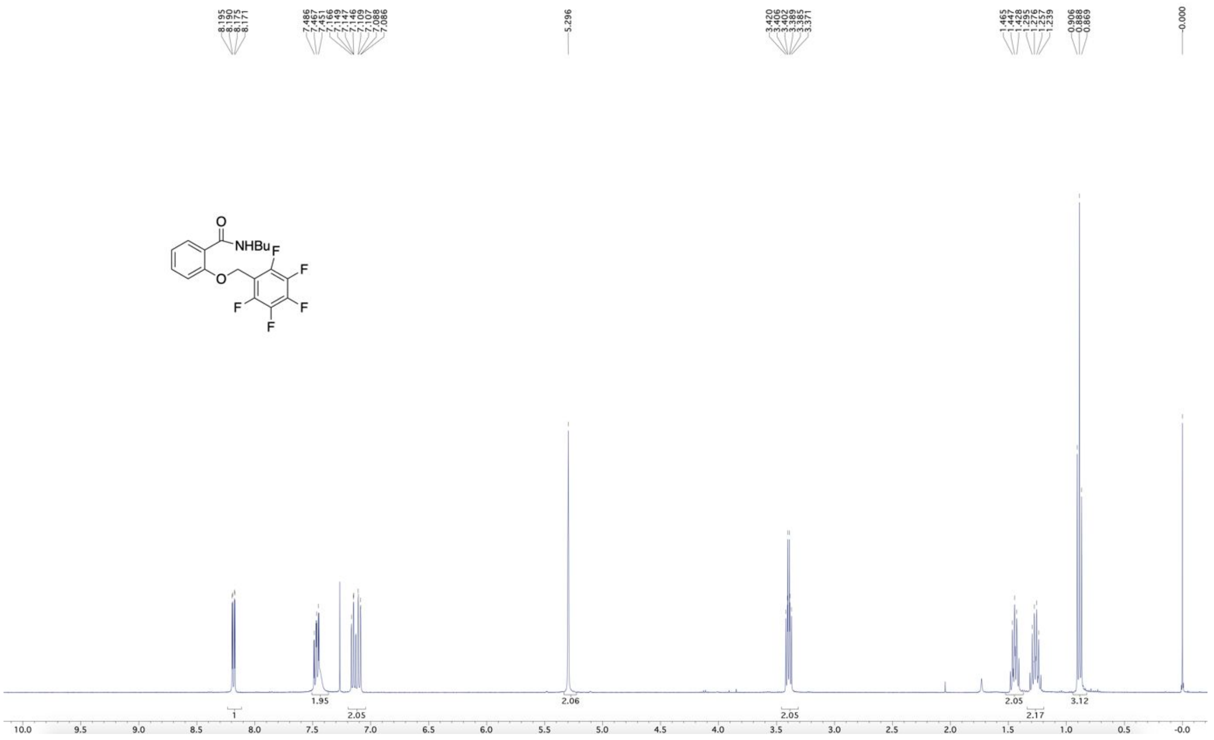

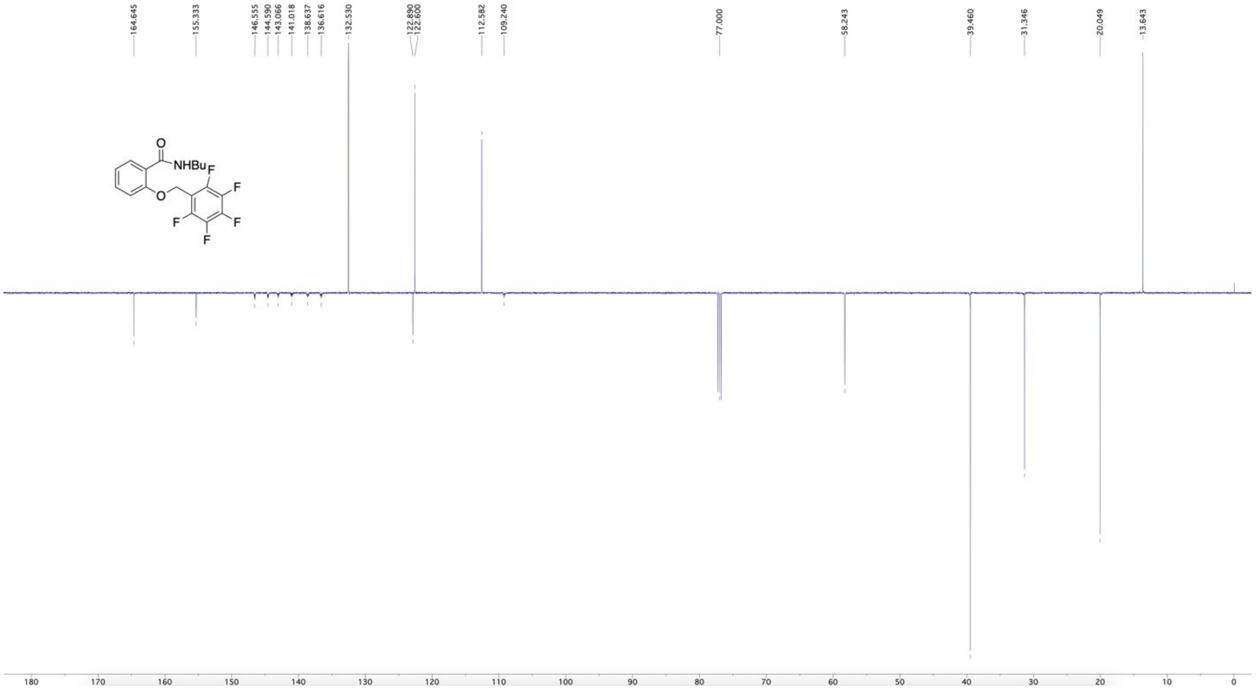

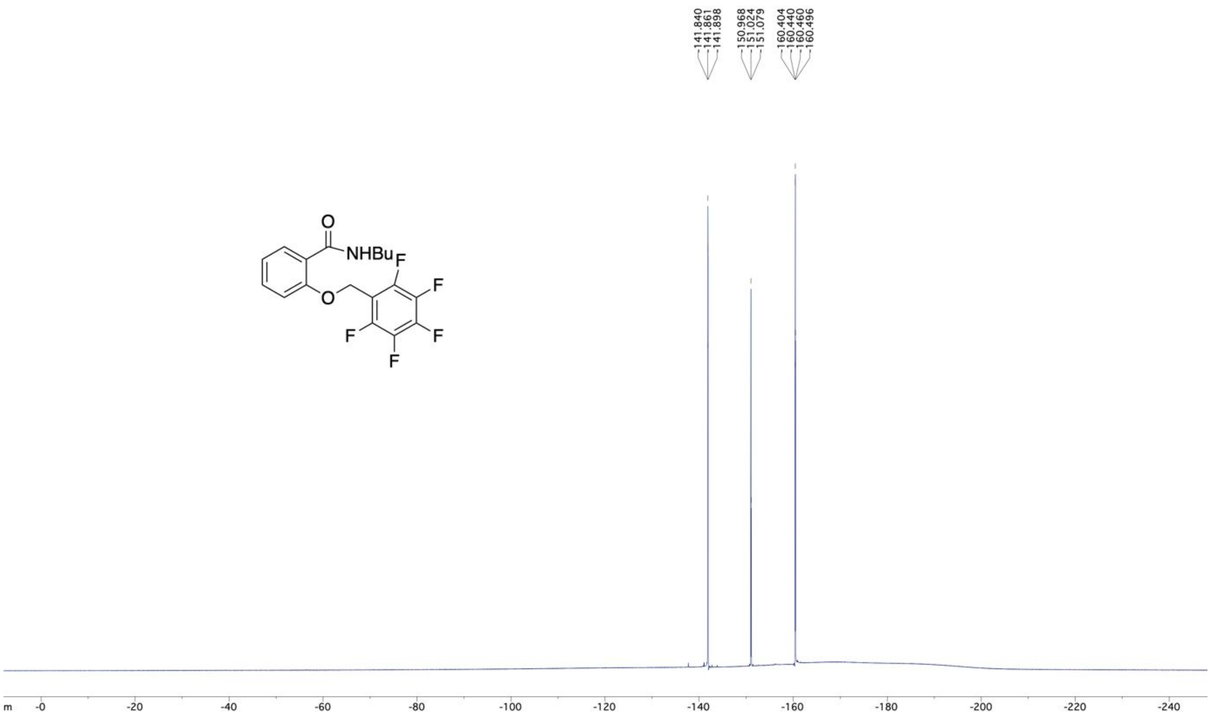

N-Butyl-2-((4-nitrobenzyl)oxy)benzamide **21m**

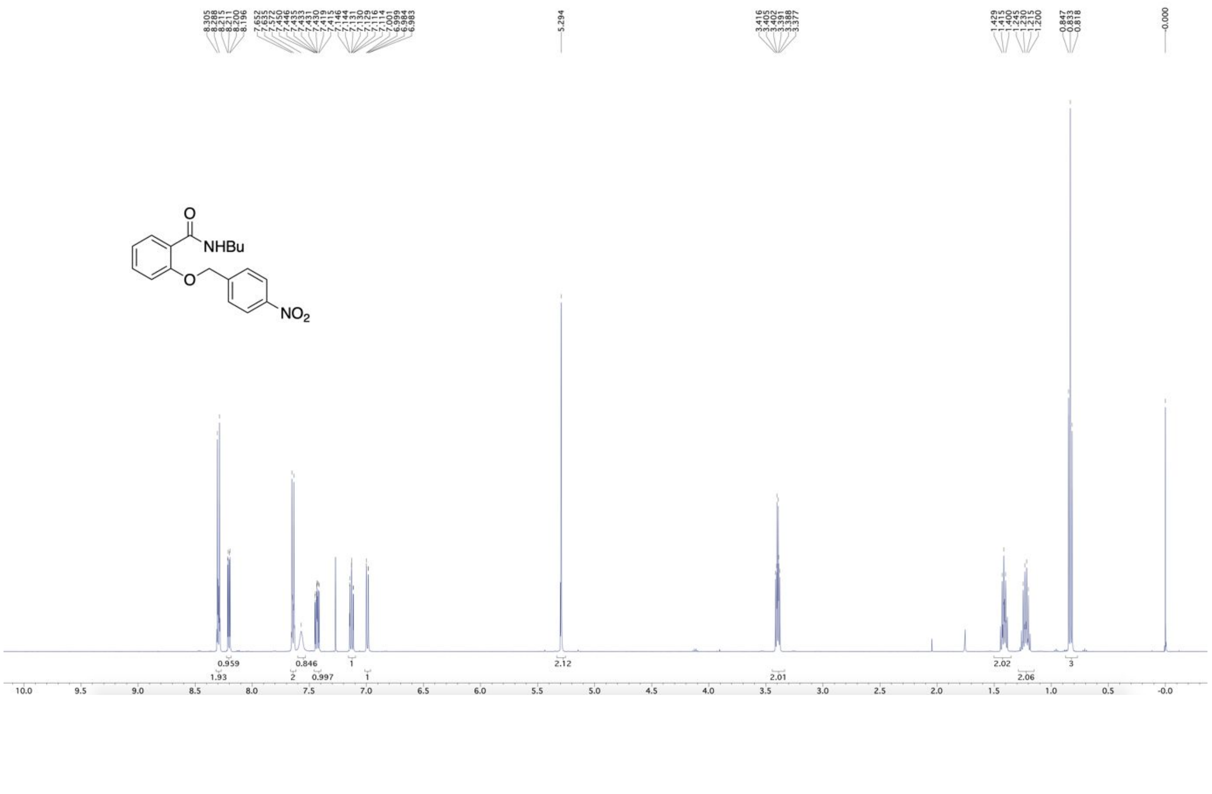

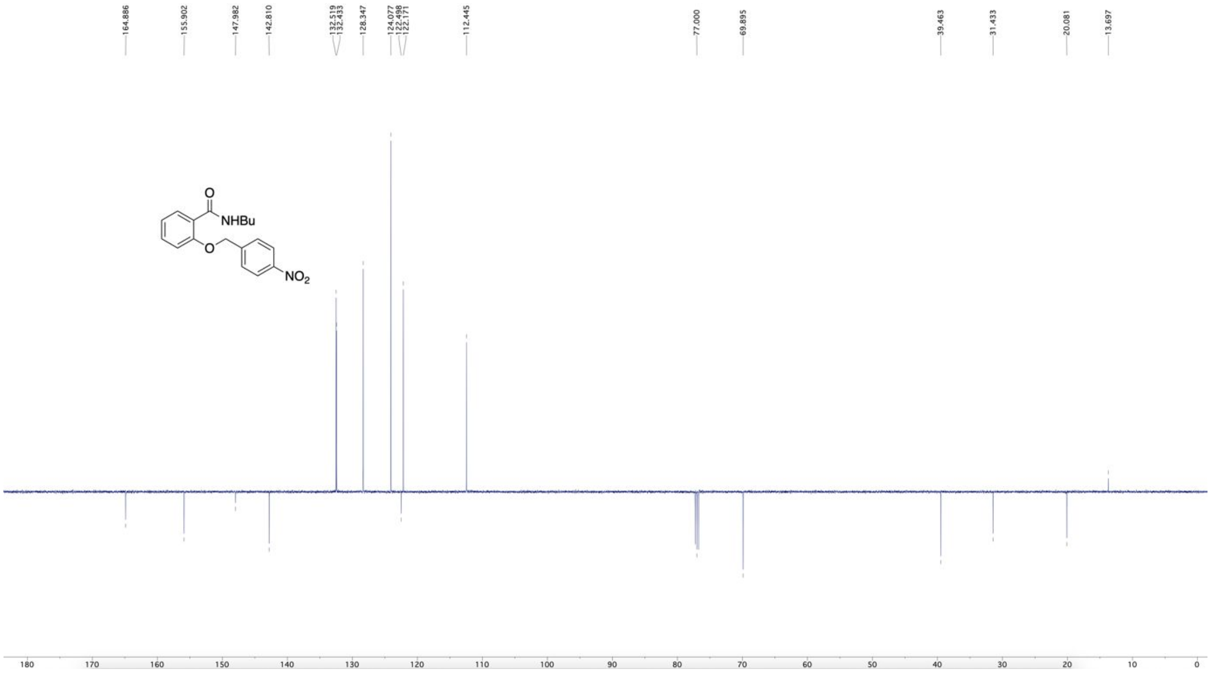

N-Butyl-2-((3-nitrobenzyl)oxy)benzamide **21n**

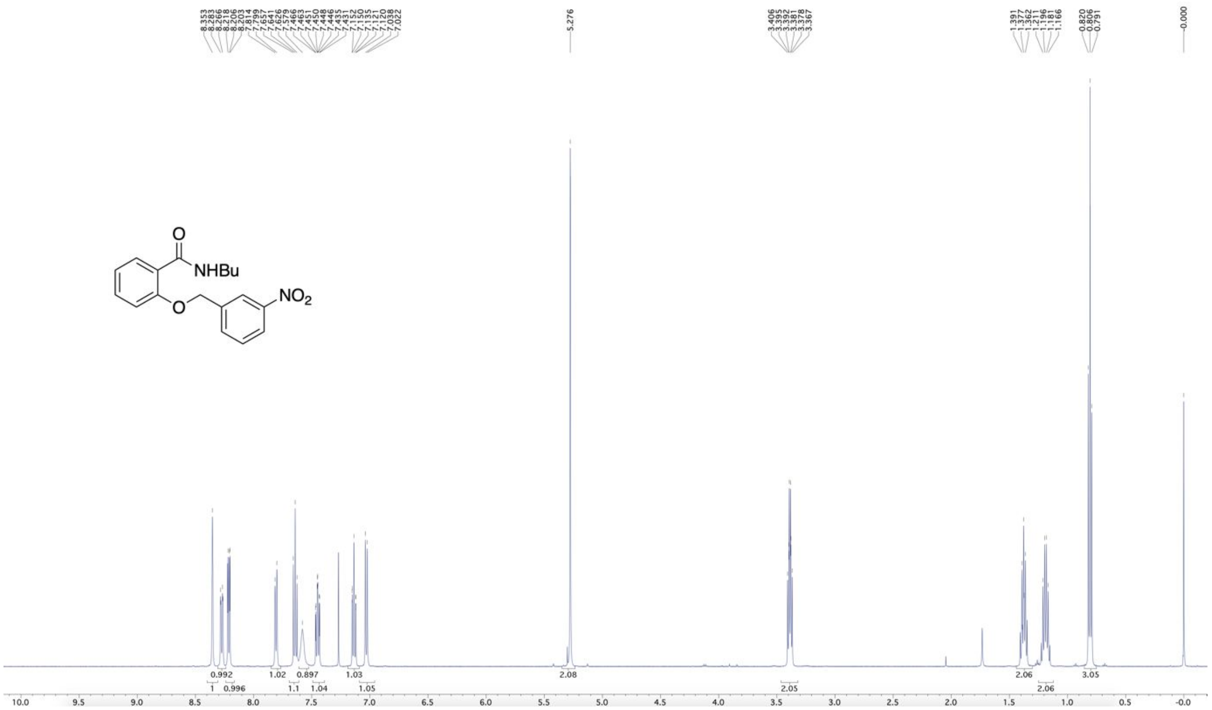

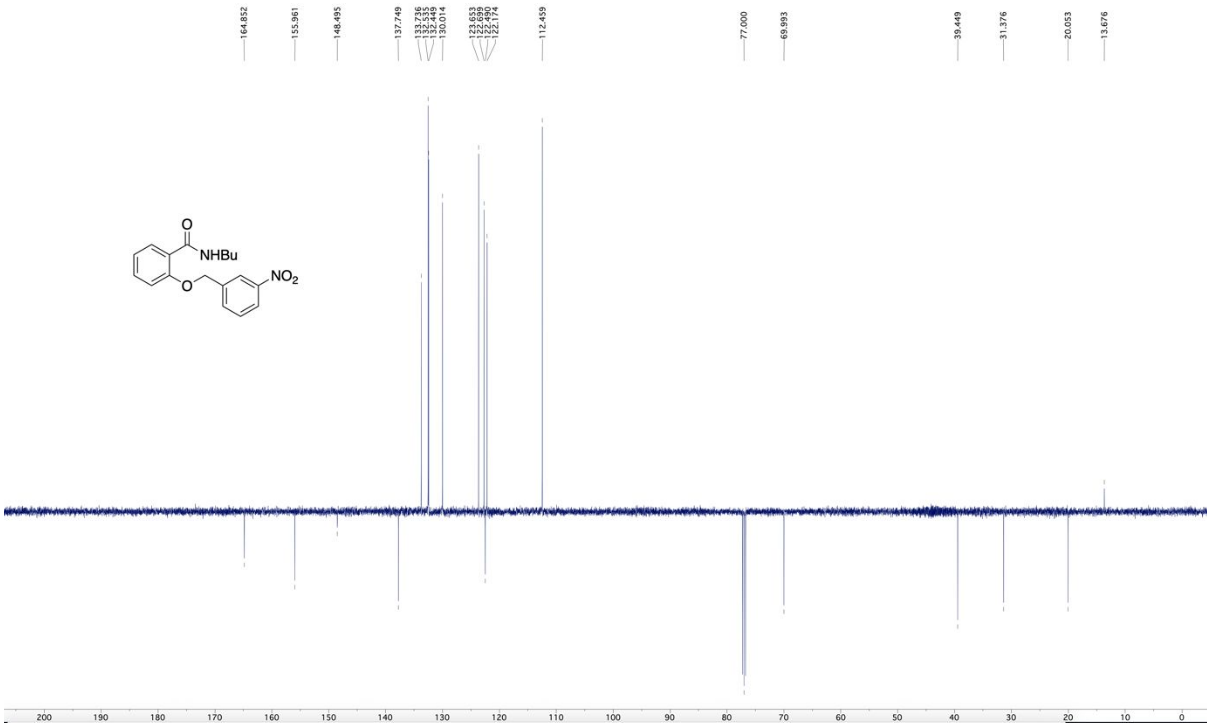

N-Butyl-2-((2-nitrobenzyl)oxy)benzamide **21o**

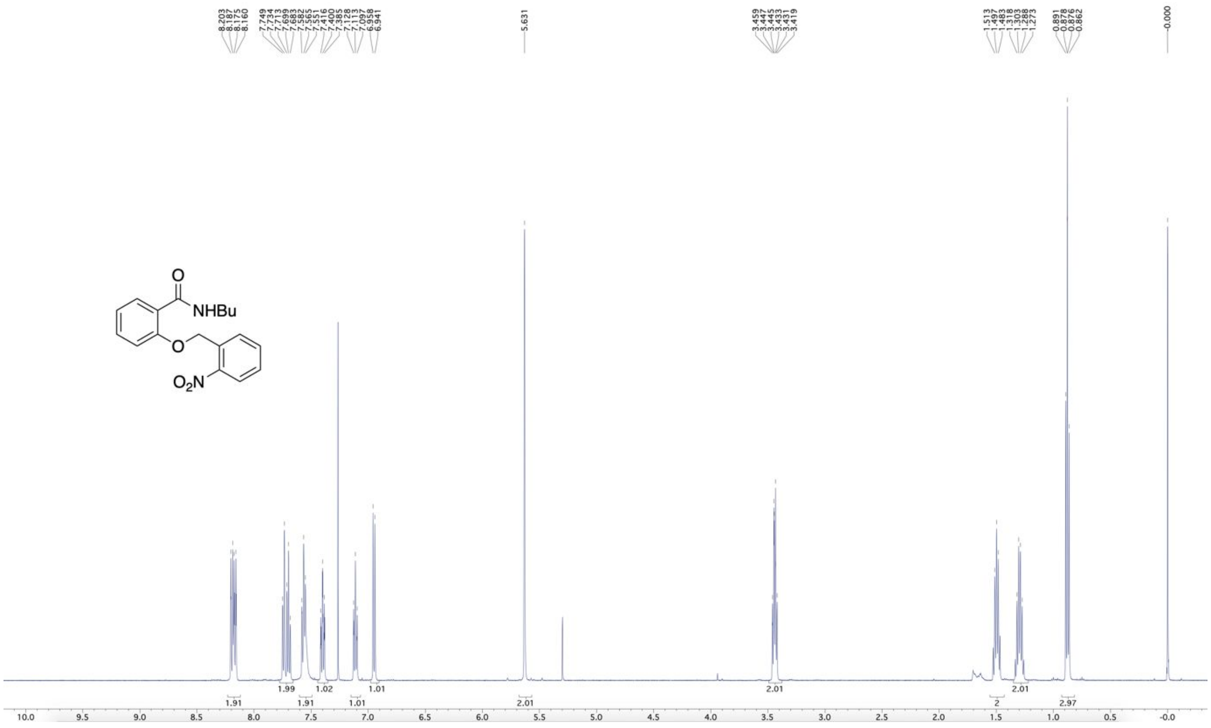

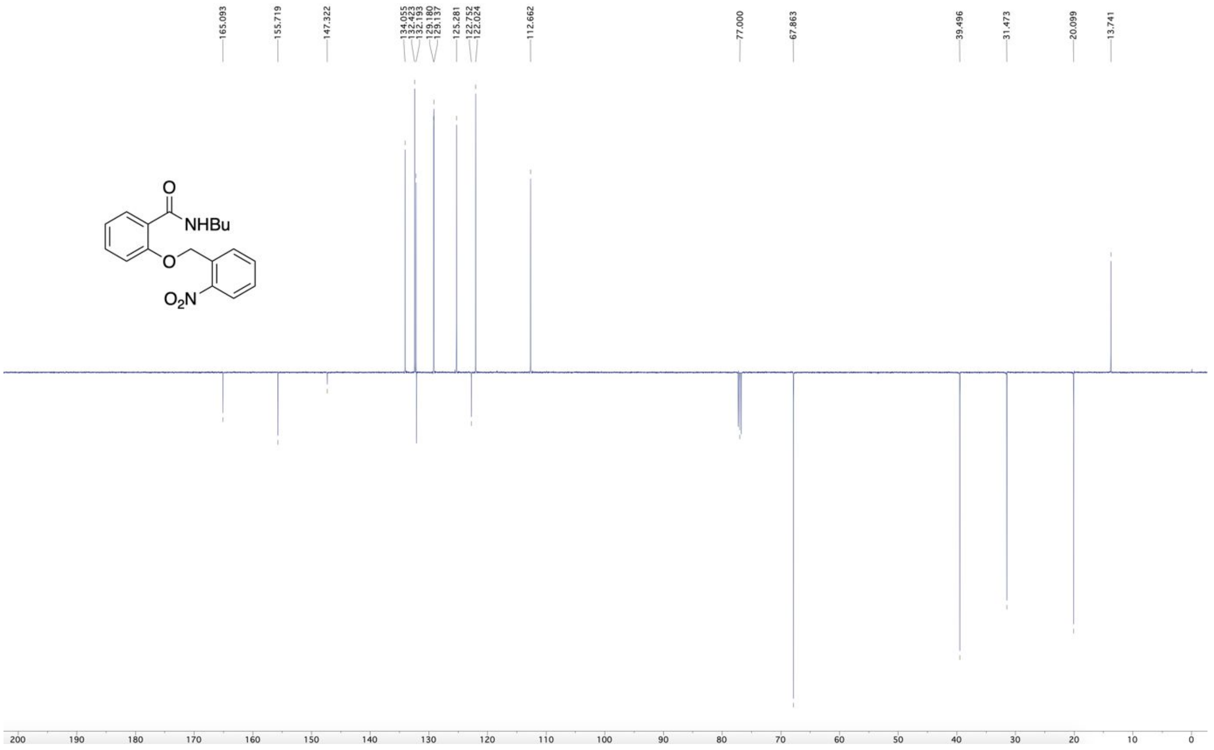

N-Butyl-2-(pyridin-4-ylmethoxy)benzamide **21p**

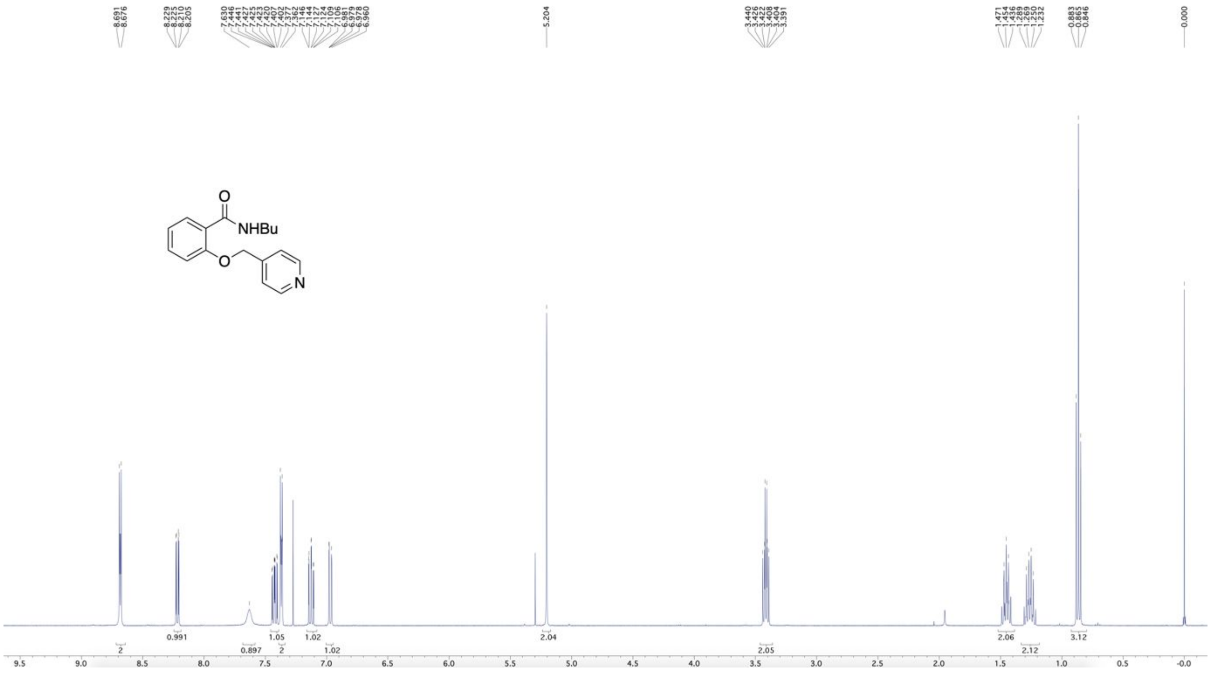

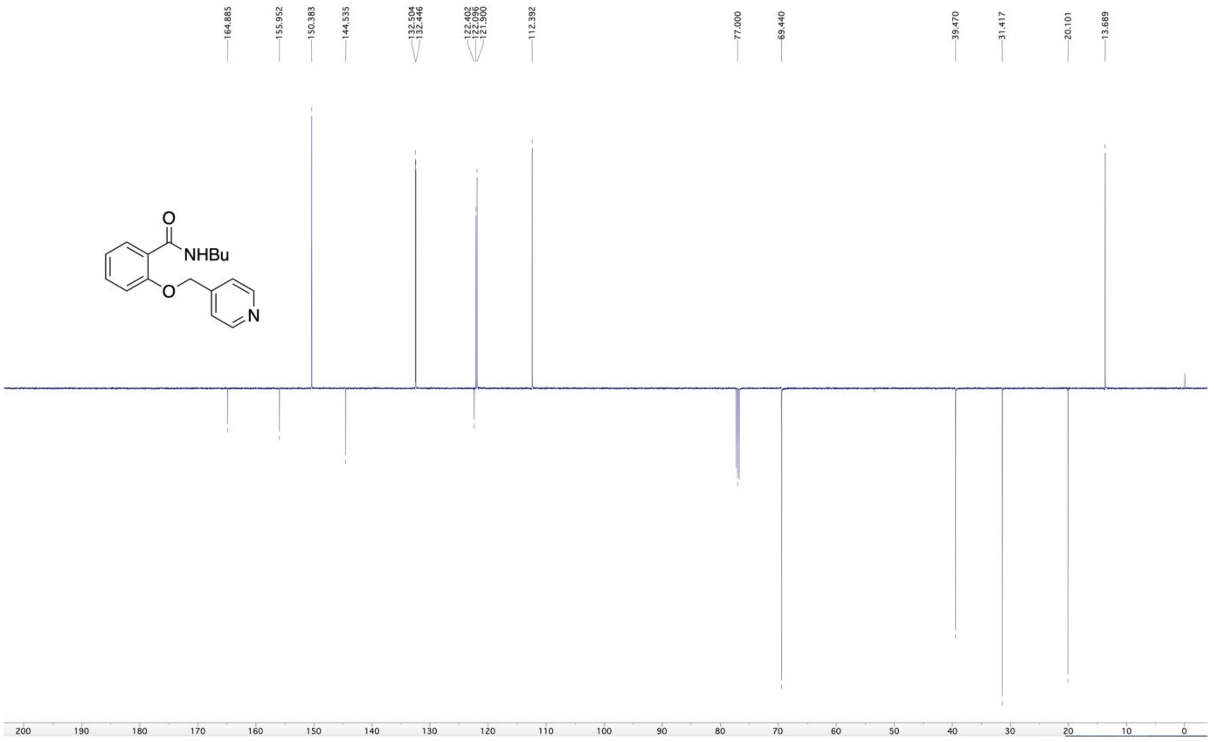

N-Butyl-2-(thiophen-2-ylmethoxy)benzamide **21q**

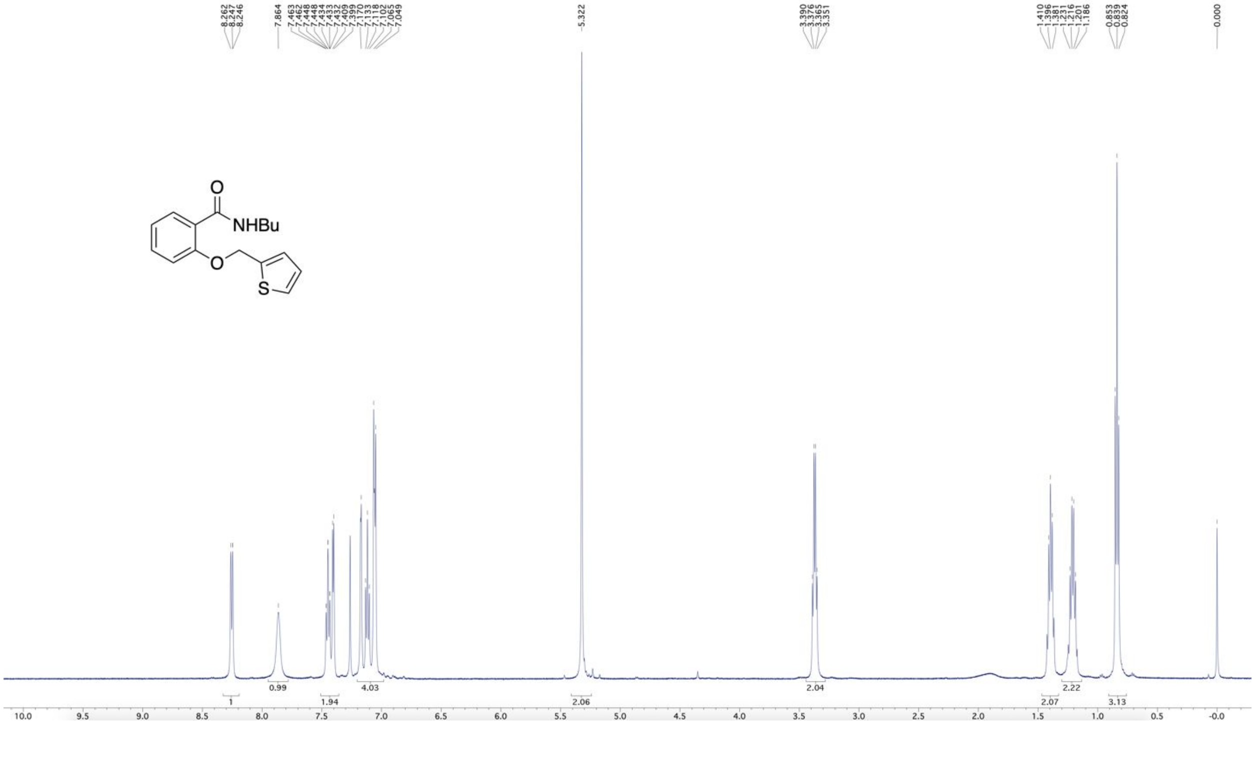

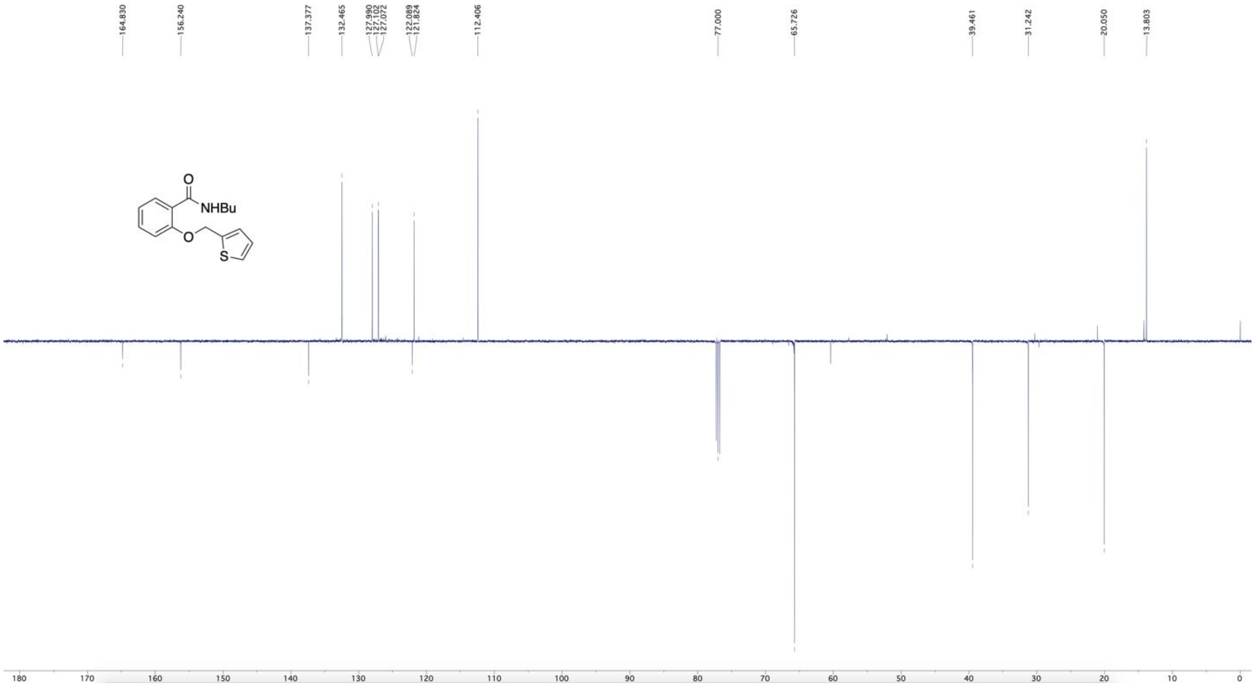

2-(Diphenylmethoxy)-*N*-butylbenzamide **21r**

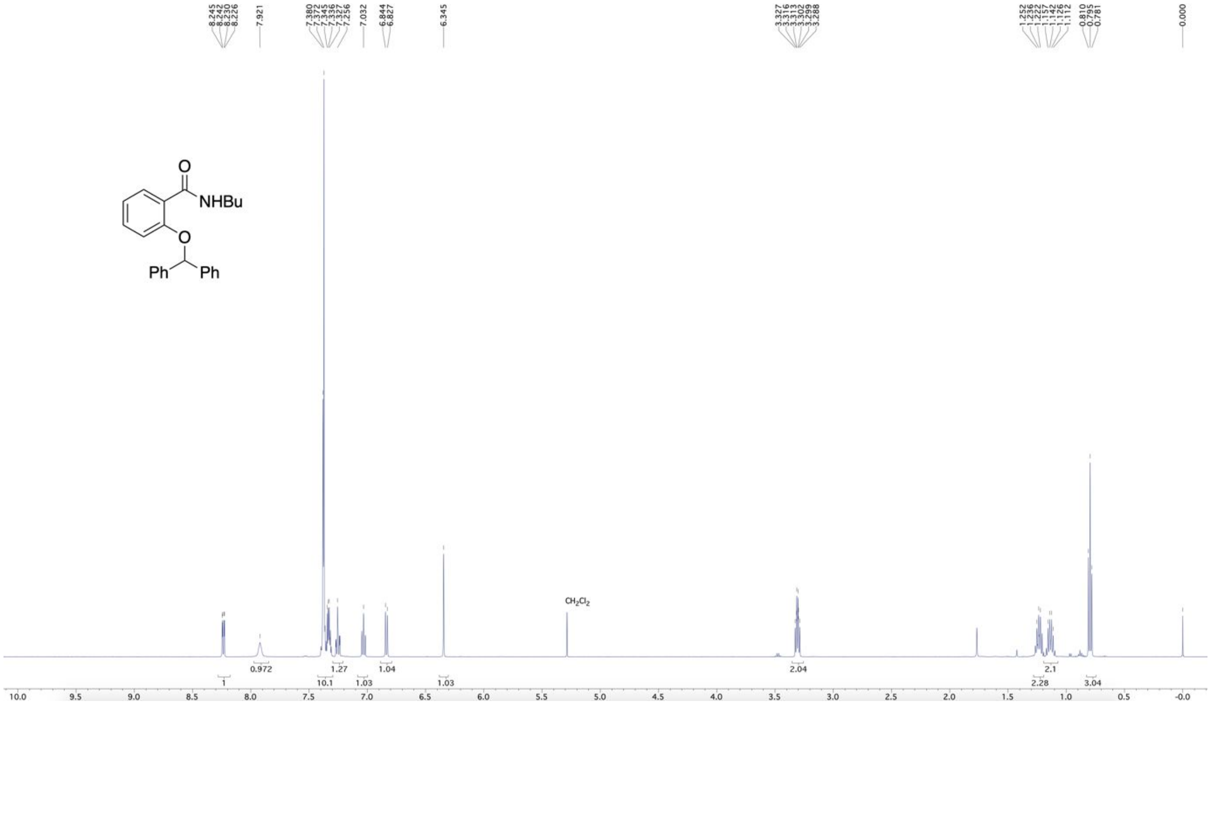

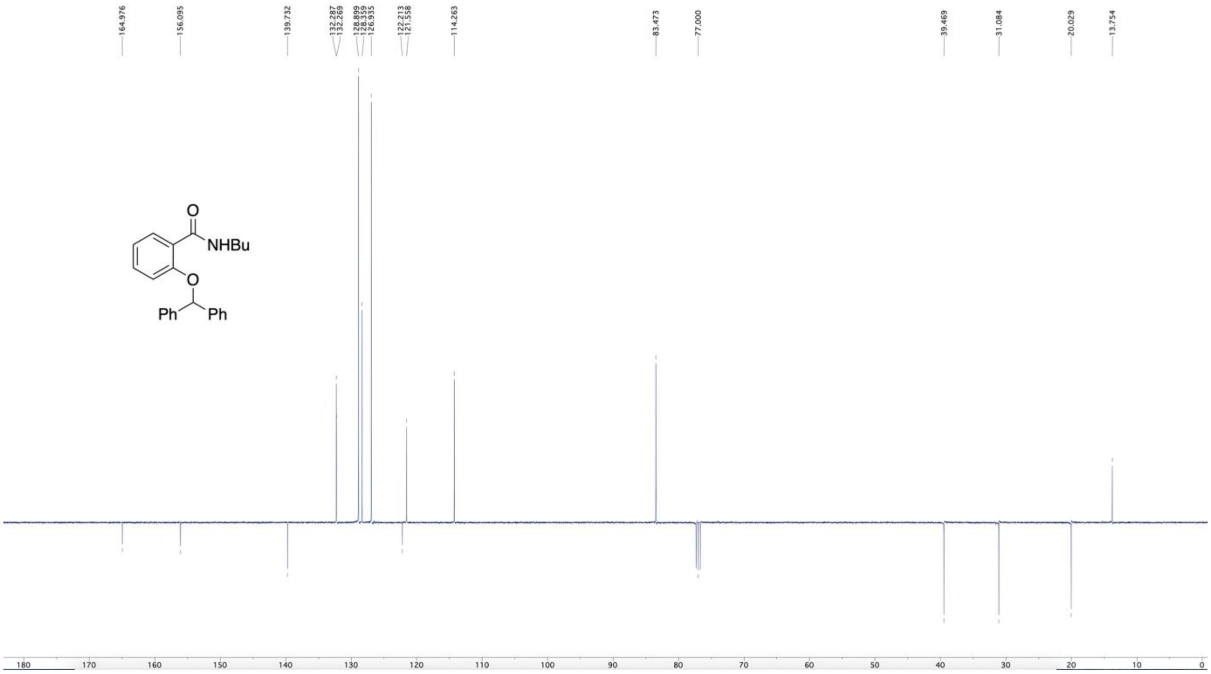

N-Butyl-2-(1-phenylethoxy)benzamide **21s**

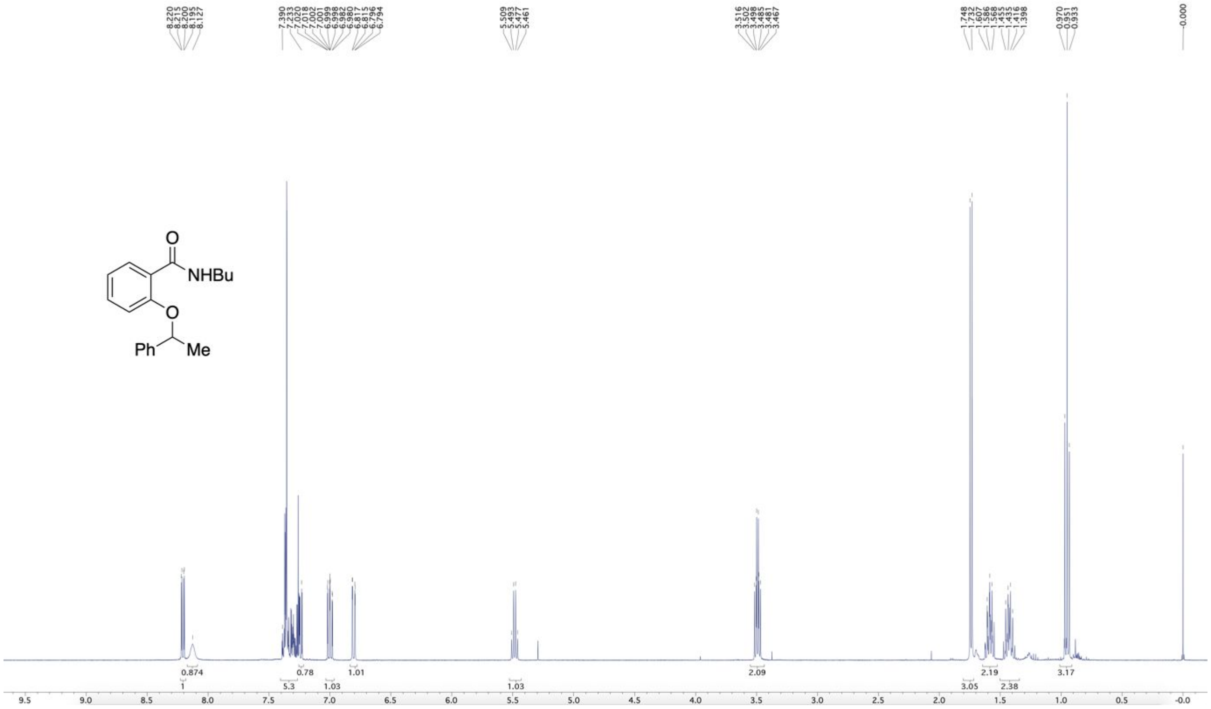

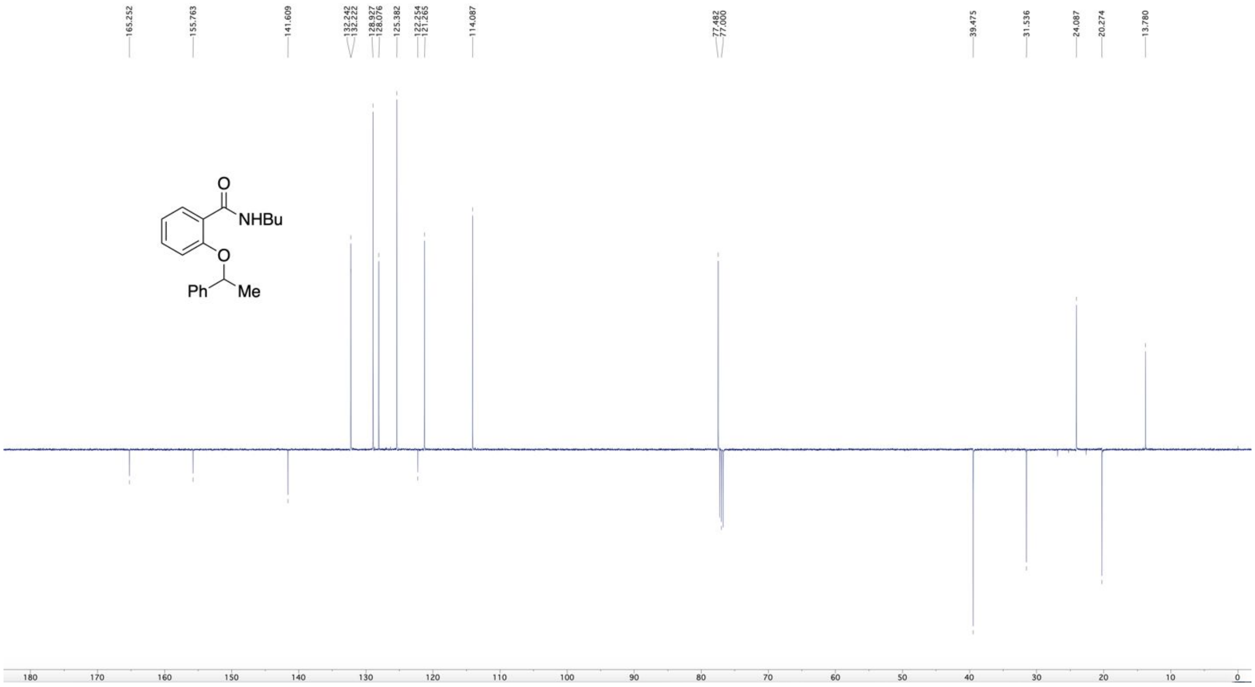

2-(Allyloxy)-*N*-butylbenzamide **21t**

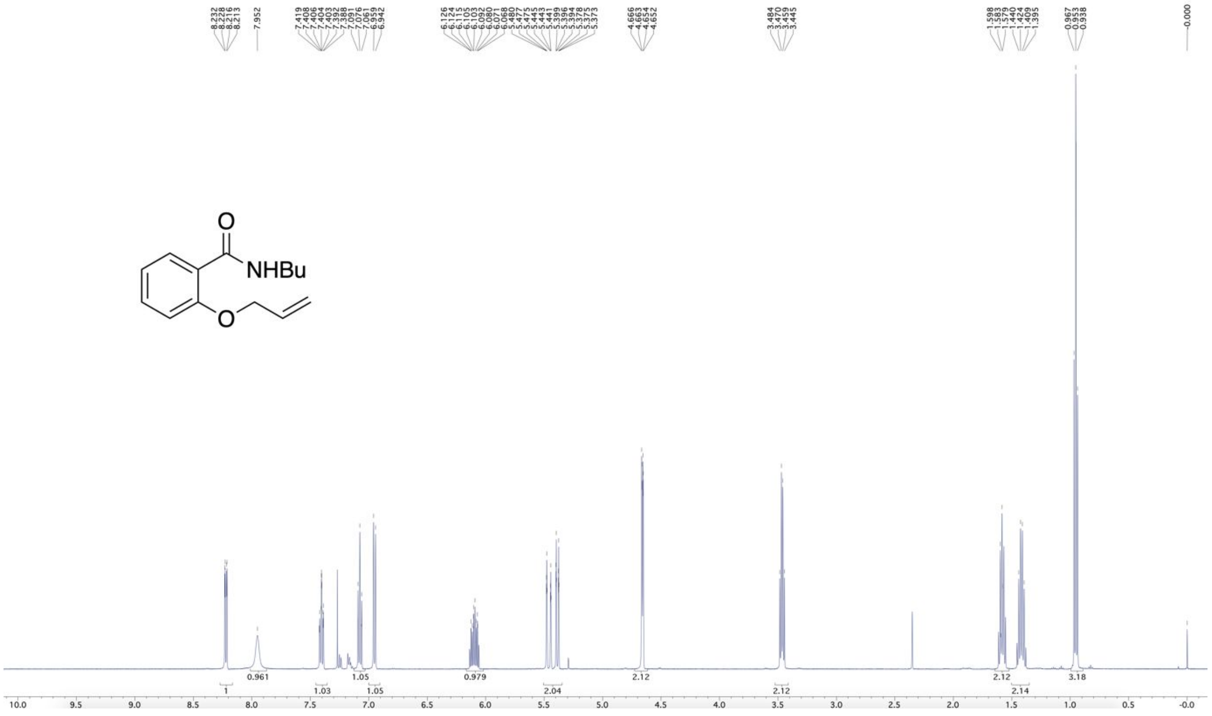

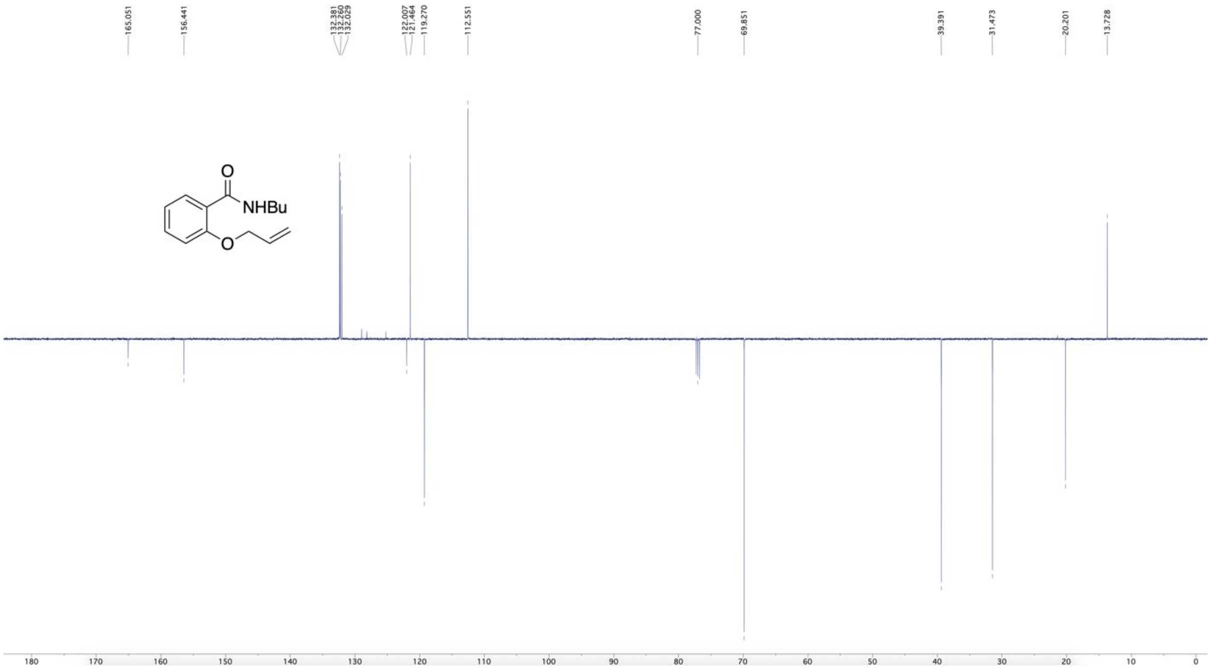

N-Butyl-2-((3-methylbut-2-en-1-yl)oxy)benzamide **21u**

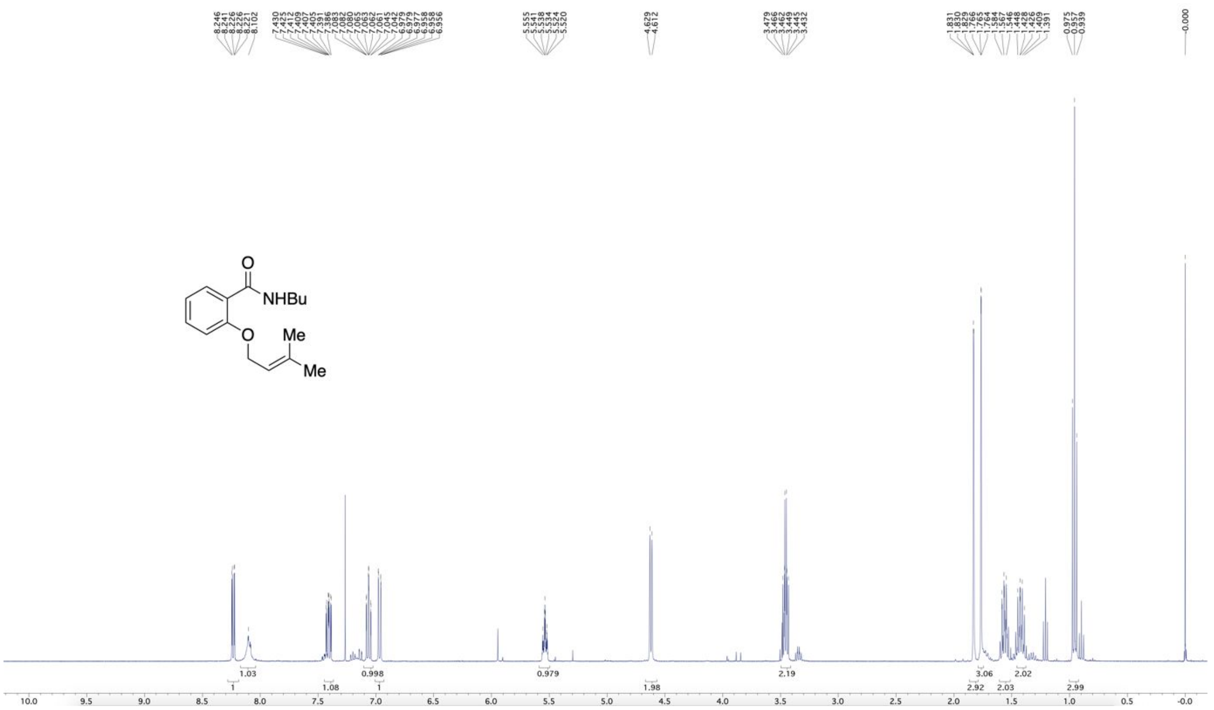

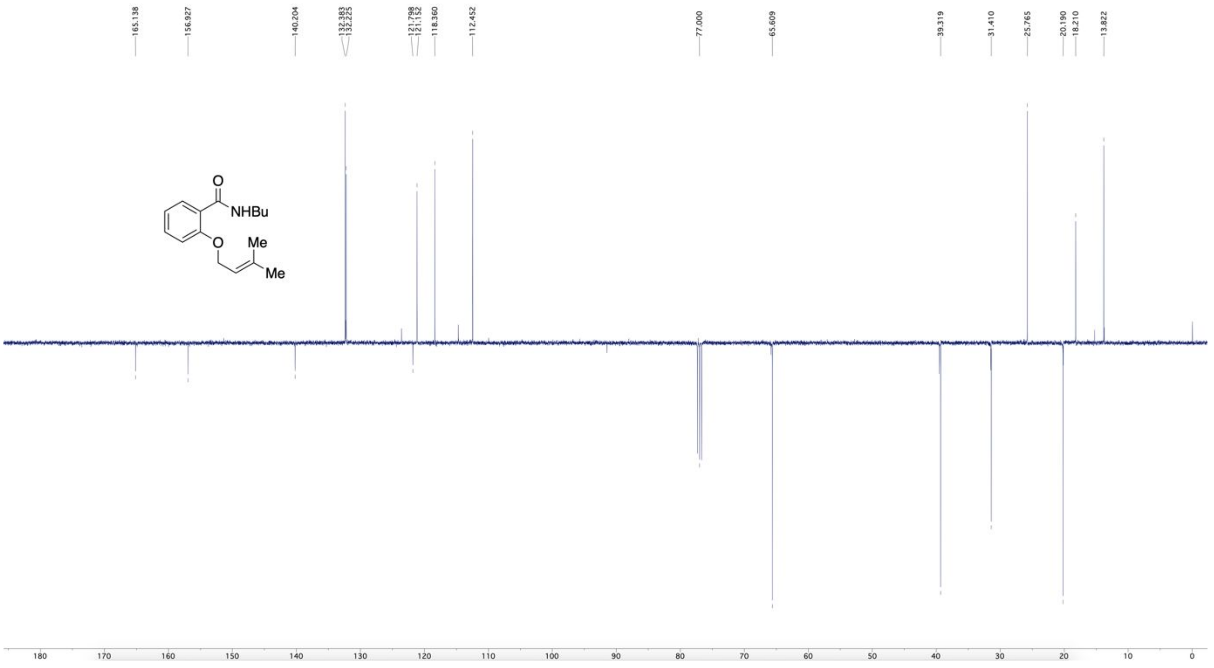

N-Butyl-2-(2,2,2-trifluoroethoxy)benzamide **21v**

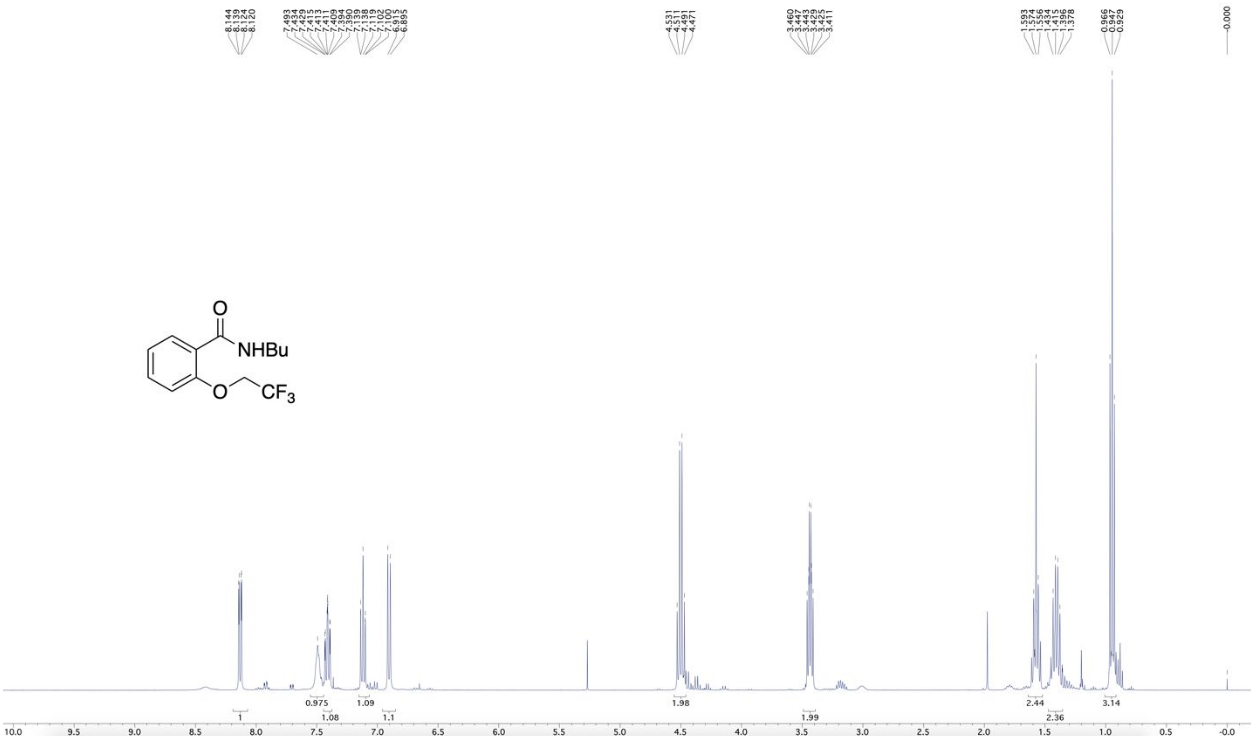

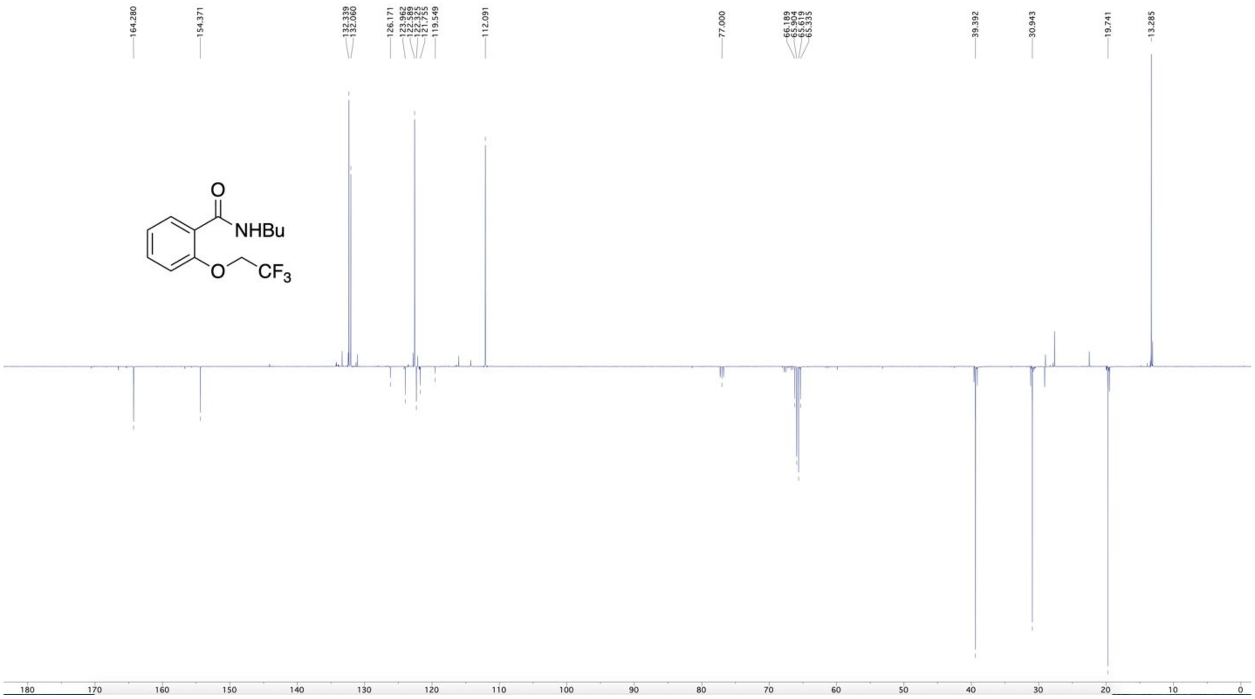
164.280
154.371
132.339
132.060
126.171
123.962
122.589
122.325
121.755
119.549
112.091
77.000
66.189
65.904
65.619
65.335
39.392
30.943
19.741
13.285
O
NHBu
O
CF_3
180
170
160
150
140
130
120
110
100
90
80
70
60
50
40
30
20
10
0

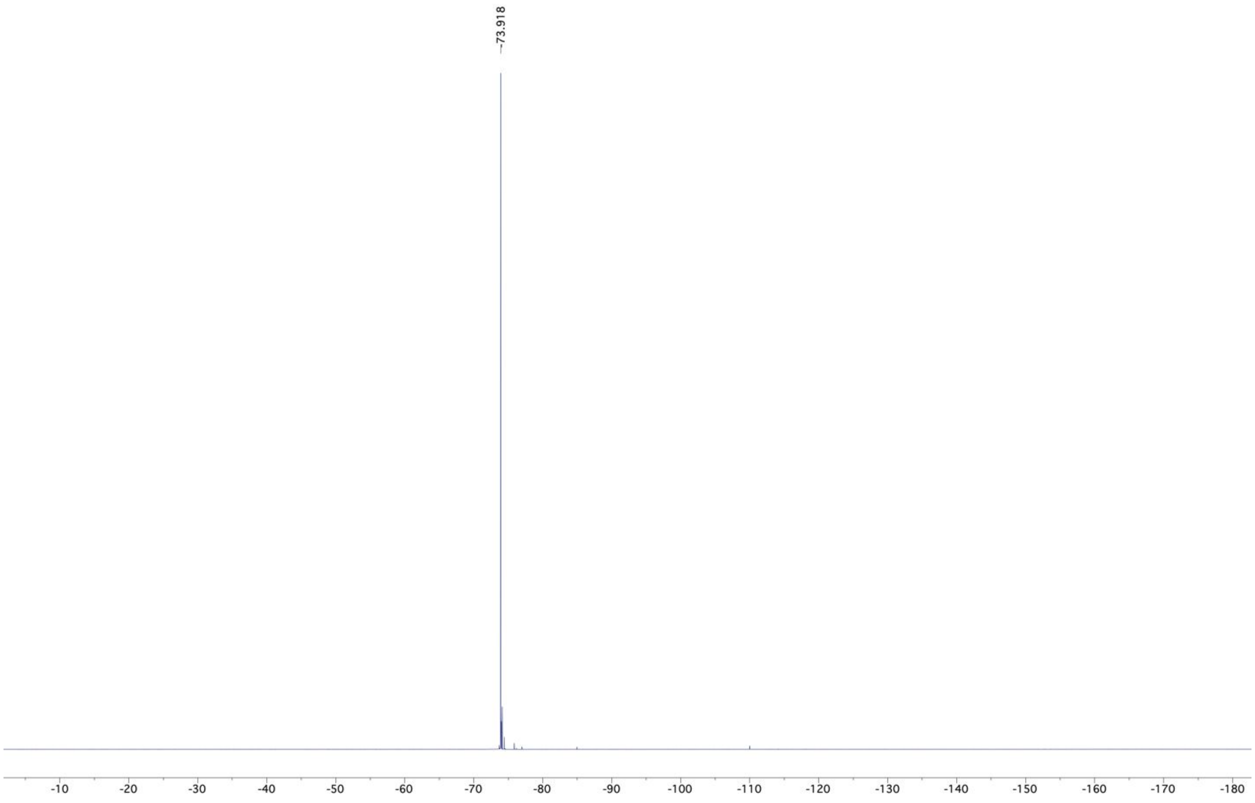
-73.918
-10
-20
-30
-40
-50
-60
-70
-80
-90
-100
-110
-120
-130
-140
-150
-160
-170
-180

N-Butyl-2-((3-phenylprop-2-yn-1-yl)oxy)benzamide **21w**

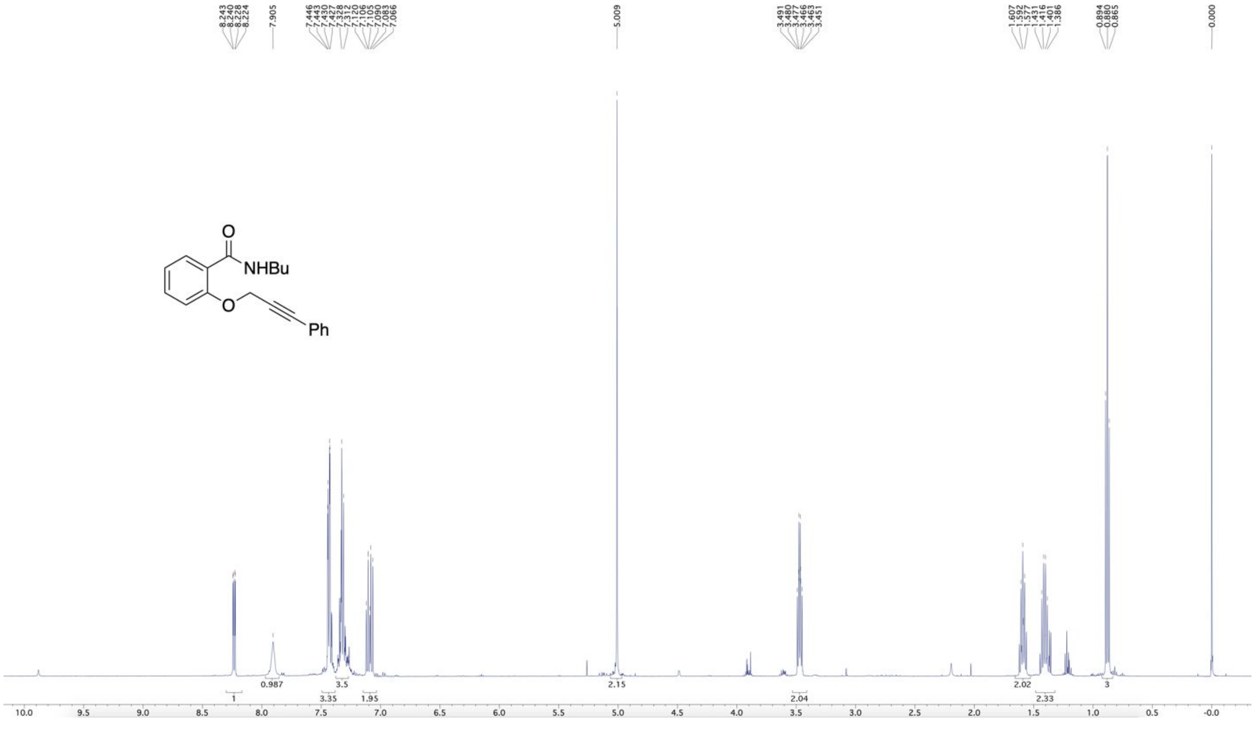

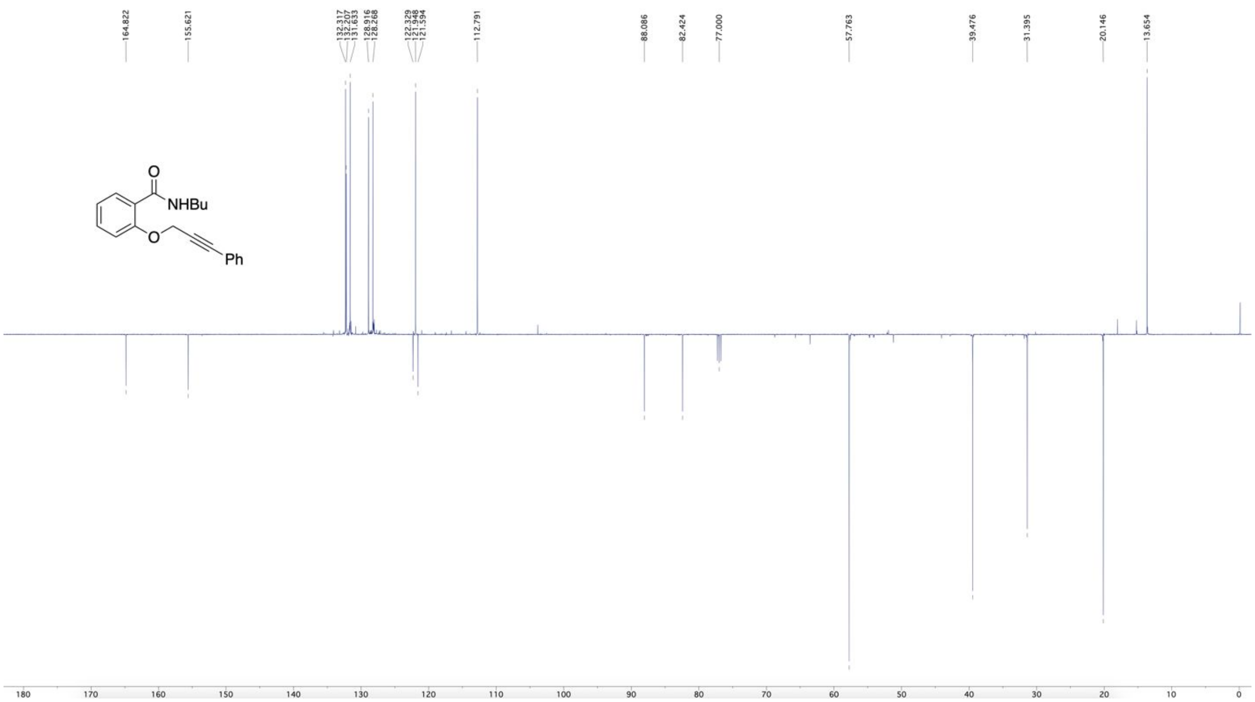

N-Butyl-2-(hydroxy(phenyl)methyl)benzamide **22a**

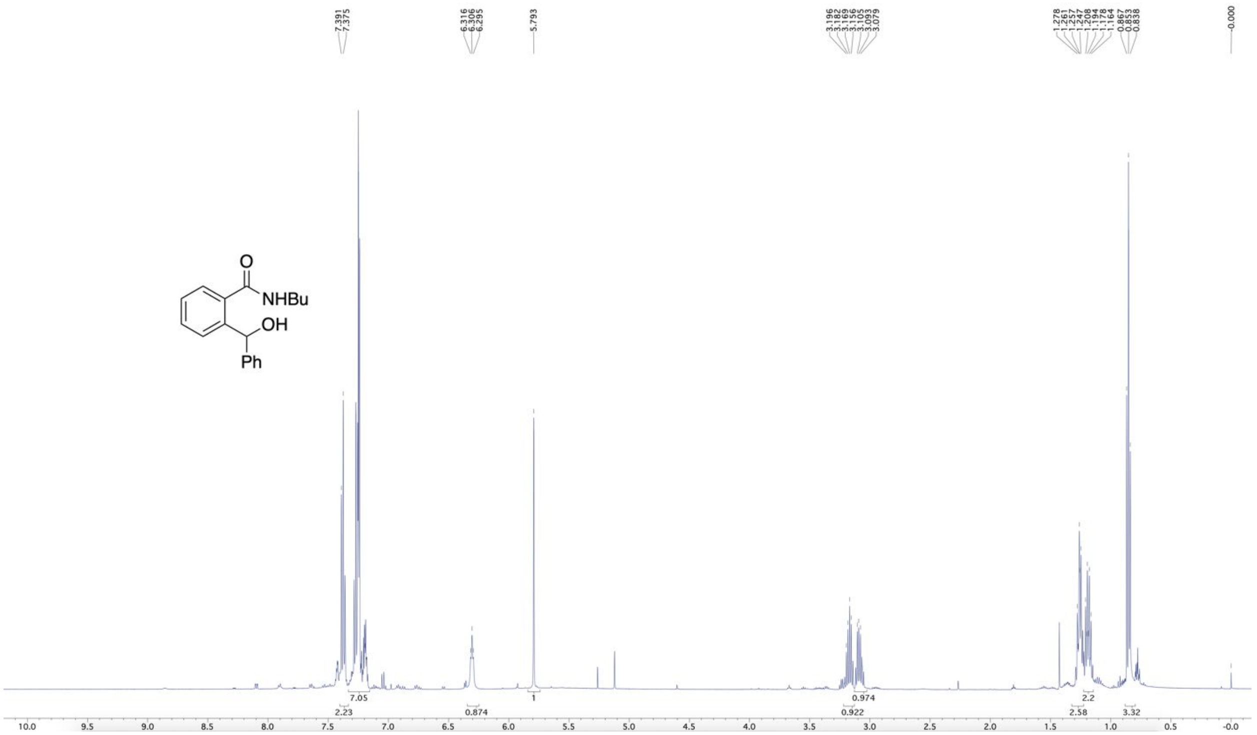

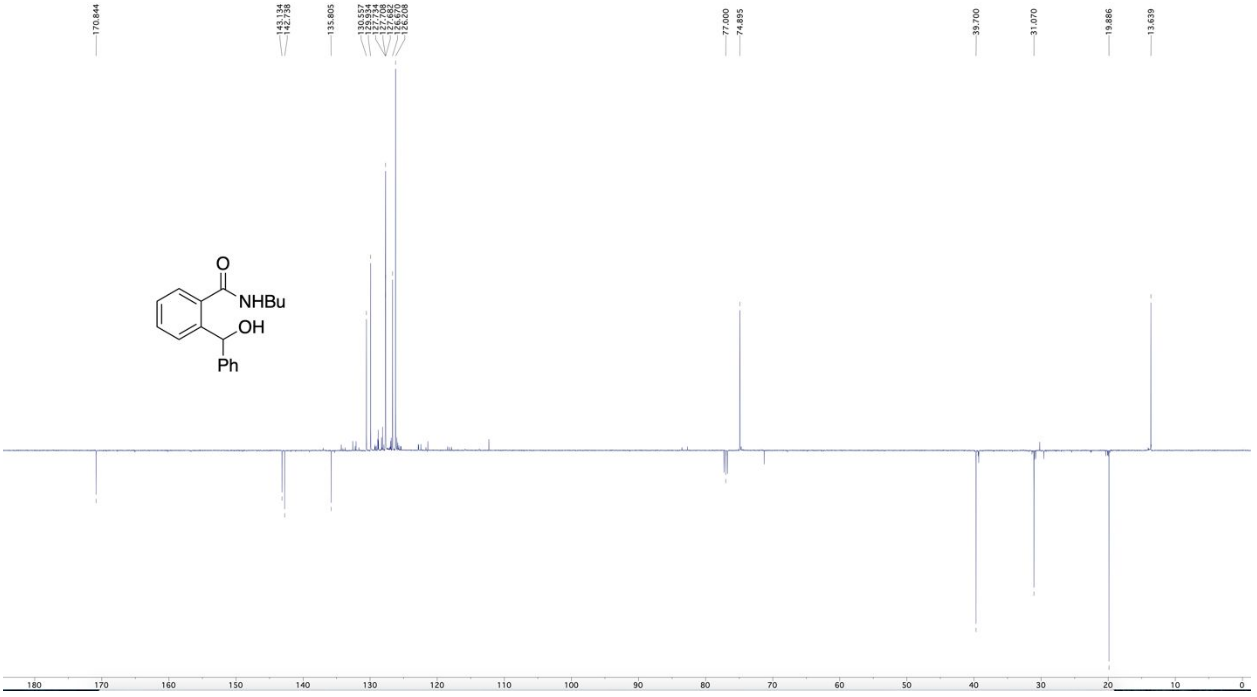

N-Butyl-2-(hydroxy(*p*-tolyl)methyl)benzamide **22b**

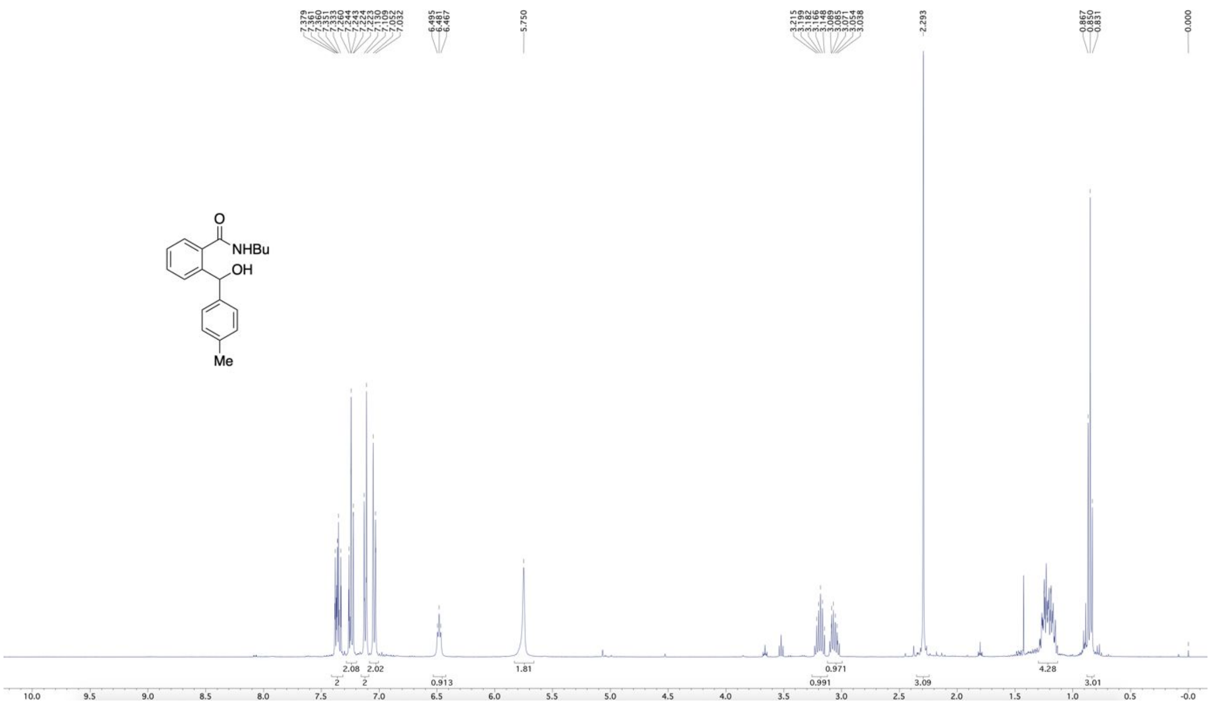

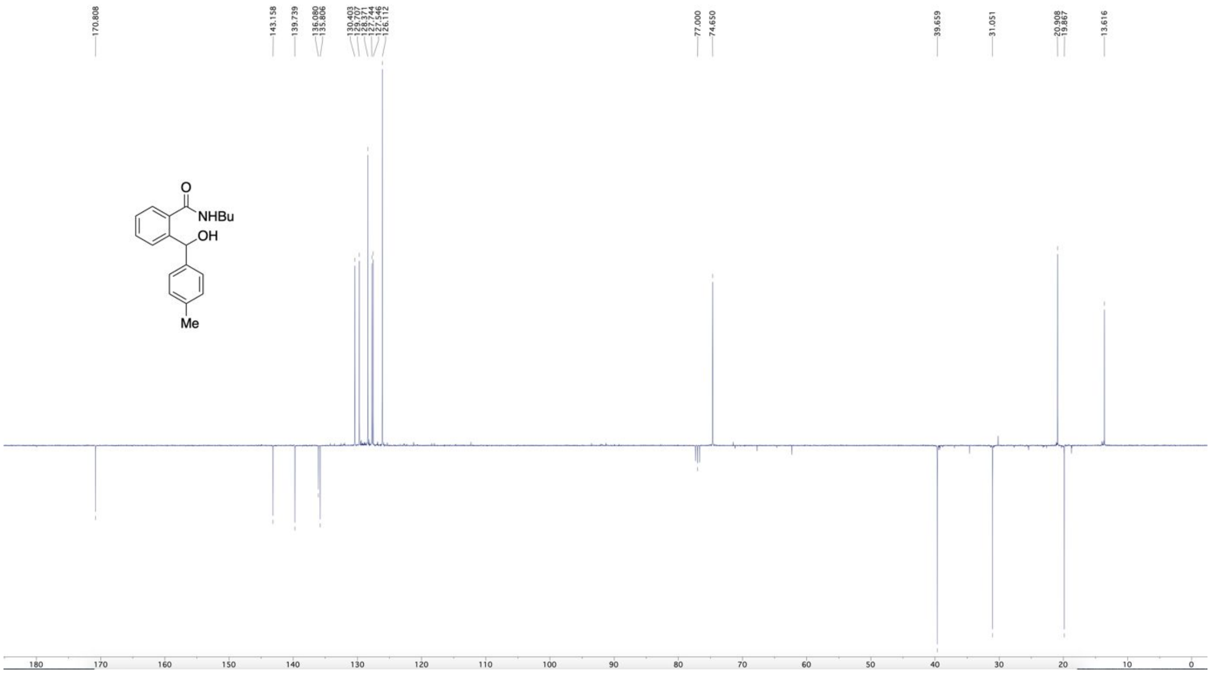

N-Butyl-2-(hydroxy(*o*-tolyl)methyl)benzamide **22c**

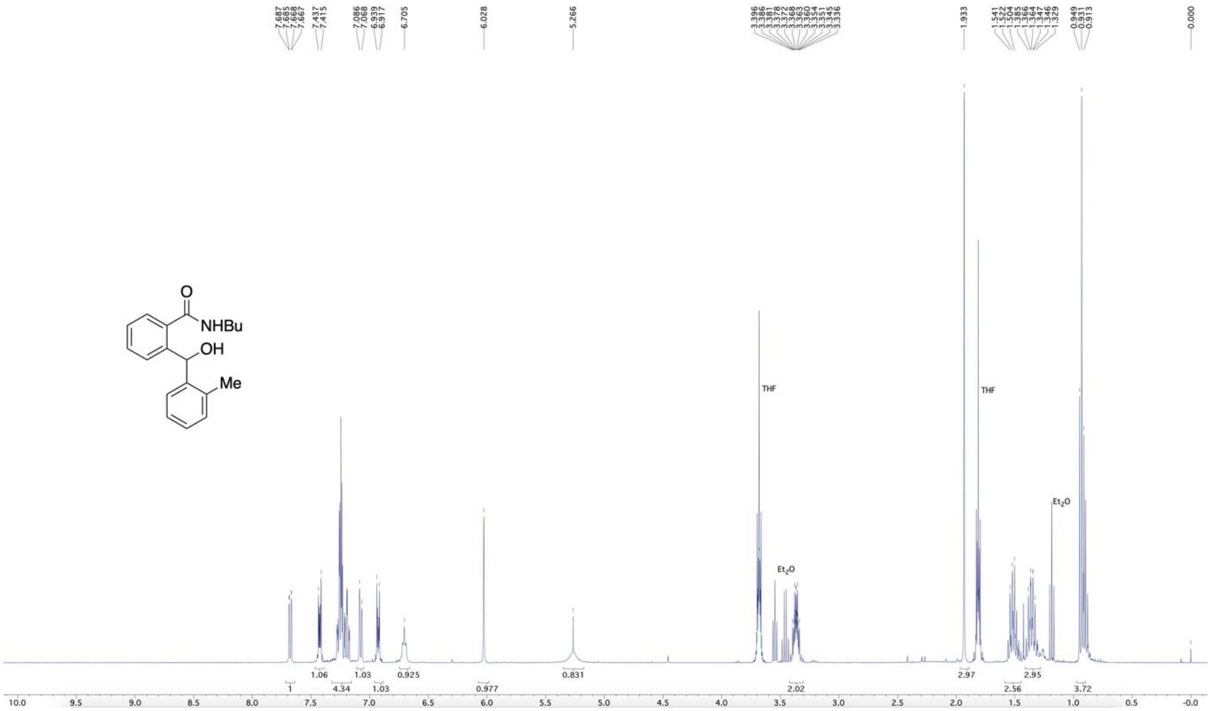

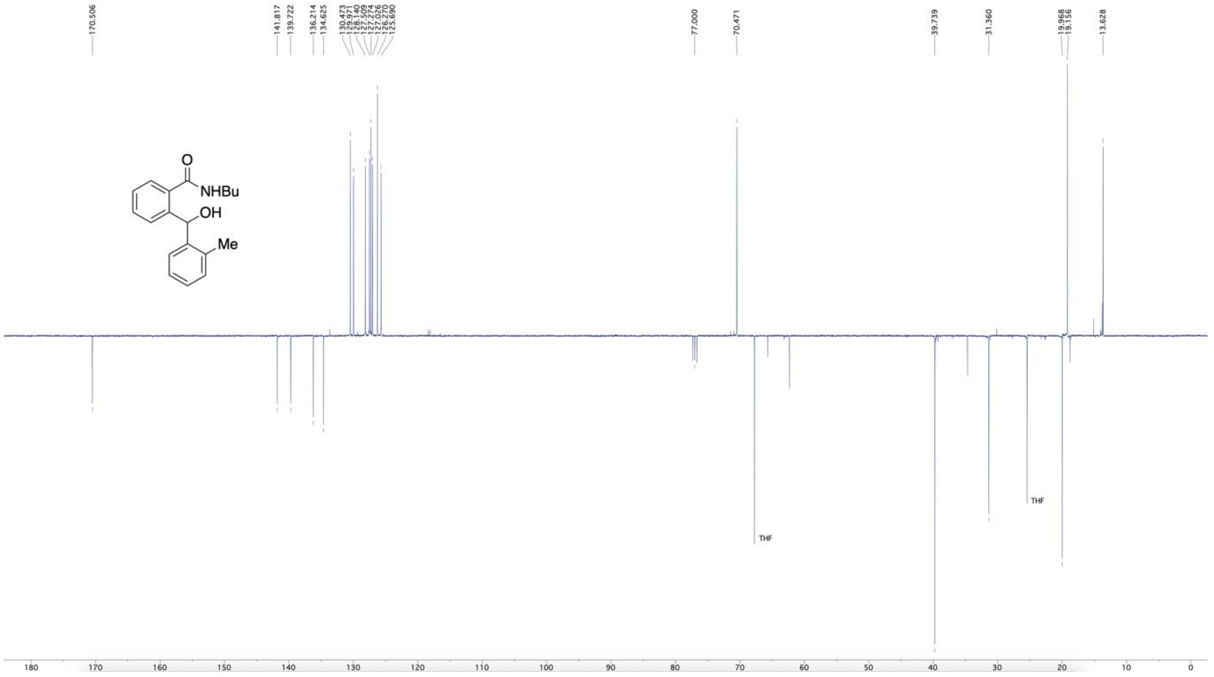

N-Butyl-2-(hydroxy(4-methoxyphenyl)methyl)benzamide **22d**

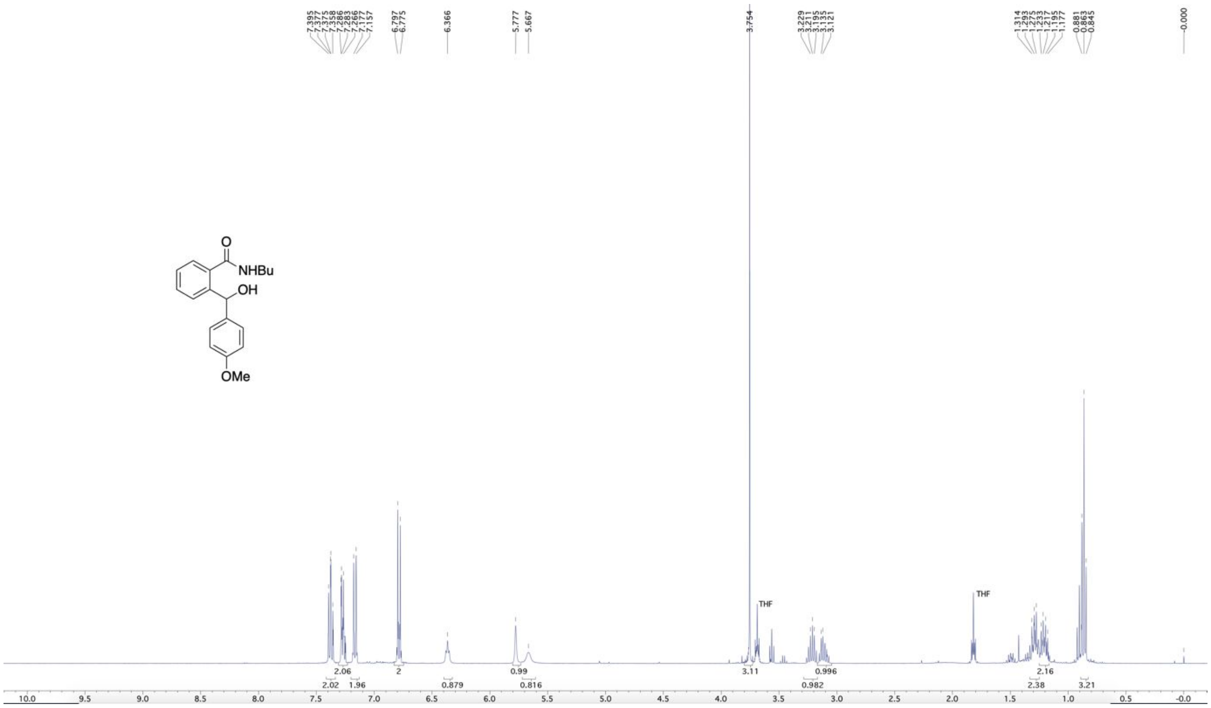

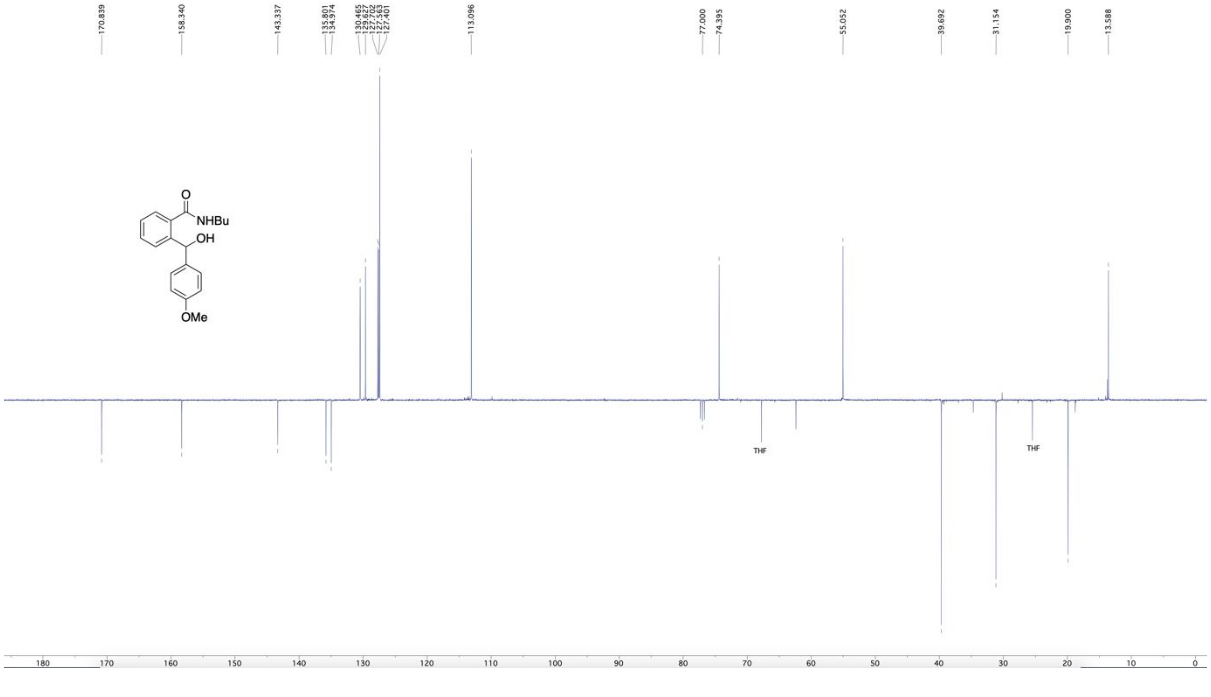

N-Butyl-2-(hydroxy(3-methoxyphenyl)methyl)benzamide **22e**

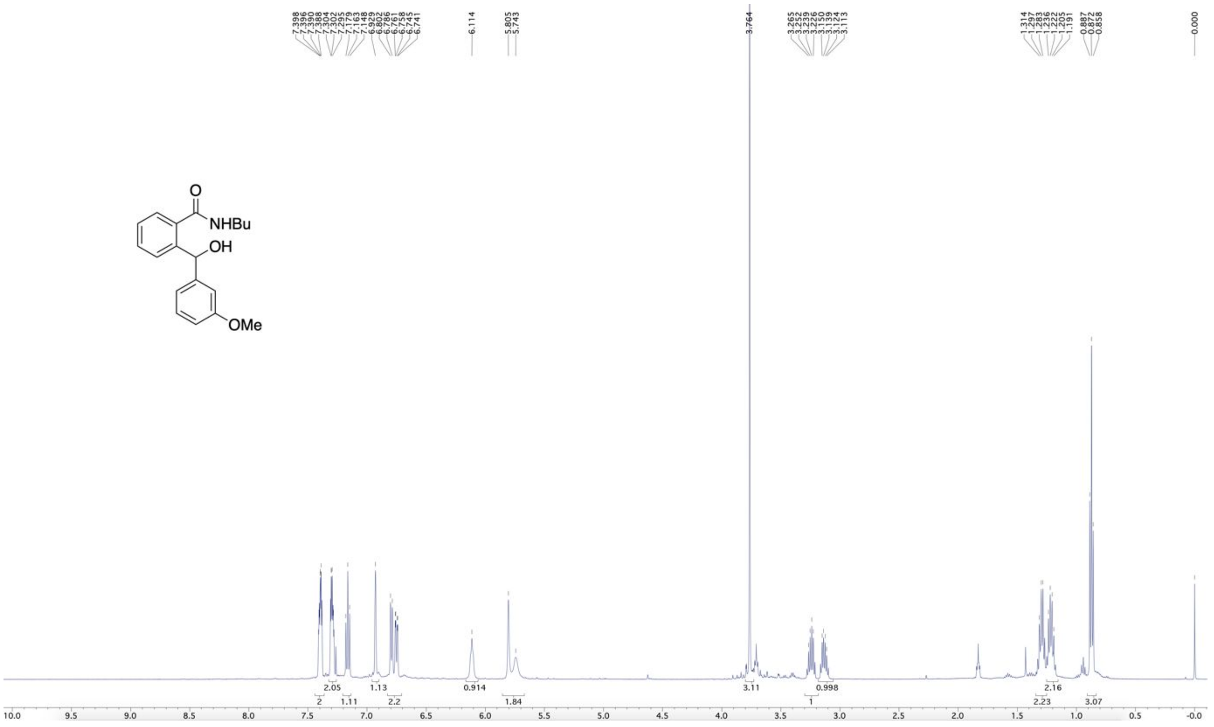

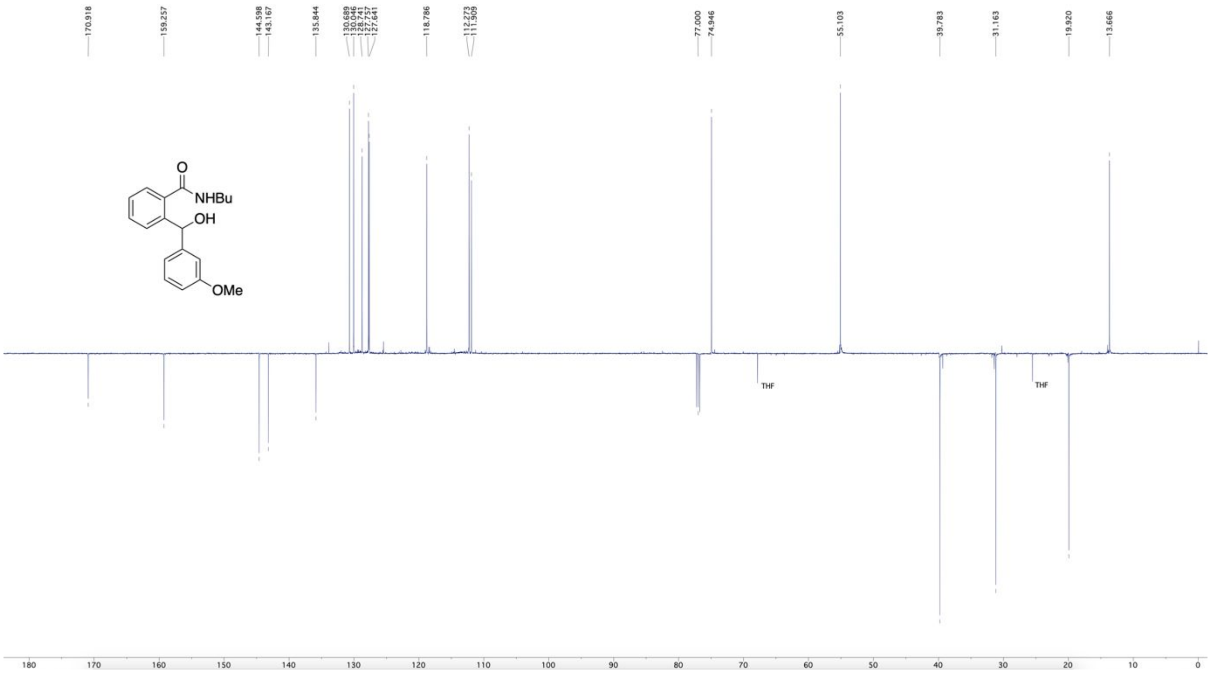

N-Butyl-2-(hydroxy(2-methoxyphenyl)methyl)benzamide **22f**

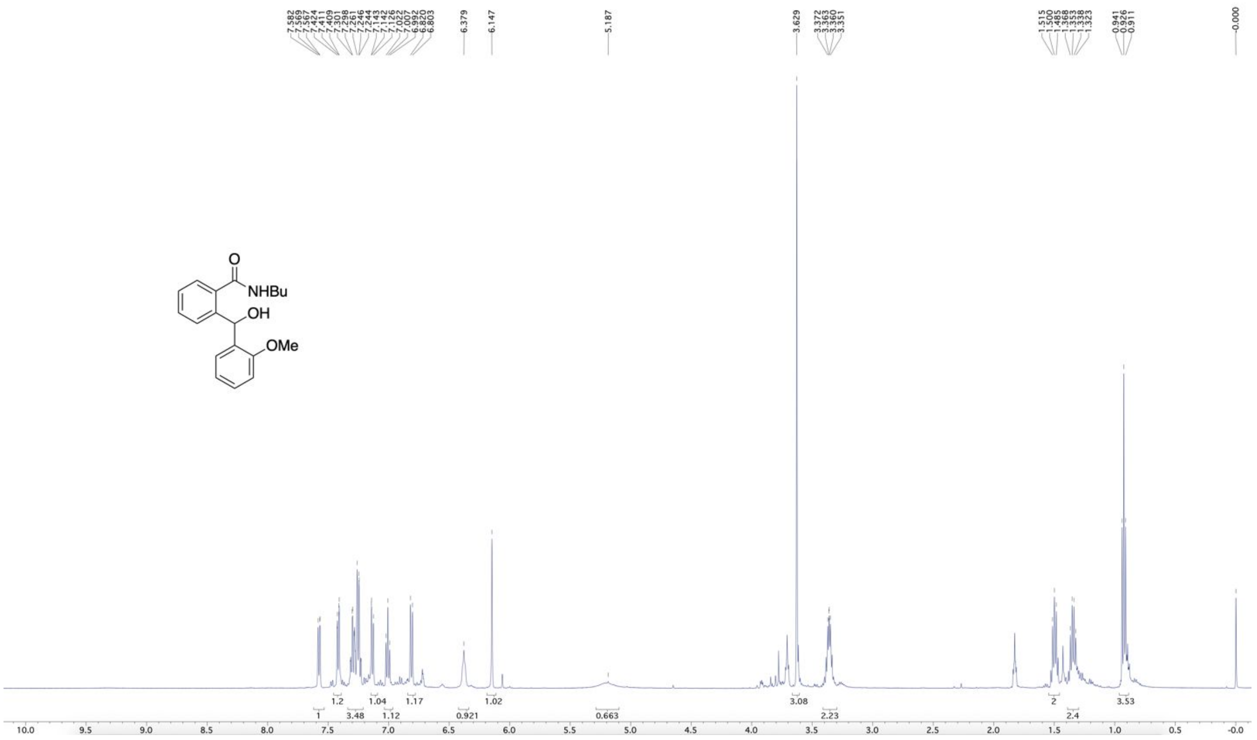

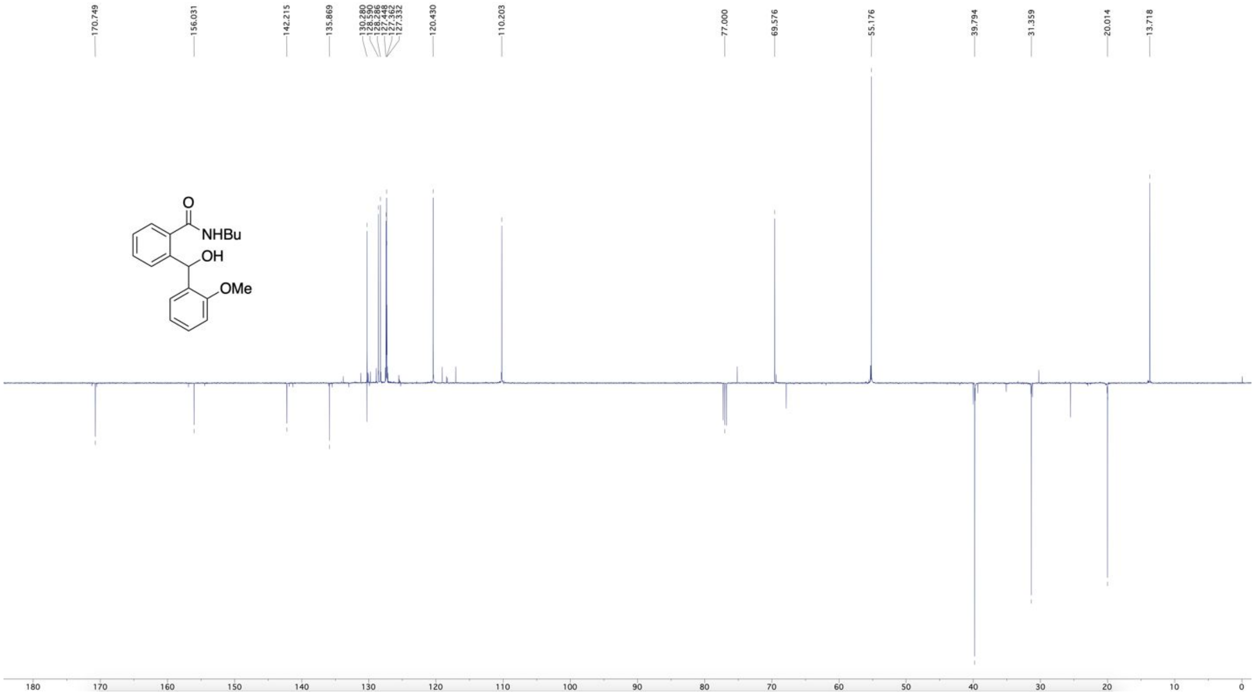

N-Butyl-2-(hydroxy(2-methoxynaphthalen-1-yl)methyl)benzamide **22g**

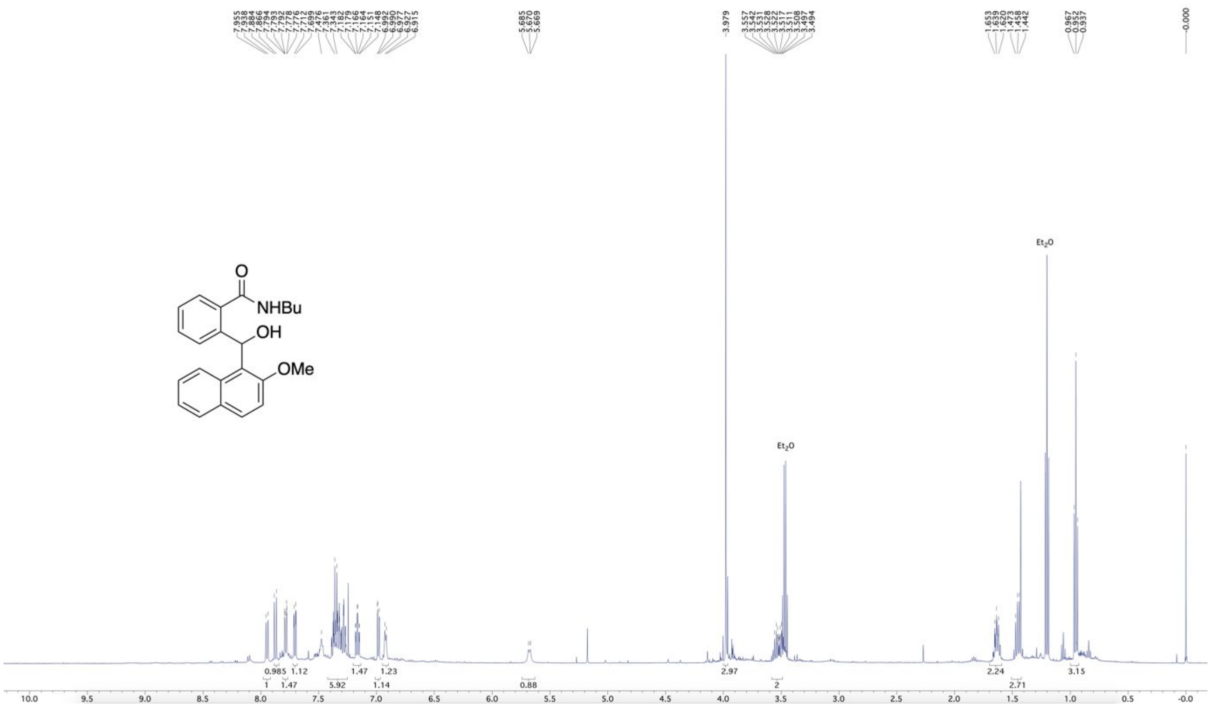

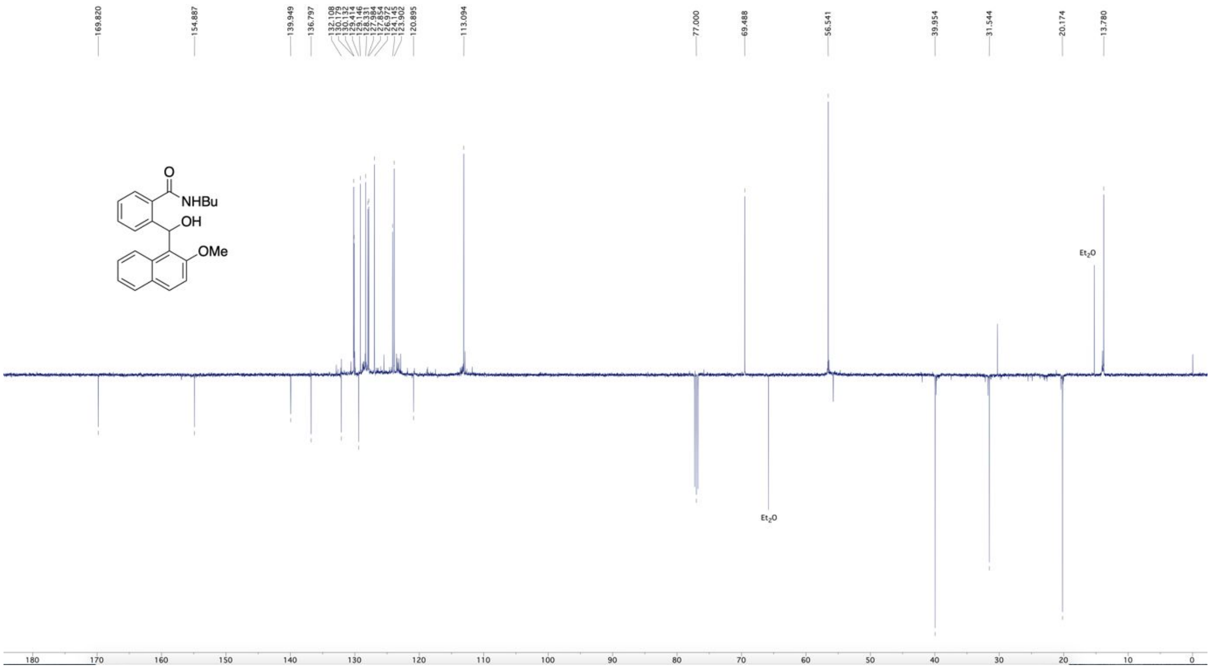

N-Butyl-2-((4-fluorophenyl)(hydroxy)methyl)benzamide **22h**

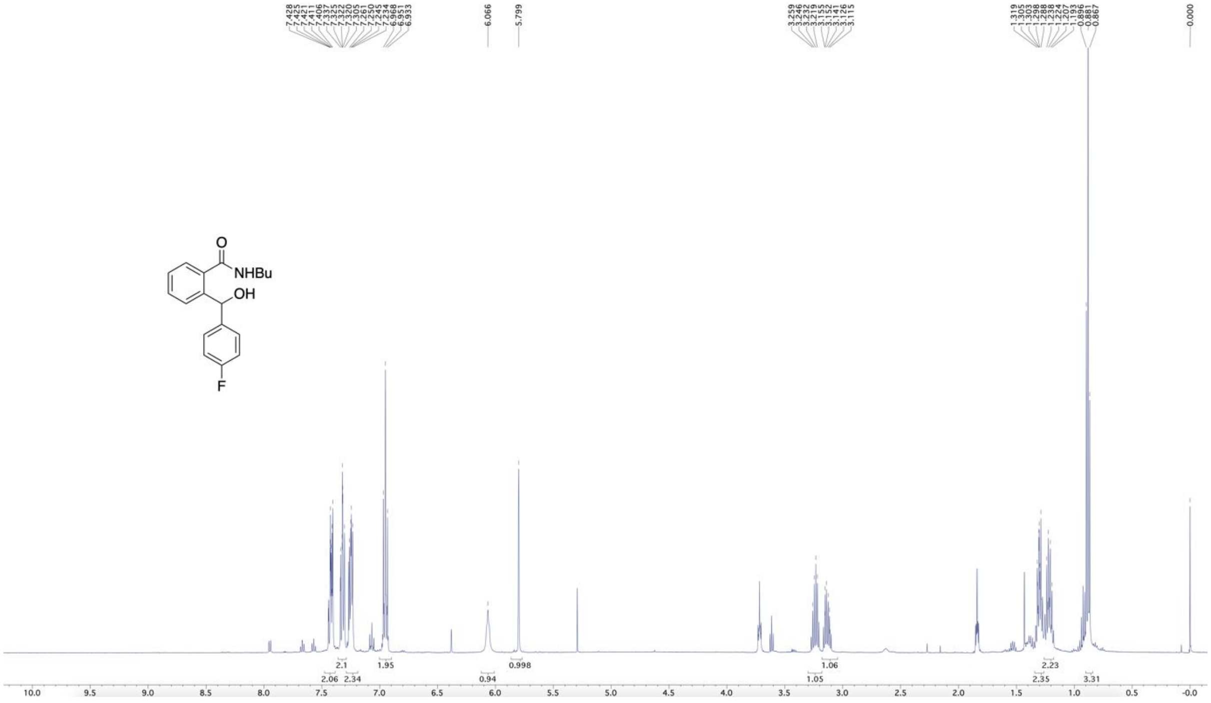

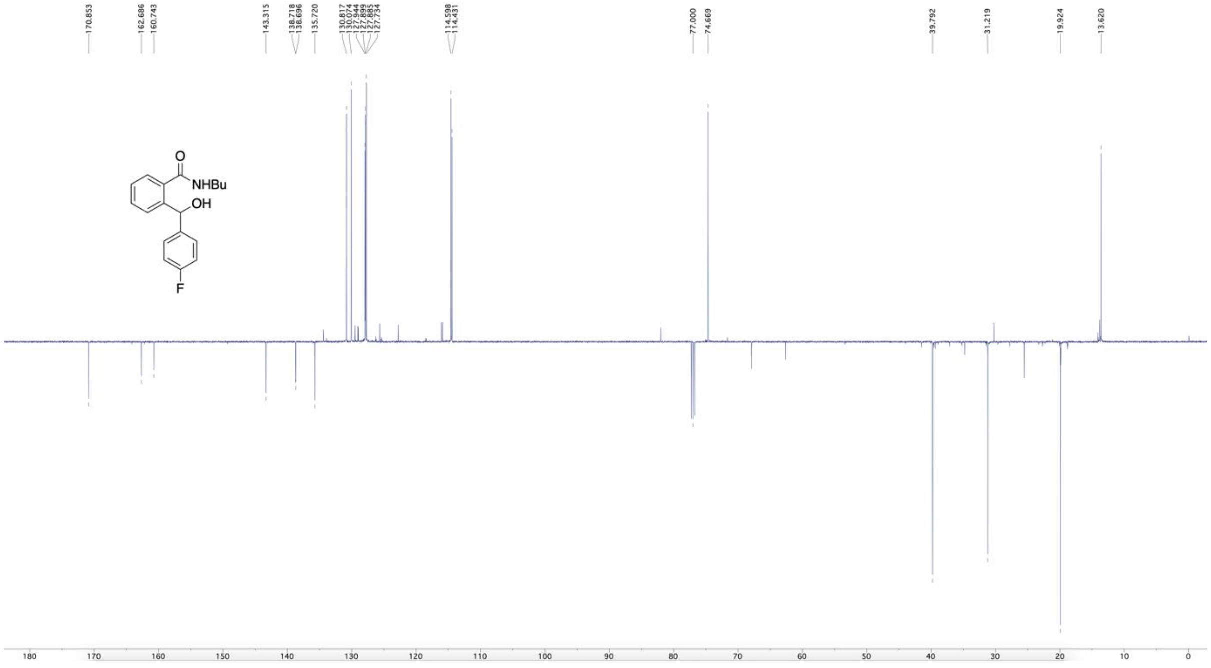

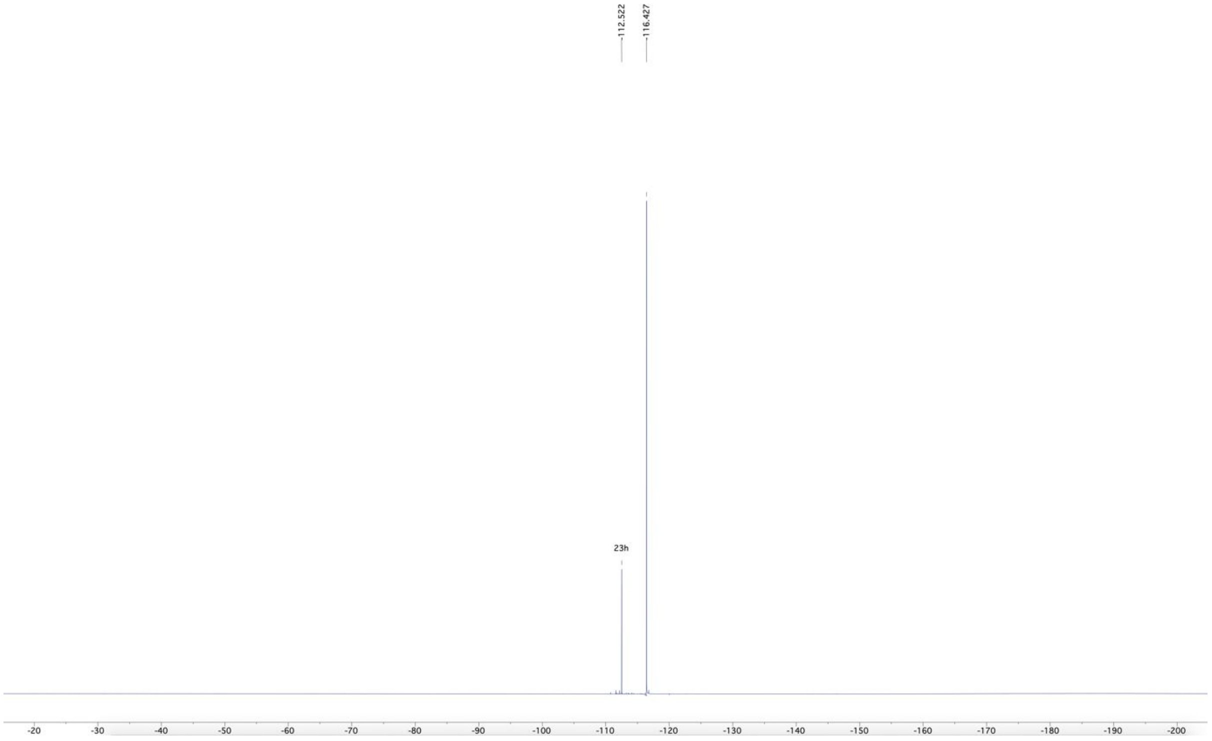

N-Butyl-2-((3-fluorophenyl)(hydroxy)methyl)benzamide **22i**

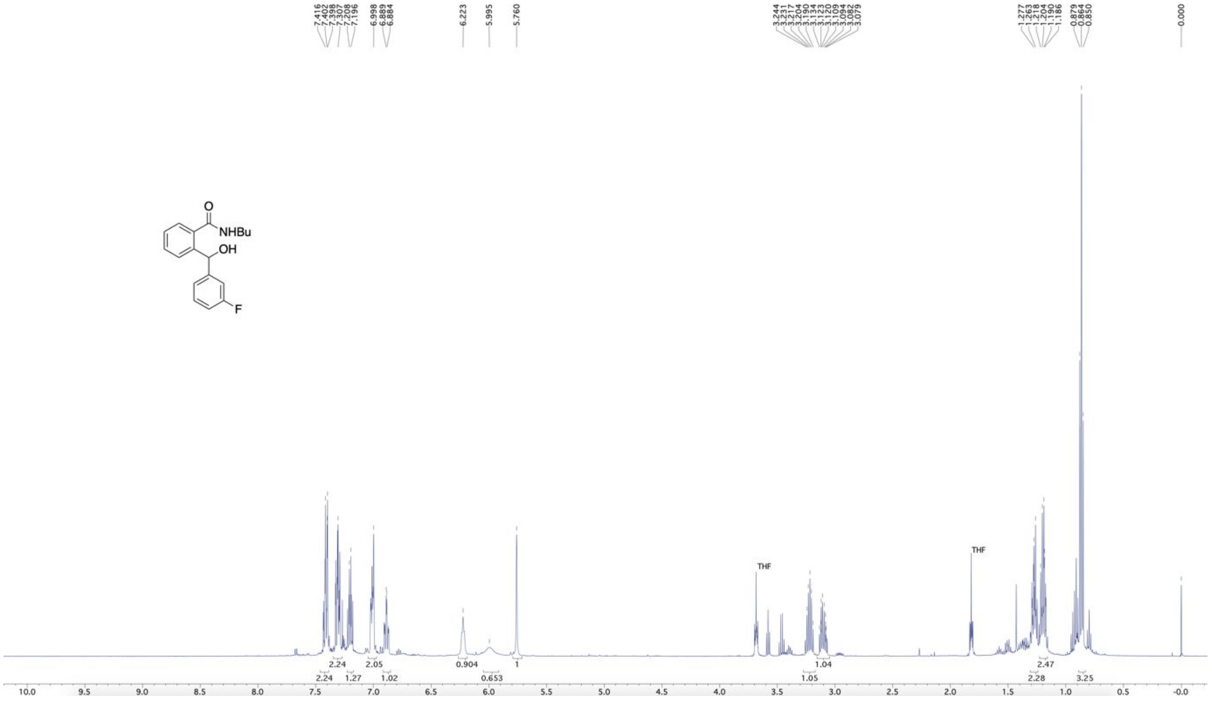

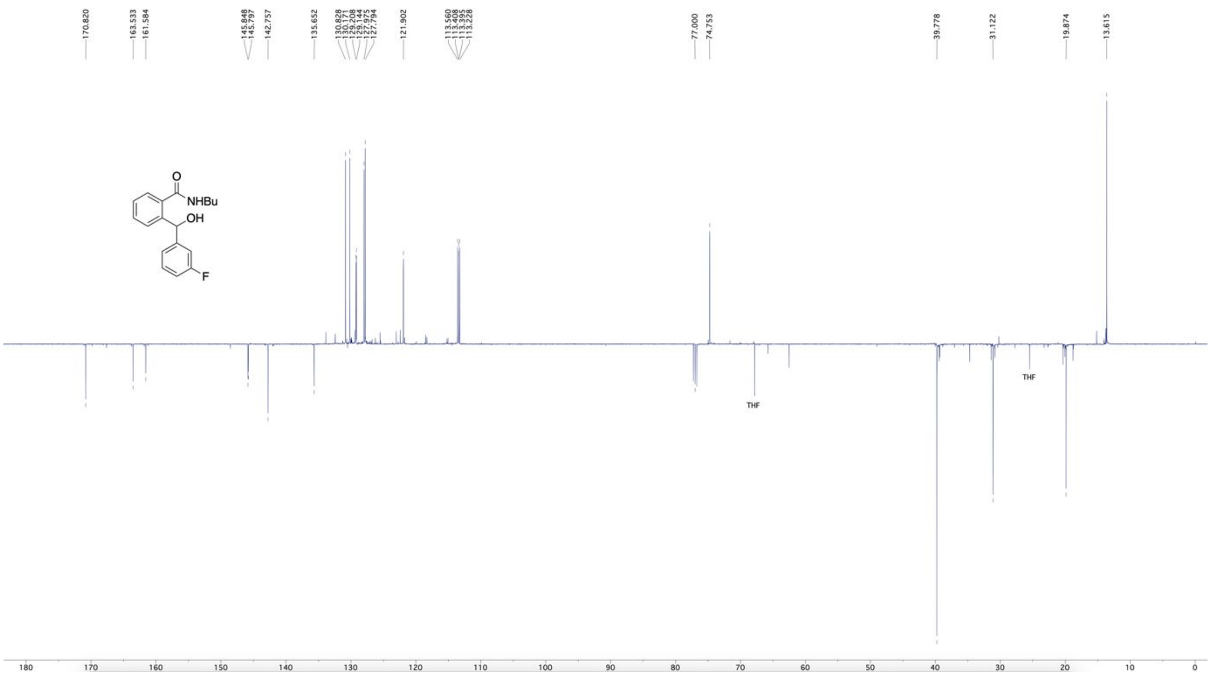

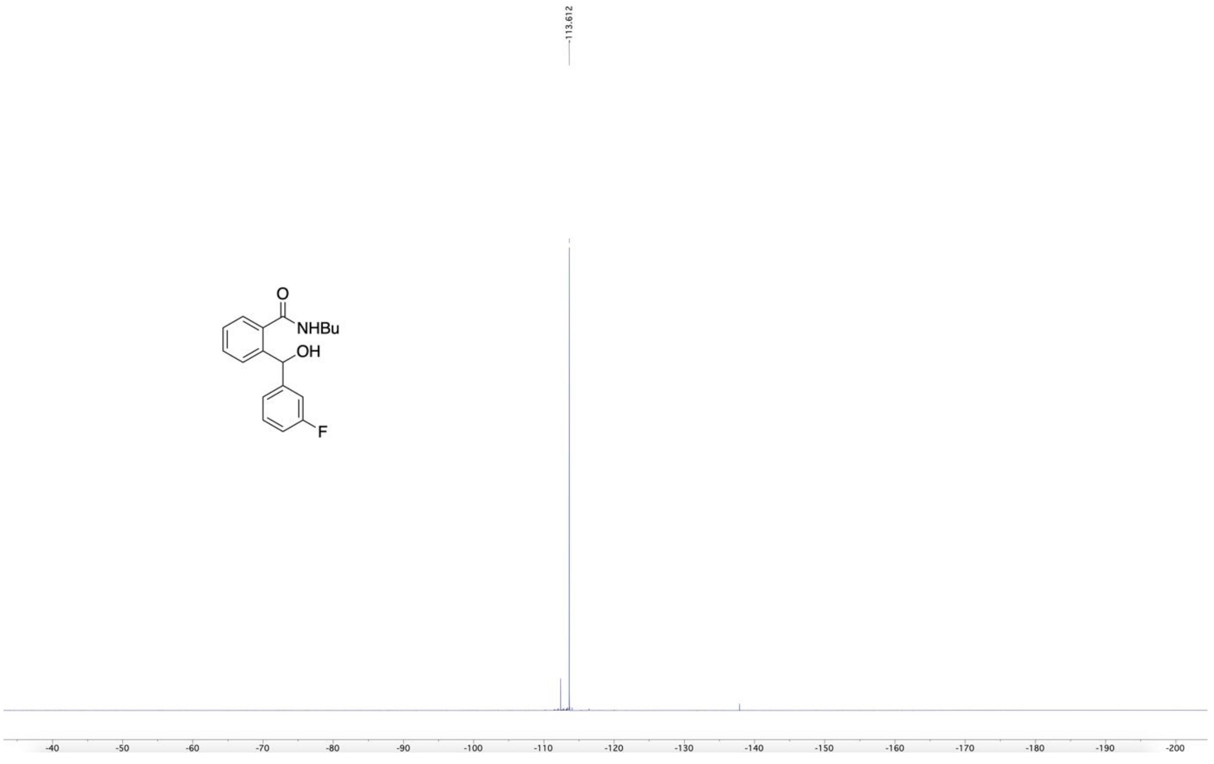

N-Butyl-2-((2-fluorophenyl)(hydroxy)methyl)benzamide **22j**

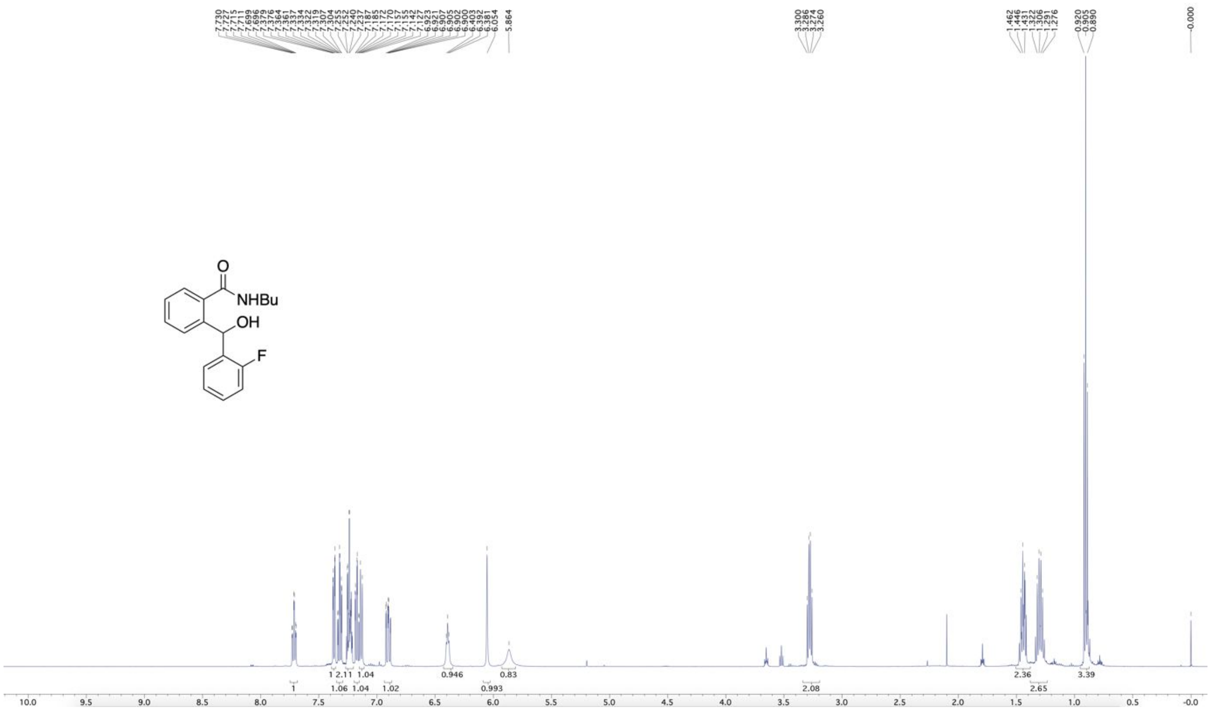

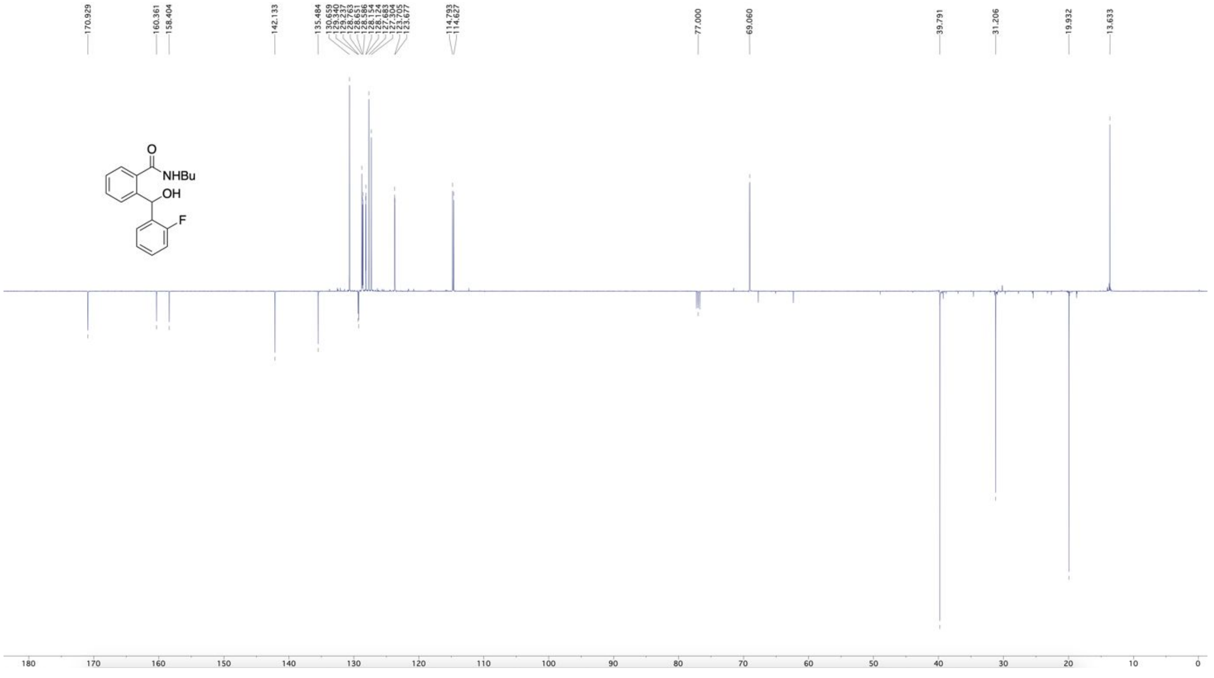

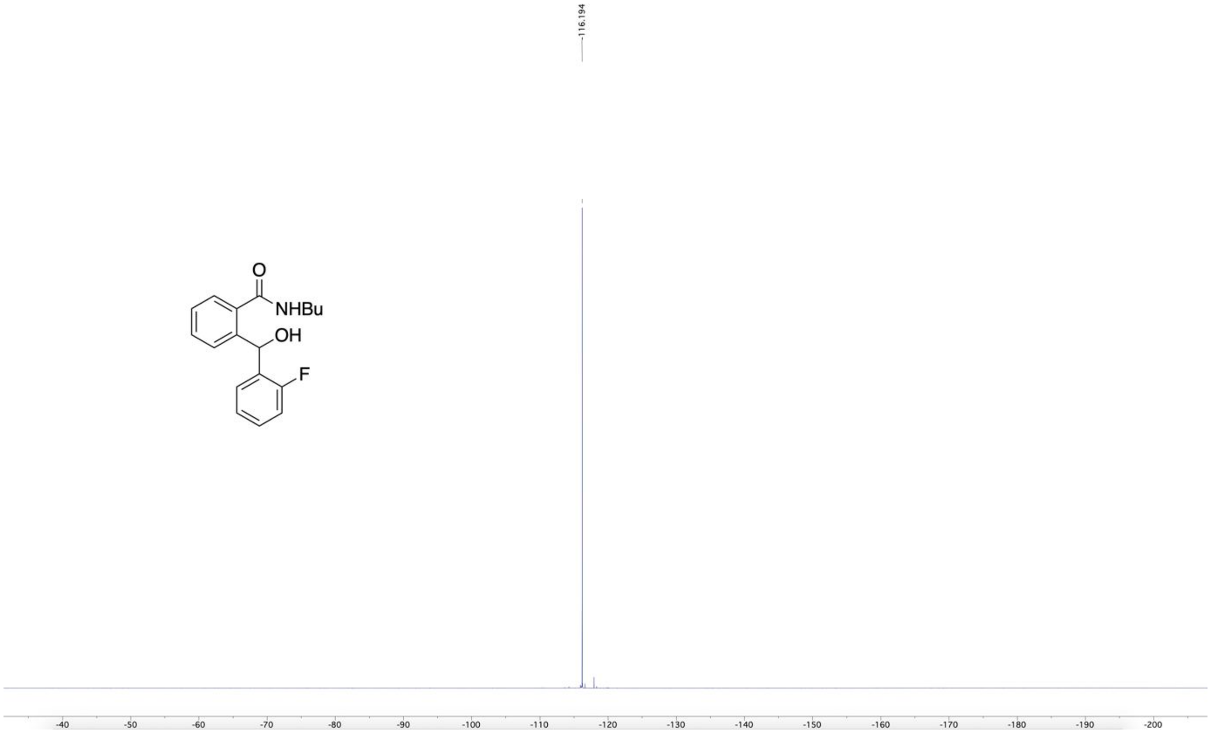

N-Butyl-2-(1-hydroxy-1-phenylethyl)benzamide **22s**

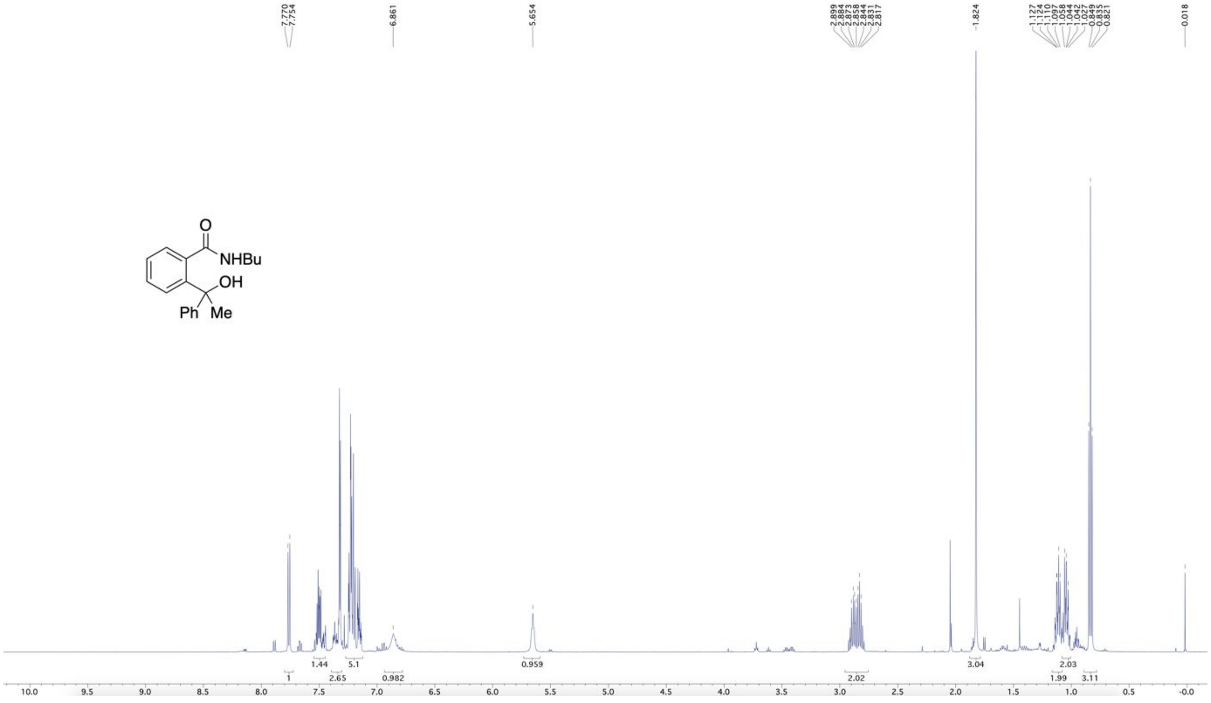

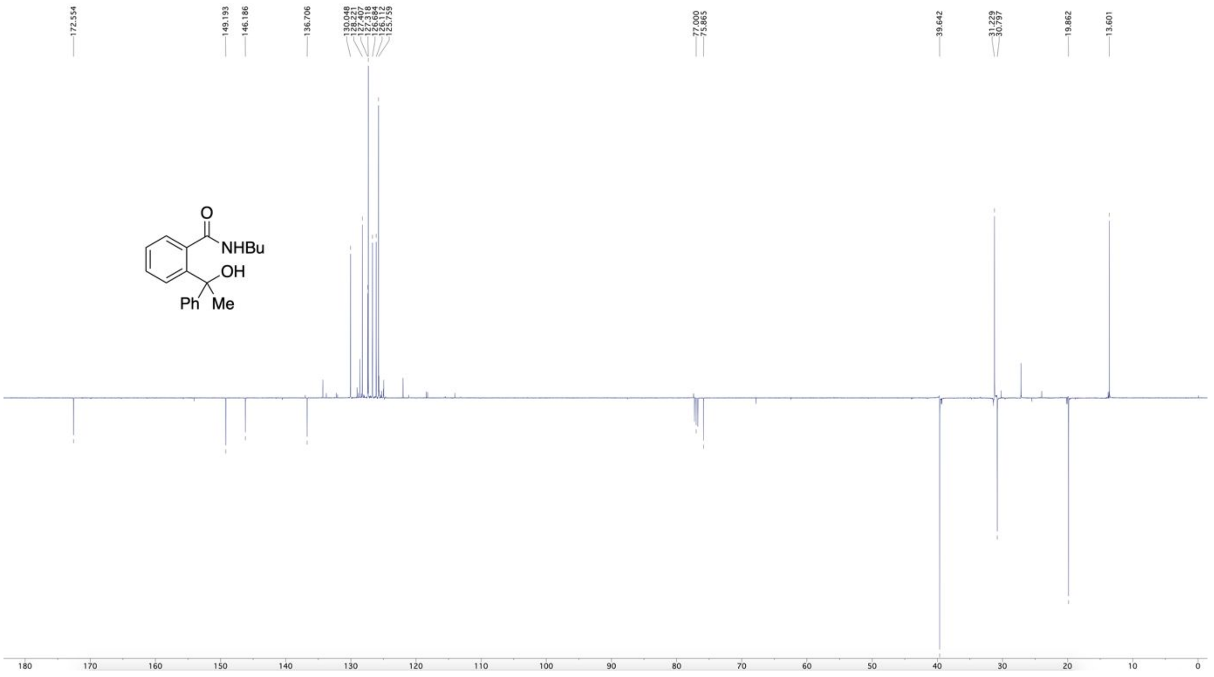

N-Butyl-2-(1-hydroxyallyl)benzamide **22t**

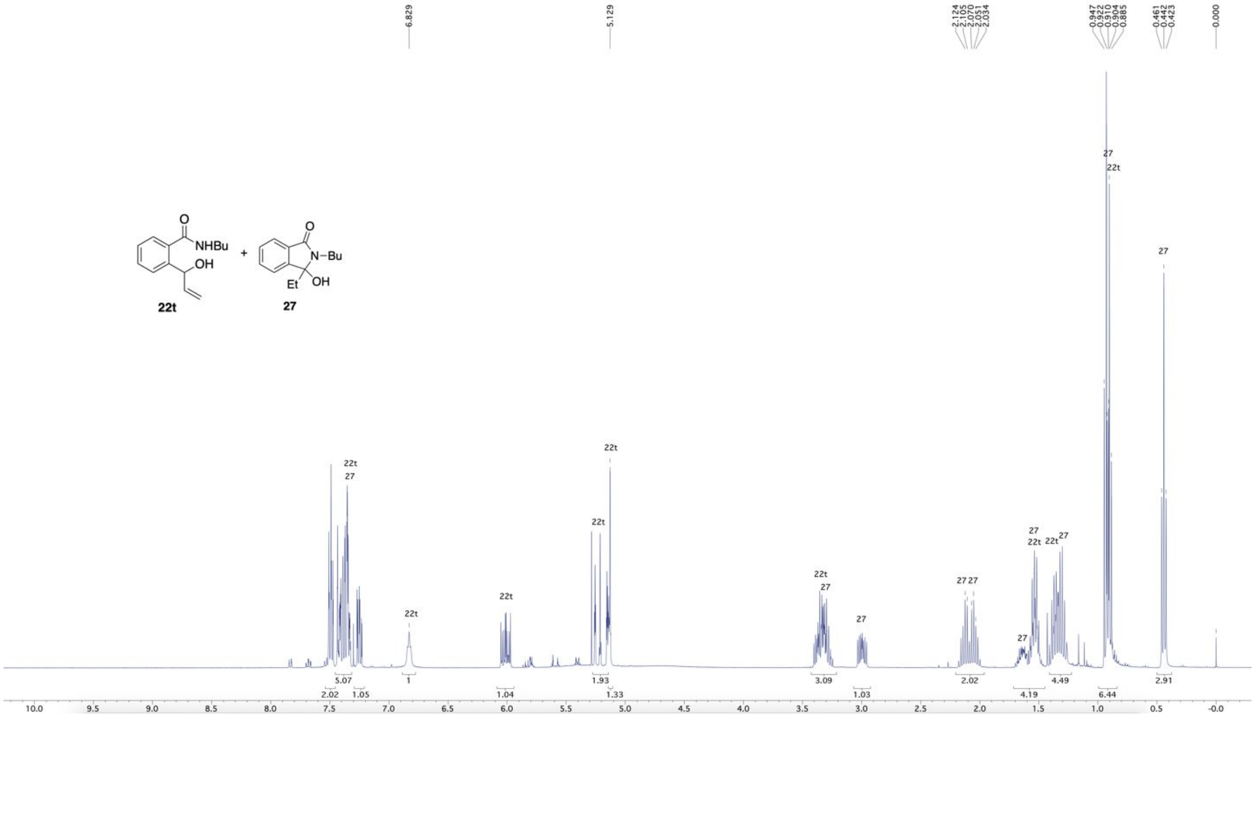

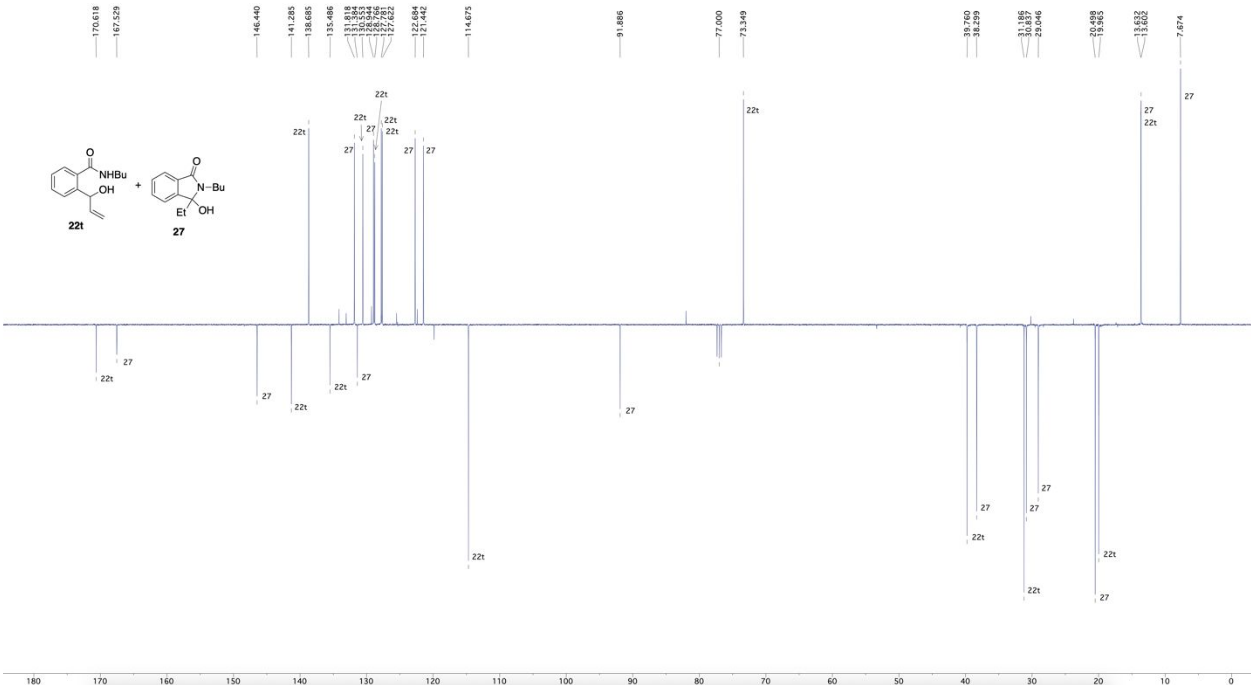

3-(2-Methoxynaphthalen-1-yl)phthalide **23g**

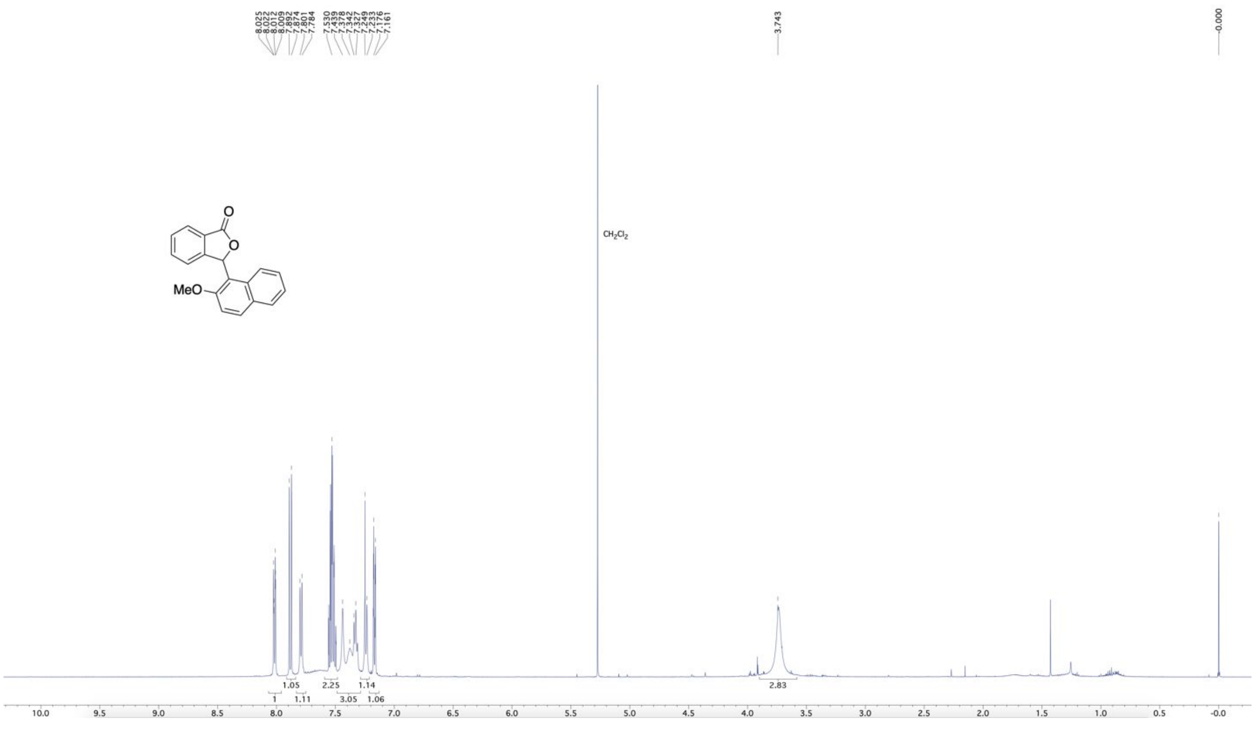

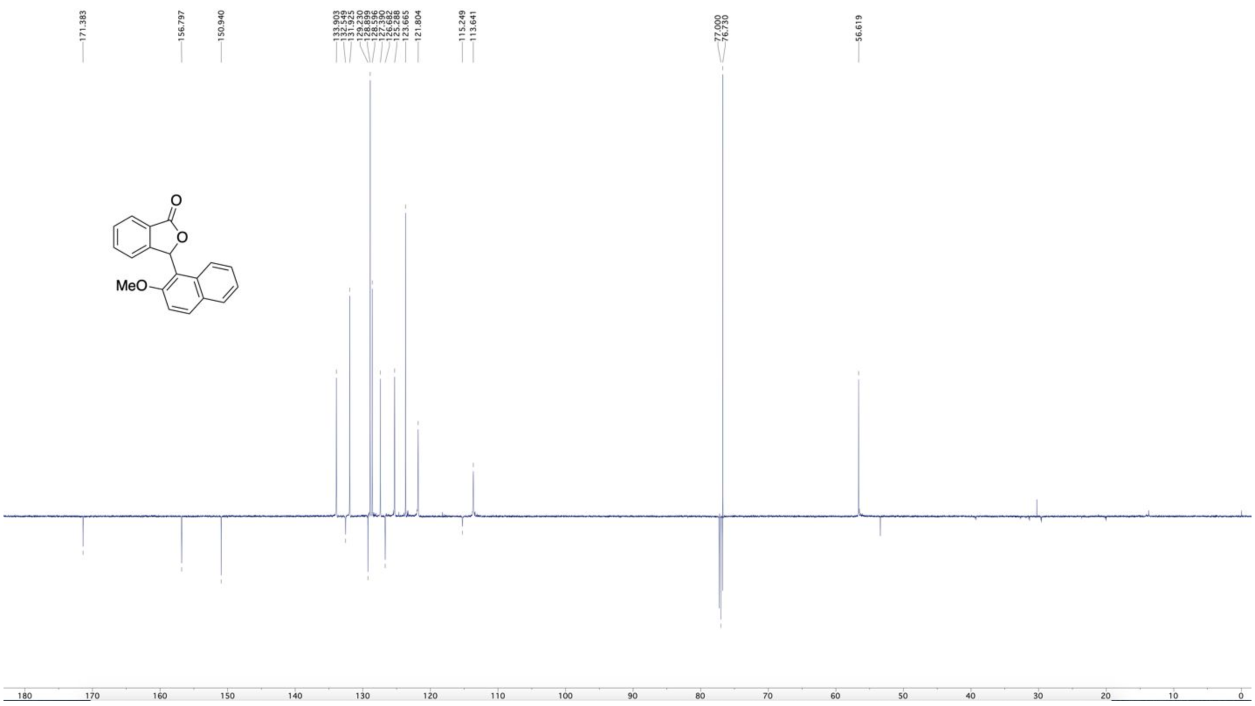

3-(2-Methylprop-1-en-1-yl)phthalide **23u**

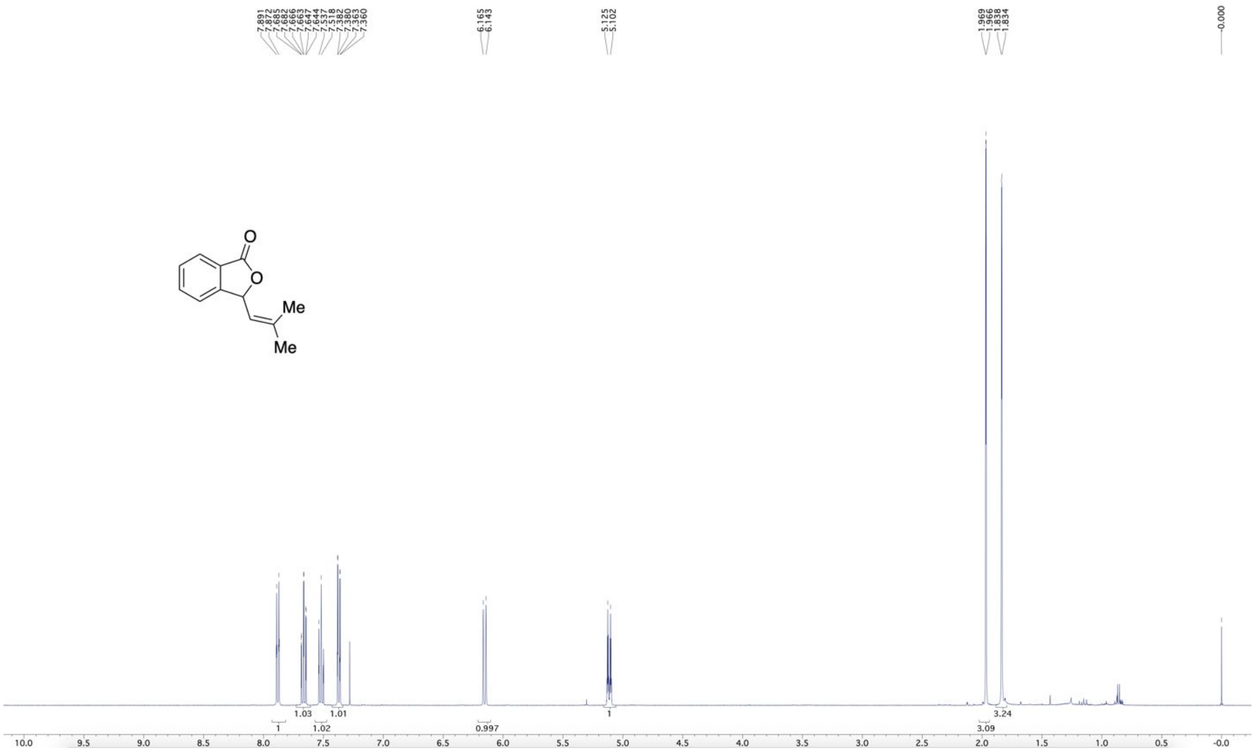

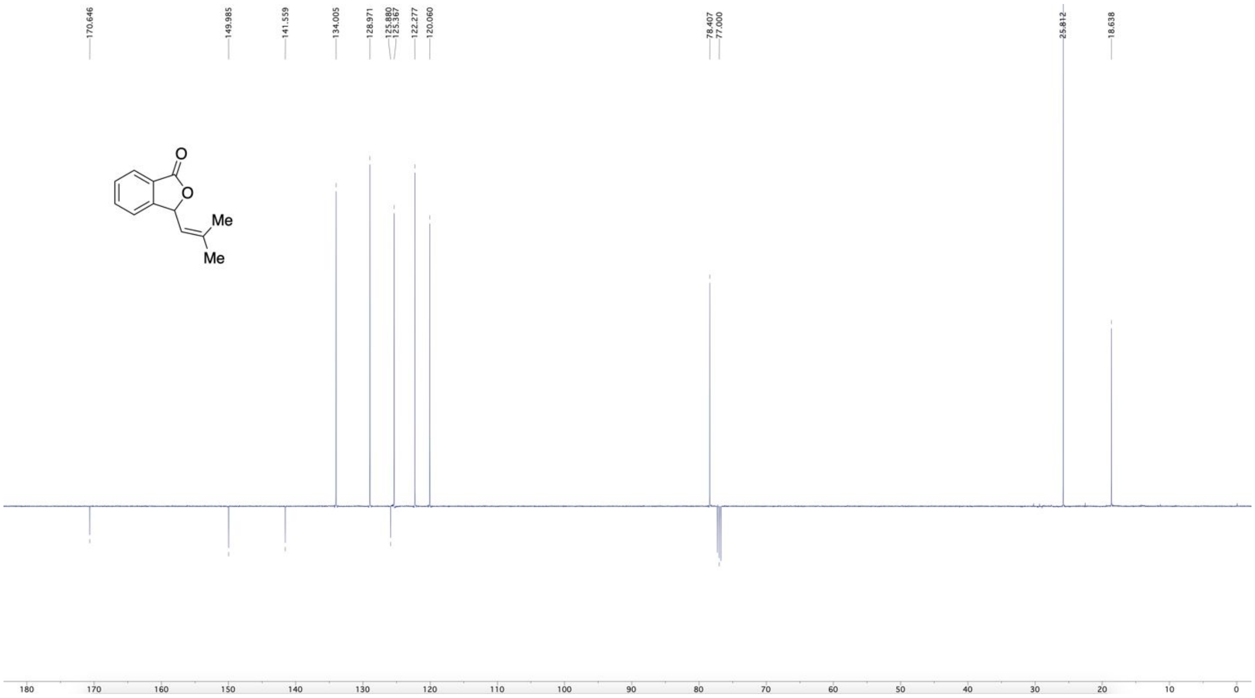

1-(Butylamino)anthraquinone **24j**

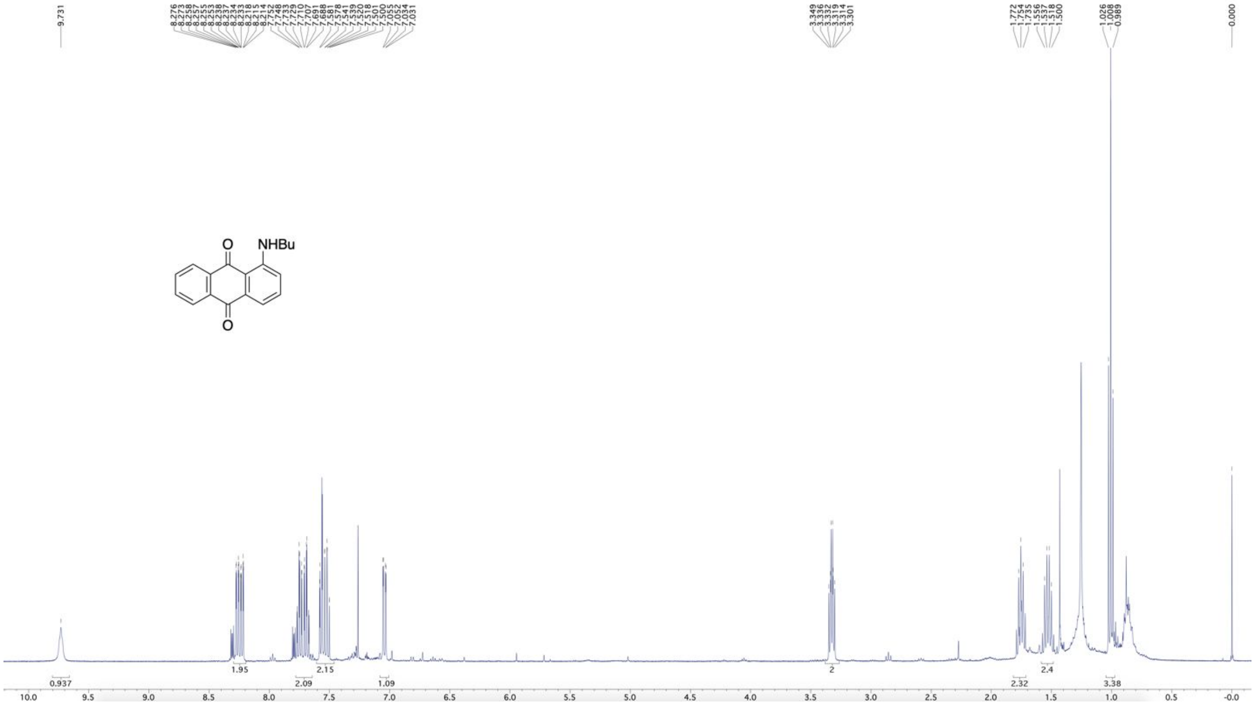

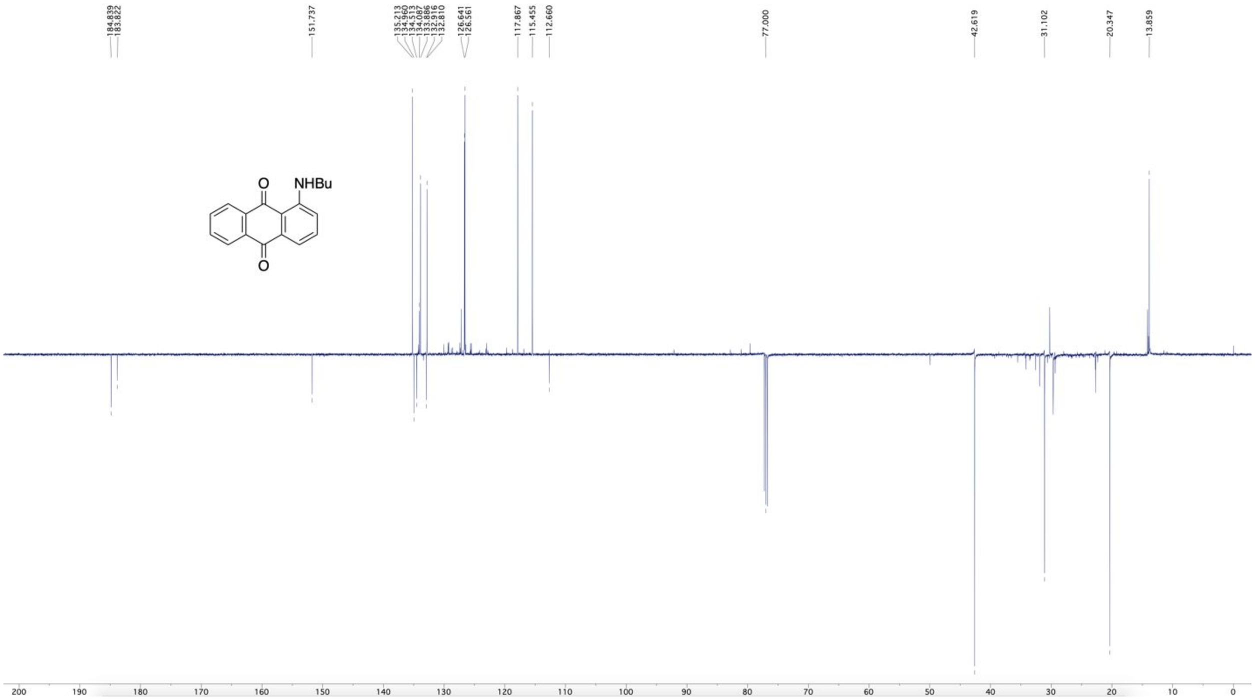

2-Butyl-3-ethyl-3-hydroxyisoindolin-1-one **27**

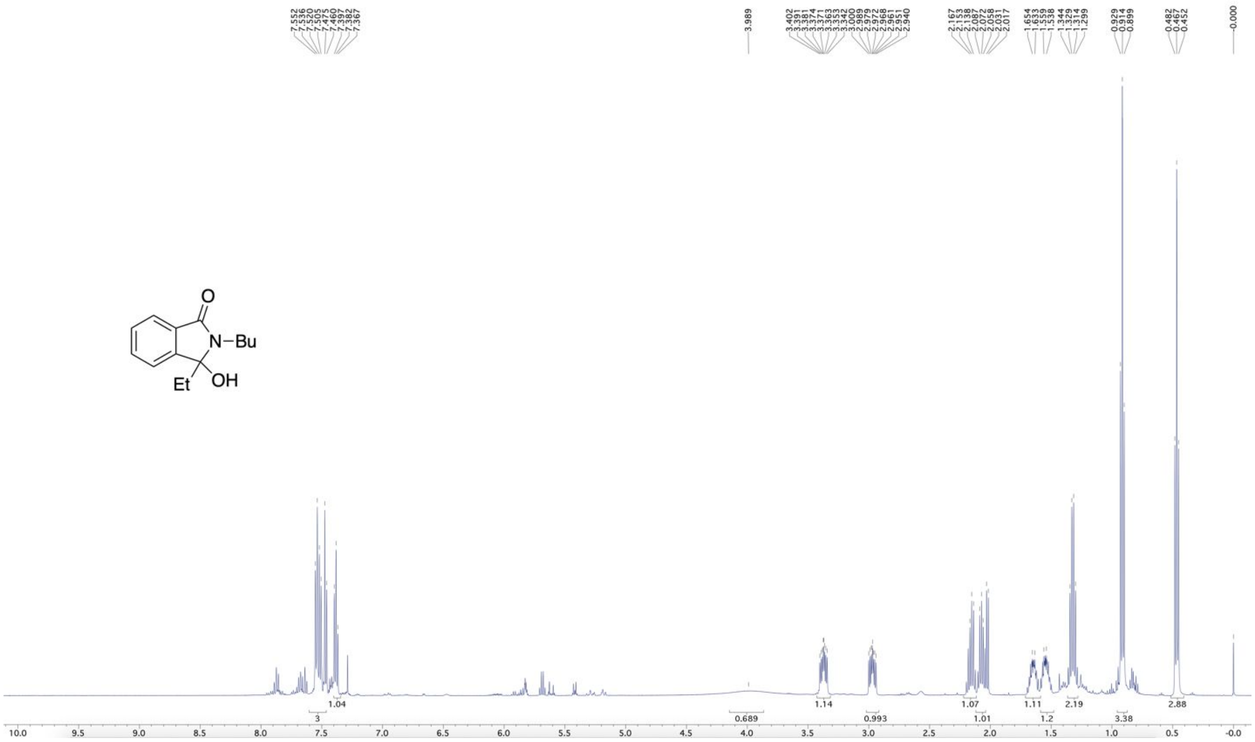

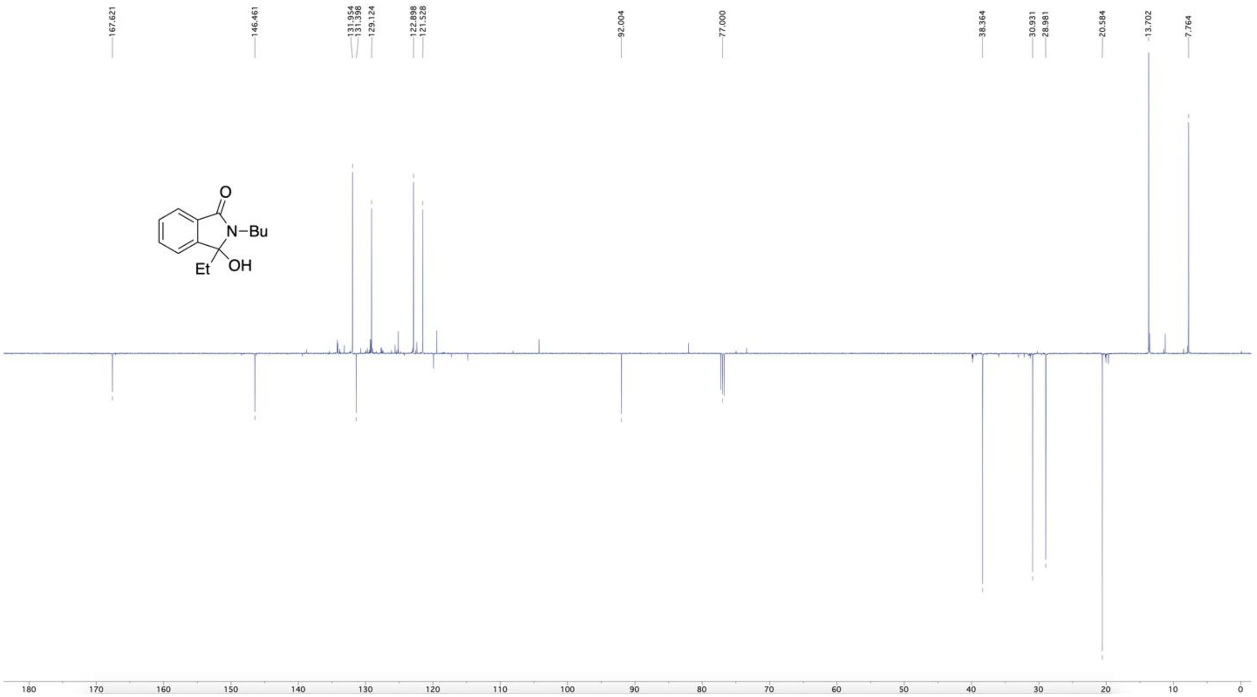

N-Butyl-4-((4-methylbenzyl)oxy)benzamide **29b**

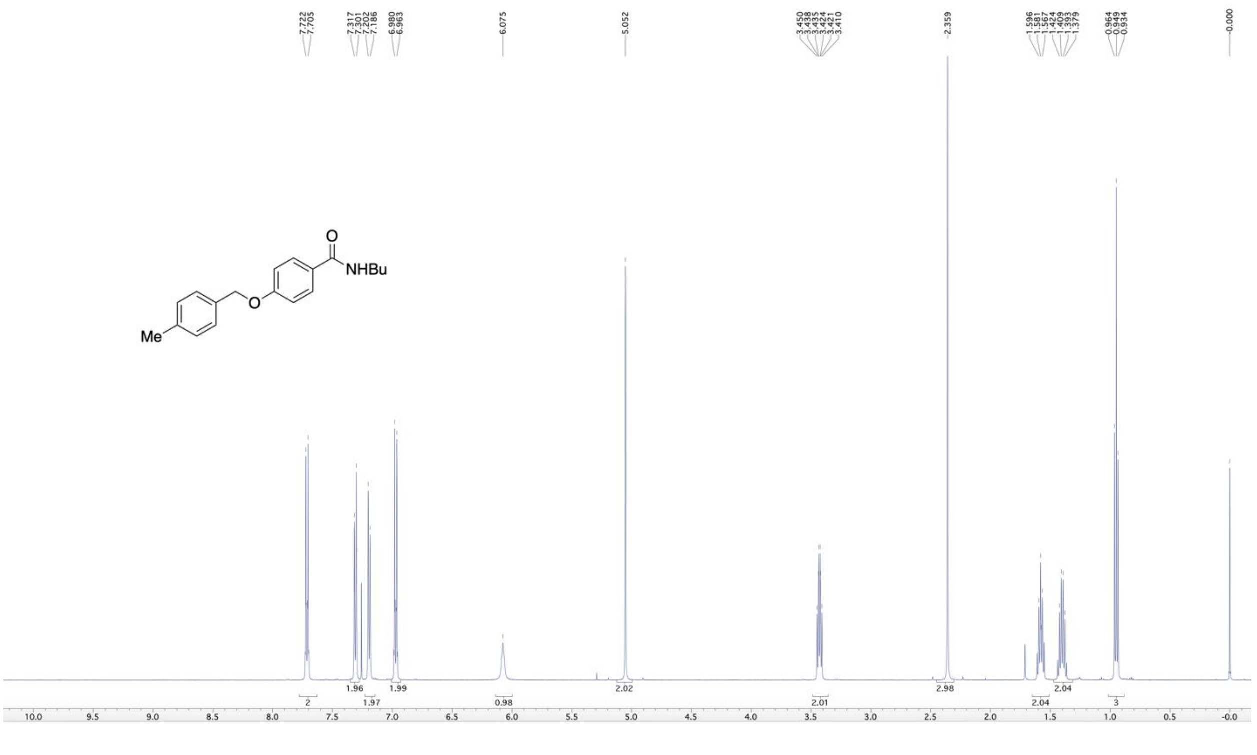

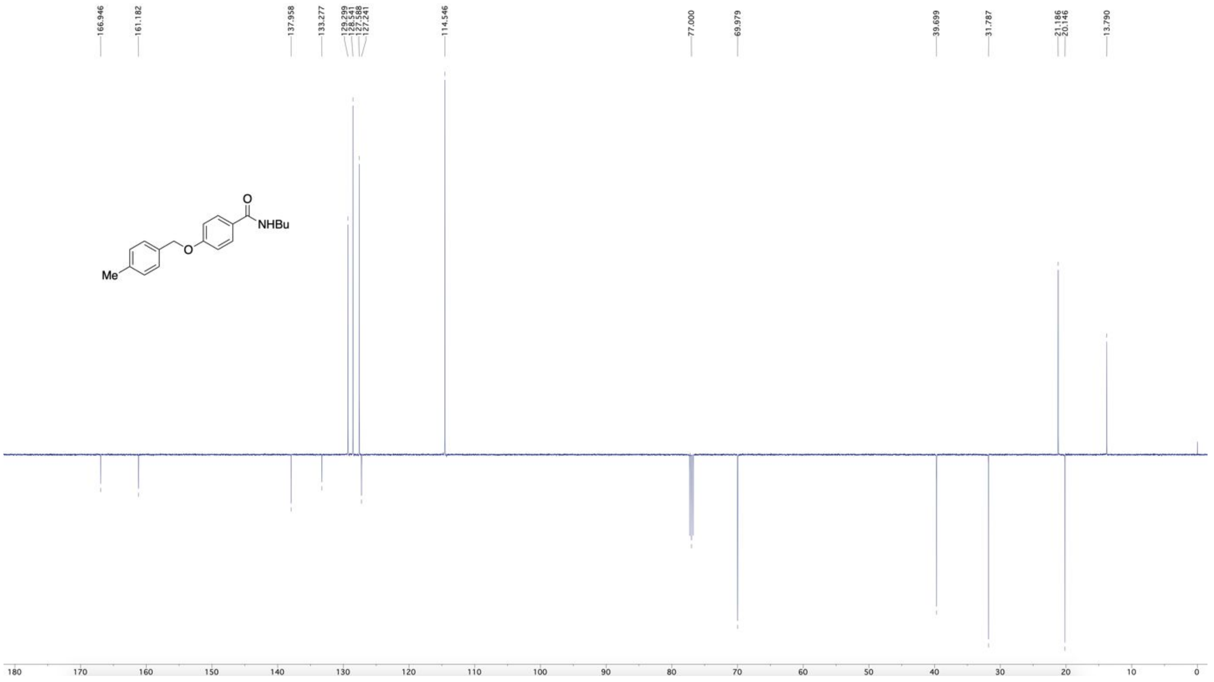

N-Butyl-4-((2-methylbenzyl)oxy)benzamide **29c**

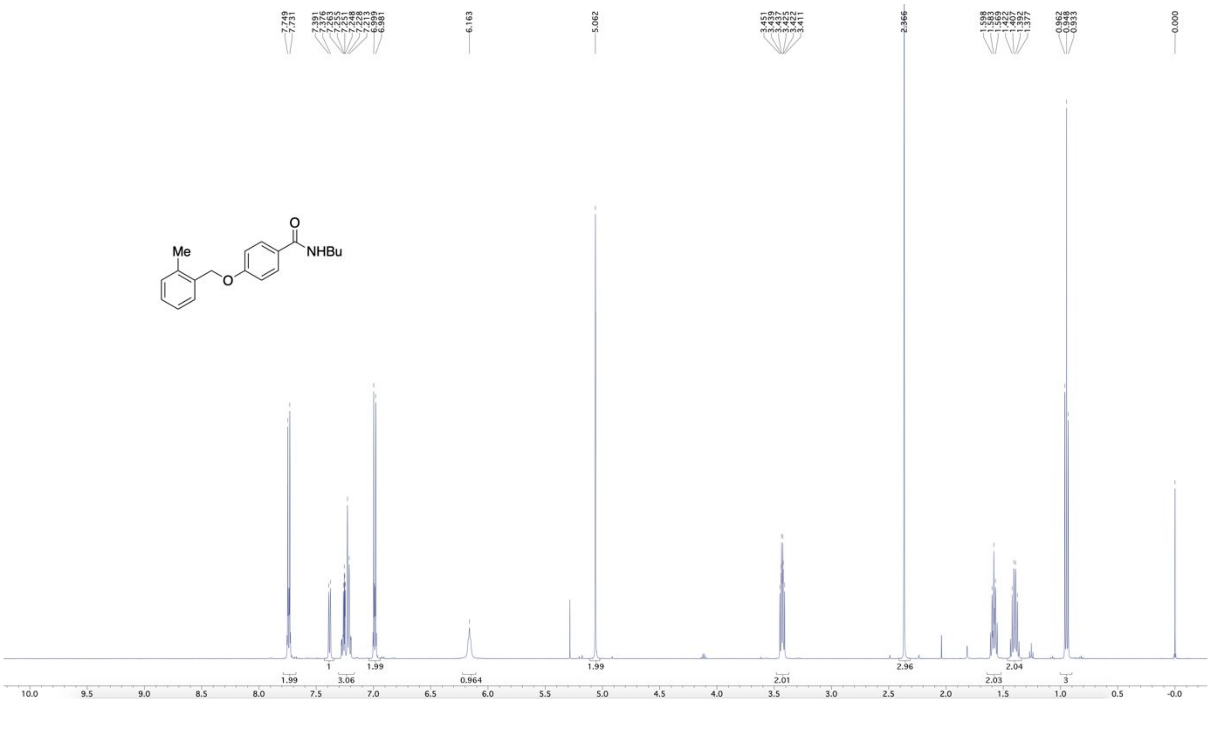

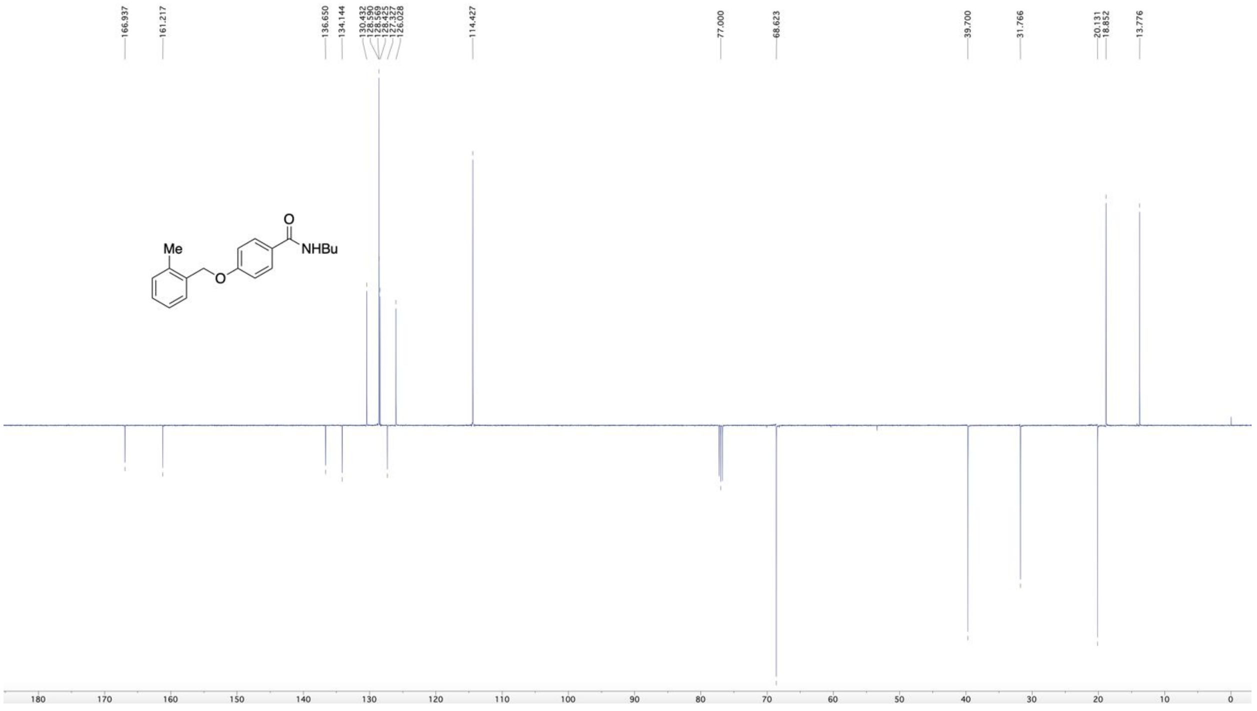

N-Butyl-4-((4-methoxybenzyl)oxy)benzamide **29d**

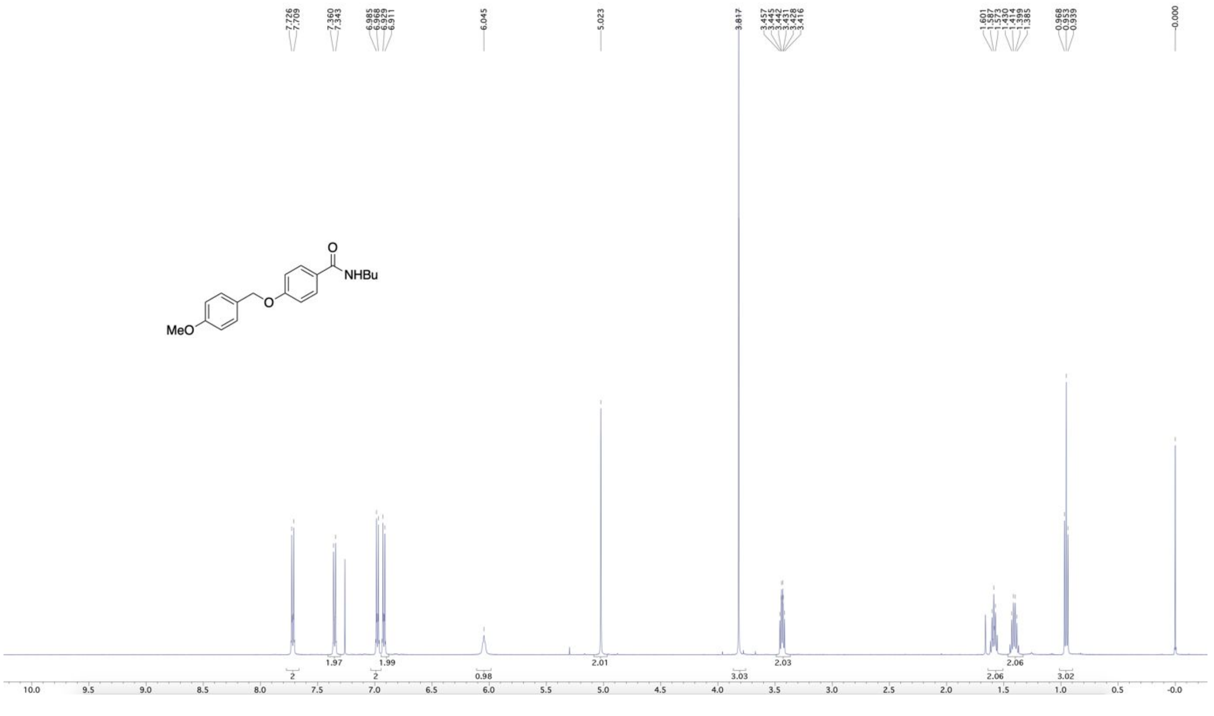

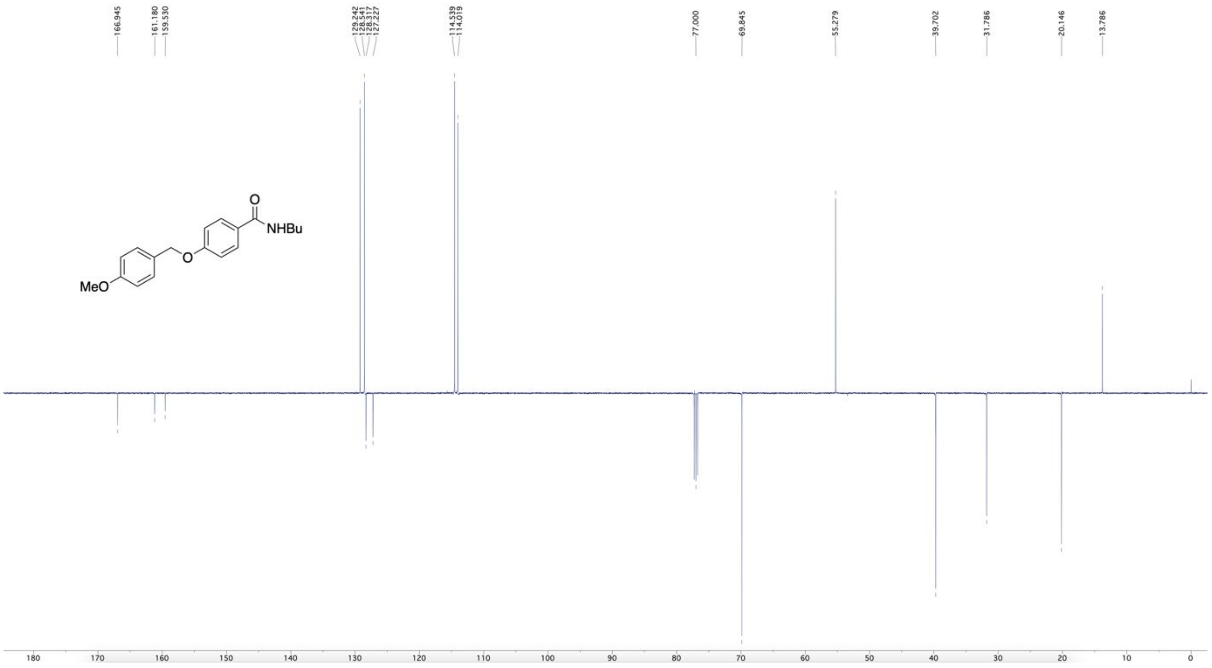

N-Butyl-4-((3-methoxybenzyl)oxy)benzamide **29e**

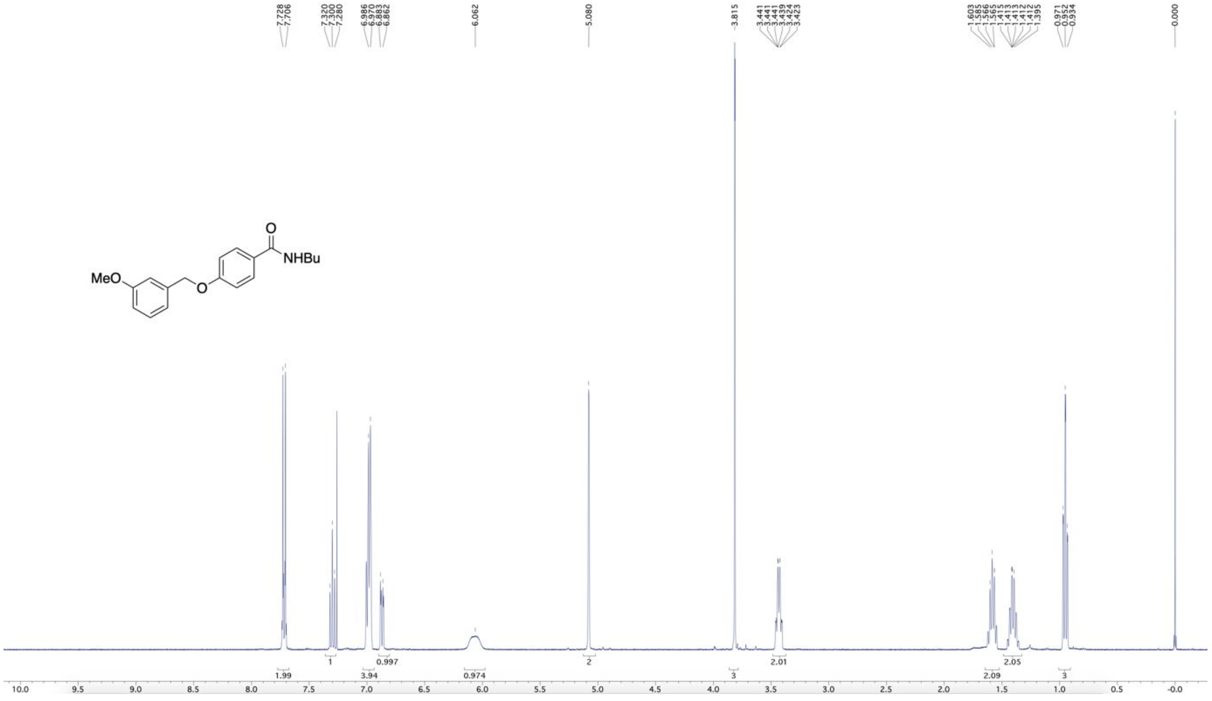

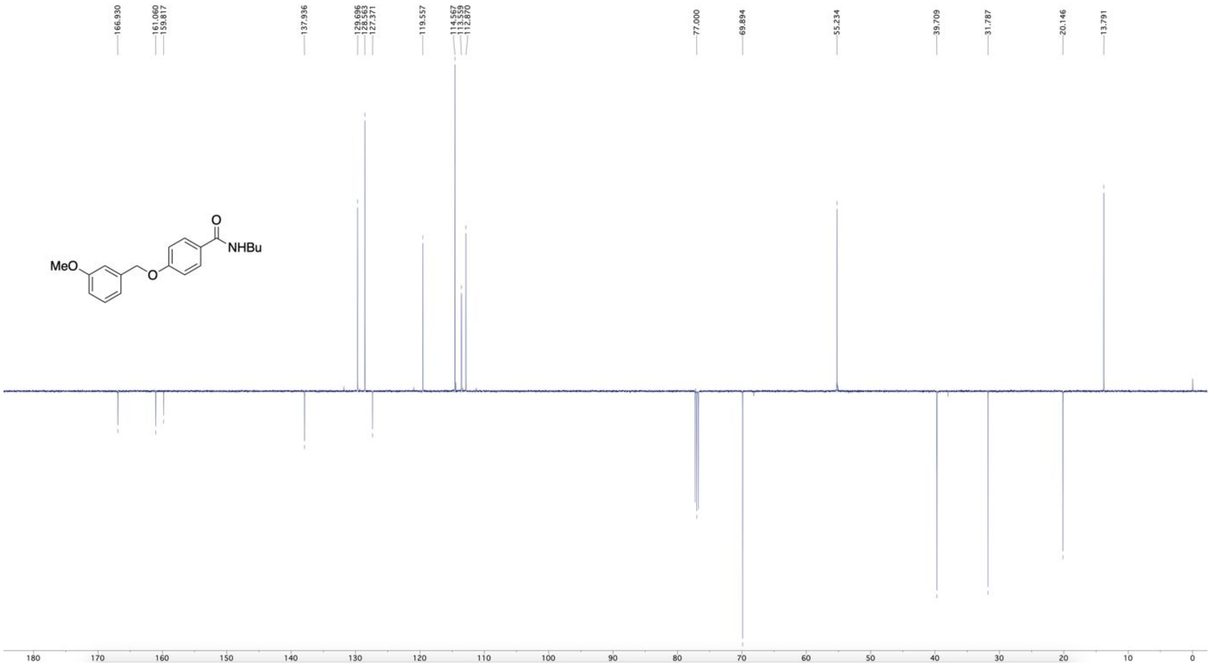

N-Butyl-4-((2-methoxybenzyl)oxy)benzamide **29f**

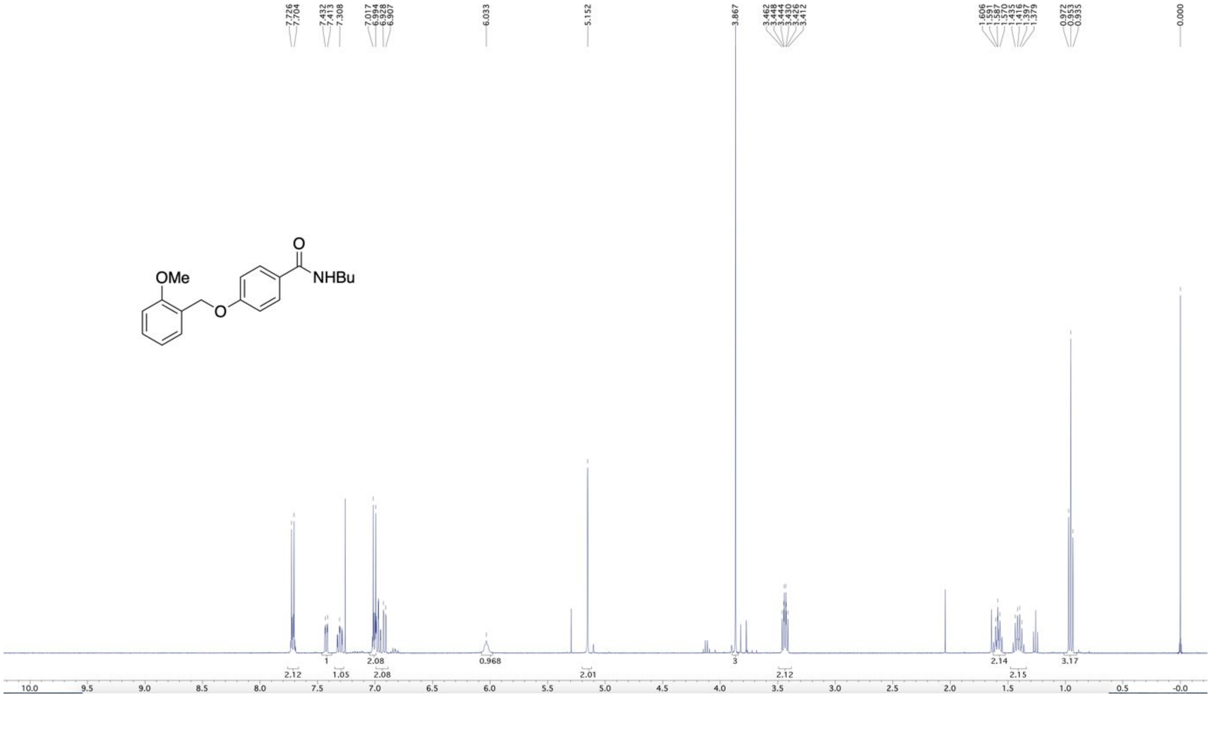

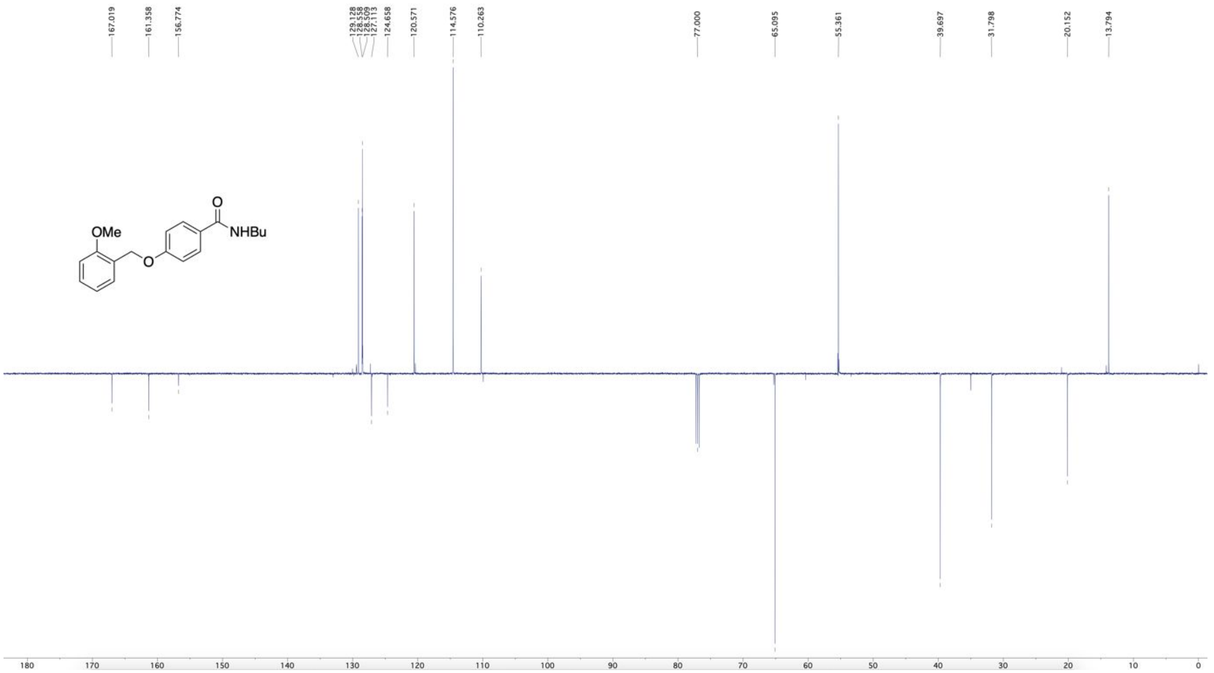

N-Butyl-4-((4-fluorobenzyl)oxy)benzamide **29g**

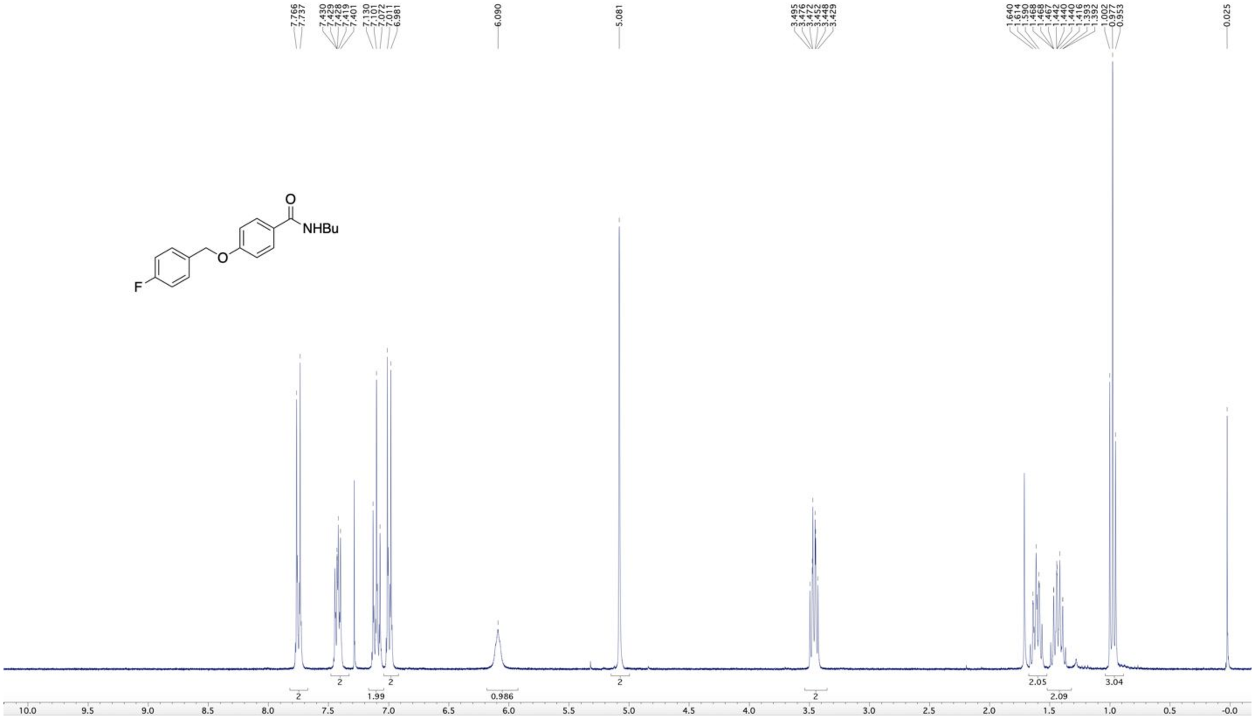

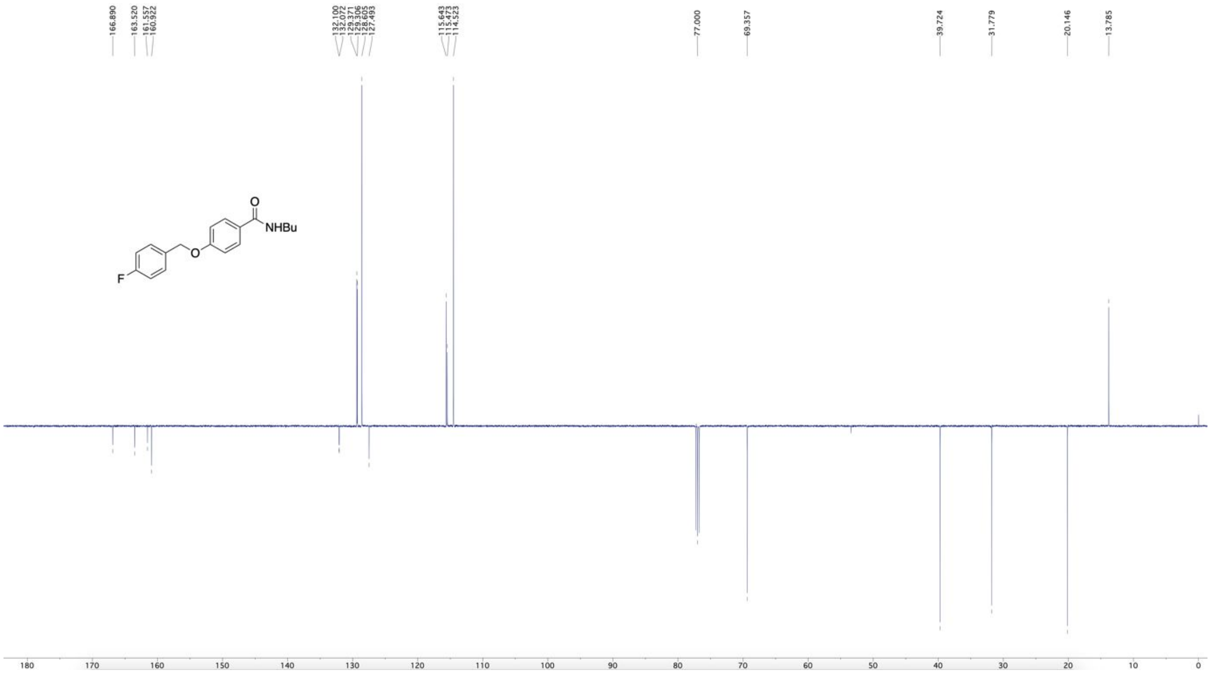

166.890
163.520
161.557
160.922
132.100
132.072
129.371
129.306
128.605
127.493
115.643
115.473
114.523
77.000
69.357
39.724
31.779
20.146
13.785
NHBu
O
F
180
170
160
150
140
130
120
110
100
90
80
70
60
50
40
30
20
10
0

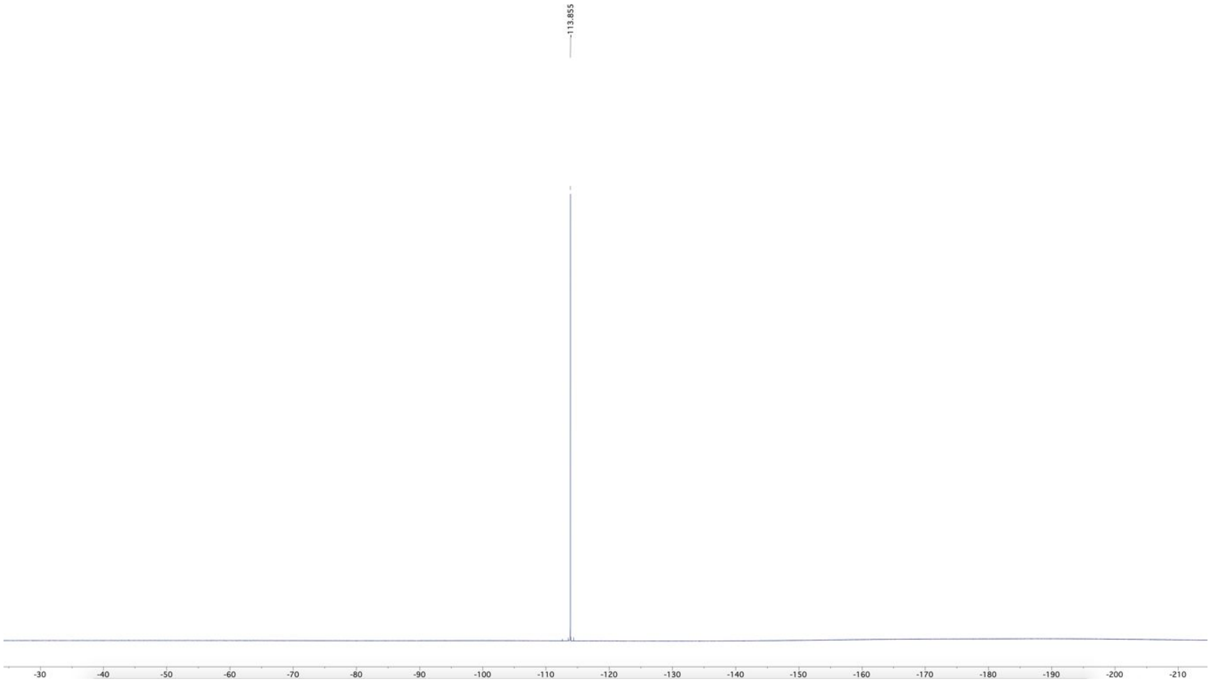

-113.855
-30
-40
-50
-60
-70
-80
-90
-100
-110
-120
-130
-140
-150
-160
-170
-180
-190
-200
-210

N-Butyl-4-((2-fluorobenzyl)oxy)benzamide **29h**

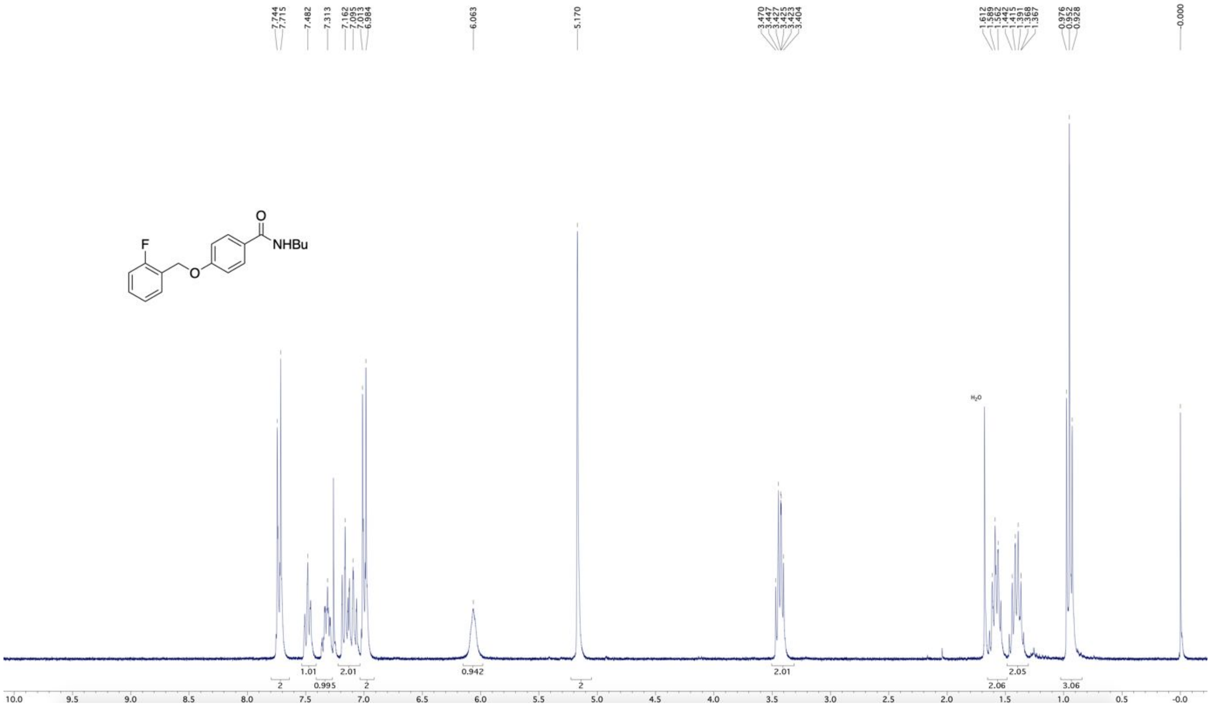

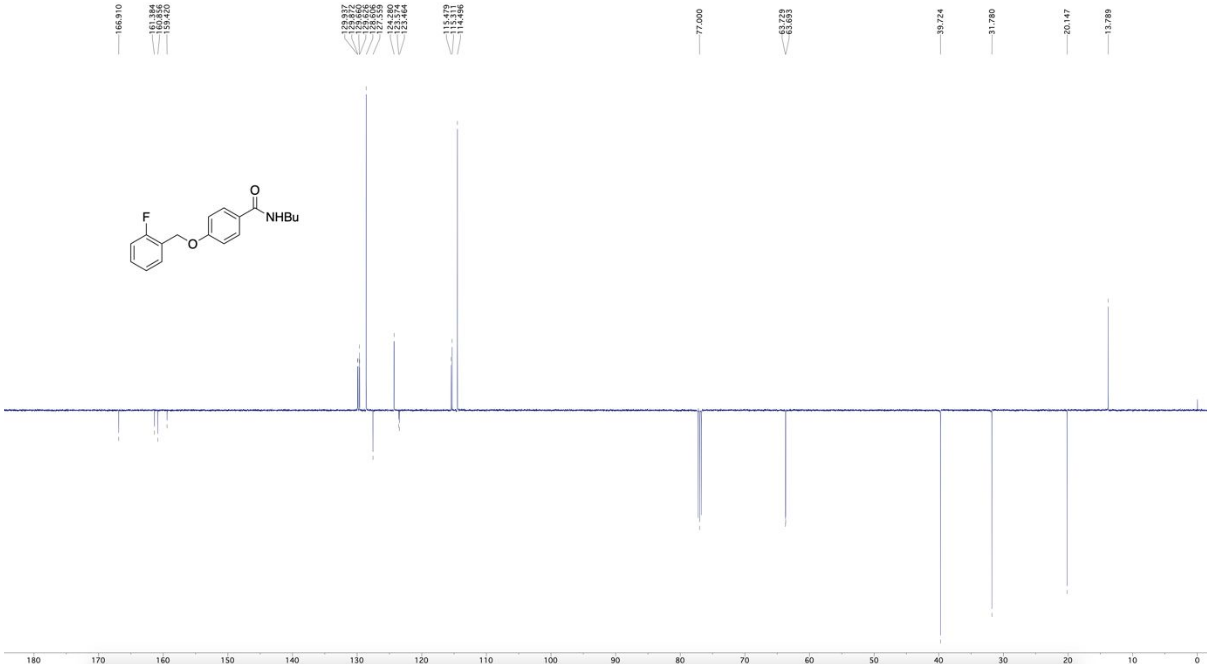

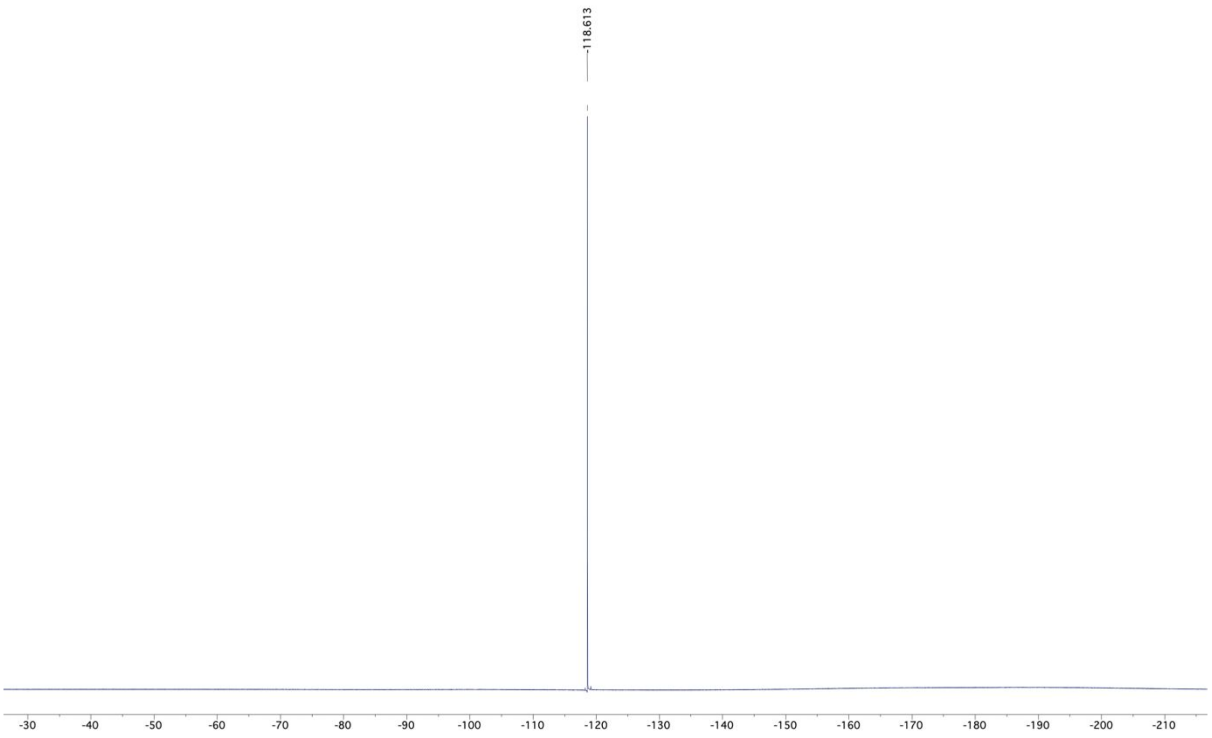

N-Butyl-4-((2-methoxynaphthalen-1-yl)methoxy)benzamide **29i**

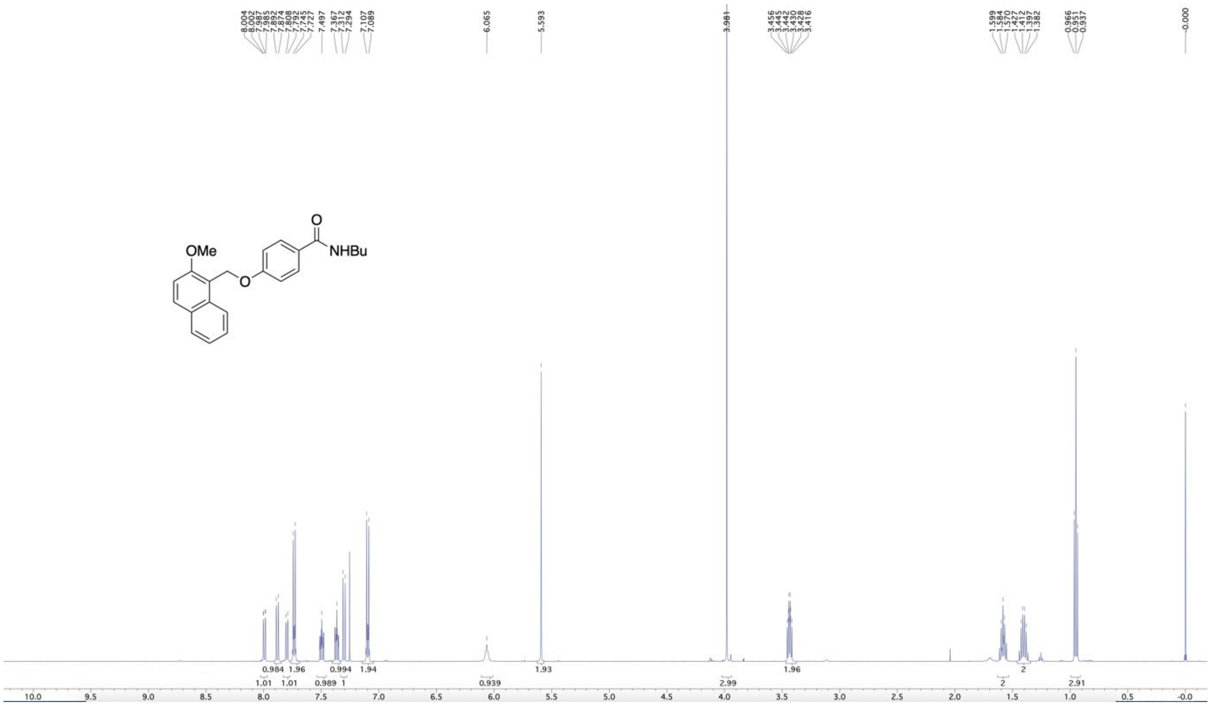

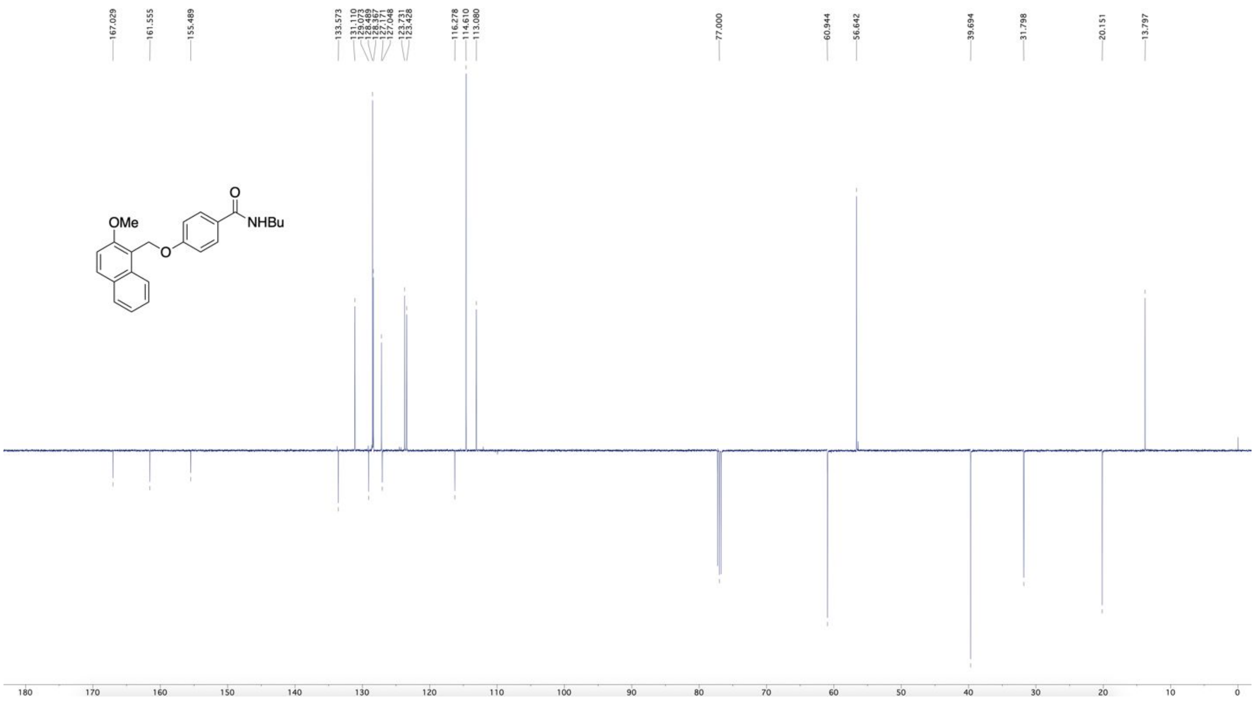

N-Butyl-4-((4-nitrobenzyl)oxy)benzamide **29j**

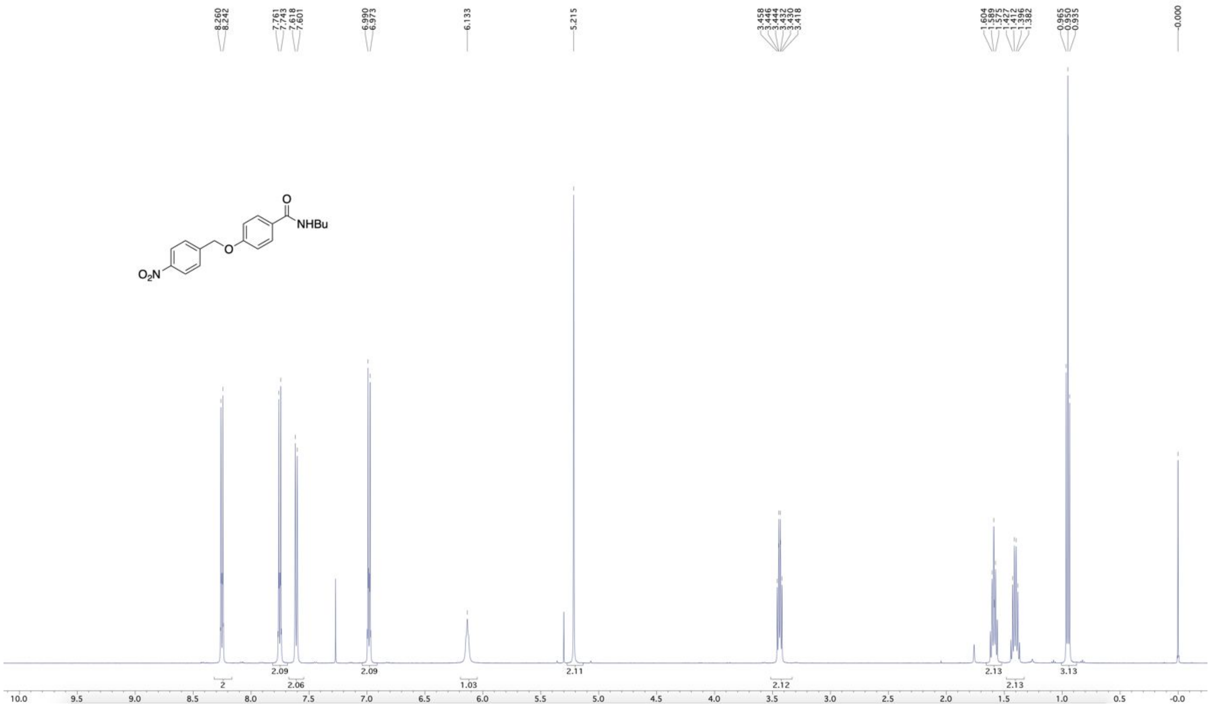

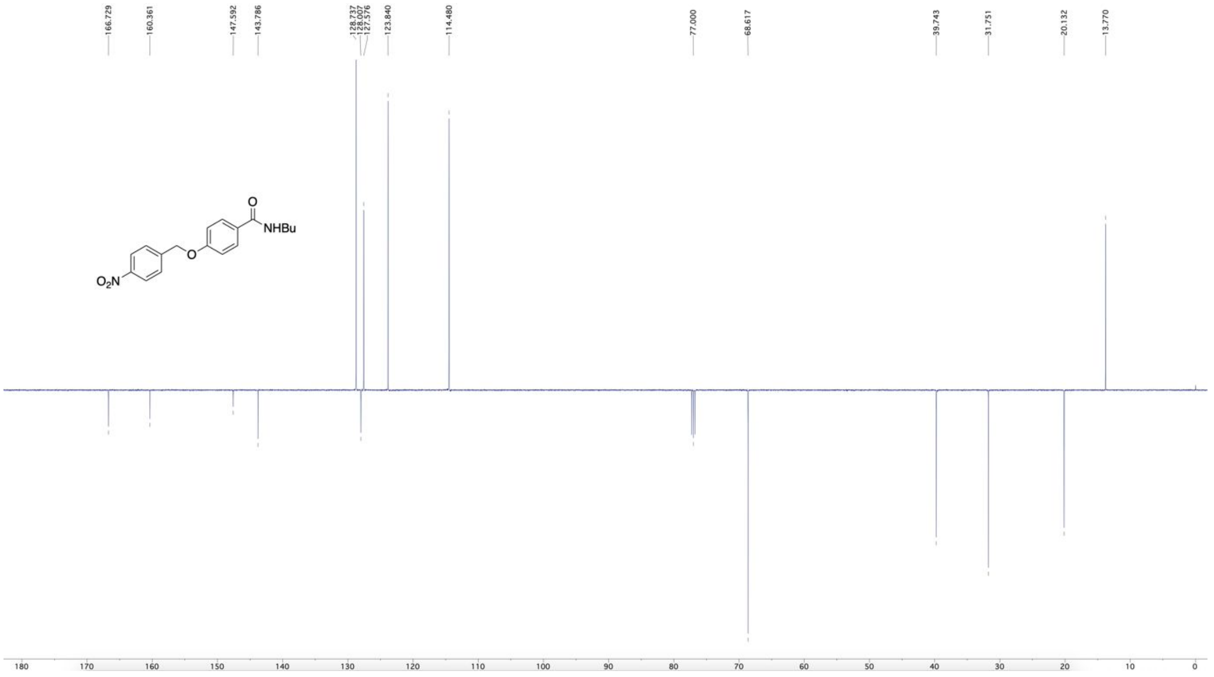

N-Butyl-4-((3-nitrobenzyl)oxy)benzamide **29k**

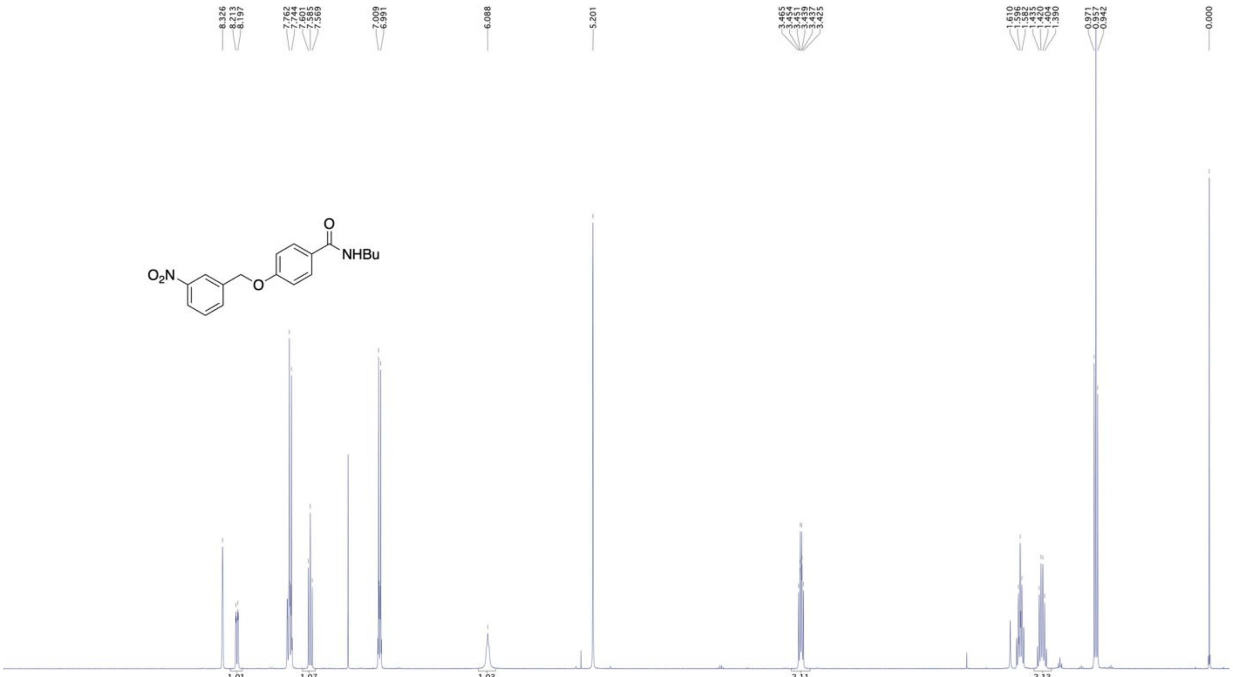

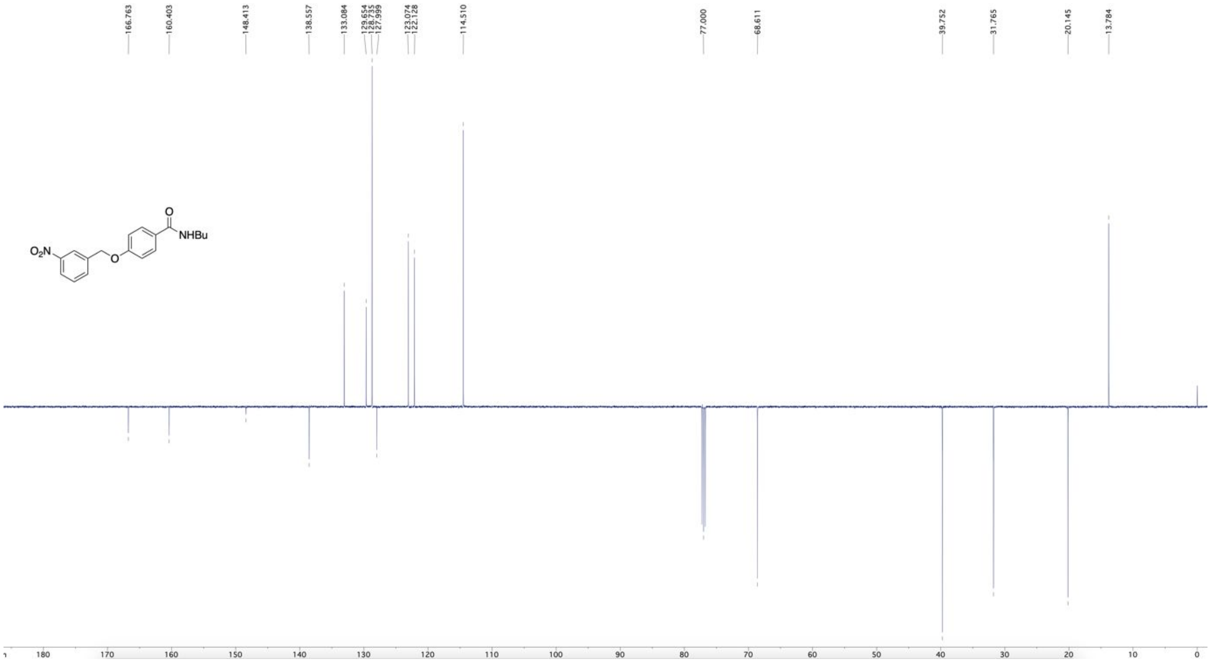

N-Butyl-4-((2-nitrobenzyl)oxy)benzamide **29l**

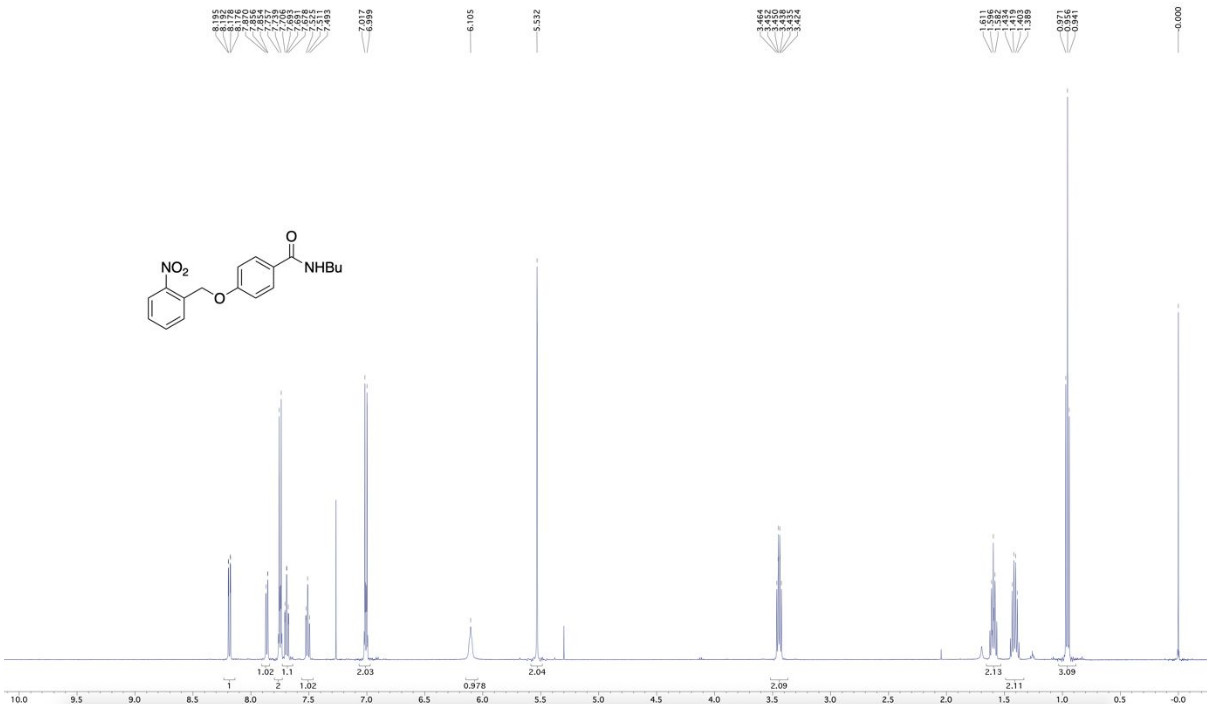

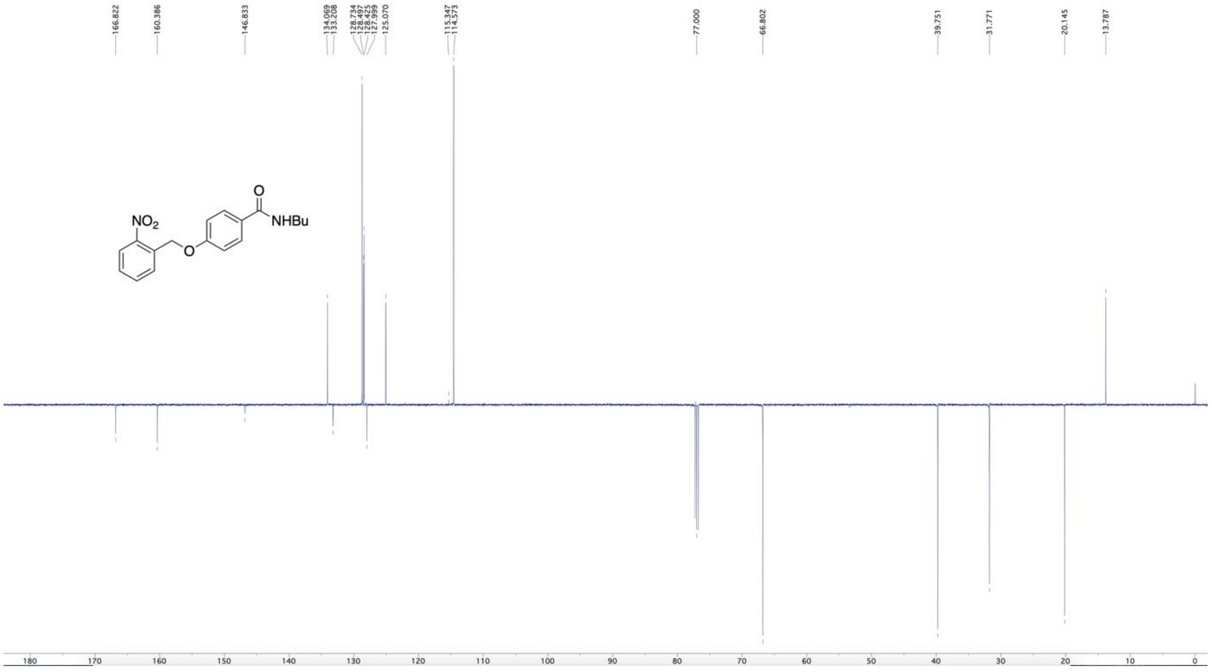

N-Butyl-4-((perfluorophenyl)methoxy)benzamide **29m**

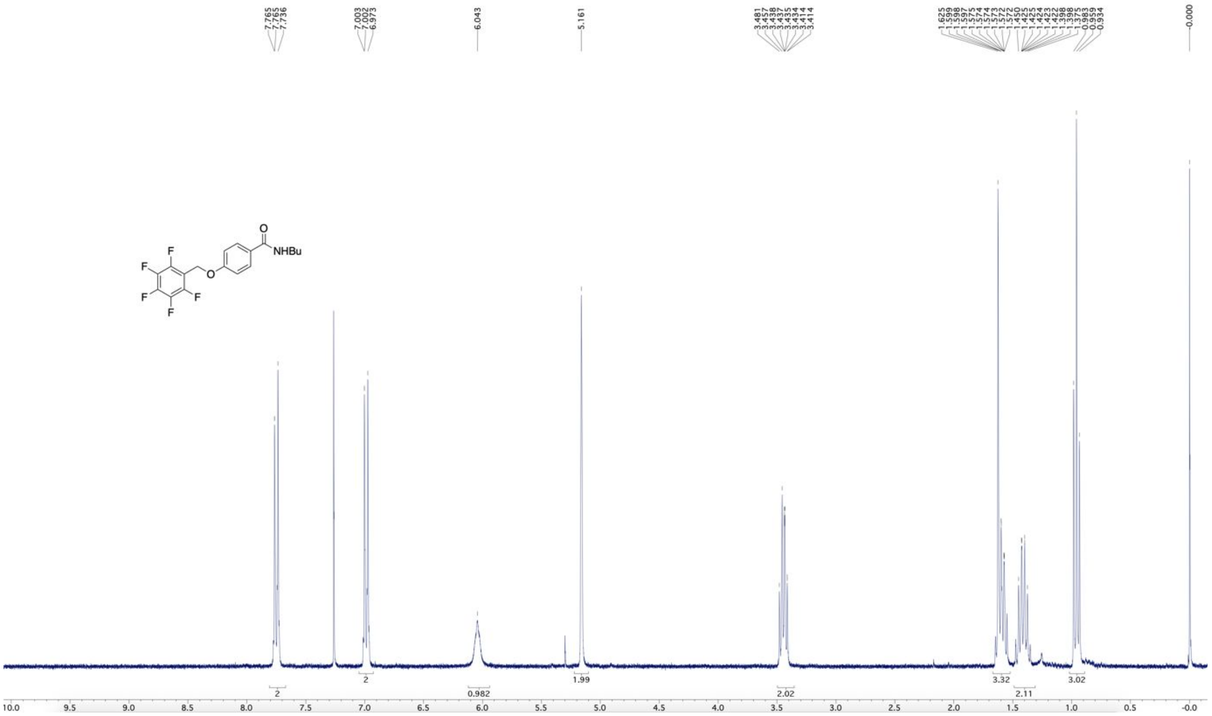

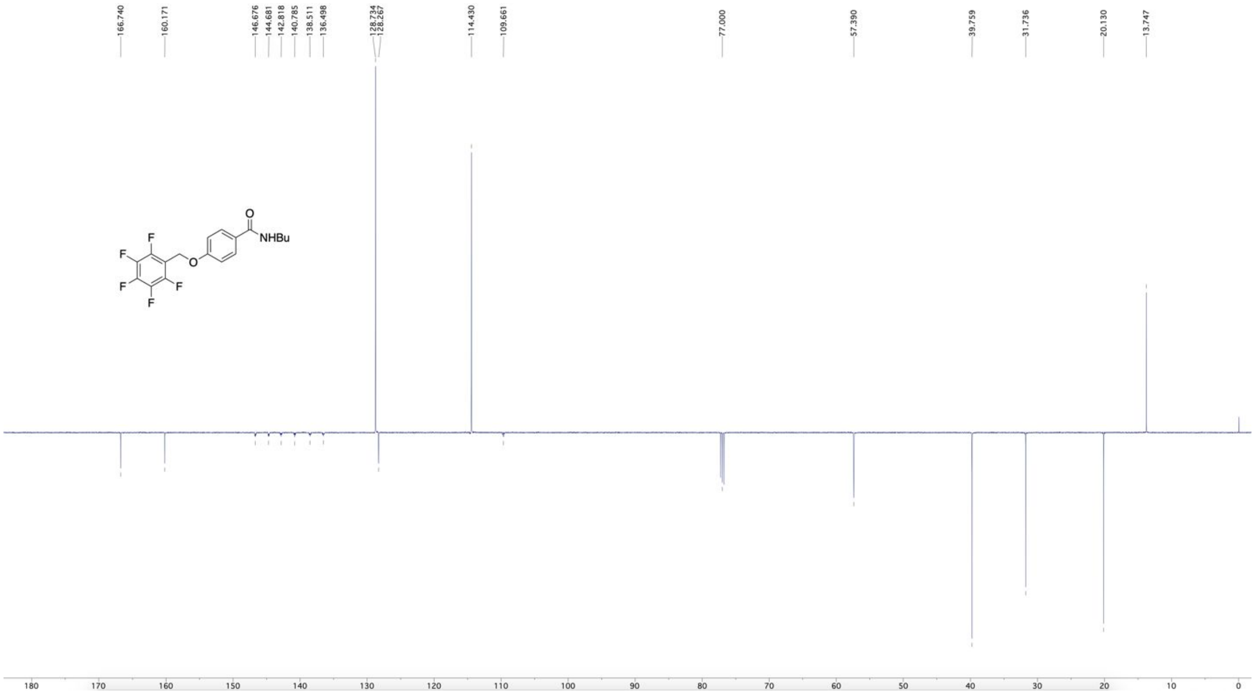

166.740
160.171
146.676
144.681
142.818
140.785
138.511
136.498
128.734
128.267
114.430
109.661
77.000
57.390
39.759
31.736
20.130
13.747
NHBu

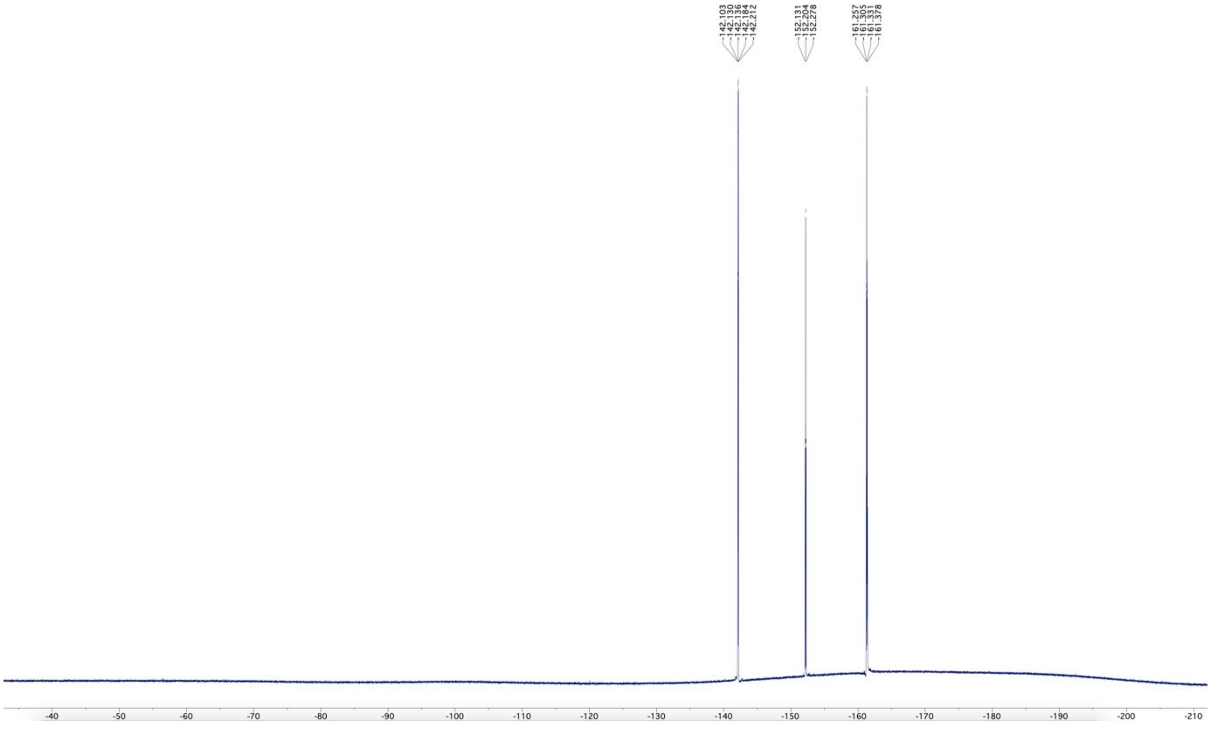

-142.103
-142.130
-142.136
-142.184
-142.212
-152.131
-152.204
-152.278
-161.257
-161.305
-161.331
-161.378

4-(Diphenylmethoxy)-*N*-butylbenzamide **29n**

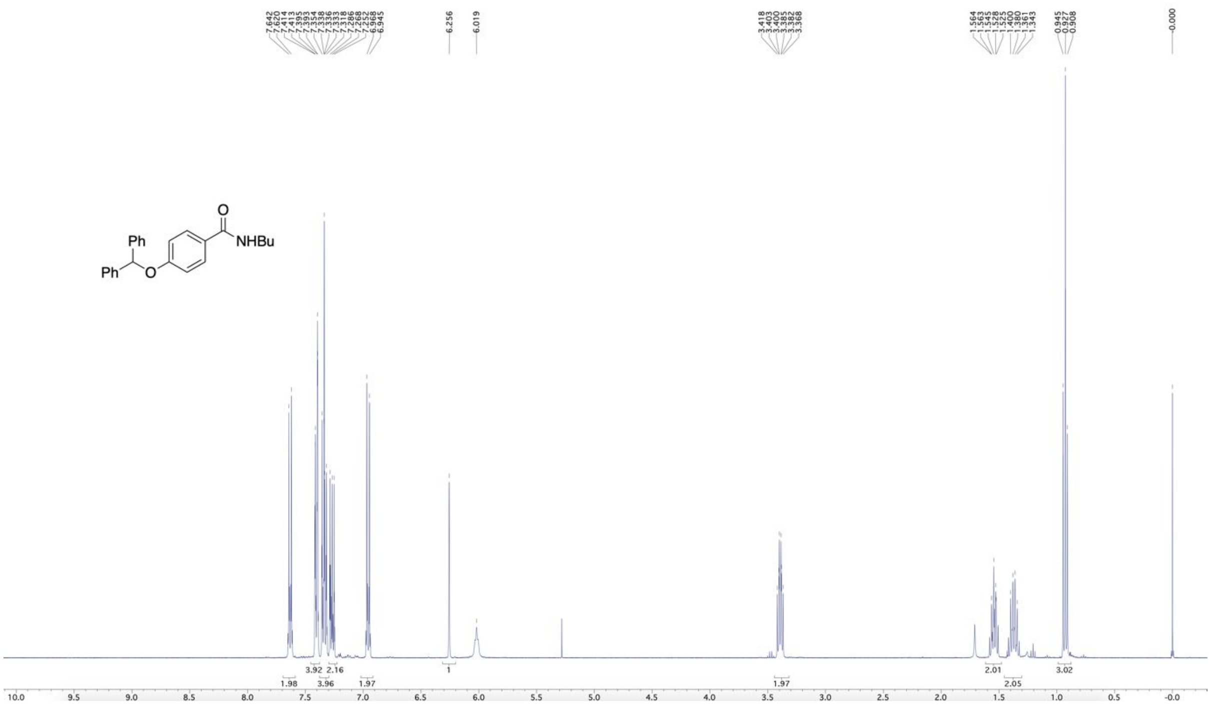

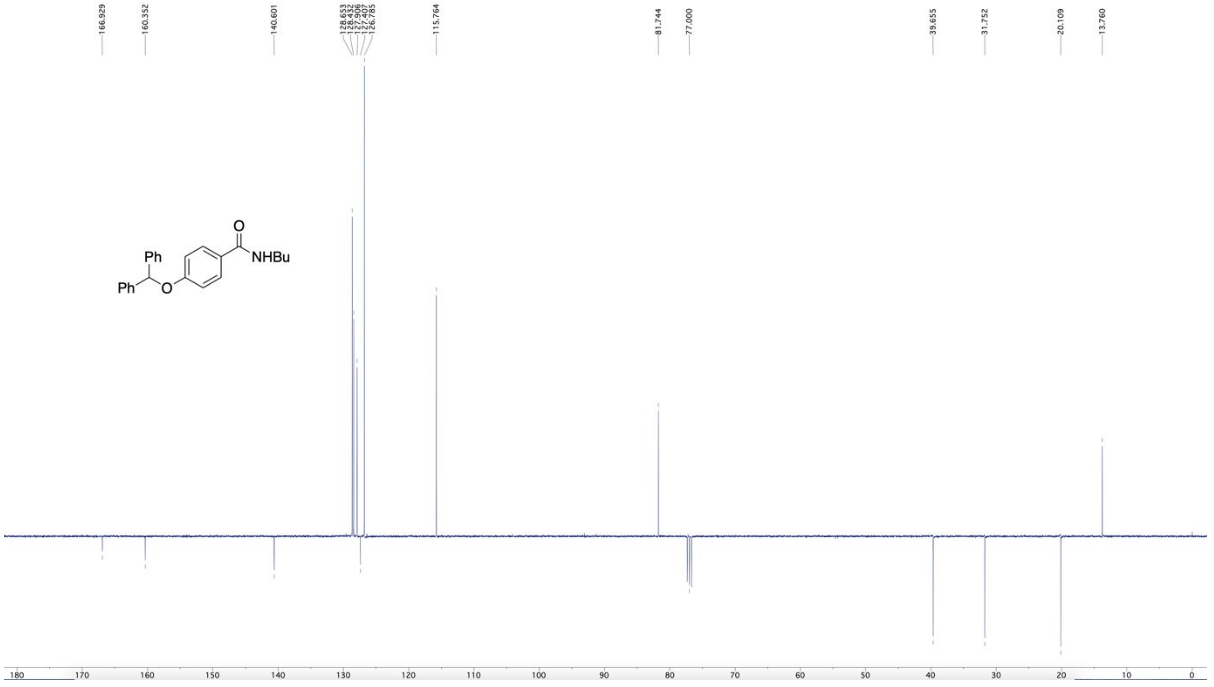

N-Butyl-4-(1-phenylethoxy)benzamide **29o**

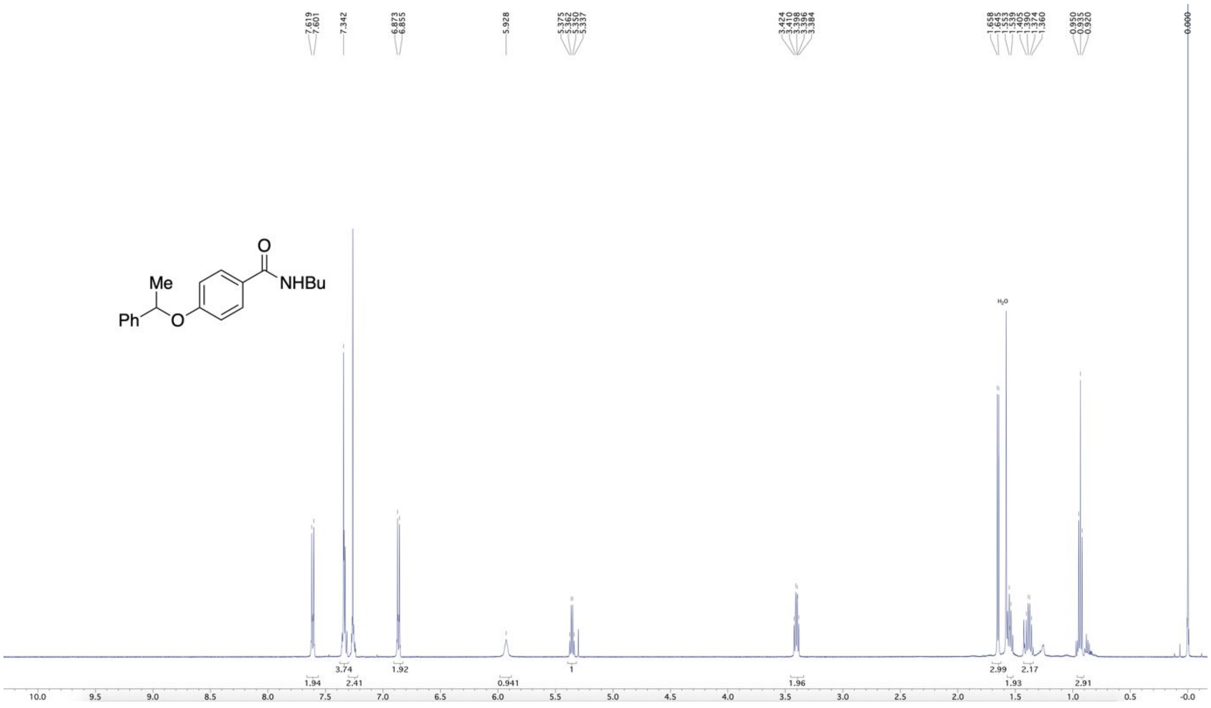

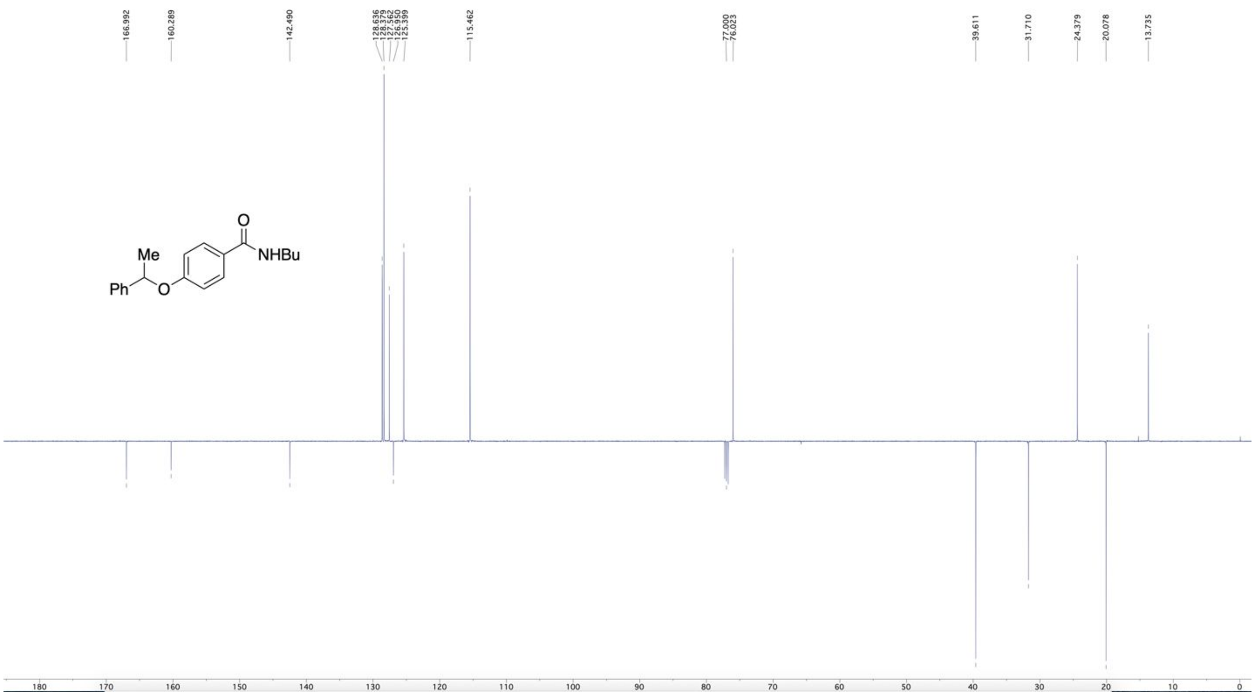

N-Butyl-4-((3-methylbut-2-en-1-yl)oxy)benzamide **29p**

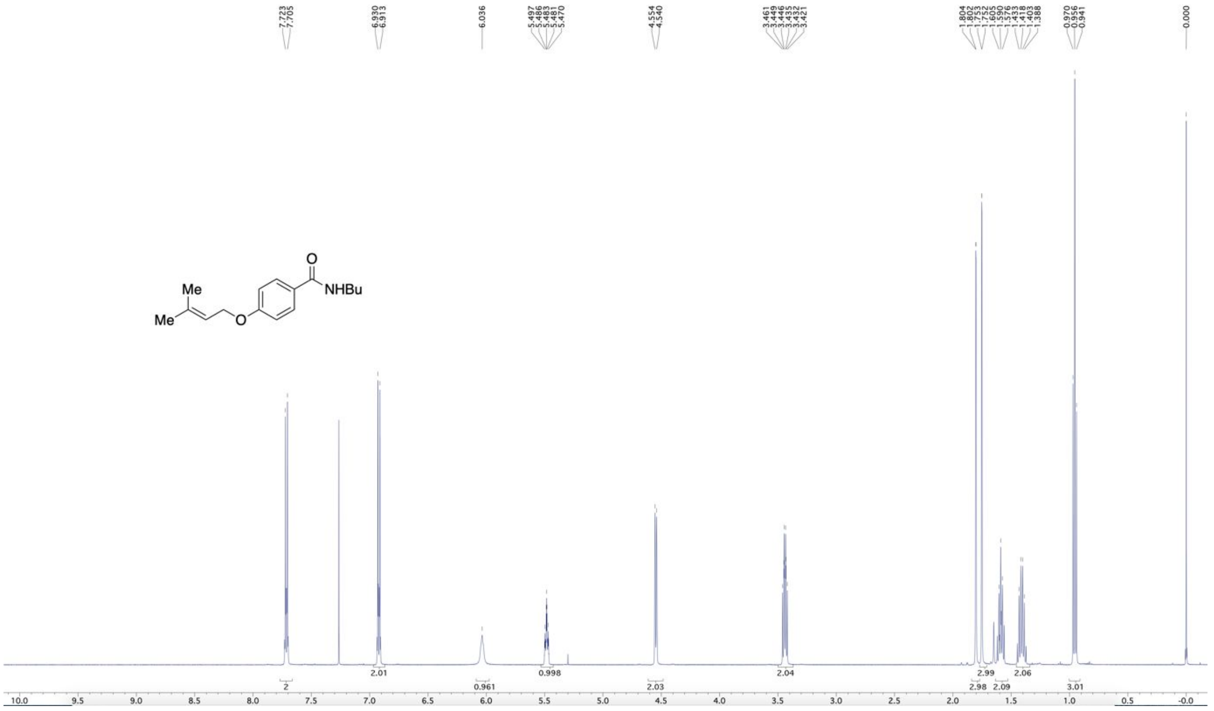

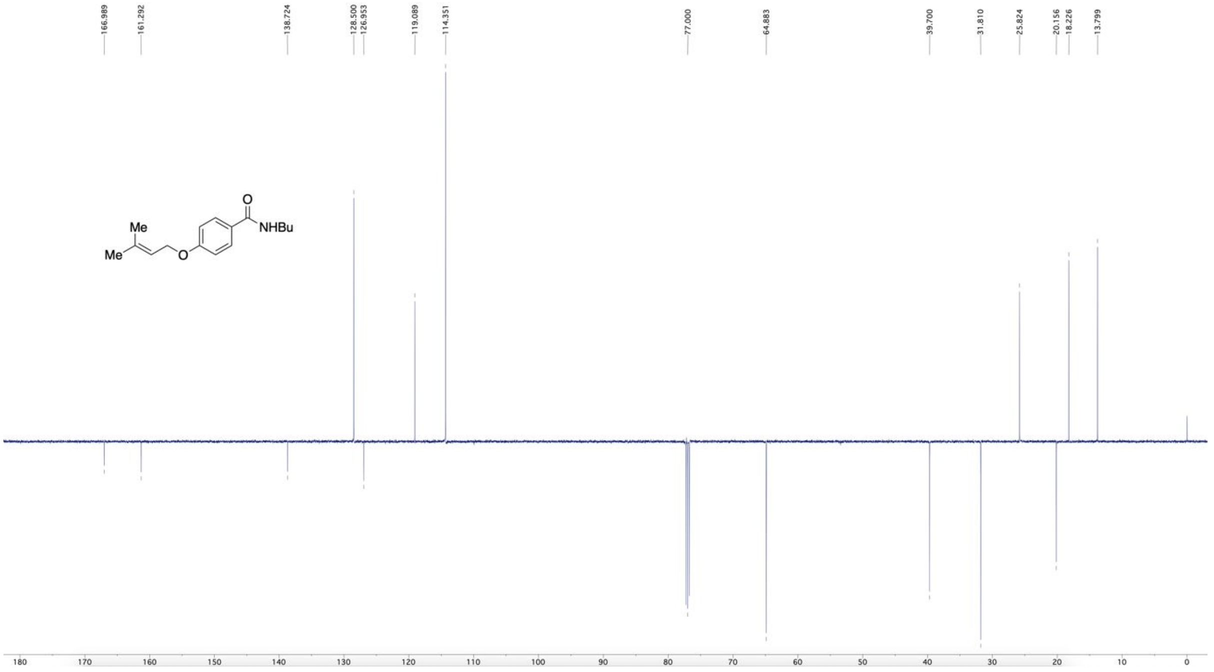

N-Butyl-4-(hydroxy(phenyl)methyl)benzamide **30a**

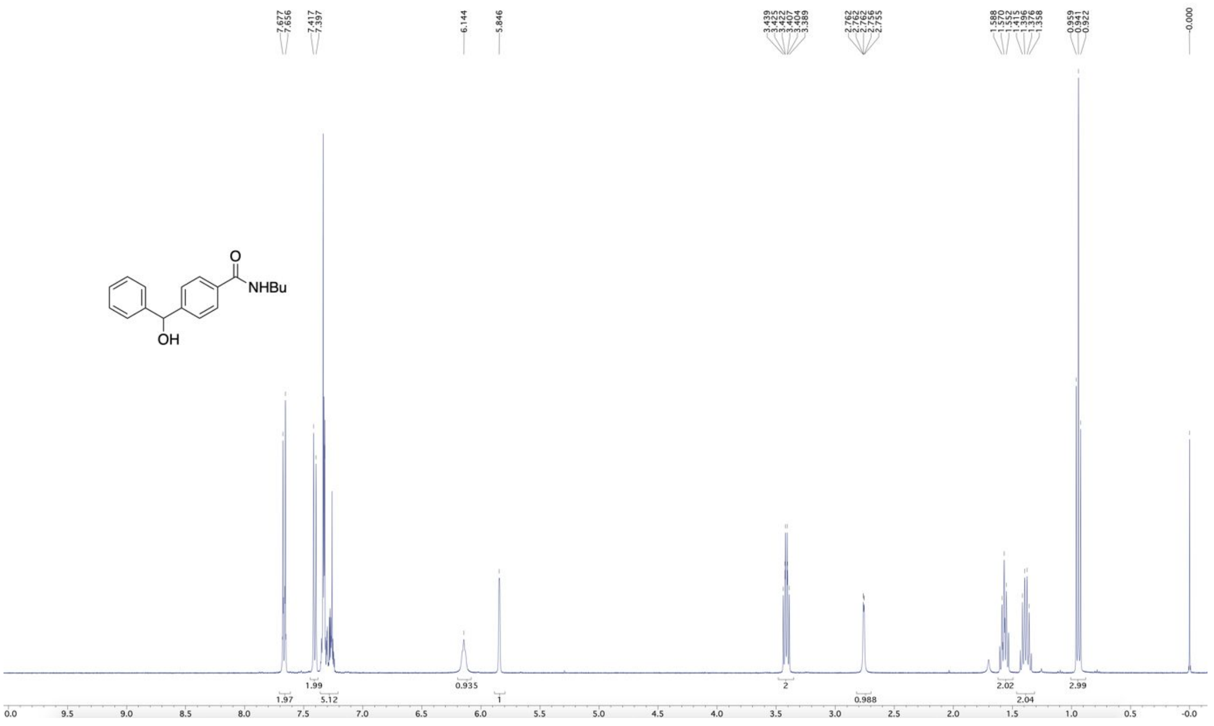

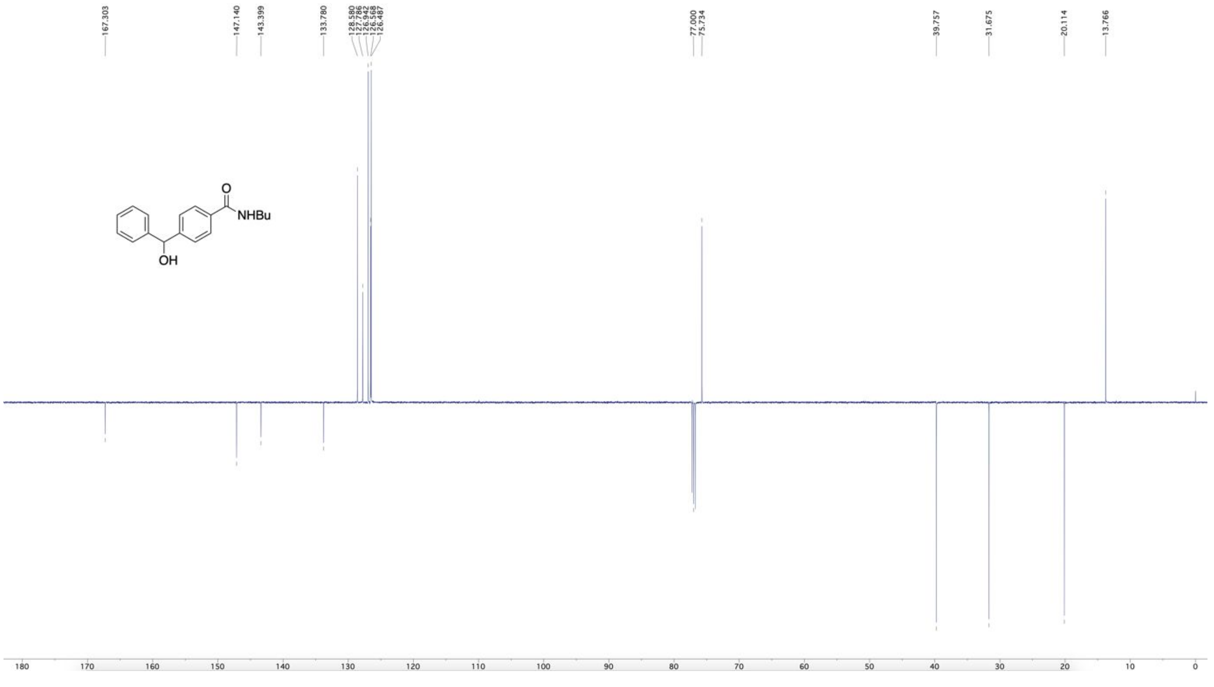

N-Butyl-4-(hydroxy(*p*-tolyl)methyl)benzamide **30b**

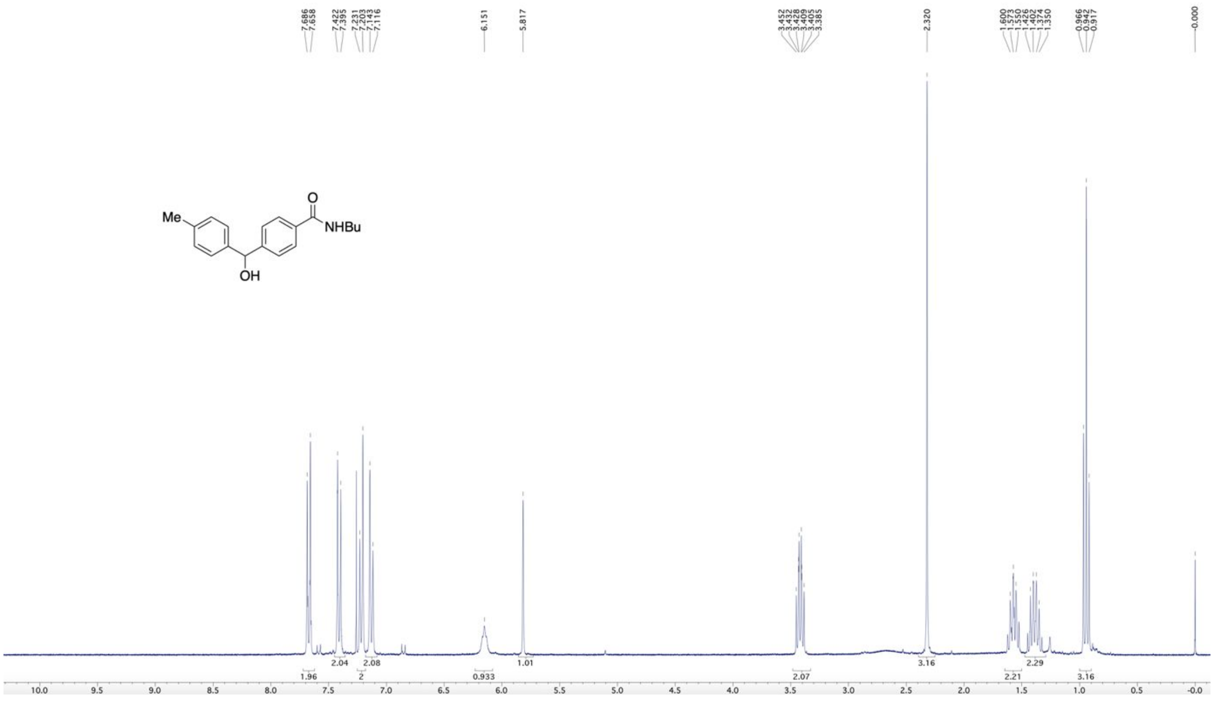

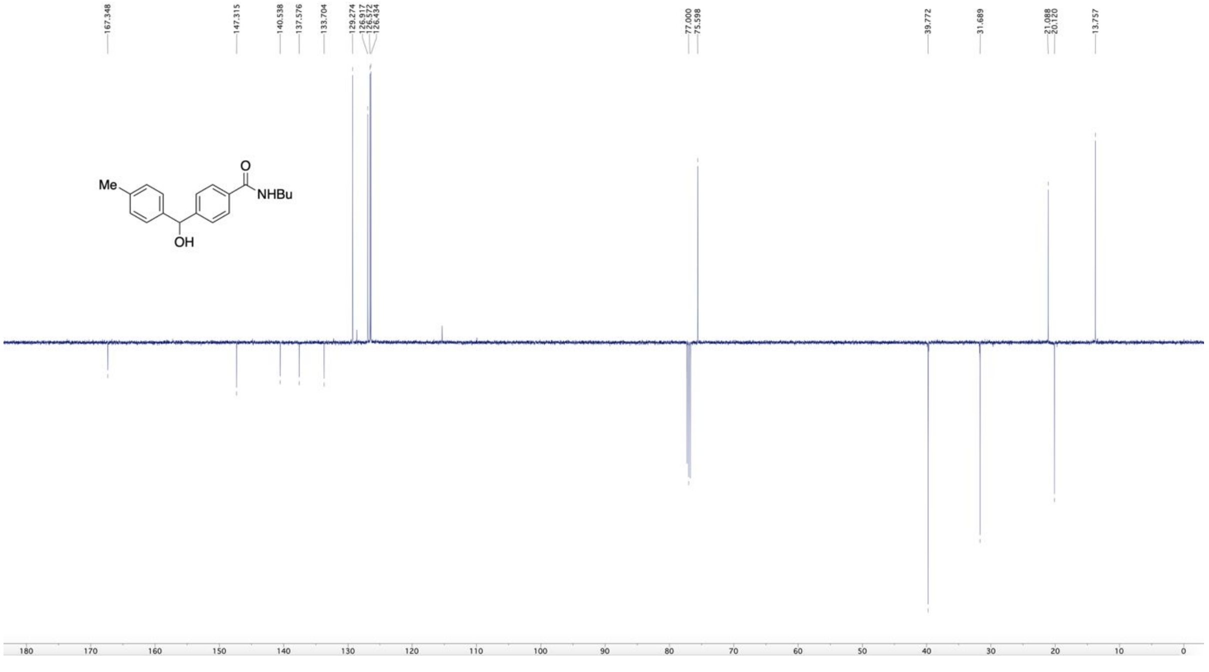

N-Butyl-4-(hydroxy(*o*-tolyl)methyl)benzamide **30c**

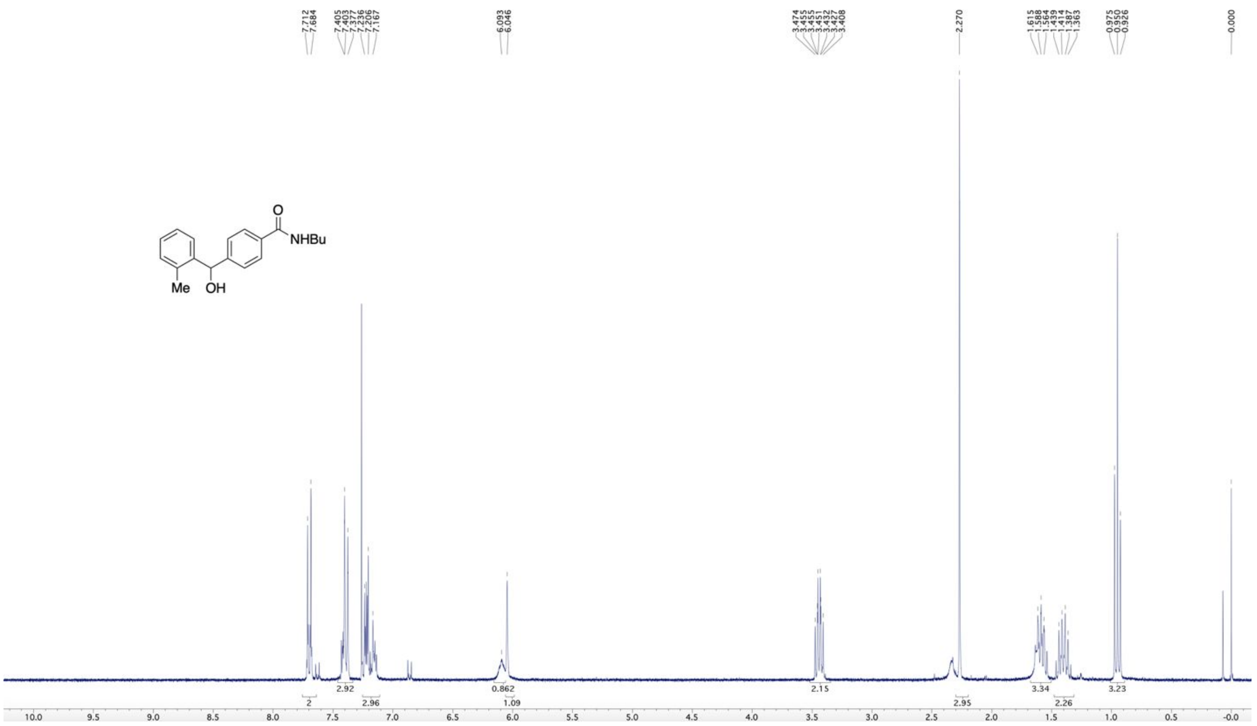

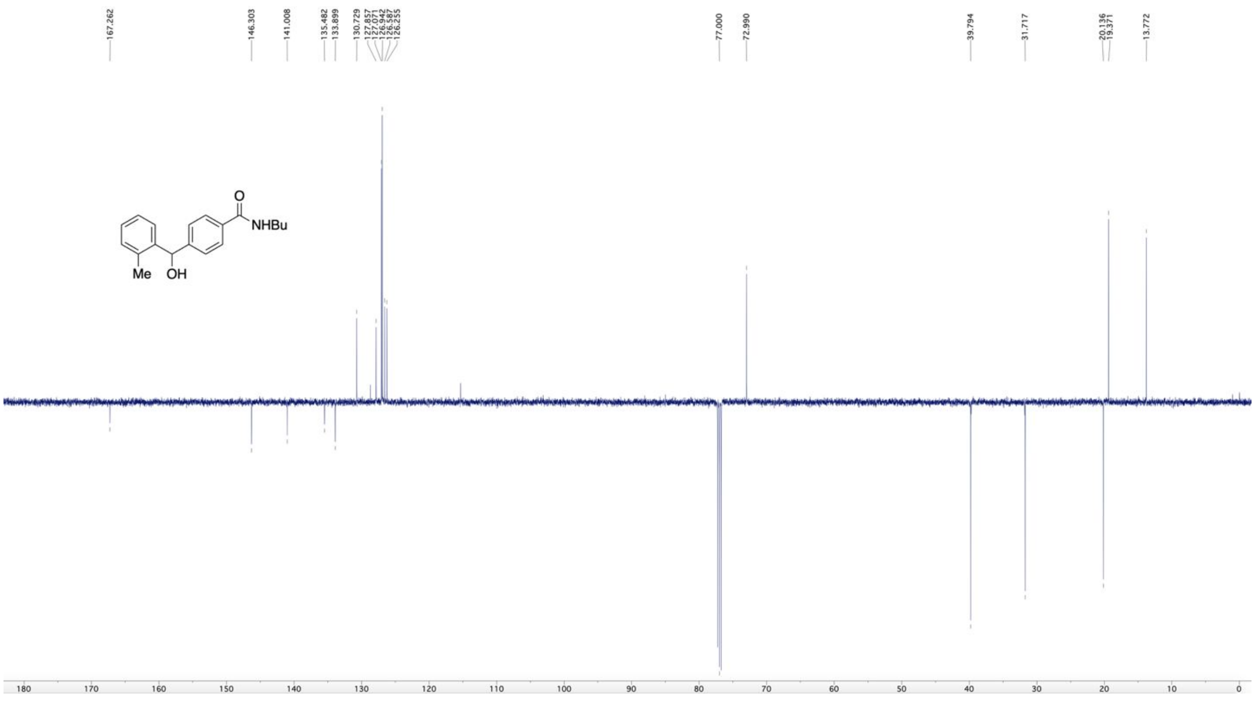

N-Butyl-4-(hydroxy(4-methoxyphenyl)methyl)benzamide **30d**

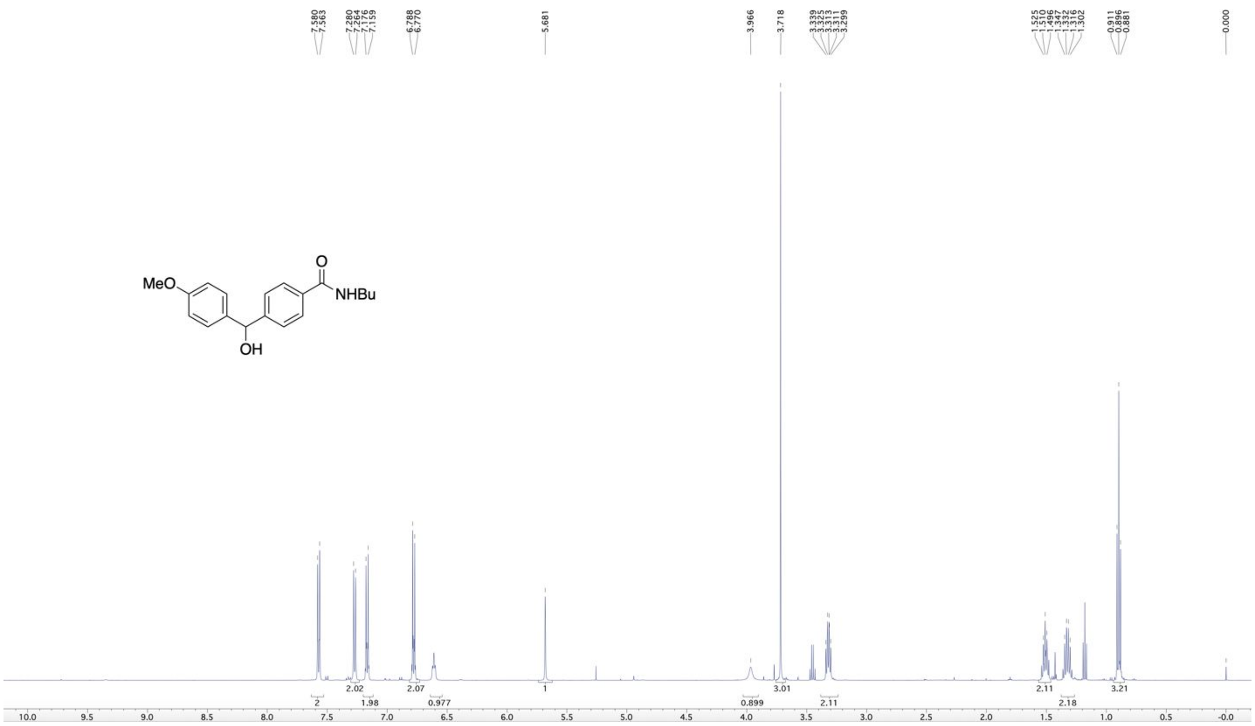

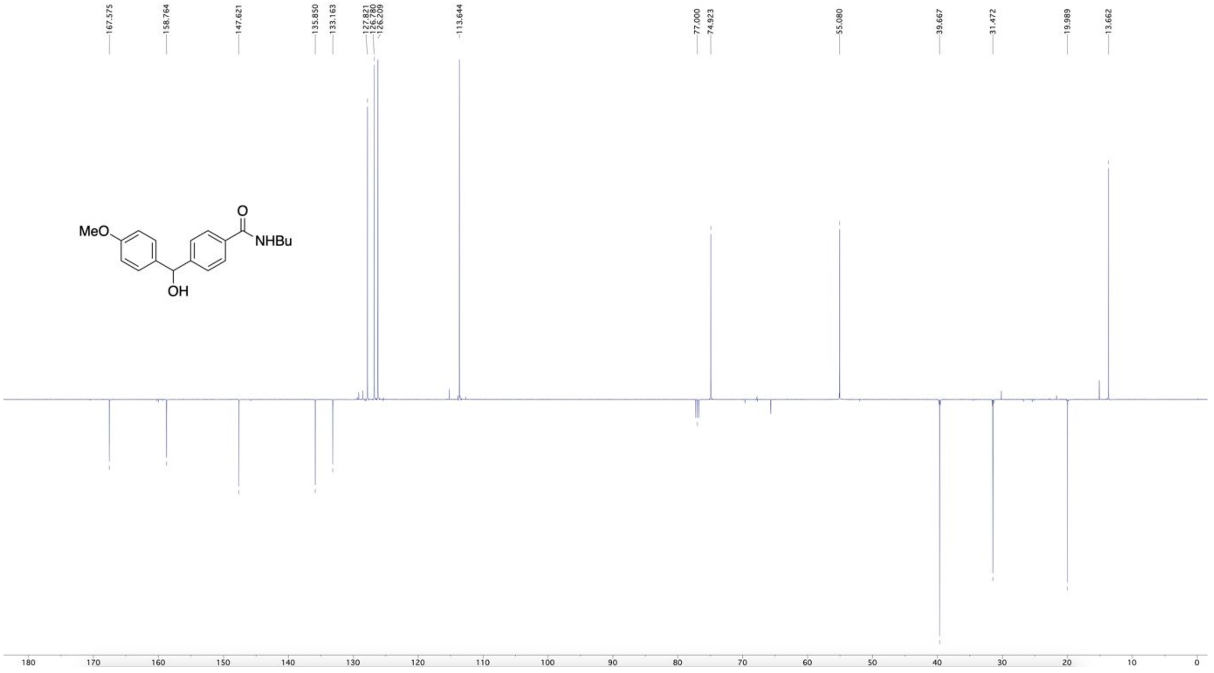

N-Butyl-4-(hydroxy(3-methoxyphenyl)methyl)benzamide **30e**

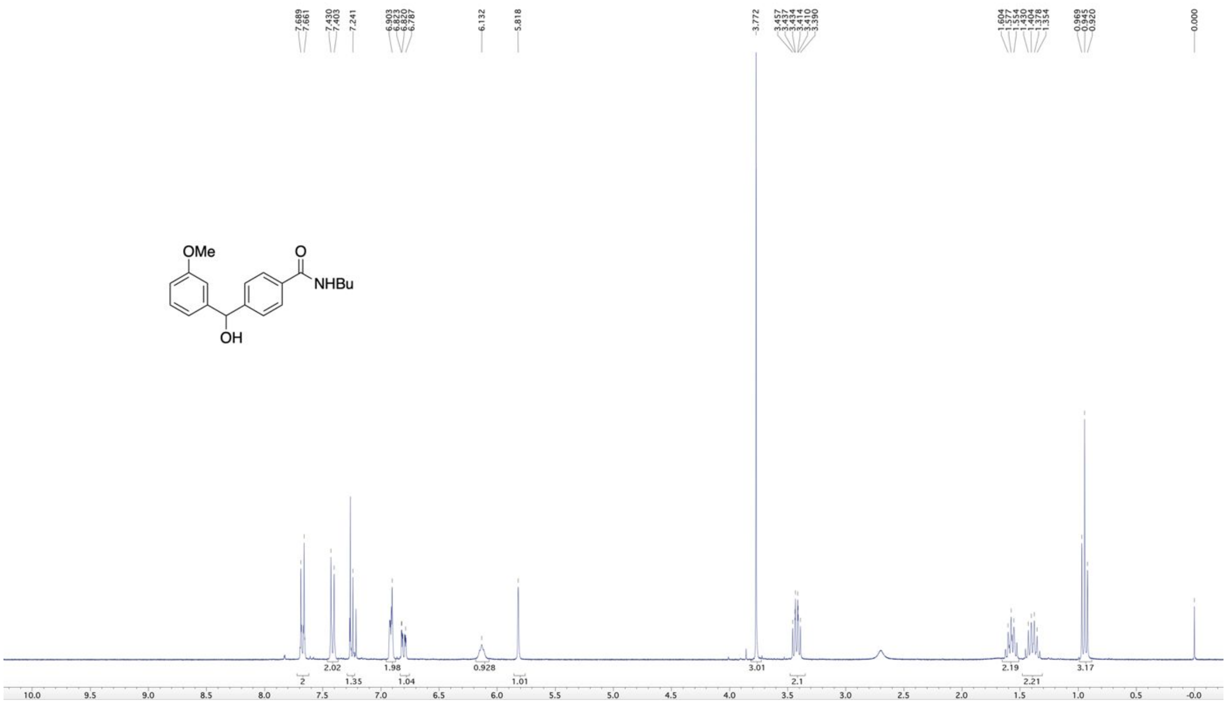

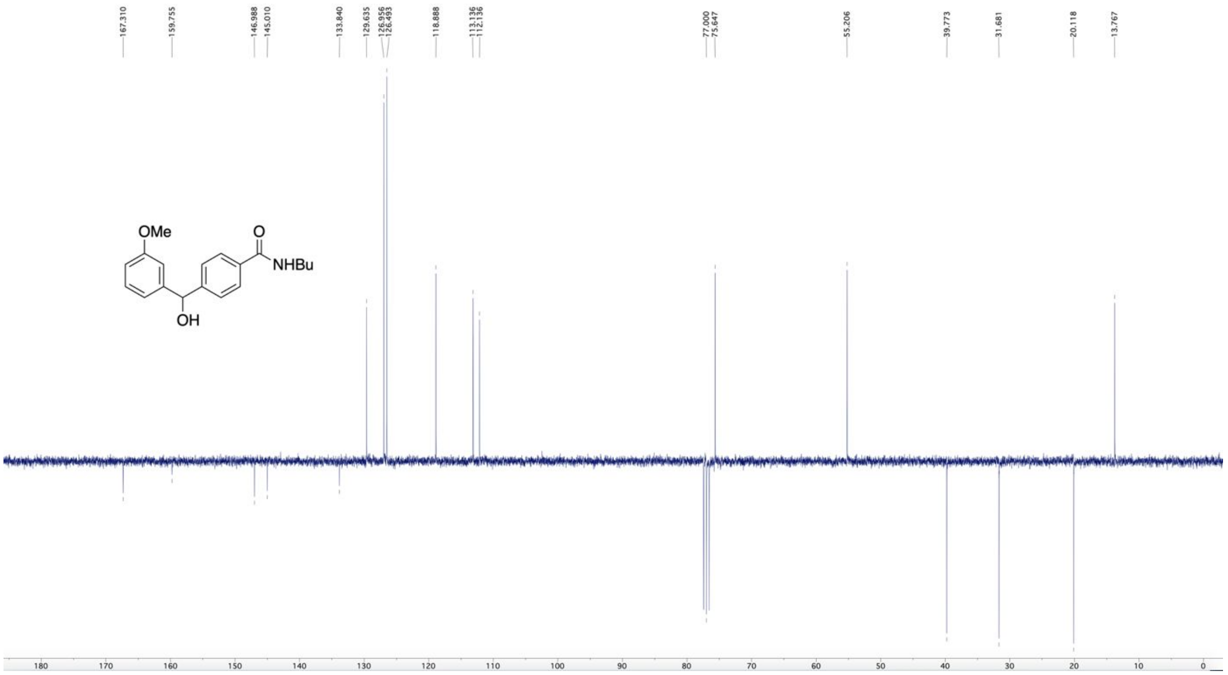

N-Butyl-4-(hydroxy(2-methoxyphenyl)methyl)benzamide **30f**

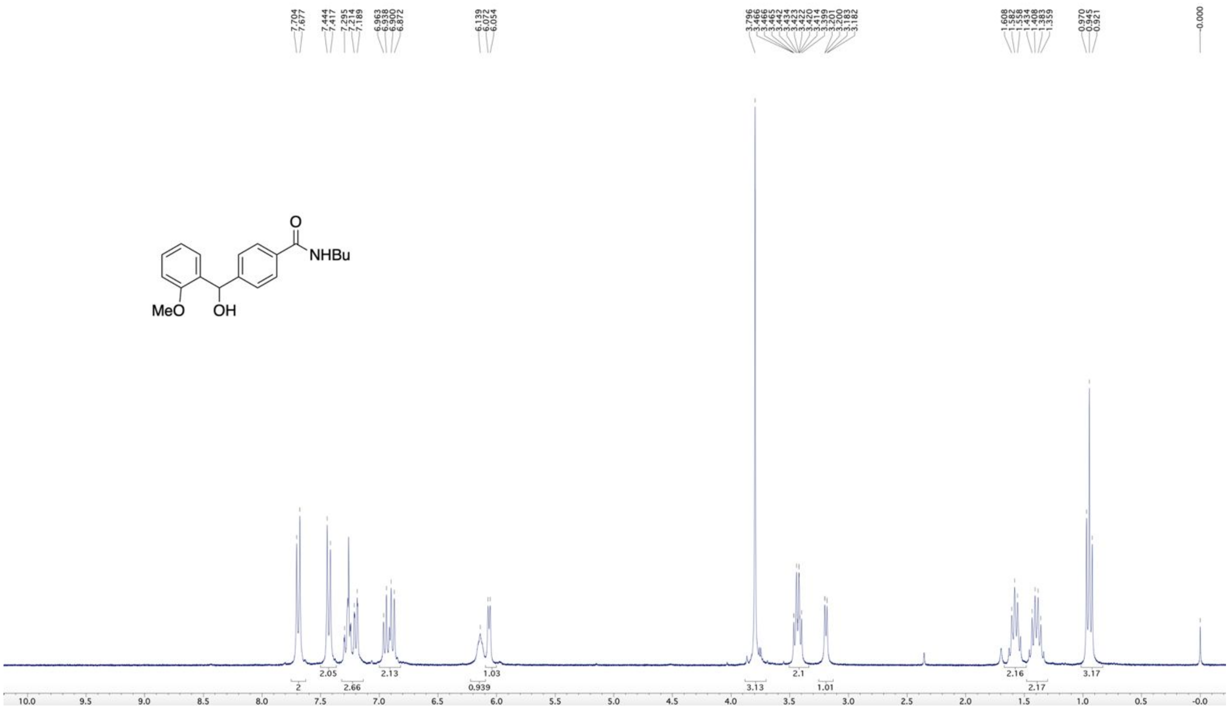

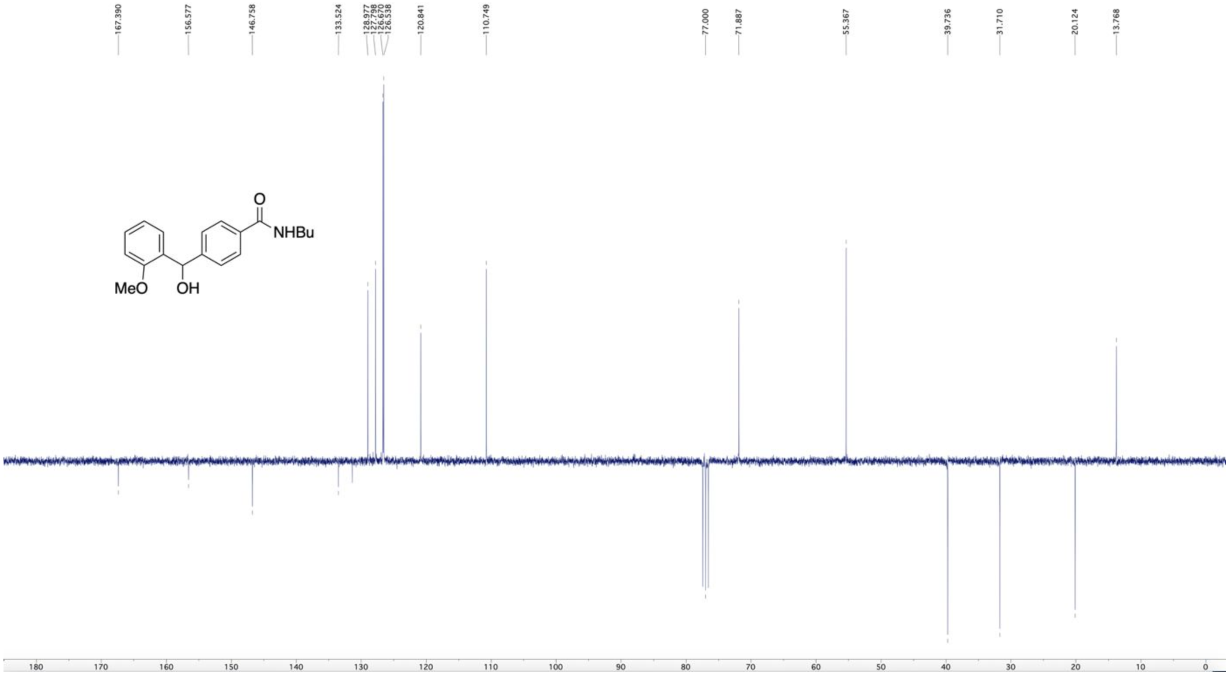

N-Butyl-4-((4-fluorophenyl)(hydroxy)methyl)benzamide **30g**

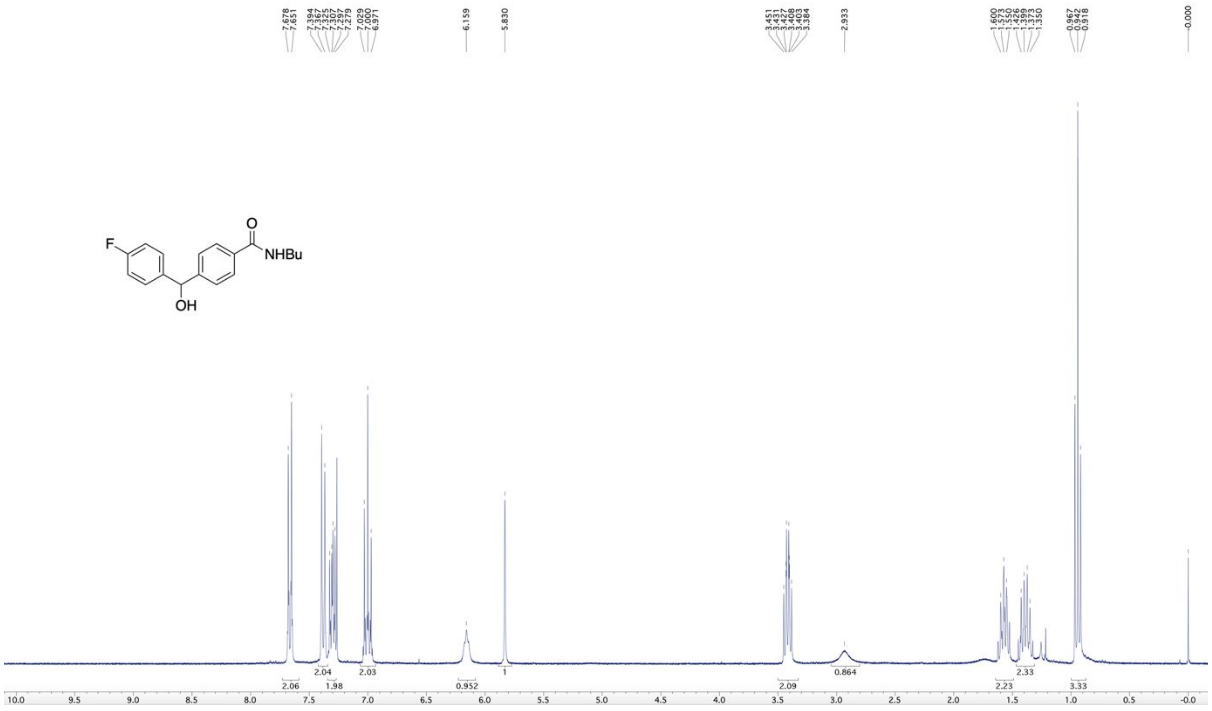

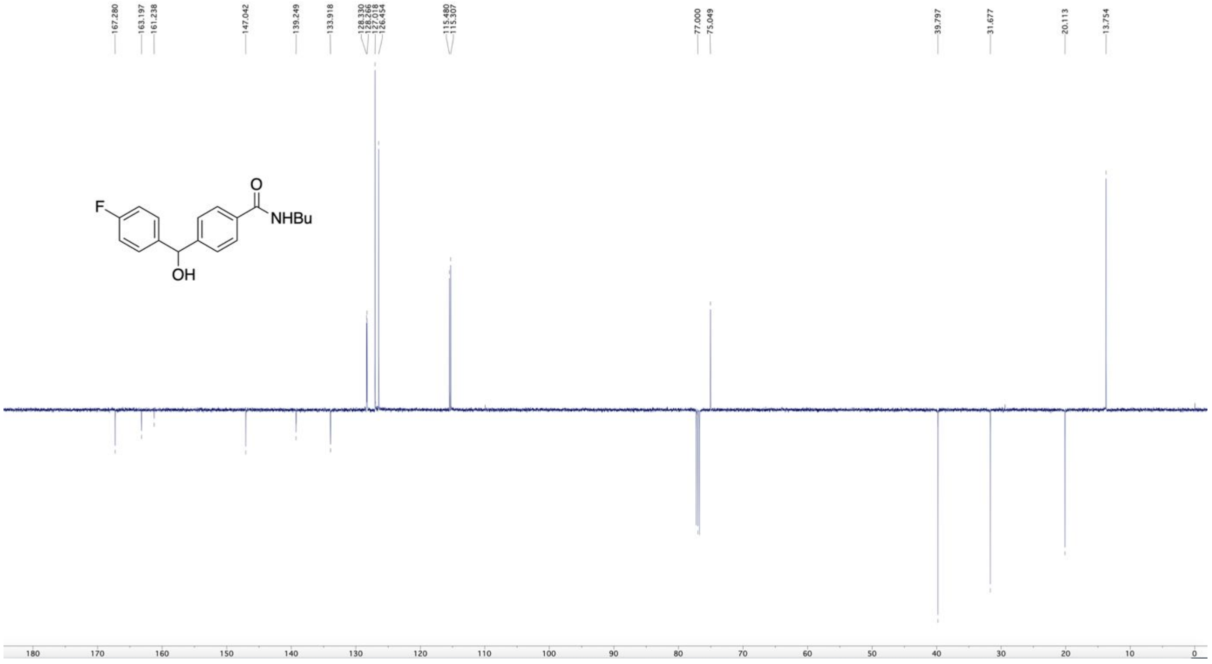

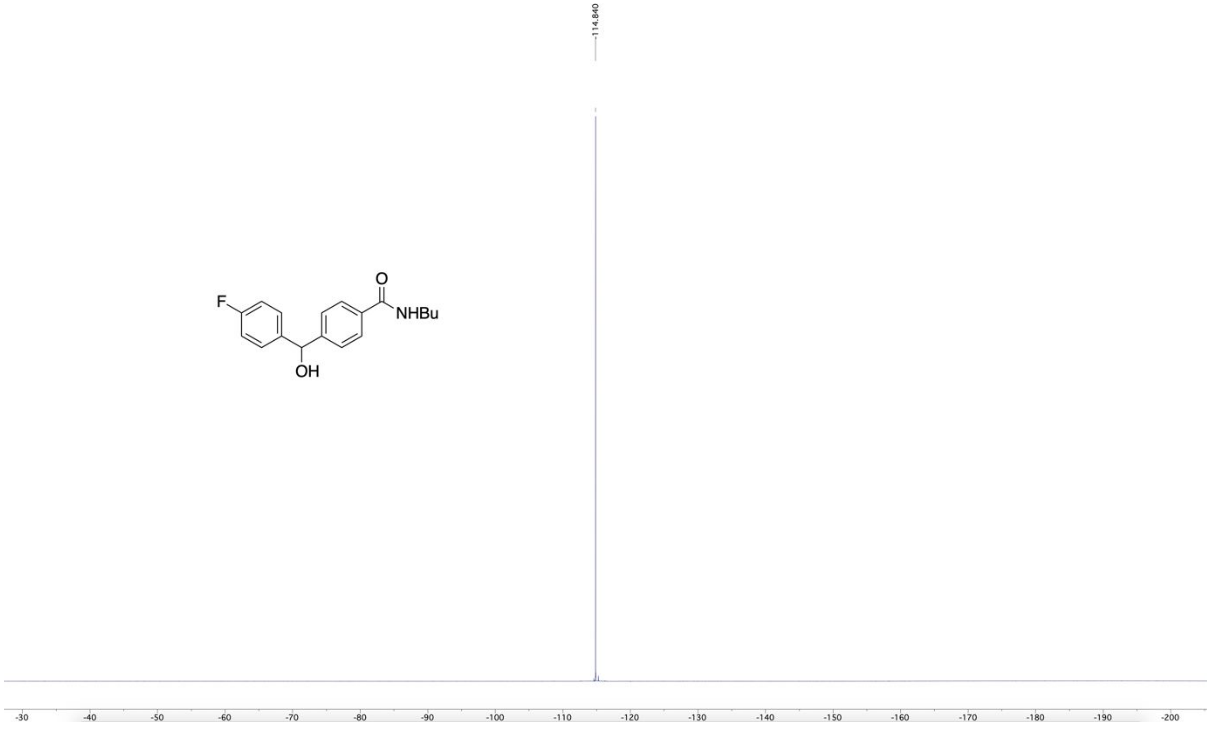

N-Butyl-4-((2-fluorophenyl)(hydroxy)methyl)benzamide **30h**

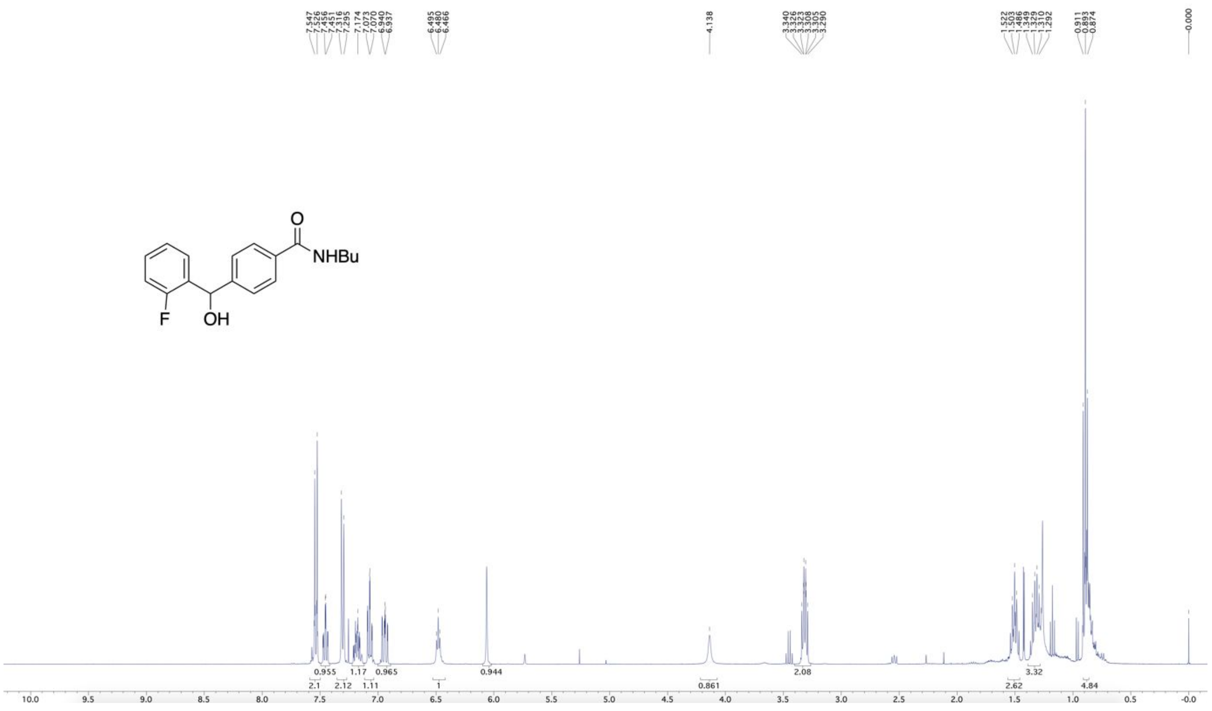

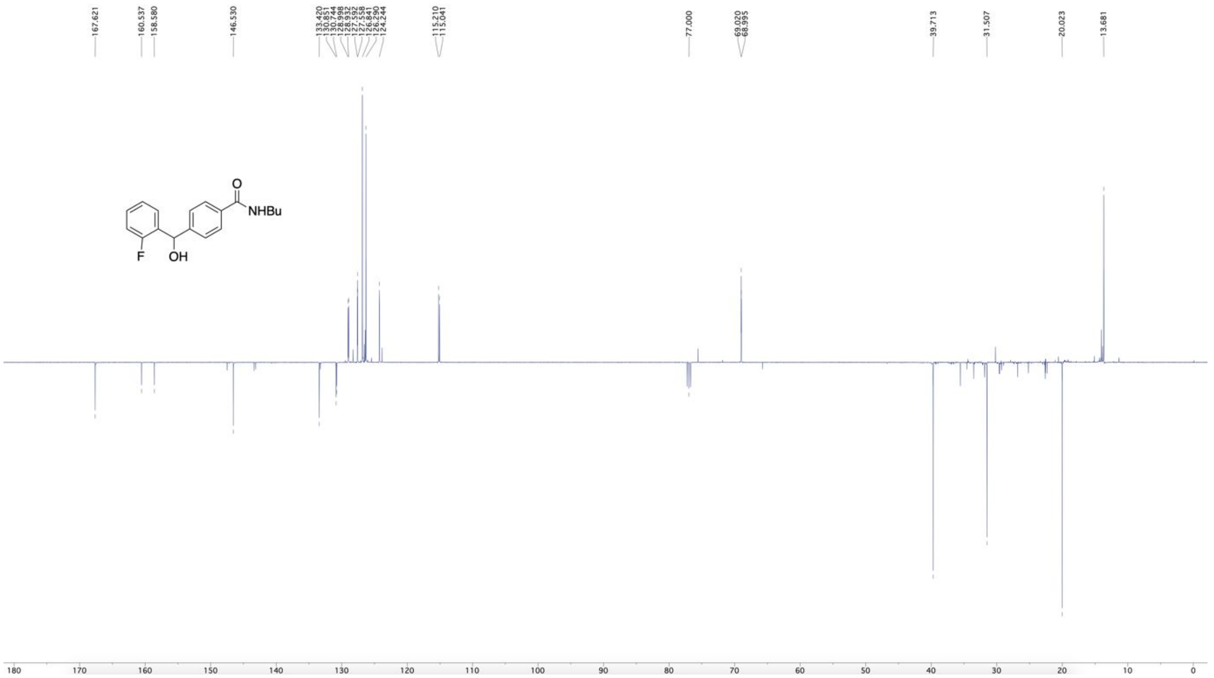

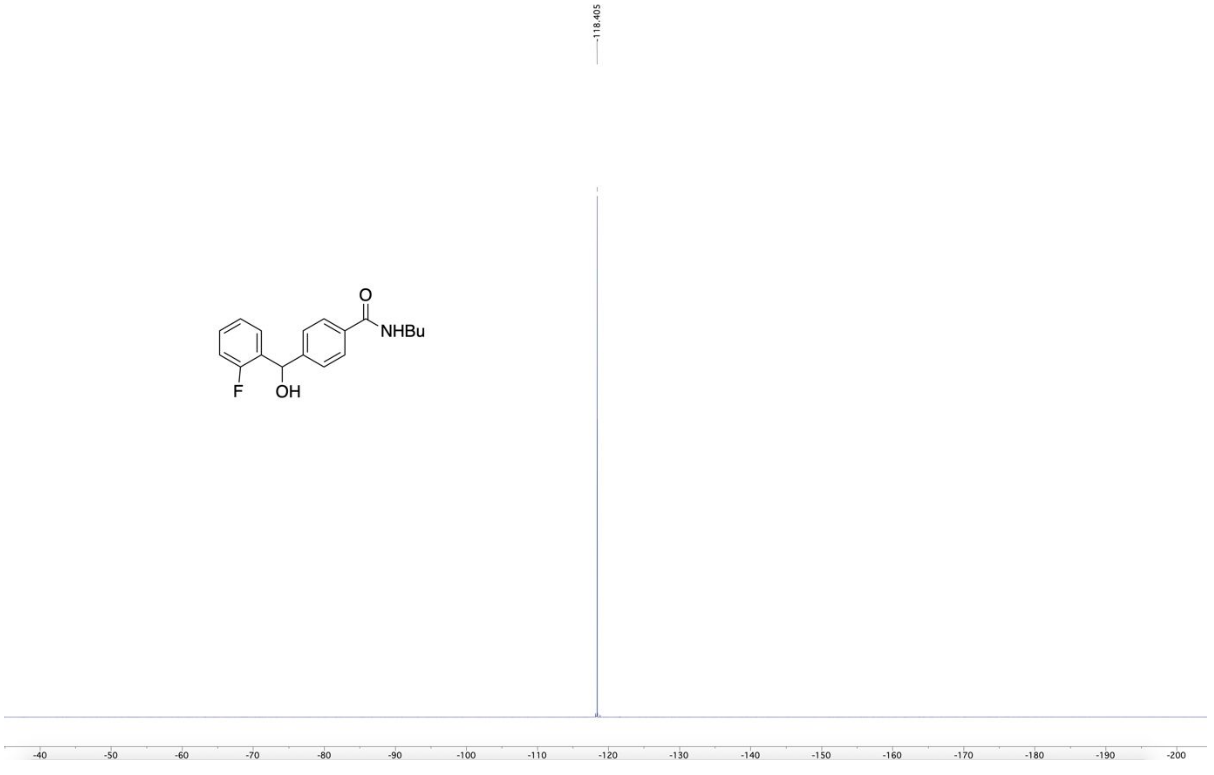

N-Butyl-4-(1-hydroxy-1-phenylethyl)benzamide **30o**

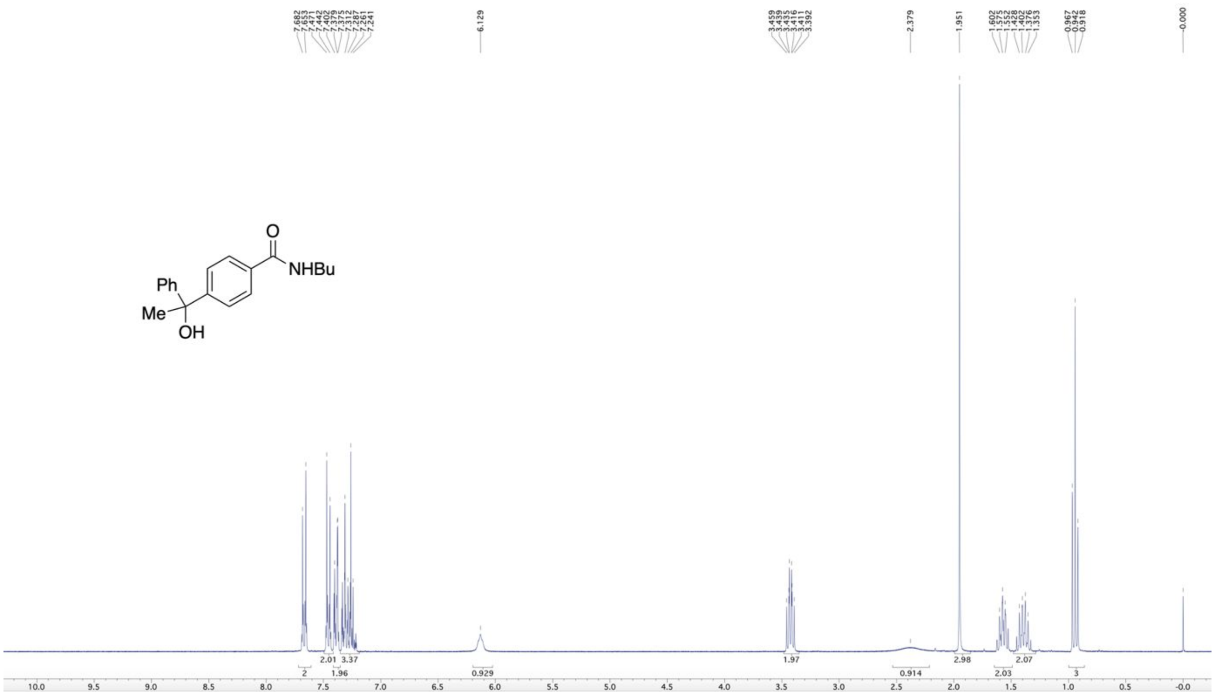

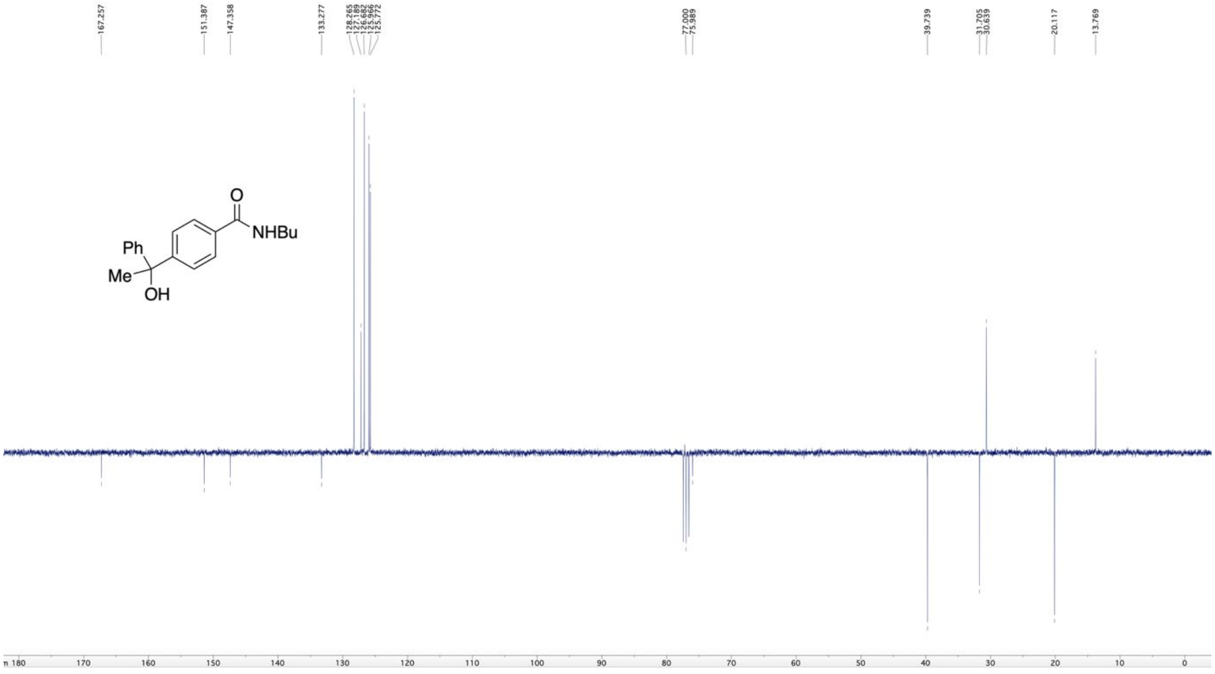

N-Butyl-4-(1-hydroxy-3-methylbut-2-en-1-yl)benzamide **30p**

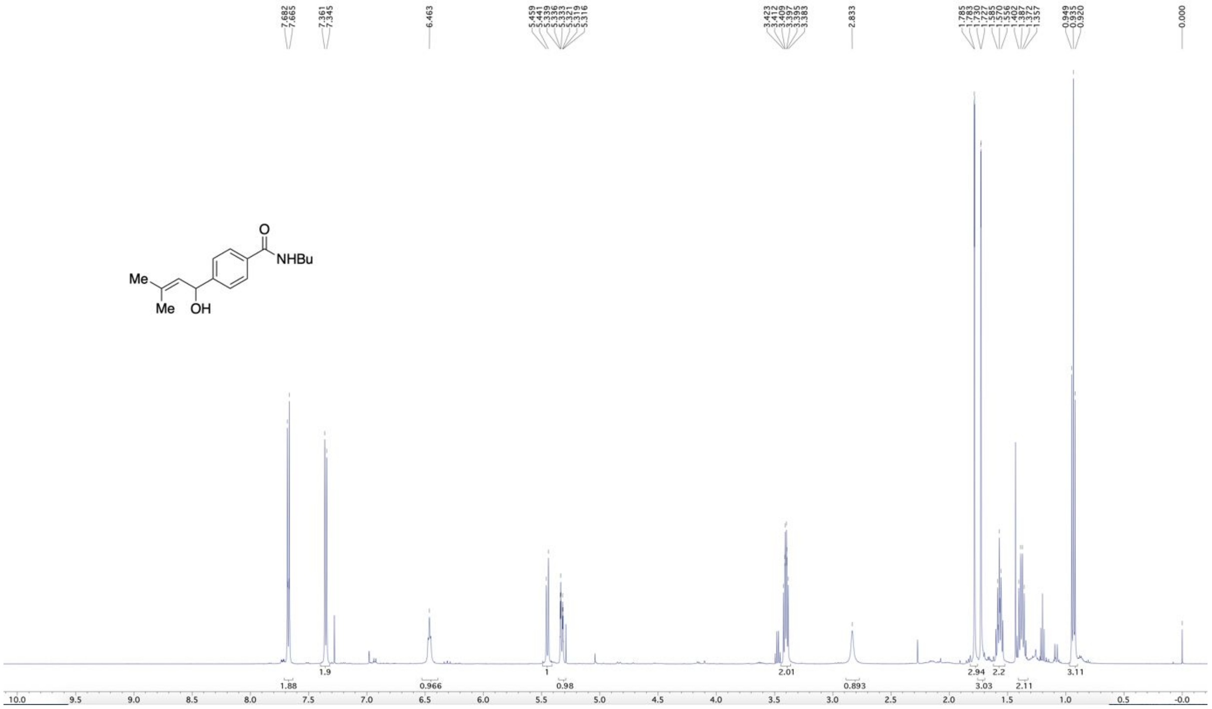

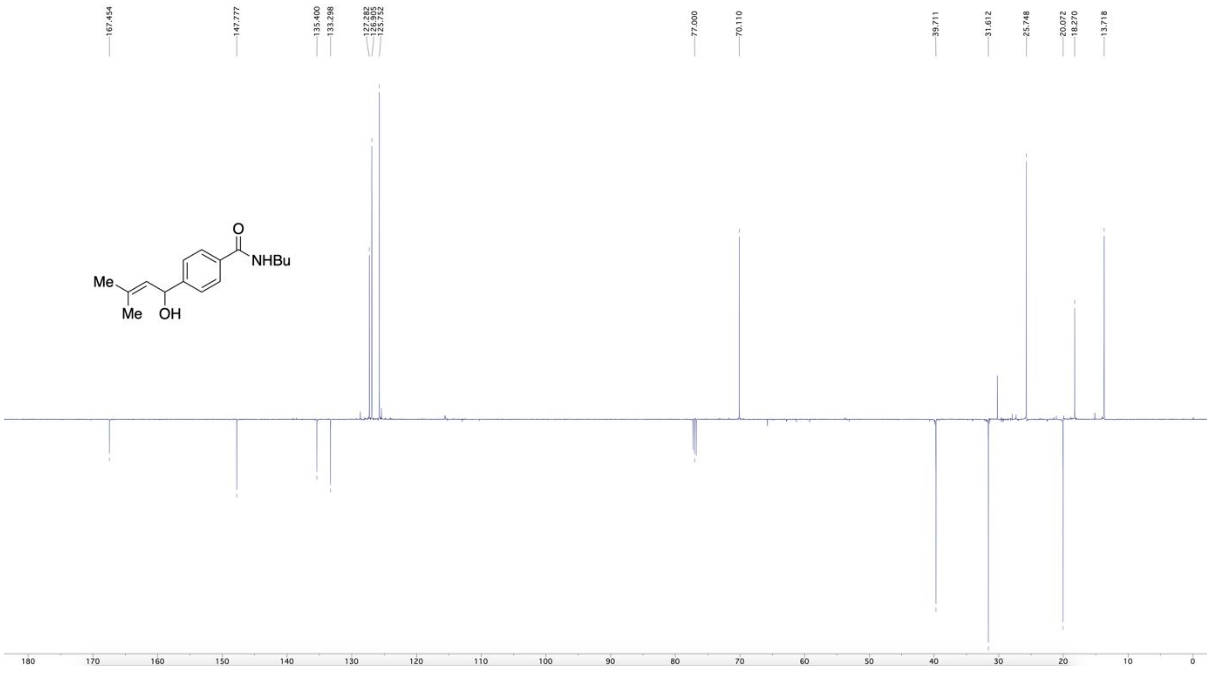

2,4-Bis(benzyloxy)-*N*-butylbenzamide **31**

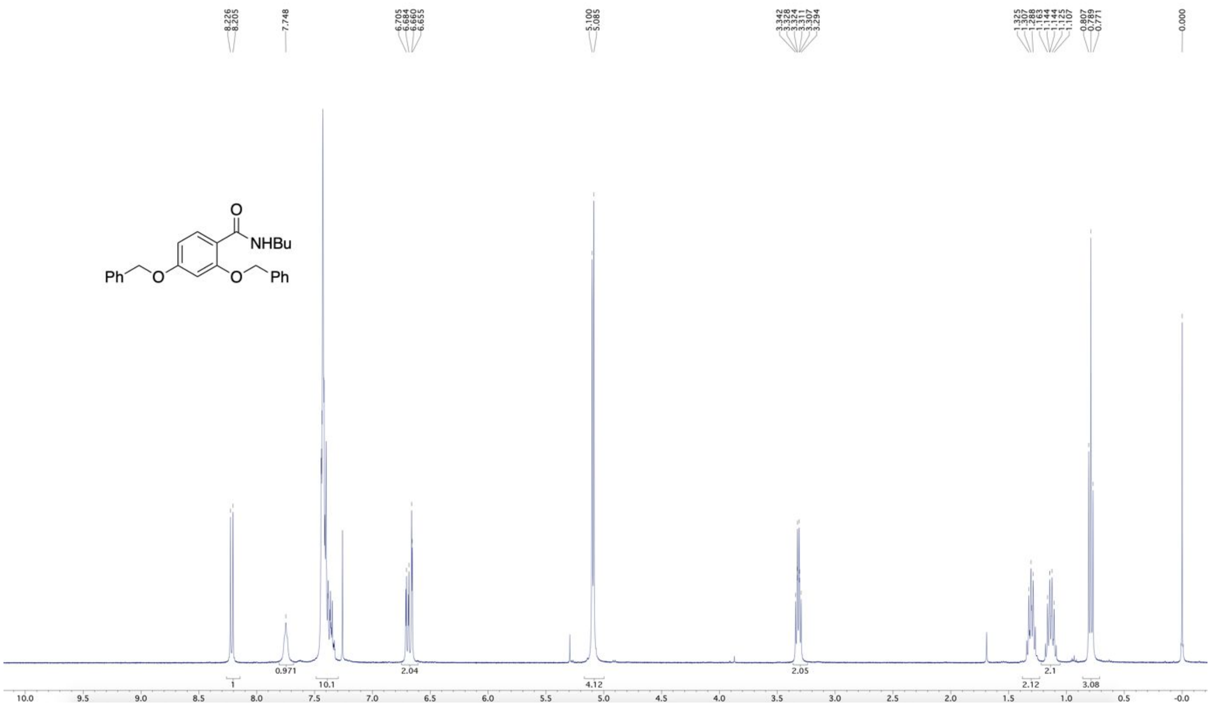

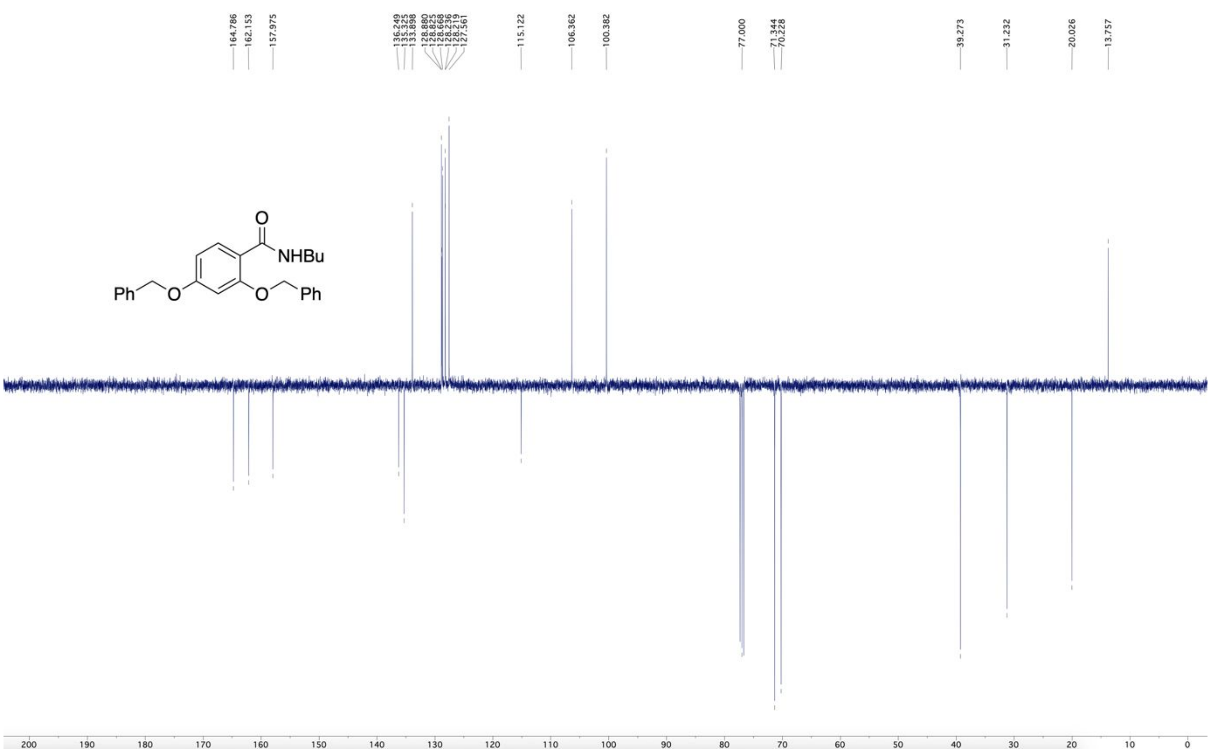

4-(Benzyloxy)-*N*-butyl-2-(hydroxy(phenyl)methyl)benzamide **32**

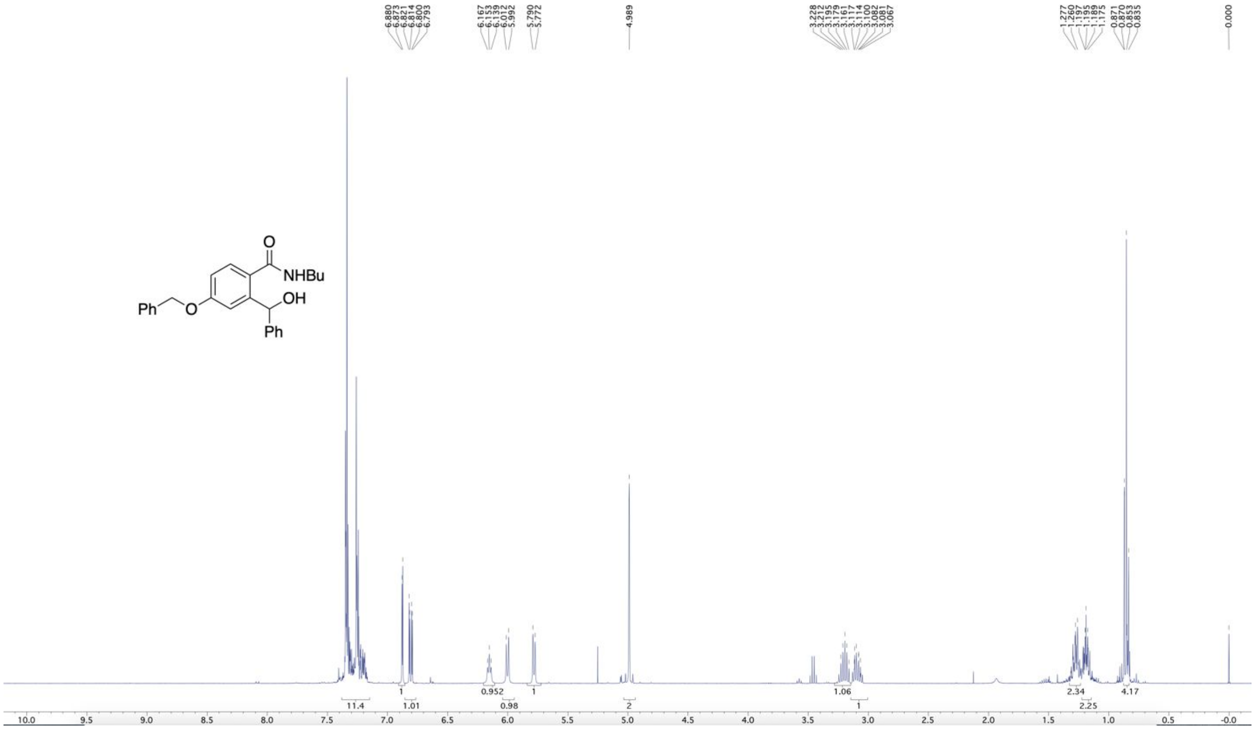

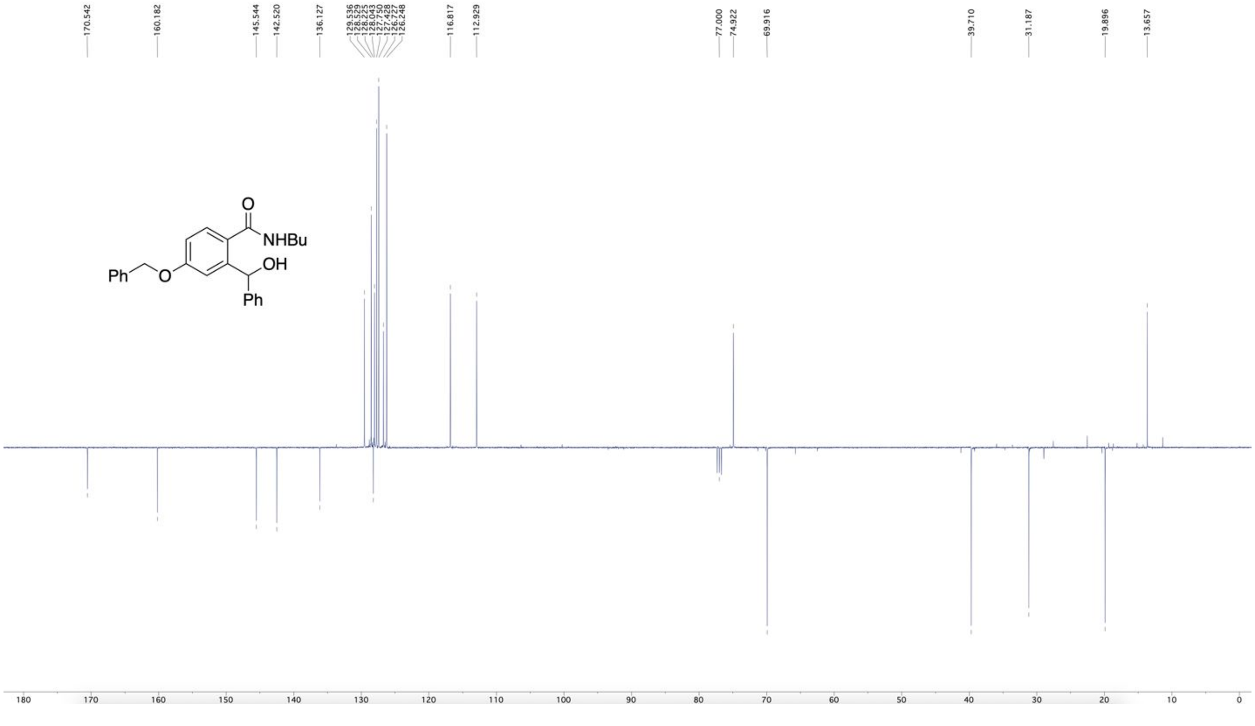

2-(Benzyloxy)-*N*-butyl-4-(hydroxy(phenyl)methyl)benzamide **33**

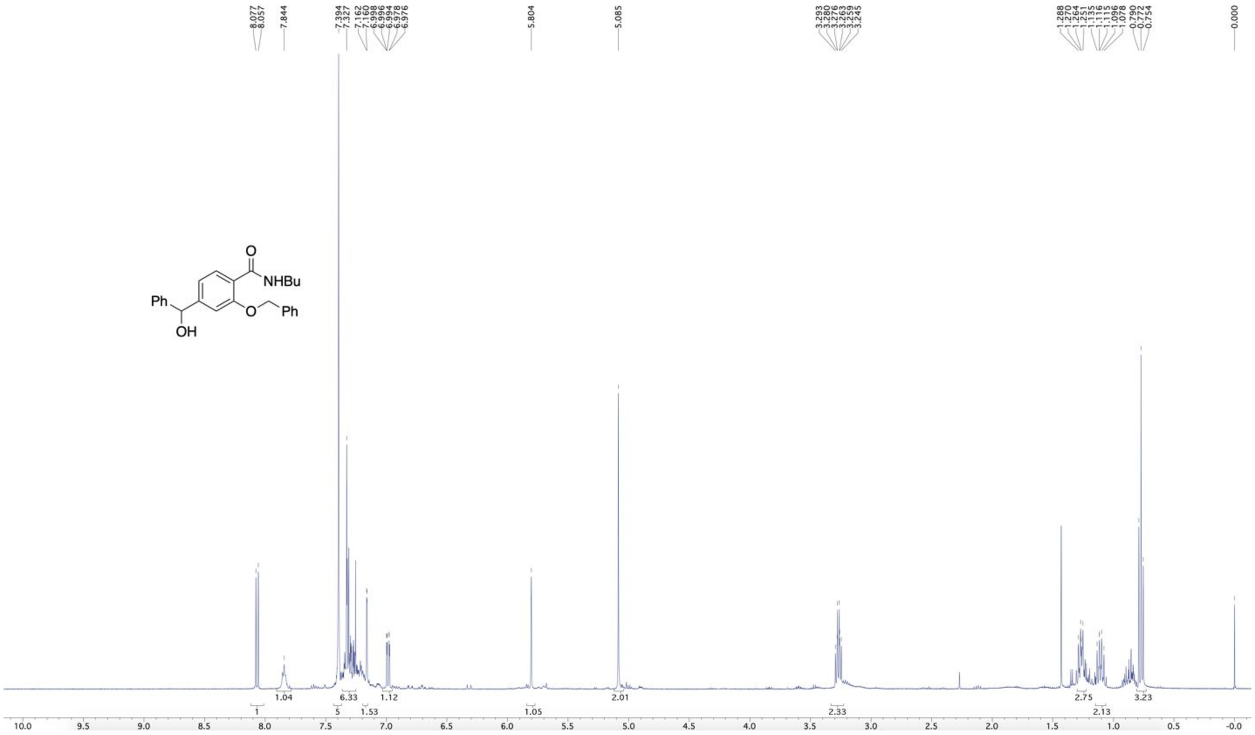

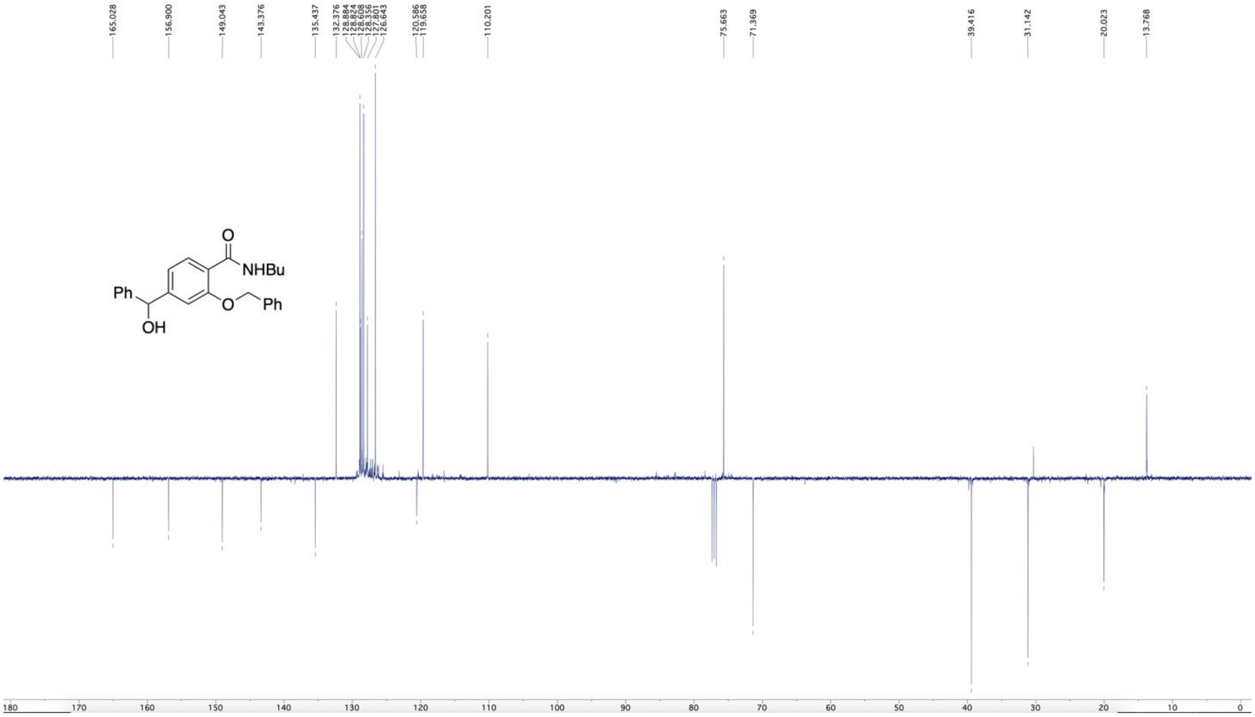

5-(Benzyloxy)-3-phenylphthalide **34**

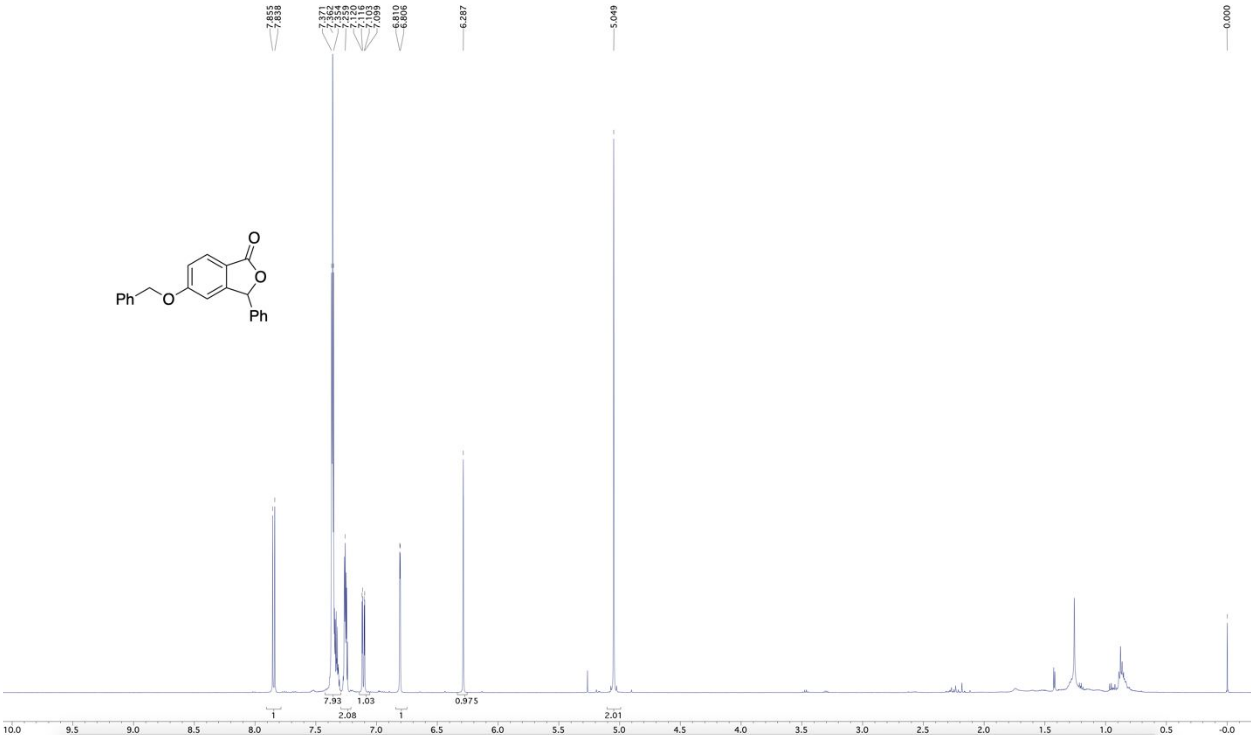

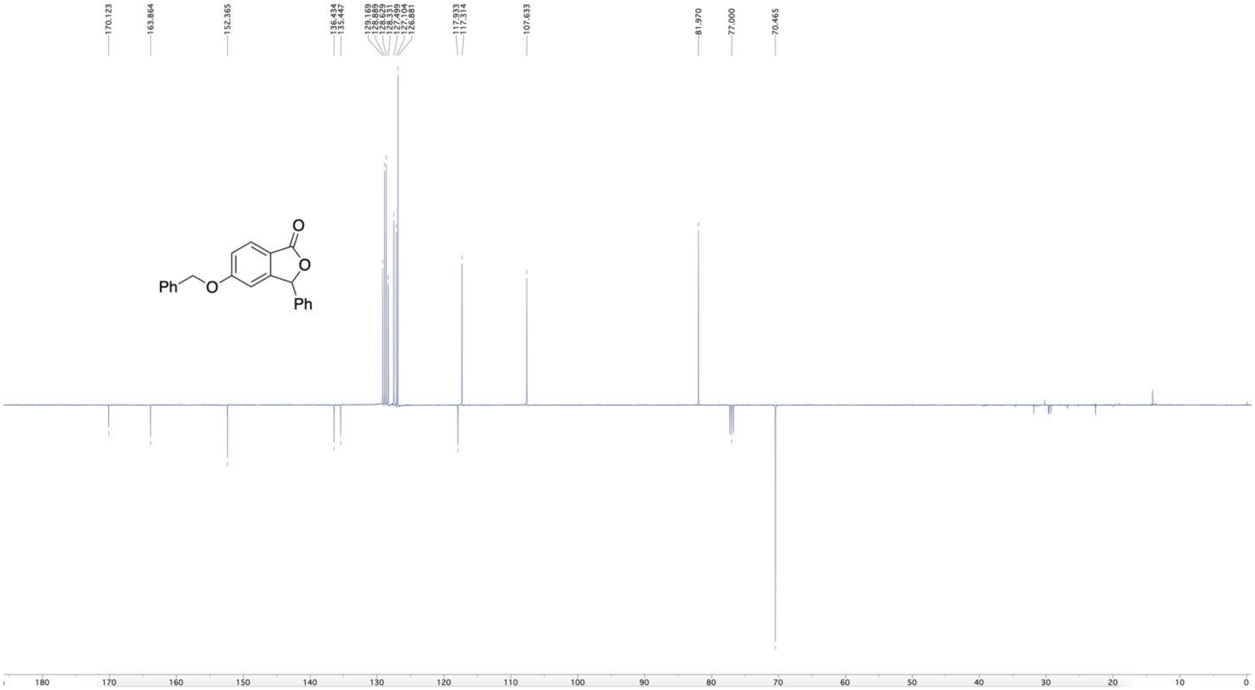

N-Butyl-2,4-bis(hydroxy(phenyl)methyl)benzamide **35**

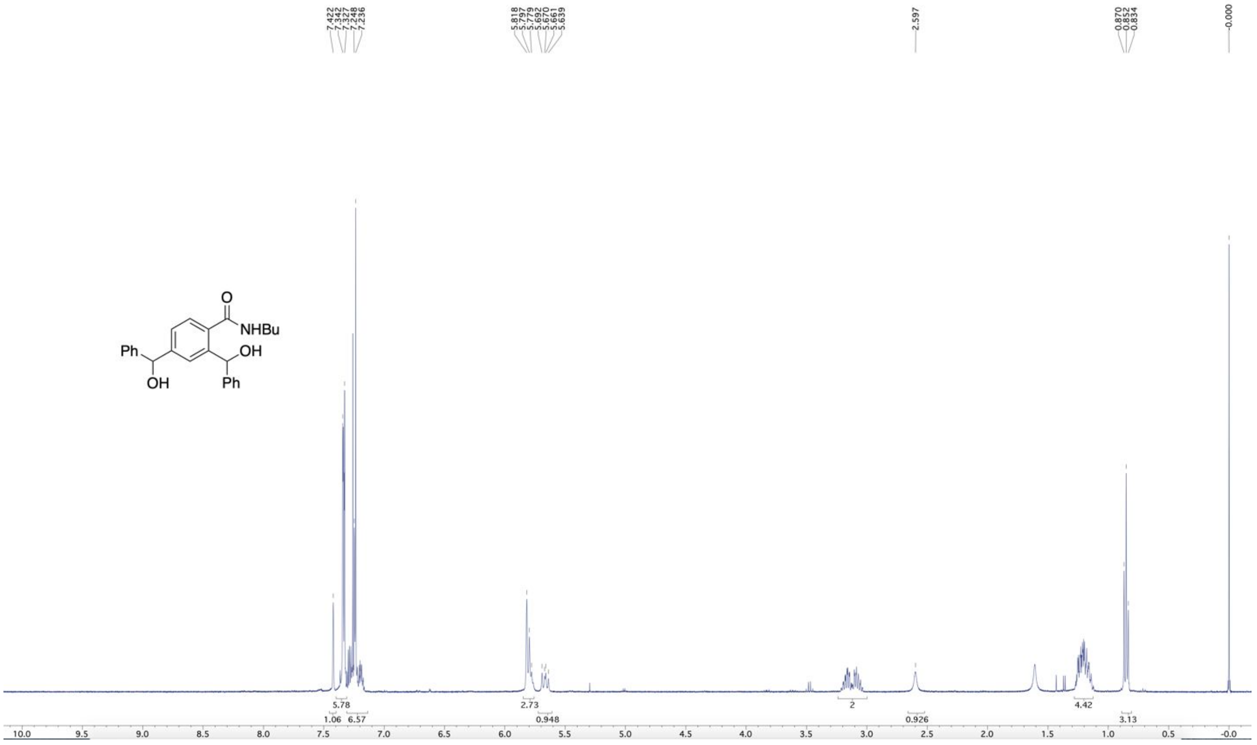

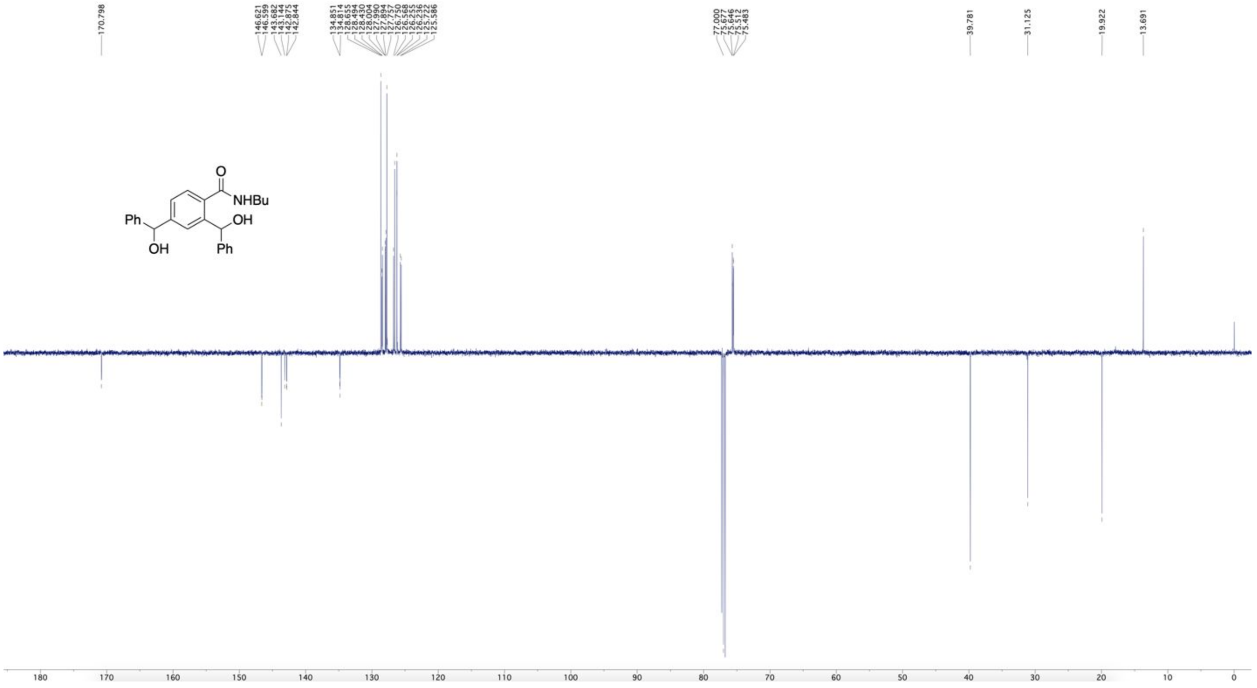

3-(benzyloxy)-*N*-butylbenzamide **37a**

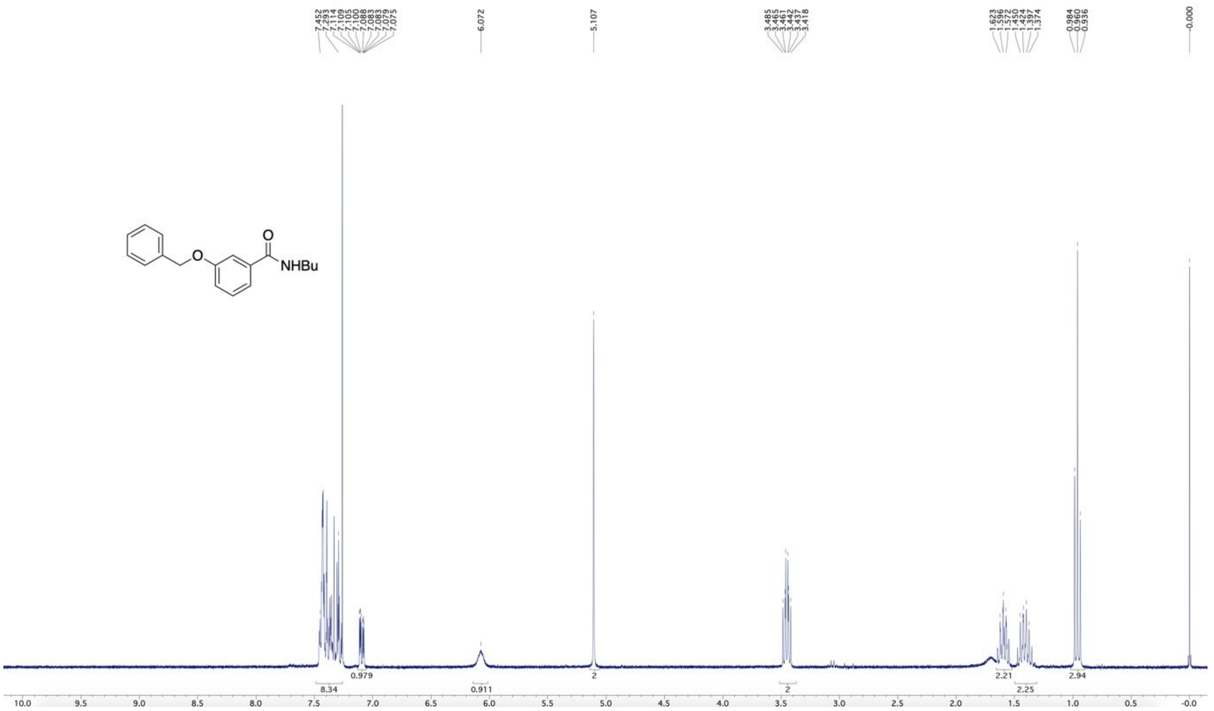

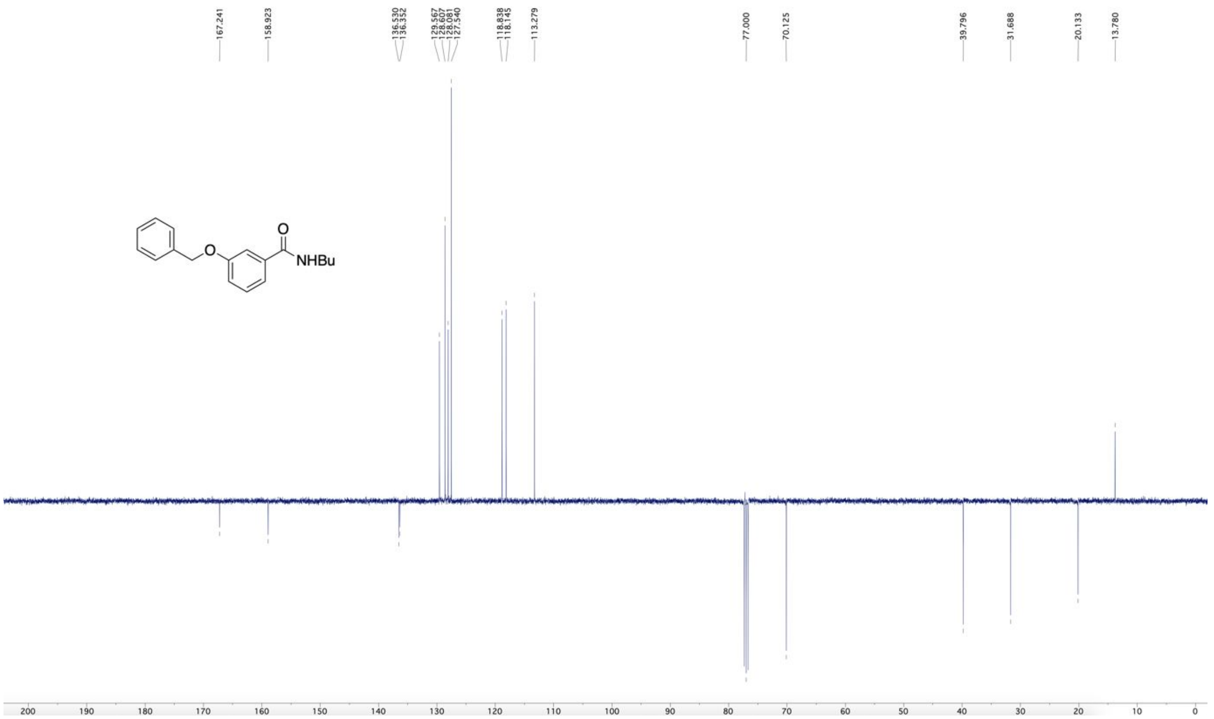

N-butyl-3-((4-(*tert*-butyl)benzyl)oxy)benzamide **37b**

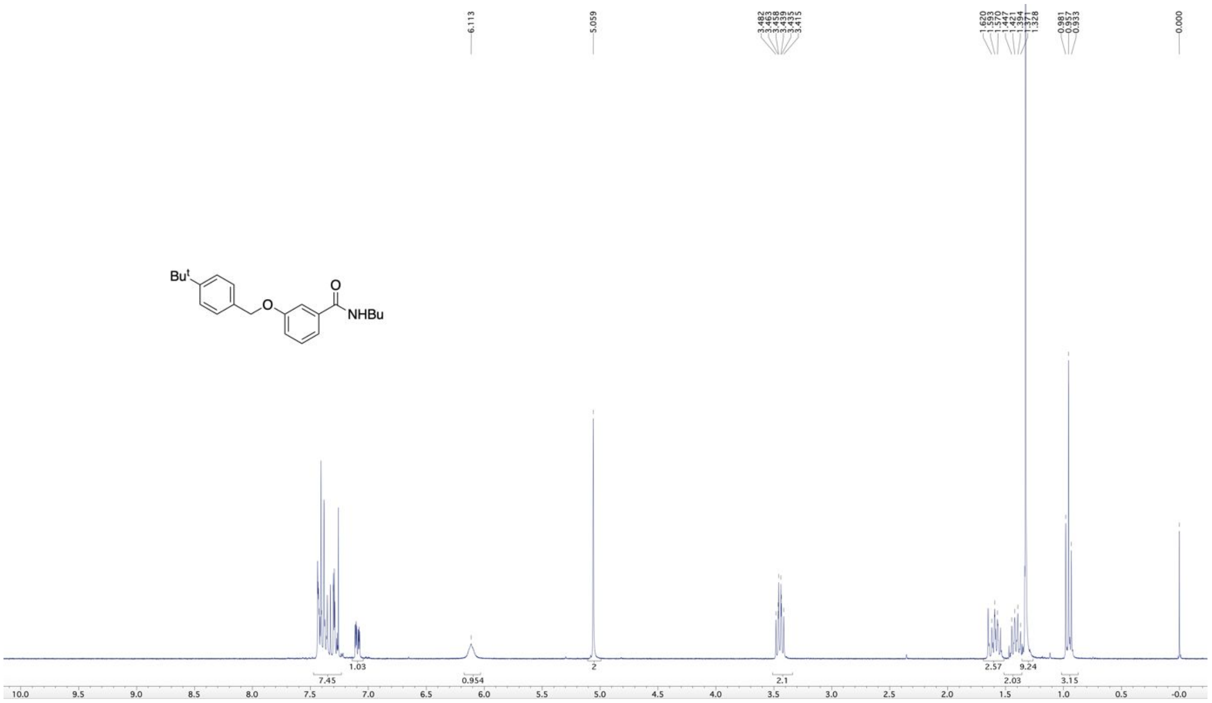

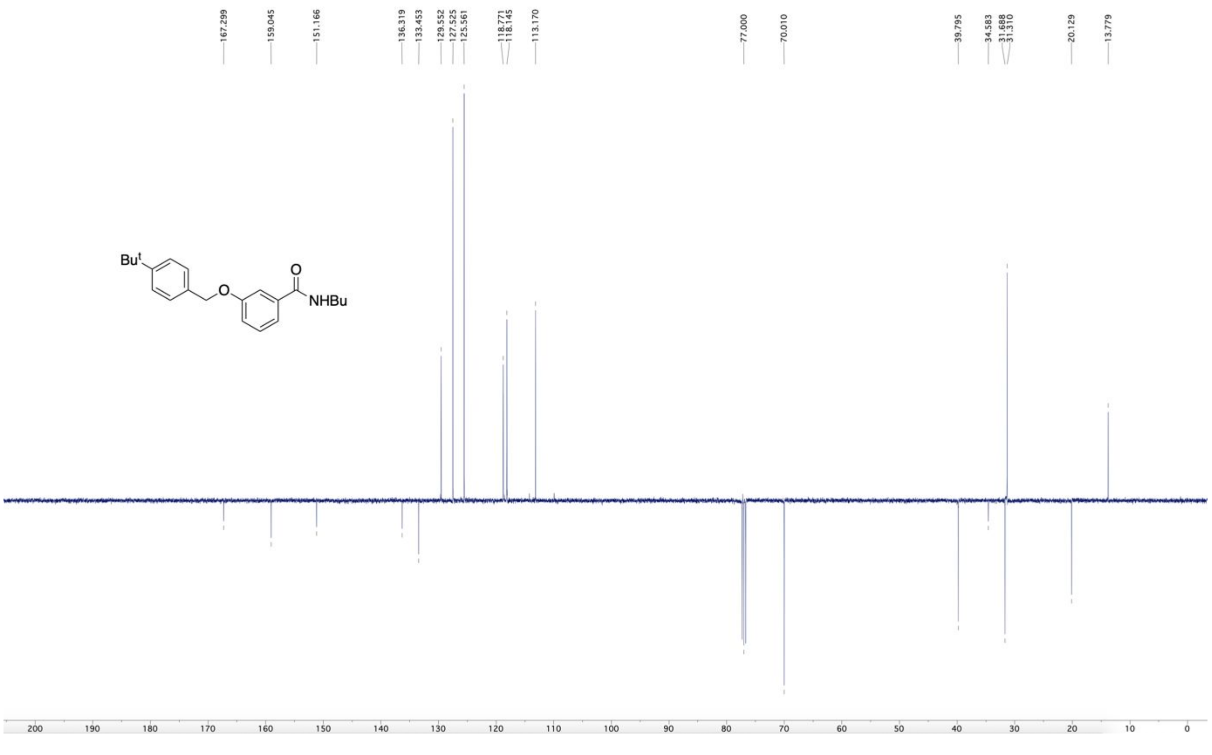

N-butyl-3-((2-methylbenzyl)oxy)benzamide **37c**

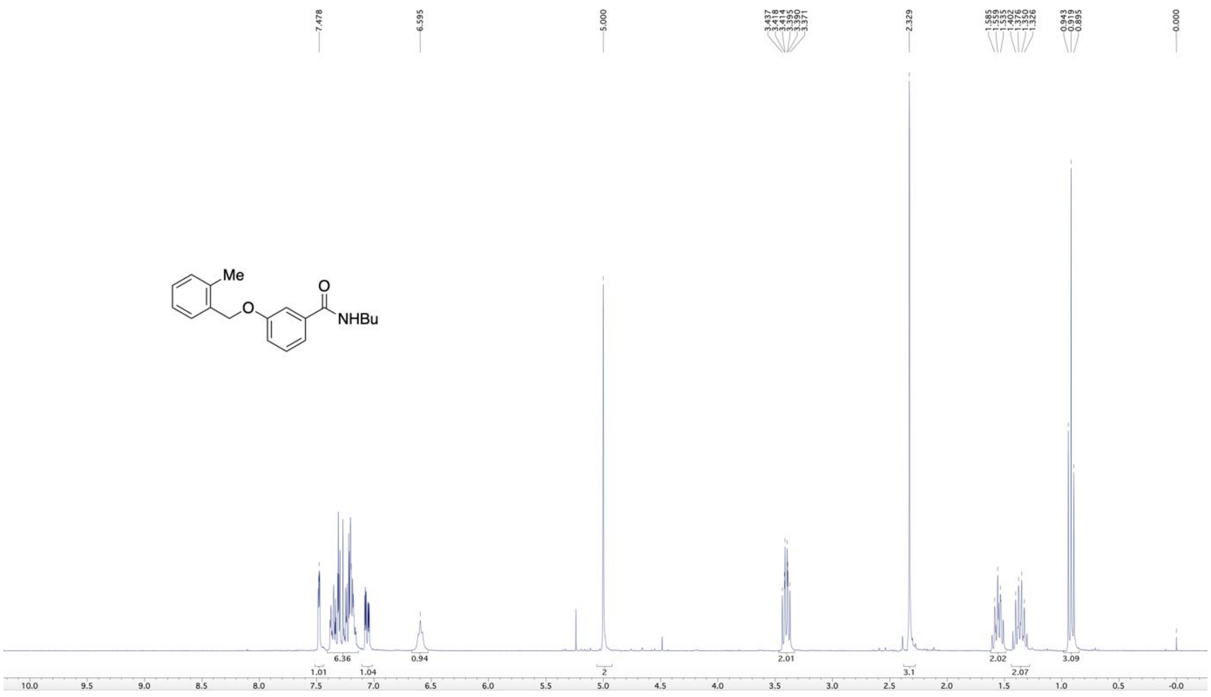

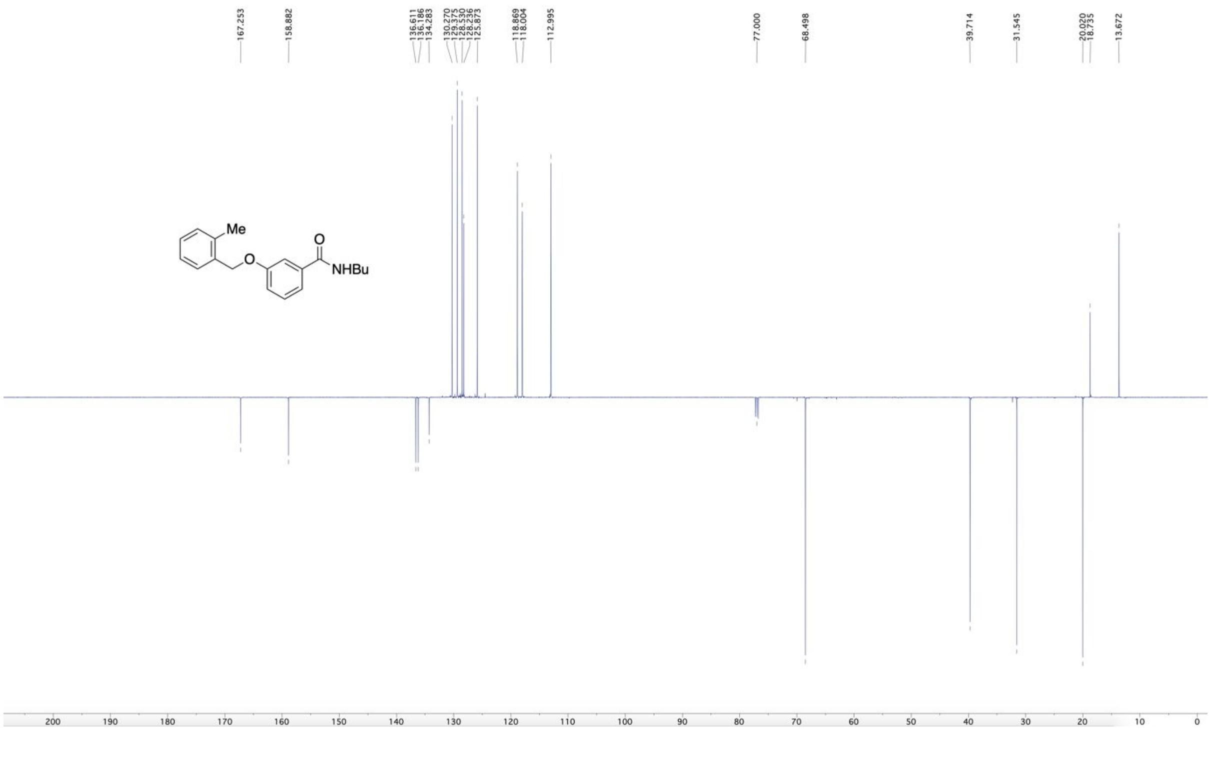

N-butyl-3-((2,4,6-trimethylbenzyl)oxy)benzamide **37d**

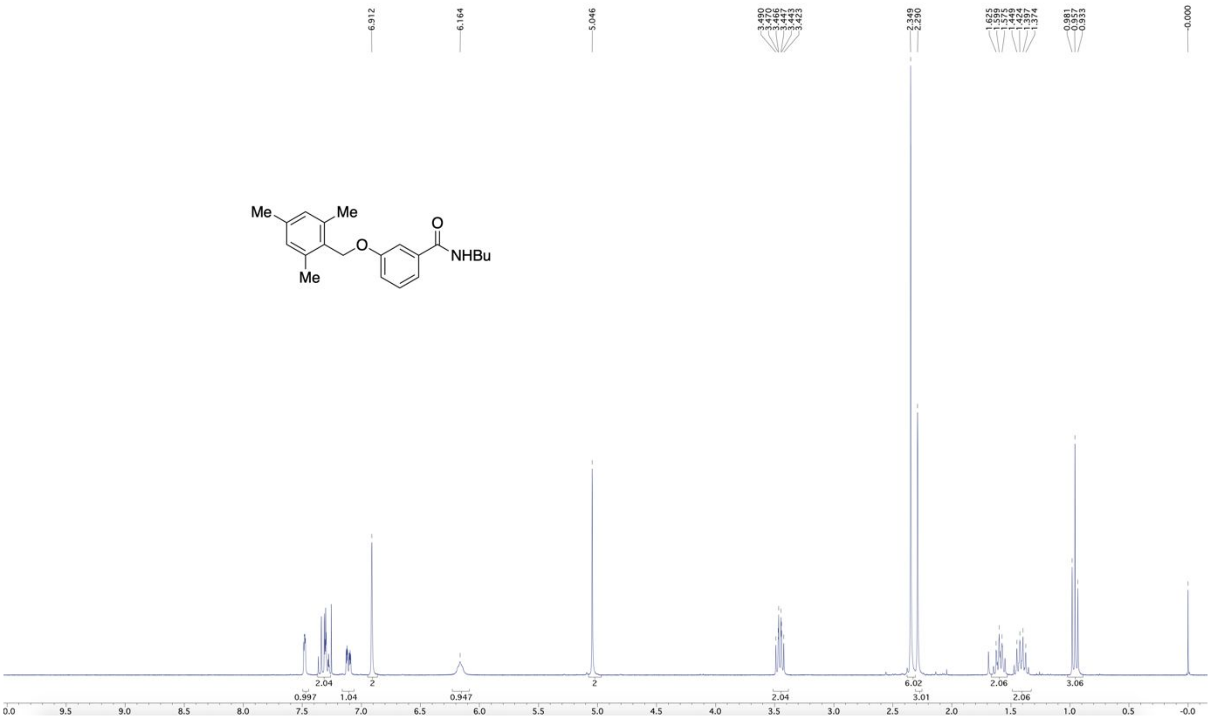

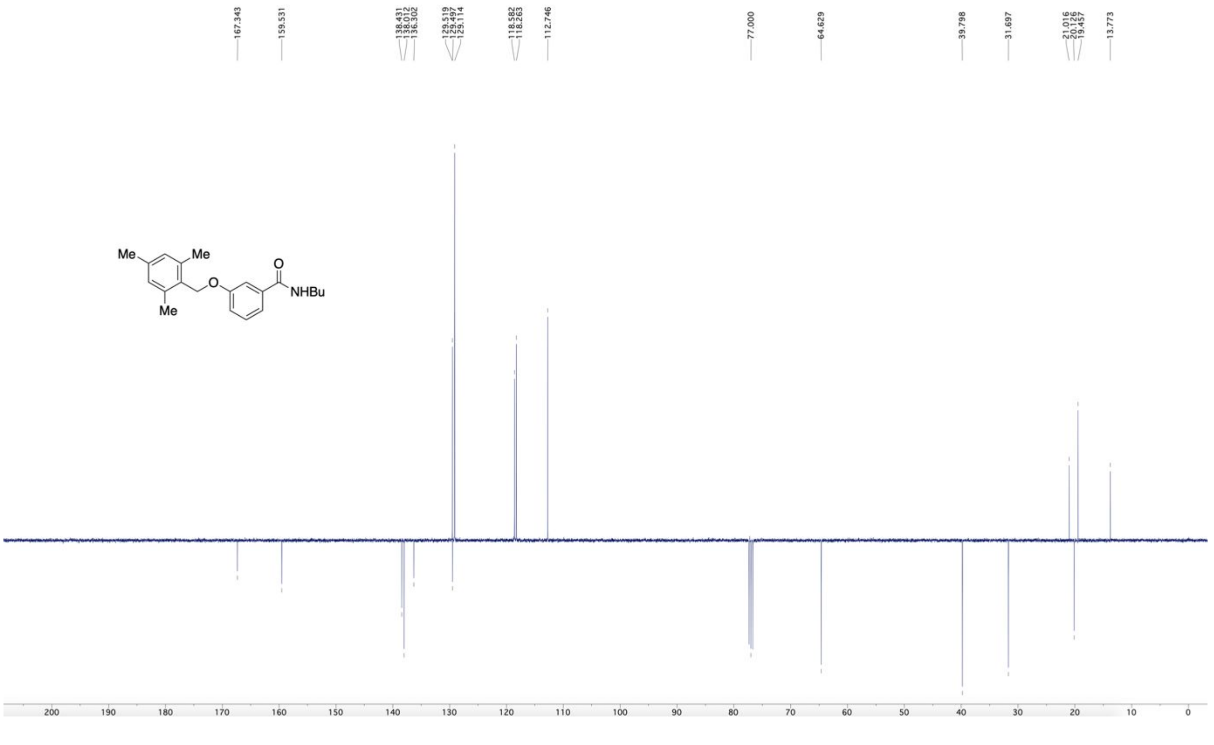

N-butyl-3-((2-nitrobenzyl)oxy)benzamide **37e**

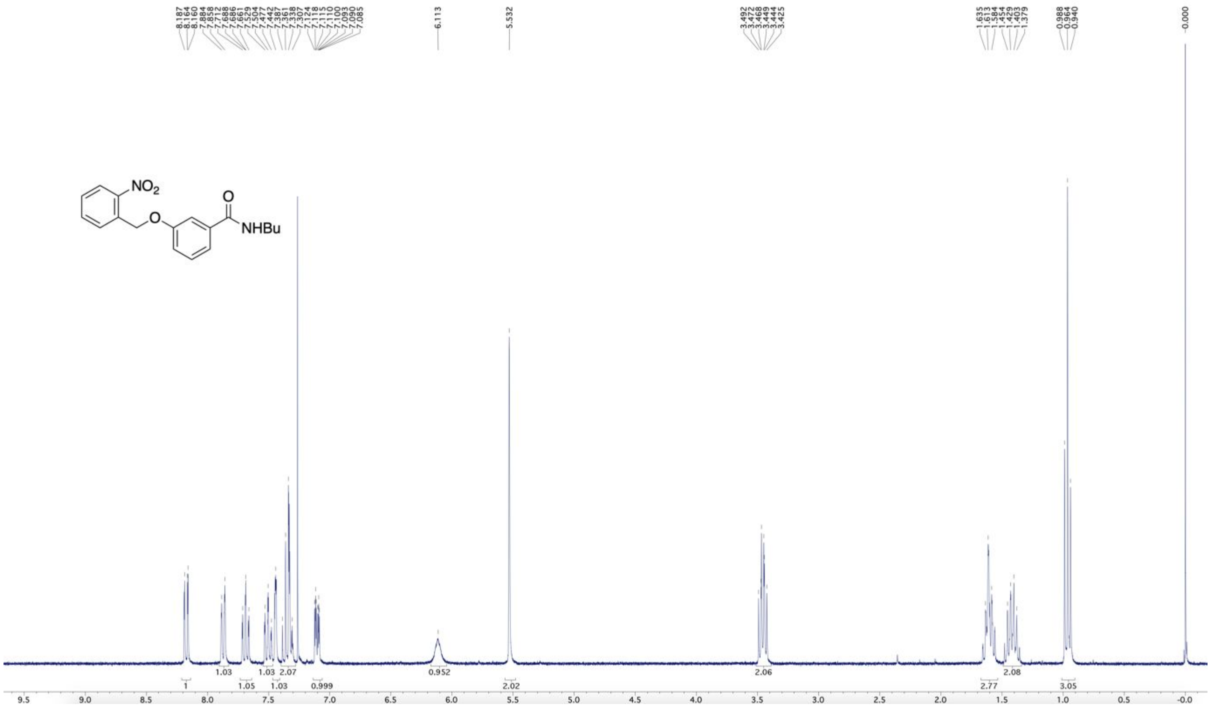

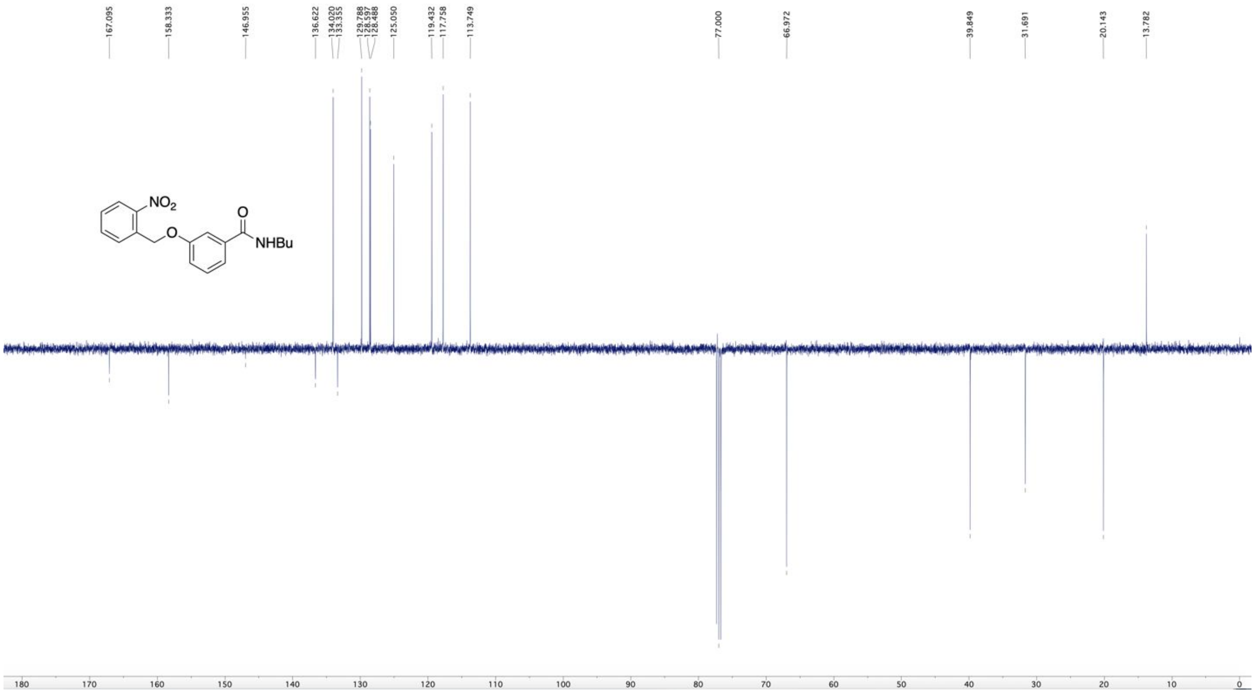

N-butyl-3-((3-nitrobenzyl)oxy)benzamide **37f**

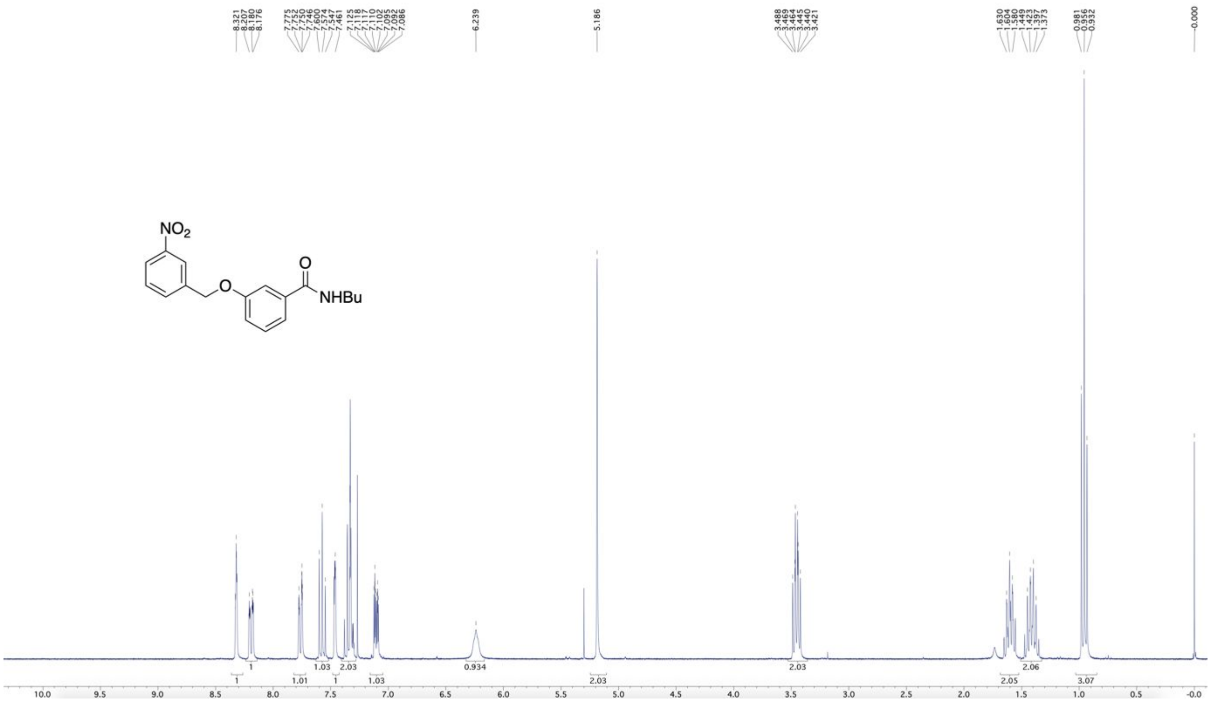

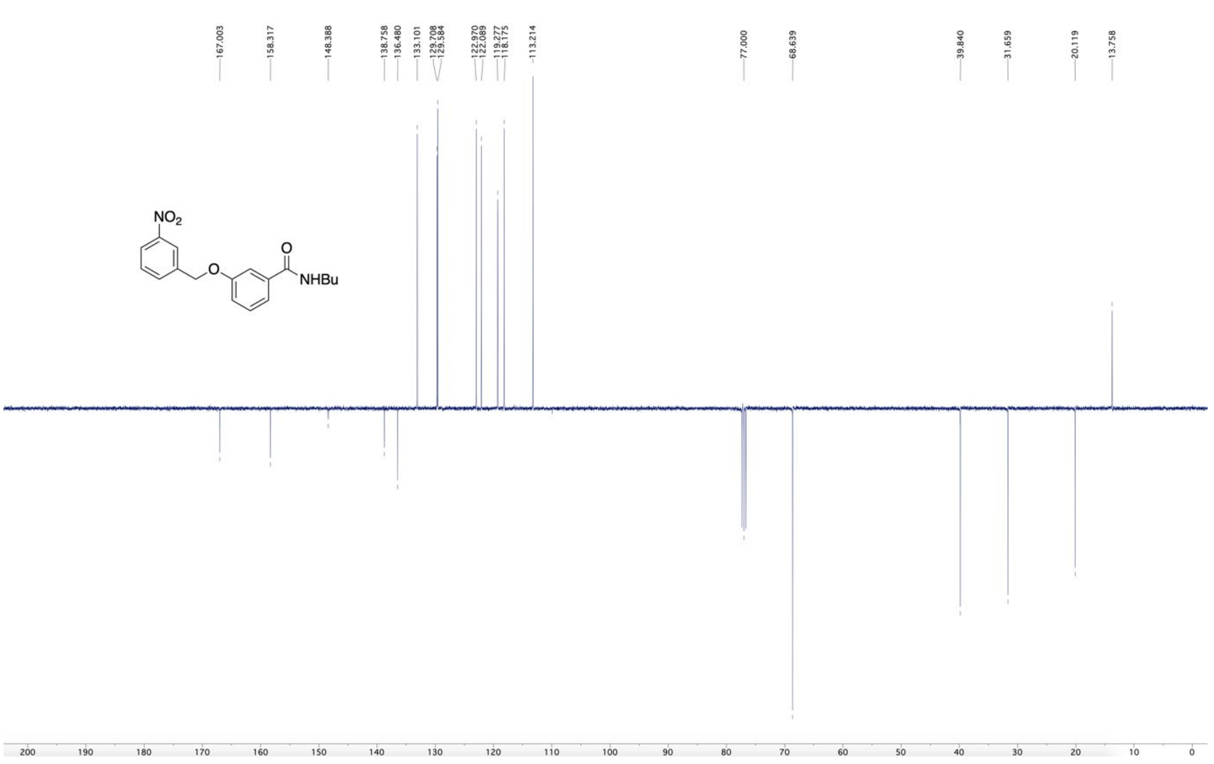

N-butyl-3-((2-methoxybenzyl)oxy)benzamide **37g**

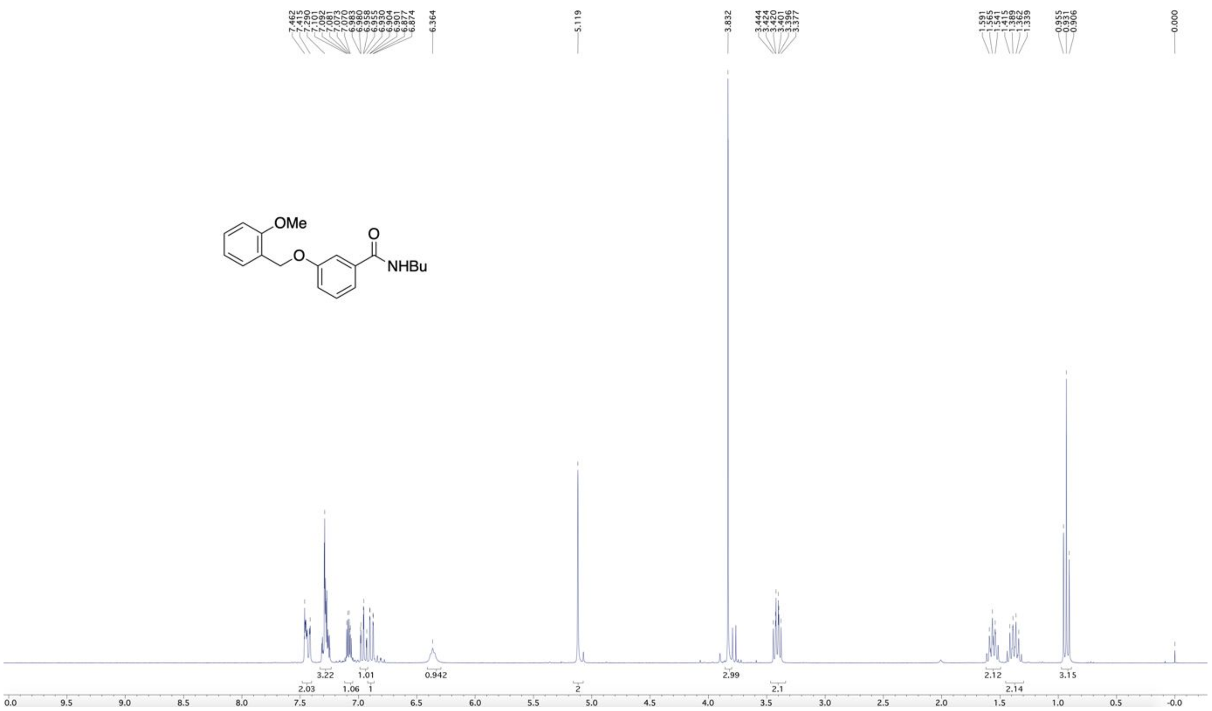

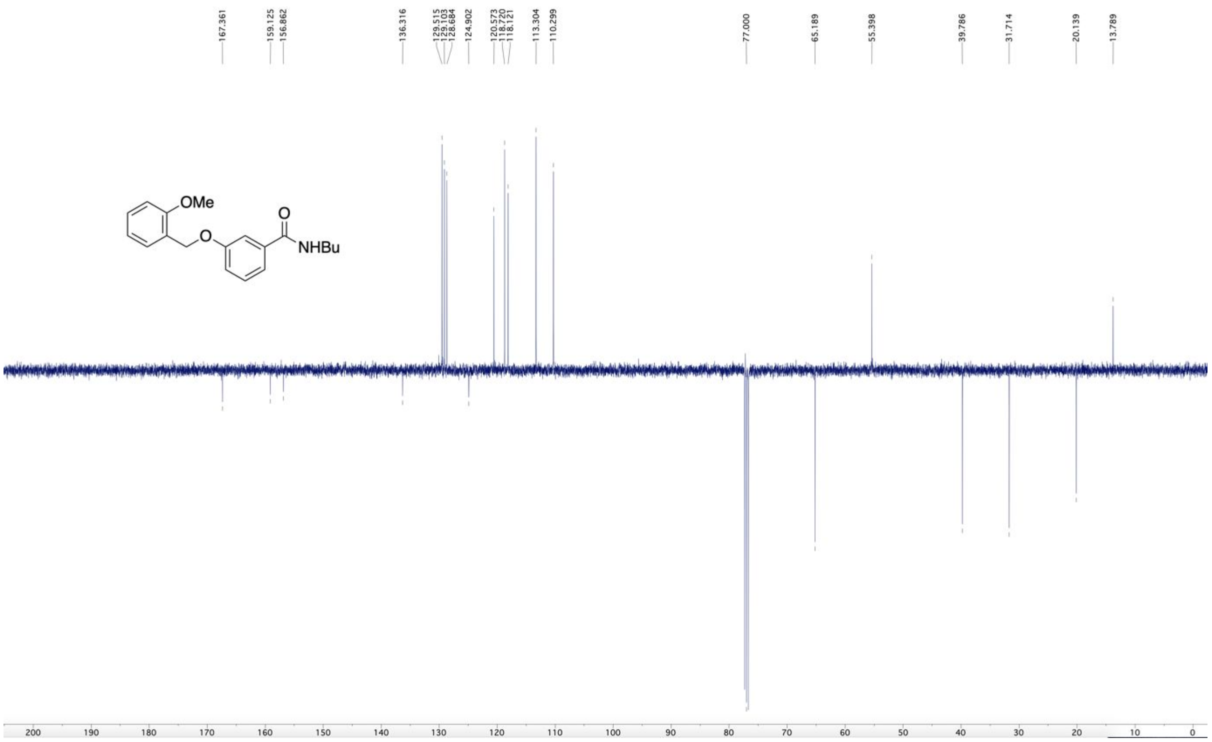

N-butyl-3-((4-methoxybenzyl)oxy)benzamide **37h**

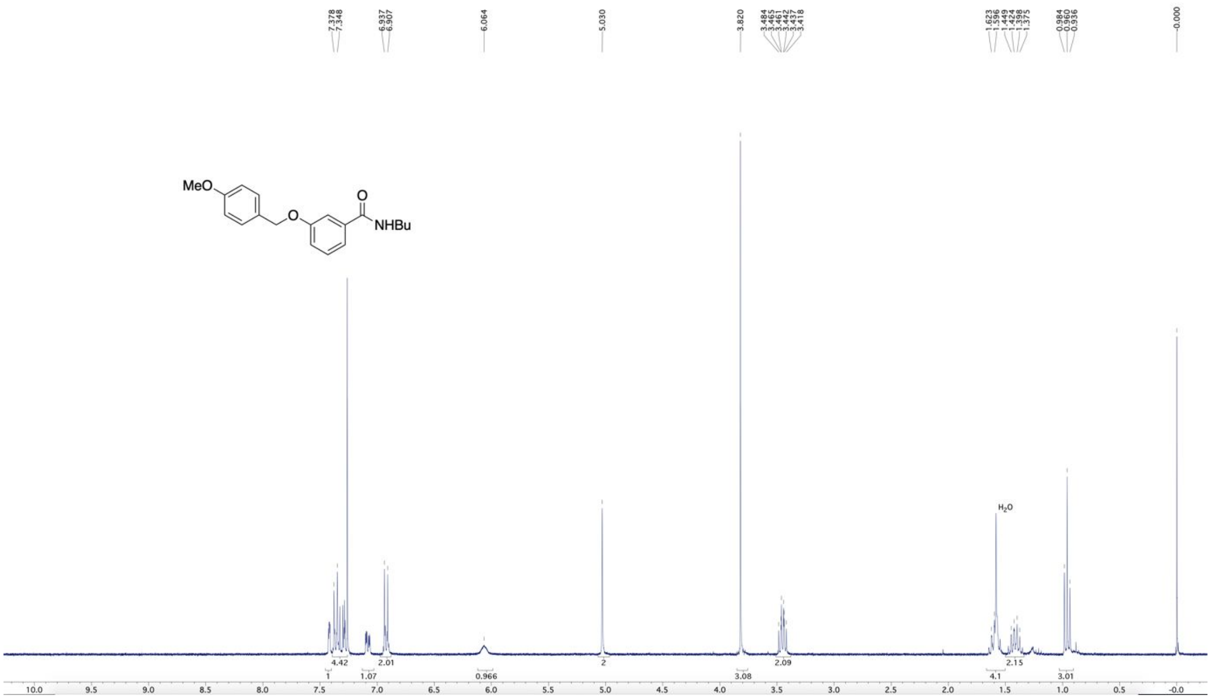

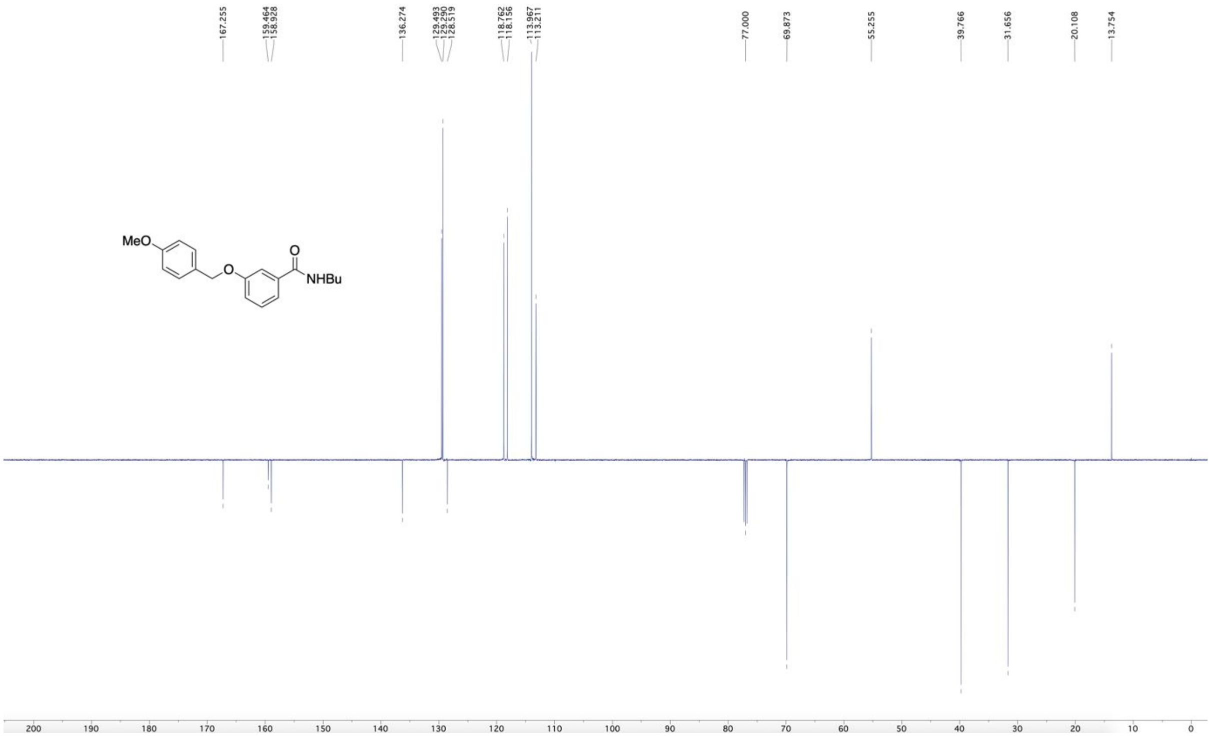

N-butyl-3-((2-fluorobenzyl)oxy)benzamide **37i**

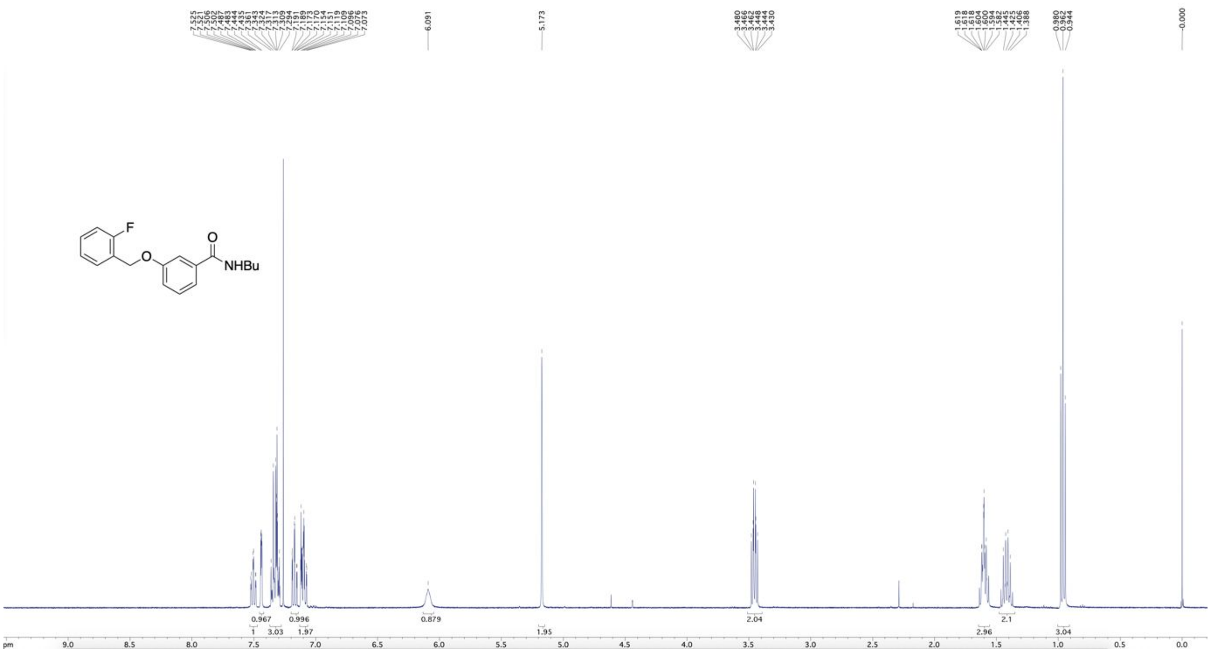

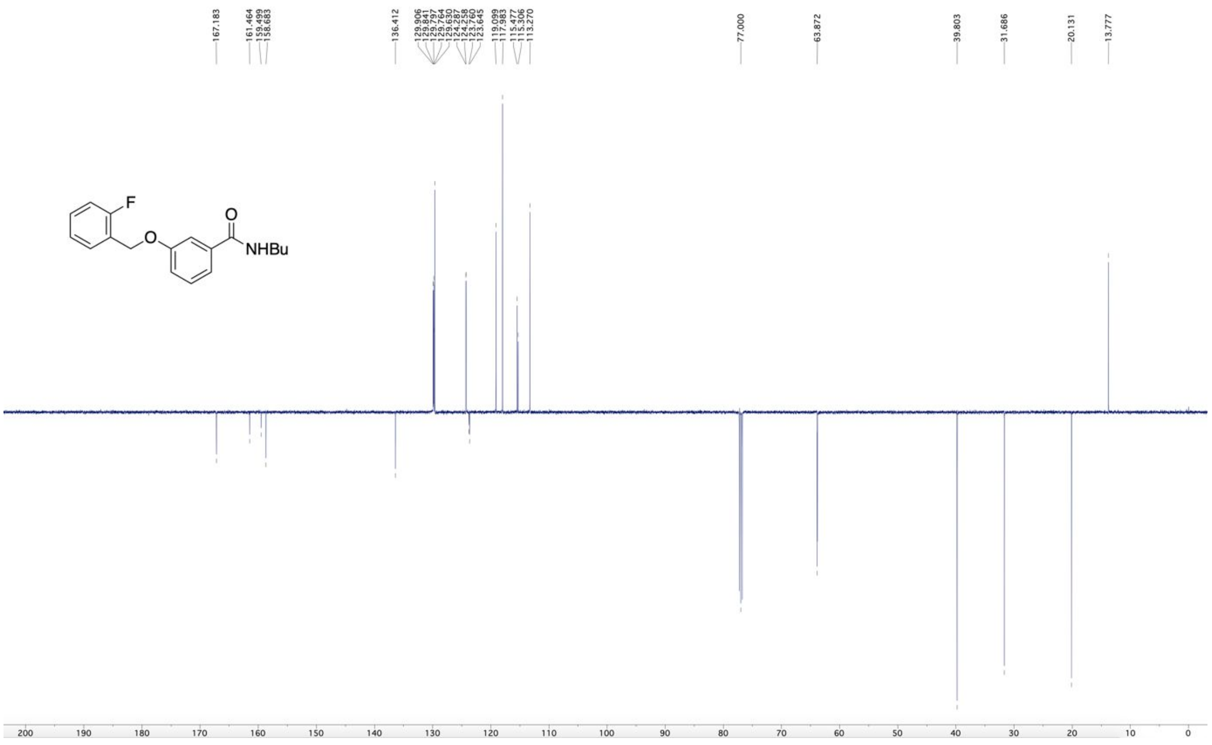

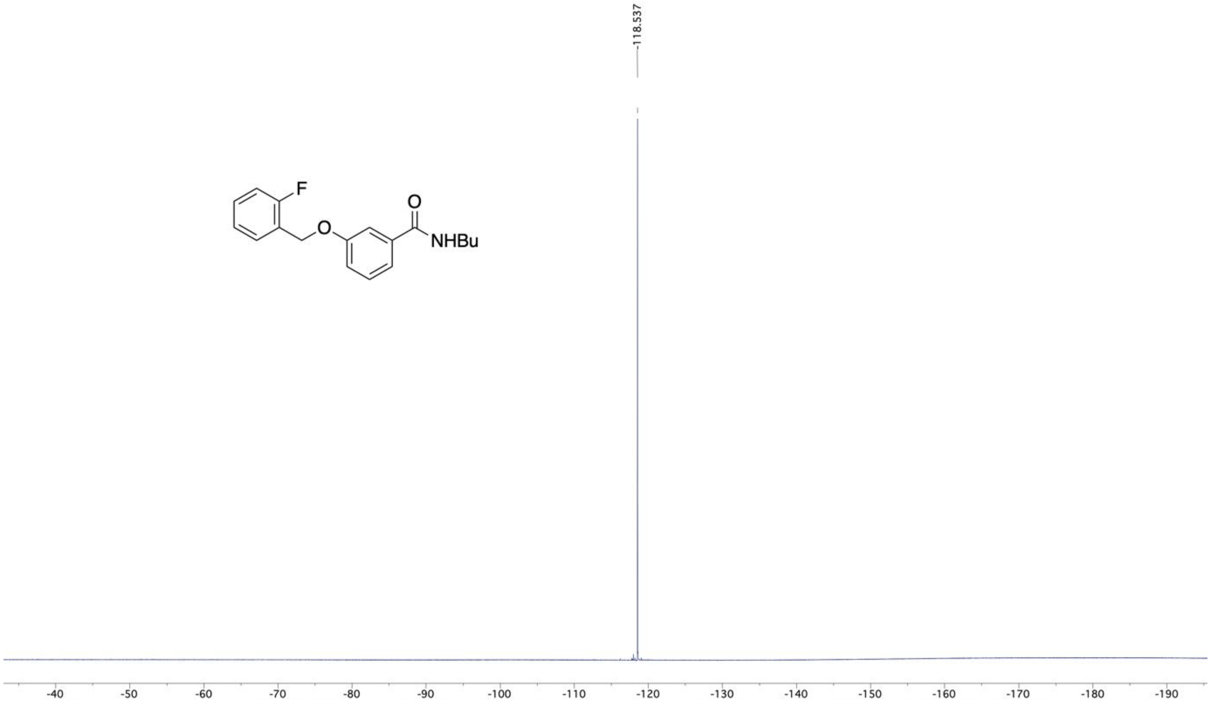

N-butyl-3-((4-fluorobenzyl)oxy)benzamide **37j**

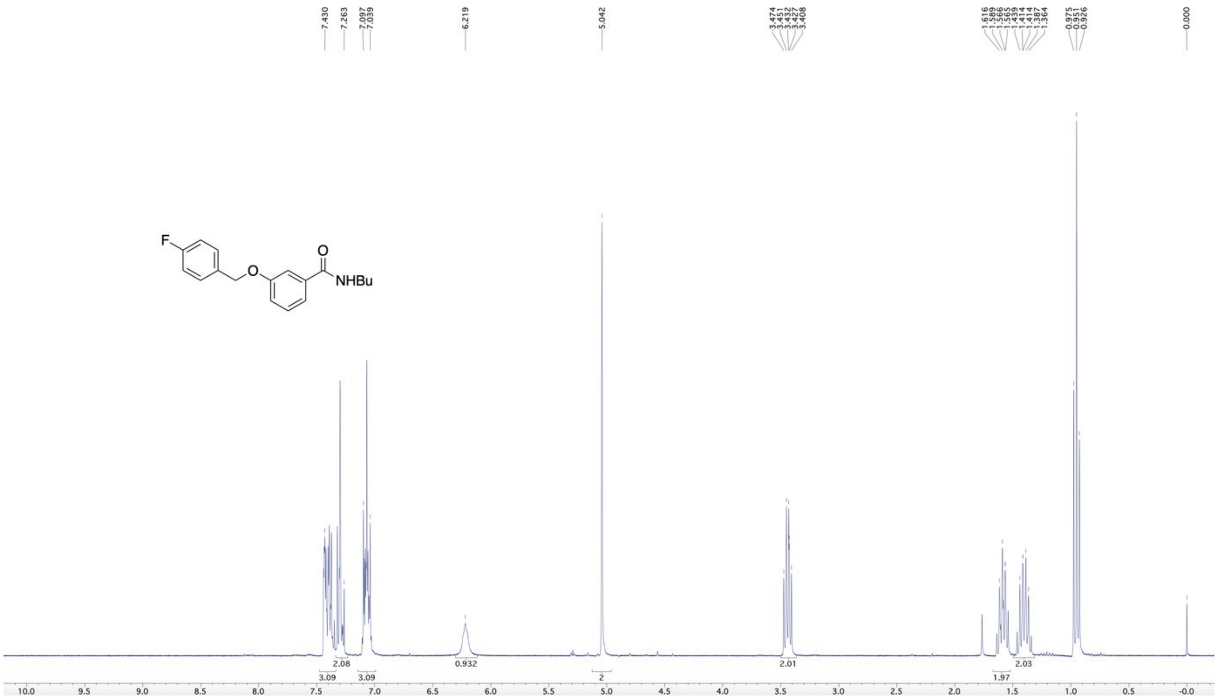

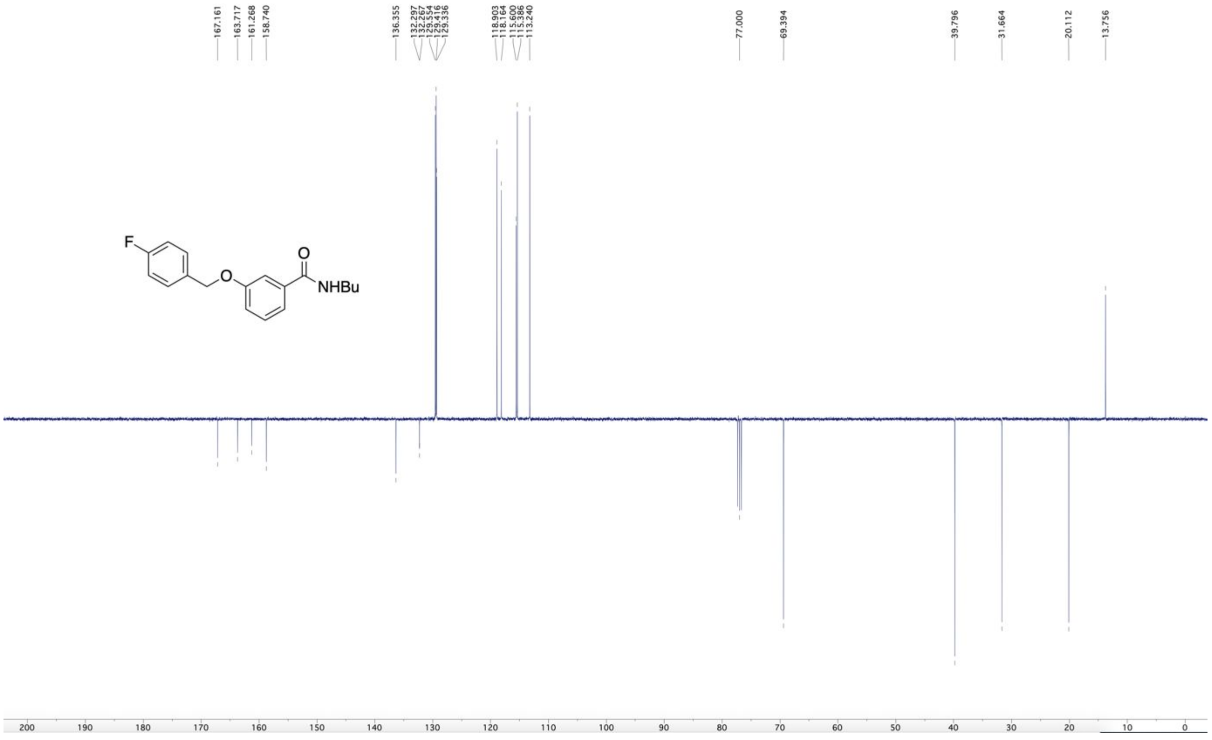

167.161
163.717
161.268
158.740
136.355
132.297
132.267
129.554
129.416
129.336
118.903
118.164
115.600
115.386
113.240
77.000
69.394
39.796
31.664
20.112
13.756
F
O
O
NHBu
200
190
180
170
160
150
140
130
120
110
100
90
80
70
60
50
40
30
20
10
0

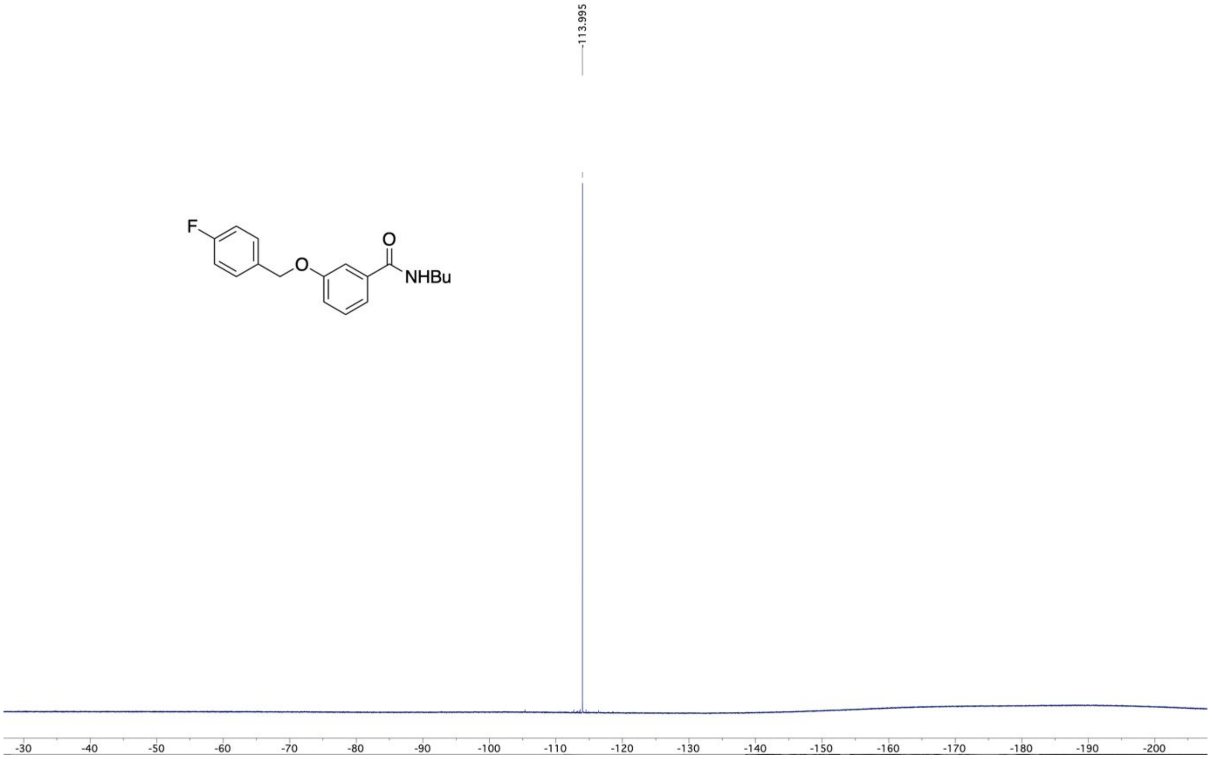

-113.995
F
O
O
NHBu
-30
-40
-50
-60
-70
-80
-90
-100
-110
-120
-130
-140
-150
-160
-170
-180
-190
-200

N-butyl-3-(1-phenylethoxy)benzamide **37k**

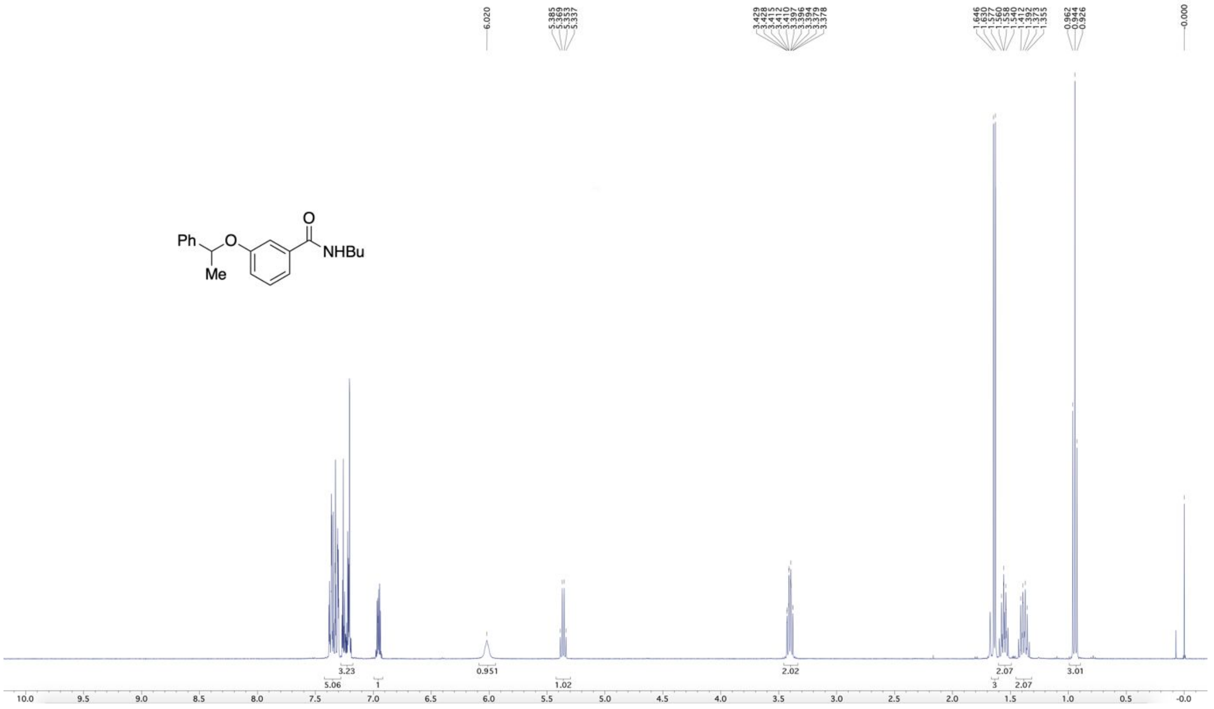

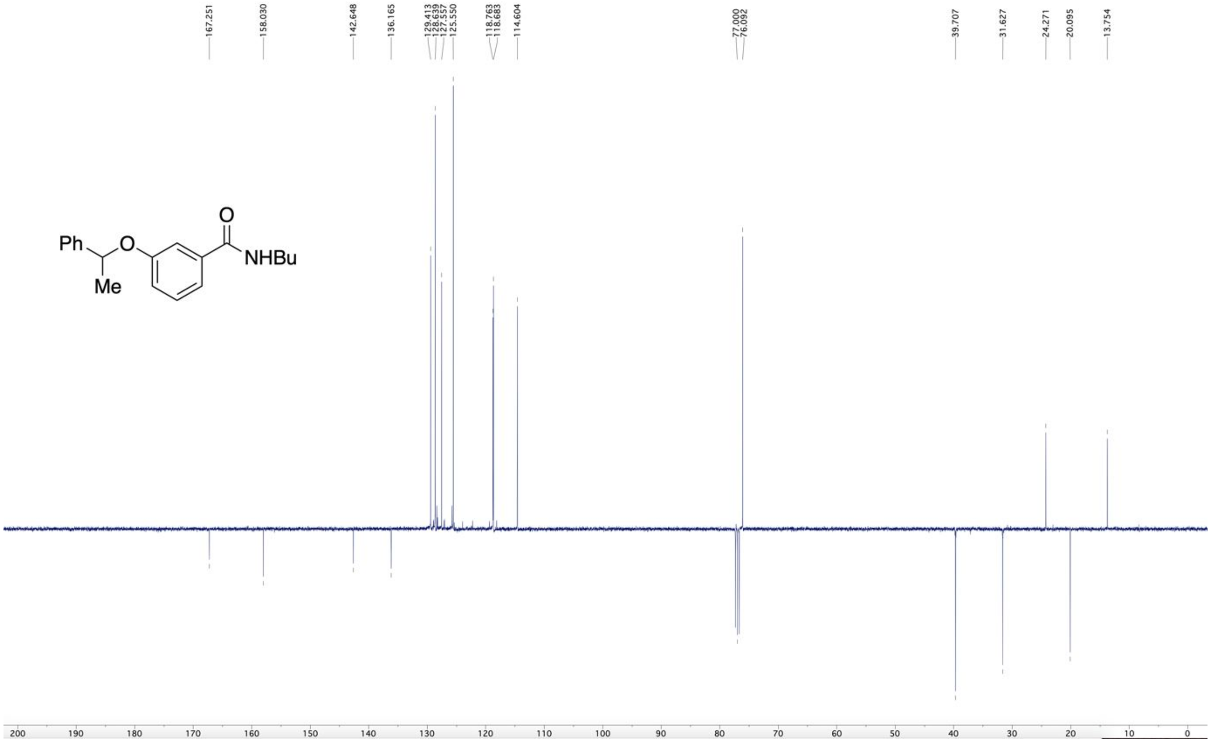

N-butyl-3-((3-methylbut-2-en-1-yl)oxy)benzamide **37l**

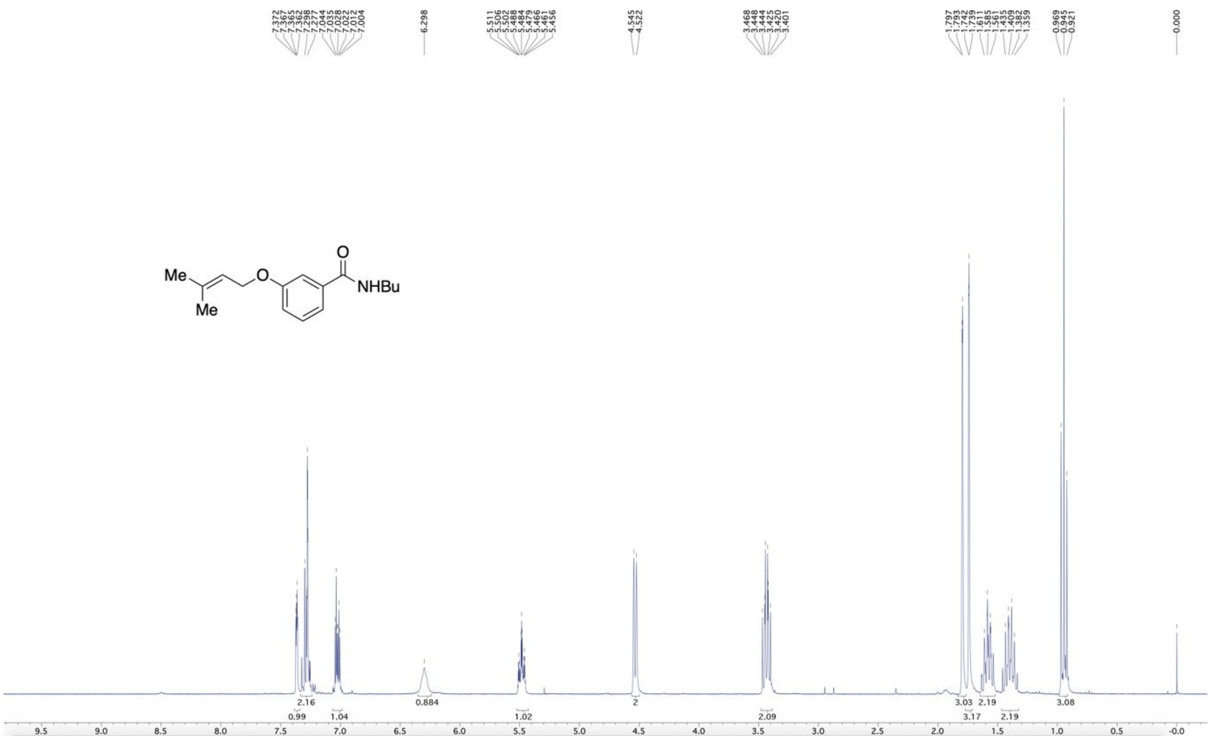

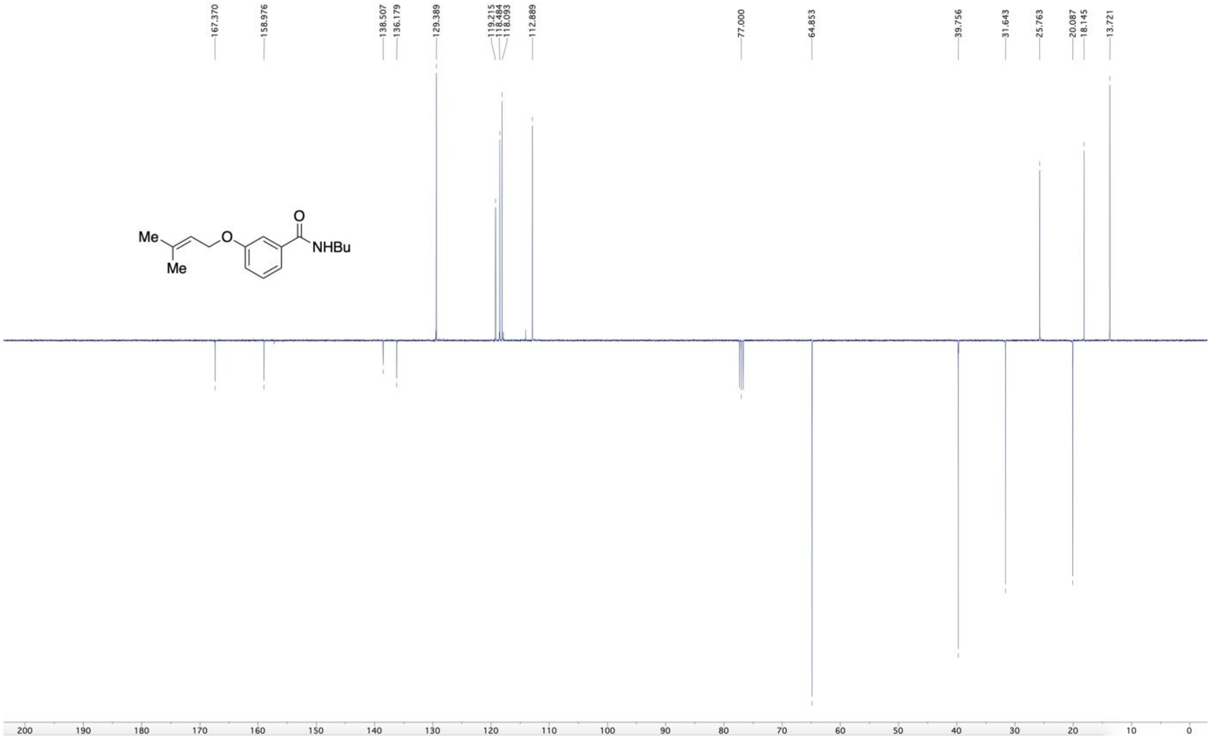

N-butyl-3-(thiophen-2-ylmethoxy)benzamide **37m**

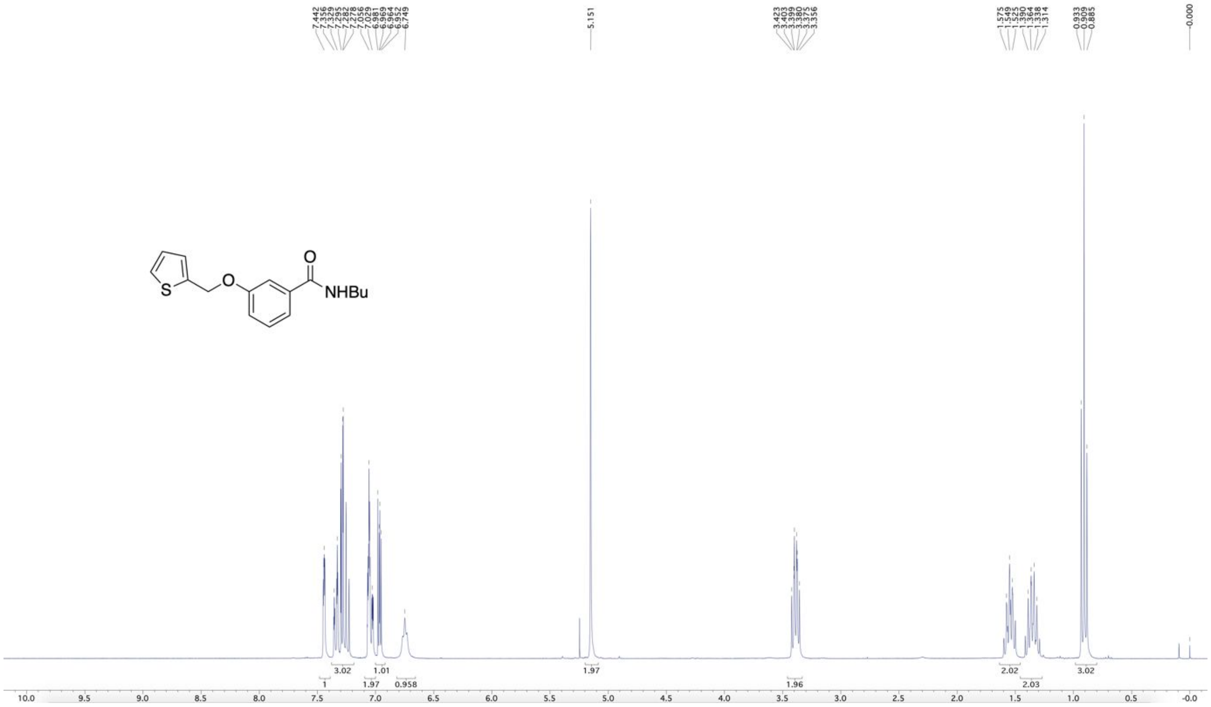

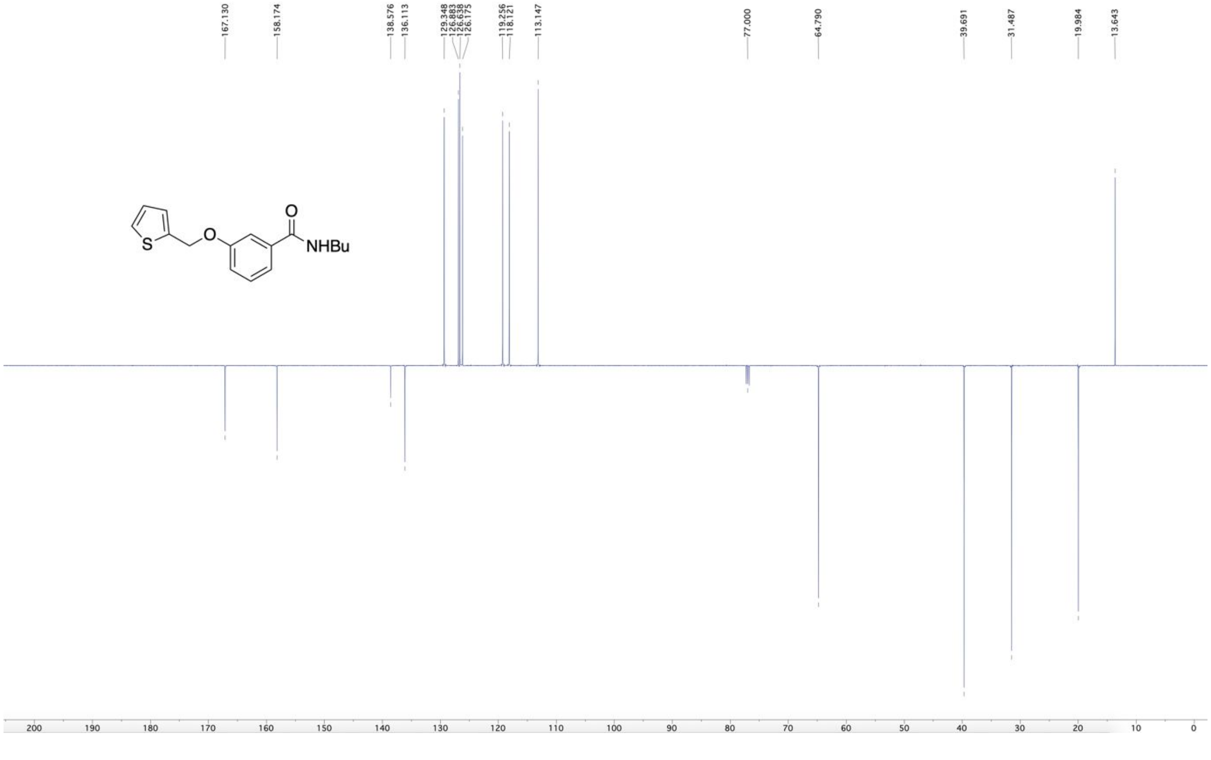

N-butyl-3-((2-methoxynaphthalen-1-yl)methoxy)benzamide **37n**

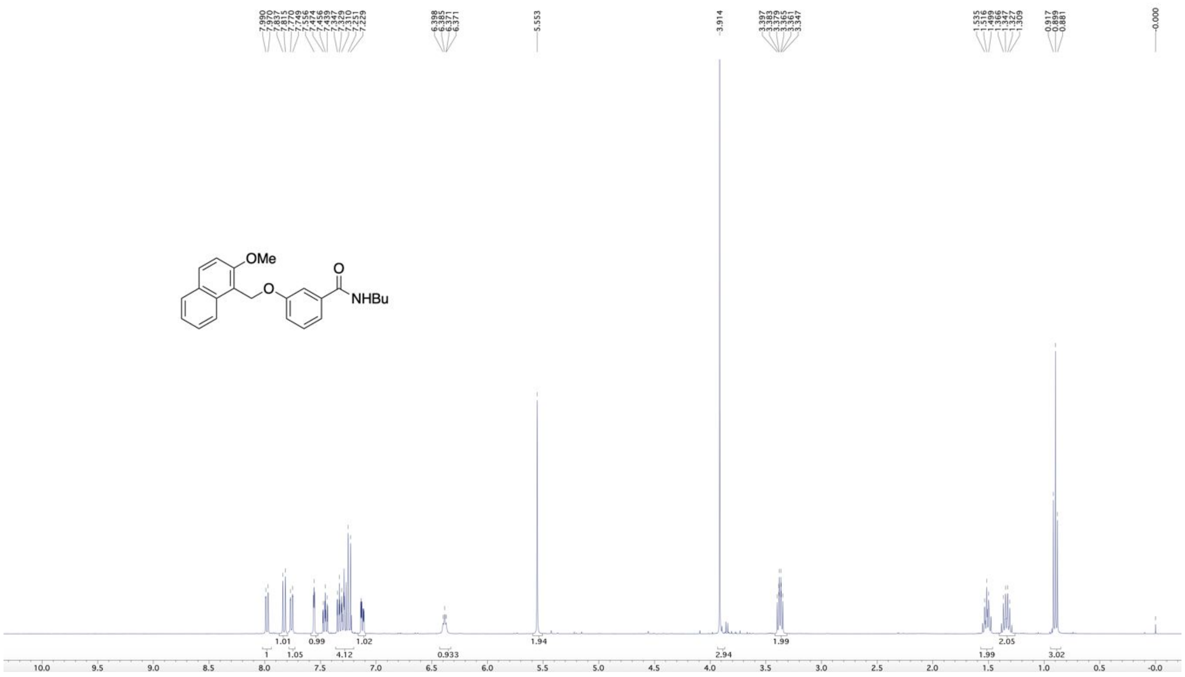

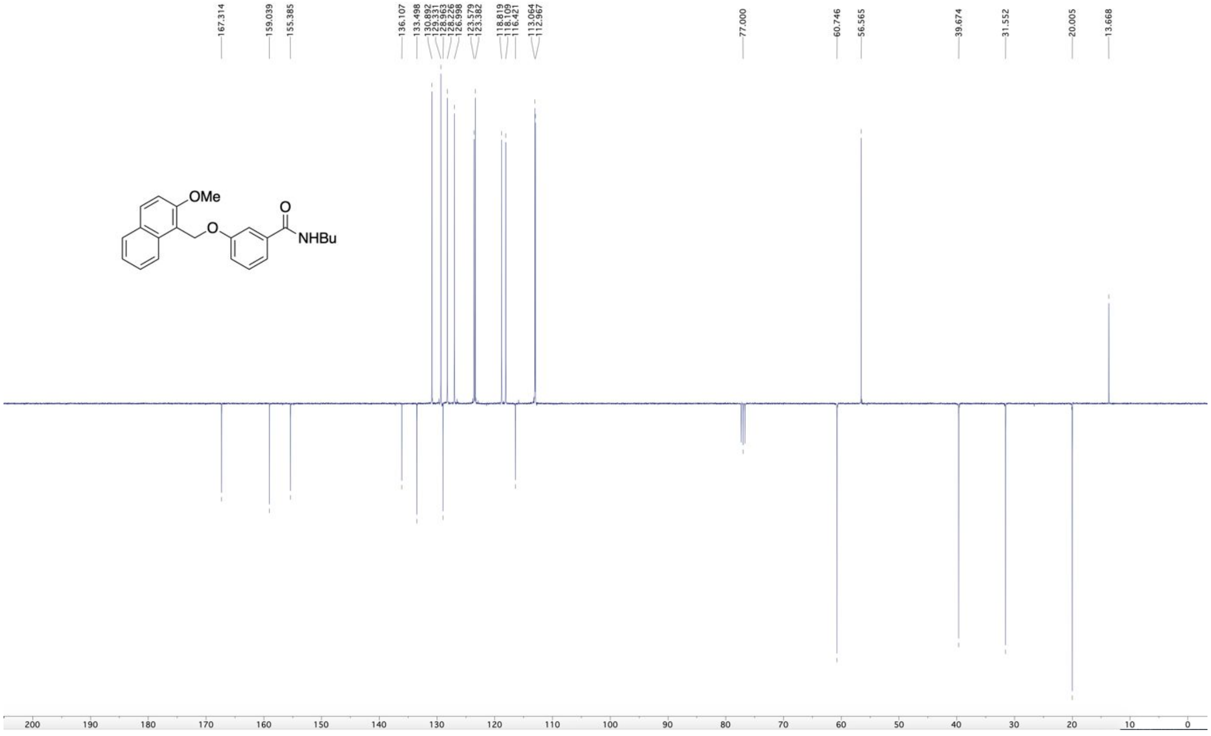

N-butyl-3-(hydroxy(phenyl)methyl)benzamide **38a**

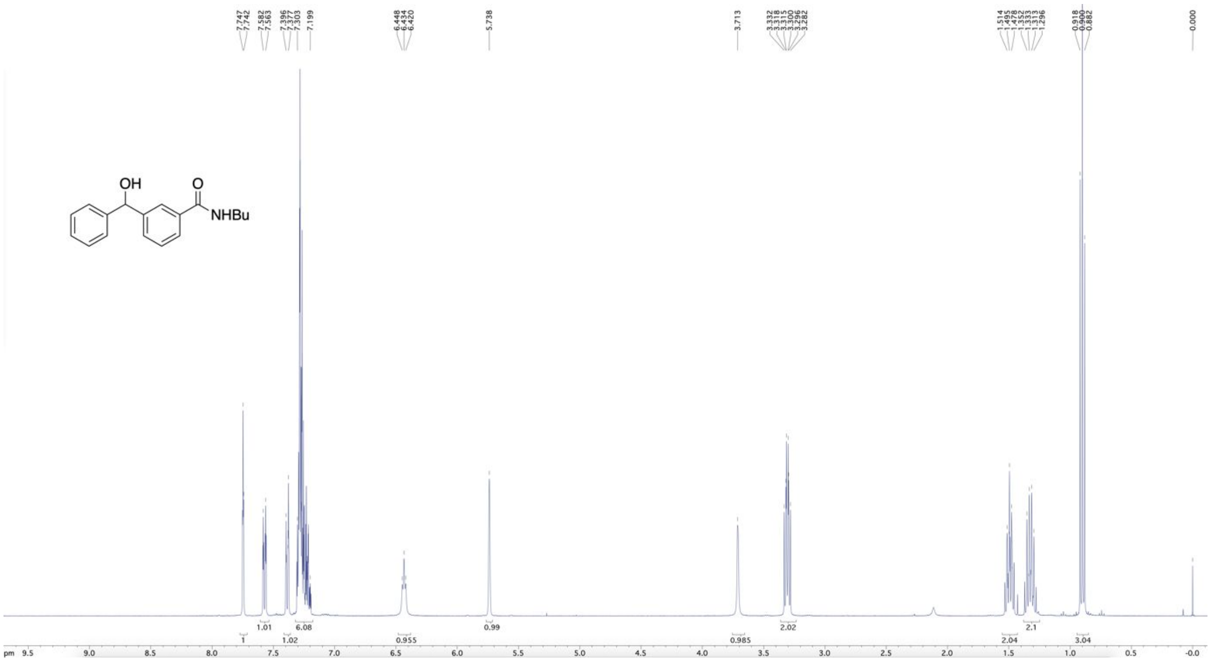

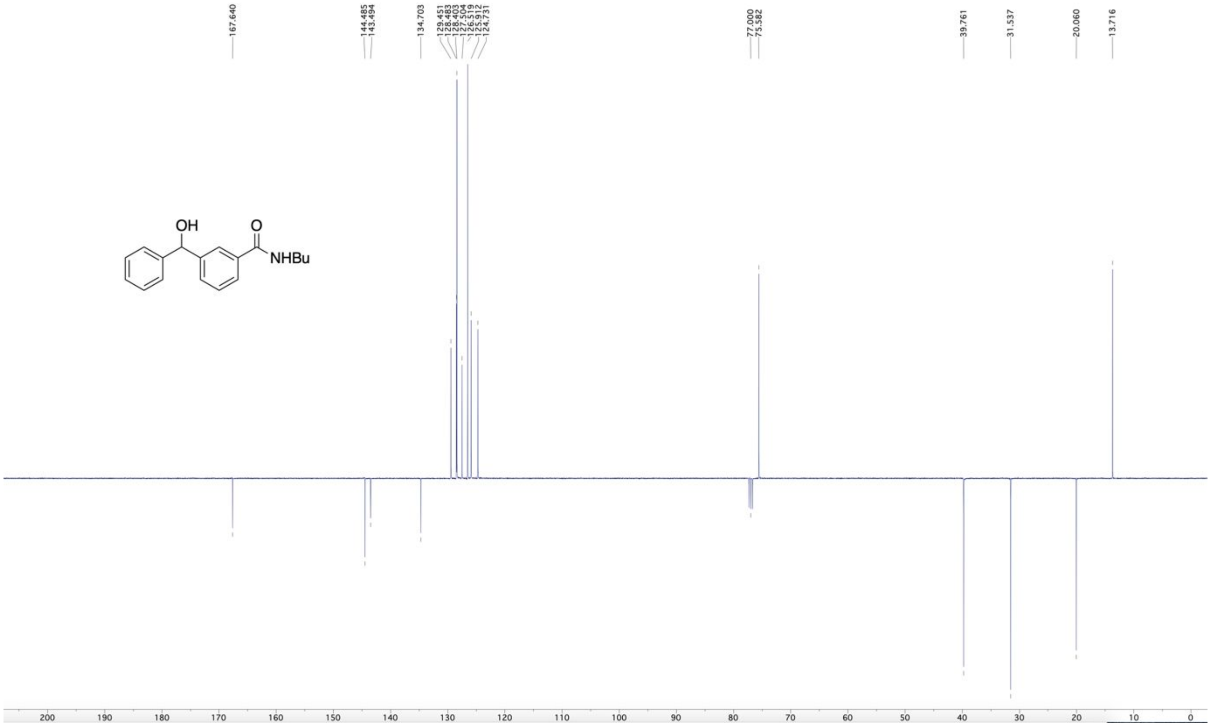

N-butyl-3-((4-(*tert*-butyl)phenyl)(hydroxy)methyl)benzamide **38b**

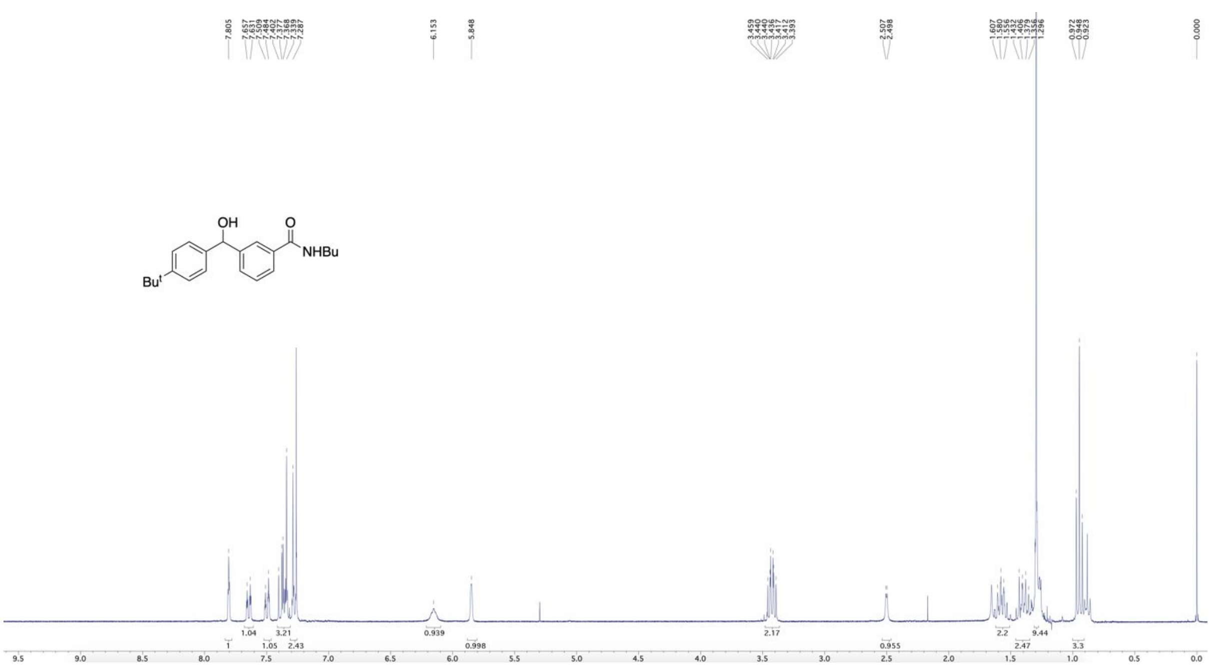

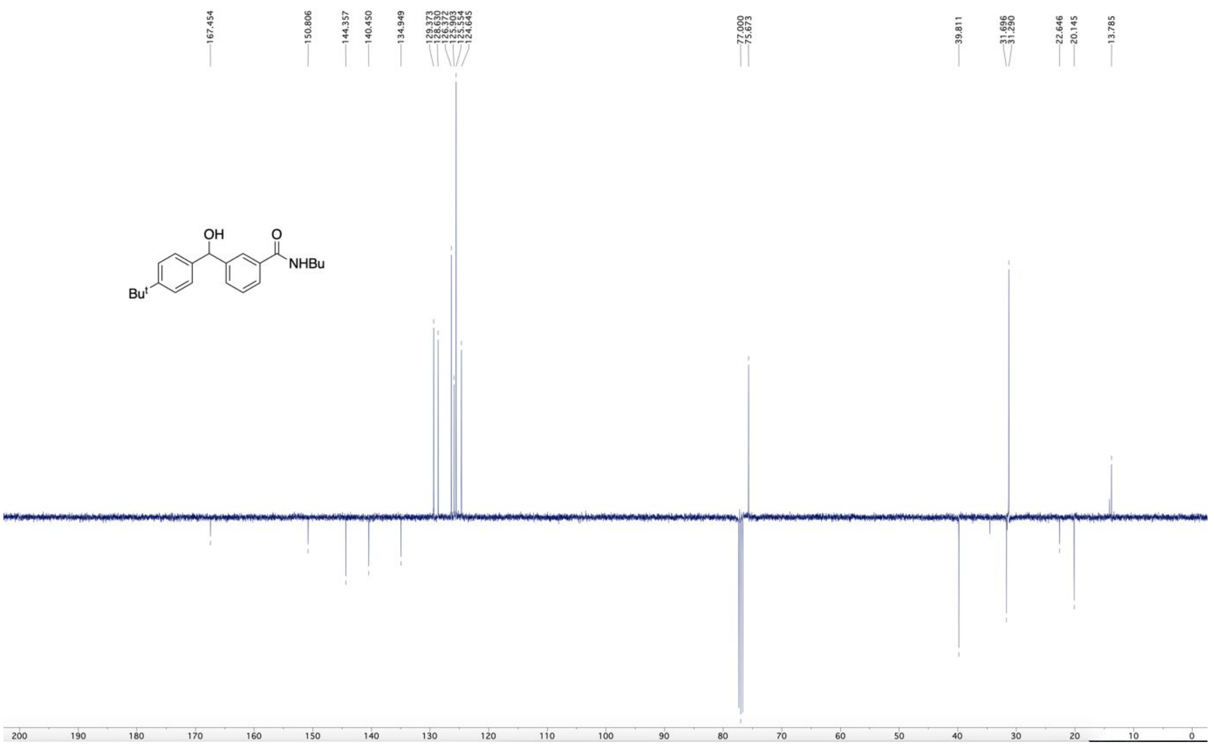

N-butyl-3-(hydroxy(*o*-tolyl)methyl)benzamide **38c**

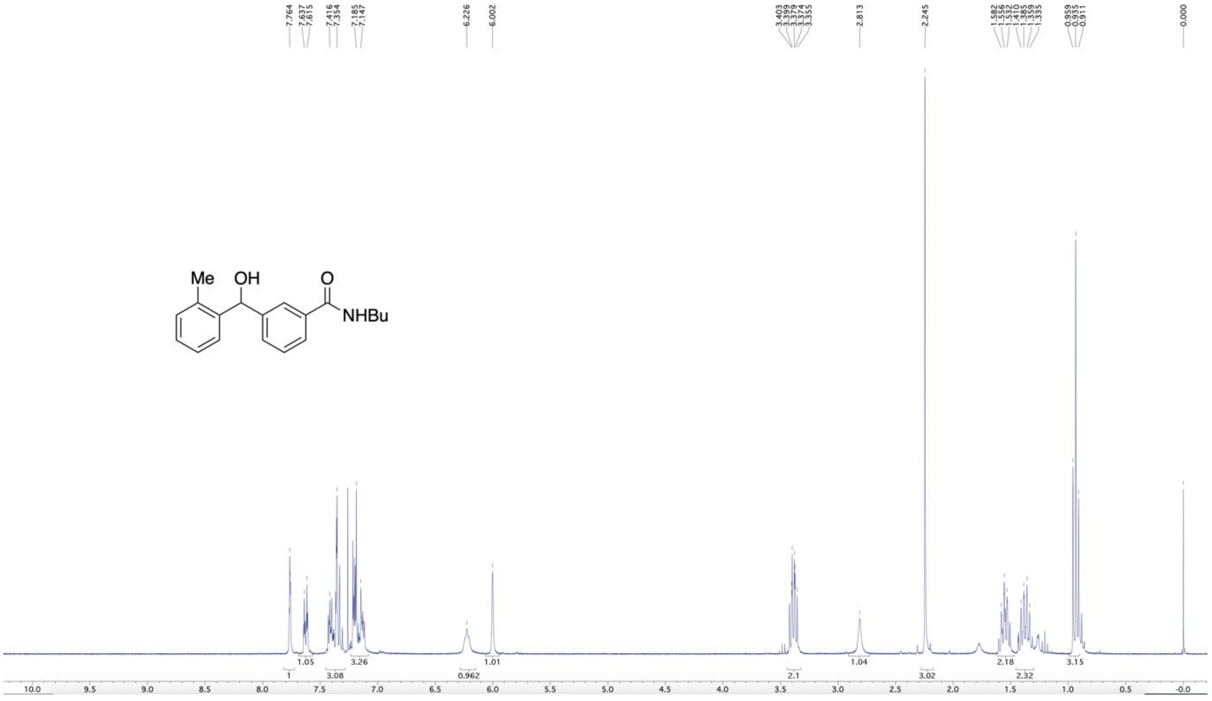

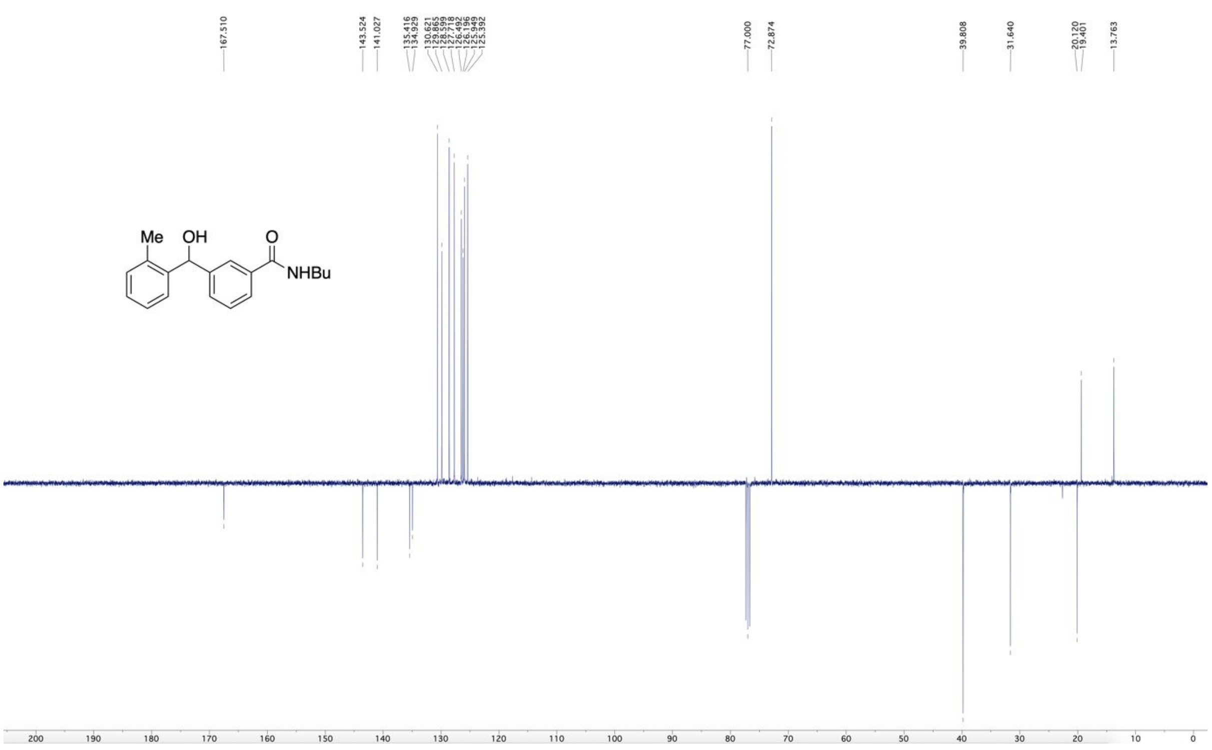

N-butyl-3-(hydroxy(2-methoxyphenyl)methyl)benzamide **38g**

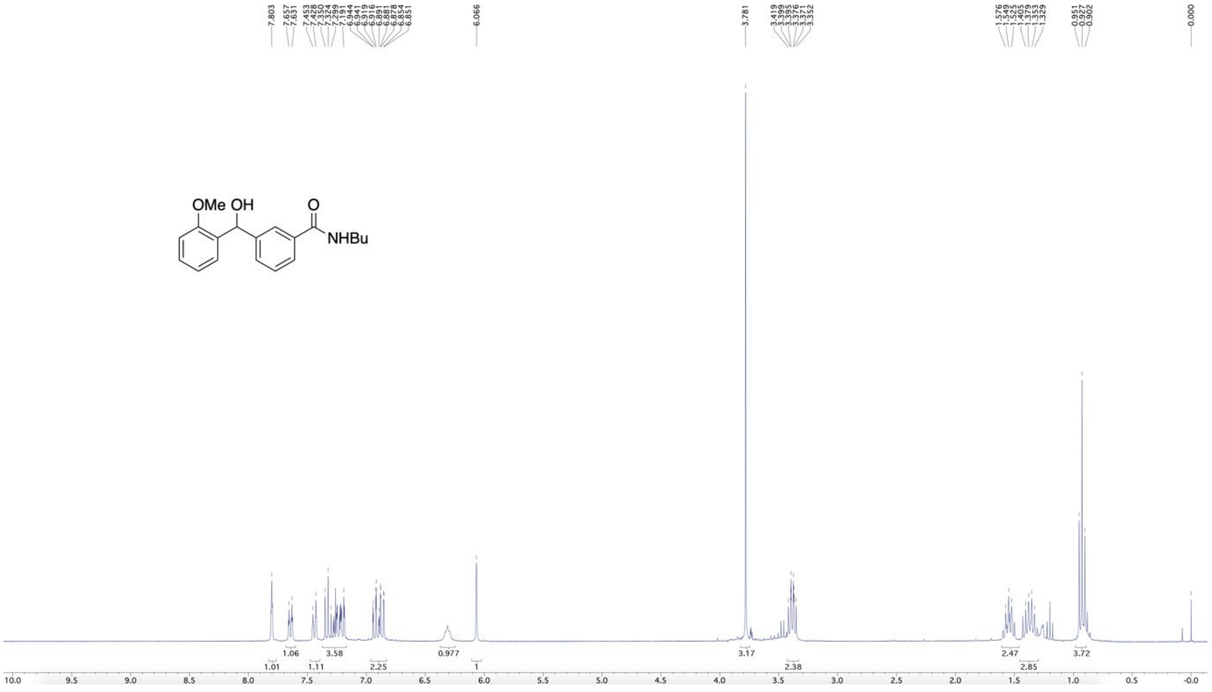

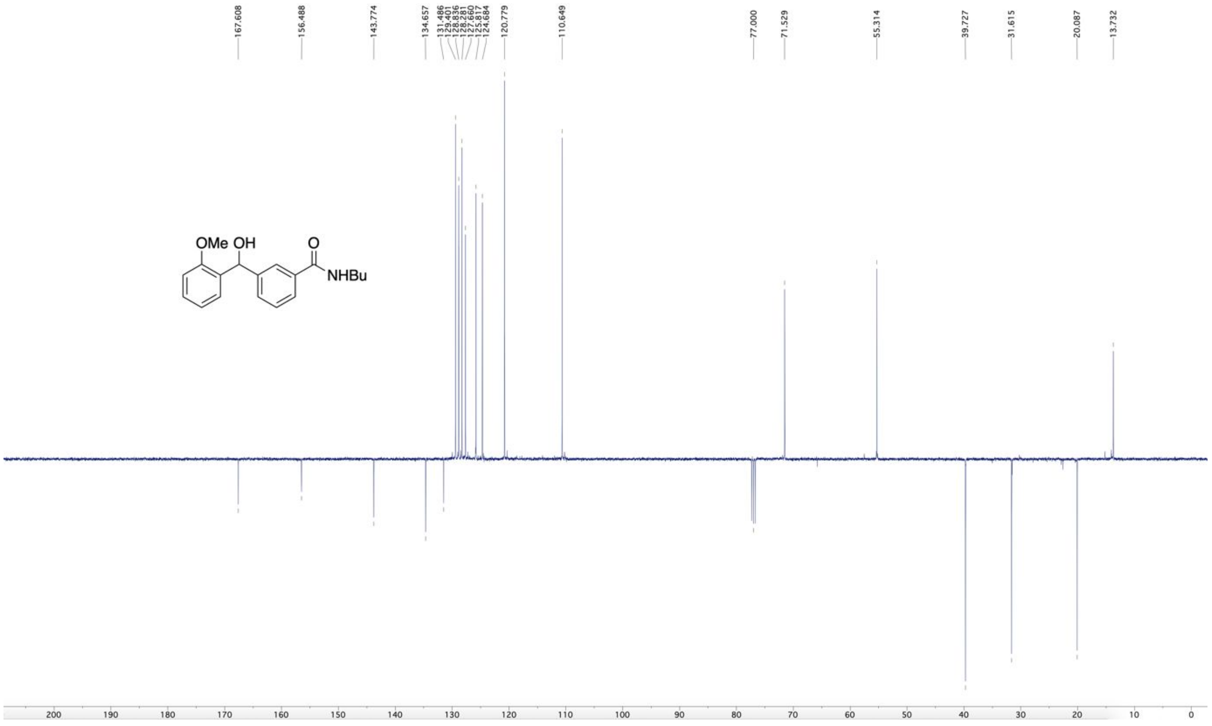

N-butyl-3-(hydroxy(4-methoxyphenyl)methyl)benzamide **38h**

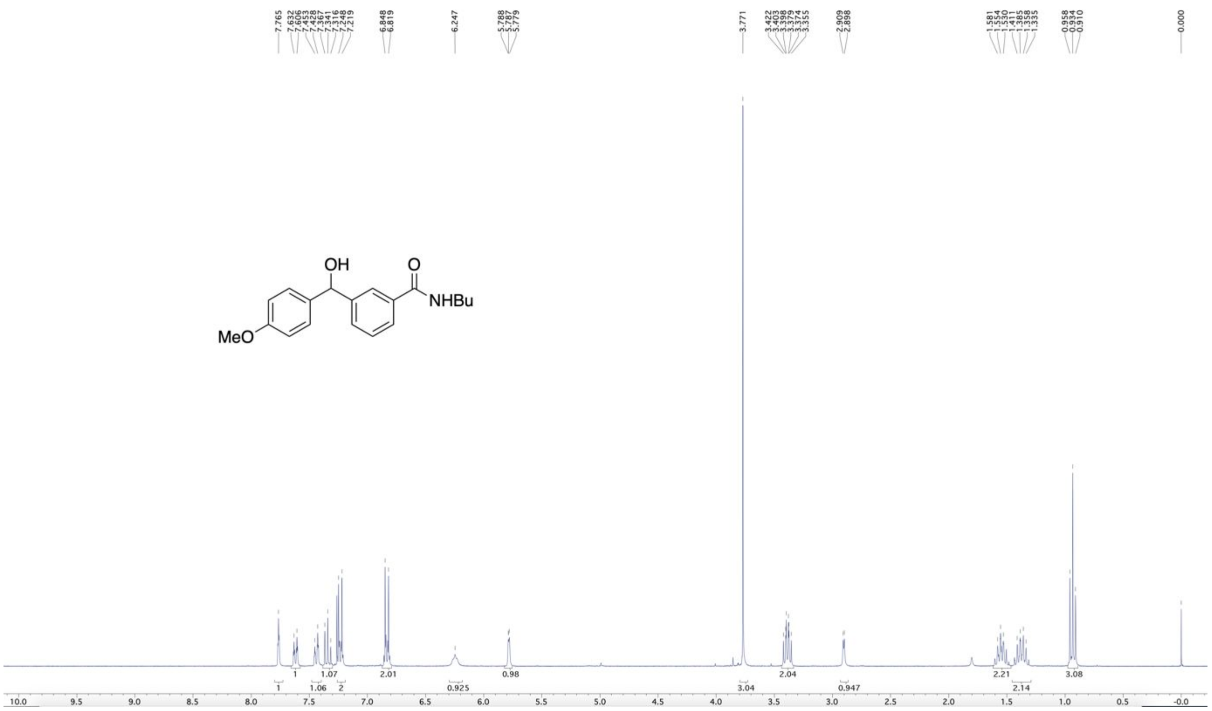

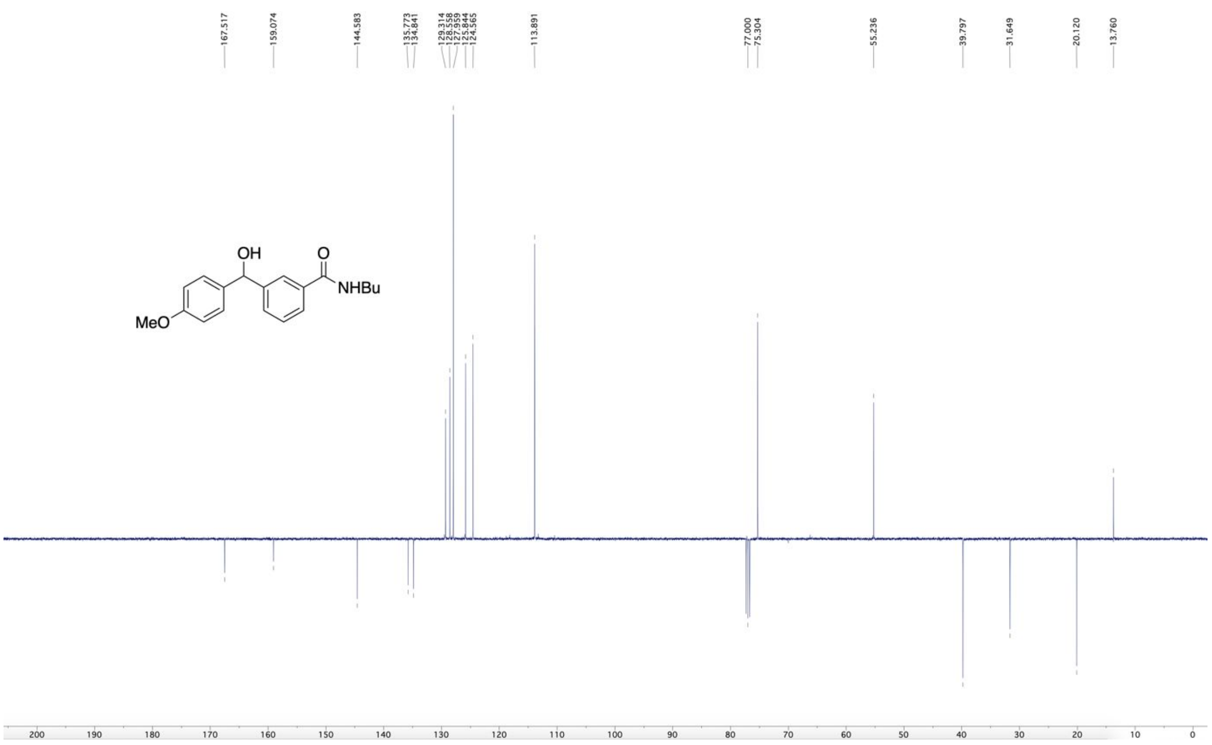

N-butyl-3-((4-fluorophenyl)(hydroxy)methyl)benzamide **38j**

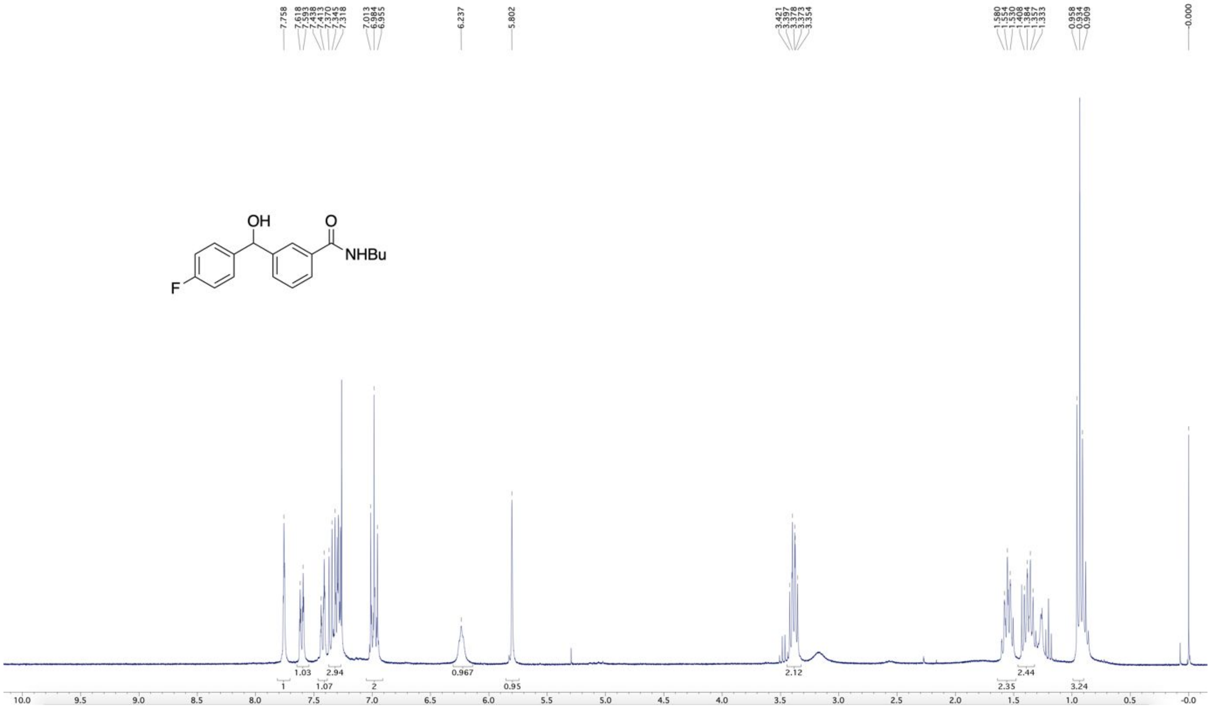

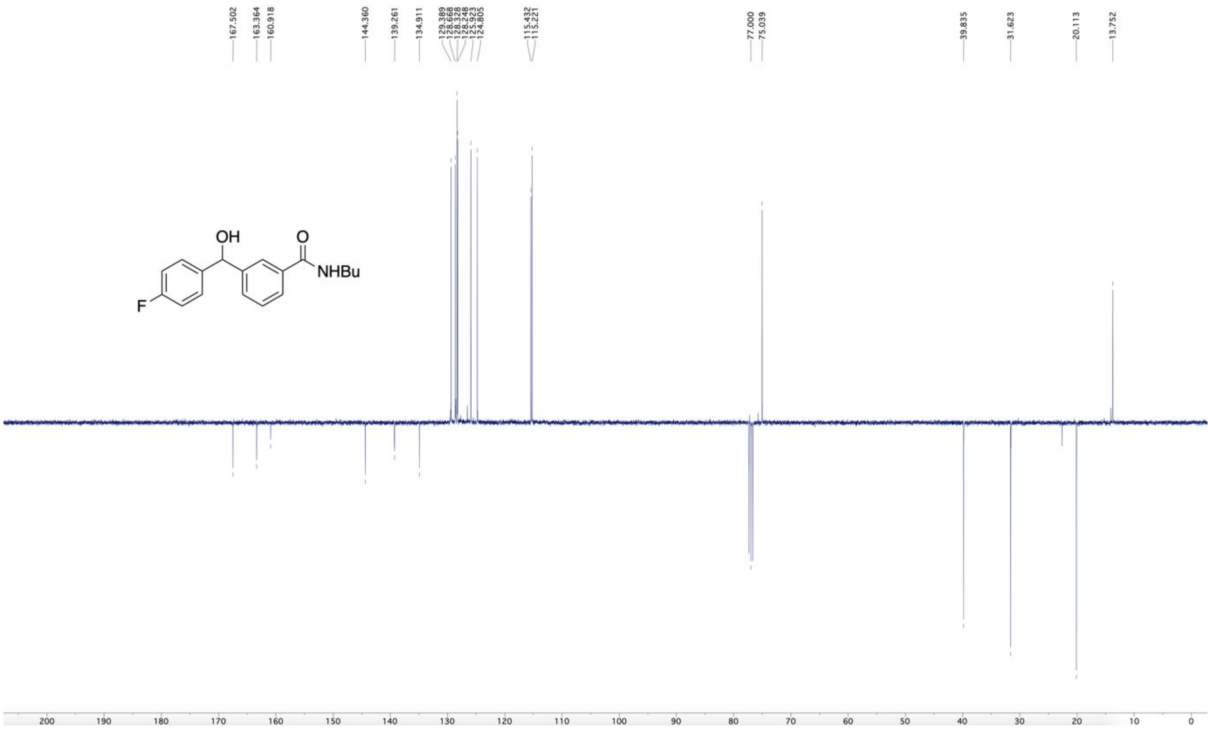

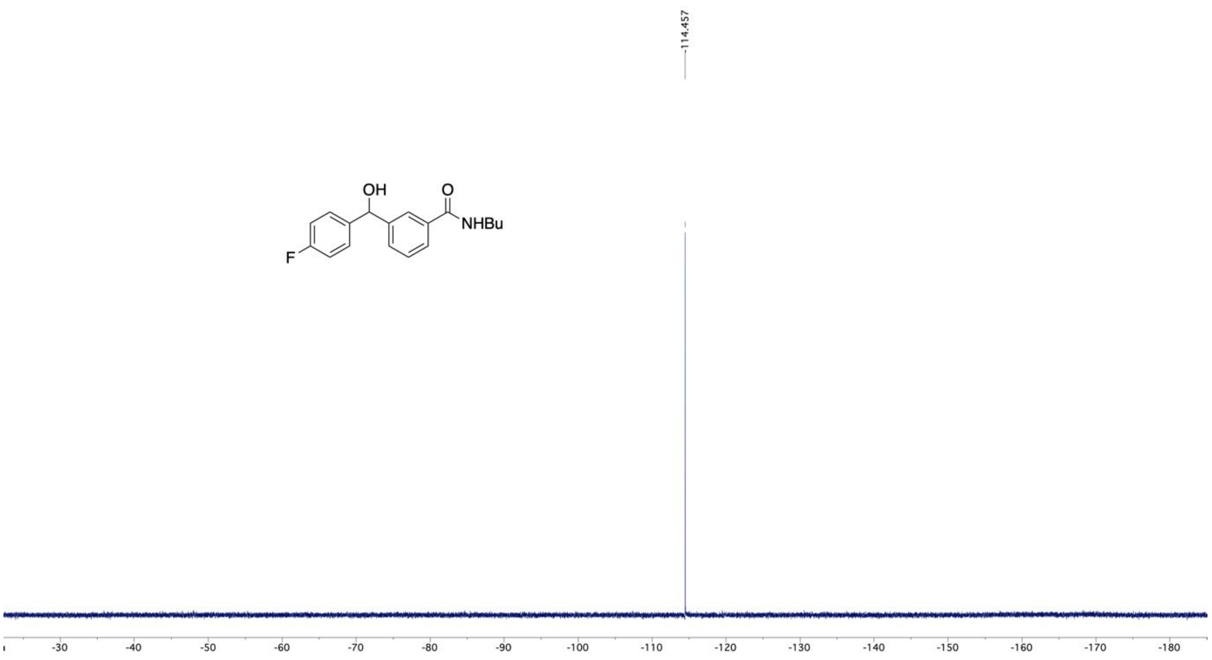

N-butyl-3-(hydroxy(thiophen-2-yl)methyl)benzamide **38m**

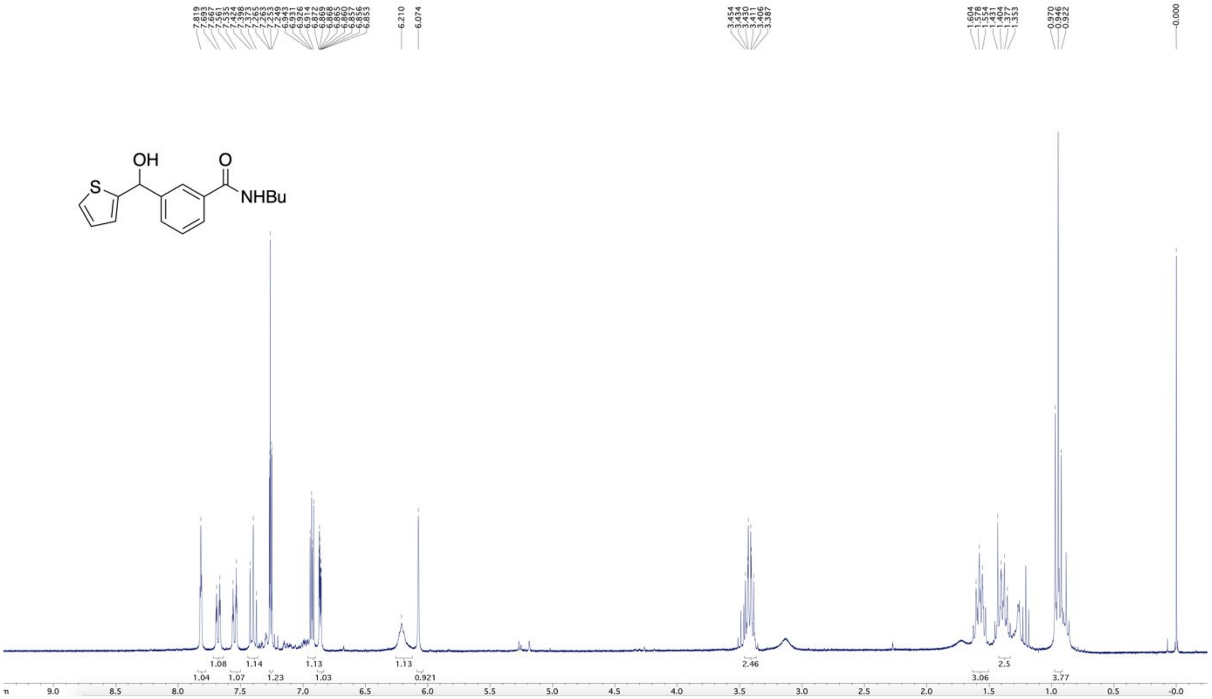

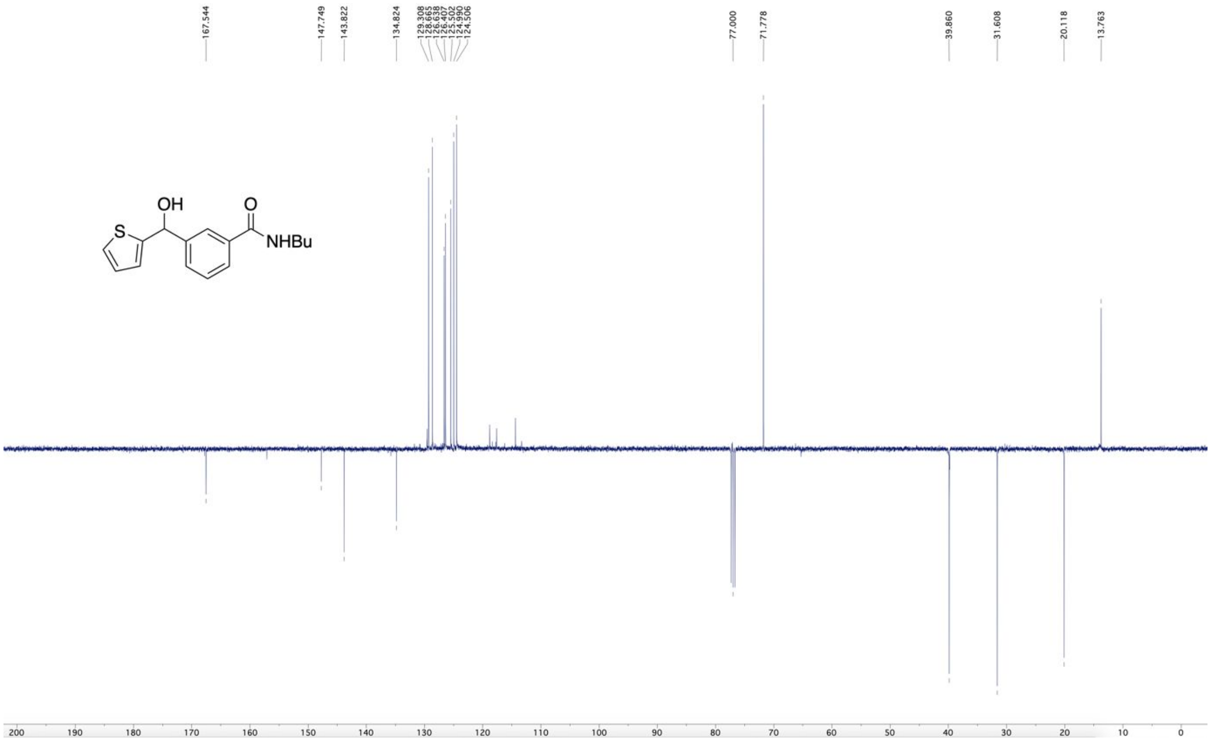

N-Butyl-2-hydroxy-5-nitrobenzamide **39**

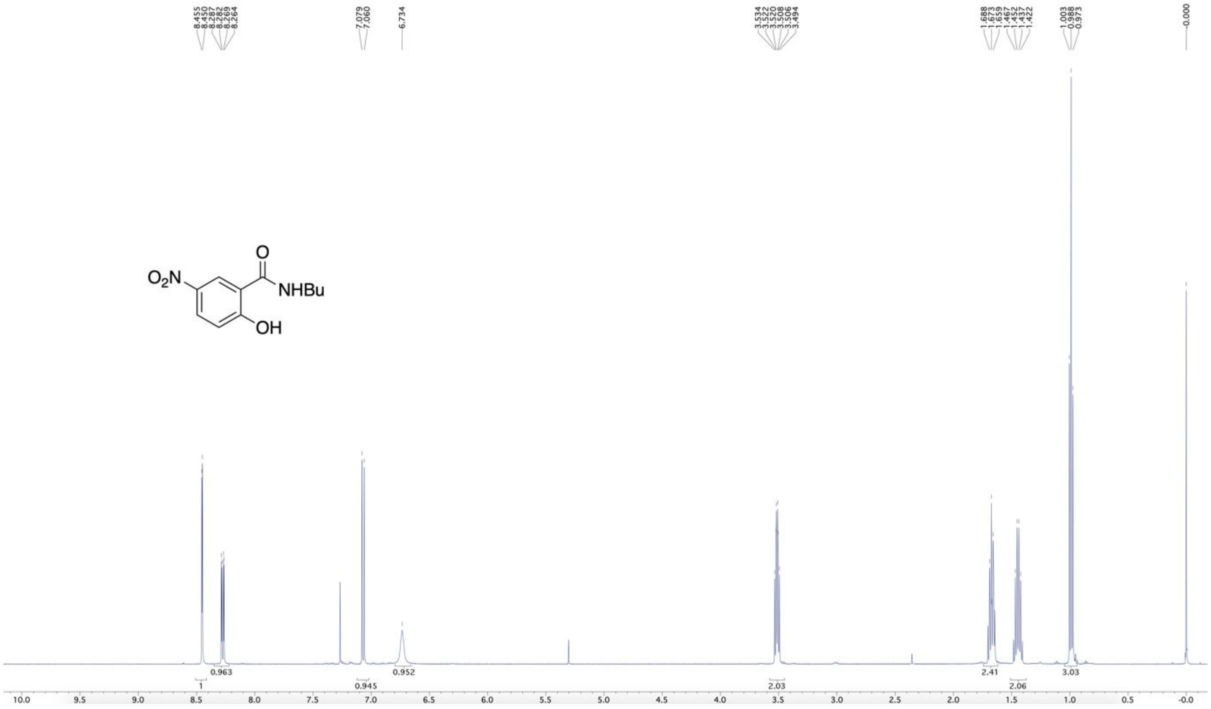

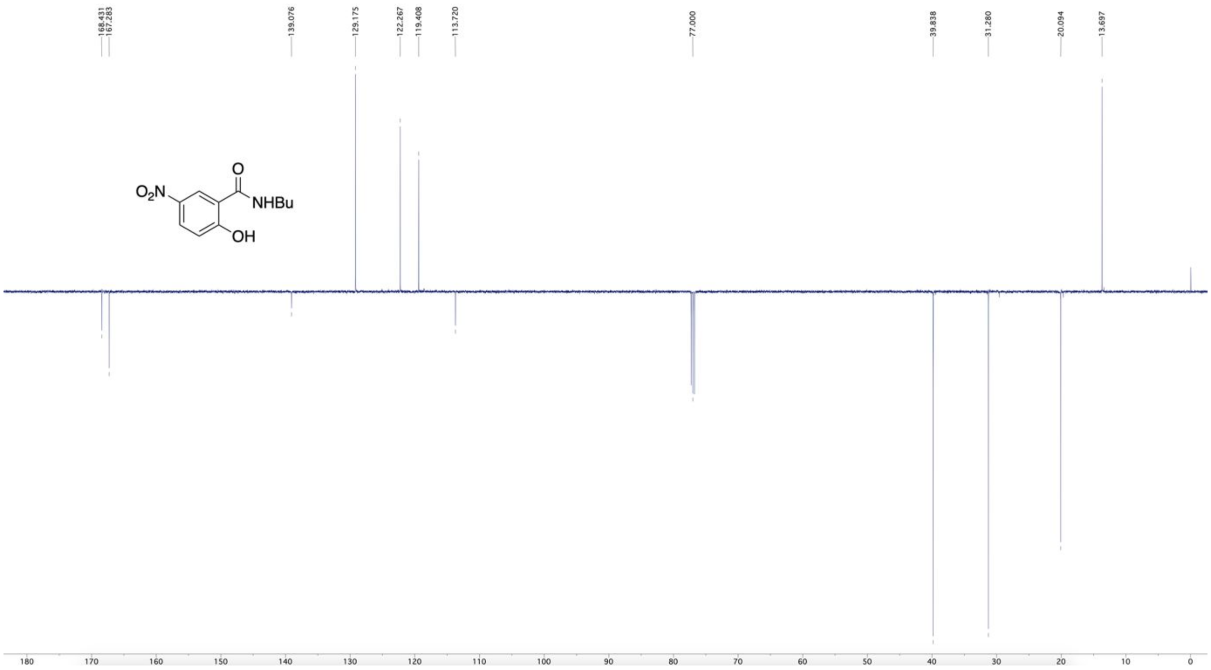

2-(Benzyloxy)-*N*-butyl-5-nitrobenzamide **40a**

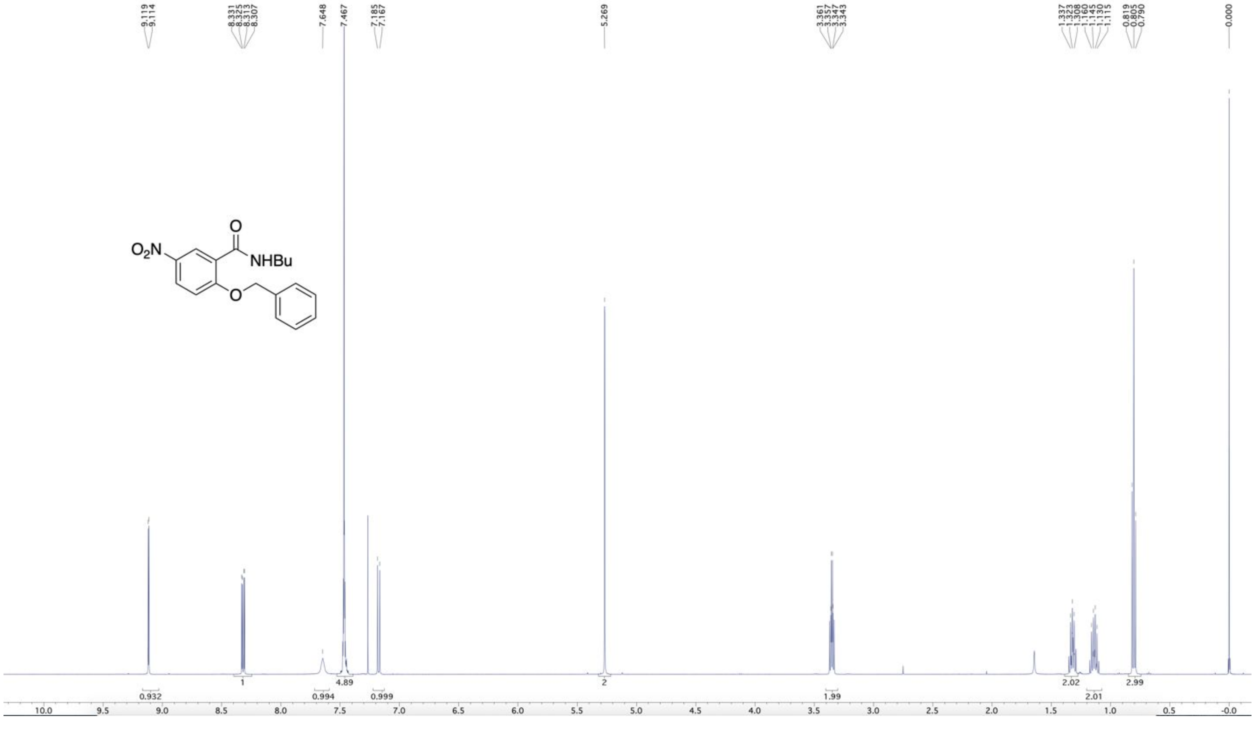

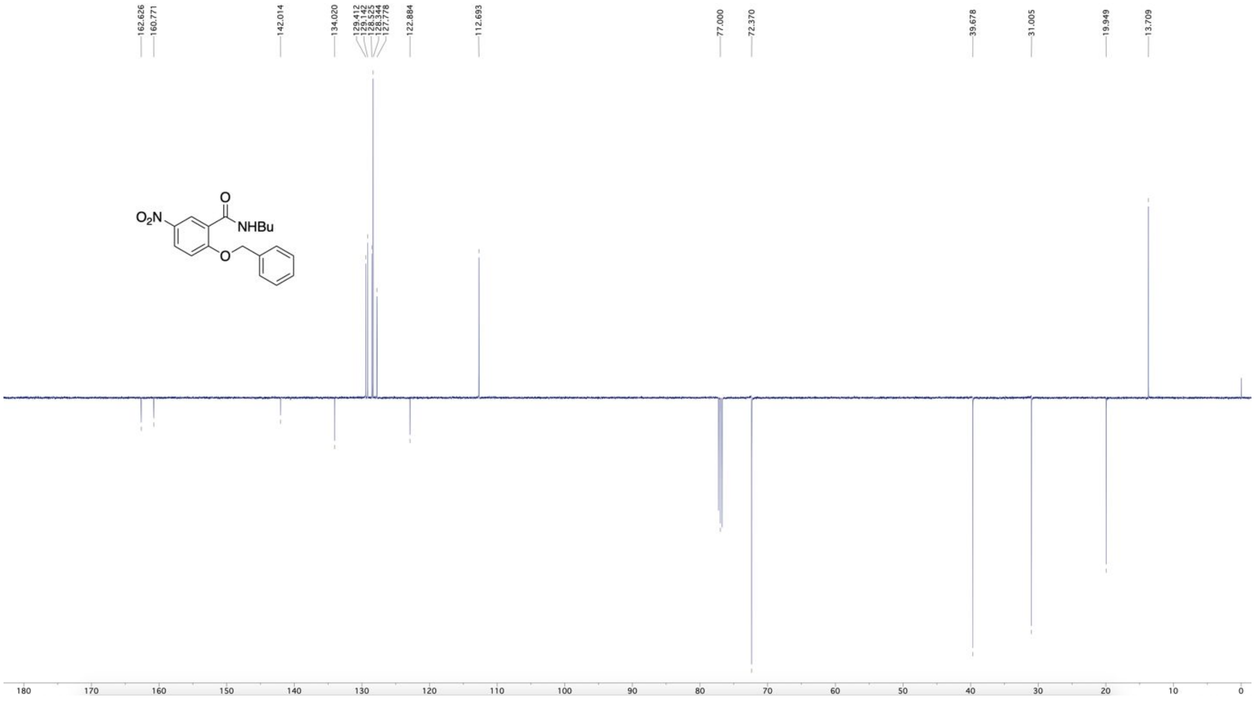

N-Butyl-5-nitro-2-((4-nitrobenzyl)oxy)benzamide **40b**

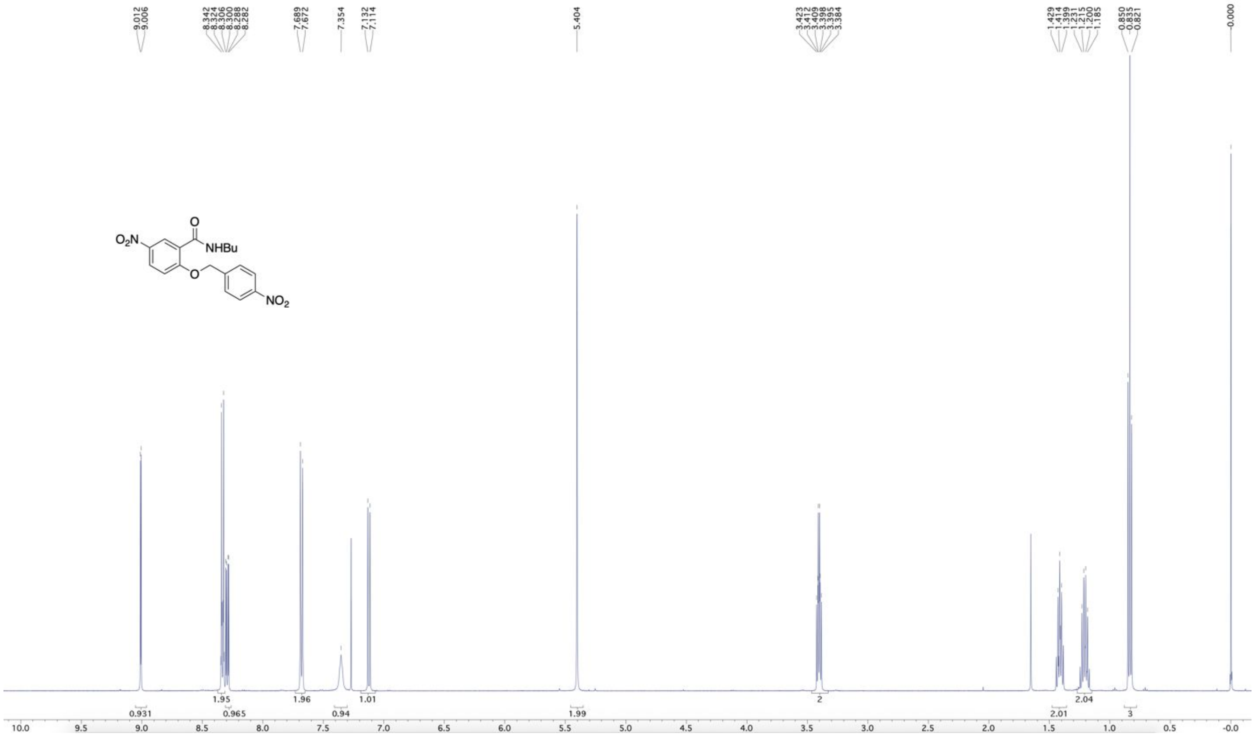

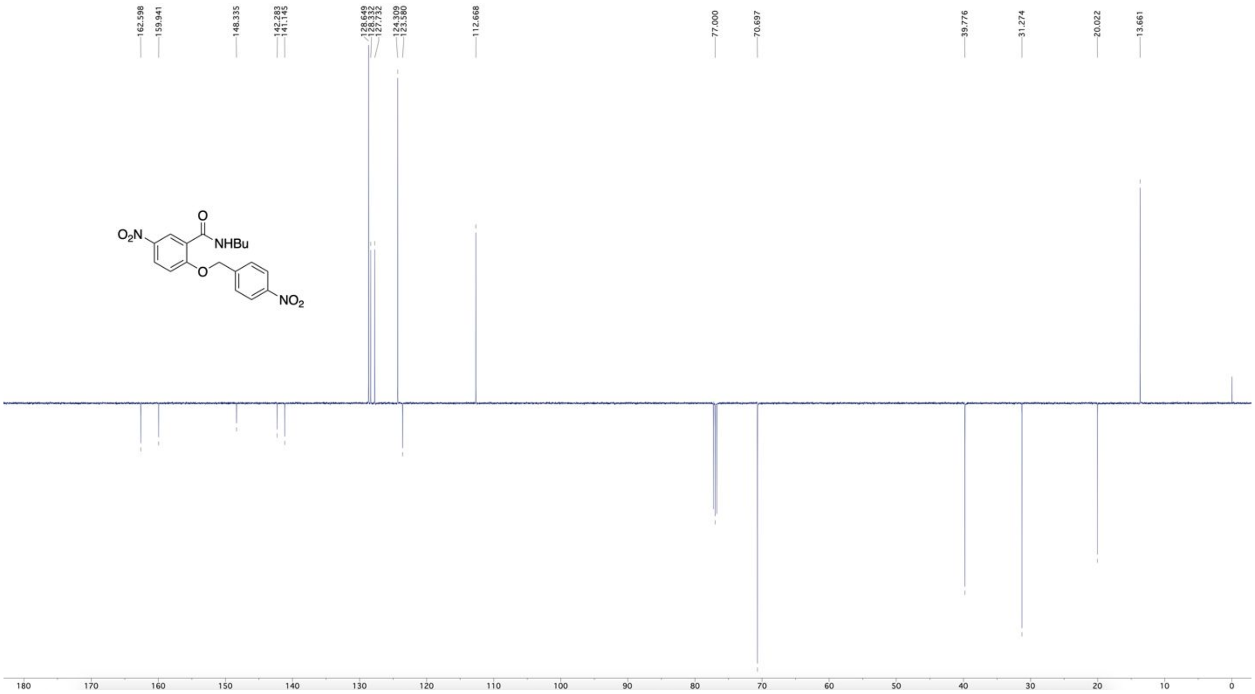

N-Butyl-2-((4-methoxybenzyl)oxy)-5-nitrobenzamide **40c**

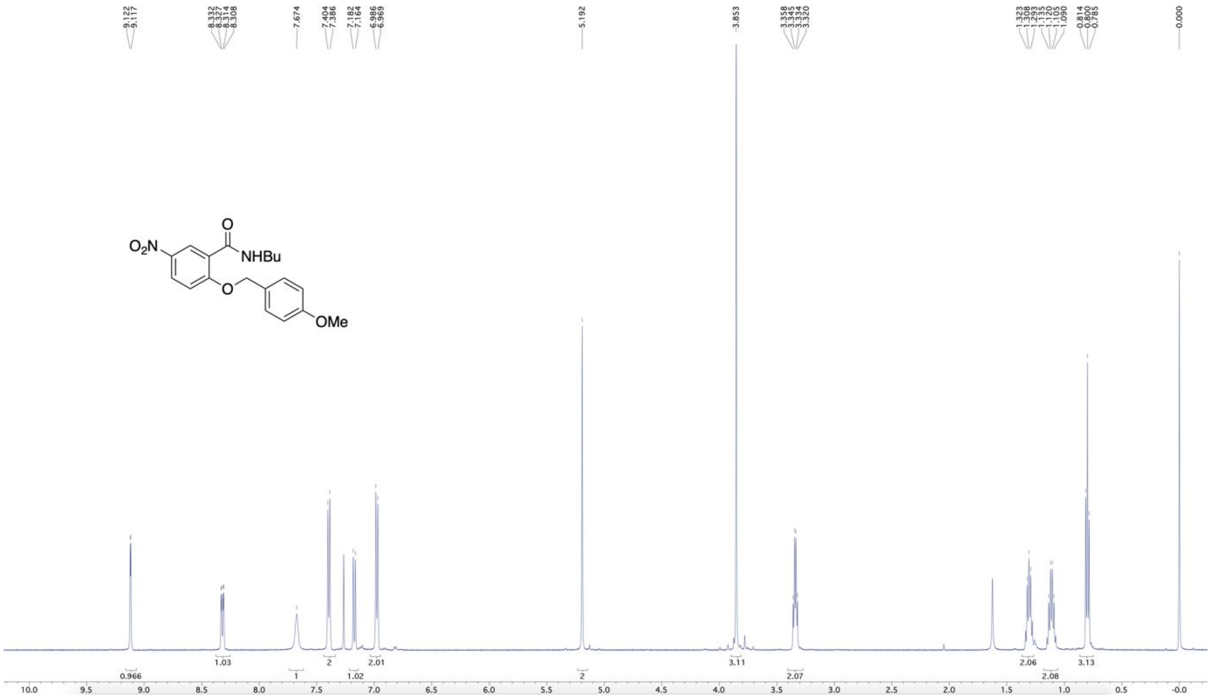

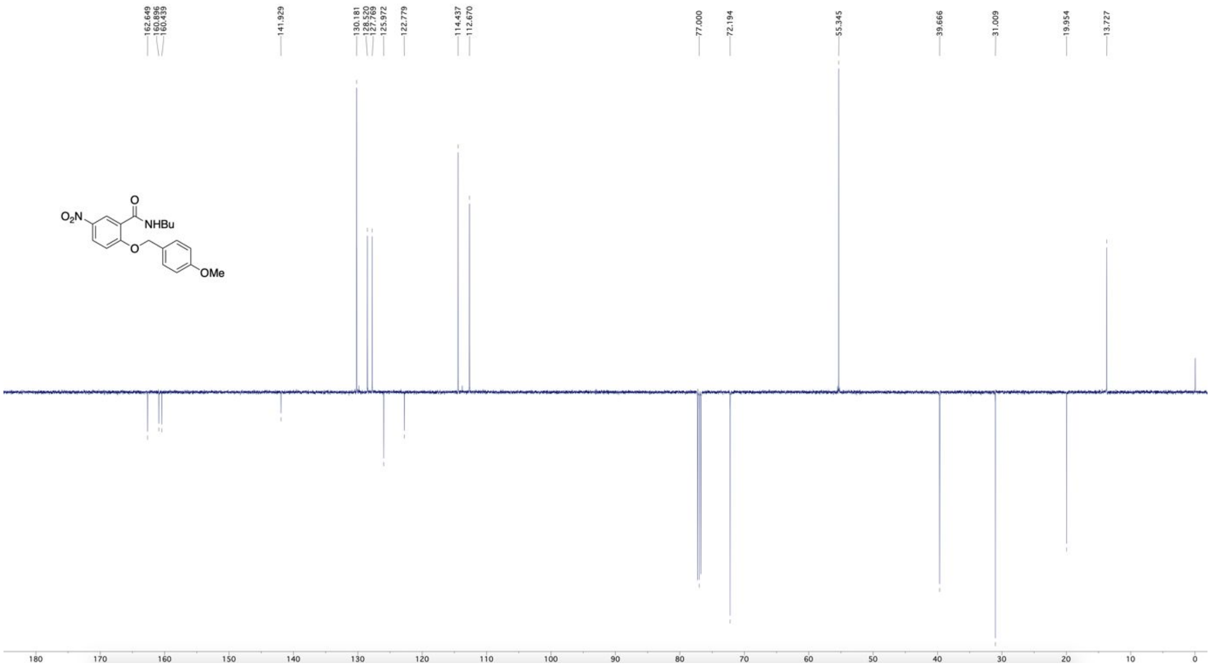

Methyl 5-(dimethylamino)-2-hydroxybenzoate **E**

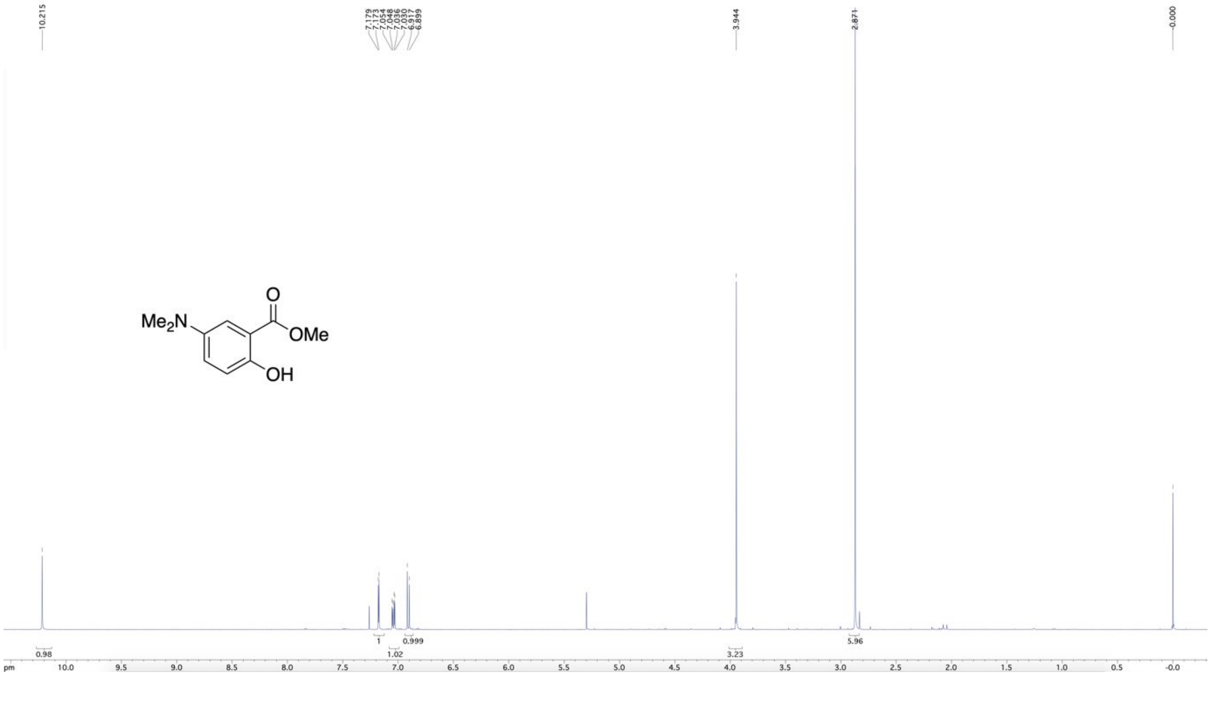

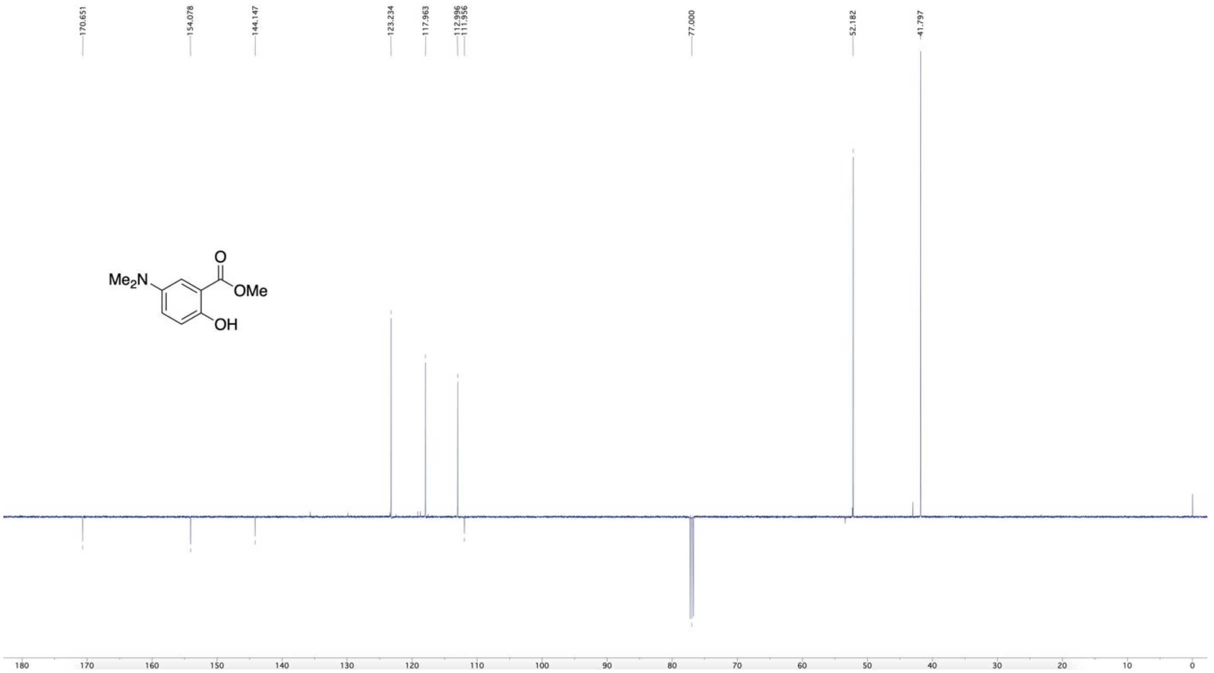

N-Butyl-5-(dimethylamino)-2-hydroxybenzamide **41**

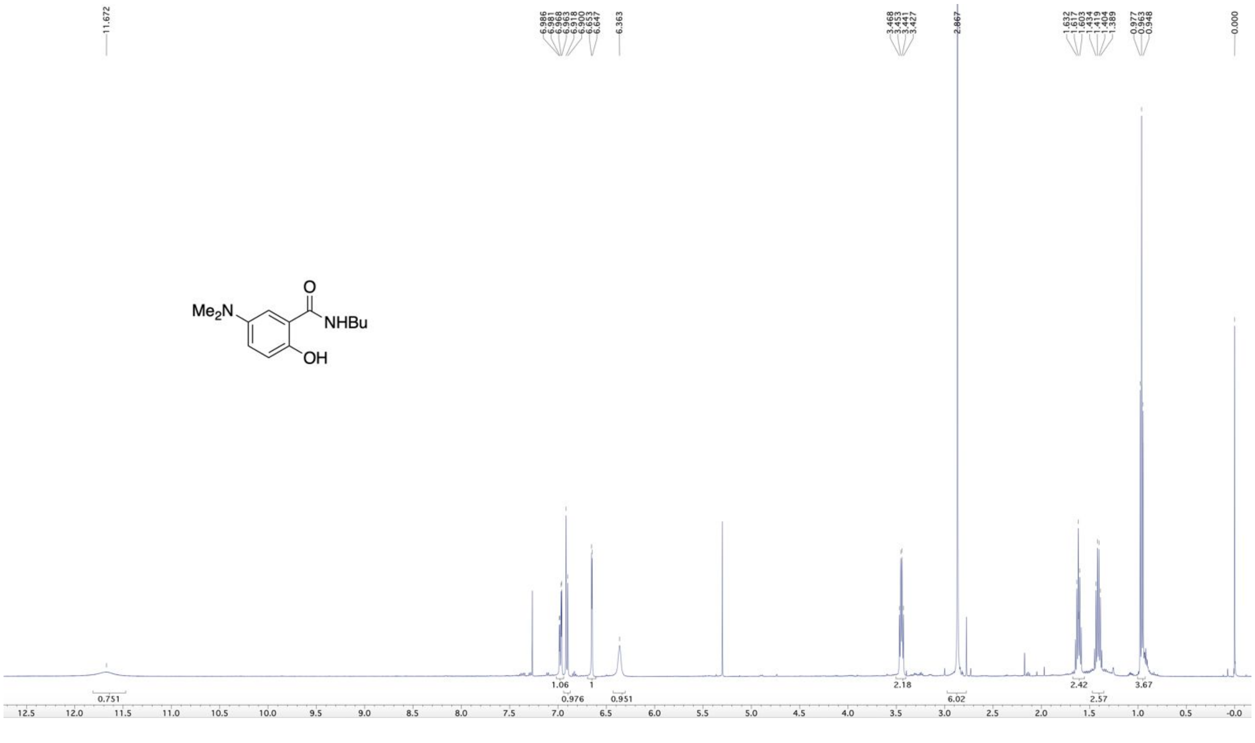

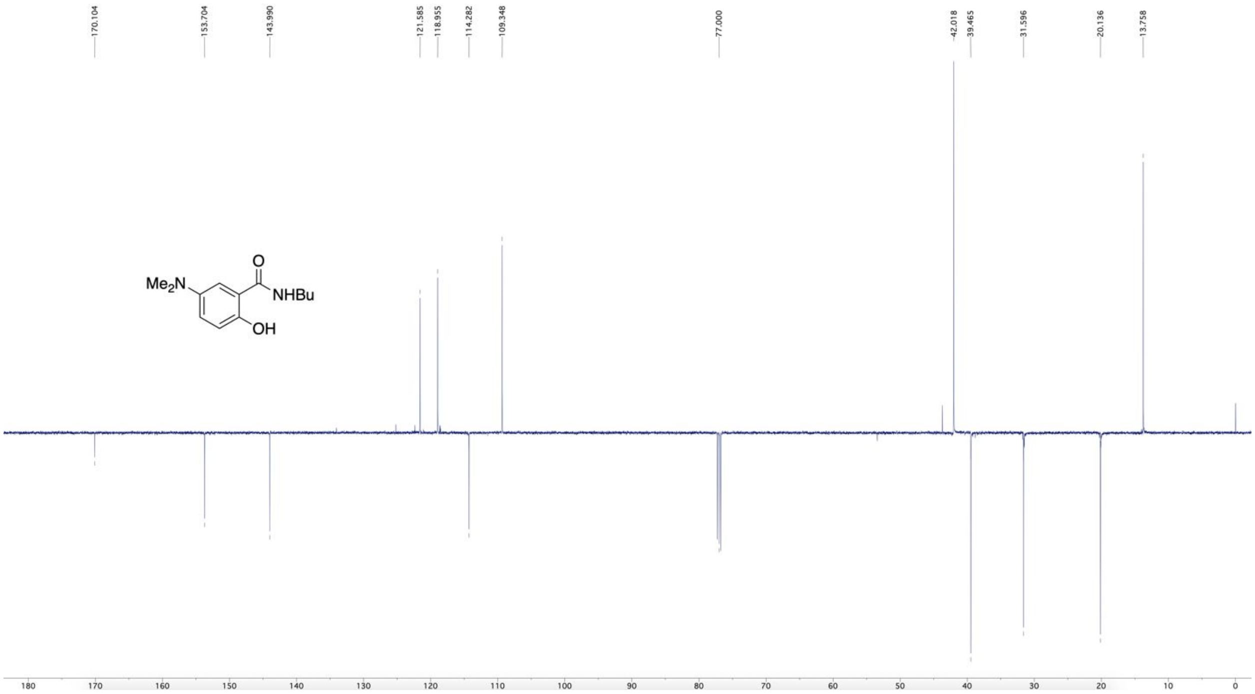

2-(Benzyloxy)-*N*-butyl-5-(dimethylamino)benzamide **42a**

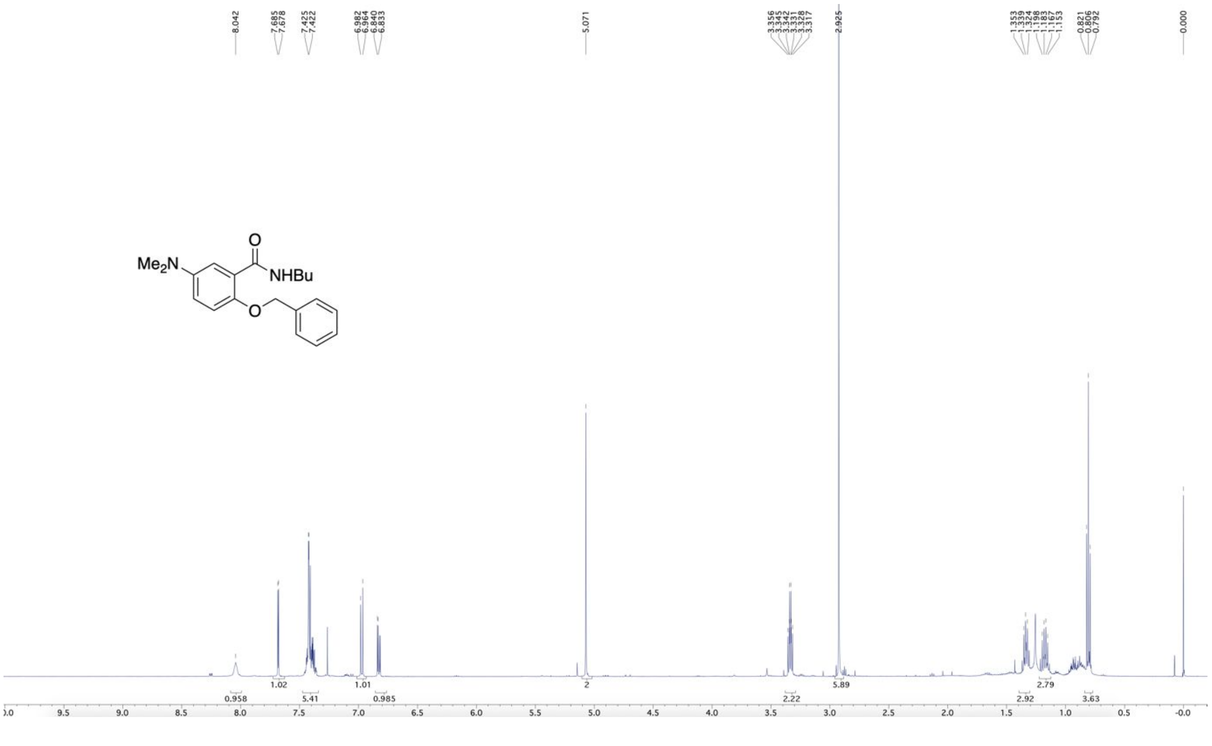

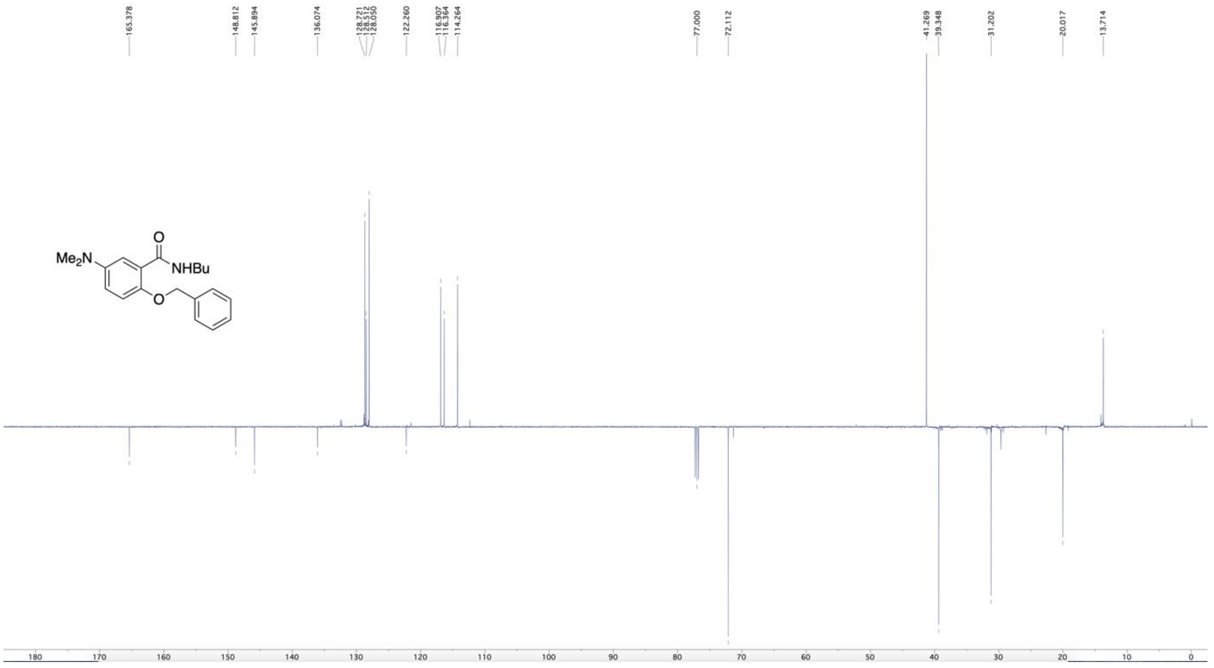

N-Butyl-5-(dimethylamino)-2-((4-nitrobenzyl)oxy)benzamide **42b**

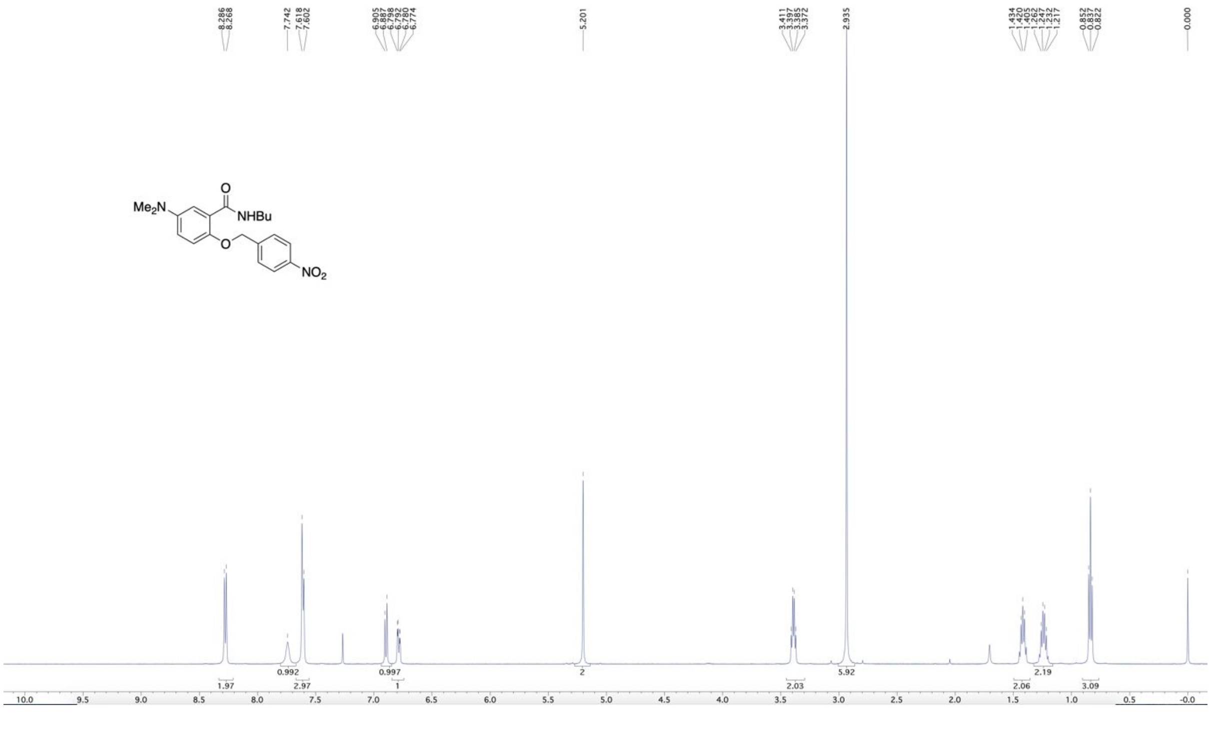

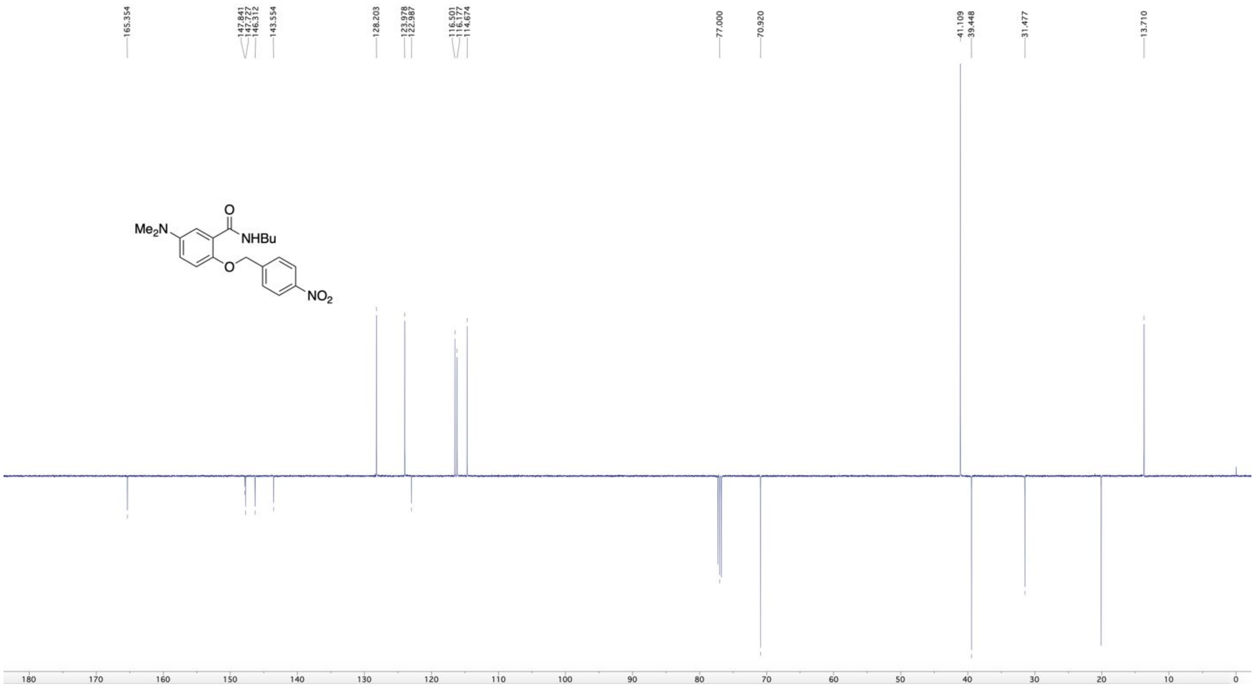

N-Butyl-5-(dimethylamino)-2-((4-methoxybenzyl)oxy)benzamide **42c**

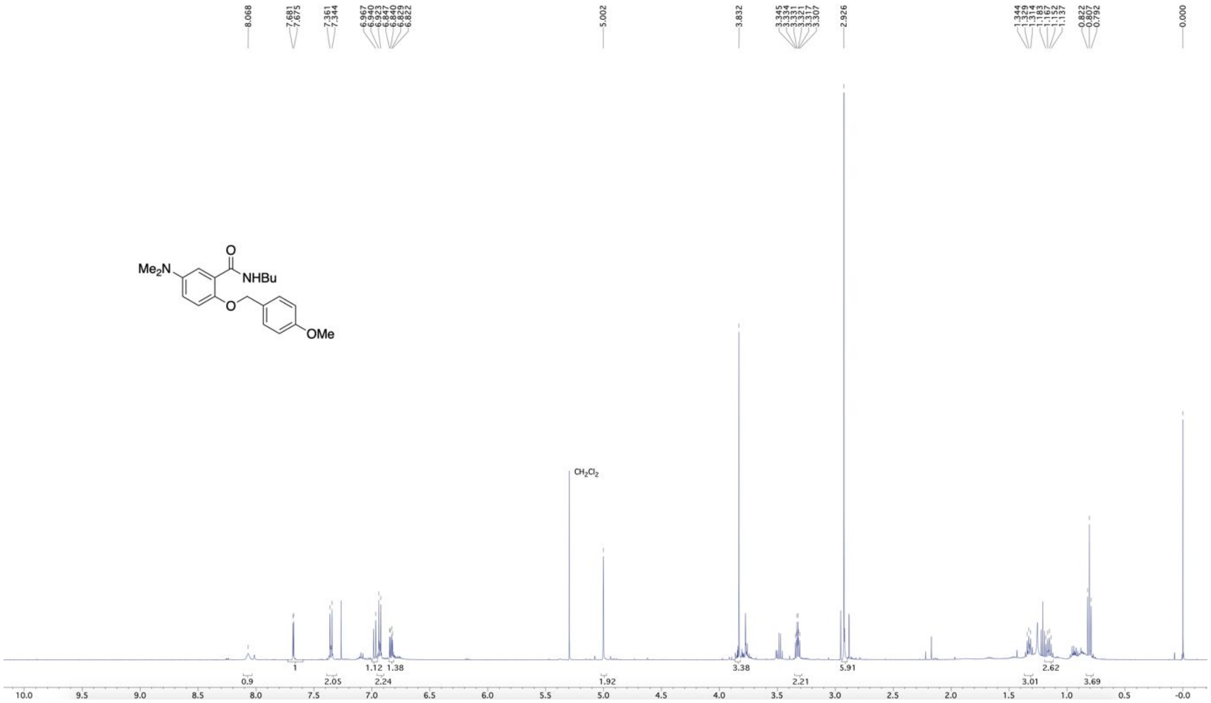

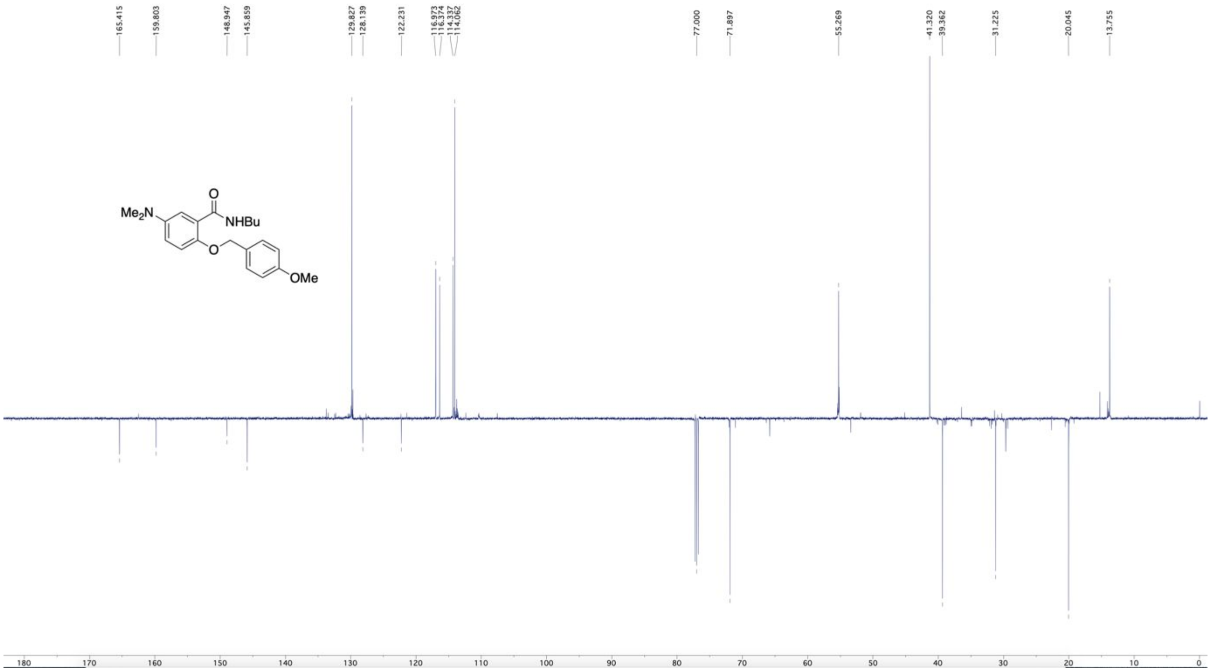

N-Butyl-5-(dimethylamino)-2-(hydroxy(phenyl)methyl)benzamide **43a**

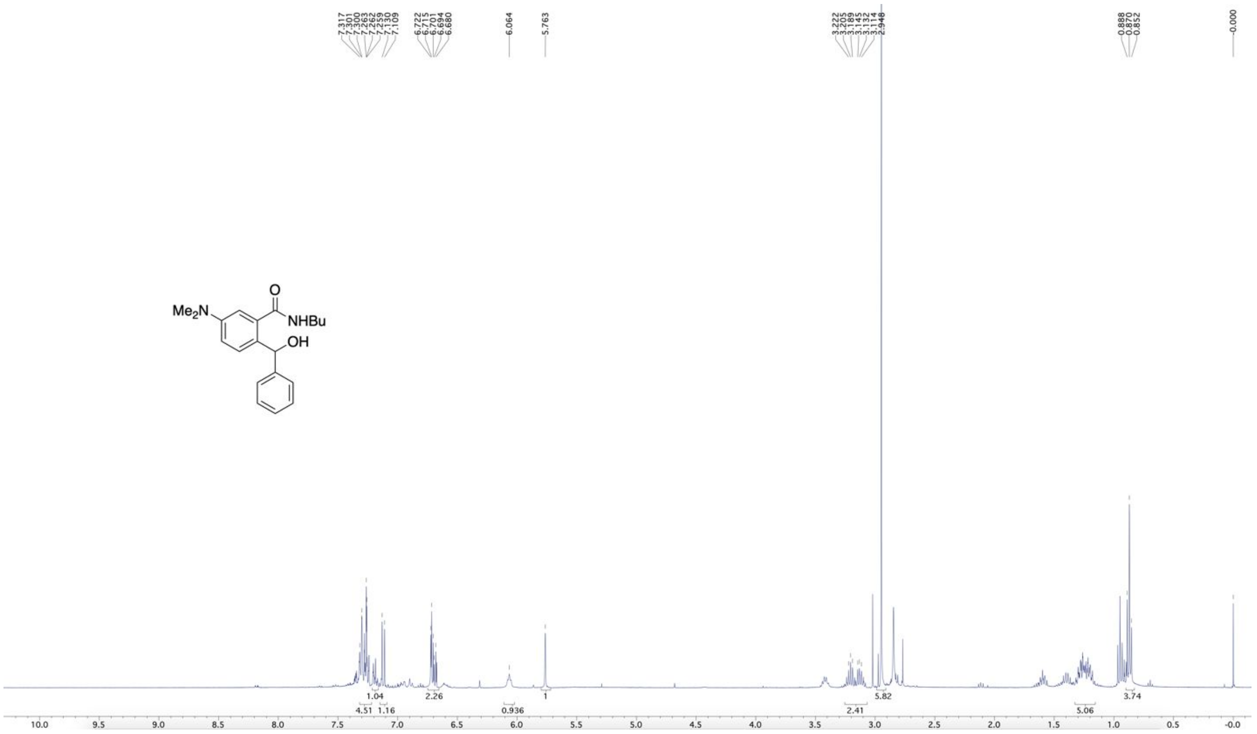

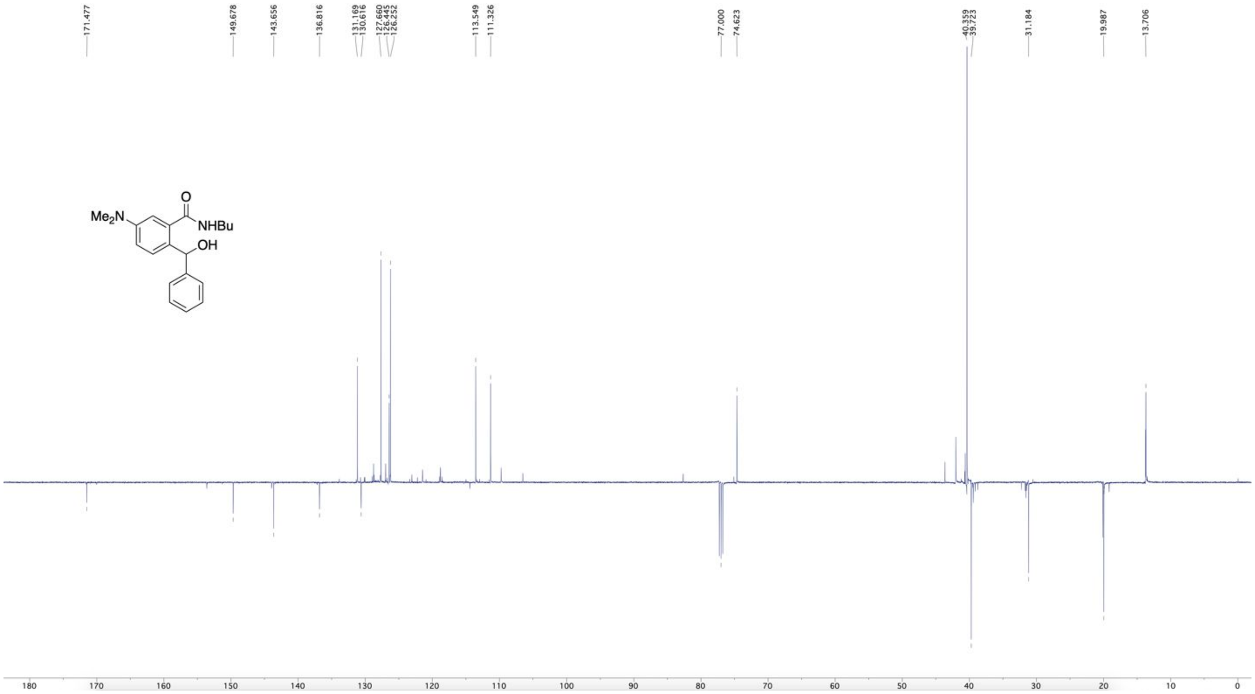

N-Butyl-5-(dimethylamino)-2-(hydroxy(4-methoxyphenyl)methyl)benzamide **43c**

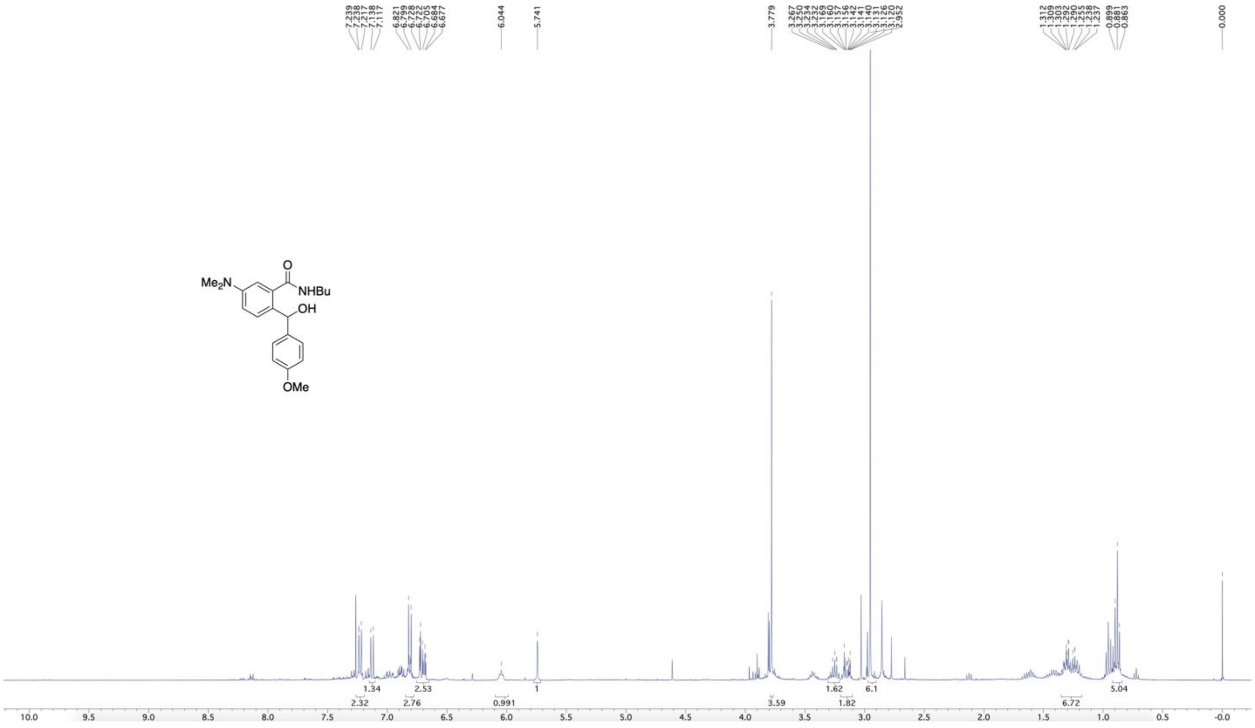

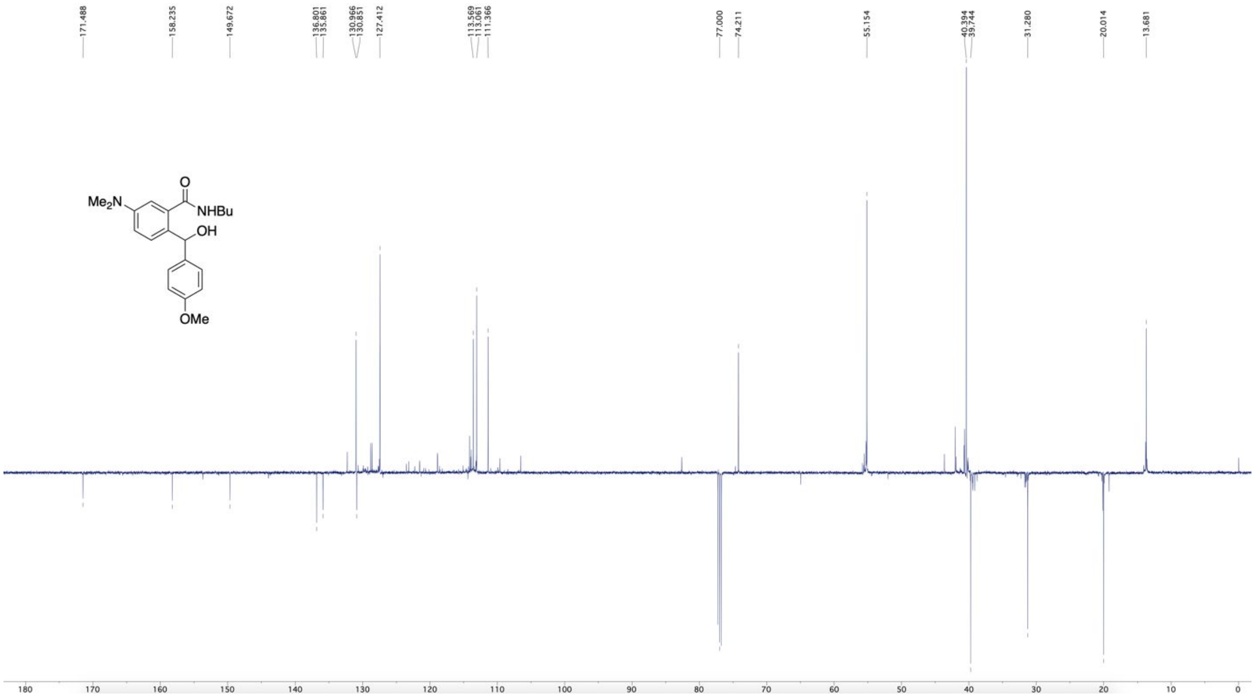

2-(benzyloxy)-*N*-butyl-6-fluorobenzamide **44**

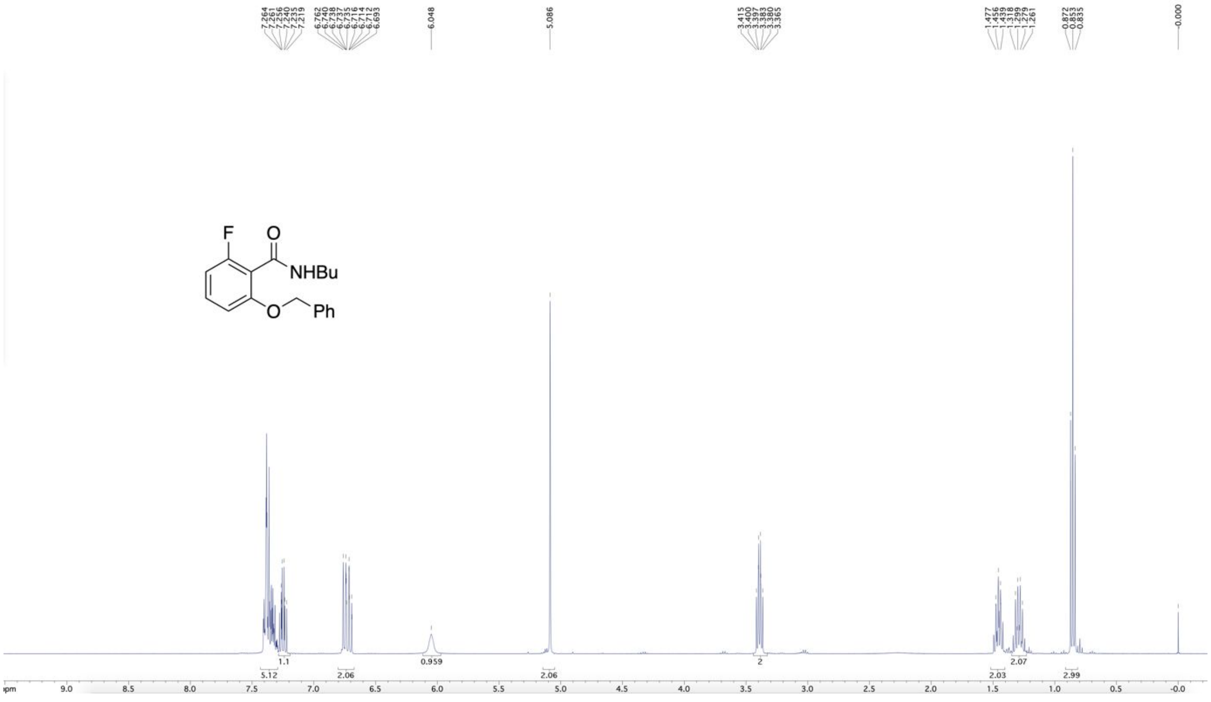

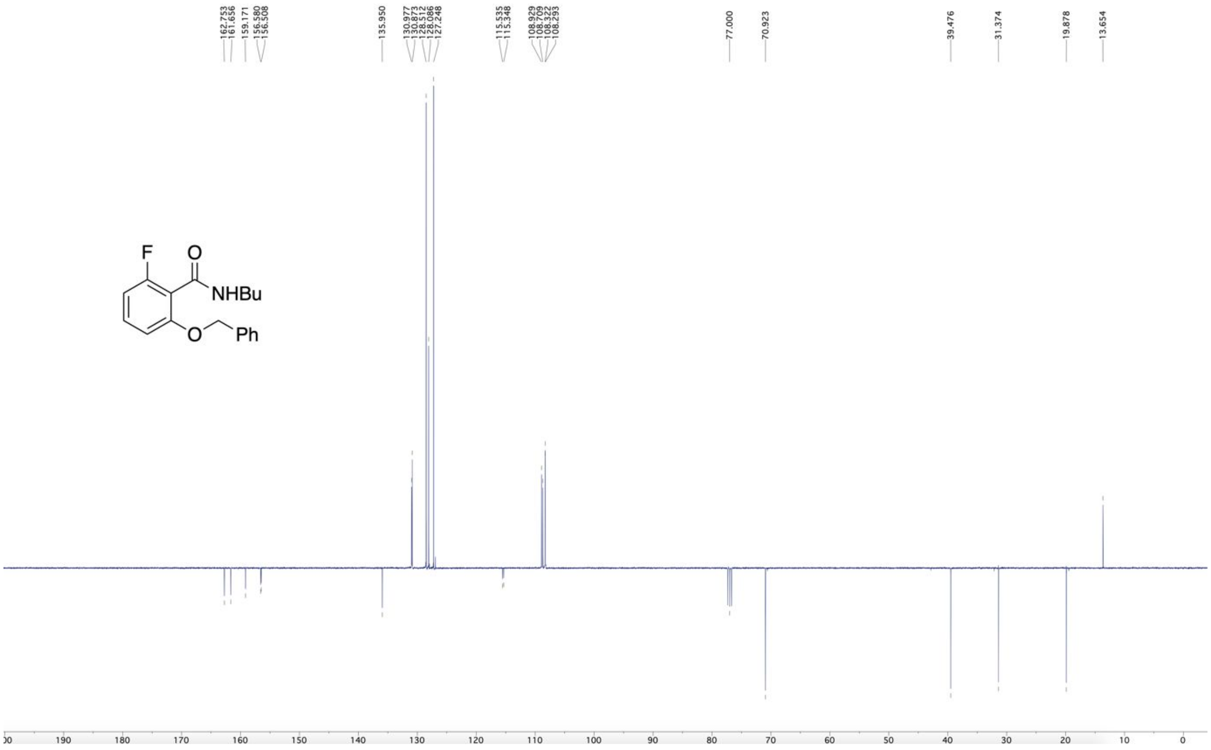
162.753
161.656
159.171
156.580
156.508
135.950
130.977
130.873
128.512
128.086
127.248
115.535
115.348
108.929
108.709
108.322
108.293
77.000
70.923
39.476
31.374
19.878
13.654
F
O
NHBu
O
Ph
190
180
170
160
150
140
130
120
110
100
90
80
70
60
50
40
30
20
10
0

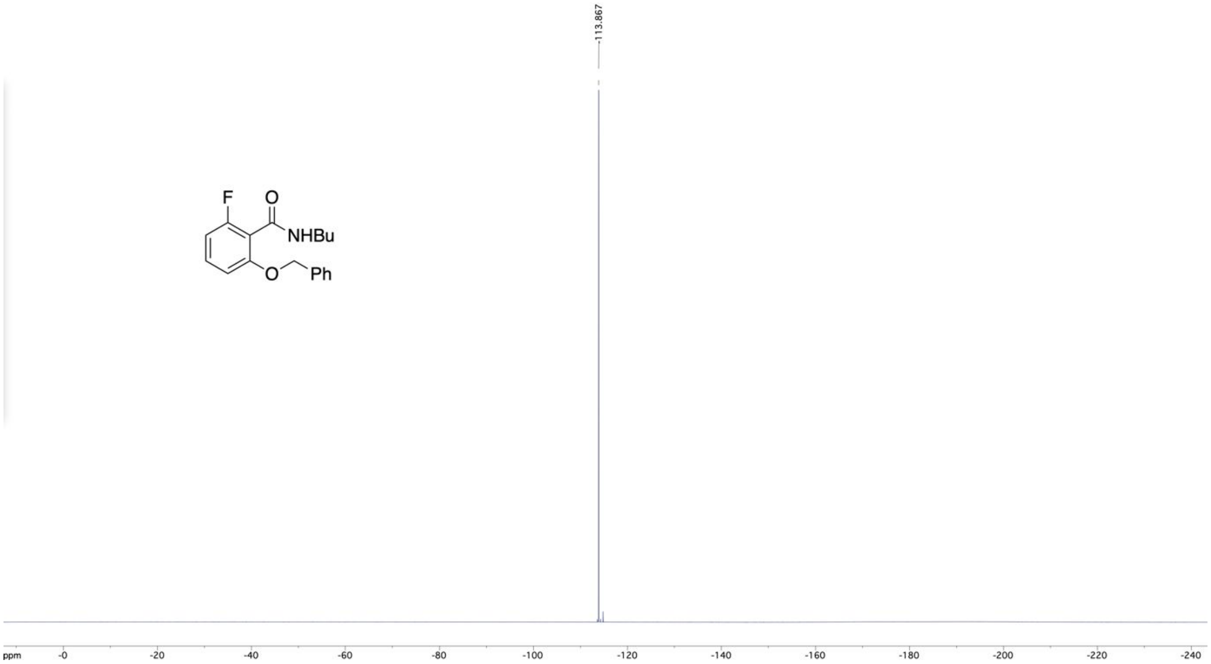
-113.867
F
O
NHBu
O
Ph
ppm
-0
-20
-40
-60
-80
-100
-120
-140
-160
-180
-200
-220
-240

7-butyl-3-phenylisobenzofuran-1(3*H*)-one **47**

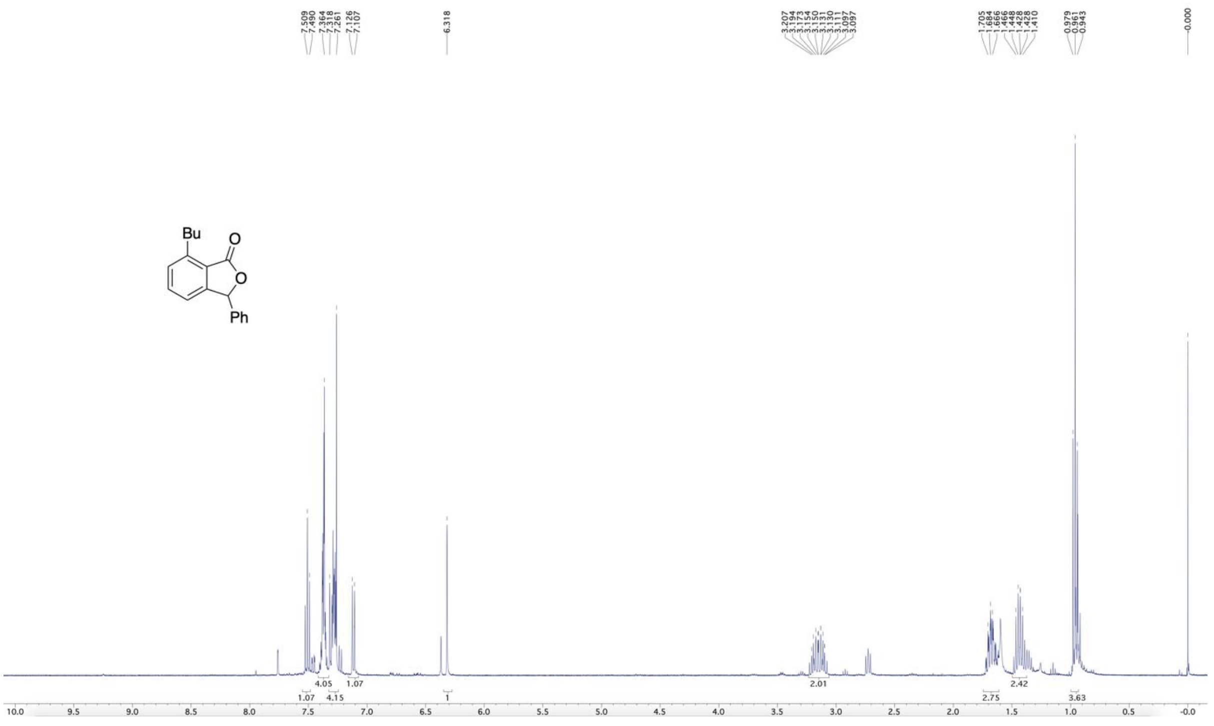

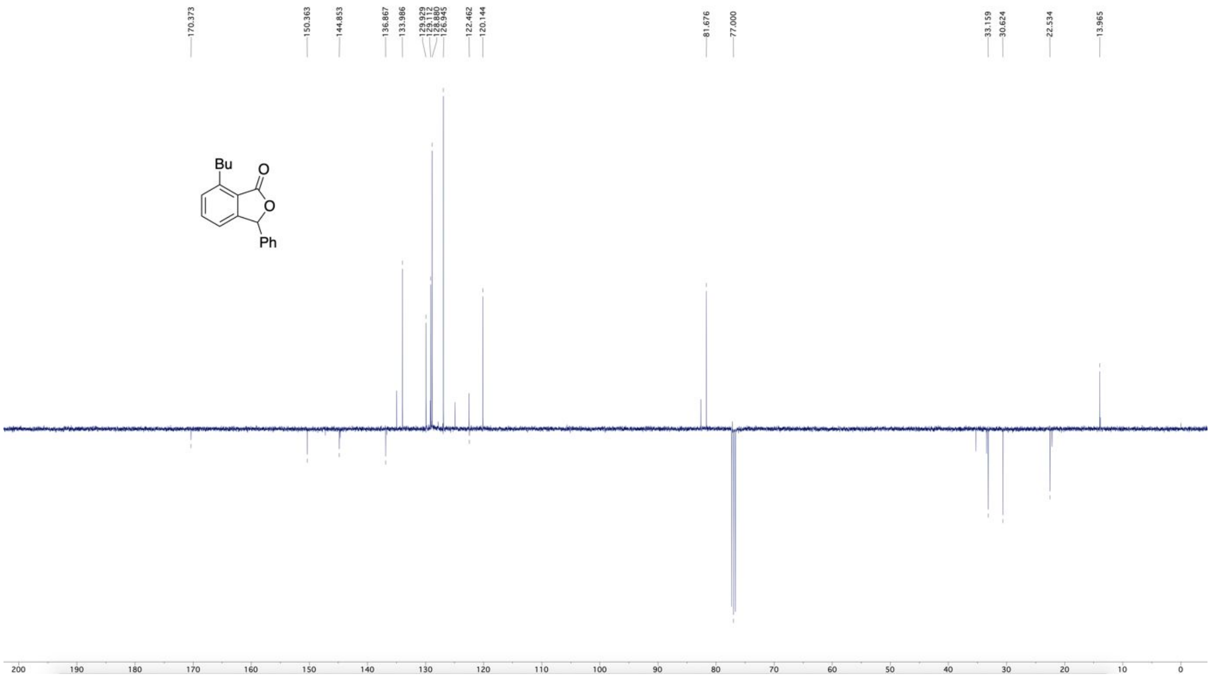

2-(benzyloxy)-*N*-butyl-5-methoxybenzamide **48**

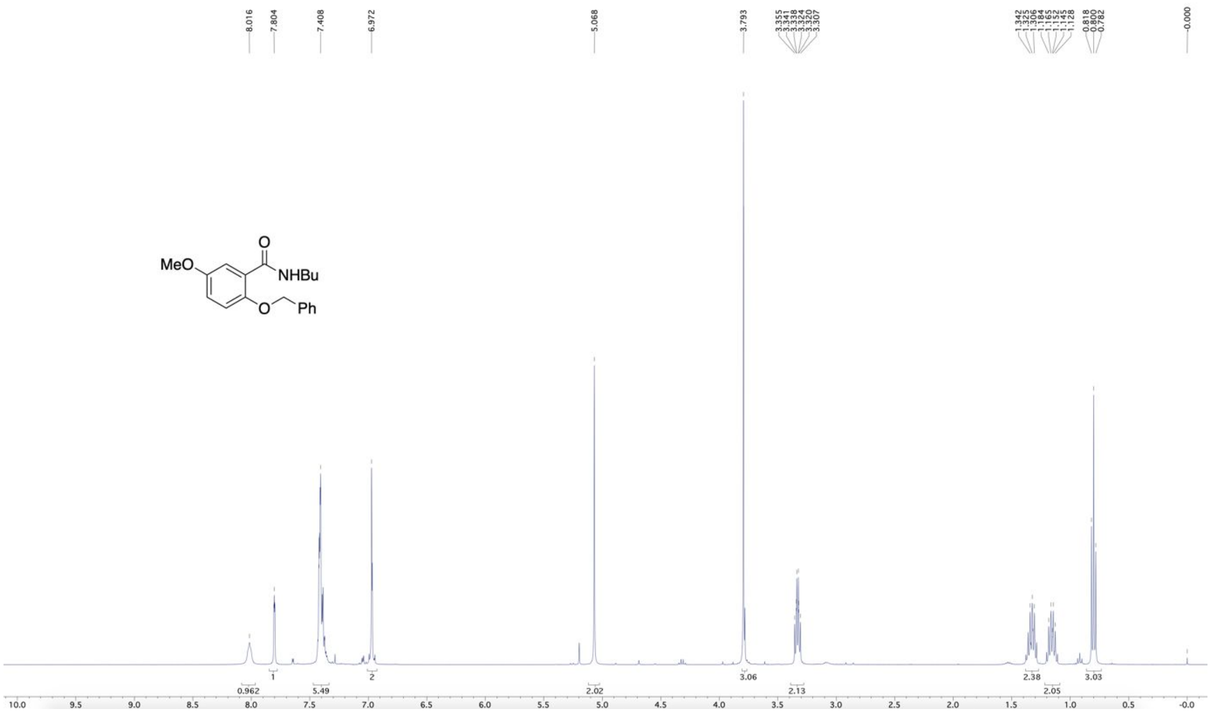

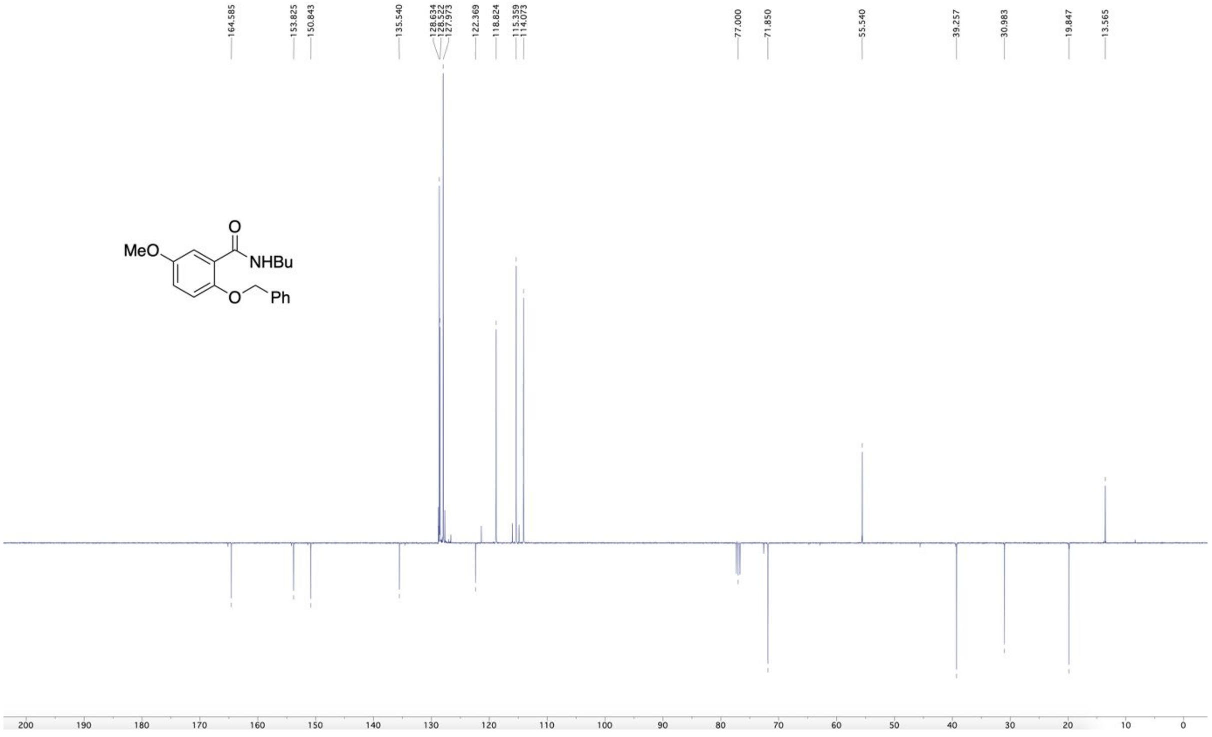

2-(benzyloxy)-*N*-butyl-4-methoxybenzamide **51**

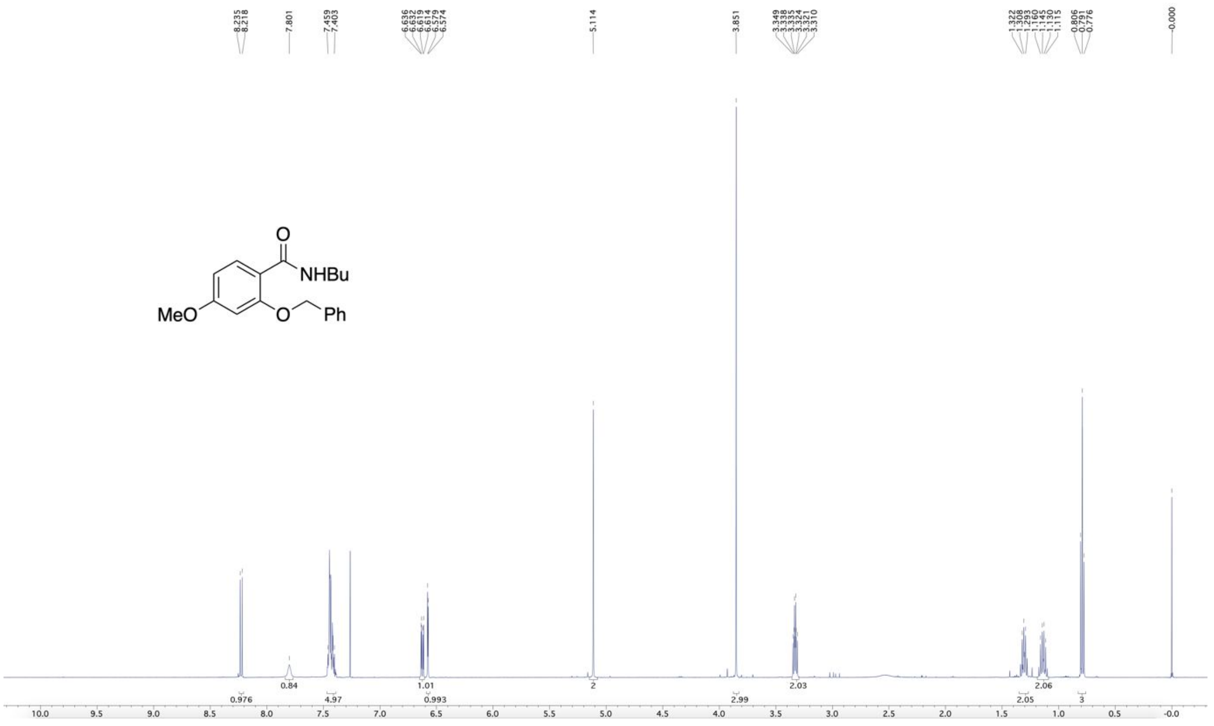

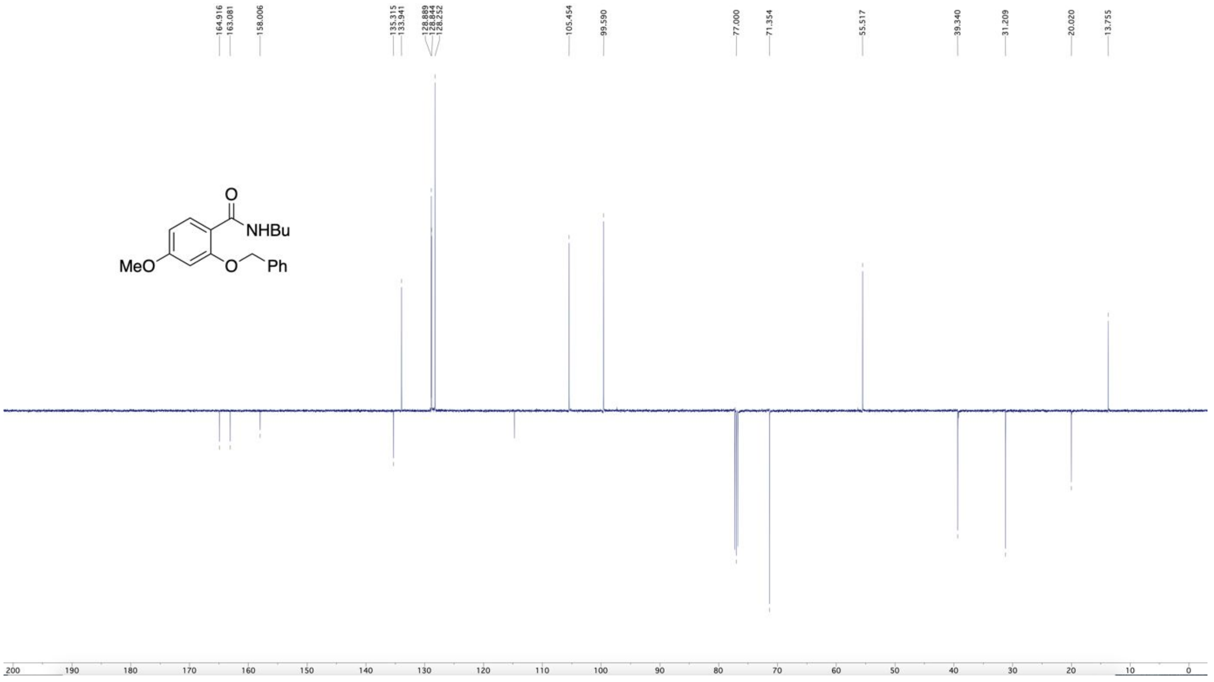

2-(benzyloxy)-*N*-butyl-5-fluorobenzamide **54**

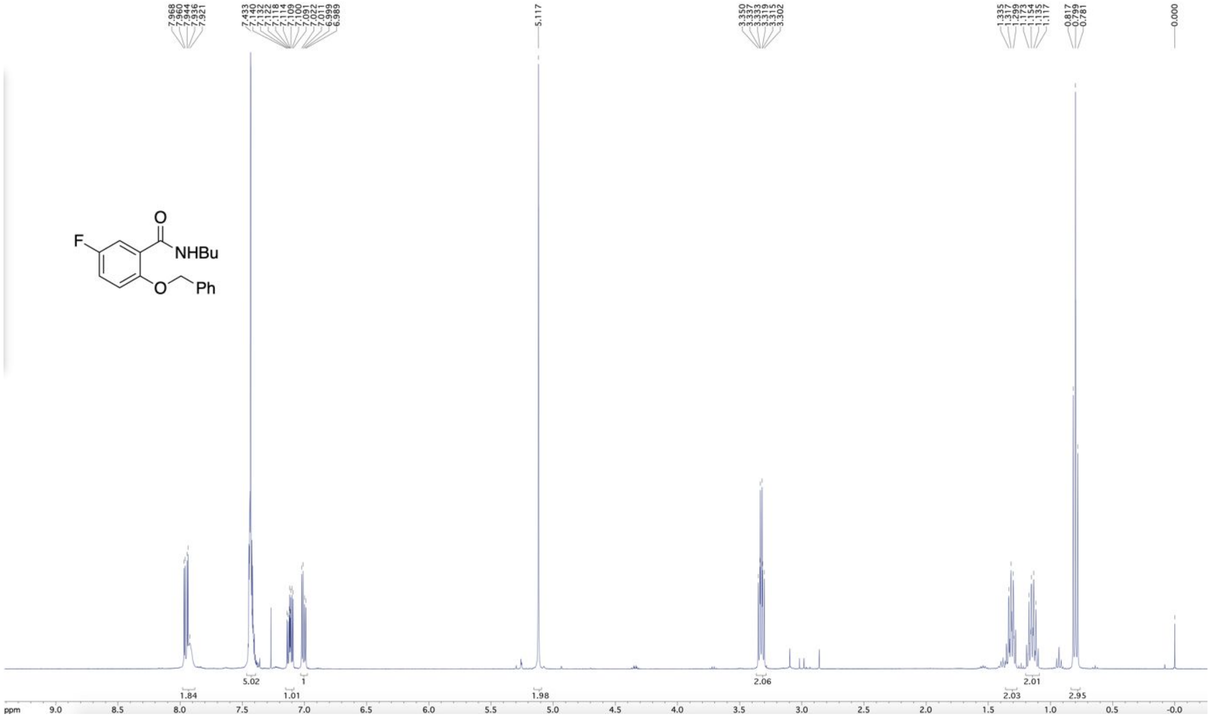

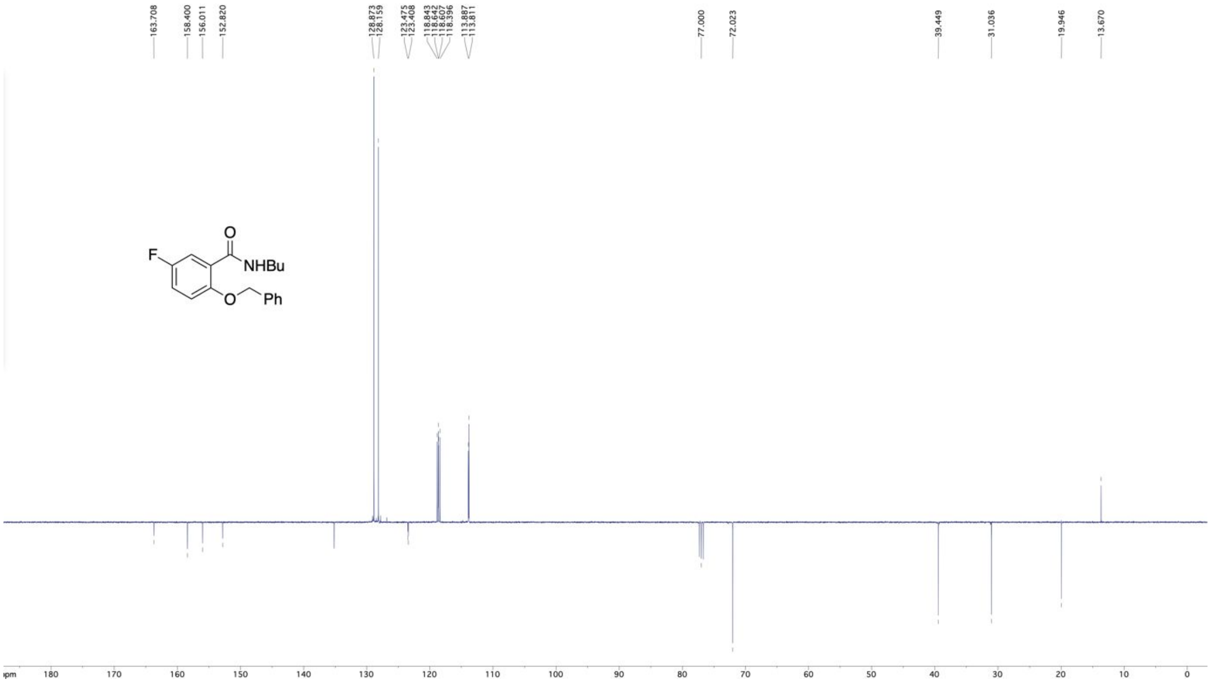

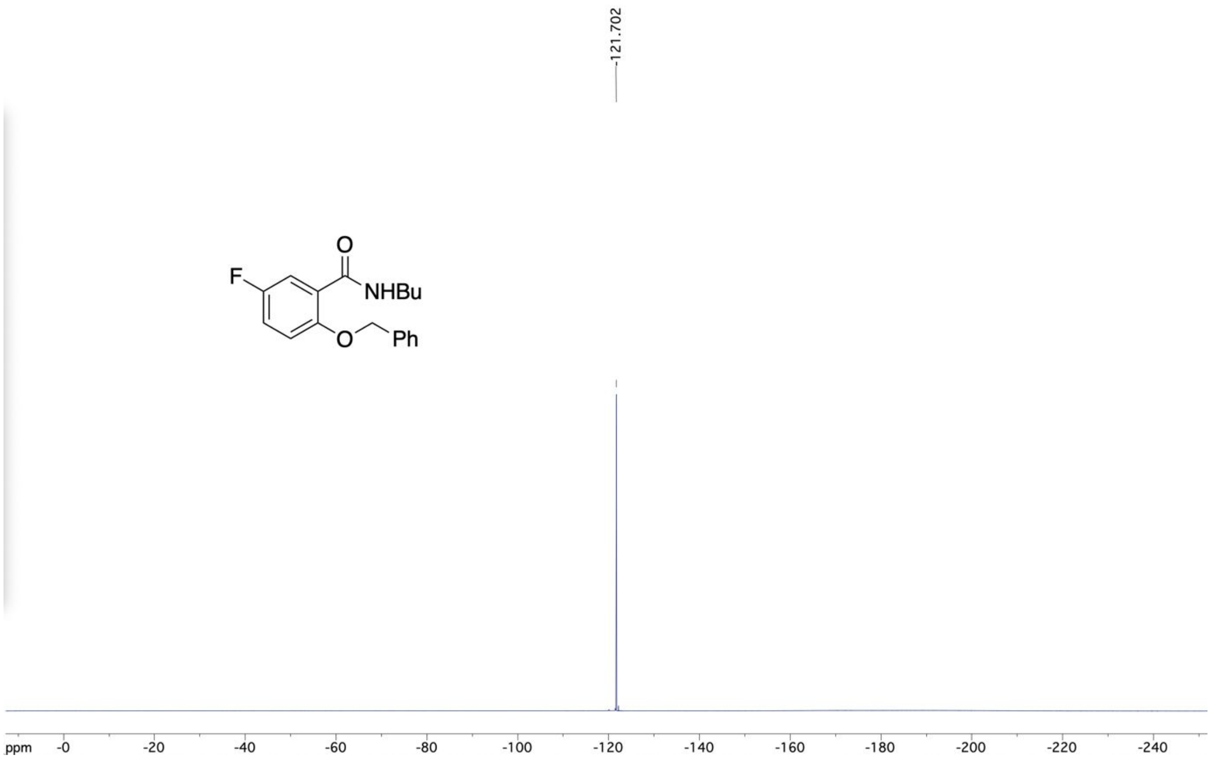

4-(benzyloxy)-3,5-dimethylbenzoic acid **F**

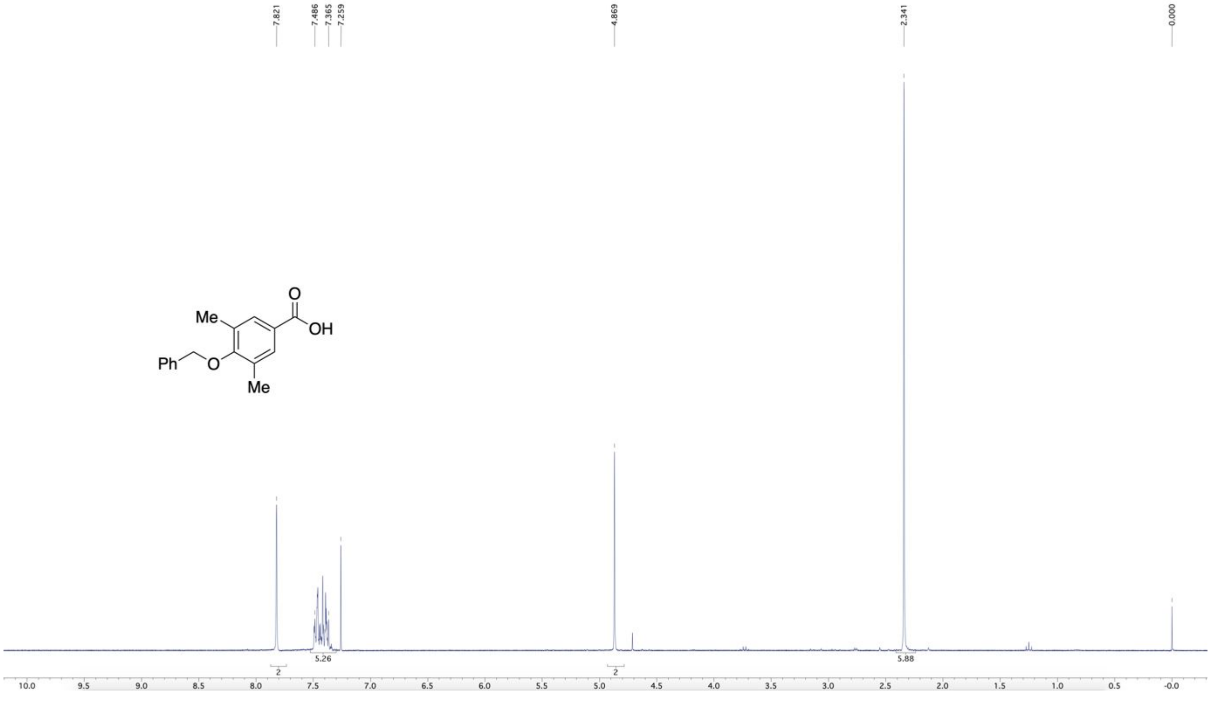

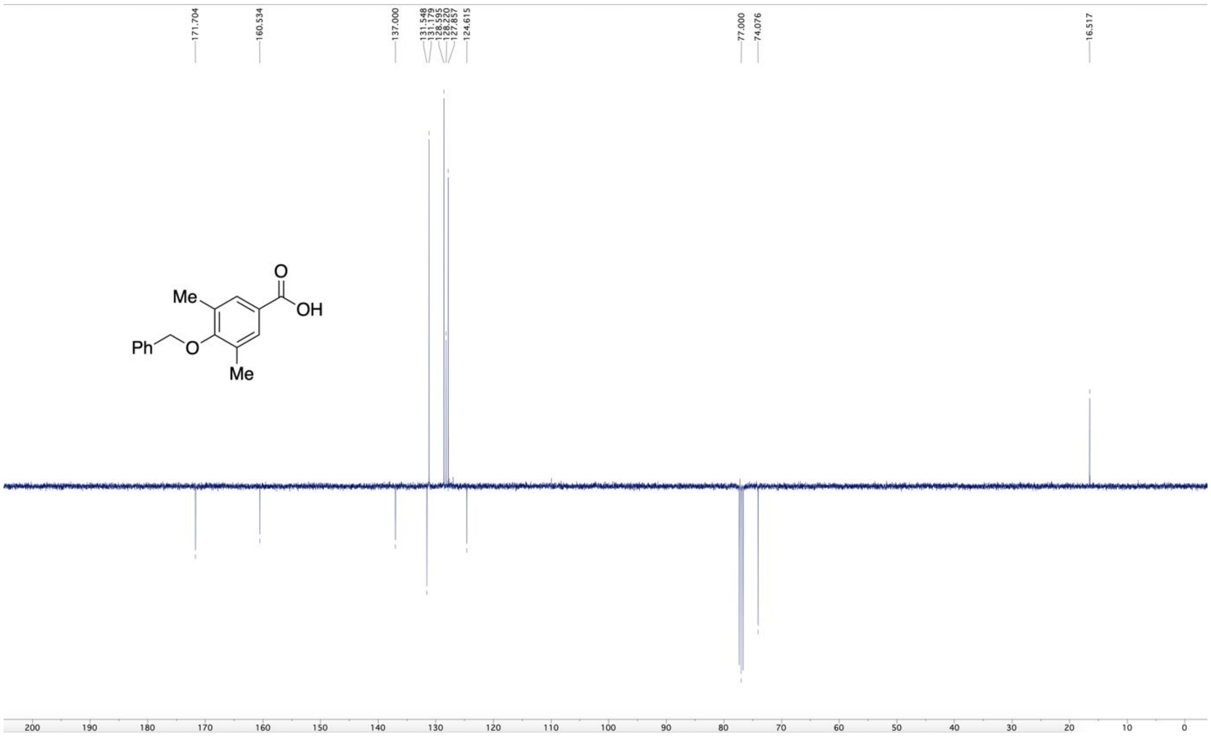

4-(benzyloxy)-*N*-butyl-3,5-dimethylbenzamide **55**

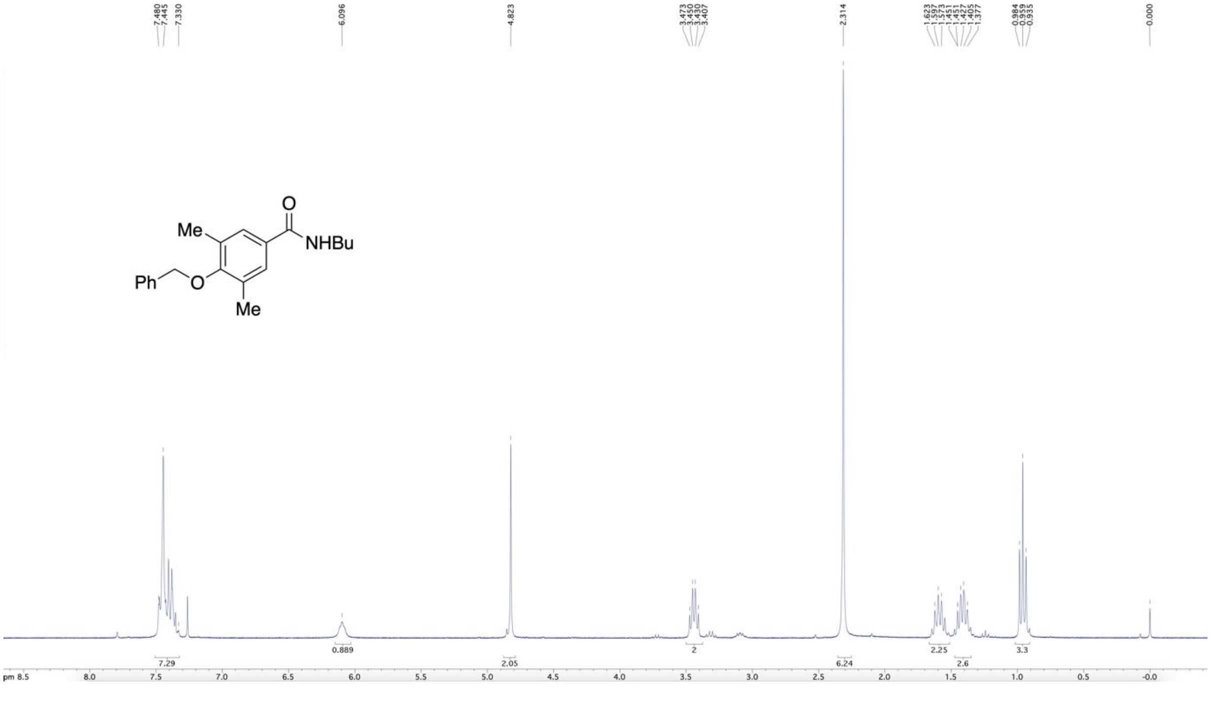

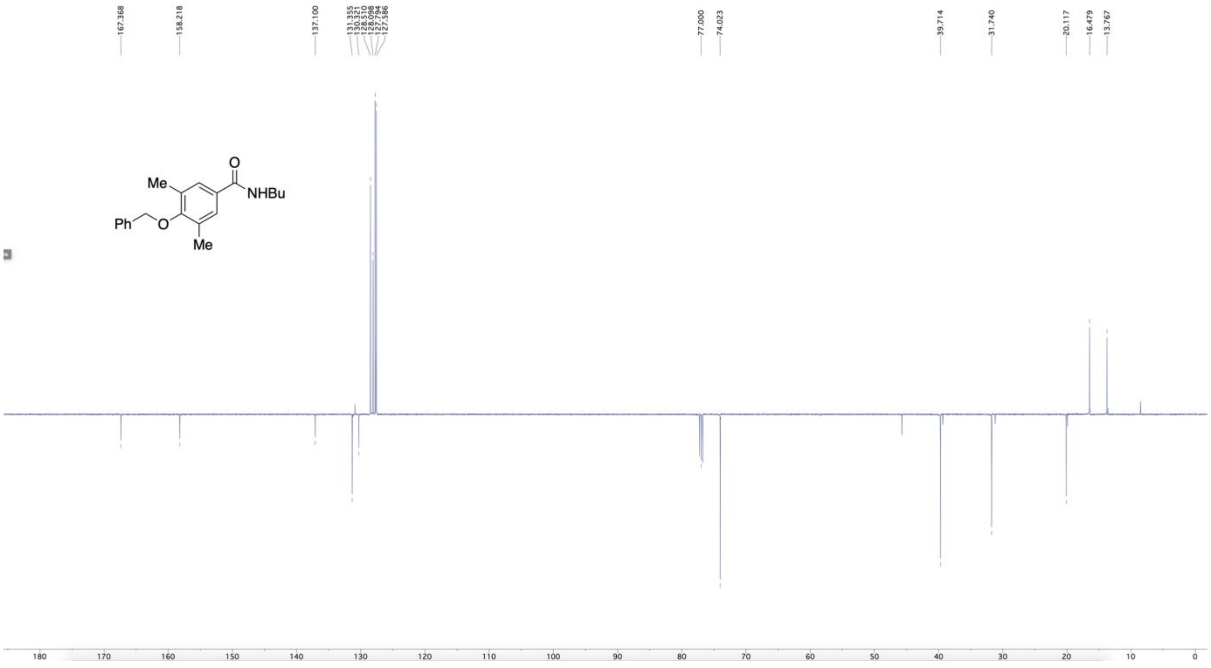

N-butyl-2-(phenoxymethyl)benzamide **58**

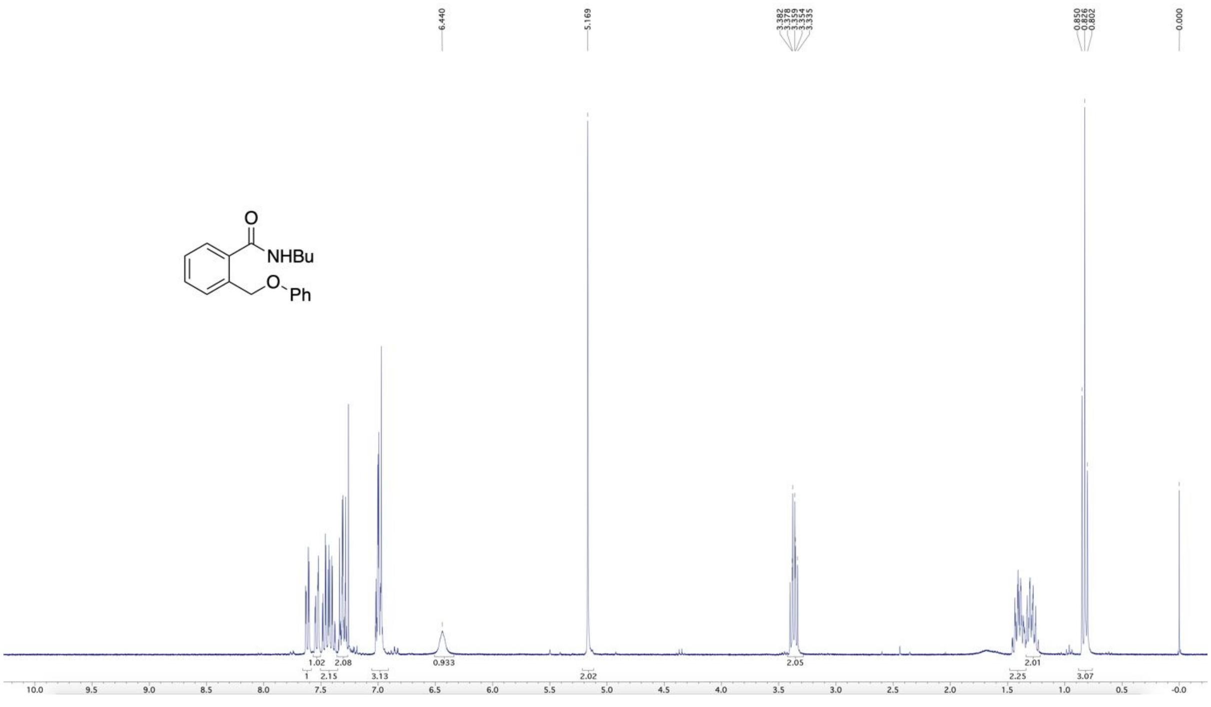

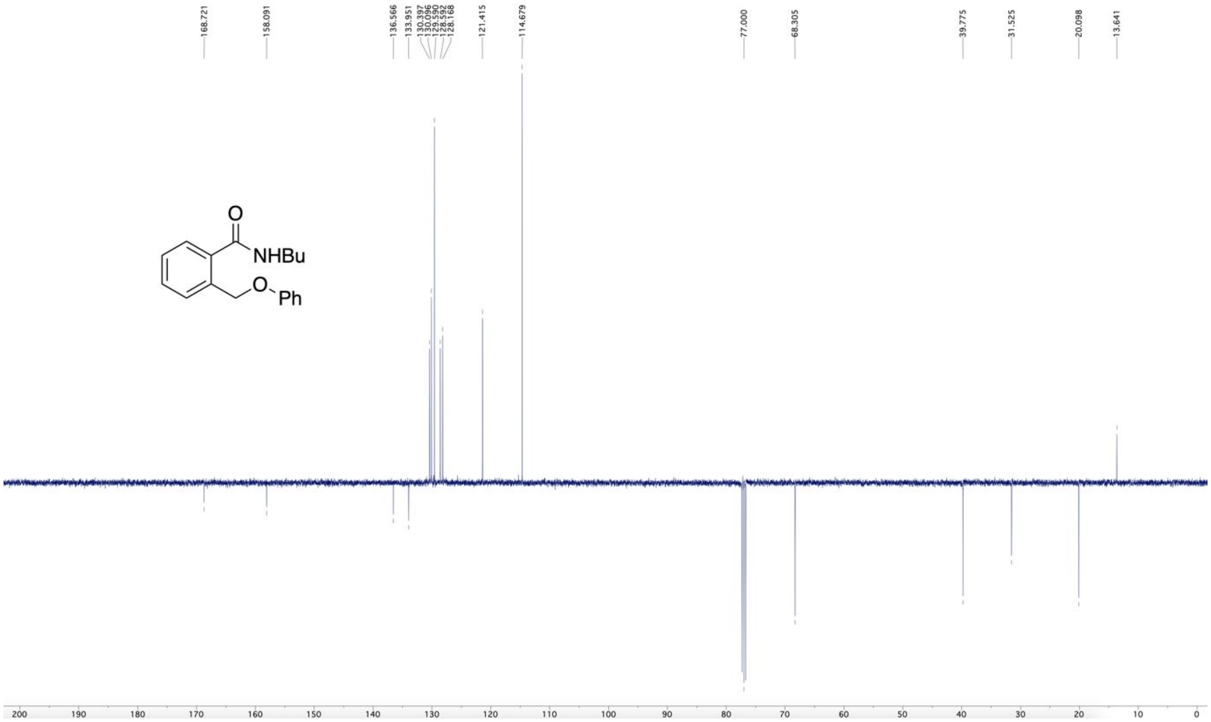

N-butyl-4-(phenoxymethyl)benzamide **59**

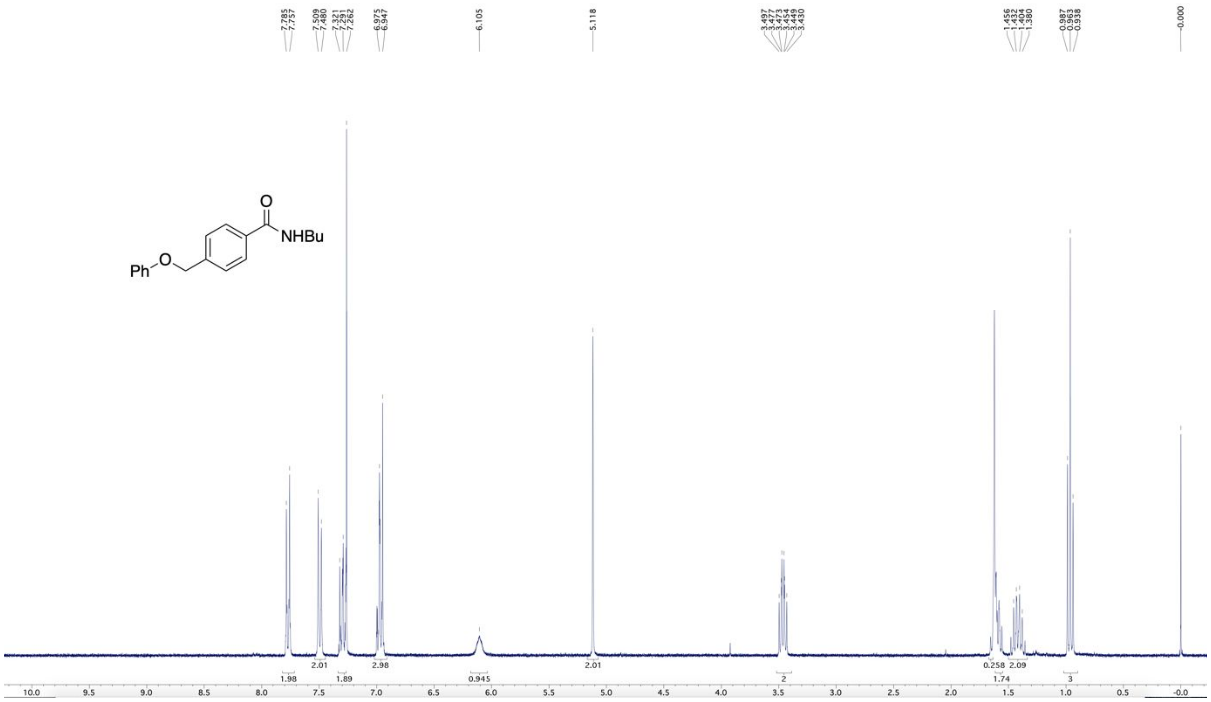

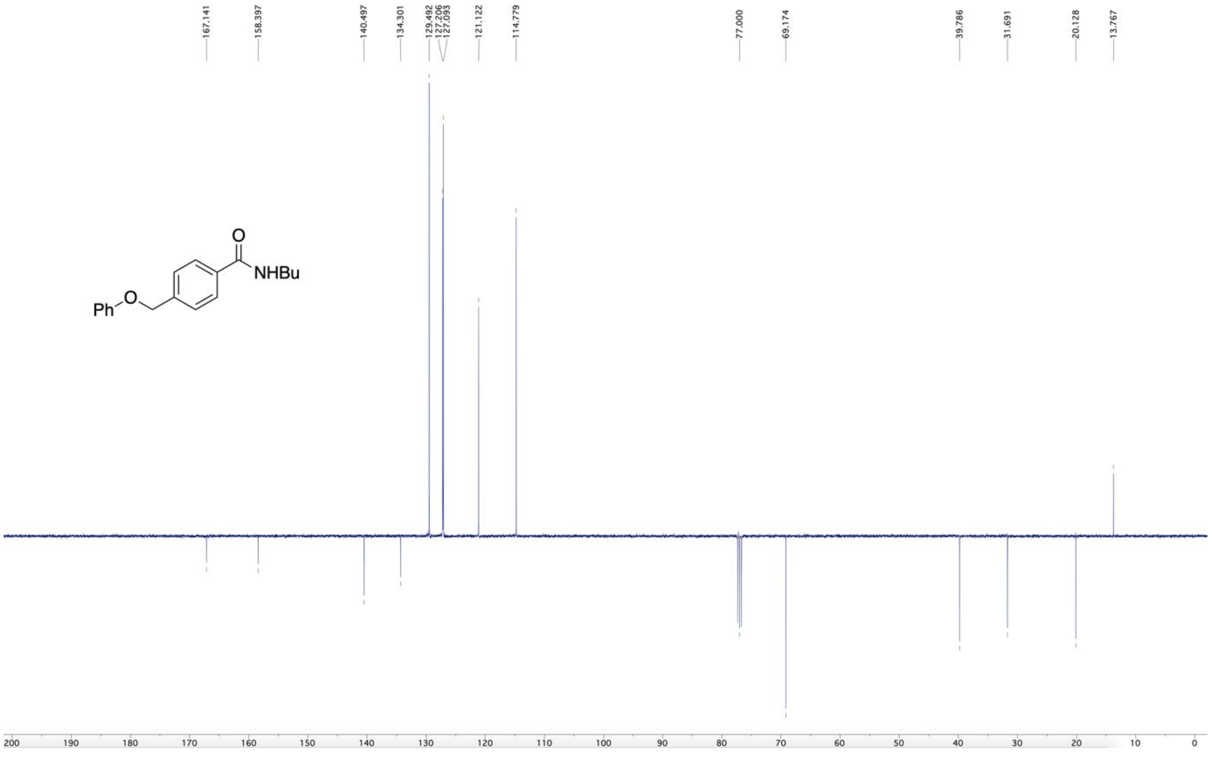

2-(Benzylthio)-*N*-butylbenzamide **66**

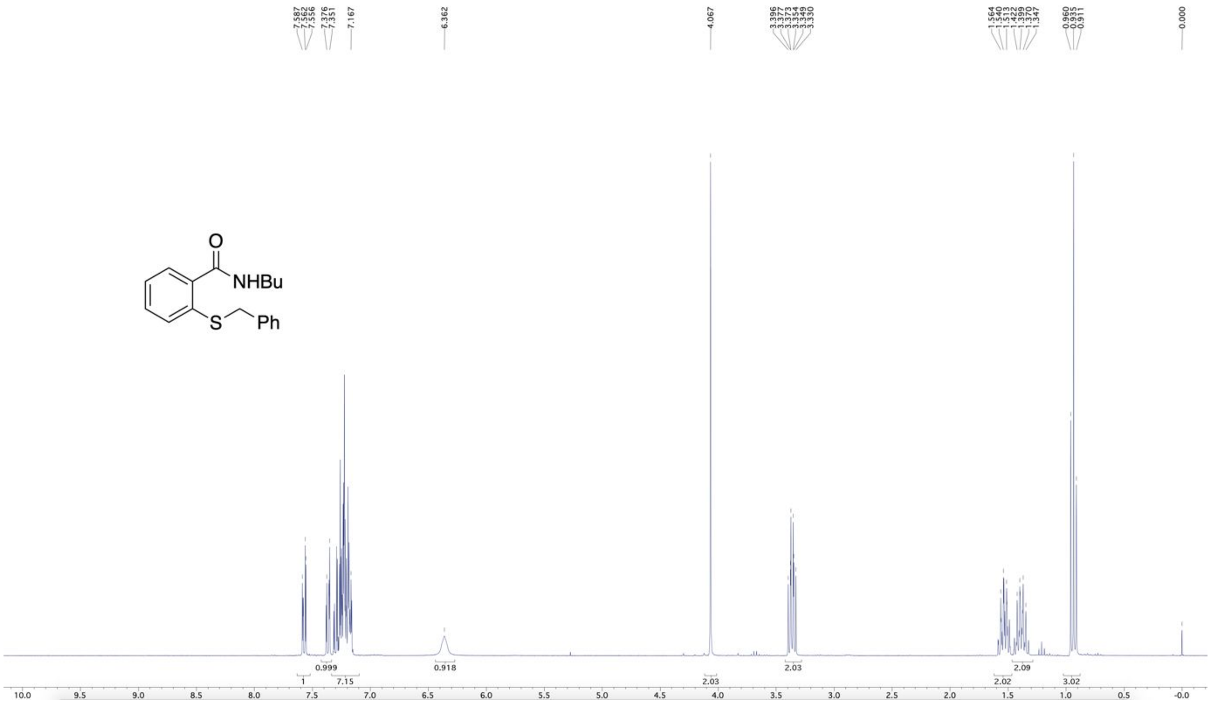

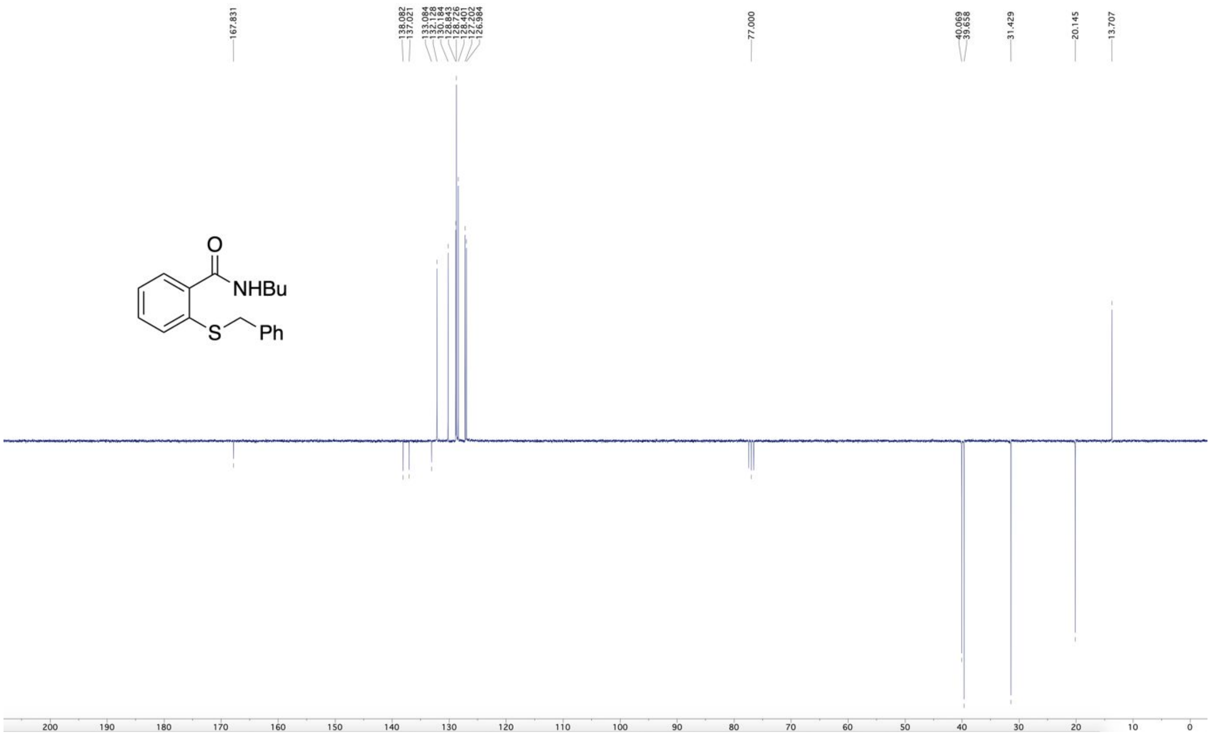

N-butyl-2-phenylbenzo[*b*]thiophen-3-amine **67**

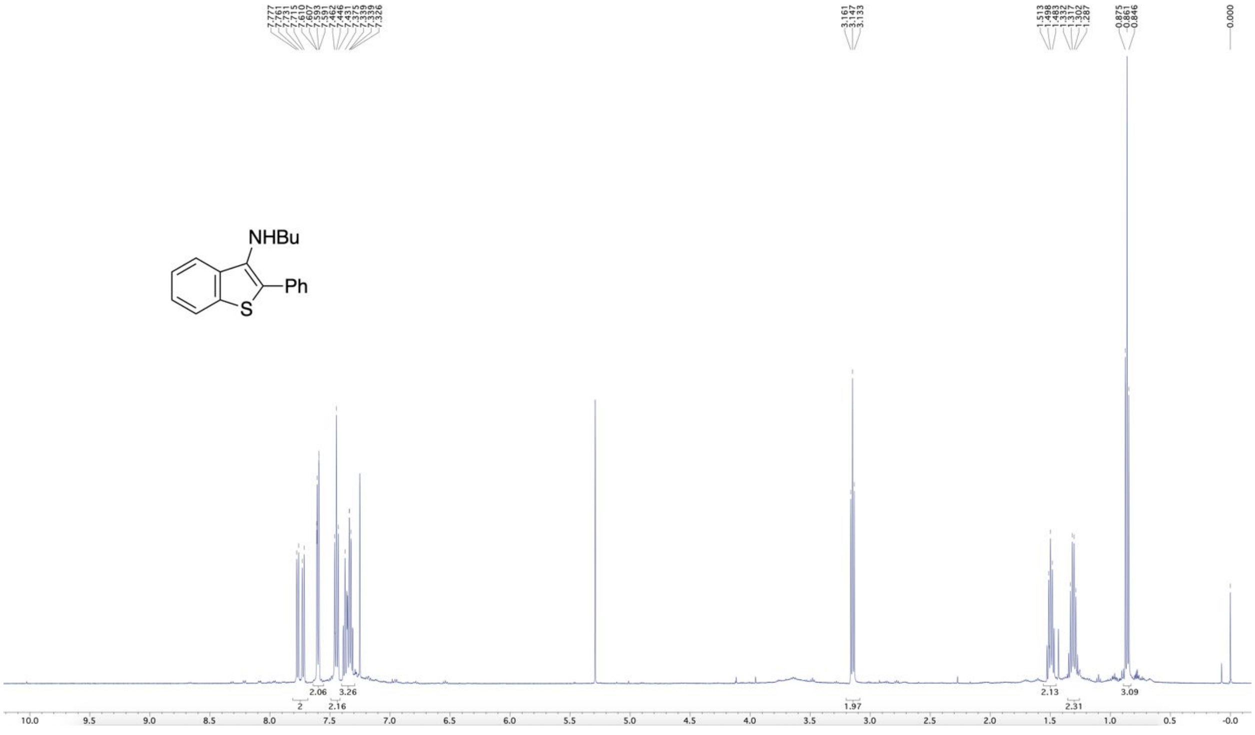

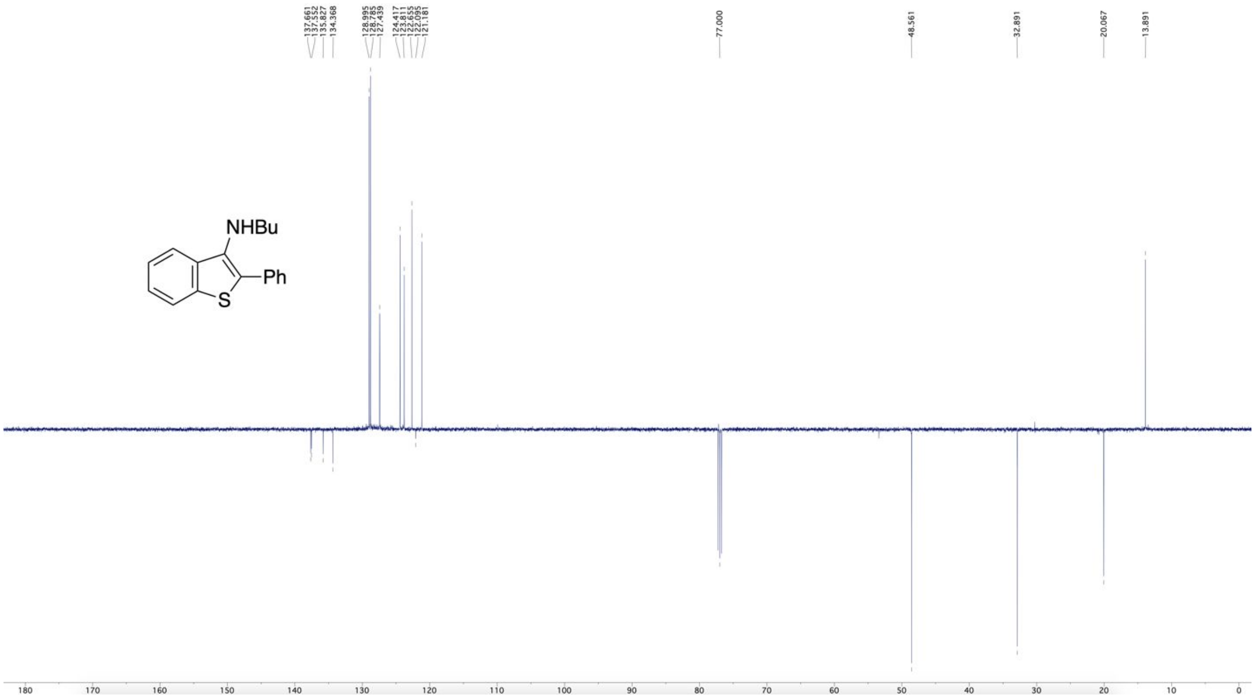

2-(Benzylthio)-*N*-(*tert*-butyl)benzamide **68**

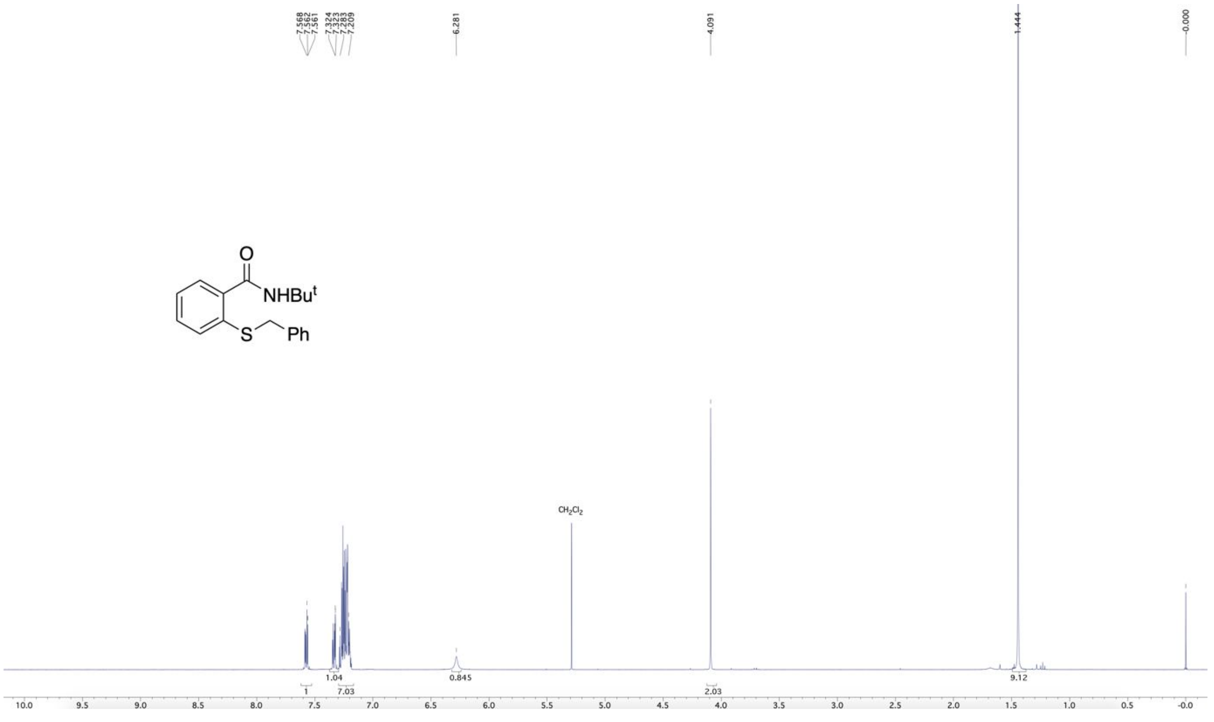

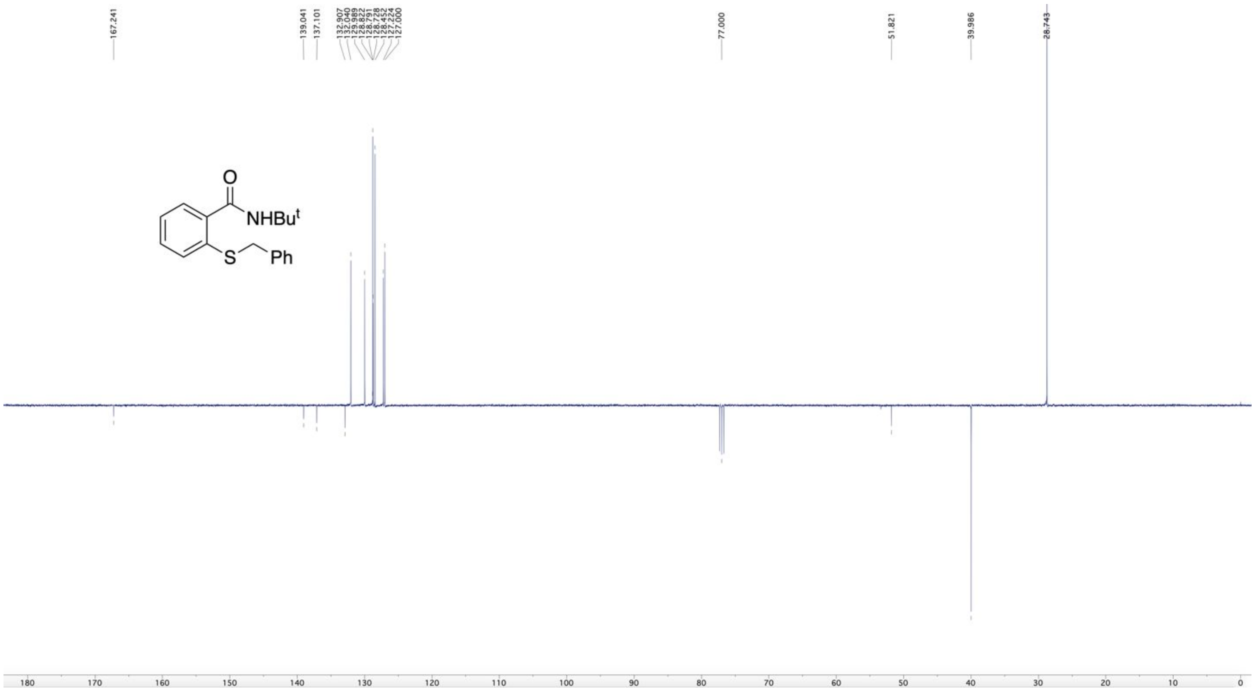

2-(Benzyl(methyl)amino)-*N*-butylbenzamide **69**

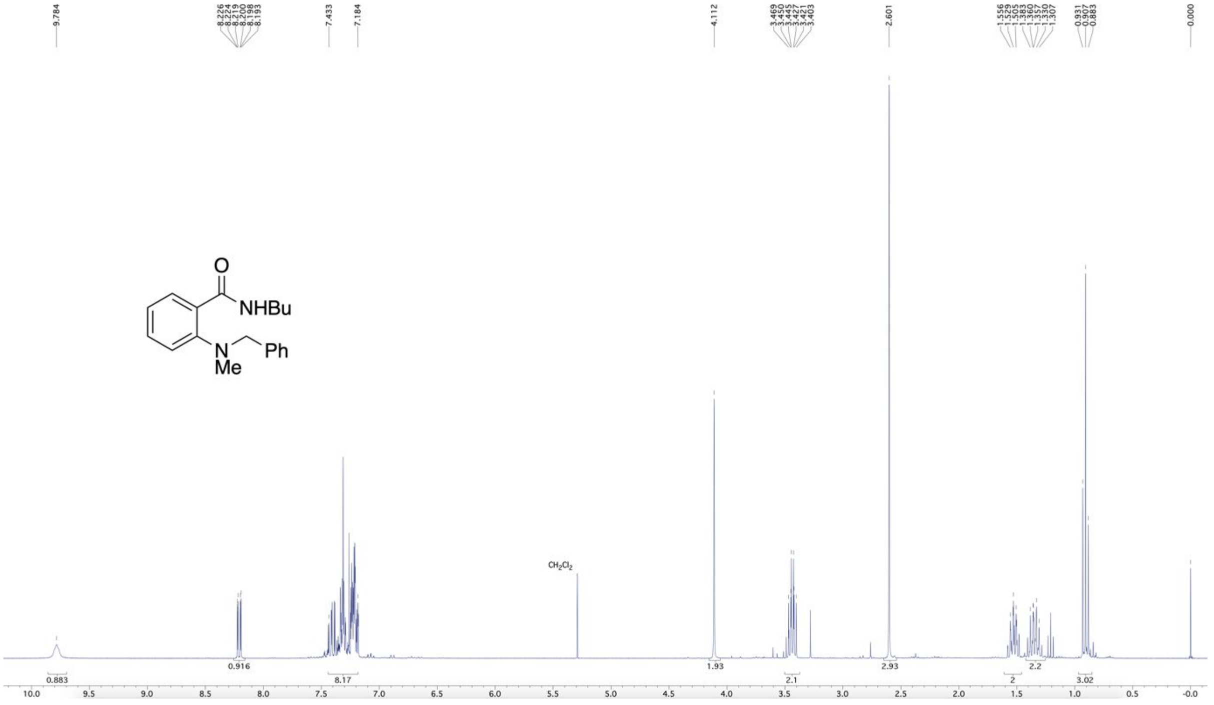

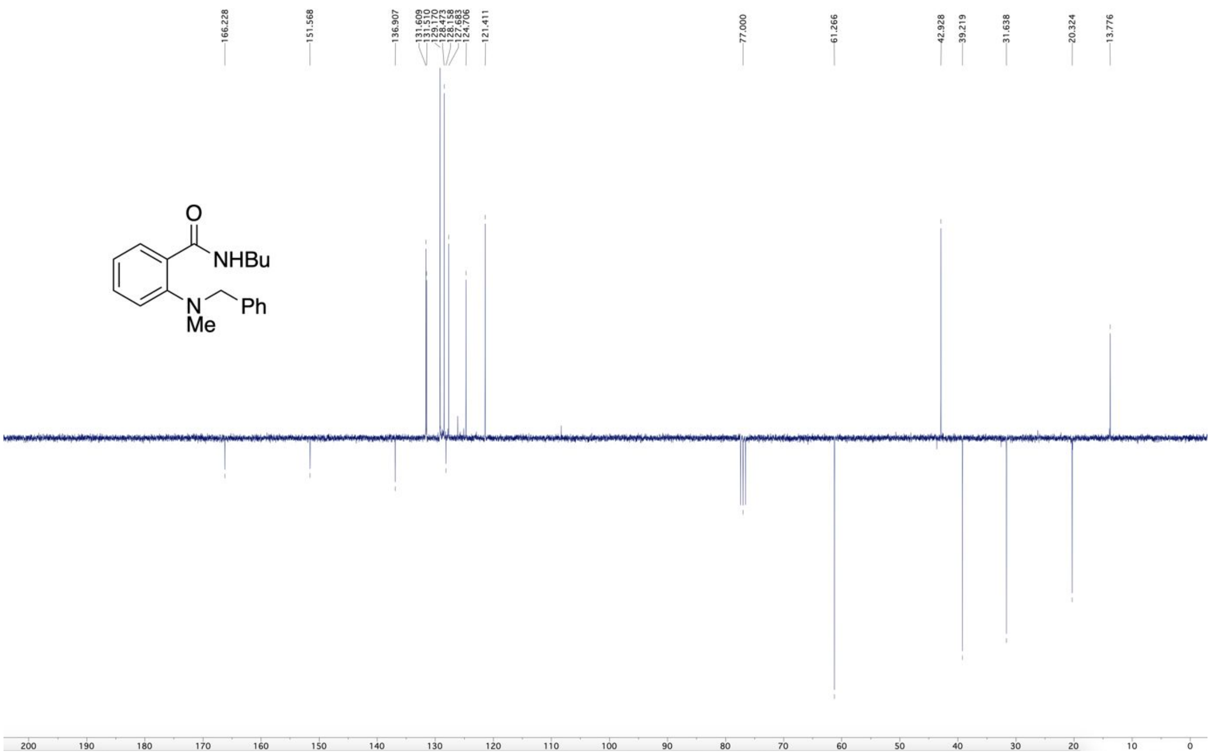
166.228
151.568
136.907
131.609
131.510
129.170
128.473
128.158
127.683
124.706
121.411
77.000
61.266
42.928
39.219
31.638
20.324
13.776
NHBu
O
N
Me
Ph
200
190
180
170
160
150
140
130
120
110
100
90
80
70
60
50
40
30
20
10
0

X-Ray Structures

The following crystal data were obtained: **4** (CCDC No. 2105490)
$C_{25}H_{23}NO_3$, M = 385.46, monoclinic space group $P2_1/n$; a = 13.846(5), b = 8.562(3), c = 17.283(6) Å, β = 104.959(9) °, V = 1979.4(11) Å^3, Z = 4, D_C = 1.293 g cm^{-3}, R = 0.0475, wR = 0.1140 for 3560 data with $I > 2\sigma(I)$ and 262 parameters. Data were recorded at 93 K using Mo Kα radiation and the structure was solved by direct methods and refined using full-matrix least squares analysis.

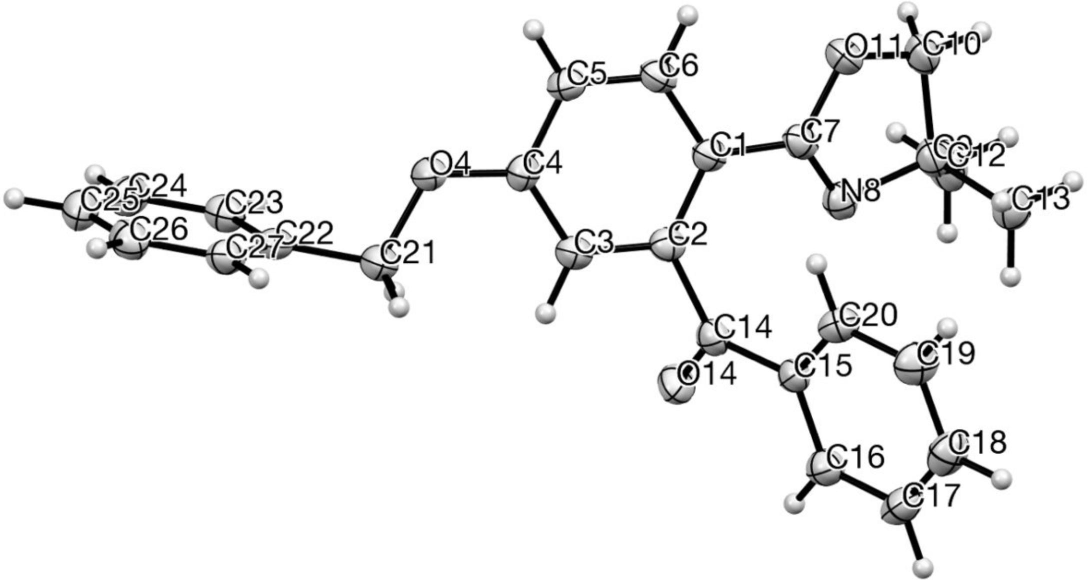

Figure 1. Molecular structure of **4** (ORTEP diagram at 50% level)

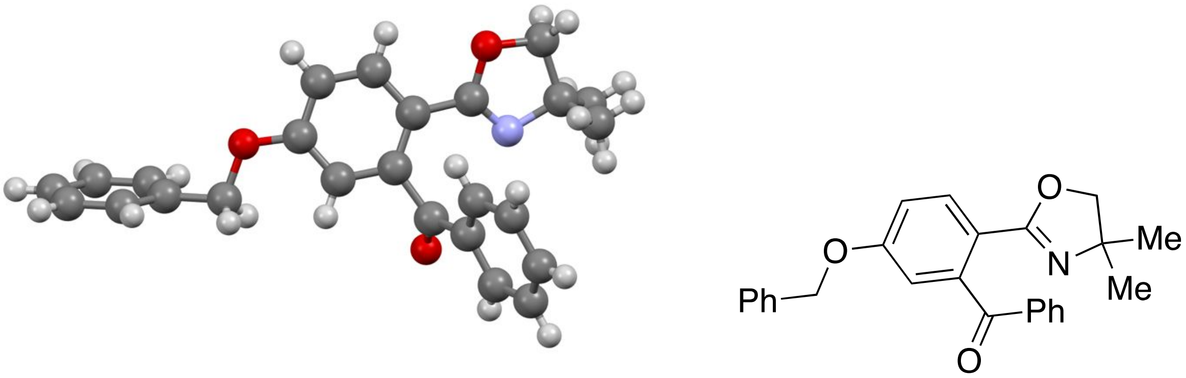

Figure 2. Ball and stick representation and Chemdraw showing orientation.

The following crystal data were obtained: **27** (CCDC No. 2105491)
$C_{14}H_{19}NO_2$, M = 233.31, colourless prism, crystal dimensions 0.20 × 0.05 × 0.05 mm, triclinic, space group $P\bar{1}$, a = 8.8749(13), b = 12.0133(19), c = 12.2118(18) Å, α = 88.375(7), β = 80.778(7), γ = 84.713(9)°, V = 1279.6(3) Å^3, Z = 4, D_c = 1.211 Mg m^{-3}, T = 93 K, R = 0.0360, R_W = 0.1109 for 3866 reflections with I>2σ(I) and 315 variables. Data were collected using graphite monochromated Mo-Kα radiation, λ = 0.71075 Å and the structure was solved by direct methods and refined using full-matrix least squares analysis.

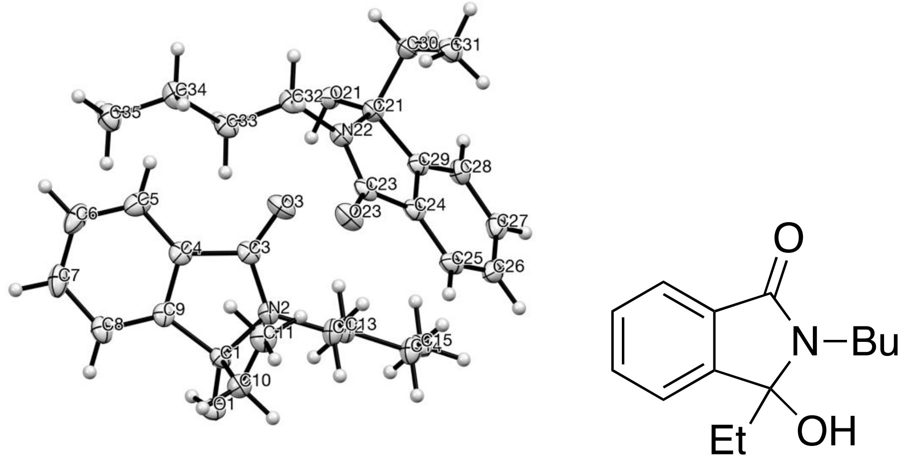

Figure 3. Molecular structure of the two independent molecules of **27** showing the key O(21)–H……O=C(3) hydrogen bonding interaction(ORTEP diagram at 50% level)

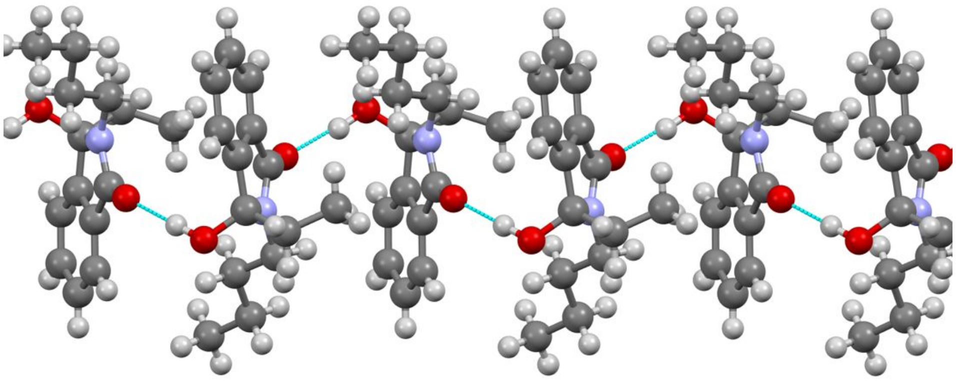

Donor	H	Acceptor	D...A	D-H	H...A	D-H...A
O1	H1	O23	2.7056(13) Å	0.975(15) Å	1.736(15) Å	172.6(21)°
O21	H21	O3	2.6944(12) Å	0.971(11) Å	1.725 (11) Å	177.0(13)°

Figure 4. Hydrogen bonding interactions between molecules of **27**

The following crystal data were obtained: **30c** (CCDC No. 2105493)
$C_{19}H_{23}NO_2$, M = 297.40, colourless prism, crystal dimensions 0.20 × 0.20 × 0.20 mm, monoclinic, space group $P2_1/c$, a = 17.719(15), b = 7.481(5), c = 13.0952(10) Å, β = 111.570(18)°, V = 1614.3(18) Å^3, Z = 4, D_c = 1.224 Mg m^{-3}, T = 93 K, R = 0.1001, R_W = 0.2657 for 2064 reflections with I>2σ(I) and 207 variables. Data were collected using graphite monochromated Mo-Kα radiation, λ = 0.71075 Å and the structure was solved by direct methods and refined using full-matrix least squares analysis.

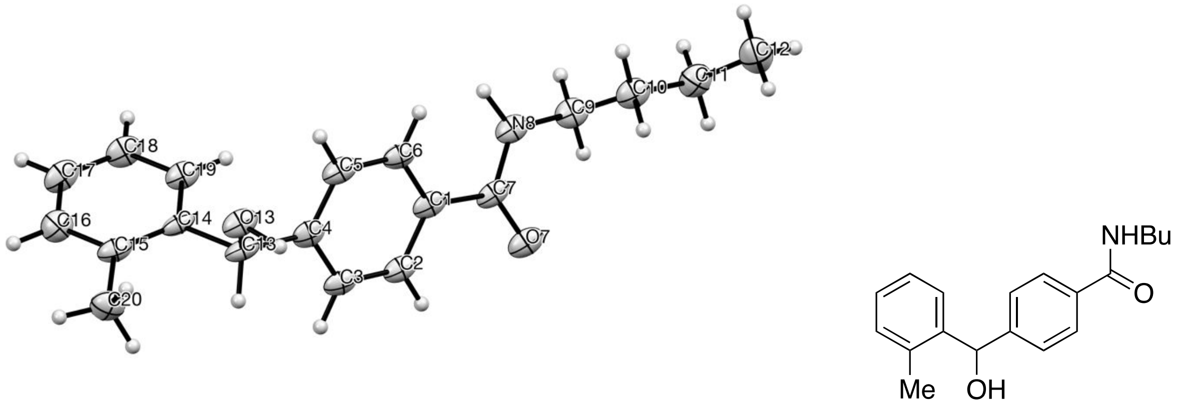

Figure 5. Molecular structure of **30c** (ORTEP diagram at 50% level)

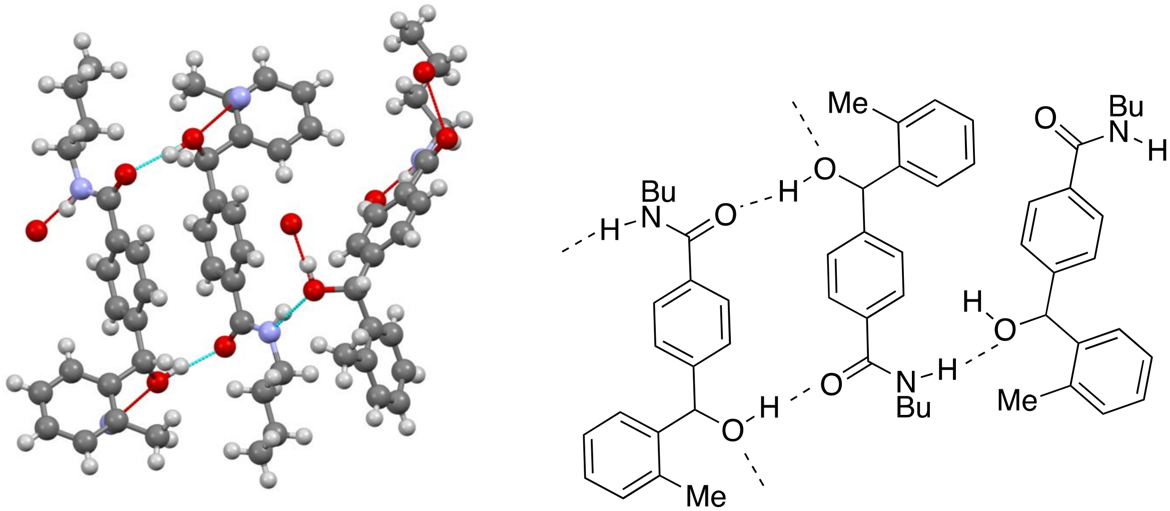

Donor	H	Acceptor	D...A	D-H	H...A	D-H...A
O13	H13	O7	2.673(4) Å	0.98(4) Å	1.70(5) Å	170(4)°
N8	H8	O13	2.874(5) Å	0.979(15) Å	1.95 (3) Å	157(4)°

Figure 6. Hydrogen bonding interactions between molecules of **30c**

The following crystal data were obtained: **38a** (CCDC No. 2105492)

$C_{18}H_{21}NO_2$, M = 283.37, colourless prism, crystal dimensions 0.05 × 0.05 × 0.03 mm, monoclinic, space group $P2_1/c$, a = 15.7001(7), b = 8.4768(4), c = 11.5571(5) Å, β = 90.258(4)°, V = 1538.08(12) Å^3, Z = 4, D_c = 1.224 Mg m^{-3}, T =125 K, R = 0.0595, R_W = 0.1662 for 2550 reflections with I>2σ(I) and 199 variables. Data were collected using graphite monochromated Cu-Kα radiation, λ = 1.54184 Å and the structure was solved by direct methods and refined using full-matrix least squares analysis.

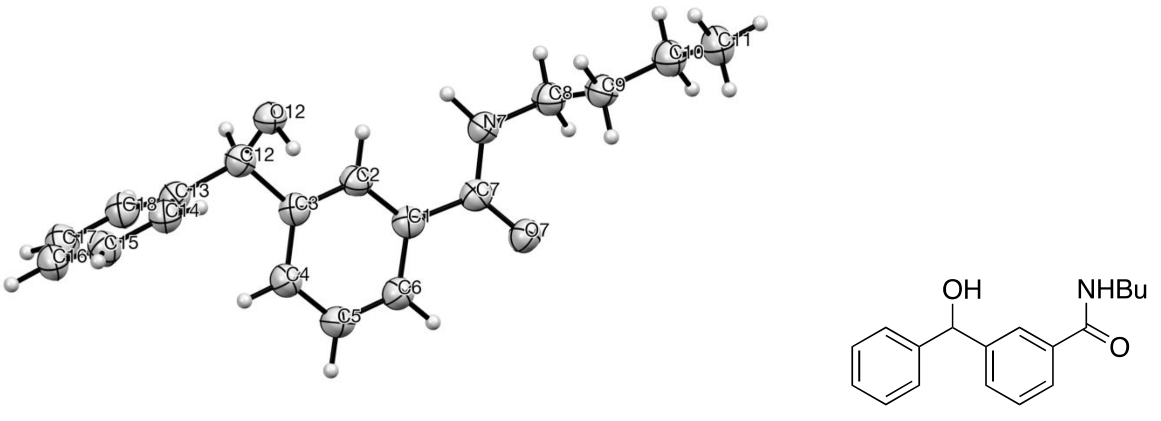

Figure 7. Molecular structure of **38a** (ORTEP diagram at 50% level)

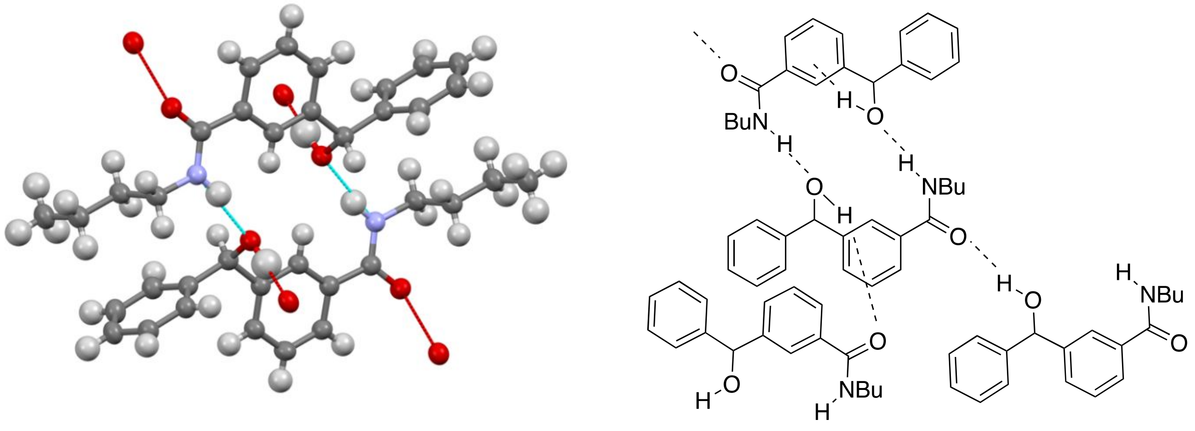

Donor	H	Acceptor	D...A	D-H	H...A	D-H...A
O12	H12	O7	2.7046(18) Å	0.980(8) Å	1.742(10) Å	167(2)°
N7	H7	O12	2.8947(18) Å	0.976(19) Å	1.936 (18) Å	167.0(17)°

Figure 8. Hydrogen bonding interactions between molecules of **38a**
